# THE CHANGING PACE OF INSULAR LIFE: 5000 YEARS OF MICROEVOLUTION IN THE ORKNEY VOLE *(MICROTUS ARVALIS ORCADENSIS)*

**DOI:** 10.1111/evo.12476

**Published:** 2014-07-29

**Authors:** Thomas Cucchi, Ross Barnett, Natália Martínková, Sabrina Renaud, Elodie Renvoisé, Allowen Evin, Alison Sheridan, Ingrid Mainland, Caroline Wickham‐Jones, Christelle Tougard, Jean Pierre Quéré, Michel Pascal, Marine Pascal, Gerald Heckel, Paul O'Higgins, Jeremy B. Searle, Keith M. Dobney

**Affiliations:** ^1^ CNRS‐Muséum National d’Histoire NaturelleUMR 7209, Archéoozoologie, histoire des sociétés humaines et de peuplements animaux, 55 rue Buffon75005ParisFrance; ^2^ Department of ArchaeologyUniversity of AberdeenSt. Mary's, Elphinstone RoadAberdeenUnited Kingdom; ^3^ Durham Evolution and Ancient DNADepartment of ArchaeologyDurham UniversitySouth Road, Durham DH1 3LEUnited Kingdom; ^4^ Institute of Vertebrate BiologyAcademy of Sciences of the Czech Republicv.v.i., Květná 8, 60365 BrnoCzech Republic; ^5^ Institute of Biostatistics and AnalysesMasaryk UniversityKamenice 3, 62500 BrnoCzech Republic; ^6^ Laboratoire de Biométrie et Biologie EvolutiveUMR 5558, CNRS, Université Lyon 169622VilleurbanneFrance; ^7^ Institute of BiotechnologyUniversity of HelsinkiP.O. Box 56 (Viikinkaari 9)FIN‐00014HelsinkiFinland; ^8^ National Museums ScotlandChambers Street, Edinburgh EH1 1JFUnited Kingdom; ^9^ Department of ArchaeologyUniversity of the Highlands and IslandsOrkney CollegeKirkwallOrkneyKW15 1LXUnited Kingdom; ^10^ Institut des Sciences de l'Evolution de MontpellierUMR CNRS 5554 and UMR IRD 226, Université Montpellier II, Place Eugène Bataillon, CC065, 34095 Montpellier cedex 05France; ^11^ UMR CBGP (Inra/Ird/Cirad /Montpellier SupAgro)INRACampus international de BaillarguetCS 30016F‐34988 Montferrier‐sur‐Lez cedexFrance; ^12^ INRAUMR 0985 EIBCampus de Beaulieu F‐35000 Rennes CedexFrance; ^13^ Computational and Molecular Population Genetics (CMPG)Institute of Ecology and EvolutionUniversity of BernCH3012BernSwitzerland; ^14^ Swiss Institute of BioinformaticsGenopodeCH 1015LausanneSwitzerland; ^15^ Centre for Anatomical and Human SciencesHull York Medical SchoolThe University of YorkHeslingtonYork YO10 5DDUnited Kingdom; ^16^ Department of Ecology and Evolutionary BiologyCorson Hall, Cornell UniversityIthacaNew York14853‐2701

**Keywords:** Dispersal, evolutionary rate, geometric morphometrics, island evolution, tooth shape, zooarchaeology

## Abstract

Island evolution may be expected to involve fast initial morphological divergence followed by stasis. We tested this model using the dental phenotype of modern and ancient common voles (*Microtus arvalis*), introduced onto the Orkney archipelago (Scotland) from continental Europe some 5000 years ago. First, we investigated phenotypic divergence of Orkney and continental European populations and assessed climatic influences. Second, phenotypic differentiation among Orkney populations was tested against geography, time, and neutral genetic patterns. Finally, we examined evolutionary change along a time series for the Orkney Mainland. Molar gigantism and anterior‐lobe hypertrophy evolved rapidly in Orkney voles following introduction, without any transitional forms detected. Founder events and adaptation appear to explain this initial rapid evolution. Idiosyncrasy in dental features among different island populations of Orkney voles is also likely the result of local founder events following Neolithic translocation around the archipelago. However, against our initial expectations, a second marked phenotypic shift occurred between the 4th and 12th centuries AD, associated with increased pastoral farming and introduction of competitors (mice and rats) and terrestrial predators (foxes and cats). These results indicate that human agency can generate a more complex pattern of morphological evolution than might be expected in island rodents.

Alien species on islands, whether they arrive with humans or by natural dispersal (Williamson 1981), can show rapid changes, in which evolutionary and ecological processes overlap (Lambrinos 2004; Bradshaw and Holzapfel 2006; Kinnison and Hairston 2007). Both founder events and adaptation to specific insular conditions should promote rapid initial divergence, with subsequent stasis once the population reaches a demographic equilibrium and its local ecological optimum (Sondaar 2000; Millien 2006; Nagorsen and Cardini 2009). However, many islands suffer human‐induced disturbances (destruction of habitat, introduction of species) that may also promote rapid phenotypic changes (Palumbi 2001; Price et al. 2003; Pigliucci et al. 2006; Carroll et al. 2007; Ghalambor et al. 2007; Hendry et al. 2008). In Europe the bioarchaeological record reveals that island systems were subjected to substantial anthropogenic disturbance through the early to middle Holocene, when the first (Neolithic) farmers spread across Eurasia (Cherry 1990; Blondel and Vigne 1993; Schüle 1993; Vigne 1999). A phenotypic stasis for introduced animals in these insular contexts is, therefore, unlikely. Instead, many phenotypic changes should be expected to maintain fitness in a dynamic environment in which directions of selection driven by human activities fluctuated.

Testing these ideas requires documenting phenotypic change through time. However, most studies of phenotypic evolution following island colonization make inferences solely from present‐day populations (e.g., Clegg et al. 2008) or on the basis of laboratory (e.g., Templeton 1996) or field experiments (e.g., Losos et al. 1997). In their study of natural colonization, it is notable that Nargosen and Cardini (2009) did compare 30 subfossil specimens of an endemic insular marmot (*Marmota vancouverensis*) with its extant insular and continental relatives. They found that most of the divergence occurred soon after the island became isolated during the Pleistocene (with subsequent morphological stasis continuing to the present day), supporting the ecological “optimum model.” However, this model remains largely untested in situations in which human introduction and human impact to the environment occurred, a fact largely due to the lack of continuous subfossil records documenting each step of insular evolution.

In this article, our objective is to examine morphological change through time in populations of the Orkney vole, *Microtus arvalis orcadensis* (Major 1905)—an endemic subspecies of the common vole *Microtus arvalis* (Pallas 1778)—introduced to the Orkney archipelago (Fig. 1) by Neolithic farmers around 5000 years ago, from a source outside the British Isles (Martínková et al. 2013). Large samples of archaeological Orkney voles, as well as good palaeo‐environmental records revealing anthropization of the Orkney archipelago (Bunting 1994, 1996), provided an important opportunity to investigate the pace of evolutionary change in this insular rodent over the last 5000 years, within the context of an island environment impacted by humans.

**Figure 1:**
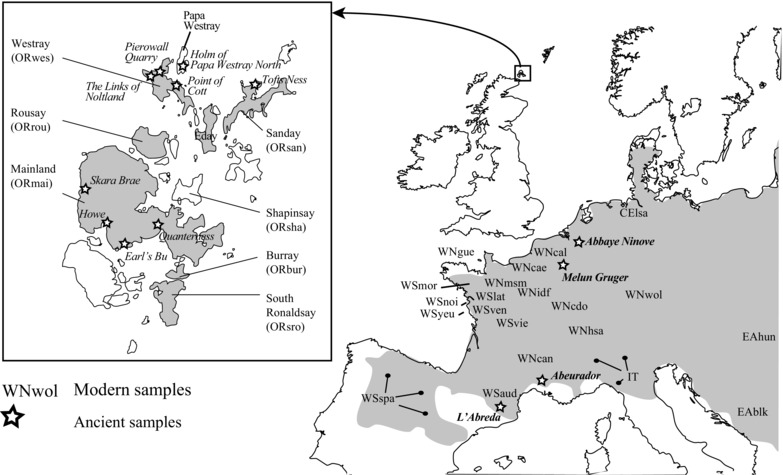
Localization of the modern samples (abbreviated group names) and ancient samples (stars with site names) of *Microtus arvalis* in continental Europe and Orkney (insert) distribution area of the species (gray shading; Shenbrot and Krasnov 2005).

Present‐day Orkney voles are morphologically characterized by a large body size—twice that of their continental European cousins—and differing in many other skeletal traits and pelage (Berry 1996). Considerable morphological diversity has been described among the different island populations of the archipelago (Fig. 1). For instance, those from islands in the northern part of the archipelago (Westray and Sanday) show a darker pelage and a molar morphology different to those from the main island (Mainland) and the South Isles (Corbet 1964). Divergence within Orkney vole populations has been attributed to either founding events (Berry 1996) or fast adaptive radiation (Corbet 1986). A recent study has also revealed higher genetic diversity in Orkney voles from Mainland (compared to those of the outer isles). These data suggest an initial introduction of voles to Mainland, from where successive founding events occurred in other islands of the group through further human‐mediated dispersal (Martínková et al. 2013).

To investigate microevolution in Orkney voles through time, we used molar morphology as a phenotypic marker. Vole teeth are the most abundant and diagnostic element in the fossil and subfossil record, and their complex form is evolutionary tractable (Guthrie 1965; Renvoisé et al. 2012). First we examined the extent of molar size and shape divergence of Orkney voles from their continental counterparts and from other insular populations. Increase in molar size (as a marker for body size increase) is expected in insular populations of rodents according to the island rule (Foster 1964; van Valen 1973), whereas niche widening and reduction of predation and interspecific competition (together with founder events and drift in small, isolated populations) may induce rapid molar shape change (Renaud et al. 2011, 2013). To test the influence of climatic gradients (Piras et al. 2009; McGuire 2010) on the phenotype of *M. arvalis*, we assessed how much molar size and shape covaries with climate among populations from Western Europe—‐including Orkney (Fig. 1).

We then investigated morphological divergence among Orkney populations since their introduction. To test the expectation of greater divergence on more remote and smaller islands (Renaud and Millien 2001; Millien 2011), we examined phenotypic variation in the context of interisland distances, as well as island areas. The suggestion of dispersal throughout the archipelago from a source population on Mainland (Martínková et al. 2013) was tested using the correlation between molar shape and geographic distances calculated from Mainland. Further, the hypothesis of stochastic processes of divergence by, for example, founding events and genetic drift (Berry 1996) was assessed by testing (i) the influence of the time elapsed since introduction using direct radiocarbon age of some of the samples and (ii) the covariance between dental morphology and neutral molecular markers from the same modern and ancient individuals from Orkney. A significant correlation between morphological and neutral genetic variation is expected if random processes underlie phenotypic diversification in Orkney voles since their introduction (Clegg et al. 2002). Finally, because of the detailed archaeological record on Mainland Orkney, we were able to examine the pace of evolutionary change in molar shape for a single island, along a time series spanning the 5000 years since the introduction of *M. arvalis* to the Orkney archipelago.

## Material and Methods

### MODERN AND ANCIENT SAMPLES

A total of 853 *M. arvalis* first lower molars (M_1_) were measured: 582 modern (Table 1) and 271 ancient (Table 2). The modern dataset includes 378 specimens from across mainland continental Europe, 73 specimens from three islands off the coast of France (Guernsey, Yeu, and Noirmoutier), and 131 *M. arvalis orcadensis* from seven Orkney Islands (Fig. 1). The modern continental dataset covers several (previously identified) mitochondrial DNA (mtDNA) haplogroups of *M. arvalis* (Table 1): the Western‐North (WN), Western‐South (WS), Italian (IT), Eastern (EA), and Central (CE) clades (and the Orkney [OR] clade within the WN clade), based either on direct genotyping or on the geographic distribution of evolutionary lineages (Haynes et al. 2003; Heckel et al. 2005; Tougard et al. 2008; Braaker and Heckel 2009; Martínková et al. 2013). The three coastal island populations of Guernsey, Yeu, and Noirmoutier are considered here as subspecies and potential relic populations from the Last Glacial Maximum (LGM, Berry and Rose 1975), although no fossil evidence exists to support this assumption. The Guernsey voles belong to the same WN haplogroup as the Orkney vole but with very distinctive mtDNA haplotypes (Martínková et al. 2013), whereas Noirmoutier and Yeu voles belong to the WS haplotype lineage.

**Table 1:** Modern samples of *Microtus arvalis*

Country	Localities	MtDNA Lineages	Code	*N*
France	Various (M)	*M. agrestis*		29
United Kingdom (I)	Orkney, Burray (T)	Orkney	ORbur	2 (2)
	Orkney, Mainland (T)	Orkney	ORmai	46 (19)
	Orkney, Rousay (T)	Orkney	ORrou	10 (1)
	Orkney, Sanday (T)	Orkney	ORsan	20 (3)
	Orkney, Shapinsay (T)	Orkney	ORsha	3
	Orkney, South Ronaldsay (T)	Orkney	ORsro	17 (15)
	Orkney, Westray (T)	Orkney	ORwes	33 (10)
	Guernsey (M)	Western‐North	WNgue	35
France (I)	Noirmoutier (T)	Western‐South	WSnoi	30
	Yeu (T)	Western‐South	WSyeu	8
France (C)	Morbihan (T)	Western‐South	WSmor	13
	Vendée (T)	Western‐South	WSven	19
	Loire Atlantique (M)	Western‐South	WSlat	12
	Mont Saint‐Michel (T)	Western‐North	WNmsm	8
	Caen (T)	Western‐North	WNcae	29
	Calais (T)	Western‐North	WNcal	23
	Île‐de‐France (T)	Western‐North	WNidf	17
	Vienne (T)	Western‐North	WNvie	13
	Cantal (M)	Western‐North	WNcan	27
	Côte‐D'or (M)	Western‐North	WNcdo	24
	Aude (T)	Western‐South	WSaud	30
	Haute‐Savoie (M)	Western‐North	WNhsa	19
Spain (C)	Various (M)	Western‐South	WSspa	15
Germany (C)	Wolfach (T)	Western‐North	WNwol	17
	Lower Saxony (M)	Central	CElsa	23
Italy (C)	Various (M)	Italian	IT	6
Balkan countries (C)	Various (M)	Eastern	EAblk	30
Hungary (C)	Various (M)	Eastern	EAhun	24

I, insular samples; C, continental samples; M, material from museum collections; T, trapped animals (genotype available); *N*, number of molars included in the morphometric study with, in parentheses, the number of specimens with mt‐cytb sequence (see Supporting Information 4) .

**Table 2:** Ancient samples of *Microtus arvalis*

Location	Site	Context	Period	Chronology	Group	*N*
Orkney Mainland	Quanterness	Cairn	Neolithic	Late 4th mill. BC	Qu	29
	Skara Brae	Village	Neolithic phase 0 Neolithic phase 1	3360–3160 BC 2910–2820 BC	SB1	17
			Abandonment	2850 BC	SBt	12
			Neolithic phase 2	2850–2400 BC	SB2	10
	Howe	Broch	Iron Age	4th–7th c. AD	Ho	25 (3)
	Earl's Bu	Viking building	Norse	11th–12th c. AD	EB	15
Orkney Westray	Point of Cott	Cairn	Neolithic	c3500–2800 BC	PC	27 (3)
	Holm of Papa Westray North	Cairn	Neolithic	c3500, c3000, c2600 BC	HW	20 (3)
	Pierowall Quarry	Cairn	Neolithic	c2900–2600 BC	PQ1	10 (2)
					PQ2	15
					PQ4	13
	The Links of Noltland	Village	Neolithic	c2900–2600 BC	LN	22
Orkney Sanday	Tofts Ness	Village	Late Bronze Age	c1000 BC	TN	22
Spain	L'Abreda	Cave	Solutrean	22,000–17,000 BP	Abr1	8
France	L'Abeurador	Rock shelter	Mesolithic	8000–6000 BC	Abe	11
	Melun Grüber	Village	Iron Age	1st–4th c. AD	MG	7
Belgium	Abbaye Ninove	Building	Medieval	12th c. AD	AN	8

Period: Chronocultural context of the vole samples. Chronology: Time frame of the context in which samples have been collected (BP, Before Present; BC, Before Christ; AD, Anno Domini; c., century). Group: Sample codes used throughout this study. *N*: number of molars considered in the study with, in parentheses, the number of specimens with mt‐cytb sequence (see Supporting Information 4).

The archaeological samples included 237 *M. a. orcadensis* from nine sites in Orkney and 34 *M. arvalis* from four continental European sites (Fig. 1, Table 2). The *M. arvalis* subfossils from continental Europe required preliminary identification (see *Statistical analyses*), which was unnecessary for Orkney voles. The subfossil Orkney voles were sampled from archaeological sites on Mainland, Westray, Papa Westray, and Sanday (Fig. 1). Mainland samples span the longest temporal transect; from Neolithic Skara Brae (SB, Clarke 1976) and Quanterness (Qu, Renfrew 1979)—dating between 3500 and 2500 BC, the Iron Age broch at Howe (Ho, Smith and Carter 1994)—dating between 4th and 7th centuries AD, and Medieval Earl's Bu (EB, Batey et al. 1993)—dating between the 11th and 12th centuries AD. Northern isles samples include those from Westray, Papa Westray, and Sanday. From the former two islands, specimens have been recovered from four Neolithic contexts; the chambered tombs at Point of Cott (Barber 1997), Pierowall Quarry (Sharples 1984), Holm of Papa Westray North (Ritchie 2009), and the settlement at the Links of Noltland (Clarke and Sharples 1985), whereas on Sanday, samples have been collected from the Late Bronze Age phase of occupation at Tofts Ness (Dockrill et al. 1994; Simpson and Dockrill 1996).

Ancient samples from continental Europe (Table 2) include those from the Upper Pleistocene cave deposits from L'Abreda (M. S. Segui, pers. comm.) in Spain, the Mesolithic deposits of L'Abeurador cave (Marquet 1993) in France, the Iron Age settlement of Melun Grüber (Mistrot 2000) in France, and the Late Medieval Abbaye Ninove (Wouters and Peersman 1994; A. Ervynck, pers. comm.) in Belgium.

#### Geometric morphometrics of the first lower molar

**Figure 2:**
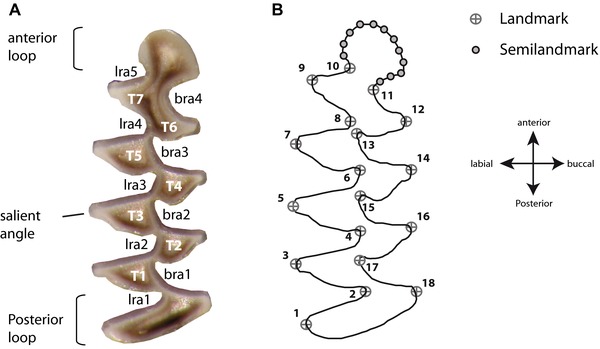
(A) Occlusal surface of *Microtus arvalis* right first lower molar (M_1_): T, triangle; bra, buccal reentrant angle; lra, lingual reentrant angle. (B) Position of the 18 landmarks and 12 semilandmarks.

To quantify molar size and shape, we used a two‐dimensional geometric morphometric approach (Zelditch et al. 2012; Adams et al. 2013). The form of the occlusal surface of the M_1_ in *M. arvalis* (Fig. 2A) was quantified using 18 landmarks positioned at the maximum curvature of the salient and reentrant angles of the posterior loop and the triangle cusps on the buccal and lingual sides of the M_1_ (Fig. 2B). The smooth curve of the anterior loop (Fig. 2A), which is lacking landmarks, was further quantified using 12 equidistant sliding semilandmarks (Fig. 2B) to extend the landmark‐based statistics to curves (Mitteroecker and Gunz 2009). The Cartesian coordinates of the landmarks and semilandmarks were captured using TPSdig2 v.2.16 (Rohlf 2010a).

This molar shape dataset (Supporting Information 1) was analyzed using a generalized Procrustes analysis (GPA, (Rohlf and Slice 1990). Using this procedure, information on position, scale, and orientation are removed from the Cartesian coordinates configuration and the semilandmarks are forced to slide on a tangent according to the Bending Energy algorithm (Bookstein 1997). The resulting Procrustes shape coordinates (Supporting Information 1) from this superimposition were used as shape variables for subsequent statistical analyses. Overall M_1_ size was measured by the centroid size (CS), that is, the square root of the sum of the squared distances between each point and the centroid of the configuration (Supporting Information 1). GPA was performed with TPSrelw v. 1.49 (Rohlf 2010b).

#### Acquisition of climatic, geographic, temporal and genetic data

Geoclimatic data—i.e., longitude, latitude, altitude and annual mean monthly, maximal monthly, minimal monthly values of precipitation and temperature (Supporting Information 2)—were collected throughout the distribution of the modern vole dataset (Fig. 1) from weather stations between 1960 and 1995 (World Meteorological Organization [WMO] stations; sources: National Oceanic and Atmospheric Administration [NOAA], National Climatic Data Center [NCDC], and Global Climate Perspectives System [GCPS]). Geographic characteristics (Supporting Information 3) of the different Orkney Islands, such as their size (km^2^), and their shortest interisland linear distances (km), were acquired from the EDINA Digimap collections software (Edinburgh University).

Dating of Orkney vole specimens collected from the different archaeological sites was ascertained through direct ^14^C assays using Accelerator Mass Spectrometry (AMS) on hemimandibles (Martínková et al. 2013). The mean of the calibrated ^14^C age values in years before present (BP) for each archaeological site was used as the radiocarbon age of the different Orkney vole samples in our dataset. Vole samples without direct ^14^C dating (such as those from the Links of Noltland and Tofts Ness) have been assigned ages in calendar years BP estimated from ^14^C dating of the archaeological context from which they have been collected.

We could match 61 modern and ancient specimens from Orkney for combined geometric morphometric and genetic analyses (Supporting Information 4), using the molar and mtDNA sequence data (*mt‐cytb*, Martínková et al. 2013). Modern samples that were morphologically and genetically analyzed were from locations widely distributed over the Orkney archipelago (Table 1), whereas ancient specimens similarly analyzed were from Neolithic Westray (Orkney) and Iron Age Mainland (Orkney, see Table 2). The molecular distance between individuals (Supporting Information 5) was generated using the Kimura 2‐parameter (K2P) model in PAUP* (Swofford 2002).

### STATISTICAL METHODS

#### Taxonomic identification of archaeological specimens

Two sympatric species of *Microtus* (*arvalis* and *agrestis*) occur in continental Europe and display very similar tooth morphology (Chaline and Mein 1979) that can result in misidentification (Hall and Yalden 1978). Therefore, species identification of archaeological voles from continental Europe was performed with a predictive approach based on a linear discriminant analysis (LDA) using a modern comparative sample of 31 *M. arvalis* and 29 *M. agrestis* from France (Table 1). Of the available 26 principal components (PC) of shape, the first nine were used to classify specimens following dimensionality reduction based on cross‐validation percentages (leave‐one‐out procedure) of correct reassignments (Baylac and Friess 2005; Sheets et al. 2006). This classification was based on the generalized distance (*D* ^2^) and the associated probability of group membership between the archaeological specimen and the centroid of both reference taxonomic groups. Only ancient specimens associated to *M. arvalis* with a predictive probability above 0.9 have been included in the study.

#### Differentiation of molar size and shape in M. arvalis

Molar size variation was assessed among samples by an analysis of variance (ANOVA) on the log‐transformed CS, with subsequent pairwise *t*‐tests between samples using a Bonferroni correction. The molar shape variation among samples was tested using multivariate analysis of variance (MANOVA) and measured by Procrustes distances (Euclidian distances between two configurations of Procrustes coordinates) using permutation tests (10,000 runs). The main axes of variation are displayed with a principal components analysis (PCA). The visualization of molar shape‐change along the principal axes is depicted by the magnitude of change in Procrustes distances.

#### Covariates of molar size and shape differentiation

To assess the influence of climatic factors (latitude, longitude, altitude, precipitation, temperature), island size (area), and time elapsed since introduction on molar size and shape among Orkney vole populations, we used univariate (CS) and multivariate (Procrustes coordinates) linear regressions with permutation tests (10,000 runs). The influence of geographic distance from Mainland Orkney on shape divergence among other Orkney populations was tested with a linear regression model and Pearson's *R* correlation test between the paired geographic and shape distances of each modern sample from Mainland, respectively measured in kilometers and Procrustes distances.

The size‐related shape changes (allometry) among samples were tested with a multivariate analysis of covariance (MANCOVA) with Procrustes coordinates as dependent variables and log CS as a covariate. The lack of a significant interaction between the shape and size differences among samples would signal a lack of influence of size over the patterns of shape divergence, interpreted as common allometric trajectories among samples. The amount of shape change explained by size difference was assessed by multivariate regression with Procrustes coordinates as dependent variables and the log‐transformed CS as the independent variable. To test the null hypothesis of independence, permutation tests (1000 runs) were carried out.

The influence of the interisland and genetic distances over molar shape differentiation among the Orkney vole populations was tested using PROTEST, since it has proven to perform better than the Mantel test (Jackson 1995; Peres‐Neto and Jackson 2001). We used a principal coordinate analysis (PCoA) to convert distances matrices into a Cartesian coordinate system (Gower 1966) and 1000 random permutations to test for the significance of the correlation index (Monte‐Carlo, Jackson 1995).

#### Allochronic changes along the Orkney Mainland time series

The evolution of molar shape changes in Mainland Orkney voles, from their Neolithic introduction until the present time, is an allochronic design (Hendry and Kinisson 1999), involving Mainland Orkney populations composed of six assemblages representing a time series of four broad chronological periods: Neolithic (Qu, SB1 and SB2), Iron Age (Ho), Medieval (EB), and present‐day (Modern Mainland), with varying time intervals between the successive samples. Evolutionary rates are dependent on the time interval over which they are measured, with shorter time intervals tending to lead to higher observed evolutionary rates, because short‐term fluctuations are buffered over long time periods (Gingerich 1983). The limited number of successive samples and the uneven time intervals separating them, renders it difficult to calculate evolutionary rates accurately. Instead, morphological distances among successive samples were calculated. Mahalanobis's distances (*D*, Mahalanobis 1936) were chosen, because expressing among‐sample difference relative to within‐sample variance provides a multivariate analog to the morphological distances used to estimate evolutionary rate of complex traits in *haldanes* (Lerman 1965; Cherry et al. 1982), appropriate for geometric morphometric analyses (Arnegard et al. 2010; Carlson et al. 2011; Adams 2014). The calculation of *D* was performed based on 32 PCs, according to a procedure of dimensionality reduction described previously (*Taxonomic identification of archaeological specimens*).

Size (log CS), shape (estimated by the second axis of the analysis focused on Orkney) and morphological distances (*D*) were expressed against time to provide a comprehensive picture of phenotypic evolutionary change in Orkney following initial divergence.

PCA, multivariate regressions, and visualizations of shape change were performed with MorphoJ (Klingenberg 2010). Linear regressions, PCA, LDA, ANOVA, MANOVA/MANCOVA, and PROTEST were carried out with R version 2.13.0 (R development Core Team) with the ade4 package (Dray and Dufour 2007) and the library Rmorph (Baylac 2012).

## Results

### DIVERGENCE OF ORKNEY VOLES FROM THEIR CONTINENTAL AND OTHER INSULAR RELATIVES: MOLAR SIZE, SHAPE, AND ALLOMETRY

**Figure 3:**
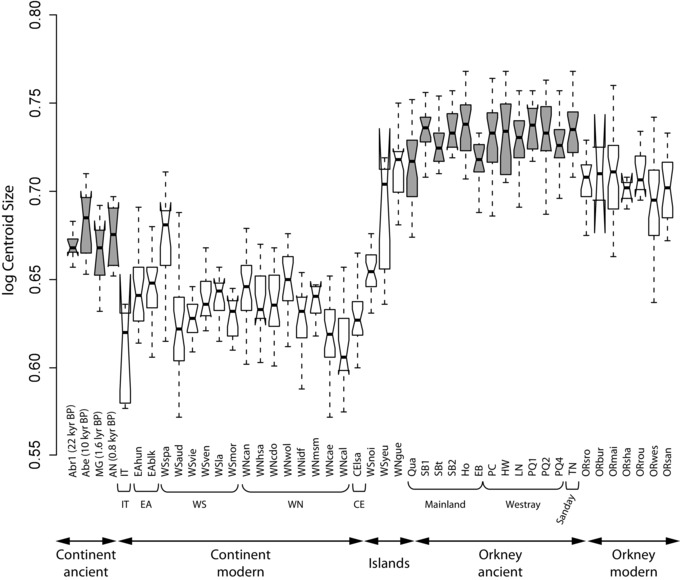
Variation in molar size (log‐transformed centroid size) in ancient (gray boxes) and modern (open boxes) *Microtus arvalis* samples (see Fig. 1 for localization and Tables 1 and 2 for sample details). Left, modern continental European samples include specimens grouped according to their genetic lineage (IT, Italian; EA, Eastern; WS, Western‐South; WN, Western‐North; CE, Central) and insular origin (Yeu, WSyeu; Noirmoutier, WSnoi; Guernsey, WNgue). Right, ancient Orkney specimens are grouped according to their geographic location in the archipelago.

Ancient and modern Orkney voles have larger molars than ancient and modern continental and other insular populations of *M. arvalis* (*F* = 79.77, *P* < 0.0001), a tooth size only matched by the insular population from Guernsey (Fig. 3). Molar sizes in ancient voles are 5% larger than present‐day ones, both in continental Europe (*F* = 40.03, *P* < 0.0001) and Orkney (*F* = 10.11, *P* < 0.0001)—except in Spain where *M. arvalis* shows no reduction in molar size since the LGM. Despite significant differences (*F* = 49.06, *P* < 0.0001), the size range of *M. arvalis*’ molars remains stable, from the LGM (Spanish Abr1) till Medieval times (Belgium AN), and shows no evidence for the drastic size reduction manifested in current populations (Fig. 3). Modern Orkney voles display a molar size reduction of 5% compared to archaeological samples (Fig. 3, Supporting Information 6). However, only the medieval specimens from EB are significantly smaller than all the other archaeological samples from Orkney (Supporting Information 6), suggesting that in Orkney, a decrease in molar size occurred earlier than on the continent—i.e., between the 4th and 12th centuries AD.

Despite great variability of molar size across recent continental European samples (ANOVA, *F* = 30.83, *P* < 0.0001), tooth size does not correlate with genetic lineage (Fig. 3). Insular samples tend to have a larger M_1_ compared to their continental European counterparts (Supporting Information 6), with only Orkney and Guernsey (WNgue) displaying “gigantism” (Fig. 3). No significant size difference was found amongst modern Orkney populations according to pairwise *t*‐tests (Supporting Information 6).

**Figure 4:**
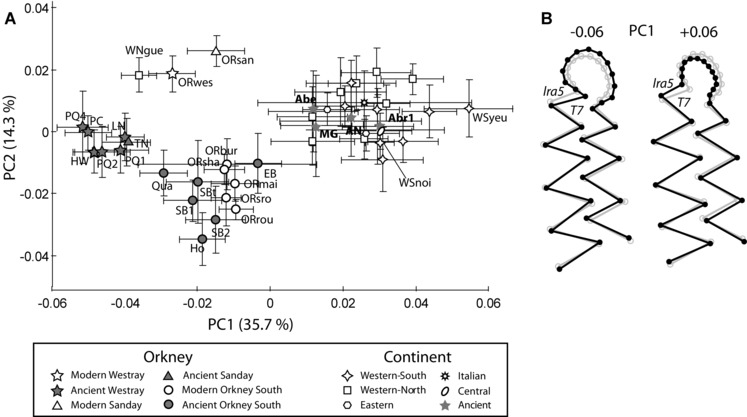
Molar shape differentiation of modern and ancient *Microtus arvalis* samples from continental Europe (islands included) and Orkney. (A) Scatter plot of the two first principal components of the morphometric analysis. Each symbol with its group code (Tables 1 and 2) corresponds to the mean value of a modern or ancient group, bracketed by the 95% confidence interval. (B) Molar shape change associated with PC1 is depicted with a wireframe graph connecting the landmarks and semilandmarks. The gray wireframe represents the mean shape and the black wireframe represents the shape changes along PC1 in negative (−0.06) and positive (+0.06) directions.

As with size, molar shape distinguishes Orkney and Guernsey voles from other *M. arvalis* (Fig. 4A). The main variation in *M. arvalis* molar shape corresponds to a broadening/narrowing of the M_1_ anterior loop associated with the closing/narrowing of reentrant angle (lra5) between triangle 7 and the anterior loop (Fig. 4B). The shape disparity among Orkney voles contrasts with the conservatism observed in continental *M. arvalis* over time, where (despite significant variation among samples [*Pillai's* = 6.434; *F* = 2.405; *P* < 0.0001]) no clear patterning emerges among genetic lineages or between ancient and modern samples (Fig. 4A). No intermediate phenotype can be observed between continental and Orkney voles, not even in the samples from northern coastal France and medieval Belgium (AN)—areas considered likely geographic sources of Orkney voles (Martínková et al. 2013).

Overall, there is no common signature for island populations, since Noirmoutier (WSnoi) and Yeu (WSyeu) lie within the range of variation of mainland European samples (Fig. 4A). Guernsey voles show aspects of shape that are close to voles from the northern Orkney Isles (Westray and Sanday) along PC1 and PC2. However, the shape of Orkney and Guernsey voles is highly differentiated (*Pillai's* = 3.432; *F* = 6.976; *P* < 0.0001; Procrustes distance: 0.049; *P* < 0.001).

**Figure 5:**
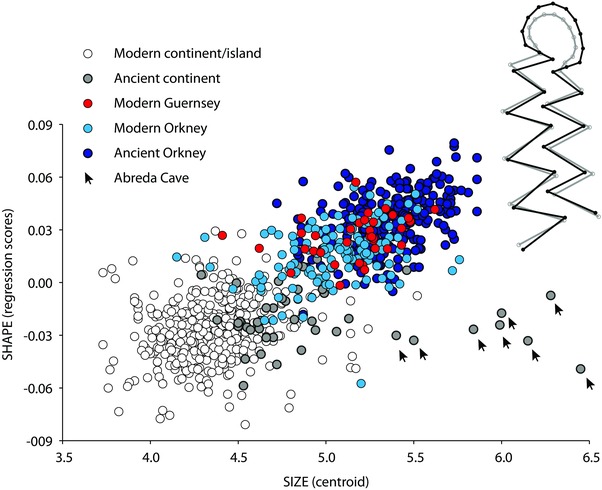
Allometric trend in modern and ancient *Microtus arvalis* estimated from the regression of Procrustes coordinates on centroid size. Insert: the gray lines and open circles represent the average shape and the black lines and circles represent the predicted shape for a centroid size increase of 2 mm.

What does differentiate the Orkney and the Guernsey populations of *M. arvalis* from all their continental (mainland and island) European relatives is the hypertrophy of the anterior loop of their molars. This peculiar trait is partly size‐related as suggested (first) by the relationship between the PC1 (Fig. 4) and the CS (*slope* = −10.449; *intercept* = 4.813; *r^2^* = 0.469; *permut. P* < 0.0001) and (second) by the significant allometric component explaining 20% of the shape variation (*permut. P* < 0.0001) mainly localized in the anterior part of the M_1_ (Fig. 5). However, a MANCOVA with tooth shape as a dependent factor showed significant effects of size (*Pillai's* = 0.4788; *F* = 10.4103, *P* < 0.0001), geographic groups (*Pillai's* = 8.3995; *F* = 2.6993, *P* < 0.0001), and size × group interaction (*Pillai's* = 4.1818; *F* = 1.2083, *P* < 0.0001). The latter indicates that the allometric pattern varies among samples.

### LOCAL DIVERGENCE AMONG ORKNEY VOLES

**Figure 6:**
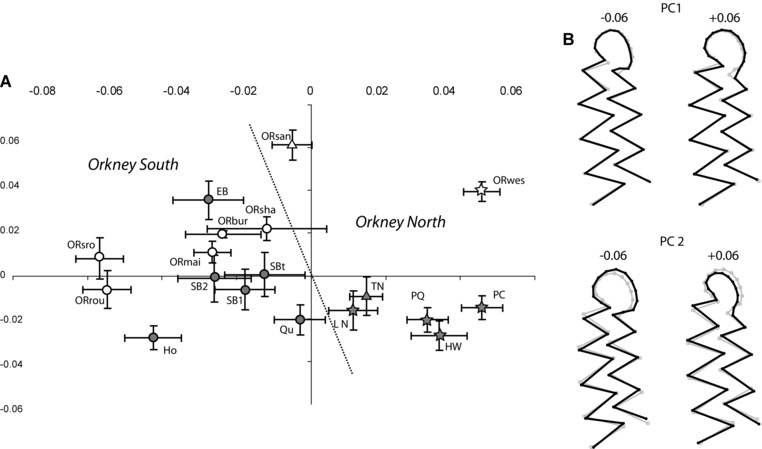
Molar shape variation in modern and ancient *Microtus arvalis* samples from Orkney. (A) Scatter plot of the two first principal components of the morphometric analysis. The modern samples are represented by empty symbols whereas the ancient samples are shown by gray‐filled circles. The dotted line separates northern from southern populations. (B) Molar shape change associated with PC1 and 2 is depicted by a wireframe graph connecting the landmarks and semilandmarks. The gray wireframe represents the mean shape and the black wireframes indicate the shape change of the PC score by 0.6 units in the positive and negative directions.

The diversification in modern and ancient Orkney voles (Fig. 6) seems to have been driven first by the geography of the archipelago (PC1 separating southern from northern populations) and second by the time elapsed since their introduction (as shown by variation along PC2). Voles from Mainland (ORmai) and its satellite islets of Shapinsay (ORsha), Burray (ORbur), South Ronaldsay (ORsro), and Rousay (ORrou) are phenotypically close compared to the highly divergent (*P* < 0.001) Westray (ORwes) and Sanday (ORsan) specimens (Fig. 6A). The molar shape differences associated with this geographic divergence are related to modifications in the buccal (bra4) and labial (lra5) reentrant angles of the sixth and seventh cusps, with a relatively smaller and less rounded anterior loop present in the southern isles populations (Fig. 6B).

Time elapsed since introduction appears to have contributed to the pattern of diversification in Orkney voles, according to the significant association between radiocarbon age of the ancient samples and their molar shape (*permut P*: 0.038)—predicting 22% of variation. Shape divergence over time, which has been more pronounced in the northern isles (Fig. 6A), is associated with a reduction of the anterior loop (Fig. 6B). On the Orkney Mainland, the temporal divergence is less substantial and suggests periods of relative morphological stability, contrasting with intervals of marked changes.

Part of the divergence in shape of Orkney voles since introduction is associated with a small (7.2%) but significant (*P* < 0.0001) allometric adjustment attributable to molar size reduction between ancient and extant populations (Fig. 5).

### CLIMATIC, GEOGRAPHIC AND GENETIC DETERMINANTS OF MORPHOLOGICAL DIVERGENCE

The climatic factors tested in relation to molar variation in the modern *M. arvalis* populations of our dataset (Tables 3 and 4) show that only size (not shape) is influenced within the geographic range studied. Thirty percent of the molar size variation is correlated with latitude, mean and maximum precipitation, and maximum temperature (Table 3) suggesting that *M. arvalis* molar size is influenced by the latitudinal gradient.

**Table 3:** A. Linear regression model testing for influence of climatic factors on centroid size. B. Multivariate regression model testing for influence of climatic factors on shape (Procrustes coordinates)

A	Slope	Error	Intercept	Error	*r* ^2^	Permutation *P*
Latitude	21.0740	8.0120	16.6440	12.0780	**0.30**	**0.0302** *
Longitude	0.0131	0.0272	4.5113	4.5113	0.01	0.6483
*T*_m_	−0.56663	0.89727	13.759	4.0787	0.01	0.6483
*T*_max_	−2.6312	1.0362	30.444	4.7101	**0.29**	**0.0327** *
*T*_min_	1.1388	1.0088	−0.67746	4.5857	0.07	0.2681
*P*_m_	14.625	5.3215	2.2146	24.19	**0.33**	**0.0128** *
*P*_max_	124.5200	58.1860	−86.9090	87.7180	**0.22**	**0.0460** *
*P*_min_	14.1480	19.4410	18.6460	21.8470	0.03	0.4767

B	Predicted Sum of Squares	Residual Sum of Squares	Percentage predicted	Permutation *P*
Latitude	0.001571	0.012390	11.25	0.0989
Longitude	0.001262	0.012699	9.04	0.1504
Altitude	0.000421	0.013540	3.01	0.0824
*T*_m_	0.001667	0.012294	11.94	0.0674
*T*_max_	0.001724	0.012237	12.35	0.0743
*T*_min_	0.007832	0.013178	5.61	0.4128
*P*_m_	0.001564	0.012396	11.21	0.0739
*P*_max_	0.001424	0.012537	10.20	0.1010
*P*_min_	0.000322	0.013639	2.31	0.9426

*r^2^* and permutation *P‐*values in bold and followed by an asterisk (*) remain significant at 0.05 level.

Molar shape diversification among Orkney Island populations (Fig. 5) is neither related to island size (*P* = 0.992) nor to the distances between them (PROTEST, Monte‐Carlo = 0.569, *P* = 0.122). However, the distance from Mainland Orkney (probably the first island colonized and thus the source for subsequent dispersal: Martínková et al. 2013) appears to be a factor that has contributed significantly to this molar shape diversification according to Pearson's test (Pearson's *R* = 0.825, *P* = 0.042) and close to significant according to the linear regression (*slope* = 0.0016; *intercept* = 0.0531; *r^2^* = 0.682; *permut P* < 0.0566). A significant correlation was found between pairwise genetic differentiation and shape differentiation measured by Procrustes distances among combined modern and ancient *M. arvalis orcadensis* populations (PROTEST, Monte‐Carlo = 0.404, *P* < 0.001), suggesting some concordance between molar shape and genetic divergence.

### MORPHOLOGICAL CHANGES IN MAINLAND ORKNEY VOLES AFTER INITIAL DIVERSIFICATION

**Figure 7:**
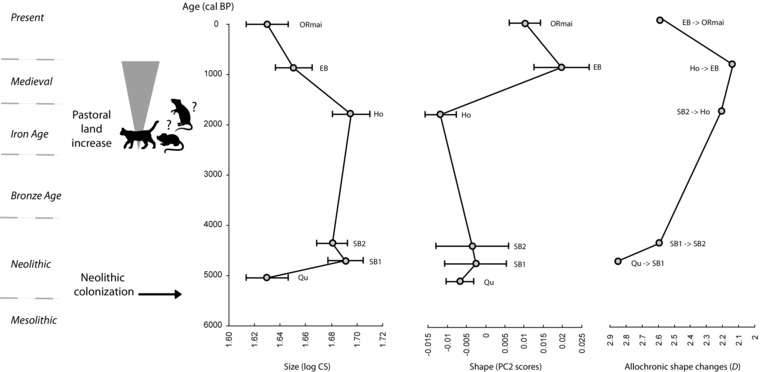
Morphological change over time in Mainland Orkney voles. (1) Molar size evolution depicted by mean size with confidence intervals. (2) Molar shape evolution depicted by mean scores along the second principal component axis detailed in Figure 6. (3) Allochronic shape changes expressed as Mahalanobis's distances (*D*) between each chronological step of the time series.

After the major divergence following their introduction, tooth morphology in Mainland Orkney voles did not follow the expected pattern of stasis (Fig. 7). During the earliest phase of the Neolithic, molar size experienced a further increase—seen between the sites of Quanterness (Qu) and Skara Brae (SB1), apparently followed by a stasis until the Late Iron Age—represented by specimens from Howe (Ho). Thereafter, specimens from Earl's Bu (EB) document a drastic size reduction between the 4th and the 12th century AD, which continued until the present time.

Far from displaying stasis after initial divergence, molar shape in Mainland Orkney voles also changed over time. During the early part of the record, limited divergence occurred more or less proportionally to the time elapsed. As was observed for size, a more substantial shift in molar shape occurred during the interval between Howe and Earl's Bu (i.e., between later prehistory and early Medieval times. Shape remained rather stable thereafter until modern times. This overall pattern is corroborated by considering morphological distance between successive samples, with the greatest morphological change occurring between Howe and Earl's Bu (Fig. 7).

## Discussion

### RAPID DIVERGENCE OF ORKNEY VOLES AFTER THEIR NEOLITHIC INTRODUCTION

The dental characteristics of Orkney voles—molar size gigantism and hypertrophied anterior loop—were acquired within less than a century (possibly within a few decades) after their Neolithic introduction and colonization of Orkney. No intermediate phenotype between continental European and Orkney *M. arvalis* was observed in the earliest (4th millennium BC) specimens from either Mainland Orkney or Westray, providing further evidence that newly colonizing island rodents exhibit extremely rapid initial divergence (Millien 2006; Nagorsen and Cardini 2009; Evans et al. 2012). Hence, phenotypic similarities that likely existed with a continental European source population were completely erased by this rapid morphological shift, rendering the identification of an ancestral phenotype among the continental populations all but impossible (Corbet 1986).

The molar size increase observed in *M. arvalis* on Orkney presumably reflects body size and may be a response to different levels of primary productivity (Rosenzweig 1968; Yom‐Tov and Geffen 2006, 2011; Medina et al. 2007; Blois et al. 2008) and/or a thermoregulatory response in accordance with Bergmann's rule. An alternative hypothesis would be that release of predation and competition pressure allowed the rodents to track their metabolic optimum by becoming larger (Damuth 1993.; Adler and Levins 1994; Michaux et al. 2002; Lomolino 2005; McNab 2010). This hypothesis is supported by a similar trend toward gigantism in Orkney and Guernsey voles, located on islands experiencing a different environmental regime but both characterized by the absence of the two major terrestrial predators of *M. arvalis* in continental Europe—i.e., stoats (*Mustela erminea*) and weasels (*Mustela nivalis*, Yalden 1999). Voles introduced to Orkney by Neolithic farmers faced only avian predation from hen harriers (*Circus cyaneus*) and short‐eared owls (*Asio flammeus*, Berry 1985)—and interspecific competition was reduced to only the wood mouse (*Apodemus sylvaticus*, Corbet 1979). In contrast, voles from Yeu and Noirmoutier—where weasels are present (Saint‐Girons and Nicolau‐Guillomet 1987)—do not display molar size gigantism as seen in the Guernsey and Orkney populations.

This molar gigantism apparently contributed to the hypertrophy seen in the anterior loop of the M_1_ through size allometry (Klingenberg 1996). In *Microtus* sp., the M_1_ anterior loop is the most variable and evolvable part of the tooth (Chaline et al. 1999; Jernvall et al. 2000; Renvoisé et al. 2009) due to its late development during morphogenesis (Jernvall et al. 2000). A similar mechanism has been evoked to explain parallel evolution in molar shape in insular house mice (*Mus musculus domesticus*, Renaud et al. 2011). The anterior elongation of the molar may correspond to a “line of least resistance to evolution” (Schluter 1996) related to developmental properties that are prone to being mobilized by size increase. Hence, drastic molar‐size increase driven by predation release and change in food resources (acting on overall body size) could have induced a broadening of the anterior loop of the molar.

The parallel evolution of a partly similar dental phenotype in Orkney and Guernsey voles—despite different phylogenetic signatures (Martínková et al. 2013)—supports the interpretation of a line of least evolutionary resistance, rather than a consequence of a common origin, as suggested by early 20th‐century naturalists (Miller 1909).

### FOUNDING EVENTS AND ORKNEY VOLE DIVERSIFICATION

The molar shape divergence revealed among Orkney voles—first separating voles from the southern and northern isles, and then islands within the two parts of the archipelago—could fit a scenario of diversification related to progressive subdivision of a single landmass as a consequence of sea‐level rise. This scenario, however, is not supported by the geology of the archipelago. Although Orkney was a single island during the Younger Dryas 13,000 years ago (Bates et al. 2013), relative sea‐level around Orkney by the time of Neolithic colonization between 5300 and 5100 years ago, is thought to have been approximately only 2 m lower than today (Bates et al. 2013).

Given the environmental uniformity among the Orkney Islands, the most likely processes driving this local evolution are chance effects related to successive founder events and subsequent genetic drift in the progressive colonization of the archipelago, together with the impermeability of the local population (once installed) to later invaders (Granjon and Cheylan 1988; Hardouin et al. 2010). According to data from mitochondrial and nuclear DNA, colonization occurred first on Mainland Orkney, followed by human‐mediated transportation of a few founders to the northern islands (Martínková et al. 2013). This colonization scenario is supported by the influence of geographic distance from Mainland Orkney in molar shape divergence of current Orkney vole populations, the small but significant overall influence of time elapsed since introduction on molar shape divergence and the congruence between the divergence patterns provided by molar shape and neutral molecular markers.

### ANTHROPOGENIC FORCES TRIGGERING POST‐ NEOLITHIC EVOLUTIONARY CHANGES

The archaeological time series on Mainland Orkney has also recorded phenotypic shifts in the morphology of Orkney voles millenia after their prehistoric introduction (Fig. 7). This finding contradicts the expectation that in an island setting, the voles should achieve an ecological optimum (after an initial divergence), and then change little after that. Instead, a major phenotypic shift is observed between the late Iron Age (4th to 7th centuries AD) and Medieval times (11th to 12th centuries AD). This phenotypic shift, long after their Neolithic introduction, suggests that evolution in the Orkney vole has been intimately linked with human influence on habitat and environment of the archipelago throughout its Holocene history (Hendry et al. 2008).

A later (post‐Medieval) molar size reduction in continental European *M. arvalis* also appears to be recorded in our data. This suggests that, for *M. arvalis* at least, body size reduction between fossil and modern specimens has not been a response to long‐term, natural global warming since the LGM (Millien et al. 2006), but rather a more recent anthropogenic phenomenon (Sheridan and Bickford 2011). A similar trend observed in mice species has been linked with pervasive anthropogenic perturbations—from habitat destruction to climate change (Cassaing et al. 2011; Stoetzel et al. 2013)—during very recent times. However, the archaeological record for Mainland Orkney shows size reduction in voles much earlier, between the 4th and 12th centuries AD, suggesting that anthropogenic impacts could have had greater/earlier effects on small mammals in confined/insular environments and/or in higher latitudinal locations.

The most dramatic shift in molar shape within Orkney voles is observed at the same time as size change, supporting the idea that major changes in the environment of the voles occurred at that period. Although human‐induced modification of the Orkney landscape was initiated as early as 5000 BP (Bunting 1994), the expansion of pastoral farming during the early and middle Iron Age (Bond 2002) led to an almost entirely open landscape with increasing numbers of livestock (Bunting 1994).

Around the same time, commensal mice (*Mus musculus domesticus*) and rats (*Rattus rattus*) were probably introduced to Orkney—they were present in mainland Britain during the Iron Age and Roman times, respectively (Yalden 1999). These commensal alien species might not have had a drastic effect on the Orkney vole populations since they did not compete for the same habitat. However, during the Iron Age, new terrestrial predators such as the domestic cat (*Felis sylvestris*) and fox (*Vulpes vulpes*)—both considered major predators of Orkney voles—were introduced to Orkney. Foxes are first recorded on Orkney in various Iron Age sites (Fairnell and Barrett 2007), whereas domestic cats are present as early as the 1st century AD at Ho (Ballin Smith 1994; O'Connor 2007.), Mine Howe (Mainland; unpublished data) and in later Iron Age deposits at Pool (Bond 2007) on Sanday. By the Viking and Norse periods, between 8th and 12th century AD, cats were clearly well established, occurring frequently in most archaeological sites of this period (Fairnell and Barrett 2007), while foxes disappear from the record.

The various changes associated with the increase in pasture land, and especially the introduction of foxes and cats, likely impacted Orkney vole populations (Whittaker 1998)—disturbing the local ecological equilibrium (Yom‐Tov et al. 1999; Sondaar 2000) of the species. Nevertheless, the impact of these changes on vole morphology seems to have become significant only when anthropogenic changes had become extensive.

## Conclusion

Orkney voles have evolved their own particular dental phenotype, likely the result of human agency influencing its evolutionary trajectory in different ways over the last 5000 years. This human influence began with its Neolithic introduction to the Orkney Mainland at a time when there were no terrestrial predators and only one competing species (the wood mouse). The Orkney vole population rapidly diverged from continental European *M. arvalis* to reach a new ecological optimum, that included evolutionary changes in morphology of the molar teeth. Neolithic farmers then dispersed the species to other islands of the archipelago—from Mainland to Westray and during the Bronze Age to Sanday—generating several founding events contributing to idiosyncratic differences in dental characteristics. This initial divergence and diversification in Orkney voles was not followed by morphological stasis because the Orkney environment was subjected to continued human disturbance.

The case of the Orkney vole presented here demonstrates how, from Neolithic times, humans have played a major role in species evolution and suggests that anthropogenic modifications of the environment may have repeatedly disturbed the phenotypic evolutionary stasis of insular species. Given the continental‐scale and increasing intensity of human‐induced impact on ecosystems in the last centuries, such changes in the evolutionary trajectories of vertebrates are likely not restricted to insular systems.

## Supporting information

Disclaimer: Supplementary materials have been peer‐reviewed but not copyedited.

**Table S1**.
