# Supplementary material for: THE CHANGING PACE OF INSULAR LIFE: 5000 YEARS OF MICROEVOLUTION IN THE ORKNEY VOLE (MICROTUS ARVALIS ORCADENSIS)
Source: Evolution. 2014 Jul 29;68(10):2804–20. doi: 10.1111/evo.12476 (PMC5366975; doi:10.1111/evo.12476)
Supplement: Supplementary file 1 — Table S1. [file EVO-68-2804-s001.zip › evo12476-sup-0001-table.pdf]

SI 1

Morphometric dataset of the 823 specimens studied (*M. agrestis* excluded) including the centroid size variable for each specimens (CS, logCS), the cartesian coordinates (x,y) of the 30 landmarks/ semilandmarks and the Procrustes coordinates (ProcCoo) after the GPA.

ID	Gppop	CS	logCS	x1	y1	x2	y2	x3	y3	x4	y4	x5	y5	x6	y6	x7	y7	x8	y8	x9	y9	x10	y10	x11	y11
Fr_Caen_10	M_Fr_Caen	4.02E+00	6.04E-01	1904	858	1750	1244	1624	829	1468	1207	1333	802	1155	1185	990	807	875	1156	668	874	877	1433	977	1193
Fr_Caen_11	M_Fr_Caen	3.99E+00	6.01E-01	2006	714	1873	1089	1711	696	1555	1056	1420	702	1197	1067	1091	696	970	1009	753	793	942	1284	1026	1071
Fr_Caen_12	M_Fr_Caen	4.36E+00	6.39E-01	2046	671	1917	1085	1731	649	1579	1054	1395	653	1202	1062	1050	673	860	1018	704	740	953	1302	1011	1020
Fr_Caen_13	M_Fr_Caen	4.26E+00	6.29E-01	2126	705	2019	1098	1826	684	1693	1054	1493	682	1333	1051	1155	680	999	1022	844	753	1104	1280	1106	1044
Fr_Caen_14	M_Fr_Caen	4.30E+00	6.33E-01	2099	678	1970	1078	1775	656	1662	1038	1448	651	1297	1045	1122	665	984	993	757	756	1059	1274	1104	1009
Fr_Caen_15	M_Fr_Caen	4.29E+00	6.32E-01	2117	769	1955	1142	1788	733	1637	1120	1460	729	1273	1149	1104	771	951	1156	797	878	1082	1407	1106	1169
Fr_Caen_16	M_Fr_Caen	4.03E+00	6.05E-01	1959	760	1833	1162	1648	740	1506	1113	1346	734	1173	1105	1046	738	822	1093	668	805	975	1325	993	1080
Fr_Caen_18	M_Fr_Caen	4.48E+00	6.51E-01	2182	658	2031	1034	1837	602	1679	1002	1477	613	1300	991	1128	614	982	954	750	713	1028	1267	1108	1004
Fr_Caen_19	M_Fr_Caen	4.03E+00	6.05E-01	2073	625	1951	1014	1788	642	1640	1011	1473	627	1306	1031	1151	665	1053	991	824	753	1051	1245	1130	1029
Fr_Caen_1	M_Fr_Caen	4.31E+00	6.34E-01	2095	682	1984	1102	1800	676	1673	1067	1473	673	1302	1071	1139	684	940	1047	811	762	1080	1296	1119	1056
Fr_Caen_21	M_Fr_Caen	4.20E+00	6.23E-01	1860	809	1826	1182	1571	785	1497	1178	1240	794	1100	1169	890	818	831	1127	571	905	799	1400	908	1162
Fr_Caen_22	M_Fr_Caen	4.08E+00	6.11E-01	2051	696	1866	1078	1720	667	1575	1060	1417	667	1219	1047	1100	676	964	1011	733	758	964	1309	1042	1045
Fr_Caen_24	M_Fr_Caen	4.14E+00	6.17E-01	2075	594	1913	969	1766	542	1566	942	1406	536	1177	933	1033	569	884	905	697	698	840	1165	1002	924
Fr_Caen_25	M_Fr_Caen	4.11E+00	6.14E-01	1990	711	1866	1094	1700	674	1544	1062	1371	676	1184	1073	1039	689	897	1033	700	787	900	1322	1015	1078
Fr_Caen_26	M_Fr_Caen	4.39E+00	6.42E-01	2126	756	1957	1156	1784	718	1650	1111	1440	720	1246	1122	1091	740	951	1065	766	813	984	1384	1039	1105
Fr_Caen_28	M_Fr_Caen	3.73E+00	5.72E-01	1911	736	1788	1091	1640	733	1515	1078	1364	722	1195	1080	1057	742	964	1051	744	809	960	1289	1028	1082
Fr_Caen_2	M_Fr_Caen	4.16E+00	6.19E-01	1986	698	1877	1107	1686	714	1570	1107	1384	705	1231	1104	1051	724	948	1082	710	804	977	1373	1048	1104
Fr_Caen_37	M_Fr_Caen	3.91E+00	5.92E-01	1999	794	1850	1140	1691	767	1542	1094	1357	745	1200	1100	1000	765	910	1076	664	880	973	1360	984	1104
Fr_Caen_39	M_Fr_Caen	4.23E+00	6.26E-01	2066	665	1935	1020	1766	629	1617	1018	1417	644	1239	1034	1080	658	997	984	750	756	960	1273	1062	1011
Fr_Caen_3	M_Fr_Caen	4.05E+00	6.07E-01	1899	696	1770	1089	1608	662	1455	1040	1280	656	1146	1040	970	667	864	1020	590	773	902	1318	970	1033
Fr_Caen_40	M_Fr_Caen	4.36E+00	6.39E-01	2055	682	1948	1109	1740	685	1600	1107	1417	694	1239	1118	1057	700	968	1071	713	765	968	1360	1048	1096
Fr_Caen_41	M_Fr_Caen	3.91E+00	5.92E-01	2037	691	1904	1076	1715	693	1582	1047	1428	698	1290	1040	1115	718	990	1024	795	767	1075	1267	1148	1025
Fr_Caen_42	M_Fr_Caen	4.14E+00	6.17E-01	2155	709	2050	1113	1855	693	1737	1064	1526	669	1384	1053	1150	684	1093	1024	839	756	1153	1289	1188	1029
Fr_Caen_43	M_Fr_Caen	4.19E+00	6.22E-01	2044	791	1888	1189	1695	756	1555	1147	1322	749	1179	1158	1013	776	851	1138	675	878	939	1402	968	1149
Fr_Caen_4	M_Fr_Caen	4.49E+00	6.52E-01	2124	754	1971	1149	1793	727	1651	1113	1451	727	1260	1111	1059	744	944	1087	691	847	975	1364	1044	1091
Fr_Caen_5	M_Fr_Caen	4.35E+00	6.38E-01	2142	722	1988	1084	1835	678	1639	1058	1475	684	1253	1047	1113	691	986	1011	771	791	997	1313	1062	1053
Fr_Caen_6	M_Fr_Caen	4.04E+00	6.06E-01	2040	716	1940	1102	1750	716	1613	1053	1437	691	1240	1060	1111	700	970	1029	755	796	999	1296	1055	1049
Fr_Caen_7	M_Fr_Caen	4.07E+00	6.10E-01	2146	836	1971	1185	1848	778	1613	1098	1504	731	1277	1071	1140	714	940	1065	753	800	1030	1309	1104	1044
Fr_Caen_8	M_Fr_Caen	4.16E+00	6.19E-01	2004	773	1875	1144	1700	718	1559	1084	1382	684	1184	1071	1017	696	920	1036	684	793	920	1291	1017	1065
Fr_Cal_183	M_Fr_Cal	4.12E+00	6.15E-01	1966	704	1844	1069	1653	673	1546	1054	1351	694	1179	1091	1028	702	890	1049	659	811	904	1307	970	1080
Fr_Cal_184	M_Fr_Cal	4.01E+00	6.03E-01	2084	705	1891	1022	1775	622	1564	987	1453	604	1244	985	1128	613	990	954	780	665	968	1194	1077	985
Fr_Cal_185	M_Fr_Cal	4.50E+00	6.53E-01	2190	664	2017	1038	1870	625	1671	1007	1502	642	1257	1033	1139	651	988	1014	760	747	993	1253	1091	1047
Fr_Cal_186	M_Fr_Cal	4.14E+00	6.17E-01	2013	631	1831	960	1706	571	1497	942	1368	564	1110	949	1011	578	880	907	651	682	880	1127	966	949
Fr_Cal_187	M_Fr_Cal	4.01E+00	6.03E-01	2004	660	1886	1056	1690	653	1575	1013	1382	644	1211	1016	1037	667	971	987	699	760	973	1273	1057	1024
Fr_Cal_188	M_Fr_Cal	4.24E+00	6.27E-01	1980	624	1839	982	1657	613	1517	956	1342	609	1164	982	986	618	890	956	640	711	879	1205	982	964
FR_CAL_201	M_Fr_Cal	3.76E+00	5.75E-01	1870	660	1737	1002	1584	624	1420	969	1264	607	1075	991	942	631	870	945	606	704	840	1178	937	976
Fr_Cal_202	M_Fr_Cal	4.01E+00	6.03E-01	2122	704	1966	1054	1828	651	1640	1013	1506	636	1270	1009	1160	649	1048	974	835	747	1008	1231	1113	1013
FR_CAL_203	M_Fr_Cal	4.26E+00	6.29E-01	1962	756	1828	1118	1600	702	1470	1085	1251	705	1091	1080	868	727	811	1044	553	844	811	1314	902	1074
FR_CAL_204	M_Fr_Cal	4.11E+00	6.14E-01	1990	669	1837	1051	1666	625	1493	1034	1317	636	1100	1049	962	667	804	989	646	774	810	1262	886	1016
FR_CAL_215	M_Fr_Cal	4.54E+00	6.57E-01	2030	689	1891	1080	1686	649	1570	1042	1337	660	1150	1084	966	704	842	1069	582	838	900	1356	971	1091
FR_CAL_216	M_Fr_Cal	4.14E+00	6.17E-01	1995	754	1873	1129	1697	727	1533	1085	1368	722	1170	1109	1024	738	926	1071	679	818	946	1351	1002	1100
FR_CAL_217	M_Fr_Cal	3.97E+00	5.99E-01	1970	764	1833	1102	1650	718	1491	1085	1328	724	1157	1096	1002	754	880	1080	670	856	931	1334	991	1089
FR_CAL_219	M_Fr_Cal	3.92E+00	5.93E-01	2088	709	1944	1038	1808	665	1642	1016	1495	638	1335	1005	1171	651	1071	973	830	725	1086	1222	1208	996
FR_CAL_225	M_Fr_Cal	4.45E+00	6.48E-01	2066	733	1937	1067	1730	654	1575	1029	1351	642	1188	1016	966	673	882	971	577	807	844	1276	964	1004
FR_CAL_230	M_Fr_Cal	3.88E+00	5.89E-01	1970	813	1800	1189	1682	780	1517	1153	1375	782	1193	1154	1030	800	900	1149	673	896	946	1400	1044	1154
FR_CAL_231	M_Fr_Cal	4.00E+00	6.02E-01	2060	767	1910	1134	1737	734	1571	1120	1440	720	1208	1118	1090	727	984	1074	722	807	975	1304	1055	1104
FR_CAL_233	M_Fr_Cal	3.96E+00	5.98E-01	2015	811	1868	1131	1695	771	1559	1104	1375	749	1219	1093	1062	745	975	1044	731	840	951	1320	1046	1084
FR_CAL_234	M_Fr_Cal	3.93E+00	5.94E-01	1926	749	1820	1107	1639	733	1530	1100	1346	734	1208	1102	1013	769	935	1104	706	834	913	1309	1040	1125
FR_CAL_235	M_Fr_Cal	4.52E+00	6.55E-01	2206	724	2042	1134	1871	689	1699	1087	1522	671	1277	1089	1119	700	1006	1044	742	791	971	1340	1086	1084
FR_CAL_236	M_Fr_Cal	4.47E+00	6.50E-01	2122	745	1919	1094	1755	689	1553	1078	1411	669	1179	1071	1020	718	866	1031	635	838	855	1298	968	1067
FR_CAL_237	M_Fr_Cal	3.93E+00	5.94E-01	1939	718	1782	1027	1646	642	1464	987	1337	620	1146	985	1011	642	920	982	722	733	910	1174	1015	1034
FR_CAL_238	M_Fr_Cal	4.04E+00	6.06E-01	1908	760	1770	1116	1586	720	1446	1102	1277	724	1086	1122	940	727	864	1076	568	842	790	1365	944	1125
FR_SM_255	M_Fr_SM	4.15E+00	6.18E-01	2010	711	1871	1078	1690	704	1540	1051	1373	694	1182	1042	1000	724	904	1031	671	825	913	1284	995	1062
FR_SM_256	M_Fr_SM	4.26E+00	6.29E-01	2033	765	1890	1064	1726	694	1570	1027	1395	676	1153	1022	1042	691	908	1004	699	778	884	1242	986	1049
FR_SM_260	M_Fr_SM	4.41E+00	6.44E-01	2259	696	2079	1060	1913	665	1737	1062	1559	656	1348	1071	1222	687	1075	1054	875	811	1055	1331	1160	1098
FR_SM_261	M_Fr_SM	4.34E+00	6.37E-01	2137	713	1931	1054	1771	673	1615	1038	1428	660	1217	1040	1042	685	922	1029	695	798	962	1265	1030	1073
FR_SM_262	M_Fr_SM	4.44E+00	6.47E-01	2095	694	1922	1022	1748	649	1588	1022	1391	653	1191	1024	1020	671	882	1007	679	769	930	1302	991	1029
FR_SM_270	M_Fr_SM	4.85E+00	6.86E-01	2213	571	2004	949	1860	544	1633	925	1420	540	1219	924	1024	558	862	913	615	673	960	1202	984	918
FR_SM_272	M_Fr_SM	4.42E+00	6.45E-01	2102	593	1959	962	1841	582	1619	951	1426	576	1202	931	1053	584	922	920	693	673	919	1185	1008	953
FR_SM_288	M_Fr_SM	4.30E+00	6.33E-01	2182	778	2024	1104	1871	738	1708	1073	1537	729	1348	1084	1195	745	1091	1056	840	827	1091	1347	1186	1098
FR_YEU_F10Y4	M_Fr_Yeu	5.14E+00	7.11E-01	2102	751	1999	1091	1788	744	1668	1082	1435	734	1275	1084	1090	756	962	1051	757	836	1053	1300	1070	1062
FR_YEU_F11Y5	M_Fr_Yeu	5.15E+00	7.12E-01	2039	727	1911	1073	1710	707	1571	1073	1380	694	1173	1082	1013	722	931	1053	660	820	937	1278	1019	1087
FR_YEU_12Y5	M_Fr_Yeu	5.24E+00	7.19E-01	2113	800	1962	1142	1804	833	1624	1134	1450	753	1242	1158	1086	778	995	1113	739	874	1008	1376	1075	1142
FR_YEU_F16Y2	M_Fr_Yeu	4.54E+00	6.57E-01	1866	722	1742	1025	1582	705	1453	1038	1293	700	1115	1033	977	707	848	976	620	807	868	1256	924	1005
FR_YEU_F19Y8	M_Fr_Yeu	5.17E+00	7.13E-01	2002	744	1880	1067	1682	702	1560	1042	1328	693	1144	1071	995	696	873	1020	650	787	919	1256	951	1054
FR_YEU_F1Y2	M_Fr_Yeu	4.52E+00	6.55E-01	1955	756	1846	1082	1680	745	1559	1078	1395	733	1220	1065	1090	731	999	1024	755	802	975	1280	1064	1054
FR_YEU_F22Y4	M_Fr_Yeu	4.98E+00	6.97E-01	2010	700	1839	1034	1682	645	1522	1014	1330	649	1142	1034	990	684	897	1009	668	774	911	1267	984	1029
FR_YEU_F7Y2	M_Fr_Yeu	4.33E+00	6.36E-01	1973	753	1860	1051	1691	731	1564	1044	1384	707	1251	1027	1059	725	973	993	788	809	1035	1202	1102	1009
Aud_667	M_Fr_Aude	4.06E+00	6.09E-01	498	113	481	202	434	108	403	194	359	108	317	197	280	113	247	189	195	127	259	262	268	196
Aud_668	M_Fr_Aude	4.28E+00	6.31E-01	446	181	433	267	377	173	351	259	299	168	263	254	219	166	187	252	130	182	186	321	223	256
Aud_669	M_Fr_Aude	4.04E+00	6.06E-01	427	160	407	246	359	148	323	242	273	153	234	244	182	163	165	238	106	191	180	308	191	248
Aud_678	M_Fr_Aude	4.11E+00	6.14E-01	450	187	425	270	381	173	338	266	297	173	248	266	208	177	177	256	131	198	197	328	209	262
Aud_681	M_Fr_Aude	3.93E+00	5.94E-01	468	166	441	249	398	154	361	239	324	150	277	235	249	154	211	227	169	172	220	296	238	233
Aud_682	M_Fr_Aude	3.90E+00	5.91E-01	450	169	422	255	383	162	350	248	313	159	270	244	236	154	203	236	158	168	213	302	226	238
Aud_683	M_Fr_Aude	4.07E+00	6.10E-01	457	168	424	247	382	157	349	242	297	157	267	243	217	162	194	237	137	186	202	309	216	249
Aud_685	M_Fr_Aude	4.37E+00	6.40E-01	480	180	453	275	406	170	369	268	330	166	278	264	256	168	216	240	170	180	216	322	235	248
Aud_689	M_Fr_Aude	4.27E+00	6.30E-01	482	169	463	252	411	161	383	247	336	161	293	249	258	164	194	251	171	178	230	308	230	252
Aud_690	M_Fr_Aude	3.77E+00	5.76E-01	460	176	436	257	396	170	357	256	323	165	276	255	245	166	212	245	163	188	213	311	233	249
Aud_691	M_Fr_Aude	4.23E+00	6.26E-01	463	167	439	251	390	161	352	250	304	162	257	255	212	167	189	253	122	197	188	301	210	256

Aud_692	M_Fr_Aude	4.46E+00	6.49E-01	513	166	457	238	416	138	380	233	322	135	280	230	233	144	200	226	144	169	200	292	230	233
Aud_698	M_Fr_Aude	4.05E+00	6.07E-01	443	146	415	239	372	139	338	233	294	138	251	237	208	139	184	228	122	166	182	288	207	238
Aud_699	M_Fr_Aude	4.33E+00	6.36E-01	522	154	497	241	447	149	410	244	367	145	312	239	283	147	223	230	192	161	257	294	261	234
Aud_700	M_Fr_Aude	4.45E+00	6.48E-01	529	139	498	223	452	128	408	223	371	125	316	220	277	125	234	212	184	147	248	284	263	217
Aud_708	M_Fr_Aude	3.93E+00	5.94E-01	425	186	402	273	360	181	331	267	289	179	249	269	211	184	175	269	131	211	180	319	205	268
Aud_710	M_Fr_Aude	4.53E+00	6.56E-01	495	161	464	250	418	146	383	248	332	145	290	237	241	144	208	230	153	168	214	312	238	242
Aud_718	M_Fr_Aude	4.38E+00	6.41E-01	483	132	462	224	408	124	383	220	327	122	282	214	244	126	184	222	152	150	219	279	236	218
Aud_719	M_Fr_Aude	4.58E+00	6.61E-01	500	133	479	240	421	128	393	230	335	126	287	233	247	131	209	218	146	158	216	302	230	225
Aud_722	M_Fr_Aude	4.22E+00	6.25E-01	514	129	491	213	444	123	411	212	357	118	309	211	265	126	234	202	177	147	247	275	264	204
Aud_724	M_Fr_Aude	4.87E+00	6.88E-01	565	192	535	289	481	181	448	286	383	183	343	293	291	200	252	285	192	229	280	364	285	295
Aud_745	M_Fr_Aude	4.16E+00	6.19E-01	490	136	466	222	421	127	394	221	343	125	296	225	258	133	241	212	181	150	244	283	255	223
Aud_760	M_Fr_Aude	4.24E+00	6.27E-01	525	164	482	247	448	150	408	240	361	150	318	239	275	153	251	230	192	175	258	294	272	234
Aud_761	M_Fr_Aude	4.31E+00	6.34E-01	477	175	450	269	400	172	362	261	322	175	271	260	223	180	195	253	141	196	204	325	220	259
Aud_762	M_Fr_Aude	4.02E+00	6.04E-01	481	177	450	254	416	162	375	246	333	158	288	244	256	167	223	239	174	183	218	297	244	243
Aud_763	M_Fr_Aude	3.79E+00	5.79E-01	452	158	425	240	385	152	348	233	311	143	261	236	236	149	196	217	152	166	205	280	219	223
Aud_766	M_Fr_Aude	4.41E+00	6.44E-01	491	184	458	268	411	167	377	260	327	165	288	255	245	173	206	247	156	196	216	328	237	258
Aud_768	M_Fr_Aude	4.05E+00	6.07E-01	452	182	425	270	383	169	345	267	299	169	255	270	218	180	191	265	130	210	202	328	216	278
Aud_770	M_Fr_Aude	3.73E+00	5.72E-01	466	153	437	230	400	142	369	224	330	144	285	226	251	150	233	220	175	169	222	268	250	231
Aud_771	M_Fr_Aude	3.91E+00	5.92E-01	441	166	422	247	378	159	348	240	302	156	266	237	223	157	184	231	144	178	205	296	215	233
Cant_003	M_Fr_Cant	4.44E+00	6.47E-01	714	275	681	396	618	271	566	395	513	269	451	400	409	279	355	403	302	308	371	490	403	406
Cant_004	M_Fr_Cant	4.50E+00	6.53E-01	683	293	630	402	576	280	518	399	472	275	405	397	354	282	319	387	232	309	313	468	346	395
Cant_005	M_Fr_Cant	4.68E+00	6.70E-01	750	333	719	458	644	333	606	457	532	338	469	470	407	352	367	459	298	390	387	547	400	466
Cant_006	M_Fr_Cant	4.62E+00	6.65E-01	768	278	722	411	660	275	599	409	556	271	473	401	437	280	365	398	306	315	384	490	409	397
Cant_007	M_Fr_Cant	4.30E+00	6.33E-01	719	319	671	432	620	304	560	420	508	301	443	423	390	312	349	427	289	343	366	498	391	425
Cant_008	M_Fr_Cant	4.76E+00	6.78E-01	721	301	675	427	618	285	556	415	493	285	419	419	374	290	301	411	250	322	364	492	352	411
Cant_009	M_Fr_Cant	4.70E+00	6.72E-01	677	373	642	486	587	355	535	480	478	351	405	482	360	353	303	470	224	389	323	562	335	475
Cant_010	M_Fr_Cant	4.39E+00	6.42E-01	690	307	638	426	588	291	533	416	480	287	410	418	357	294	316	410	245	332	323	502	353	414
Cant_011	M_Fr_Cant	4.00E+00	6.02E-01	696	306	668	414	606	303	572	421	510	305	467	422	408	317	395	422	313	356	400	503	425	433
Cant_012	M_Fr_Cant	4.16E+00	6.19E-01	692	312	657	425	600	300	555	421	499	307	443	430	391	317	363	421	289	348	367	504	395	430
Cant_013	M_Fr_Cant	4.36E+00	6.39E-01	658	250	622	359	569	233	515	356	463	229	401	350	359	237	317	336	252	269	308	430	342	343
Cant_014	M_Fr_Cant	4.43E+00	6.46E-01	712	290	695	401	626	277	592	402	518	284	472	413	408	292	389	403	302	329	401	493	418	416
Cant_015	M_Fr_Cant	4.05E+00	6.07E-01	669	297	641	408	582	289	540	406	481	287	428	406	377	292	351	389	280	317	362	482	378	402
Cant_016	M_Fr_Cant	4.51E+00	6.54E-01	687	343	633	462	592	327	526	446	481	320	404	439	367	322	320	424	250	350	315	521	343	442
Cant_017	M_Fr_Cant	4.42E+00	6.45E-01	708	308	672	425	606	301	565	423	498	306	444	430	382	312	352	421	273	353	351	515	379	430
Cant_018	M_Fr_Cant	4.53E+00	6.56E-01	756	266	708	391	651	255	595	382	538	249	475	379	425	258	375	361	310	281	388	453	409	372
Cant_019	M_Fr_Cant	4.29E+00	6.32E-01	695	285	649	397	603	271	539	386	494	267	424	383	380	272	328	382	266	309	344	466	374	382
Cant_020	M_Fr_Cant	4.77E+00	6.79E-01	724	333	675	450	621	330	557	446	503	317	428	447	382	325	329	430	263	357	348	520	364	442
Cant_021	M_Fr_Cant	4.43E+00	6.46E-01	734	161	680	265	640	140	566	253	523	134	443	261	412	141	361	247	295	171	375	329	394	263
Cant_022	M_Fr_Cant	4.57E+00	6.60E-01	734	258	700	373	638	249	580	374	514	248	451	375	400	250	339	364	284	287	351	449	380	367
Cant_023	M_Fr_Cant	4.31E+00	6.34E-01	738	344	693	455	641	333	591	454	540	334	478	462	427	342	395	455	323	378	404	539	422	459
Cant_024	M_Fr_Cant	4.53E+00	6.56E-01	602	239	549	342	500	220	432	334	379	217	309	342	258	228	191	347	146	276	224	426	245	334
Cant_025	M_Fr_Cant	4.27E+00	6.30E-01	560	291	521	408	471	284	415	413	369	289	305	414	257	301	211	408	154	333	224	490	247	419
Cant_026	M_Fr_Cant	4.33E+00	6.36E-01	705	387	673	502	612	389	570	498	499	383	452	496	379	399	365	498	274	439	362	583	395	504
Cant_027	M_Fr_Cant	4.59E+00	6.62E-01	722	297	683	418	621	285	566	411	515	292	446	412	394	293	333	406	272	325	373	488	372	403
Cant_028	M_Fr_Cant	4.27E+00	6.30E-01	587	324	542	435	490	314	435	431	379	317	320	438	263	328	234	433	148	364	250	505	266	439
Cant_029	M_Fr_Cant	4.08E+00	6.11E-01	548	313	514	413	465	297	415	417	371	298	310	414	272	296	238	401	164	316	225	485	265	410
CotDo_12095	M_Fr_Cdo	4.15E+00	6.18E-01	1112	633	1040	844	933	612	825	842	724	601	589	855	481	631	438	837	268	727	414	1018	495	863
CotDo_12096	M_Fr_Cdo	4.14E+00	6.17E-01	1151	645	1087	883	976	633	880	895	759	638	649	906	532	676	440	892	341	752	497	1083	527	888
CotDo_12097	M_Fr_Cdo	4.54E+00	6.57E-01	1208	610	1141	826	1011	589	924	846	781	619	697	860	554	658	497	853	318	725	534	1043	557	867
CotDo_12098	M_Fr_Cdo	4.29E+00	6.32E-01	1128	619	1066	876	928	612	853	878	719	629	610	897	495	663	419	879	268	738	447	1037	483	888
CotDo12099	M_Fr_Cdo	4.20E+00	6.23E-01	1110	631	1073	867	933	645	876	888	731	663	662	904	524	688	490	894	313	764	513	1071	550	911
CoDo_12100	M_Fr_Cdo	4.06E+00	6.09E-01	1148	580	1119	787	969	585	924	809	784	605	704	830	580	638	499	819	380	711	531	996	577	837
CoDo_12101	M_Fr_Cdo	4.29E+00	6.32E-01	1110	612	1063	855	926	612	850	876	740	631	619	890	513	652	437	878	309	729	463	1046	511	892
CoDo_12102	M_Fr_Cdo	4.45E+00	6.48E-01	1212	660	1116	874	992	640	907	871	765	652	641	890	536	684	490	890	314	769	451	1059	532	915
CotDo_12103	M_Fr_Cdo	3.99E+00	6.01E-01	1361	566	1265	748	1181	527	1077	738	979	521	869	732	779	528	699	699	568	562	703	858	747	727
CoDo_12115	M_Fr_Cdo	4.28E+00	6.31E-01	1073	704	1004	963	868	688	781	959	649	702	531	972	440	729	353	952	213	812	375	1156	423	961
CotDo_12116	M_Fr_Cdo	4.50E+00	6.53E-01	1187	637	1126	856	1001	624	928	851	779	633	676	863	540	658	477	840	305	716	508	1027	547	860
CoDo_12117	M_Fr_Cdo	4.46E+00	6.49E-01	1217	693	1144	938	1040	679	958	933	837	676	727	931	607	690	518	908	378	750	532	1105	605	926
CoDo_12118	M_Fr_Cdo	4.45E+00	6.48E-01	1229	603	1171	839	1047	589	962	839	827	598	717	863	609	624	541	826	376	700	534	1014	596	851
CoDo_12119	M_Fr_Cdo	4.66E+00	6.68E-01	1272	631	1178	856	1052	621	946	879	823	645	699	901	570	679	525	902	339	771	536	1083	580	917
CotDor_1210	M_Fr_Cdo	4.30E+00	6.33E-01	1176	681	1084	901	988	654	892	887	770	656	649	895	550	674	462	867	314	754	485	1037	529	887
CotDo_12121	M_Fr_Cdo	4.35E+00	6.38E-01	1146	390	1056	610	937	358	846	601	708	358	589	610	472	390	394	603	240	475	421	771	467	615
CoDo_12122	M_Fr_Cdo	4.21E+00	6.24E-01	1091	762	1018	975	907	757	816	981	692	762	616	993	467	798	424	986	252	879	435	1160	474	1011
CoDo_12123	M_Fr_Cdo	4.44E+00	6.47E-01	1293	668	1217	915	1100	647	999	904	876	649	752	915	630	683	543	885	407	750	543	1048	630	902
CoDo_12124	M_Fr_Cdo	4.49E+00	6.52E-01	1228	629	1153	844	1024	619	944	849	814	621	697	853	568	624	509	823	318	690	488	1009	561	853
CoDo_12125	M_Fr_Cdo	4.51E+00	6.54E-01	1183	360	1123	601	988	355	926	624	770	381	685	637	547	402	485	622	330	473	531	816	541	637
CoDo_12126	M_Fr_Cdo	4.23E+00	6.26E-01	1190	443	1128	679	1011	422	919	654	816	413	687	649	584	429	516	615	366	486	495	810	589	649
CoDo_12129	M_Fr_Cdo	4.11E+00	6.14E-01	1187	397	1148	642	995	392	951	644	784	408	726	672	564	465	559	663	368	564	587	844	609	679
CoDo_12130	M_Fr_Cdo	4.50E+00	6.53E-01	1135	626	1091	855	946	612	871	860	713	642	618	887	477	667	417	865	268	748	458	1060	472	878
CoDo_12131	M_Fr_Cdo	4.50E+00	6.53E-01	1228	406	1142	631	1011	392	912	628	798	386	673	628	555	395	458	599	316	466	516	785	540	613
HauSav_30	M_Fr_Hsa	4.24E+00	6.27E-01	558	236	524	359	466	228	416	352	362	229	305	353	255	231	216	332	163	245	233	420	241	337
HauSav_31	M_Fr_Hsa	4.49E+00	6.52E-01	628	301	598	419	532	303	491	426	427	316	373	438	322	333	279	443	211	373	302	529	309	448
HauSav_32	M_Fr_Hsa	4.32E+00	6.35E-01	592	356	549	466	493	337	448	469	384	346	324	480	266	363	231	482	161	393	256	558	278	479
HauSav_33	M_Fr_Hsa	4.19E+00	6.22E-01	559	212	533	337	468	204	432	332	369	206	330	331	261	217	247	325	161	238	256	414	272	330
HauSav_34	M_Fr_Hsa	4.52E+00	6.55E-01	607	345	577	461	508	340	464	449	405	338	356	458	299	349	265	446	193	381	266	539	293	452
HauSav_35	M_Fr_Hsa	4.66E+00	6.68E-01	636	177	598	297	534	162	481	294	429	161	371	297	317	166	272	284	208	191	283	375	305	286
HauSav_36	M_Fr_Hsa	4.26E+00	6.29E-01	608	196	558	301	508	195	464	294	410	191	355	295	306	200	272	286	205	218	266	368	296	294
HauSav_37	M_Fr_Hsa	4.26E+00	6.29E-01	560	320	516	424	454	315	415	430	356	324	306	437	249	341	223	432	134	381	222	510	253	440
HauSav_38	M_Fr_Hsa	4.49E+00	6.52E-01	586	327	560	442	498	325	456	443	398	333	338	448	286	338	256	425	183	364	252	518	276	439
HauSav_39	M_Fr_Hsa	4.45E+00	6.48E-01	591	192	540	314	472	195	421	322	361	197	305	320	243	218	212	314	132	254	217	405	244	317
HauSav_40	M_Fr_Hsa	4.21E+00	6.24E-01	531	331	514	451	442	338	408	452	346	344	299	460	231	360	201	470	125	399	223	544	234	468
HauSav_41	M_Fr_Hsa	4.30E+00	6.33E-01	604	193	564	307	510	178	452	302	398	177	335	306	282	193	259	290	179	222	250	378	284	302
HauSav_42	M_Fr_Hsa	4.36E+00	6.39E-01	597	231	548	345	499	223	445	345	400	222	334	338	297	228	259	325	186	249	250	403	282	333

HauSav_43	M_Fr_Hsa	4.28E+00	6.31E-01	573	313	539	413	504	309	438	417	398	310	331	429	279	317	248	420	179	346	255	517	276	428
HauSav_44	M_Fr_Hsa	4.20E+00	6.23E-01	614	330	566	438	518	321	471	437	419	323	366	445	327	333	285	433	217	363	282	516	314	441
HauSav_45	M_Fr_Hsa	4.01E+00	6.03E-01	555	303	515	426	457	294	415	420	362	307	318	428	262	325	232	429	159	355	252	506	267	434
HauSav_49	M_Fr_Hsa	4.68E+00	6.70E-01	602	341	565	454	506	335	468	454	395	341	351	458	291	349	260	447	173	381	250	547	295	462
HauSav_50	M_Fr_Hsa	4.28E+00	6.31E-01	590	178	552	295	500	173	454	286	395	174	343	290	298	175	256	279	185	204	263	370	288	286
HauSav_51	M_Fr_Hsa	4.98E+00	6.97E-01	666	223	637	339	574	210	520	336	457	205	389	341	334	214	279	325	196	261	288	423	314	333
Jouy_1507	M_Fr_IDF	4.44E+00	6.47E-01	539	97	509	186	458	89	428	189	373	92	327	192	280	104	249	192	194	139	256	263	270	205
Jouy_1510	M_Fr_IDF	4.29E+00	6.32E-01	488	142	461	230	412	139	383	224	330	139	275	231	241	144	202	226	154	167	213	294	224	232
Jouy_1511	M_Fr_IDF	4.32E+00	6.35E-01	538	114	503	210	458	112	418	205	373	113	325	205	284	121	263	196	200	144	262	269	280	211
Jouy_1512	M_Fr_IDF	4.18E+00	6.21E-01	500	92	467	178	426	86	386	179	349	85	291	178	256	86	233	163	175	111	228	239	251	178
Jouy_1513	M_Fr_IDF	4.16E+00	6.19E-01	530	108	483	194	454	96	402	184	370	92	311	180	277	95	254	168	196	121	238	248	272	180
Jouy_1540	M_Fr_IDF	4.14E+00	6.17E-01	483	152	436	229	402	137	358	221	322	134	263	222	232	137	197	207	146	167	196	278	219	222
Jouy_1815	M_Fr_IDF	4.51E+00	6.54E-01	519	157	475	250	437	146	388	240	351	143	292	237	261	147	230	222	166	166	223	301	248	240
Sac_1455	M_Fr_IDF	4.37E+00	6.40E-01	500	165	469	249	419	156	384	251	336	160	287	253	245	172	222	248	163	200	222	317	245	256
Sac_1457	M_Fr_IDF	3.82E+00	5.82E-01	508	116	470	200	439	105	391	192	354	100	307	192	269	98	248	178	190	128	241	234	270	187
Sac_1463	M_Fr_IDF	4.29E+00	6.32E-01	476	150	444	241	397	140	366	234	312	139	285	229	220	144	205	226	141	169	205	289	233	238
Sac_1764	M_Fr_IDF	4.42E+00	6.45E-01	490	142	469	233	410	141	388	233	333	147	294	238	242	153	225	229	155	180	228	301	241	239
Vers_105	M_Fr_IDF	4.20E+00	6.23E-01	530	92	497	186	456	85	410	180	369	89	314	179	273	97	260	172	183	130	250	235	274	180
Vers_362	M_Fr_IDF	4.04E+00	6.06E-01	527	193	500	276	457	190	420	278	383	190	344	283	298	199	281	280	215	222	277	340	297	293
Vers_363	M_Fr_IDF	3.87E+00	5.88E-01	504	128	473	215	434	121	389	207	361	119	308	203	277	119	244	198	196	136	248	265	265	205
Vers_393	M_Fr_IDF	4.34E+00	6.37E-01	522	129	493	223	447	123	405	213	361	121	308	215	272	124	230	207	186	145	243	273	262	218
Vers_399	M_Fr_IDF	3.95E+00	5.97E-01	545	104	504	185	472	88	420	180	393	83	346	168	305	81	280	163	225	103	274	216	310	166
Vil_1814	M_Fr_IDF	4.46E+00	6.49E-01	508	171	471	250	431	150	386	243	361	152	292	243	260	154	226	235	163	173	216	303	256	244
LoAtl_61	M_Fr_Lat	4.42E+00	6.45E-01	583	317	556	437	486	317	446	440	376	320	332	449	272	339	224	451	156	369	259	543	268	448
LoAtl_62	M_Fr_Lat	4.28E+00	6.31E-01	604	301	567	417	498	295	463	411	397	300	355	420	280	316	268	421	174	347	267	510	297	427
LoAtl_63	M_Fr_Lat	4.16E+00	6.19E-01	618	381	588	502	525	372	481	498	420	381	368	508	308	395	282	506	204	424	284	587	311	510
LoAtl_64	M_Fr_Lat	4.39E+00	6.42E-01	618	263	581	365	517	250	484	369	403	252	346	377	295	264	246	362	178	298	264	456	282	372
LoAtl_65	M_Fr_Lat	4.54E+00	6.57E-01	610	290	577	407	519	281	473	412	402	275	349	415	296	291	244	410	183	313	272	498	290	402
LoAtl_66	M_Fr_Lat	4.46E+00	6.49E-01	597	313	549	430	499	303	448	421	385	303	340	424	260	315	223	427	156	348	246	519	265	430
LoAtl_67	M_Fr_Lat	4.36E+00	6.39E-01	656	227	621	349	556	217	516	341	452	218	389	347	342	223	303	343	233	253	323	428	332	351
LoAtl_68	M_Fr_Lat	4.44E+00	6.47E-01	594	341	561	452	491	322	457	450	377	333	350	459	266	357	232	469	169	391	260	559	276	465
LoAtl_69	M_Fr_Lat	4.31E+00	6.34E-01	598	358	563	472	495	349	455	466	393	341	339	462	298	350	246	462	179	378	252	545	282	459
LoAtl_70	M_Fr_Lat	4.43E+00	6.46E-01	653	358	616	469	560	352	516	462	449	346	395	461	335	351	280	462	225	383	320	559	323	468
LoAtl_71	M_Fr_Lat	4.96E+00	6.95E-01	687	212	634	338	582	200	523	333	464	196	406	334	352	208	270	333	221	236	316	424	333	334
LoAtl_72	M_Fr_Lat	4.12E+00	6.15E-01	588	187	552	306	503	180	458	304	403	180	354	305	298	188	259	301	184	224	283	395	292	304
Morb_3956	M_Fr_Mor	4.32E+00	6.35E-01	552	235	515	326	477	227	438	324	394	224	347	319	297	227	278	307	216	246	278	379	300	319
Morb_3957	M_Fr_Mor	4.42E+00	6.45E-01	562	214	522	299	485	201	444	296	393	199	352	301	302	207	272	294	219	232	274	371	298	303
Morb_3958	M_Fr_Mor	4.34E+00	6.37E-01	556	221	520	315	484	211	433	307	396	206	341	301	301	213	272	295	211	235	272	368	294	302
Morb_3959	M_Fr_Mor	4.26E+00	6.29E-01	523	226	497	309	447	211	421	309	361	210	334	302	276	215	251	297	198	243	261	376	274	306
Morb_3960	M_Fr_Mor	4.15E+00	6.18E-01	527	235	483	307	448	215	406	302	370	207	326	296	286	207	257	282	195	229	249	360	277	293
Morb_3961	M_Fr_Mor	4.15E+00	6.18E-01	531	232	499	305	457	218	422	305	369	216	337	307	284	222	262	302	205	235	266	371	283	307
Morb_3962	M_Fr_Mor	4.35E+00	6.38E-01	552	195	519	275	470	179	438	272	376	178	336	274	284	187	266	264	205	211	266	345	285	278
Morb_3963	M_Fr_Mor	4.39E+00	6.42E-01	546	209	516	297	468	201	434	290	384	202	343	293	294	207	269	282	204	237	269	355	288	290
Morb_3964	M_Fr_Mor	4.25E+00	6.28E-01	527	192	495	274	447	179	420	273	365	179	342	276	280	186	264	273	200	211	266	352	288	283
Morb_3965	M_Fr_Mor	4.29E+00	6.32E-01	548	215	523	306	482	213	443	305	405	210	357	307	319	212	284	298	223	235	286	363	312	309
Morb_3966	M_Fr_Mor	4.69E+00	6.71E-01	565	238	527	328	485	222	441	317	391	217	337	318	293	224	246	298	202	245	259	374	280	305
Morb_3967	M_Fr_Mor	4.07E+00	6.10E-01	532	221	502	308	454	218	423	310	374	215	339	302	291	215	252	299	211	235	274	370	289	299
Morb_3968	M_Fr_Mor	4.07E+00	6.10E-01	553	221	522	310	483	217	441	297	400	212	351	301	314	210	289	283	230	231	280	357	310	295
Noir_3915	M_Fr_Noi	4.54E+00	6.57E-01	459	211	426	298	384	200	348	289	306	197	257	292	222	200	194	278	142	221	198	352	214	290
Noir_3916	M_Fr_Noi	4.41E+00	6.44E-01	463	180	433	261	386	175	355	257	308	173	263	258	227	175	198	250	150	193	207	313	221	260
Noir_3918	M_Fr_Noi	4.59E+00	6.62E-01	468	180	438	256	392	168	357	254	311	169	272	250	225	171	182	248	138	195	205	316	219	250
Noir_3920	M_Fr_Noi	4.51E+00	6.54E-01	472	194	444	280	404	191	362	275	325	187	273	272	246	184	201	251	166	195	208	319	214	262
Noir_3921	M_Fr_Noi	4.66E+00	6.68E-01	512	189	474	262	432	173	391	263	345	173	300	259	258	176	205	249	169	203	239	314	244	250
Noir_3922	M_Fr_Noi	4.49E+00	6.52E-01	474	187	434	258	398	171	358	247	318	168	267	247	238	169	197	236	151	188	205	308	221	252
Noir_3924	M_Fr_Noi	4.63E+00	6.66E-01	472	170	442	252	404	160	361	248	322	156	277	245	242	159	200	233	158	182	216	308	228	243
Noir_3925	M_Fr_Noi	4.48E+00	6.51E-01	483	183	449	263	406	172	369	253	330	167	281	259	252	169	226	247	170	187	224	315	246	257
Noir_3927	M_Fr_Noi	4.39E+00	6.42E-01	472	186	438	265	402	175	356	253	321	169	277	251	244	168	205	235	170	188	216	308	229	248
Noir_3928	M_Fr_Noi	4.62E+00	6.65E-01	506	161	472	247	430	152	398	237	350	149	311	233	273	147	224	215	182	164	247	295	261	228
Noir_3929	M_Fr_Noi	4.61E+00	6.64E-01	515	175	481	255	438	168	403	250	360	168	306	251	278	168	222	239	193	188	258	307	261	253
Noir_3930	M_Fr_Noi	4.60E+00	6.63E-01	461	191	436	271	393	183	359	266	316	183	264	269	235	184	196	253	152	200	204	322	222	267
Noir_3931	M_Fr_Noi	4.28E+00	6.31E-01	445	150	416	228	377	143	342	230	304	146	260	228	222	149	193	225	148	170	203	294	222	226
Noir_3932	M_Fr_Noi	4.56E+00	6.59E-01	495	154	467	233	425	144	392	219	344	137	302	219	268	136	218	205	184	151	238	278	250	213
Noir_3934	M_Fr_Noi	4.43E+00	6.46E-01	460	178	418	255	384	165	338	248	307	163	250	245	218	165	186	233	143	182	194	301	209	242
Noir_3935	M_Fr_Noi	4.42E+00	6.45E-01	486	189	451	260	419	179	380	250	347	169	292	249	261	166	216	236	184	186	238	303	245	246
Noir_3936	M_Fr_Noi	4.46E+00	6.49E-01	475	162	448	244	403	158	372	242	325	160	282	241	244	160	212	227	157	176	219	298	233	237
Noir_3937	M_Fr_Noi	4.33E+00	6.36E-01	497	177	459	255	425	172	392	247	350	167	310	244	275	167	235	236	196	187	247	302	266	243
Noir_3938	M_Fr_Noi	4.66E+00	6.68E-01	511	175	473	253	431	163	395	250	351	159	300	253	267	165	218	237	181	185	239	305	249	247
Noir_3939	M_Fr_Noi	4.74E+00	6.76E-01	498	194	461	270	417	183	380	268	334	181	288	267	252	185	206	255	162	208	228	328	233	266
Noir_3940	M_Fr_Noi	4.37E+00	6.40E-01	472	188	432	258	394	170	361	251	318	171	275	252	233	170	212	242	155	186	215	312	235	252
Noir_3941	M_Fr_Noi	4.69E+00	6.71E-01	502	176	461	255	426	154	377	251	342	150	286	241	261	147	203	227	172	177	226	300	233	236
Noir_3942	M_Fr_Noi	4.68E+00	6.70E-01	483	176	455	259	408	170	374	257	323	169	278	257	244	174	195	249	155	199	219	317	228	257
Noir_3943	M_Fr_Noi	4.52E+00	6.55E-01	462	170	428	255	390	159	350	247	310	154	264	244	221	157	186	240	135	186	194	300	215	250
Noir_3944	M_Fr_Noi	4.46E+00	6.49E-01	453	203	427	279	383	192	350	275	308	187	266	269	225	185	192	255	141	208	198	325	214	264
Noir_3945	M_Fr_Noi	4.43E+00	6.46E-01	471	155	447	236	400	152	368	233	319	155	273	236	247	160	201	225	163	179	214	288	231	236
Noir_4332	M_Fr_Noi	4.55E+00	6.58E-01	482	158	454	236	408	149	371	236	322	153	275	239	240	158	200	228	153	179	219	286	227	236
Noir_4333	M_Fr_Noi	4.43E+00	6.46E-01	473	189	438	264	400	178	362	261	325	176	272	261	243	178	207	246	155	201	208	312	225	257
Noir_4334	M_Fr_Noi	4.48E+00	6.51E-01	474	192	435	264	400	175	361	255	319	167	270	249	242	168	200	241	155	184	206	309	227	249
Noir_4335	M_Fr_Noi	4.52E+00	6.55E-01	494	183	461	261	421	172	380	253	343	168	288	250	259	164	222	237	172	183	230	300	241	246
Vend_1265	M_Fr_Ven	4.25E+00	6.28E-01	532	207	511	299	459	197	422	288	368	189	319	290	283	195	252	276	200	218	243	351	275	281
Vend_1268	M_Fr_Ven	4.25E+00	6.28E-01	487	152	461	232	416	136	378	225	330	137	282	228	244	142	206	225	160	164	216	294	238	228
Vend_1272	M_Fr_Ven	4.51E+00	6.54E-01	562	224	525	310	487	210	438	304	391	207	347	306	300	215	269	301	211	240	283	374	293	299
Vend_1274	M_Fr_Ven	4.19E+00	6.22E-01	529	218	483	307	451	206	400	299	365	201	308	295	269	205	243	292	182	233	246	346	271	296
Vend_1276	M_Fr_Ven	4.28E+00	6.31E-01	541	170	518	257	469	165	433	257	383	162	338	258	294	169	263	251	202	197	288	320	288	255

Vend_1277	M_Fr_Ven	4.31E+00	6.34E-01	533	188	516	274	462	185	432	272	380	186	332	276	300	190	258	260	208	209	275	340	286	272
Vend_1279	M_Fr_Ven	4.33E+00	6.36E-01	519	123	500	219	447	114	415	211	365	111	313	219	277	119	241	208	190	142	259	279	269	217
Vend_1311	M_Fr_Ven	4.18E+00	6.21E-01	524	105	500	192	452	95	419	186	365	99	321	194	277	105	252	183	199	132	261	252	274	189
Vend_1312	M_Fr_Ven	4.50E+00	6.53E-01	560	187	529	280	477	177	447	271	385	179	342	276	295	185	264	267	209	213	287	343	288	274
Vend_1318	M_Fr_Ven	4.29E+00	6.32E-01	558	182	526	269	487	171	441	267	398	171	344	265	311	171	279	246	219	200	280	316	302	260
Vend_1766	M_Fr_Ven	4.39E+00	6.42E-01	530	119	501	204	449	109	415	203	359	113	315	203	265	126	231	200	180	155	240	266	266	204
Vend_1767	M_Fr_Ven	4.45E+00	6.48E-01	465	166	448	260	398	158	367	254	316	155	271	251	225	155	196	241	135	175	189	311	225	246
Vend_1768	M_Fr_Ven	4.47E+00	6.50E-01	577	194	547	279	500	181	460	271	411	177	361	274	314	177	286	265	219	206	297	329	311	267
Vend_1769	M_Fr_Ven	4.43E+00	6.46E-01	524	225	500	308	450	218	412	305	362	214	306	309	268	215	241	292	177	241	238	360	261	303
Vend_1770	M_Fr_Ven	4.33E+00	6.36E-01	547	214	523	305	475	209	432	301	391	207	333	301	308	208	270	289	219	242	272	360	284	297
Vend_1771	M_Fr_Ven	4.54E+00	6.57E-01	491	164	463	243	412	150	372	243	327	145	272	242	241	145	208	222	145	169	198	301	230	235
Vend_1772	M_Fr_Ven	4.66E+00	6.68E-01	533	113	502	214	454	102	405	208	363	103	295	217	261	118	229	205	166	148	225	284	245	215
Vend_1773	M_Fr_Ven	4.45E+00	6.48E-01	564	243	530	330	476	228	440	324	380	221	330	318	288	223	250	308	200	252	270	379	281	311
Vend_1774	M_Fr_Ven	4.21E+00	6.24E-01	539	199	519	273	469	191	438	276	383	190	344	280	305	197	271	265	226	215	279	336	295	274
Vien_1624	M_Fr_Vie	4.18E+00	6.21E-01	533	219	499	305	458	210	421	290	380	201	331	291	291	200	255	279	207	214	266	348	280	283
Vien_1629	M_Fr_Vie	4.25E+00	6.28E-01	552	220	521	309	480	212	437	301	392	208	344	299	307	210	269	285	223	224	280	351	294	291
Vien_1630	M_Fr_Vie	4.33E+00	6.36E-01	527	231	498	316	457	218	418	307	368	211	314	303	286	213	244	288	195	228	260	354	268	297
Vien_1634	M_Fr_Vie	4.43E+00	6.46E-01	560	232	527	314	483	220	438	305	401	216	346	307	315	217	250	302	230	224	283	358	292	297
Vien_1636	M_Fr_Vie	4.15E+00	6.18E-01	546	195	522	282	475	193	444	282	392	191	347	287	313	202	282	284	238	225	286	346	303	289
Vien_1637	M_Fr_Vie	4.33E+00	6.36E-01	552	221	529	307	479	210	444	309	404	203	348	303	315	215	272	293	230	236	283	360	297	300
Vien_1638	M_Fr_Vie	4.06E+00	6.09E-01	544	232	511	314	472	216	433	304	392	210	344	305	317	210	272	285	228	225	293	352	294	293
Vien_1643	M_Fr_Vie	4.32E+00	6.35E-01	534	230	505	324	461	219	421	317	375	218	325	318	291	221	254	310	198	245	253	379	280	313
Vien_1644	M_Fr_Vie	4.39E+00	6.42E-01	551	226	519	305	477	213	435	310	397	213	338	309	309	216	269	302	220	235	275	363	295	309
Vien_1652	M_Fr_Vie	4.17E+00	6.20E-01	542	224	509	307	467	215	422	302	386	213	327	306	306	215	257	296	225	232	269	354	297	310
Vien_1653	M_Fr_Vie	4.13E+00	6.16E-01	544	246	504	316	469	229	425	314	388	221	339	310	311	220	266	298	233	230	284	360	295	303
Vien_1661	M_Fr_Vie	4.26E+00	6.29E-01	541	207	519	312	470	206	433	304	388	204	345	304	297	213	265	298	214	230	278	362	295	301
Vien_1664	M_Fr_Vie	4.20E+00	6.23E-01	547	241	510	315	479	230	432	313	403	221	339	312	329	217	282	298	244	227	280	356	297	307
GerSax_12132	M_Gr_Sax	4.25E+00	6.28E-01	1189	608	1116	848	992	606	892	862	788	631	680	888	563	661	493	865	341	723	524	1050	547	879
GerSax_12133	M_Gr_Sax	4.55E+00	6.58E-01	1204	713	1142	933	1002	725	908	949	790	739	665	968	536	752	469	959	309	824	504	1154	529	984
GerSax_12134	M_Gr_Sax	4.20E+00	6.24E-01	1150	665	1077	853	949	638	852	867	726	651	600	879	504	668	437	863	266	746	431	1034	481	881
GerSax_12135	M_Gr_Sax	3.98E+00	6.00E-01	1228	427	1110	628	1022	408	924	608	818	404	692	605	595	411	518	594	376	475	524	746	575	615
GerSax_12136	M_Gr_Sax	4.21E+00	6.24E-01	1185	642	1107	858	1002	617	898	837	777	613	673	846	550	624	508	826	321	688	509	1007	559	856
GerSax_12137	M_Gr_Sax	4.24E+00	6.27E-01	1181	589	1114	785	990	576	923	778	767	583	676	798	561	610	492	803	348	684	540	956	552	819
GerSax_12138	M_Gr_Sax	4.06E+00	6.08E-01	1181	411	1096	606	974	402	884	594	756	397	648	603	522	408	456	587	305	473	499	768	515	612
GerSax_12139	M_Gr_Sax	4.08E+00	6.11E-01	1222	408	1151	637	1036	390	947	619	829	386	713	624	600	410	515	608	391	488	541	780	598	621
GerSax_12140	M_Gr_Sax	4.75E+00	6.76E-01	1577	541	1508	830	1361	555	1272	821	1125	569	1020	844	873	606	798	853	632	700	864	1028	868	858
GerSax_12141	M_Gr_Sax	4.32E+00	6.35E-01	1150	624	1070	844	963	598	862	839	742	601	632	853	515	624	451	821	282	706	451	1028	499	849
GerSAx_12142	M_Gr_Sax	4.15E+00	6.18E-01	1167	676	1105	894	956	677	889	904	743	697	651	931	504	736	481	924	295	816	518	1074	536	940
GerSax_12143	M_Gr_Sax	4.03E+00	6.05E-01	1197	706	1139	908	1029	695	947	917	836	704	731	938	600	734	557	952	398	826	587	1122	614	965
GerSax_12144	M_Gr_Sax	4.18E+00	6.21E-01	1103	699	1061	918	921	700	864	922	713	707	621	938	479	723	421	915	261	784	483	1112	502	938
GerSax_12145	M_Gr_Sax	4.16E+00	6.19E-01	1158	440	1068	651	983	426	880	642	784	411	667	631	571	413	483	612	320	463	525	785	525	628
GerSax_12146	M_Gr_Sax	4.36E+00	6.40E-01	1249	720	1176	945	1052	700	947	943	832	695	708	952	589	725	509	945	369	800	520	1121	610	961
GerSax_12147	M_Gr_Sax	4.11E+00	6.14E-01	1174	647	1107	856	981	644	892	860	768	649	665	876	524	686	486	876	314	754	540	1028	552	888
GerSax_12148	M_Gr_Sax	4.62E+00	6.65E-01	1256	434	1173	645	1077	402	951	629	813	381	671	645	555	413	474	631	314	484	497	805	548	649
GerSax_12149	M_Gr_Sax	4.20E+00	6.23E-01	1178	697	1123	940	1001	679	923	934	790	681	687	940	552	711	502	879	364	762	476	1094	548	908
GerSax_12150	M_Gr_Sax	4.30E+00	6.33E-01	1212	628	1126	855	1004	608	921	842	777	621	692	874	561	651	486	860	341	729	513	1035	566	863
GerSax_12152	M_Gr_Sax	4.24E+00	6.27E-01	1126	598	1040	816	935	571	832	803	717	574	612	807	481	592	407	787	268	656	435	977	481	810
GerSax_12153	M_Gr_Sax	4.56E+00	6.59E-01	1263	417	1158	667	1063	385	924	642	830	390	676	637	593	415	474	603	366	475	492	803	550	622
GerSAx_12154	M_Gr_Sax	4.51E+00	6.55E-01	1215	629	1174	869	1032	642	953	871	811	651	687	902	554	684	506	883	323	769	511	1078	555	906
GerSax_12155	M_Gr_Sax	4.26E+00	6.30E-01	1176	355	1091	569	983	340	876	562	784	330	658	555	575	355	493	528	330	413	485	715	548	558
MarDSC_1	M_Gr_Wol	4.62E+00	6.65E-01	1178	475	1096	670	999	450	902	652	810	450	706	662	623	471	562	644	458	518	569	786	602	664
MarDSC_3	M_Gr_Wol	4.39E+00	6.42E-01	1102	487	1052	668	960	468	894	654	793	462	694	657	620	484	562	641	443	528	567	793	597	662
MarDSC_4	M_Gr_Wol	4.74E+00	6.76E-01	1165	452	1102	641	1010	427	915	626	829	428	723	633	643	439	566	610	468	493	570	770	618	633
MarDSC_5	M_Gr_Wol	4.09E+00	6.12E-01	1139	451	1068	632	995	432	908	612	838	429	745	621	670	444	617	612	507	492	631	737	656	629
MarDSC_6	M_Gr_Wol	4.49E+00	6.52E-01	1173	469	1092	648	1008	449	938	644	848	452	762	650	686	461	614	623	515	498	632	765	661	643
MarDSC_7	M_Gr_Wol	4.53E+00	6.56E-01	1171	521	1059	695	993	470	876	684	794	463	697	674	607	485	538	666	425	543	561	812	575	686
MarDSC_8	M_Gr_Wol	4.57E+00	6.60E-01	1147	505	1044	667	972	465	880	670	785	459	697	670	607	475	548	651	424	525	554	796	594	665
MarDSC_9	M_Gr_Wol	4.47E+00	6.50E-01	1224	492	1133	669	1055	474	961	664	896	465	784	664	717	466	651	632	544	505	627	780	690	655
MarDSC_10	M_Gr_Wol	4.35E+00	6.38E-01	1167	493	1098	675	1007	475	938	666	854	478	764	661	692	471	612	638	520	502	630	771	659	656
MarDSC_11	M_Gr_Wol	4.25E+00	6.28E-01	1094	454	1022	642	937	435	858	619	775	429	677	619	603	437	562	601	436	483	556	731	599	620
MarDSC_14	M_Gr_Wol	4.60E+00	6.63E-01	1208	472	1113	648	1034	439	935	632	859	432	754	634	671	440	611	605	494	485	610	765	651	623
MarDSC_15	M_Gr_Wol	3.97E+00	5.99E-01	1092	448	1024	621	938	430	876	631	785	437	719	629	638	450	601	605	487	494	596	739	629	623
MarDSC_16	M_Gr_Wol	4.19E+00	6.22E-01	1167	465	1078	651	1011	450	905	635	835	442	725	643	667	450	604	631	492	505	611	756	639	643
MarDSC_17	M_Gr_Wol	4.36E+00	6.39E-01	1141	563	1059	741	994	547	905	728	838	530	739	713	672	524	600	689	488	563	598	840	643	706
MarDSC_18	M_Gr_Wol	4.67E+00	6.69E-01	1231	476	1126	640	1056	431	944	638	862	429	756	644	689	445	602	618	511	502	635	778	660	643
MarDSC_19	M_Gr_Wol	4.36E+00	6.39E-01	1152	463	1064	642	985	440	925	622	825	443	745	625	666	445	598	599	494	485	618	741	637	610
MarDSC_20	M_Gr_Wol	4.65E+00	6.67E-01	1242	429	1160	615	1070	402	976	610	901	399	793	605	703	409	649	576	527	469	647	737	689	588
Guern_1429	M_Guer	5.12E+00	7.09E-01	904	285	839	467	755	273	685	456	604	282	510	460	444	295	379	424	268	341	398	591	407	451
Guern_1431	M_Guer	5.25E+00	7.20E-01	896	281	829	460	762	273	671	448	594	273	489	450	419	284	356	412	252	329	369	566	388	436
Guer_1433	M_Guer	5.14E+00	7.11E-01	879	328	818	490	742	314	656	477	593	315	492	479	441	317	360	444	282	353	362	588	404	464
Guer_1435	M_Guer	5.48E+00	7.39E-01	966	270	883	457	823	261	725	444	658	267	543	445	492	273	424	413	318	315	419	568	456	432
Guer_1438	M_Guer	4.41E+00	6.44E-01	906	321	797	467	762	292	667	433	604	292	508	439	464	288	408	407	316	317	393	573	425	430
Guer_1440	M_Guer	5.34E+00	7.28E-01	925	236	873	411	776	234	709	411	616	254	526	444	460	270	387	411	287	318	417	571	423	423
Guer_1442	M_Guer	4.86E+00	6.87E-01	896	253	835	431	763	249	691	421	606	259	528	422	446	272	403	384	309	321	433	547	421	407
Guer_1444	M_Guer	5.08E+00	7.06E-01	875	303	812	480	739	283	658	458	580	289	488	454	425	297	363	428	267	334	360	580	393	441
Guer_1446	M_Guer	5.21E+00	7.17E-01	919	241	846	418	775	226	693	407	608	233	504	412	445	244	366	391	265	288	372	545	405	417
Guer_1448	M_Guer	5.62E+00	7.50E-01	971	289	887	477	824	271	712	462	661	268	527	473	499	277	407	434	317	322	397	600	438	451
Guer_1450	M_Guer	5.22E+00	7.18E-01	882	284	811	460	743	272	652	438	578	270	470	441	411	269	346	412	243	308	349	550	379	436
Guer_1452	M_Guer	5.43E+00	7.35E-01	904	285	850	472	771	274	694	452	619	278	512	453	456	275	376	415	292	298	392	566	421	432
Guer_1454	M_Guer	5.27E+00	7.22E-01	879	292	804	468	736	275	651	450	587	275	481	442	431	275	360	406	273	301	356	551	402	424
Guer_1456	M_Guer	5.27E+00	7.22E-01	907	281	843	465	764	273	695	448	620	281	515	450	461	280	371	417	310	304	394	562	427	433
Guer_1458	M_Guer	5.28E+00	7.23E-01	918	271	864	465	763	270	682	454	597	275	503	462	436	285	378	419	291	329	377	586	399	445
Guer_1460	M_Guer	5.24E+00	7.19E-01	928	291	840	461	780	276	695	444	623	276	519	436	461	270	400	414	286	303	391	566	434	432

Guer_1462	M_Guer	4.86E+00	6.87E-01	889	217	824	406	748	219	665	391	582	216	486	399	421	223	347	367	247	260	376	523	395	387
Guer_1464	M_Guer	4.80E+00	6.81E-01	950	319	871	501	810	301	711	474	649	290	542	462	484	286	410	429	310	316	408	565	448	443
Guer_1466	M_Guer	4.93E+00	6.93E-01	906	269	827	456	756	258	667	440	588	266	485	449	414	273	335	427	242	308	362	581	391	431
Guer_1468	M_Guer	4.97E+00	6.96E-01	925	264	852	440	783	250	703	418	624	255	527	423	458	259	384	398	291	294	408	543	432	410
Guer_1470	M_Guer	5.47E+00	7.38E-01	942	285	878	471	793	274	705	462	626	285	524	476	456	293	383	448	280	344	392	619	428	457
Guer_1472	M_Guer	5.05E+00	7.03E-01	871	268	818	443	746	261	667	420	599	264	503	429	451	264	377	400	298	296	383	554	407	418
Guer_1474	M_Guer	5.38E+00	7.31E-01	924	230	856	425	778	228	696	415	613	236	510	423	455	248	380	386	280	284	405	568	413	405
Guer_1476	M_Guer	5.43E+00	7.35E-01	922	261	848	434	779	249	698	417	622	256	530	416	475	258	395	391	302	292	410	532	436	403
Guer_1478	M_Guer	5.25E+00	7.20E-01	901	278	831	449	773	264	681	431	616	263	501	434	458	267	386	399	288	308	386	553	420	416
Guer_1480	M_Guer	5.26E+00	7.21E-01	912	217	853	409	768	218	694	404	611	229	524	415	455	239	394	382	290	283	409	538	423	402
Guer_1482	M_Guer	5.35E+00	7.28E-01	943	260	872	444	795	249	714	430	630	254	524	434	457	268	396	395	276	315	398	553	430	410
Guer_1486	M_Guer	5.19E+00	7.15E-01	896	264	836	447	750	257	677	443	595	271	516	449	427	276	358	417	272	318	385	571	411	433
Guer_1536	M_Guer	4.94E+00	6.94E-01	901	296	827	469	763	282	672	452	610	274	498	444	452	274	366	400	277	305	367	555	394	416
Guer_1538	M_Guer	5.19E+00	7.15E-01	971	304	880	470	819	289	717	454	666	286	551	445	514	278	438	400	351	297	421	544	461	419
Guer_1540	M_Guer	5.22E+00	7.18E-01	895	294	813	449	753	279	669	433	605	279	512	435	456	280	398	399	303	314	388	545	418	417
Guer_1542	M_Guer	5.17E+00	7.13E-01	919	267	839	441	775	254	669	426	631	245	506	408	465	240	394	365	282	276	376	528	425	390
Guer_1544	M_Guer	5.27E+00	7.22E-01	914	290	843	454	773	276	680	439	614	275	509	428	455	277	379	406	276	300	380	562	422	424
Guer_1546	M_Guer	4.88E+00	6.88E-01	966	282	878	461	816	267	717	448	652	264	539	441	486	265	408	417	300	311	399	578	444	434
Guer_1549	M_Guer	4.62E+00	6.65E-01	961	231	872	419	779	228	707	407	614	232	534	410	448	243	388	390	263	291	398	556	422	403
Hung_6805	M_Hung	4.13E+00	6.16E-01	846	287	795	422	721	269	649	425	579	266	495	436	433	282	388	433	280	343	400	555	424	445
Hung_6806	M_Hung	4.57E+00	6.60E-01	888	301	861	460	769	289	699	463	621	300	533	476	465	325	397	462	308	381	423	594	446	477
Hung_6807	M_Hung	4.77E+00	6.79E-01	886	253	830	414	746	245	690	418	599	258	522	436	433	286	363	441	270	344	397	579	432	440
Hung_6808	M_Hung	4.22E+00	6.25E-01	933	237	877	412	804	229	727	416	646	238	569	421	491	261	455	408	346	322	458	538	490	418
Hung_6809	M_Hung	4.46E+00	6.49E-01	888	313	845	484	761	299	706	472	621	297	548	479	452	319	412	475	297	365	414	610	459	485
Hung_6810	M_Hung	4.36E+00	6.39E-01	867	307	817	469	732	308	659	472	574	318	506	485	417	341	386	480	262	396	378	603	426	487
Hung_6811	M_Hung	4.51E+00	6.54E-01	901	258	839	426	756	253	693	421	593	255	523	407	425	262	349	397	276	297	382	520	414	396
Hung_6812	M_Hung	4.58E+00	6.61E-01	909	263	850	431	768	249	700	429	610	252	535	433	447	282	393	424	286	336	413	545	439	439
Hung_6813	M_Hung	4.61E+00	6.64E-01	875	266	826	436	733	258	671	442	576	264	520	449	421	287	402	449	259	356	392	568	444	450
Hung_6814	M_Hung	4.91E+00	6.91E-01	923	256	852	432	776	240	705	408	609	237	536	412	447	252	384	397	268	305	394	540	440	406
Hung_6815	M_Hung	4.24E+00	6.27E-01	837	278	797	439	719	276	667	438	578	276	499	451	427	293	384	441	295	336	380	570	419	449
Hung_6816	M_Hung	4.73E+00	6.75E-01	956	307	901	458	814	275	743	452	642	279	576	458	463	307	404	468	293	364	440	583	472	460
Hung_6817	M_Hung	4.44E+00	6.47E-01	906	339	844	485	769	315	700	481	620	310	537	482	460	325	419	468	291	370	399	590	456	479
Hung_6818	M_Hung	4.51E+00	6.54E-01	938	278	856	433	809	249	716	419	655	236	564	411	498	248	422	401	338	290	439	531	475	416
Hung_6819	M_Hung	4.26E+00	6.29E-01	858	233	801	406	725	230	652	400	584	231	512	405	432	258	411	401	293	311	392	530	447	414
Hung_6820	M_Hung	4.18E+00	6.21E-01	825	297	777	462	699	287	645	456	567	295	497	458	425	317	388	465	282	373	393	579	433	469
Hung_6821	M_Hung	4.33E+00	6.36E-01	900	242	832	390	752	207	692	377	598	210	534	381	435	229	402	363	278	291	402	500	443	373
Hung_6822	M_Hung	4.41E+00	6.44E-01	891	299	833	446	756	285	692	439	614	291	534	443	449	301	395	419	286	336	400	553	451	437
Hung_6824	M_Hung	4.28E+00	6.31E-01	864	278	812	435	737	261	686	418	590	259	528	415	433	272	365	410	277	322	404	530	433	413
Hung_6825	M_Hung	4.40E+00	6.43E-01	844	304	813	493	715	312	663	489	578	326	513	511	428	343	380	494	278	389	408	625	423	495
Hung_6826	M_Hung	4.23E+00	6.26E-01	814	306	772	445	674	284	631	448	531	290	467	458	385	310	333	463	239	366	373	578	390	467
Hung_6827	M_Hung	4.36E+00	6.39E-01	885	337	825	494	756	332	700	489	626	330	536	493	468	344	419	481	328	385	427	593	462	491
Hung_6828	M_Hung	4.11E+00	6.14E-01	859	264	788	408	721	250	649	411	585	253	505	420	419	278	387	413	288	331	387	523	446	424
Hung_6839	M_Hung	4.12E+00	6.15E-01	848	324	801	473	724	315	673	475	587	313	517	474	449	328	394	468	304	359	410	583	437	478
Ital_6893	M_Ital	4.28E+00	6.31E-01	829	270	777	409	690	259	642	406	537	273	483	427	388	298	347	430	252	338	371	540	399	428
Ital_6894	M_Ital	4.10E+00	6.13E-01	850	281	781	421	719	263	653	420	586	262	516	416	446	281	390	410	308	317	401	499	449	409
Ital_6895	M_Ital	4.24E+00	6.27E-01	756	331	725	487	639	325	591	496	501	338	433	521	343	368	313	525	201	438	344	630	350	525
Ital_6896	M_Ital	3.78E+00	5.77E-01	788	291	741	415	685	281	619	428	571	287	489	441	440	317	406	447	319	368	399	552	455	451
Ital_6897	M_Ital	3.80E+00	5.80E-01	779	358	738	497	673	348	612	499	549	350	481	493	424	361	384	479	303	390	391	590	414	487
Ital_6898	M_Ital	4.33E+00	6.36E-01	834	303	785	458	704	297	648	455	548	303	485	481	397	330	341	482	255	399	368	598	399	475
Bur_233	M_Or_Bur	4.96E+00	6.95E-01	1135	510	1066	663	984	481	906	648	819	469	742	637	662	474	616	623	516	505	623	754	663	630
Bur_234	M_Or_Bur	5.31E+00	7.25E-01	1160	480	1108	644	1000	476	948	637	841	472	769	651	674	492	626	643	527	512	664	774	685	646
Main_148	M_Or_Main	4.99E+00	6.98E-01	1115	519	1065	691	974	505	904	678	812	490	737	666	649	499	603	648	499	540	626	777	649	643
Main_149	M_Or_Main	4.31E+00	6.34E-01	1117	511	1067	681	990	504	929	665	857	494	798	663	729	502	699	648	601	541	693	758	740	651
Main_161	M_Or_Main	5.17E+00	7.13E-01	1109	483	1063	648	982	465	895	633	803	458	730	631	635	471	601	625	473	515	602	752	645	625
Main_162	M_Or_Main	5.37E+00	7.30E-01	1165	524	1099	695	1021	501	931	681	835	501	751	677	655	504	594	661	490	546	624	799	657	646
Main_167	M_Or_Main	5.43E+00	7.35E-01	1221	500	1152	695	1063	487	978	689	891	491	807	686	710	505	663	670	561	549	674	801	721	656
Main_168	M_Or_Main	4.81E+00	6.82E-01	1098	475	1039	645	963	463	890	623	806	450	737	618	645	454	629	603	497	492	609	732	667	615
Main_169	M_Or_Main	5.00E+00	6.99E-01	1135	525	1075	697	995	518	918	689	828	516	761	696	660	532	641	675	503	579	635	806	677	687
Main_170	M_Or_Main	5.18E+00	7.14E-01	1193	518	1098	694	1032	504	929	659	860	493	748	658	689	505	624	640	533	553	647	775	668	642
Main_172	M_Or_Main	4.89E+00	6.89E-01	1103	512	1048	683	962	508	887	670	796	510	726	673	644	502	616	643	499	534	608	769	651	647
Main_200	M_Or_Main	4.82E+00	6.83E-01	1162	492	1098	653	1008	485	943	646	852	479	789	646	720	479	689	625	567	530	669	765	721	645
Main_209	M_Or_Main	4.81E+00	6.82E-01	1126	471	1054	632	994	455	907	615	844	443	753	606	693	452	648	592	535	485	647	718	686	601
Main_211	M_Or_Main	4.82E+00	6.83E-01	1081	476	1023	643	939	463	860	623	784	449	692	624	632	456	589	602	486	480	579	717	629	608
Main_216	M_Or_Main	5.21E+00	7.17E-01	1237	476	1155	659	1086	454	996	636	915	452	827	637	732	465	697	630	587	510	718	778	743	628
Main_218	M_Or_Main	5.28E+00	7.23E-01	1212	502	1131	669	1052	477	959	643	886	470	784	640	711	477	656	621	542	519	657	753	701	617
Main_241	M_Or_Main	4.63E+00	6.66E-01	1081	471	1034	651	948	474	876	650	800	475	725	643	656	485	612	632	522	514	622	762	658	636
Main_242	M_Or_Main	5.04E+00	7.02E-01	1166	491	1119	677	1021	482	959	670	846	487	785	675	679	508	637	666	534	557	679	790	695	655
Main_284	M_Or_Main	4.90E+00	6.90E-01	1103	450	1034	634	949	437	886	613	802	433	714	611	631	449	603	592	488	494	637	714	640	600
Main_286	M_Or_Main	4.79E+00	6.80E-01	1106	488	1058	666	964	481	896	650	808	483	740	650	663	497	626	633	510	525	641	756	664	639
Main_289	M_Or_Main	5.33E+00	7.27E-01	1138	483	1081	658	988	466	918	647	820	471	745	646	657	480	590	644	499	521	618	777	656	632
Ork_Main_1308	M_Or_Main	4.60E+00	6.63E-01	886	261	824	434	749	237	673	431	600	241	519	430	457	248	379	431	309	275	408	551	438	417
Ork_Main_1310	M_Or_Main	5.14E+00	7.11E-01	933	318	857	501	787	301	690	479	620	296	521	474	451	306	381	460	283	342	386	597	440	462
Ork_Main_1312	M_Or_Main	4.87E+00	6.88E-01	960	292	891	479	816	271	710	465	634	260	525	457	437	280	360	447	277	317	402	592	436	438
Ork_Main_1317	M_Or_Main	5.08E+00	7.06E-01	925	250	868	429	775	231	696	418	605	230	528	423	445	248	378	405	283	287	391	550	439	405
Ork_Main_1319	M_Or_Main	5.20E+00	7.16E-01	882	255	825	453	739	241	631	445	536	245	455	447	358	265	286	429	204	303	360	576	349	423
Ork_Main_1321	M_Or_Main	5.19E+00	7.15E-01	962	297	893	473	804	278	714	467	629	275	530	467	452	288	396	458	283	339	410	599	443	458
Ork_Main12432	M_Or_Main	5.20E+00	7.16E-01	1528	477	1392	754	1271	437	1137	727	992	442	873	723	733	473	651	720	492	556	719	920	738	710
Ork_Main12436	M_Or_Main	5.14E+00	7.11E-01	1535	667	1440	934	1323	639	1175	917	1066	635	919	915	811	642	711	891	597	704	752	1094	792	892
Ork_Main12442	M_Or_Main	5.13E+00	7.10E-01	1588	422	1451	696	1335	403	1192	691	1078	413	938	689	823	444	751	680	602	508	792	898	819	679
Ork_Main12443	M_Or_Main	5.13E+00	7.10E-01	1476	472	1390	727	1273	446	1140	710	1019	444	890	699	769	458	685	682	530	513	719	887	780	685
Ork_Main12444	M_Or_Main	5.13E+00	7.10E-01	1506	606	1388	884	1280	584	1135	861	1019	575	868	856	773	585	668	822	540	646	702	1025	749	823
Ork_Main_611	M_Or_Main	5.22E+00	7.18E-01	1594	610	1462	924	1326	573	1172	895	1041	574	889	895	758	602	663	863	477	690	690	1115	748	861
Ork_Main627	M_Or_Main	5.32E+00	7.26E-01	1656	374	1509	678	1385	332	1211	644	1090	317	909	641	777	342	639	617	517	408	684	854	760	612

Ork_Main643	M_Or_Main	5.29E+00	7.23E-01	1662	525	1531	839	1384	493	1226	812	1056	495	890	835	738	541	662	810	456	622	695	1063	733	819
Ork_Main659	M_Or_Main	5.42E+00	7.34E-01	1709	595	1568	900	1424	537	1246	858	1106	530	902	859	767	547	614	844	436	644	646	1095	724	835
Ork_Main676	M_Or_Main	5.24E+00	7.19E-01	1717	342	1587	666	1453	310	1258	641	1114	330	950	658	799	346	697	627	528	429	743	885	792	630
Ork_Main693	M_Or_Main	5.76E+00	7.60E-01	1495	622	1397	861	1267	591	1138	856	1009	608	875	863	751	641	656	847	495	720	697	1034	755	851
Ork_Main_709	M_Or_Main	4.98E+00	6.97E-01	1516	598	1402	869	1268	563	1134	861	1007	561	860	866	738	576	675	835	487	634	655	1058	748	841
Ork_Main725	M_Or_Main	5.72E+00	7.57E-01	1667	346	1528	639	1395	298	1231	617	1077	288	914	608	755	307	645	564	450	393	672	820	739	576
Ork_Main741	M_Or_Main	5.36E+00	7.29E-01	1658	525	1536	820	1373	474	1224	807	1062	478	904	810	750	515	643	798	468	610	685	1039	750	783
Ork_Main757	M_Or_Main	5.46E+00	7.37E-01	1621	583	1495	885	1353	559	1194	859	1031	576	882	871	721	610	634	832	443	695	660	1091	726	849
Ork_Main962	M_Or_Main	5.36E+00	7.29E-01	1646	593	1501	910	1380	559	1217	885	1080	558	923	912	811	581	702	868	511	666	694	1098	785	874
Ork_Main978	M_Or_Main	5.04E+00	7.02E-01	1711	524	1573	852	1417	510	1267	830	1112	503	936	859	760	542	673	841	465	646	728	1078	792	832
Ork_Main1011	M_Or_Main	5.47E+00	7.38E-01	1723	530	1560	864	1465	497	1258	842	1155	469	950	829	821	486	684	815	551	578	719	1049	809	802
Ork_Main1028	M_Or_Main	5.35E+00	7.28E-01	1762	469	1595	795	1472	419	1284	758	1129	403	972	758	817	420	716	705	523	500	751	981	812	697
Ork_Main1046	M_Or_Main	4.91E+00	6.91E-01	1662	552	1524	893	1363	527	1217	893	1062	534	897	900	739	547	665	873	453	624	638	1103	750	871
Ork_Main1063	M_Or_Main	4.63E+00	6.66E-01	1670	612	1562	985	1397	586	1223	951	1080	576	907	935	748	595	623	907	448	658	685	1146	755	895
Rou_205	M_Or_Rou	5.07E+00	7.05E-01	1209	471	1121	636	1040	447	955	625	883	446	790	615	715	457	657	609	547	505	677	741	711	602
Ork_Rous_1359	M_Or_Rou	4.95E+00	6.95E-01	938	294	858	487	788	281	686	464	620	266	506	449	444	266	376	426	273	296	390	573	411	430
Ork_Rous_1361	M_Or_Rou	5.25E+00	7.20E-01	943	269	867	451	795	250	684	440	628	245	549	415	456	252	375	414	292	289	393	561	435	413
Ork_Rou_1363	M_Or_Rou	5.11E+00	7.08E-01	950	318	855	514	785	289	679	503	597	303	496	510	418	320	355	497	238	379	367	649	407	501
Ork_Rou_1365	M_Or_Rou	5.00E+00	6.99E-01	985	251	888	457	813	227	709	438	620	231	522	439	437	241	377	419	246	302	404	584	430	423
Ork_Rou773	M_Or_Rou	5.18E+00	7.14E-01	1589	367	1451	663	1322	324	1166	647	1030	319	876	635	760	332	625	595	476	383	692	824	720	588
Ork_Rou793	M_Or_Rou	5.42E+00	7.34E-01	1661	642	1533	947	1404	595	1237	924	1087	598	933	932	805	606	669	904	463	701	723	1116	778	875
Ork_Rou811	M_Or_Rou	5.02E+00	7.01E-01	1709	598	1596	947	1441	575	1279	926	1109	576	953	924	778	593	692	888	481	663	701	1139	769	890
Ork_Rou829	M_Or_Rou	5.03E+00	7.02E-01	1728	426	1582	721	1466	390	1296	686	1174	365	1014	683	879	365	778	637	574	426	804	865	851	647
Ork_Rou845	M_Or_Rou	5.34E+00	7.28E-01	1638	575	1514	863	1382	549	1228	849	1094	532	937	824	797	534	697	793	525	590	733	1014	778	786
San_257	M_Or_San	4.84E+00	6.85E-01	1151	499	1104	661	1014	494	944	659	856	492	772	664	699	512	632	655	545	546	671	773	681	668
San_266	M_Or_San	4.75E+00	6.77E-01	1107	556	1055	711	957	548	898	694	799	542	728	695	644	553	616	677	503	575	619	789	647	690
San_282	M_Or_San	4.92E+00	6.92E-01	1154	496	1105	669	1019	492	942	661	858	490	772	654	702	508	636	639	543	531	671	764	685	648
Ork_San_1371	M_Or_San	5.00E+00	6.99E-01	926	221	885	425	765	242	686	423	590	236	505	441	401	261	351	418	243	310	395	560	407	436
Ork_San_1373	M_Or_San	5.14E+00	7.11E-01	934	175	865	364	754	164	669	360	566	165	484	368	378	192	316	367	198	250	363	509	369	378
Ork_San_1375	M_Or_San	5.29E+00	7.23E-01	963	319	876	510	796	298	685	492	615	291	500	494	427	306	371	472	246	345	373	625	415	492
Ork_San_1377	M_Or_San	4.82E+00	6.83E-01	928	303	866	487	786	295	704	469	616	291	523	466	445	304	395	462	289	341	412	591	447	478
Ork_San1379	M_Or_San	4.84E+00	6.85E-01	933	326	859	510	764	315	674	500	581	306	483	500	399	322	339	490	224	364	368	628	395	501
Ork_San1381	M_Or_San	5.03E+00	7.02E-01	931	289	855	466	780	281	683	450	601	267	494	456	425	283	366	443	232	336	372	576	405	462
Ork_San_1383	M_Or_San	5.18E+00	7.14E-01	935	321	859	512	780	303	681	498	599	301	493	506	415	321	355	496	244	381	370	640	407	498
Ork_San1385	M_Or_San	5.24E+00	7.19E-01	949	304	862	478	782	279	683	459	608	277	503	463	439	281	381	436	270	325	381	578	422	450
Ork_San1387	M_Or_San	4.83E+00	6.84E-01	897	302	835	478	748	297	664	468	581	293	490	479	409	313	345	471	254	347	373	591	401	479
Ork_San1389	M_Or_San	5.12E+00	7.09E-01	920	269	862	459	761	266	674	461	585	266	493	469	402	286	359	463	244	334	381	603	422	472
Ork_San1391	M_Or_San	5.04E+00	7.02E-01	918	311	834	494	765	294	659	482	585	288	492	480	417	299	346	466	244	340	373	604	408	475
Ork_San1393	M_Or_San	5.41E+00	7.33E-01	947	263	853	458	771	251	669	440	569	249	484	453	391	269	328	453	214	335	356	577	389	451
Ork_San519	M_Or_San	4.70E+00	6.72E-01	1509	448	1439	706	1299	449	1185	706	1044	447	937	716	796	483	746	699	560	542	764	885	806	723
Ork_San536	M_Or_San	5.15E+00	7.12E-01	1638	599	1538	877	1360	526	1210	855	1056	520	877	870	739	555	627	845	442	623	664	1083	713	859
Ork_San559	M_Or_San	5.32E+00	7.26E-01	1738	542	1594	849	1414	499	1251	834	1110	495	916	856	791	529	685	829	495	615	714	1072	770	854
Ork_San577	M_Or_San	4.92E+00	6.92E-01	1684	573	1566	906	1385	529	1216	881	1073	524	889	890	764	548	602	865	442	622	677	1099	730	873
Ork_San594	M_Or_San	5.36E+00	7.29E-01	1487	663	1394	919	1235	642	1120	899	996	644	853	922	724	690	624	919	492	754	684	1094	727	926
Ork_Shap862	M_Or_Sha	5.11E+00	7.08E-01	1743	383	1590	686	1456	335	1275	656	1141	332	962	661	811	363	731	629	512	444	733	861	804	632
Ork_Shap878	M_Or_Sha	4.90E+00	6.90E-01	1767	547	1617	864	1484	503	1306	842	1146	491	982	856	816	525	739	832	555	620	746	1069	834	830
Ork_Shap894	M_Or_Sha	5.03E+00	7.02E-01	1701	688	1567	990	1417	652	1280	964	1101	649	948	954	806	668	702	941	473	756	729	1188	790	949
SRon_132	M_Or_Sro	5.12E+00	7.09E-01	1168	498	1103	684	1020	490	921	672	845	482	752	663	673	490	642	637	526	529	643	787	678	642
SRon_133	M_Or_Sro	4.82E+00	6.83E-01	1122	548	1062	712	973	542	900	691	819	529	742	678	648	549	635	674	506	588	648	790	679	681
SRon_134	M_Or_Sro	5.13E+00	7.10E-01	1180	496	1098	677	1025	485	927	663	846	482	754	664	668	503	634	656	526	546	657	793	667	662
SRon_135	M_Or_Sro	5.15E+00	7.12E-01	1152	482	1078	656	997	472	912	645	822	468	735	643	640	489	613	631	511	543	644	773	649	634
SRon_136	M_Or_Sro	5.02E+00	7.01E-01	1132	446	1049	625	983	433	895	603	806	431	725	599	642	445	597	582	487	487	614	728	635	595
SRon_137	M_Or_Sro	5.36E+00	7.29E-01	1196	537	1108	719	1032	514	924	686	849	505	741	688	653	530	629	665	505	574	636	822	664	669
SRon_138	M_Or_Sro	5.24E+00	7.19E-01	1180	461	1087	652	1017	457	908	629	838	445	739	626	665	457	616	602	506	507	646	753	664	611
SRon_157	M_Or_Sro	5.08E+00	7.06E-01	1206	522	1096	700	1043	502	932	665	874	482	766	642	688	491	641	636	541	534	659	774	675	641
SRon_158	M_Or_Sro	5.11E+00	7.08E-01	1138	485	1054	652	989	470	898	632	819	463	723	619	634	480	603	618	484	530	626	742	642	620
SRon_159	M_Or_Sro	4.75E+00	6.77E-01	1155	492	1092	652	1018	479	928	635	866	465	769	623	701	475	658	609	555	512	673	739	693	617
SRon_165	M_Or_Sro	4.73E+00	6.75E-01	1173	450	1109	602	1031	437	949	593	883	423	789	574	718	435	664	567	575	469	695	697	703	574
SRon_166	M_Or_Sro	5.19E+00	7.15E-01	1142	530	1070	706	992	508	899	690	813	513	731	689	643	537	588	698	482	591	626	819	646	684
SRon_204	M_Or_Sro	4.75E+00	6.77E-01	1113	483	1050	650	977	470	884	637	814	468	732	626	656	478	612	607	520	504	626	741	651	612
SRon_206	M_Or_Sro	4.98E+00	6.97E-01	1141	489	1070	661	992	476	898	642	828	475	730	636	646	486	598	624	509	526	634	755	640	629
SRon_207	M_Or_Sro	5.22E+00	7.18E-01	1175	482	1111	656	1023	471	941	655	854	478	772	648	681	500	636	644	517	544	685	774	676	644
Ork_SRon910	M_Or_Sro	4.99E+00	6.98E-01	1714	463	1585	808	1475	452	1292	781	1153	463	980	805	836	513	778	781	539	615	790	1047	833	798
Ork_SRon926	M_Or_Sro	5.23E+00	7.19E-01	1563	619	1463	913	1301	591	1136	897	990	585	814	912	677	624	582	897	404	697	616	1117	670	917
Wes_106	M_Or_Wes	4.95E+00	6.95E-01	1174	499	1073	669	996	480	905	651	832	474	739	659	686	485	624	655	506	559	618	774	667	663
Wes_20	M_Or_Wes	4.64E+00	6.67E-01	1103	519	1020	676	954	503	869	661	799	500	715	665	640	514	604	655	505	551	608	767	638	664
Wes_21	M_Or_Wes	4.80E+00	6.81E-01	1127	479	1033	641	979	464	887	620	813	449	723	622	659	461	600	612	510	499	621	728	653	614
Wes_22	M_Or_Wes	4.62E+00	6.65E-01	1141	523	1081	687	1005	514	926	664	852	514	774	677	714	519	656	663	567	549	662	769	703	668
Wes_32	M_Or_Wes	5.23E+00	7.19E-01	1192	509	1107	681	1038	490	935	650	875	477	754	651	673	491	598	646	515	529	645	764	665	646
Wes_33	M_Or_Wes	5.08E+00	7.06E-01	1130	499	1040	685	974	492	869	659	802	483	703	658	629	493	590	643	472	536	579	766	631	648
Wes_51	M_Or_Wes	4.65E+00	6.67E-01	1116	536	1047	697	974	516	895	672	807	515	734	683	659	523	618	666	520	559	620	786	652	670
Wes_52	M_Or_Wes	5.13E+00	7.10E-01	1204	531	1133	696	1048	502	953	666	875	488	778	661	692	501	652	651	544	543	654	777	697	651
Wes_71	M_Or_Wes	4.73E+00	6.75E-01	1138	505	1081	669	999	493	919	651	861	486	761	656	697	494	647	637	543	532	638	756	692	645
Wes_76	M_Or_Wes	4.34E+00	6.37E-01	1049	501	974	659	906	496	830	650	763	497	684	647	623	503	586	643	499	526	589	742	624	648
Ork_West_1323	M_Or_Wes	5.22E+00	7.18E-01	938	295	847	459	788	276	681	440	630	262	518	435	460	291	381	437	292	316	375	556	452	436
Ork_Wes1325	M_Or_Wes	5.00E+00	6.99E-01	943	226	849	413	779	214	675	392	596	214	490	405	406	238	369	402	250	302	386	535	413	411
Ork_Wes1327	M_Or_Wes	4.92E+00	6.92E-01	961	282	854	449	796	260	678	427	617	246	495	430	424	255	355	420	255	296	375	556	413	421
Ork_Wes1329	M_Or_Wes	5.52E+00	7.42E-01	968	324	870	497	804	296	688	461	620	287	506	463	450	291	380	446	281	339	377	575	432	449
Ork_Wes1331	M_Or_Wes	4.47E+00	6.50E-01	901	293	802	460	741	266	639	444	572	268	469	455	412	283	354	446	256	306	343	564	402	455
Ork_Wes1333	M_Or_Wes	5.25E+00	7.20E-01	919	299	838	462	767	278	672	433	605	275	501	445	431	289	351	445	267	337	406	556	420	440
Ork_Wes1335	M_Or_Wes	4.15E+00	6.18E-01	956	335	852	517	766	317	667	492	586	313	479	491	408	311	379	474	250	345	353	590	430	479
Ork_Wes1337	M_Or_Wes	4.65E+00	6.67E-01	937	325	849	509	749	311	678	496	587	312	508	490	416	329	391	486	266	371	387	604	446	485
Ork_Wes1339	M_Or_Wes	4.73E+00	6.75E-01	935	303	858	475	781	285	683	469	598	291	501	485	440	304	389	476	259	368	371	608	423	484

Ork_Wes1341	M_Or_Wes	5.16E+00	7.13E-01	922	289	850	459	751	290	669	442	583	261	480	455	398	284	352	450	245	330	368	577	405	451
Ork_Wes1343	M_Or_Wes	4.92E+00	6.92E-01	875	320	789	501	730	309	616	484	549	298	440	483	376	310	315	476	213	345	328	601	365	484
Ork_Wes1345	M_Or_Wes	5.15E+00	7.12E-01	943	289	861	475	783	276	692	450	611	273	504	459	436	282	388	455	268	331	375	585	431	460
Ork_Wes1347	M_Or_Wes	4.69E+00	6.71E-01	936	256	857	437	780	242	680	423	606	236	497	431	421	250	365	423	258	283	369	554	411	422
Ork_Wes1349	M_Or_Wes	5.12E+00	7.09E-01	948	266	859	438	794	230	674	413	604	223	489	418	423	238	359	410	247	294	361	540	412	414
Ork_Wes1351	M_Or_Wes	4.90E+00	6.90E-01	915	328	819	483	755	297	657	454	596	293	482	470	415	301	367	478	254	352	370	593	426	472
Ork_Wes1353	M_Or_Wes	5.07E+00	7.05E-01	933	278	846	451	773	256	675	424	601	247	482	433	422	258	362	421	255	298	353	547	411	422
Ork_Wes1355	M_Or_Wes	4.99E+00	6.98E-01	939	307	831	491	770	292	660	460	593	281	483	466	420	294	367	463	261	344	353	589	428	470
Ork_Wes1357	M_Or_Wes	4.96E+00	6.95E-01	962	356	861	517	808	332	696	491	642	320	519	503	473	338	405	497	298	381	395	613	444	499
Ork_Wes12435	M_Or_Wes	4.94E+00	6.94E-01	1494	594	1383	837	1283	558	1138	804	1035	549	876	799	785	570	675	798	532	658	721	992	769	799
Ork_Wes12438	M_Or_Wes	5.28E+00	7.23E-01	1587	468	1440	720	1351	425	1176	670	1078	408	911	679	806	423	721	658	552	485	735	867	780	673
Ork_Wes12439	M_Or_Wes	5.00E+00	6.99E-01	1492	582	1338	823	1256	556	1097	794	1001	548	838	791	742	567	637	784	487	635	657	968	716	785
Ork_Wes12440	M_Or_Wes	5.51E+00	7.41E-01	1556	453	1401	696	1311	410	1133	637	1030	385	849	642	756	398	635	627	478	461	659	818	721	627
Ork_Wes12441	M_Or_Wes	5.30E+00	7.24E-01	1535	667	1413	923	1307	630	1140	880	1028	627	871	903	763	644	668	889	509	720	688	1089	735	885
Spain_1401	M_Spain	4.80E+00	6.81E-01	892	218	839	417	755	213	669	412	578	210	486	414	396	226	313	426	196	297	353	570	391	416
Spain_1403	M_Spain	4.83E+00	6.84E-01	954	265	878	447	800	243	705	430	617	239	504	429	441	248	344	416	261	294	384	565	412	428
Spain_1405	M_Spain	4.82E+00	6.83E-01	953	284	898	481	795	268	728	475	631	265	525	487	446	288	378	462	284	330	393	627	426	487
Spain_1407	M_Spain	4.94E+00	6.94E-01	950	277	865	475	805	255	696	450	603	249	489	464	424	260	352	448	248	319	340	590	393	464
Spain_1409	M_Spain	4.80E+00	6.81E-01	921	295	844	474	764	269	679	464	593	267	475	474	393	284	308	455	195	322	330	624	372	471
Spain_1411	M_Spain	5.14E+00	7.11E-01	958	249	890	415	810	218	727	390	640	214	533	391	456	226	361	397	264	269	421	534	448	392
Spain_1413	M_Spain	5.14E+00	7.11E-01	916	295	829	454	771	263	669	432	590	244	489	408	411	252	346	413	238	295	324	552	391	430
Spain_1415	M_Spain	5.52E+00	7.42E-01	944	321	841	475	785	273	678	446	612	254	479	433	444	261	346	422	255	299	373	580	395	443
Spain_1488	M_Spain	4.12E+00	6.15E-01	915	229	865	400	772	214	711	408	602	221	537	407	425	237	400	408	267	292	406	533	460	403
Spain_1490	M_Spain	4.56E+00	6.59E-01	871	275	813	445	730	262	651	451	572	268	476	451	403	290	352	430	237	341	339	578	387	441
Spain_1493	M_Spain	4.79E+00	6.80E-01	918	271	828	464	755	254	649	456	571	266	453	457	376	282	319	447	192	375	321	576	358	464
Spain_101	M_Spain	4.75E+00	6.77E-01	661	381	614	489	553	364	512	484	436	366	380	484	314	376	269	479	181	411	290	588	313	488
Spain_102	M_Spain	4.18E+00	6.21E-01	615	227	562	351	508	223	457	340	400	223	337	340	281	229	254	337	175	262	258	420	282	346
Spain_103	M_Spain	4.30E+00	6.33E-01	691	281	639	402	583	273	528	391	475	270	409	384	365	274	312	378	254	291	331	463	350	381
Spain_104	M_Spain	4.54E+00	6.57E-01	601	333	568	459	480	330	429	457	358	329	306	447	240	338	197	438	129	361	207	526	241	444
Youg6971	M_Yug	4.72E+00	6.74E-01	820	300	753	454	693	289	616	442	551	281	469	453	399	304	334	438	258	340	351	549	377	446
Youg_6972	M_Yug	4.46E+00	6.49E-01	834	310	800	459	708	309	661	462	573	321	508	483	442	340	403	476	313	382	405	582	437	482
Youg_6973	M_Yug	4.61E+00	6.64E-01	808	365	753	510	695	349	621	510	549	346	487	507	402	365	360	486	255	408	354	602	397	494
Youg_6974	M_Yug	4.43E+00	6.46E-01	827	309	781	455	709	296	652	456	582	304	502	467	442	318	400	453	312	372	403	566	438	462
Youg_6975	M_Yug	4.49E+00	6.52E-01	856	329	822	487	746	324	699	483	622	329	553	496	488	348	436	485	364	386	457	604	469	497
Youg_6976	M_Yug	4.54E+00	6.57E-01	853	239	785	373	730	227	661	362	603	226	503	366	464	233	368	355	317	261	400	468	427	365
Youg_6977	M_Yug	4.64E+00	6.67E-01	860	317	817	473	737	304	680	476	605	317	523	487	463	333	409	472	325	372	432	591	445	485
Youg_6978	M_Yug	4.31E+00	6.34E-01	826	353	774	492	709	330	646	478	568	325	503	491	426	345	391	482	281	411	388	598	429	501
Youg_6979	M_Yug	4.27E+00	6.30E-01	786	260	751	415	677	256	621	415	551	264	477	428	411	282	381	423	289	330	368	529	413	431
Youg_6987	M_Yug	4.45E+00	6.48E-01	885	345	826	486	749	329	688	491	613	330	536	509	465	352	414	489	317	390	436	624	459	498
Youg_6988	M_Yug	4.34E+00	6.37E-01	822	313	785	481	703	308	651	478	563	315	498	481	421	319	382	472	281	353	386	596	419	482
Youg_6989	M_Yug	4.31E+00	6.34E-01	832	336	781	485	711	334	644	476	573	318	492	472	438	321	383	444	286	346	363	563	412	460
Youg_6990	M_Yug	4.04E+00	6.06E-01	828	392	781	544	705	385	652	539	584	387	510	551	450	405	394	541	309	434	401	658	431	553
Youg_6992	M_Yug	4.61E+00	6.64E-01	818	206	764	367	692	191	617	355	553	181	447	359	390	195	301	354	219	247	318	469	367	357
Youg_6993	M_Yug	4.46E+00	6.49E-01	929	247	866	405	784	224	706	396	628	219	542	411	464	236	434	402	317	309	425	505	469	421
Youg_6994	M_Yug	4.36E+00	6.39E-01	868	289	813	440	733	288	666	443	581	288	503	453	432	305	391	436	299	377	374	550	421	446
Youg_6995	M_Yug	4.79E+00	6.80E-01	864	258	810	441	721	248	654	440	555	263	484	458	392	294	362	453	236	364	352	586	399	460
Youg_6997	M_Yug	4.41E+00	6.44E-01	852	332	803	484	730	313	661	483	591	325	518	498	443	341	376	496	303	381	404	616	439	495
Youg_6998	M_Yug	4.49E+00	6.52E-01	811	248	757	409	660	246	616	420	515	260	455	430	361	293	313	420	214	356	347	552	357	431
Youg_6999	M_Yug	4.65E+00	6.67E-01	889	337	832	488	758	320	692	495	617	325	525	498	461	332	405	475	302	382	393	598	444	484
Youg_7000	M_Yug	4.54E+00	6.57E-01	887	324	833	473	760	305	683	467	607	300	514	467	450	310	390	455	295	338	394	574	442	463
Youg_7001	M_Yug	4.11E+00	6.14E-01	829	301	783	459	712	296	640	461	570	294	487	449	430	295	371	439	285	324	384	561	411	448
Youg_7087	M_Yug	4.59E+00	6.62E-01	843	253	795	400	708	246	656	406	569	257	506	432	419	288	368	419	279	337	403	533	416	428
Youg_7088	M_Yug	4.18E+00	6.21E-01	863	276	808	437	739	268	668	424	604	261	518	427	458	262	415	406	307	321	399	520	451	418
Youg_7089	M_Yug	4.53E+00	6.56E-01	850	296	804	467	724	296	672	461	581	306	501	477	425	342	369	484	276	403	390	589	418	486
Youg_7090	M_Yug	4.27E+00	6.30E-01	809	323	761	453	690	312	629	450	548	312	480	463	411	325	360	456	272	367	377	569	405	460
Youg_7091	M_Yug	4.27E+00	6.30E-01	869	311	802	446	740	290	668	433	591	286	520	442	430	307	409	438	299	361	398	548	444	453
Youg_7092	M_Yug	4.39E+00	6.42E-01	872	261	812	428	747	255	677	424	609	262	540	436	465	283	430	427	320	335	434	539	472	431
Youg_7093	M_Yug	4.45E+00	6.48E-01	919	282	868	452	795	284	729	448	656	288	578	454	505	314	471	441	357	357	489	560	498	458
Youg_7094	M_Yug	3.91E+00	5.92E-01	887	305	839	456	782	310	731	457	670	310	598	459	547	315	506	449	415	350	509	558	535	460
EB_130	A_Or_EB	5.22E+00	7.18E-01	854	323	771	467	723	300	637	447	560	296	486	447	412	302	375	427	316	316	376	545	413	428
EB_140	A_Or_EB	5.13E+00	7.10E-01	875	275	801	413	746	278	661	409	606	273	523	407	469	268	420	397	345	308	428	506	457	401
EB_144	A_Or_EB	5.36E+00	7.29E-01	948	315	862	479	805	286	718	454	647	282	564	430	494	285	429	415	341	312	444	539	474	415
EB_150	A_Or_EB	4.87E+00	6.88E-01	834	329	758	489	674	336	619	492	527	347	460	497	398	341	353	482	245	401	357	597	390	488
EB_156	A_Or_EB	5.35E+00	7.28E-01	901	346	838	482	774	320	686	465	626	310	532	464	478	318	412	443	328	350	418	564	460	442
EB_160	A_Or_EB	4.97E+00	6.96E-01	932	271	849	445	760	254	688	431	601	251	509	443	432	269	386	424	273	321	401	556	435	430
EB_168	A_Or_EB	5.41E+00	7.33E-01	957	308	877	482	796	297	699	473	626	290	506	469	447	303	364	455	268	348	388	587	424	456
EB_174	A_Or_EB	5.25E+00	7.20E-01	982	283	871	455	818	263	699	444	614	262	514	440	434	274	375	418	275	311	402	557	432	425
EB_178	A_Or_EB	5.15E+00	7.12E-01	849	278	775	421	708	255	632	413	558	251	480	417	404	260	364	403	266	290	356	518	399	409
EB_199	A_Or_EB	5.29E+00	7.23E-01	940	257	862	408	784	235	700	412	619	227	528	403	443	241	376	383	286	280	409	511	433	382
EB_211	A_Or_EB	5.38E+00	7.31E-01	961	268	884	437	799	246	701	419	622	237	521	402	425	237	376	371	265	268	371	508	421	376
EB_221	A_Or_EB	5.19E+00	7.15E-01	592	294	549	416	490	299	440	414	384	290	322	419	275	311	241	409	165	347	252	505	271	414
EB_222	A_Or_EB	5.19E+00	7.15E-01	625	293	576	409	527	286	466	404	419	280	353	402	320	290	272	395	205	316	276	490	302	391
EB_225	A_Or_EB	5.11E+00	7.08E-01	887	237	821	384	765	221	679	370	632	221	542	371	500	232	442	353	353	263	440	474	478	358
EB_229	A_Or_EB	5.31E+00	7.25E-01	823	324	761	484	680	315	618	480	544	319	470	479	397	334	358	462	254	369	377	592	392	460
Howe_R58	A_Or_How	5.69E+00	7.55E-01	2037	684	1870	980	1771	643	1571	963	1472	638	1274	958	1142	677	1018	949	858	787	1062	1192	1116	955
How_R59	A_Or_How	5.50E+00	7.40E-01	1946	727	1793	1018	1677	680	1510	1005	1381	681	1208	996	1056	702	965	968	744	796	980	1233	1058	970
Howe_R60	A_Or_How	5.53E+00	7.43E-01	1886	755	1737	1058	1634	718	1463	1040	1324	720	1164	1037	1006	747	929	1009	731	845	954	1260	1028	1011
How_2168	A_Or_How	5.53E+00	7.43E-01	948	326	858	497	805	290	689	452	635	269	516	445	457	266	384	416	280	296	389	561	439	417
How_2170	A_Or_How	5.47E+00	7.38E-01	929	304	856	487	780	271	688	470	604	268	516	466	425	278	364	449	274	317	392	586	425	438
How_2172	A_Or_How	5.68E+00	7.54E-01	1002	244	892	427	833	216	703	397	636	201	498	392	436	213	360	360	253	262	377	515	404	373
How_2178	A_Or_How	5.28E+00	7.23E-01	883	350	800	517	749	329	649	499	586	306	492	475	436	309	377	453	292	340	375	580	422	453
How_2180	A_Or_How	5.19E+00	7.15E-01	951	290	876	438	817	270	722	427	663	262	564	422	508	263	463	394	366	290	446	521	498	396
How_2184	A_Or_How	5.64E+00	7.51E-01	946	197	858	365	794	162	692	340	625	151	522	326	458	152	392	295	306	173	401	435	435	299

How_2186	A_Or_How	5.54E+00	7.44E-01	936	196	853	357	797	166	697	344	637	161	525	336	480	169	407	321	298	199	396	446	445	310
How_2190	A_Or_How	5.29E+00	7.23E-01	993	338	888	508	840	311	728	486	674	299	569	469	522	296	464	429	383	316	448	570	497	442
How_2192	A_Or_How	5.45E+00	7.36E-01	951	253	848	407	796	222	680	386	630	212	516	380	459	217	401	367	311	247	394	487	449	364
How_2196	A_Or_How	5.68E+00	7.54E-01	945	301	832	444	793	264	679	423	625	250	504	413	455	249	384	391	301	274	372	522	435	391
How_2198	A_Or_How	5.29E+00	7.23E-01	853	194	762	366	700	167	604	331	546	147	448	322	392	145	329	291	229	172	332	436	370	296
How_2202	A_Or_How	5.73E+00	7.58E-01	967	268	863	444	801	244	698	413	622	233	521	414	452	236	404	376	291	279	405	531	436	391
How_2204	A_Or_How	5.16E+00	7.13E-01	987	150	902	326	827	134	743	305	666	128	561	299	480	139	432	282	332	165	425	421	470	284
How_2206	A_Or_How	5.09E+00	7.07E-01	908	281	840	435	767	258	676	419	601	251	492	425	436	248	380	394	271	283	362	528	409	409
How_2208	A_Or_How	5.46E+00	7.37E-01	954	217	846	398	798	193	676	382	615	181	505	367	422	190	364	352	243	241	368	505	410	336
How_2210	A_Or_How	5.34E+00	7.28E-01	915	170	837	347	766	149	663	331	585	140	485	317	404	144	372	301	250	190	360	439	405	307
How_2212	A_Or_How	5.12E+00	7.09E-01	918	211	861	377	779	186	697	369	614	184	522	363	441	188	400	338	287	224	387	485	453	341
How_2218	A_Or_How	5.38E+00	7.31E-01	991	199	911	383	825	164	731	361	654	164	541	357	470	169	388	336	311	201	423	490	456	336
How_2220	A_Or_How	5.22E+00	7.18E-01	980	181	888	367	808	156	700	355	631	164	525	351	449	172	411	326	280	218	417	478	444	338
How_2222	A_Or_How	5.86E+00	7.68E-01	1077	157	943	355	918	142	765	317	712	123	578	317	507	126	425	293	314	162	435	443	488	296
How_2224	A_Or_How	5.58E+00	7.47E-01	1018	351	915	525	851	321	726	501	671	310	539	477	498	296	396	441	316	324	401	593	456	445
How_2232	A_Or_How	5.61E+00	7.49E-01	998	399	878	535	825	346	705	509	628	332	527	500	465	336	404	481	315	373	413	632	452	486
HPW_R20	A_Or_HPW	5.46E+00	7.37E-01	1959	673	1801	970	1716	632	1516	941	1429	614	1220	954	1137	632	999	932	854	703	1024	1146	1094	938
HPW_R23	A_Or_HPW	5.86E+00	7.68E-01	1945	665	1806	941	1681	626	1487	908	1375	610	1167	904	1055	640	913	905	774	705	945	1131	1034	898
HPW_R25	A_Or_HPW	5.58E+00	7.47E-01	1971	617	1839	929	1727	594	1522	898	1416	576	1226	897	1135	594	999	900	815	695	1034	1109	1112	889
HPW1_1889	A_Or_HPW	5.07E+00	7.05E-01	1031	239	903	420	856	197	719	375	669	177	521	360	465	178	397	338	293	202	376	499	449	356
HPW1_1893	A_Or_HPW	5.40E+00	7.32E-01	987	162	880	363	827	134	702	340	634	127	520	329	447	129	372	303	265	167	381	455	429	309
HPW1_2067	A_Or_HPW	5.22E+00	7.18E-01	927	247	829	391	789	226	686	364	638	204	533	339	489	204	430	319	345	221	408	450	461	338
HPW1_2069	A_Or_HPW	5.45E+00	7.36E-01	883	283	786	470	742	264	621	438	582	251	447	437	388	254	307	428	234	298	330	560	373	421
HPW1_2071	A_Or_HPW	5.69E+00	7.55E-01	988	217	873	389	827	186	712	356	665	171	536	356	473	172	397	341	301	210	408	478	449	338
HPW1_2073	A_Or_HPW	5.54E+00	7.44E-01	940	331	836	492	780	298	668	465	618	284	498	450	440	287	341	446	271	319	360	575	424	436
HPW1_2075	A_Or_HPW	5.12E+00	7.09E-01	885	258	789	418	748	229	641	395	588	216	475	379	430	217	353	358	275	247	355	486	405	362
HPW1_2079	A_Or_HPW	5.73E+00	7.58E-01	929	276	817	436	762	250	661	403	604	227	490	400	435	224	352	368	276	254	360	506	409	375
HPW1_2085	A_Or_HPW	5.70E+00	7.56E-01	1006	301	902	450	863	269	745	416	693	249	571	403	529	251	463	375	370	281	456	519	505	390
HPW1_2089	A_Or_HPW	5.14E+00	7.11E-01	854	259	774	426	721	235	620	394	562	215	454	384	400	222	346	367	250	255	332	498	390	380
HPW1_2091	A_Or_HPW	5.65E+00	7.52E-01	928	272	826	433	785	240	673	408	594	229	496	403	440	235	359	388	278	262	370	521	418	388
HPW1_2093	A_Or_HPW	5.11E+00	7.08E-01	965	145	877	332	809	117	701	309	637	103	527	299	468	97	391	279	281	142	397	425	439	279
HPW2_2061	A_Or_HPW	5.13E+00	7.10E-01	931	192	831	366	775	175	675	342	605	163	495	345	438	167	367	340	273	210	372	470	421	341
HPW2_2065	A_Or_HPW	5.12E+00	7.09E-01	900	261	813	428	747	237	657	403	588	221	476	403	407	233	361	380	259	271	354	524	400	396
HPW2_2132	A_Or_HPW	5.22E+00	7.18E-01	939	315	835	482	790	291	674	446	624	264	509	423	451	262	390	402	294	288	370	546	428	416
HPW2_2134	A_Or_HPW	5.09E+00	7.07E-01	940	179	853	352	790	156	697	318	644	130	520	298	461	131	414	277	309	159	394	417	448	294
HPW2_2136	A_Or_HPW	5.44E+00	7.36E-01	980	211	871	398	821	189	696	376	636	176	511	370	455	190	370	352	285	217	378	493	428	351
LoN_FP88_10_1	A_Or_LoN	5.04E+00	7.02E-01	937	548	886	650	846	530	788	634	740	521	682	627	649	519	594	623	539	553	611	702	631	625
LoN_FP88_10_3	A_Or_LoN	5.50E+00	7.40E-01	994	511	935	620	905	498	829	609	785	497	711	611	679	494	632	599	568	524	622	679	661	603
LoN_FP88_10_4	A_Or_LoN	5.41E+00	7.33E-01	1010	544	938	649	896	526	820	639	780	517	710	630	665	529	638	626	566	555	635	714	668	630
LoN_FP88_10_5	A_Or_LoN	5.72E+00	7.57E-01	997	509	932	617	892	501	826	608	767	497	704	608	654	501	601	608	547	531	614	686	645	602
LoN_FP88_13	A_Or_LoN	5.33E+00	7.27E-01	1046	481	986	606	948	475	877	597	831	484	763	603	727	491	673	597	640	504	691	688	704	593
LoN_FP89_10	A_Or_LoN	5.52E+00	7.42E-01	974	532	921	640	871	516	815	634	762	511	694	634	650	519	599	629	541	552	622	731	640	628
LoN_FP89_11	A_Or_LoN	4.91E+00	6.91E-01	920	500	880	609	835	490	784	594	732	485	681	594	629	490	596	588	540	509	610	665	631	585
LoN_FQ87_5_2	A_Or_LoN	5.23E+00	7.19E-01	940	508	887	616	844	493	784	604	733	488	668	606	629	498	588	602	522	522	601	677	622	596
LoN_FQ87_5_3	A_Or_LoN	5.34E+00	7.28E-01	972	559	920	662	888	537	818	646	772	535	708	647	669	537	619	635	571	552	640	717	660	634
LoN_FQ87_5_4	A_Or_LoN	5.36E+00	7.29E-01	1097	573	1038	685	995	558	934	661	892	545	825	650	787	540	736	638	685	559	746	722	776	636
LoN_FQ87_5_6	A_Or_LoN	5.48E+00	7.39E-01	991	508	925	616	887	486	817	600	776	477	703	601	669	476	602	601	565	495	628	675	656	594
LoN_FQ88_6_1	A_Or_LoN	5.51E+00	7.41E-01	1037	493	972	606	932	473	866	595	817	463	760	585	704	472	666	585	608	495	671	674	704	587
LoN_FQ88_6_2	A_Or_LoN	5.69E+00	7.55E-01	997	496	948	612	902	477	837	594	786	470	712	589	679	468	625	579	570	500	633	672	664	581
LoN_FQ88_6_3	A_Or_LoN	5.12E+00	7.09E-01	934	511	875	634	832	500	778	612	733	495	664	611	624	494	573	599	530	524	584	683	604	599
LoN_FQ88_6_4	A_Or_LoN	5.47E+00	7.38E-01	1017	508	966	627	915	498	854	615	805	491	746	618	685	508	645	622	587	543	684	707	694	609
LoN_FQ88_6_5	A_Or_LoN	5.68E+00	7.54E-01	1097	499	1028	616	990	481	913	596	880	470	798	595	749	472	714	583	647	500	716	675	745	583
LoN_FQ88_6_6	A_Or_LoN	5.39E+00	7.32E-01	1041	496	974	604	932	484	868	580	833	467	757	580	717	468	665	570	623	491	684	649	713	567
LoN_FQ88_6_7	A_Or_LoN	5.30E+00	7.24E-01	1031	558	980	664	936	549	880	656	831	544	773	660	718	553	676	651	616	581	683	723	718	650
LoN_FQ88_6_6	A_Or_LoN	5.18E+00	7.14E-01	870	540	825	649	781	529	720	642	686	529	616	644	575	537	540	631	475	564	542	718	565	630
LoN_FQ88_6_9	A_Or_LoN	5.24E+00	7.19E-01	991	481	931	591	901	464	834	570	802	454	727	566	691	452	647	553	586	471	641	633	674	547
LoN_FR88_4_1	A_Or_LoN	5.42E+00	7.34E-01	1046	527	986	636	940	506	877	623	824	496	760	621	715	499	671	604	613	524	679	687	707	607
LoN_FR88_4_2	A_Or_LoN	5.15E+00	7.12E-01	948	526	892	634	851	514	788	620	742	510	679	622	642	514	607	617	543	537	601	694	635	622
PC_R39	A_Or_PC	5.24E+00	7.19E-01	1838	774	1711	1084	1585	759	1444	1049	1311	758	1134	1053	1036	769	921	1042	768	832	957	1251	1021	1043
PC_R44	A_Or_PC	5.26E+00	7.21E-01	1920	632	1778	924	1671	608	1516	883	1410	605	1217	894	1106	618	1015	859	850	683	1011	1062	1090	866
PC_R45	A_Or_PC	5.11E+00	7.08E-01	1853	661	1700	951	1618	638	1434	924	1328	636	1157	919	1075	651	932	917	807	705	967	1121	1027	897
PC1_2586	A_Or_PC	5.81E+00	7.64E-01	1012	275	907	455	847	243	743	419	673	228	548	403	495	233	423	383	326	247	420	526	479	391
PC1_2595	A_Or_PC	5.41E+00	7.33E-01	983	321	911	495	825	299	727	476	643	289	524	473	446	300	404	453	276	341	404	591	456	458
PC1_2601	A_Or_PC	5.38E+00	7.31E-01	960	335	881	516	804	317	695	490	624	310	508	491	427	320	360	481	263	355	365	602	428	478
PC1_2603	A_Or_PC	5.73E+00	7.58E-01	997	379	899	533	847	350	723	503	658	332	539	499	493	324	391	475	309	347	393	620	447	480
PC1_2605	A_Or_PC	5.54E+00	7.44E-01	962	315	864	493	802	292	691	466	631	284	502	459	455	284	373	440	292	305	384	573	437	444
PC1_2621	A_Or_PC	5.46E+00	7.37E-01	1038	395	910	562	864	354	732	525	678	333	538	518	510	328	386	486	303	357	397	619	446	488
PC1_2623	A_Or_PC	5.37E+00	7.30E-01	1013	353	931	533	855	324	743	506	670	318	543	504	490	325	405	479	321	354	402	622	470	478
PC1_2625	A_Or_PC	5.73E+00	7.58E-01	985	317	887	492	823	286	709	468	634	272	512	465	452	270	364	445	275	301	383	585	432	444
PC1_2627	A_Or_PC	5.18E+00	7.14E-01	945	440	843	609	791	417	683	585	609	406	501	579	433	411	392	548	277	437	363	690	422	564
PC1_2631	A_Or_PC	4.99E+00	6.98E-01	988	378	868	553	809	354	697	520	623	338	523	520	434	344	358	506	272	370	356	640	422	500
PC1_2633	A_Or_PC	5.63E+00	7.51E-01	1022	329	920	514	859	309	737	483	655	297	547	477	481	294	396	453	285	333	404	602	465	463
PC1_2635	A_Or_PC	5.74E+00	7.59E-01	1054	395	921	576	874	361	743	541	679	343	552	528	482	356	377	521	301	392	400	656	458	519
PC1_2637	A_Or_PC	5.45E+00	7.36E-01	970	398	872	581	809	363	678	540	620	337	499	535	434	334	361	499	268	370	363	635	413	501
PC1_2689	A_Or_PC	5.21E+00	7.17E-01	972	256	863	430	815	228	688	398	637	210	506	389	448	206	356	376	263	239	368	505	422	372
PC1_2691	A_Or_PC	5.19E+00	7.15E-01	962	308	868	490	791	301	688	474	602	292	506	470	434	297	364	460	279	336	365	605	420	466
PC2_2588	A_Or_PC	5.20E+00	7.16E-01	994	274	892	441	843	235	726	403	680	219	550	399	479	216	412	376	330	251	426	517	454	376
PC2_2590	A_Or_PC	5.56E+00	7.45E-01	1062	282	922	433	888	237	741	387	694	205	543	375	498	203	397	348	332	228	420	496	468	354
PC2_2669	A_Or_PC	5.66E+00	7.53E-01	1003	320	889	506	827	301	706	479	634	288	514	466	448	287	352	469	266	325	387	613	427	456
PC2_2671	A_Or_PC	5.21E+00	7.17E-01	982	282	886	457	821	259	720	423	655	245	539	417	467	247	393	404	297	284	415	533	451	402
PC2_2673	A_Or_PC	5.46E+00	7.37E-01	990	442	891	619	831	421	725	583	672	406	535	571	475	402	416	541	310	426	403	672	457	553
PC2_2675	A_Or_PC	5.14E+00	7.11E-01	926	405	818	551	761	371	666	532	597	356	481	521	432	355	351	501	268	375	339	623	408	501
PC2_2677	A_Or_PC	5.53E+00	7.43E-01	957	333	863	516	799	313	695	484	629	305	498	487	454	304	346	464	279	333	364	597	408	470

PC2_2679	A_Or_PC	5.47E+00	7.38E-01	982	380	887	542	821	354	717	517	644	343	523	511	448	347	400	484	289	375	361	624	440	498
PC2_2681	A_Or_PC	4.85E+00	6.86E-01	868	395	786	564	715	381	636	541	562	380	469	548	406	384	339	540	244	431	338	661	388	542
PQ1_1668	A_Or_PQ1	5.30E+00	7.24E-01	955	255	852	413	788	230	681	388	606	215	513	390	450	223	380	376	284	251	383	505	435	379
PQ1_1670	A_Or_PQ1	5.44E+00	7.36E-01	940	273	853	416	790	230	687	398	616	217	511	400	446	221	379	380	292	264	385	524	431	380
PQ1_1672	A_Or_PQ1	5.22E+00	7.18E-01	917	206	823	372	758	190	657	350	575	187	479	348	400	199	326	336	236	240	355	464	388	343
PQ1_1676	A_Or_PQ1	5.71E+00	7.57E-01	991	328	881	500	825	295	699	472	629	278	506	472	450	278	328	455	250	320	372	596	407	447
PQ1_1678	A_Or_PQ1	5.61E+00	7.49E-01	976	313	875	487	833	277	691	447	646	243	503	422	462	224	357	387	292	239	360	534	420	385
PQ1_1680	A_Or_PQ1	5.46E+00	7.37E-01	952	204	856	392	795	181	682	359	623	166	501	349	436	172	378	327	267	218	360	467	432	334
PQ1_1682	A_Or_PQ1	5.47E+00	7.38E-01	939	237	823	399	782	198	650	361	607	164	475	340	427	166	356	319	263	186	336	450	401	319
PQ1_1696	A_Or_PQ1	5.21E+00	7.17E-01	925	167	824	322	766	148	676	295	618	133	507	289	454	129	398	272	294	157	380	394	436	281
PQ1_1710	A_Or_PQ1	5.59E+00	7.47E-01	986	282	863	426	832	233	696	382	645	204	507	365	452	199	363	346	283	223	329	478	425	352
PQ1_1714	A_Or_PQ1	5.49E+00	7.40E-01	950	269	835	419	804	229	673	385	636	196	502	358	471	182	368	329	294	200	355	461	418	337
PQ2_1786	A_Or_PQ2	5.55E+00	7.44E-01	774	143	694	286	655	123	568	261	509	116	415	262	367	115	310	249	241	143	317	365	358	247
PQ2_1790	A_Or_PQ2	5.52E+00	7.42E-01	778	183	716	313	665	168	580	300	531	158	440	292	380	154	318	278	246	179	334	384	372	289
PQ2_1792	A_Or_PQ2	5.62E+00	7.50E-01	801	266	726	395	683	238	590	368	543	226	443	370	401	223	341	340	264	242	339	455	377	347
PQ2_1794	A_Or_PQ2	5.71E+00	7.57E-01	897	210	811	367	762	183	670	348	610	181	517	347	456	189	396	335	321	220	429	438	454	320
PQ2_1796	A_Or_PQ2	5.39E+00	7.32E-01	777	387	708	529	662	371	578	503	515	356	435	498	374	362	342	477	260	392	347	585	377	483
PQ2_1800	A_Or_PQ2	4.86E+00	6.87E-01	763	196	698	322	644	186	581	309	532	179	460	307	407	174	361	299	289	207	362	401	403	306
PQ2_1802	A_Or_PQ2	5.41E+00	7.33E-01	788	378	736	512	668	375	594	505	523	376	445	525	382	390	344	515	267	426	367	628	388	519
PQ2_1804	A_Or_PQ2	5.41E+00	7.33E-01	759	274	679	403	630	253	547	394	506	256	407	397	351	264	307	388	228	298	300	499	361	391
PQ2_1806	A_Or_PQ2	5.31E+00	7.25E-01	750	210	664	346	628	185	544	321	489	178	404	308	345	176	294	295	219	203	300	404	332	298
PQ2_1816	A_Or_PQ2	5.22E+00	7.18E-01	836	370	776	507	703	345	620	492	551	334	463	487	410	339	355	486	269	374	350	589	406	468
PQ2_1822	A_Or_PQ2	5.26E+00	7.21E-01	883	327	801	478	740	300	642	455	587	281	473	447	438	271	365	422	288	287	345	555	399	428
PQ2_1826	A_Or_PQ2	5.74E+00	7.59E-01	904	307	801	470	765	279	644	436	604	264	479	438	419	274	340	426	275	293	378	556	415	425
PQ2_1828	A_Or_PQ2	5.57E+00	7.46E-01	889	247	785	389	747	208	637	358	587	190	464	349	434	188	336	327	270	214	339	453	395	330
PQ2_1836	A_Or_PQ2	4.98E+00	6.97E-01	767	147	695	301	645	127	558	278	496	115	405	265	339	118	280	248	205	147	297	371	332	256
PQ2_1836	A_Or_PQ2	5.80E+00	7.63E-01	942	215	846	376	778	203	684	369	617	204	518	377	433	222	358	379	280	263	382	496	436	362
PQ4_1616	A_Or_PQ4	5.40E+00	7.32E-01	787	246	743	387	674	246	622	385	544	252	486	401	421	272	390	408	306	298	393	503	430	399
PQ4_1618	A_Or_PQ4	4.87E+00	6.88E-01	932	262	833	449	765	246	666	425	591	243	483	426	404	258	332	417	233	309	354	557	397	410
PQ4_1622	A_Or_PQ4	5.43E+00	7.35E-01	889	222	797	368	735	208	649	357	570	205	485	365	409	223	354	370	268	268	358	482	408	361
PQ4_1624	A_Or_PQ4	5.25E+00	7.20E-01	897	192	799	354	743	166	632	332	576	153	472	326	401	156	350	312	253	192	363	451	386	315
PQ4_1626	A_Or_PQ4	5.58E+00	7.47E-01	917	173	848	374	750	179	673	373	568	194	489	399	394	227	342	420	240	295	389	553	408	403
PQ4_1632	A_Or_PQ4	5.32E+00	7.26E-01	900	265	831	443	758	253	664	426	587	262	485	447	410	292	374	450	246	353	387	574	419	446
PQ4_1634	A_Or_PQ4	5.57E+00	7.46E-01	952	310	857	467	801	297	697	465	629	305	513	489	440	322	372	499	263	375	399	600	433	469
PQ4_1636	A_Or_PQ4	5.41E+00	7.33E-01	945	259	862	458	784	256	695	466	615	270	520	487	428	307	383	489	277	365	410	620	441	480
PQ4_1642	A_Or_PQ4	5.71E+00	7.57E-01	973	195	884	389	802	173	693	368	597	181	506	393	408	218	379	382	244	273	390	525	430	379
PQ4_1644	A_Or_PQ4	5.32E+00	7.26E-01	881	241	809	426	734	241	644	424	557	253	466	447	379	280	330	445	224	340	366	572	387	443
PQ4_1646	A_Or_PQ4	5.21E+00	7.17E-01	890	344	823	509	739	328	654	511	570	345	477	524	384	367	344	525	239	406	356	644	399	516
POQ4_1650	A_Or_PQ4	5.24E+00	7.19E-01	902	268	840	438	748	271	672	442	597	291	504	458	417	310	371	461	253	363	385	597	422	455
PQ4_1660	A_Or_PQ4	4.97E+00	6.96E-01	890	228	831	403	756	230	673	419	578	255	505	443	430	273	386	445	266	325	386	564	434	442
QU3_3186_1	A_Or_QUA	5.03E+00	7.02E-01	1128	522	1047	667	1004	498	920	636	862	486	765	626	719	490	658	615	572	529	661	734	709	622
QU3_3197_1	A_Or_QUA	5.07E+00	7.05E-01	1037	490	969	644	907	473	830	633	758	474	674	633	604	485	566	624	477	526	584	730	613	622
QU3_3197_2	A_Or_QUA	4.98E+00	6.97E-01	1125	490	1070	632	1016	475	930	618	881	469	783	613	716	466	688	586	583	494	687	692	712	594
QU3_3198_1	A_Or_QUA	5.18E+00	7.14E-01	1080	499	1013	645	950	480	878	634	801	475	727	620	649	475	620	603	510	511	611	725	653	614
QU3_3198_2	A_Or_QUA	4.72E+00	6.74E-01	1084	437	1007	576	963	424	892	574	841	444	751	580	716	445	662	579	587	476	675	685	695	581
QU3_3198_3	A_Or_QUA	4.84E+00	6.85E-01	1135	441	1079	565	1020	424	943	558	888	428	807	563	754	436	709	549	623	463	711	668	743	560
QU3_3198_4	A_Or_QUA	5.36E+00	7.29E-01	1116	416	1054	564	982	398	902	556	834	396	752	544	672	404	626	538	516	440	642	660	675	535
QU3_3198_5	A_Or_QUA	5.47E+00	7.38E-01	1190	493	1097	637	1054	464	949	620	900	454	799	623	747	460	678	605	599	492	686	727	732	603
QU3_3198_6	A_Or_QUA	5.11E+00	7.08E-01	1092	403	1027	556	967	390	888	536	827	383	741	540	696	392	620	530	559	411	636	635	676	527
QU3_3199_1	A_Or_QUA	5.03E+00	7.02E-01	1033	470	991	621	910	461	853	607	773	462	701	620	632	479	585	599	486	509	603	719	630	595
QU3_3199_2	A_Or_QUA	5.22E+00	7.18E-01	1144	491	1056	631	1016	457	912	615	859	464	754	614	726	460	656	596	589	491	654	716	691	600
QU3_3199_3	A_Or_QUA	4.92E+00	6.92E-01	1029	476	986	633	906	467	843	614	769	466	694	606	628	468	599	587	493	499	602	707	631	595
QU3_3202_1	A_Or_QUA	5.35E+00	7.28E-01	1100	486	1033	628	976	464	883	613	822	453	731	604	664	458	622	598	519	497	619	717	660	604
QU3_3202_2	A_Or_QUA	4.89E+00	6.89E-01	1051	471	998	631	924	460	866	621	786	462	725	617	646	467	616	601	515	510	619	713	658	601
QU3_3202_3	A_Or_QUA	5.34E+00	7.28E-01	1103	469	1019	619	969	456	883	598	822	454	724	602	672	463	606	586	539	484	613	700	659	587
QU3_3203_1	A_Or_QUA	4.97E+00	6.96E-01	1093	555	1019	702	967	535	888	678	832	523	745	673	694	529	646	650	563	555	651	776	688	662
QU3_3206_1	A_Or_QUA	5.16E+00	7.13E-01	1050	474	982	617	928	450	834	595	789	437	683	595	654	436	563	587	505	474	581	701	614	578
QU3_3206_2	A_Or_QUA	5.41E+00	7.33E-01	1144	446	1090	604	1024	434	947	582	889	431	789	589	720	444	663	568	578	479	681	691	713	571
QU3_3208_1	A_Or_QUA	5.34E+00	7.28E-01	1142	500	1067	644	1007	479	918	630	866	476	769	618	716	477	663	603	568	510	648	726	694	606
QU3_3211_1	A_Or_QUA	5.28E+00	7.23E-01	1079	472	1020	620	945	461	873	618	795	464	729	626	661	478	594	627	520	526	618	736	657	620
QU3_3212_1	A_Or_QUA	5.65E+00	7.52E-01	1182	458	1091	618	1038	436	937	598	873	435	774	603	707	447	650	589	566	482	675	718	698	586
QU3_3213_1	A_Or_QUA	4.85E+00	6.86E-01	1045	492	978	630	921	480	850	615	788	477	715	606	653	478	609	591	526	499	620	712	649	600
QU3_3213_2	A_Or_QUA	5.21E+00	7.17E-01	1126	451	1059	596	1007	431	922	579	865	421	765	581	713	430	661	550	596	457	649	677	706	558
QU3_3213_3	A_Or_QUA	5.36E+00	7.29E-01	1200	550	1151	705	1071	533	1004	692	930	534	842	690	791	532	718	656	646	555	744	795	769	667
QU3_3133_1	A_Or_QUA	5.45E+00	7.36E-01	1093	472	1022	621	966	449	879	601	815	443	723	599	669	450	601	578	512	485	615	698	650	581
QU3_3137_1	A_Or_QUA	5.42E+00	7.34E-01	1198	521	1101	679	1068	494	971	642	914	477	813	627	772	469	711	607	623	495	705	737	760	611
QU3_3137_2	A_Or_QUA	5.42E+00	7.34E-01	1158	516	1098	676	1025	503	941	671	881	503	790	669	732	508	679	655	603	543	683	779	723	658
QU3_3140_1	A_Or_QUA	5.33E+00	7.27E-01	1069	574	997	740	938	552	855	704	793	541	702	702	642	543	591	676	490	571	580	806	626	681
QU3_3141_1	A_Or_QUA	4.84E+00	6.85E-01	1052	543	961	680	908	527	824	671	782	527	693	666	655	520	599	642	524	550	595	751	629	654
SB1_2328	A_Or_SB1	5.63E+00	7.51E-01	1901	336	1749	601	1624	270	1443	563	1347	257	1145	548	1024	279	955	523	752	346	914	775	1008	542
SB1_2332	A_Or_SB1	5.46E+00	7.37E-01	1774	759	1624	1020	1509	705	1334	974	1197	683	1031	971	916	708	813	944	661	777	844	1156	877	964
SB1_2336	A_Or_SB1	5.36E+00	7.29E-01	1747	594	1583	827	1491	541	1306	800	1211	519	1031	791	907	531	798	771	636	579	812	1007	867	783
SB1_2338	A_Or_SB1	5.49E+00	7.40E-01	1646	602	1491	888	1367	564	1235	863	1094	560	938	866	812	569	731	825	535	630	744	1067	796	841
SB1_2344	A_Or_SB1	5.53E+00	7.43E-01	1924	427	1743	727	1629	378	1402	692	1283	373	1049	704	926	393	788	670	612	458	813	944	891	668
SB1_2406	A_Or_SB1	5.44E+00	7.36E-01	1894	613	1690	929	1570	560	1352	895	1223	537	1014	892	879	554	765	843	561	633	756	1137	829	872
SB1_2408	A_Or_SB1	5.40E+00	7.32E-01	1961	642	1762	985	1646	598	1421	939	1290	582	1072	925	943	591	812	884	593	648	785	1169	908	892
SB1_2412	A_Or_SB1	5.41E+00	7.33E-01	1901	433	1771	778	1605	418	1440	752	1260	390	1087	745	910	415	797	701	580	485	850	989	879	704
SB1_2414	A_Or_SB1	5.11E+00	7.08E-01	1731	620	1547	941	1424	567	1227	910	1068	557	873	897	716	583	621	870	406	658	670	1133	719	859
SB1_2416	A_Or_SB1	5.52E+00	7.42E-01	1897	477	1738	835	1608	433	1406	769	1217	417	986	784	854	421	743	720	503	496	762	1010	839	727
SB1_2418	A_Or_SB1	5.34E+00	7.28E-01	1921	553	1760	894	1607	509	1418	847	1257	482	1058	870	923	503	832	803	604	573	790	1075	905	824
SB1_2422	A_Or_SB1	5.46E+00	7.37E-01	1895	532	1700	781	1595	465	1381	747	1249	441	1031	745	920	453	798	711	596	515	776	967	898	721
SB1_2484	A_Or_SB1	5.65E+00	7.52E-01	1980	570	1798	872	1673	507	1451	821	1321	462	1075	805	961	462	798	752	621	529	810	1053	905	765
SB1_2486	A_Or_SB1	5.16E+00	7.13E-01	1744	570	1608	898	1443	550	1293	869	1135	537	939	876	788	550	681	853	478	641	694	1108	775	853

SB1_2555	A_Or_SB1	5.31E+00	7.25E-01	1942	418	1819	780	1624	371	1463	743	1289	378	1126	755	948	405	885	723	630	480	867	995	962	724
SB1_2782	A_Or_SB1	5.27E+00	7.22E-01	1970	227	1812	631	1627	190	1419	605	1258	194	988	618	812	211	680	583	440	322	719	901	806	591
SB1_2784	A_Or_SB1	5.70E+00	7.56E-01	1985	240	1801	576	1659	185	1429	531	1286	156	1036	525	909	159	749	471	542	243	769	795	854	484
SB2_2364	A_Or_SB2	5.71E+00	7.57E-01	1938	417	1719	711	1574	347	1377	671	1220	341	1004	662	861	360	725	636	511	420	762	929	849	641
SB2_2368	A_Or_SB2	5.39E+00	7.32E-01	1750	415	1587	734	1452	383	1262	723	1113	388	944	721	777	409	654	695	466	504	711	974	769	699
SB2_2370	A_Or_SB2	5.24E+00	7.19E-01	1785	460	1617	790	1484	434	1297	739	1202	426	975	755	804	459	733	748	511	523	746	1003	833	755
SB2_2372	A_Or_SB2	5.31E+00	7.25E-01	1893	481	1725	798	1600	439	1415	763	1271	433	1073	761	918	455	822	728	593	536	861	1006	899	739
SB2_2376	A_Or_SB2	5.58E+00	7.47E-01	2030	579	1806	882	1696	515	1468	827	1337	500	1118	829	996	510	861	782	658	554	846	1042	959	803
SB2_2380	A_Or_SB2	5.42E+00	7.34E-01	2001	528	1798	842	1666	465	1466	787	1323	449	1120	774	993	457	886	711	650	512	865	982	960	737
SB2_2382	A_Or_SB2	5.55E+00	7.44E-01	1951	620	1779	922	1656	555	1452	887	1311	534	1092	884	965	536	861	845	627	595	812	1111	941	855
SB2_2388	A_Or_SB2	5.46E+00	7.37E-01	1917	595	1692	913	1619	550	1400	856	1282	537	1078	852	956	542	849	803	664	587	854	1064	939	814
SB2_2396	A_Or_SB2	5.33E+00	7.27E-01	1885	604	1679	874	1606	539	1392	823	1307	537	1083	821	983	552	827	802	688	618	859	1024	954	800
SB2_2400	A_Or_SB2	5.23E+00	7.19E-01	1712	376	1571	653	1447	356	1286	649	1179	360	985	657	843	394	782	649	592	467	819	879	851	657
SBtr_2348	A_Or_SBtr	5.40E+00	7.32E-01	1812	834	1671	1106	1506	798	1357	1114	1228	798	1038	1132	902	850	817	1117	627	945	843	1380	918	1122
SBtr_2358	A_Or_SBtr	5.48E+00	7.39E-01	1885	525	1766	853	1597	484	1427	839	1276	486	1059	872	935	515	814	837	572	657	796	1109	907	853
SBtr_2360	A_Or_SBtr	5.22E+00	7.18E-01	1941	786	1766	1043	1617	723	1419	982	1276	690	1099	971	935	716	833	948	635	752	848	1201	918	979
SBtr_2458	A_Or_SBtr	5.40E+00	7.32E-01	1907	534	1729	866	1606	471	1394	824	1252	460	1020	823	938	460	801	766	556	526	754	1034	872	794
SBtr_2462	A_Or_SBtr	5.18E+00	7.14E-01	1978	541	1785	839	1682	500	1458	805	1340	476	1113	776	994	480	844	747	674	520	859	1017	941	761
SBtr_2464	A_Or_SBtr	5.67E+00	7.54E-01	1977	610	1730	932	1648	531	1402	864	1279	497	1002	858	914	510	701	800	555	537	748	1116	819	819
SBtr_2466	A_Or_SBtr	5.23E+00	7.19E-01	1998	703	1796	1030	1634	666	1440	984	1289	647	1068	967	975	642	822	930	635	724	822	1188	906	948
SBtr_2468	A_Or_SBtr	5.20E+00	7.16E-01	1912	639	1745	963	1611	586	1398	929	1274	554	1060	901	930	546	775	845	582	623	806	1112	898	852
SBtr-2760	A_Or_SBtr	5.42E+00	7.34E-01	1970	236	1753	571	1598	190	1386	542	1258	174	1057	525	904	188	783	494	596	262	799	790	881	500
SBtr_2762	A_Or_SBtr	5.30E+00	7.24E-01	1899	248	1690	615	1547	251	1348	578	1189	227	970	591	861	240	741	539	553	301	759	808	812	555
SBtr_2764	A_Or_SBtr	5.31E+00	7.25E-01	1903	314	1703	620	1585	240	1381	583	1245	220	1015	565	877	231	737	534	564	322	793	814	872	541
SBtr_2768	A_Or_SBtr	5.13E+00	7.10E-01	1959	196	1770	463	1626	140	1419	413	1270	103	1060	410	906	114	814	370	592	164	832	623	890	386
Tof3_19754	A_Or_Tof	5.36E+00	7.29E-01	911	347	822	494	789	319	676	465	642	305	531	453	497	302	421	438	345	332	424	563	468	447
Tof3_19755	A_Or_Tof	5.10E+00	7.08E-01	896	354	786	510	758	338	646	494	595	341	506	500	447	360	388	492	321	401	418	605	427	494
Tof3_19756	A_Or_Tof	5.54E+00	7.44E-01	890	254	809	405	741	234	658	386	583	230	496	383	412	242	370	372	263	286	372	503	412	379
Tof3_19764	A_Or_Tof	4.84E+00	6.85E-01	908	364	842	506	766	349	715	490	630	350	563	504	484	368	454	503	363	411	459	619	494	507
Tof3_19765	A_Or_Tof	5.23E+00	7.19E-01	823	243	746	399	669	226	599	384	538	220	450	384	392	231	332	368	250	262	348	492	376	372
Tof3_19768	A_Or_Tof	5.34E+00	7.28E-01	854	369	772	517	719	350	623	505	561	347	486	500	411	364	360	494	257	401	370	622	423	505
Tof3_19769	A_Or_Tof	5.53E+00	7.43E-01	872	167	777	325	723	154	616	315	562	156	468	316	407	176	356	314	265	220	365	449	401	312
Tof3_19770	A_Or_Tof	5.20E+00	7.16E-01	840	232	758	386	712	208	618	360	562	206	474	362	415	217	372	344	278	244	374	458	405	358
Tof4_19773	A_Or_Tof	5.64E+00	7.51E-01	882	337	811	501	737	321	654	488	588	309	500	478	420	321	359	475	270	359	383	605	422	473
Tof4_19774	A_Or_Tof	5.37E+00	7.30E-01	933	339	839	486	795	329	704	475	624	324	545	478	478	344	419	464	336	376	443	583	469	471
Tof4_19775	A_Or_Tof	5.69E+00	7.55E-01	937	397	842	551	787	381	686	540	628	387	526	553	456	404	414	549	313	446	415	667	455	543
Tof4_19776	A_Or_Tof	5.28E+00	7.23E-01	864	343	787	511	724	343	652	491	577	331	492	488	429	344	379	467	282	383	387	580	418	473
Tof4_19777	A_Or_Tof	5.56E+00	7.45E-01	916	254	838	414	769	234	681	399	601	237	529	407	448	253	420	391	296	307	421	521	452	396
Tof4_19780	A_Or_Tof	5.51E+00	7.41E-01	862	345	779	495	722	337	646	478	577	331	476	481	409	352	366	470	266	392	361	577	408	475
Tof4_19782	A_Or_Tof	5.14E+00	7.11E-01	833	209	758	381	695	200	616	361	542	198	463	367	383	223	357	359	248	271	358	474	394	361
Tof4_19785	A_Or_Tof	5.59E+00	7.47E-01	852	286	762	431	717	262	618	399	565	247	451	391	399	251	327	378	254	286	336	494	377	385
Tof4_19786	A_Or_Tof	5.77E+00	7.61E-01	900	257	816	415	761	245	667	400	604	244	505	411	440	256	392	397	292	307	387	531	427	400
Tof4_19787	A_Or_Tof	5.51E+00	7.41E-01	806	318	728	472	680	299	579	452	526	279	424	434	381	275	302	396	233	279	316	527	352	400
Tof4_19788	A_Or_Tof	5.27E+00	7.22E-01	855	278	790	435	736	261	646	407	588	245	497	398	446	243	385	367	303	262	374	486	420	373
Tof4_19790	A_Or_Tof	5.86E+00	7.68E-01	948	290	845	445	805	256	708	407	641	236	538	415	476	245	410	384	314	284	419	524	461	396
Tof4_19791	A_Or_Tof	5.38E+00	7.31E-01	830	271	751	441	690	258	599	412	531	244	450	407	374	259	318	404	237	290	333	516	372	400
Tof4_19792	A_Or_Tof	5.48E+00	7.39E-01	864	303	772	452	736	271	620	427	554	263	460	413	396	277	357	406	252	316	349	534	400	408
Ab_C_7b_2	A_Fr_Abeu	4.55E+00	6.58E-01	1193	447	1119	605	1026	417	959	588	874	410	783	584	693	421	650	565	518	469	637	714	687	588
AB_C_7b_3	A_Fr_Abeu	4.51E+00	6.54E-01	1118	457	1037	609	950	431	877	588	787	421	688	588	620	432	558	568	441	483	568	714	604	593
AB_C_7b_4	A_Fr_Abeu	4.50E+00	6.53E-01	1238	510	1150	656	1064	480	999	636	911	469	799	635	735	476	676	611	560	525	686	755	719	634
AB_C_7b_5	A_Fr_Abeu	4.99E+00	6.98E-01	1201	486	1137	678	1057	474	969	668	910	492	780	658	740	480	588	640	535	500	644	775	676	644
AB_C_7b_6	A_Fr_Abeu	4.70E+00	6.72E-01	1106	440	1055	607	949	429	882	607	784	430	702	610	606	447	548	590	435	485	566	728	600	606
AB_C_7b_7	A_Fr_Abeu	4.73E+00	6.75E-01	1152	462	1083	640	993	436	917	628	831	433	735	622	670	451	610	598	515	479	591	722	656	627
AB_C_7b_8	A_Fr_Abeu	4.84E+00	6.85E-01	1148	444	1060	626	978	425	913	607	809	424	724	599	639	429	587	581	462	484	544	719	636	610
AB_C_7b_11	A_Fr_Abeu	5.13E+00	7.10E-01	1233	473	1156	659	1058	450	954	640	876	439	747	640	685	446	600	607	479	493	612	769	634	626
AB_C_7b_14	A_Fr_Abeu	5.06E+00	7.04E-01	1187	472	1134	656	1036	462	958	659	875	472	751	669	674	477	602	642	492	526	629	795	661	660
AB_C_7b_16	A_Fr_Abeu	4.87E+00	6.88E-01	1160	408	1091	584	1015	375	914	565	839	372	734	562	674	377	589	548	488	415	604	671	640	556
AB_C_7b_17	A_Fr_Abeu	4.95E+00	6.95E-01	1237	473	1161	656	1100	454	1003	637	907	440	812	627	726	452	652	610	541	490	669	749	686	625
ABR_E1_AE93_3	A_Sp_Abr1	4.66E+00	6.68E-01	1833	761	1751	1015	1608	744	1512	1009	1396	744	1258	1009	1156	751	1068	974	892	811	1071	1179	1131	994
AB_E1_AE93_4	A_Sp_Abr1	4.70E+00	6.72E-01	1905	828	1803	1081	1692	797	1596	1070	1505	778	1333	1050	1205	787	1142	1038	967	862	1144	1225	1203	1052
ABR_E1_AE93_5	A_Sp_Abr1	4.62E+00	6.65E-01	1800	759	1717	980	1577	727	1497	977	1369	723	1232	980	1077	754	1027	955	850	819	1032	1143	1088	962
ABR_E1_AE93_8	A_Sp_Abr1	4.72E+00	6.74E-01	1774	767	1682	1016	1557	732	1435	996	1298	736	1168	1011	1065	760	966	989	784	843	964	1193	1020	1007
ABR_E1_AE93_9	A_Sp_Abr1	4.54E+00	6.57E-01	1816	729	1728	966	1572	704	1518	964	1336	713	1289	979	1130	749	1047	988	869	823	1064	1167	1108	1003
ABR_E1_AE93_10	A_Sp_Abr1	4.82E+00	6.83E-01	1970	627	1823	868	1728	571	1585	843	1467	567	1328	857	1194	602	1080	845	942	674	1133	1058	1185	852
ABR_E1_AE93_11	A_Sp_Abr1	4.63E+00	6.66E-01	1854	722	1735	960	1598	693	1530	958	1356	698	1296	962	1148	717	1051	963	875	798	1063	1145	1121	977
BelMed1	A_Bel_Med	4.57E+00	6.60E-01	1029	480	979	629	906	468	844	620	759	471	680	634	609	491	569	620	470	535	580	723	610	640
BelMed_2	A_Bel_Med	4.94E+00	6.94E-01	1119	472	1056	639	988	457	909	629	851	453	751	630	688	470	634	611	520	524	635	743	676	629
BelMed_3	A_Bel_Med	4.86E+00	6.87E-01	1127	471	1049	609	995	438	897	599	846	426	736	599	674	439	619	556	522	482	593	695	647	575
BelMed_4	A_Bel_Med	4.53E+00	6.56E-01	1063	504	985	652	928	479	857	641	777	483	701	643	624	496	572	625	467	551	579	741	615	640
BelMed_6	A_Bel_Med	4.98E+00	6.97E-01	1061	472	1001	634	937	458	858	623	792	444	690	624	640	443	554	587	479	488	547	713	603	591
BelMed_7	A_Bel_Med	4.49E+00	6.52E-01	1115	493	1056	640	985	485	927	645	841	494	758	662	704	515	667	645	564	557	652	764	689	664
BelMed_8	A_Bel_Med	4.63E+00	6.66E-01	1117	497	1050	645	992	471	909	621	845	460	759	612	689	475	631	597	537	507	641	720	680	610
BelMed_9	A_Bel_Med	4.84E+00	6.85E-01	1096	471	1018	617	964	448	873	604	816	441	715	599	667	452	609	571	506	485	593	666	654	600
MG_1_4	A_Fr_MG	4.77E+00	6.79E-01	1139	455	1054	644	972	443	895	622	803	439	701	633	642	437	576	603	461	482	588	747	619	618
MG_1_5	A_Fr_MG	4.92E+00	6.92E-01	1214	450	1124	646	1048	443	965	623	852	453	759	629	662	461	598	617	500	496	614	762	643	632
MG_2_3	A_Fr_MG	4.61E+00	6.64E-01	1105	390	1029	565	940	378	858	549	777	369	687	555	602	378	557	541	406	427	565	669	593	558
MG_2_4	A_Fr_MG	4.29E+00	6.32E-01	1119	402	1049	573	983	387	908	554	839	381	748	542	675	389	637	546	516	430	613	674	697	555
MG_4_1	A_Fr_MG	4.38E+00	6.41E-01	1180	450	1130	635	1025	436	975	622	887	446	816	618	723	447	688	609	560	496	698	750	726	628
MG_5_1	A_Fr_MG	4.75E+00	6.77E-01	1268	445	1192	613	1124	423	1033	580	918	412	827	568	725	414	680	559	561	463	685	686	741	576
MG_5_6	A_Fr_MG	4.66E+00	6.68E-01	1192	496	1126	663	1060	475	968	633	884	466	792	640	706	474	642	611	522	519	652	757	698	626

x12	y12	x13	y13	x14	y14	x15	y15	x16	y16	x17	y17	x18	y18	x19	y19	x20	y20	x21	y21	x22	y22	x23	y23	x24	y24
1137	1473	1284	1185	1444	1464	1580	1222	1760	1498	579	1074	518	1084	456	1088	398	1109	360	1158	347	1219	357	1280	397	1328
1220	1334	1375	1025	1551	1311	1695	1054	1870	1338	699	971	636	969	573	965	512	972	467	1013	448	1073	449	1135	487	1185
1259	1347	1366	1014	1599	1347	1695	1029	1966	1331	591	1020	553	957	513	896	443	887	380	923	351	988	360	1061	393	1127
1368	1340	1457	1033	1711	1353	1810	1051	2033	1369	731	978	668	952	611	912	544	903	497	953	475	1017	490	1084	529	1141
1340	1331	1433	1007	1670	1322	1768	1034	1980	1369	688	980	612	963	538	947	467	973	414	1028	398	1102	432	1170	494	1218
1335	1451	1442	1113	1666	1424	1768	1105	1986	1449	724	1118	650	1100	578	1086	506	1108	451	1156	427	1226	444	1296	498	1347
1266	1384	1293	1082	1568	1360	1619	1085	1826	1351	604	1033	546	1021	492	995	432	988	403	1037	399	1098	406	1157	439	1207
1324	1309	1482	976	1690	1320	1811	996	2059	1333	684	965	617	934	549	902	478	918	425	967	409	1038	428	1106	477	1159
1353	1300	1451	1005	1684	1289	1759	1000	1991	1271	800	969	738	949	679	918	614	911	560	949	525	1005	512	1070	540	1129
1348	1349	1450	1027	1690	1349	1793	1045	2022	1396	720	964	651	956	583	942	513	954	463	1000	437	1066	448	1134	487	1192
1130	1464	1277	1144	1491	1438	1626	1158	1851	1445	551	1091	486	1091	421	1090	358	1099	310	1137	283	1195	293	1257	332	1308
1246	1345	1364	1022	1537	1331	1691	1049	1822	1340	662	996	598	993	536	975	475	993	426	1036	405	1097	421	1156	467	1201
1179	1244	1339	904	1537	1236	1713	905	1837	1238	655	902	601	895	550	879	499	898	459	936	437	987	437	1043	466	1088
1200	1384	1339	1045	1533	1364	1671	1049	1850	1360	642	1007	582	980	521	959	459	973	415	1020	394	1082	405	1145	440	1200
1277	1436	1422	1080	1631	1424	1777	1094	1970	1431	673	1013	600	983	531	947	466	977	426	1044	394	1117	394	1195	445	1255
1239	1327	1326	1058	1566	1294	1624	1065	1815	1327	750	1122	699	1080	656	1026	605	983	539	971	484	1006	463	1069	466	1137
1268	1416	1368	1105	1631	1391	1700	1093	1915	1398	655	1040	590	1023	528	996	463	1012	416	1059	402	1124	421	1189	461	1243
1235	1385	1335	1078	1542	1380	1651	1100	1840	1398	631	1056	574	1054	517	1059	474	1094	452	1148	459	1206	491	1253	541	1283
1273	1325	1408	998	1622	1318	1748	998	1959	1293	706	951	637	919	582	865	505	878	447	929	407	994	404	1069	448	1131
1175	1354	1273	1018	1493	1358	1577	1038	1810	1371	597	1036	526	1014	459	977	382	963	327	1008	320	1083	341	1158	383	1219
1259	1413	1384	1085	1630	1407	1726	1089	1995	1382	662	996	597	961	538	916	462	915	401	959	371	1030	374	1106	420	1169
1330	1293	1419	1024	1624	1284	1730	1027	1935	1293	739	1029	670	1009	613	963	552	937	503	991	480	1060	490	1131	538	1185
1420	1334	1526	1022	1733	1338	1853	1069	2040	1365	800	944	734	928	669	917	607	943	562	991	542	1054	560	1117	612	1161
1235	1444	1333	1102	1544	1438	1680	1116	1866	1433	602	1122	545	1093	491	1060	429	1069	385	1115	382	1180	401	1242	441	1293
1288	1425	1411	1093	1644	1407	1759	1111	1982	1414	619	1051	552	1023	486	995	418	1013	371	1069	352	1139	368	1209	417	1263
1295	1360	1415	1029	1660	1349	1775	1056	2019	1362	691	1002	625	976	563	946	497	967	446	1016	426	1084	427	1154	470	1207
1279	1334	1397	1033	1600	1334	1728	1056	1915	1342	688	1045	632	1023	577	998	518	1003	485	1053	480	1114	502	1170	541	1216
1326	1342	1406	1038	1630	1362	1753	1089	1960	1394	688	1018	640	1040	587	1041	544	1060	527	1109	525	1161	548	1208	587	1243
1233	1342	1348	1029	1557	1358	1690	1067	1831	1396	622	1002	558	992	496	976	435	991	392	1038	378	1099	396	1161	429	1214
1206	1364	1317	1065	1522	1342	1650	1056	1837	1329	602	985	539	997	475	990	414	1009	378	1060	363	1121	376	1181	415	1233
1237	1234	1390	964	1602	1264	1706	974	1860	1244	713	884	661	858	604	848	547	850	500	881	475	932	469	990	493	1042
1293	1274	1451	994	1700	1276	1819	1000	2010	1274	679	1024	617	989	558	947	488	944	435	991	400	1054	397	1126	435	1187
1124	1196	1268	911	1455	1187	1637	914	1770	1196	597	862	535	861	474	853	414	857	365	893	336	947	336	1007	373	1056
1273	1294	1360	994	1577	1280	1680	1011	1871	1311	690	1031	624	1011	573	961	507	937	447	970	434	1038	445	1108	481	1168
1146	1240	1320	951	1530	1225	1744	978	1828	1233	582	933	515	920	450	902	383	912	340	963	318	1028	332	1094	379	1141
1108	1229	1251	938	1440	1225	1533	944	1742	1224	573	885	528	904	479	908	432	923	414	966	407	1014	419	1062	452	1098
1308	1287	1419	973	1633	1293	1775	987	1940	1293	784	916	719	900	655	886	590	901	539	946	513	1005	535	1069	576	1122
1128	1360	1253	1053	1519	1358	1611	1058	1826	1358	504	1020	444	1031	382	1026	324	1045	292	1094	284	1155	307	1209	350	1252
1150	1318	1280	996	1491	1282	1640	1000	1850	1282	553	889	514	912	482	943	448	973	429	1013	413	1056	413	1101	427	1142
1219	1374	1311	1038	1553	1342	1693	1036	1897	1351	520	1100	448	1076	378	1051	308	1076	265	1139	245	1213	276	1281	337	1328
1217	1373	1351	1060	1537	1360	1671	1054	1877	1362	655	1089	592	1067	533	1033	469	1016	412	1045	389	1107	385	1175	406	1239
1230	1347	1295	1049	1546	1331	1626	1045	1830	1331	624	1065	572	1086	516	1093	462	1110	421	1147	409	1202	418	1257	454	1299
1337	1247	1475	991	1642	1251	1780	1004	1922	1265	795	976	725	963	659	933	588	923	542	973	526	1042	570	1094	593	1155
1191	1334	1340	998	1582	1347	1730	1036	1930	1344	513	916	458	941	402	962	373	1013	358	1070	354	1130	381	1183	416	1231
1199	1418	1339	1120	1506	1414	1633	1136	1813	1418	622	1085	576	1105	524	1116	479	1138	463	1186	458	1238	472	1285	512	1317
1255	1358	1377	1069	1577	1351	1722	1082	1908	1373	711	1020	654	1019	599	1002	542	998	498	1028	490	1085	490	1142	512	1192
1224	1365	1362	1091	1562	1349	1684	1107	1875	1373	675	989	613	987	551	989	497	1018	461	1069	448	1130	466	1188	505	1236
1235	1364	1320	1104	1542	1340	1622	1104	1848	1322	662	1096	604	1074	564	1025	507	1003	457	1033	422	1086	410	1149	426	1209
1304	1387	1470	1049	1679	1373	1830	1080	2015	1394	684	971	613	975	542	962	478	985	436	1044	415	1111	430	1180	477	1234
1155	1376	1355	1047	1535	1374	1715	1056	1882	1398	577	991	516	1011	452	1019	392	1041	352	1088	327	1147	330	1210	368	1259
1144	1267	1313	994	1479	1271	1617	1002	1804	1280	648	960	586	946	524	937	461	939	410	977	384	1035	381	1096	413	1151
1110	1420	1246	1087	1453	1393	1560	1084	1782	1382	573	1053	516	1049	458	1043	401	1051	361	1088	342	1142	343	1200	371	1250
1197	1344	1291	1053	1560	1333	1655	1054	1893	1344	619	1011	557	1016	495	1018	435	1032	389	1071	385	1132	399	1193	432	1244
1208	1320	1304	1033	1539	1344	1691	1051	1882	1353	628	1004	563	995	501	980	440	1001	388	1042	356	1098	348	1163	377	1218
1397	1374	1491	1054	1764	1371	1877	1053	2100	1353	799	1065	730	1042	665	1010	591	1010	529	1045	500	1112	506	1182	553	1239
1246	1356	1371	1040	1600	1362	1717	1054	1931	1360	624	1027	566	1038	507	1040	451	1056	413	1099	407	1156	430	1210	463	1259
1260	1354	1331	1022	1626	1340	1693	1024	1966	1322	610	976	539	985	468	984	397	993	354	1046	337	1115	346	1184	392	1239
1275	1251	1382	914	1648	1254	1779	922	2031	1245	548	914	472	896	396	874	322	896	260	945	245	1020	280	1090	342	1141
1228	1225	1373	922	1591	1229	1750	934	1942	1227	646	871	581	867	518	847	454	859	400	894	378	958	371	1024	404	1082
1406	1380	1486	1076	1730	1374	1828	1087	2064	1374	784	1069	713	1044	653	991	582	985	527	1039	499	1112	492	1191	511	1268
1370	1344	1420	1038	1697	1333	1828	1076	2022	1344	666	1067	619	1022	554	1011	496	1040	463	1095	474	1158	517	1208	573	1241
1237	1333	1362	1044	1599	1336	1708	1047	1964	1342	633	1020	571	1014	510	1000	450	1005	405	1042	380	1098	404	1154	454	1191
1304	1429	1413	1105	1662	1414	1771	1107	2013	1431	693	1065	623	1051	554	1039	488	1051	440	1099	416	1164	435	1229	484	1279
1151	1287	1255	1016	1479	1298	1580	1033	1768	1284	577	945	534	958	485	964	451	997	437	1045	440	1094	465	1134	506	1162
1242	1314	1315	1022	1590	1314	1671	1038	1926	1331	573	974	514	958	456	938	396	941	359	985	351	1045	377	1098	425	1137
1253	1322	1366	1040	1571	1333	1668	1069	1851	1333	708	991	659	990	610	985	565	992	544	1036	536	1084	559	1126	590	1163
1204	1302	1302	993	1533	1291	1651	1009	1848	1294	602	991	544	983	487	974	433	987	399	1033	393	1091	412	1146	460	1179
1273	1218	1379	991	1568	1242	1695	1027	1875	1260	755	976	707	970	663	949	616	946	589	980	593	1026	612	1071	640	1110
328	274	352	193	405	273	432	191	480	269	180	181	165	171	147	167	129	171	118	185	115	202	121	219	132	232
264	335	297	252	354	331	375	257	428	333	114	242	97	234	81	227	63	232	49	243	42	260	43	279	55	293
245	321	274	240	329	317	354	237	411	310	99	246	84	242	69	241	54	241	46	253	47	268	54	281	65	292
262	339	287	259	339	341	376	254	417	333	121	250	103	243	85	240	67	246	54	258	50	275	56	292	67	306
282	300	317	231	360	305	394	237	432	301	150	227	134	220	117	216	99	213	88	227	85	243	89	260	101	273
280	310	305	237	350	311	377	239	419	313	144	239	129	225	114	212	96	211	87	227	79	245	73	263	80	281
269	319	297	244	348	317	376	247	427	314	122	236	105	234	89	229	74	236	62	248	62	265	67	280	79	291
286	334	320	253	369	339	408	261	446	341	143	252	126	236	114	216	94	210	80	229	62	243	55	265	61	287
302	319	323	244	391	323	413	244	464	320	142	250	128	236	116	222	97	224	80	234	70	251	75	271	88	285
275	314	311	251	353	319	391	250	432	318	151	242	138	244	125	244	112	244	102	251	101	264	104	277	112	287
266	315	305	242	350	315	390	239	427	324	115	252	101	245	86	240	71	240	58	248	55	263	60	278	71	289

279	307	322	224	381	311	412	233	461	303	125	217	109	215	93	214	78	218	67	229	65	245	72	258	84	269
257	310	288	230	336	303	372	233	408	314	115	216	101	216	86	216	72	217	61	225	59	238	62	252	70	263
315	301	356	231	413	303	450	225	491	304	169	228	154	220	140	212	124	214	111	223	107	239	110	255	119	268
325	297	350	216	410	297	445	213	488	294	166	214	149	204	133	196	115	198	101	210	96	228	100	247	113	260
254	328	281	261	330	324	356	261	400	333	113	258	98	252	82	248	67	251	55	260	49	275	52	291	63	302
286	322	323	239	377	325	419	244	465	324	143	222	124	213	103	207	83	205	63	211	53	230	58	251	72	267
294	294	325	210	381	292	413	218	461	287	129	208	117	194	101	186	85	188	73	200	69	217	72	234	84	248
289	312	333	228	377	312	425	223	470	311	139	227	122	217	107	202	87	203	72	215	61	233	61	253	74	269
319	286	358	201	413	287	444	208	486	286	171	199	155	198	139	199	125	204	116	215	113	231	122	244	132	256
359	375	386	283	455	372	482	283	538	372	182	294	160	291	139	286	117	288	102	303	100	324	107	344	124	358
311	294	337	216	388	290	424	214	468	296	173	205	155	196	136	190	116	187	101	199	94	217	96	237	105	255
325	308	359	233	406	313	446	235	481	319	180	228	161	220	142	213	122	214	106	225	97	243	105	261	122	273
280	340	306	255	369	340	394	258	442	335	125	244	107	241	90	238	73	239	62	253	59	271	63	287	75	300
291	310	329	241	371	311	411	241	448	314	152	237	141	224	127	214	111	211	98	222	91	238	92	255	99	270
272	300	298	222	355	298	378	223	425	299	137	213	123	208	111	201	98	200	91	212	92	226	96	240	106	251
286	333	316	255	373	337	408	264	461	339	133	257	114	246	94	237	73	238	60	255	56	276	66	296	83	310
269	342	296	261	351	341	378	259	431	332	120	278	108	268	94	262	78	261	67	269	65	284	72	297	84	307
293	286	312	222	367	289	401	222	436	288	162	219	151	214	139	209	127	209	116	212	110	223	111	235	115	245
271	307	298	232	347	313	375	231	418	310	126	231	112	222	98	214	82	214	72	225	72	241	75	256	85	269
469	492	506	390	581	484	613	377	683	480	278	415	261	398	243	382	219	379	201	392	194	416	193	441	199	464
401	478	458	390	516	478	564	395	629	478	209	384	188	373	167	366	145	371	129	386	123	408	129	431	144	447
486	559	529	453	608	551	653	450	725	550	275	467	253	464	231	456	208	458	194	475	191	498	199	520	219	533
489	499	528	394	597	499	645	388	709	497	289	394	268	391	250	379	229	382	214	397	210	418	214	440	224	458
457	503	499	416	557	500	605	415	668	507	263	409	242	407	221	401	202	408	191	426	189	446	200	464	215	479
452	505	484	397	564	502	610	405	674	497	225	393	201	380	177	373	153	384	139	406	137	432	149	455	169	471
425	574	456	467	532	570	579	469	631	570	199	446	172	446	145	451	124	467	113	491	115	518	126	541	153	547
413	514	467	401	527	508	580	404	640	508	223	408	206	401	188	396	173	407	165	424	163	443	172	460	184	475
482	518	508	424	583	510	611	418	682	513	314	443	296	432	274	428	254	433	237	445	230	464	234	485	247	503
454	515	492	415	556	503	596	410	662	509	274	414	255	405	234	402	214	407	197	420	193	440	199	460	212	477
407	446	447	340	513	442	565	347	611	440	224	314	201	307	178	301	157	310	141	327	133	349	136	372	151	391
486	495	526	400	595	495	631	394	696	488	289	414	266	401	245	386	222	394	205	413	195	437	196	462	213	481
438	486	480	396	542	489	581	406	634	491	267	384	246	380	226	374	207	376	194	392	190	413	192	434	202	451
409	533	458	431	521	530	571	443	633	534	230	414	209	400	183	396	159	402	141	420	134	444	138	469	150	491
446	522	494	420	564	516	603	414	670	515	247	427	227	425	207	426	191	438	185	456	186	476	197	492	213	504
477	471	531	375	593	472	641	379	703	478	291	355	269	343	246	337	223	339	210	360	205	383	206	407	219	426
434	473	478	367	538	470	586	373	645	470	249	381	228	378	209	372	189	375	174	389	173	409	178	429	192	444
444	540	486	433	564	540	601	435	674	540	235	433	212	422	187	414	164	422	151	444	142	469	143	495	157	514
463	346	499	246	577	349	608	250	681	343	266	236	247	229	227	225	208	230	193	244	188	263	193	282	207	297
452	464	504	351	574	460	640	358	685	459	255	347	237	352	218	353	201	361	190	376	188	394	194	412	208	424
495	551	534	448	603	543	636	446	698	549	306	447	284	439	261	435	238	437	220	450	213	472	222	493	238	509
324	429	363	320	448	422	482	314	551	423	122	339	102	339	82	339	66	349	55	365	52	384	59	401	75	413
318	504	352	410	419	502	467	406	524	503	130	405	109	395	88	385	66	389	52	407	50	429	60	450	74	469
454	590	497	491	570	586	607	497	674	585	268	505	245	500	223	495	201	502	191	521	189	544	195	566	210	584
464	495	500	399	573	493	618	400	687	490	254	392	230	387	206	385	185	394	168	410	163	433	173	454	191	471
338	518	367	423	440	515	482	425	538	511	142	435	122	430	104	422	84	421	73	437	74	457	78	476	88	493
312	495	355	407	409	498	462	406	506	497	140	379	117	373	95	371	73	380	61	400	55	422	62	443	79	459
595	1041	692	830	825	1028	914	830	1025	1027	254	849	216	843	182	826	149	831	126	860	118	896	127	931	149	963
681	1089	758	869	901	1059	960	863	1107	1039	295	895	259	892	224	880	196	900	188	936	187	973	203	1007	226	1035
726	1050	795	848	953	1023	1018	828	1155	991	290	881	244	861	202	837	152	837	120	872	111	921	123	969	162	1001
619	1051	717	865	853	1044	937	858	1070	1034	240	885	201	877	161	871	124	874	99	903	97	942	114	977	144	1004
685	1080	745	888	889	1057	956	874	1087	1039	298	890	255	875	212	865	169	878	132	905	116	947	126	990	159	1020
724	1021	788	819	935	988	1001	798	1121	957	341	821	299	809	257	797	219	814	201	852	199	894	219	933	251	962
649	1078	727	865	868	1046	944	839	1061	1014	254	885	210	877	171	852	133	842	108	875	99	919	118	960	142	997
667	1069	756	871	905	1044	993	860	1128	1030	279	892	240	901	201	901	162	906	136	933	126	971	135	1010	157	1041
884	883	965	730	1084	888	1167	743	1275	890	516	672	480	666	444	656	409	662	387	691	372	723	377	760	403	784
561	1168	651	934	775	1158	871	924	999	1137	176	941	140	949	102	949	72	965	61	1000	61	1037	76	1071	101	1098
704	1046	781	844	931	1035	1018	844	1134	1032	263	869	220	851	176	833	131	851	102	889	101	936	127	978	170	1001
735	1126	820	908	956	1113	1040	917	1144	1115	320	906	280	869	239	840	190	862	159	905	142	955	157	1006	196	1044
729	1034	821	833	960	1023	1052	832	1189	1004	346	851	307	832	271	807	226	805	192	835	177	878	180	922	203	961
768	1076	807	876	972	1053	1050	863	1169	1028	291	894	244	889	198	883	158	907	141	951	141	998	160	1039	196	1070
657	1083	759	869	875	1076	974	876	1079	1064	304	869	258	867	212	865	166	872	131	899	117	943	127	987	159	1020
609	784	699	590	832	780	917	594	1052	778	204	598	166	594	128	594	97	611	87	647	91	685	108	718	134	745
623	1188	697	982	841	1161	953	1002	1045	1156	231	1002	192	1005	152	1000	115	1009	95	1041	97	1079	112	1115	142	1141
758	1083	866	885	1004	1082	1089	894	1219	1087	362	867	323	858	282	855	245	866	222	896	218	935	234	971	261	999
701	1035	814	844	938	1030	1036	848	1153	1018	291	833	252	825	213	813	173	816	145	841	144	880	159	919	188	948
706	832	772	613	928	800	1036	605	1150	780	307	610	262	595	215	584	166	592	126	620	114	668	123	715	154	753
669	840	793	654	889	848	1001	670	1105	863	329	622	290	608	250	598	209	595	186	624	176	664	185	703	203	739
743	863	811	647	951	837	1024	637	1139	826	360	670	319	671	278	667	239	678	221	713	229	752	249	788	279	816
625	1059	727	856	859	1034	956	835	1086	1014	238	865	194	855	149	854	108	871	81	908	69	949	77	992	100	1030
703	810	781	610	923	800	1009	619	1164	789	291	596	248	589	206	579	166	586	134	614	121	654	137	694	170	720
319	432	352	338	422	435	457	341	529	441	137	312	116	303	95	297	72	296	56	311	50	333	52	356	64	375
392	527	422	423	505	507	535	410	611	493	193	456	167	453	141	450	116	458	102	480	99	506	108	529	132	540
346	566	376	465	459	556	489	451	554	552	153	468	131	459	109	454	87	459	77	479	78	502	86	524	102	540
348	427	369	328	443	425	469	322	532	419	150	327	131	313	115	295	93	288	74	299	67	321	70	344	82	363
367	551	398	444	474	543	503	436	576	530	168	468	148	452	128	432	104	425	82	441	66	464	60	491	75	514
381	393	418	285	492	385	528	282	612	375	186	293	163	276	141	257	114	252	91	268	78	293	78	321	93	345
359	390	396	291	461	377	500	292	560	376	179	285	159	270	136	259	112	256	89	267	82	291	86	315	96	338
318	523	353	433	427	511	456	423	535	503	128	430	105	429	83	428	62	433	47	450	45	472	54	492	69	508
346	532	387	430	460	524	500	431	566	527	157	433	132	422	108	409	82	415	61	433	51	458	55	485	72	507
317	414	354	307	433	401	468	293	546	391	128	327	106	324	86	313	62	312	47	328	42	351	48	372	64	389
310	543	344	454	421	532	447	440	528	531	121	459	99	461	76	462	59	475	48	495	45	517	57	536	75	548
346	394	387	292	460	391	496	286	563	381	163	280	142	280	121	276	102	285	90	302	86	322	93	342	107	357
337	420	383	328	453	422	492	331	555	425	159	315	135	309	116	293	92	294	73	309	65	332	67	356	79	376

351	515	377	418	450	505	481	408	546	497	158	428	137	416	120	398	97	403	83	424	74	447	73	472	86	492
376	528	415	439	478	528	507	431	579	518	193	422	170	424	147	421	125	425	111	443	105	465	108	488	123	506
330	514	357	425	430	512	451	417	524	506	140	435	122	424	105	413	86	417	78	437	75	458	83	478	100	491
351	561	397	453	476	562	507	454	582	556	124	446	100	440	76	441	58	457	48	478	53	502	71	520	90	534
355	386	390	279	462	382	487	278	560	378	177	293	153	288	133	270	109	264	88	277	76	300	78	326	91	347
400	434	443	325	527	426	565	325	631	417	171	324	146	321	121	317	100	328	86	348	80	372	87	396	108	411
338	278	364	189	433	271	457	193	520	264	171	179	155	179	139	181	127	190	121	205	121	220	128	234	140	244
292	305	319	225	383	299	414	225	469	304	133	212	119	211	106	213	94	218	86	229	84	243	92	254	101	264
338	285	363	202	425	279	451	204	508	280	186	190	168	192	151	187	133	186	121	198	118	215	121	232	131	247
305	250	334	172	394	249	425	171	477	247	162	153	146	151	131	151	115	154	104	164	99	178	103	193	112	205
321	262	349	175	408	261	441	186	494	268	179	160	165	156	150	154	136	158	127	170	127	185	131	199	138	212
280	294	306	212	366	298	384	219	442	296	127	195	112	194	98	198	86	206	78	218	77	233	82	246	91	258
297	318	334	236	388	319	423	241	477	325	144	214	126	216	108	217	91	219	76	228	73	244	79	260	91	273
300	322	332	247	396	318	419	246	480	316	144	242	127	240	111	237	95	240	86	255	80	270	82	287	93	299
304	249	348	181	388	255	423	190	465	258	171	164	161	167	151	170	143	176	140	186	140	197	144	206	150	215
276	305	311	232	361	307	392	235	444	302	128	221	111	217	94	212	77	213	65	224	61	240	64	257	75	270
305	312	332	233	394	306	423	234	477	307	141	228	122	226	104	223	86	224	73	237	71	255	78	272	91	284
327	250	361	175	413	247	445	180	502	250	175	161	163	168	148	166	135	171	129	184	128	198	131	212	140	224
347	354	386	282	438	349	456	277	517	348	204	277	190	274	175	273	161	275	152	286	150	300	153	314	163	324
320	275	342	203	395	275	422	206	472	277	179	183	167	188	155	187	143	190	136	199	135	211	136	224	144	233
318	279	351	204	408	281	438	211	500	289	169	203	153	202	137	197	121	199	108	208	105	224	109	240	120	252
337	236	380	170	422	239	461	175	502	244	207	156	194	151	184	142	174	139	164	148	161	160	164	174	169	186
290	318	333	241	392	317	419	240	461	319	147	225	130	218	113	212	95	214	80	225	74	242	76	260	85	276
349	545	373	435	462	536	487	430	573	520	133	443	109	442	85	440	66	452	58	473	63	496	78	515	99	525
359	518	399	418	467	506	498	405	577	507	164	407	141	408	118	403	97	410	88	431	88	454	98	475	114	491
379	600	412	499	486	591	522	489	587	591	189	486	170	485	151	485	135	492	127	509	125	528	132	545	144	559
365	471	395	362	487	463	519	361	586	454	162	351	143	357	123	357	104	363	94	380	91	400	98	419	115	431
372	510	394	392	487	504	514	390	589	497	154	381	131	376	109	370	88	376	78	397	75	420	79	443	93	462
345	527	382	419	459	523	489	419	562	521	132	409	109	411	87	412	66	417	58	438	57	460	67	480	84	495
424	440	443	338	524	444	552	337	627	435	209	340	189	330	168	324	147	323	137	341	134	362	143	382	158	397
353	560	395	447	465	547	500	441	563	543	145	462	122	456	99	454	77	461	66	481	65	504	75	525	92	539
351	556	382	456	459	557	495	458	572	551	154	437	133	439	112	439	94	447	81	463	78	484	84	503	96	519
414	566	439	462	520	558	553	462	619	565	199	467	179	454	158	442	135	445	122	463	120	487	129	509	145	526
419	438	456	332	533	437	574	336	638	433	184	331	159	315	137	296	110	292	89	311	81	339	86	369	102	394
369	410	391	301	467	401	492	297	557	398	177	306	155	305	134	301	112	303	103	321	101	342	110	361	124	378
352	393	385	324	442	396	472	321	525	398	203	297	188	291	172	290	156	292	144	301	141	317	144	333	154	346
361	379	390	299	448	375	476	299	534	364	200	284	184	278	167	278	152	286	144	300	143	317	146	334	157	347
345	382	380	298	432	383	466	303	515	383	197	296	181	290	166	283	150	288	140	301	137	318	142	334	153	347
330	386	361	303	419	385	450	306	498	384	185	285	167	285	150	286	136	296	125	309	119	326	124	343	138	354
323	374	358	297	410	379	455	308	486	379	188	277	172	278	157	276	142	279	131	291	126	306	127	322	135	335
341	382	370	305	426	374	454	306	500	375	190	287	174	284	157	283	142	289	132	301	133	317	139	332	150	344
352	354	379	274	442	350	463	274	528	340	194	260	179	256	164	254	150	255	140	266	139	281	141	296	151	307
347	368	380	290	440	370	468	292	522	368	193	279	177	278	161	276	147	282	135	292	130	307	134	323	143	336
341	363	370	278	423	354	451	274	503	346	191	266	173	262	156	261	139	265	127	277	124	294	130	311	142	324
367	377	394	301	454	377	476	301	532	379	210	287	194	283	177	280	161	282	150	295	147	311	152	326	163	339
336	392	380	309	441	395	473	315	529	394	179	293	161	285	142	283	124	292	114	308	111	327	116	346	129	360
341	378	372	303	424	380	454	305	504	380	193	292	178	287	163	287	150	294	143	308	144	323	151	336	161	348
352	373	394	295	442	376	473	299	521	378	208	271	196	276	182	277	172	283	167	295	167	308	174	319	183	329
265	364	296	284	350	364	380	287	425	368	122	275	109	260	92	253	75	258	63	272	59	290	65	309	78	323
278	326	316	262	361	333	388	255	438	336	137	228	122	222	107	222	92	228	84	240	82	256	85	271	94	284
277	328	308	250	363	328	391	253	441	327	127	244	110	244	94	242	78	246	68	258	67	274	75	289	88	298
280	339	311	269	369	342	396	269	446	343	139	245	124	239	111	231	97	233	87	245	81	259	82	275	90	289
311	325	342	254	398	323	426	254	479	319	158	239	141	238	124	239	108	245	99	258	97	275	102	290	115	302
277	317	308	250	366	319	385	253	438	316	132	230	118	222	103	216	90	222	82	236	79	252	83	268	91	282
291	318	314	244	374	315	399	245	448	318	141	228	123	224	105	223	87	228	74	240	69	258	71	276	83	289
292	325	323	252	380	325	397	254	455	325	158	240	141	235	125	229	107	230	93	240	86	256	86	274	96	288
283	321	309	247	363	328	394	254	434	329	145	234	132	224	115	221	99	228	86	237	82	254	86	270	97	283
316	305	347	233	398	310	435	236	480	312	163	209	146	202	129	197	113	204	105	220	105	238	111	255	123	268
321	317	347	243	404	318	438	252	484	316	170	235	156	227	140	224	124	228	114	241	109	256	112	272	122	285
280	336	308	263	363	342	391	264	438	336	133	247	116	237	98	232	80	239	66	252	60	269	65	287	78	302
267	299	297	222	348	292	372	222	413	293	130	217	115	210	99	205	85	212	77	226	75	242	77	259	86	273
308	287	337	213	394	286	419	219	466	291	159	210	144	198	130	188	112	191	99	204	94	221	97	239	110	252
261	315	288	239	344	317	370	250	422	317	127	228	112	225	97	223	82	225	71	235	67	250	67	265	74	279
305	314	330	244	380	314	412	253	449	318	156	240	140	230	123	223	107	231	94	244	91	262	97	280	111	293
289	313	321	241	380	309	405	240	458	311	147	223	132	218	118	213	102	214	91	224	90	240	95	255	106	267
316	316	341	244	395	315	418	250	466	322	179	226	162	224	146	219	129	223	118	235	113	251	117	268	128	280
307	318	343	241	399	314	429	247	475	319	161	233	145	232	129	227	113	226	104	238	100	254	105	270	116	282
297	338	324	262	388	336	411	264	471	333	141	249	124	247	106	245	89	248	81	263	80	280	87	295	99	307
284	322	311	248	364	318	387	248	441	319	143	239	127	235	112	232	97	238	86	248	84	264	88	280	99	292
294	315	325	238	378	319	415	244	460	322	151	230	135	224	117	221	100	223	87	234	83	250	87	266	98	279
291	328	323	255	377	328	411	258	455	329	141	247	123	246	105	244	88	247	77	261	76	278	81	295	93	307
263	309	296	244	346	314	380	244	424	317	123	245	107	240	93	230	77	233	67	246	64	262	69	277	79	290
271	340	300	267	355	342	377	272	425	342	122	256	107	251	91	249	76	252	67	265	66	280	73	295	83	307
285	302	312	228	375	296	398	230	452	294	147	225	131	221	116	216	100	217	90	229	87	244	90	260	99	271
287	300	319	226	379	301	404	230	460	295	141	228	126	224	114	215	99	219	90	231	88	245	92	260	100	272
280	320	313	253	367	323	396	258	441	327	138	243	123	242	107	242	93	246	85	257	83	272	88	286	99	296
280	323	307	249	361	324	386	257	433	328	136	233	121	228	105	226	90	229	80	240	77	256	80	271	89	283
293	311	330	243	380	317	410	253	454	325	154	230	137	226	121	222	105	228	95	240	92	257	96	273	107	286
320	364	372	279	416	360	459	279	502	354	191	273	179	262	163	257	147	260	135	272	134	288	135	305	140	320
291	305	325	221	380	298	411	222	461	296	141	217	126	207	110	201	94	206	83	220	82	237	89	253	101	266
364	383	385	296	441	380	472	299	523	376	197	284	177	279	157	274	139	281	125	296	120	316	125	335	140	348
325	361	342	287	405	360	436	290	488	360	169	285	156	284	143	283	130	282	123	293	122	306	126	318	134	328
356	332	378	251	444	328	468	254	523	329	198	259	182	251	166	243	148	244	137	256	135	274	142	290	156	302

352	346	381	265	438	343	470	268	514	344	191	257	175	253	158	252	141	252	131	265	130	281	136	296	147	308
330	293	357	208	413	283	454	208	488	290	178	199	161	194	143	191	126	198	114	211	109	228	117	244	130	257
339	267	361	180	423	258	452	178	500	255	185	175	170	169	154	166	139	169	131	182	130	198	133	213	145	224
363	354	383	264	448	353	475	270	527	355	192	255	174	253	155	253	138	258	127	272	129	289	138	305	151	319
347	331	394	255	441	332	483	262	523	332	213	240	196	235	180	230	164	235	151	246	146	262	149	279	159	293
330	275	361	199	419	274	451	199	502	273	171	192	155	186	138	184	123	192	114	206	113	223	117	239	127	253
266	331	315	247	355	328	400	252	438	331	124	239	107	231	91	221	72	221	56	233	47	250	49	269	59	286
369	346	402	263	456	343	494	268	536	346	212	274	198	261	182	253	163	255	150	268	144	285	148	302	161	316
318	380	348	296	411	379	444	300	497	374	169	291	153	290	137	285	121	289	108	299	102	315	105	332	114	345
355	373	379	290	443	370	470	292	517	365	201	291	184	289	167	285	150	289	137	299	133	315	139	331	152	344
282	318	322	237	377	318	412	231	461	309	134	208	115	204	96	203	77	205	63	218	55	235	58	254	67	270
311	296	343	205	405	285	442	199	490	287	155	202	137	200	119	200	102	204	89	215	84	231	88	248	102	260
352	394	378	302	441	392	478	312	529	392	188	297	173	296	158	295	144	301	136	313	135	328	142	342	152	353
352	349	387	271	443	345	472	268	521	343	211	264	195	257	178	254	161	258	148	269	143	286	144	303	153	317
338	365	366	285	422	365	447	290	498	366	191	273	177	264	163	255	147	257	133	266	127	281	130	297	138	311
353	366	386	287	441	368	472	291	517	362	205	273	189	271	172	270	156	271	147	284	143	300	147	316	158	328
337	378	360	294	415	374	447	302	497	379	174	274	159	269	143	268	128	273	117	284	115	300	119	315	129	327
359	373	386	297	444	374	472	299	525	371	197	276	180	273	163	267	146	271	132	280	127	298	133	315	145	328
361	355	387	277	446	349	478	276	524	346	216	284	201	278	188	269	172	271	161	282	158	297	157	313	164	328
355	378	390	294	443	376	481	296	519	376	211	287	196	277	179	271	162	271	149	284	140	300	142	318	152	333
354	368	383	293	435	368	466	298	510	372	211	279	195	273	180	267	164	268	155	282	154	299	158	315	167	328
333	389	370	312	422	390	455	310	502	386	176	294	161	290	147	288	134	296	128	309	128	324	133	339	143	349
352	377	384	296	438	371	477	296	513	370	202	294	186	287	170	278	153	279	137	287	129	302	128	320	135	336
342	374	372	299	429	372	457	299	508	372	200	278	186	270	170	269	155	275	145	286	139	301	141	317	152	328
350	374	375	303	427	372	462	307	503	374	205	281	189	275	173	269	156	270	144	283	139	300	138	318	146	333
354	375	382	299	436	367	466	301	511	376	196	282	180	279	164	275	149	277	142	290	143	307	146	322	155	336
355	374	386	305	433	374	471	308	510	375	213	294	200	279	189	263	172	257	155	267	144	282	141	301	143	320
712	1069	775	872	921	1050	990	840	1132	1032	314	876	270	864	231	835	185	834	147	862	133	905	146	951	180	986
701	1170	765	965	938	1135	1008	929	1165	1103	254	988	211	967	168	948	121	947	93	985	84	1031	99	1073	133	1105
616	1039	717	853	852	1025	944	855	1079	1018	238	858	204	862	170	863	140	877	122	906	118	939	131	970	154	994
699	771	804	608	899	773	1001	617	1110	784	321	589	289	596	257	604	228	619	221	650	222	682	234	712	260	730
680	1030	772	851	896	1023	985	846	1107	1012	307	832	266	813	222	806	177	804	141	831	132	874	147	917	180	947
703	972	777	787	919	954	997	785	1114	952	290	796	243	784	196	773	152	792	129	833	125	881	150	923	189	951
685	798	754	606	894	784	965	605	1102	784	270	567	239	579	207	585	188	608	182	640	193	672	214	697	237	721
724	801	813	612	942	805	1027	606	1151	791	359	608	322	608	284	606	247	608	225	637	217	673	231	708	256	734
1050	1034	1119	826	1290	1009	1361	798	1509	998	600	865	548	850	498	828	446	835	423	883	417	937	439	986	480	1023
644	1048	735	839	862	1028	954	837	1070	1020	266	826	221	827	177	821	133	825	99	855	87	898	92	942	122	974
674	1092	743	911	896	1078	974	885	1100	1048	297	959	256	951	216	936	176	939	143	965	136	1004	158	1039	191	1066
747	1126	829	929	953	1089	1036	911	1151	1069	378	966	335	968	293	966	252	978	232	1012	226	1054	242	1090	277	1114
658	1135	712	924	875	1112	940	920	1059	1108	226	908	184	894	140	891	106	914	95	955	101	998	127	1032	163	1058
692	809	756	631	885	812	969	644	1079	817	275	635	241	600	204	571	155	567	120	598	109	646	126	690	160	726
729	1142	818	940	960	1128	1038	934	1181	1113	329	947	289	941	249	936	210	947	180	973	176	1013	195	1049	221	1080
680	1044	765	860	891	1028	983	842	1102	1002	309	895	270	894	229	883	187	880	155	904	153	943	170	980	202	1007
701	839	800	622	938	840	1052	635	1173	823	291	619	246	599	199	595	152	607	115	637	94	681	107	727	140	764
678	1126	790	926	917	1117	1006	918	1126	1098	336	878	295	871	255	857	213	861	183	887	169	925	165	967	187	1004
706	1062	804	862	930	1053	999	848	1128	1037	309	862	267	862	225	866	186	877	159	906	149	947	164	986	193	1016
619	1005	697	812	846	998	919	812	1077	982	235	798	193	782	156	749	111	755	76	789	57	833	75	874	114	903
667	830	782	628	930	832	1024	624	1155	855	307	592	255	577	203	566	156	587	114	622	98	672	107	724	142	764
713	1080	797	887	972	1062	1040	860	1206	1044	309	913	261	895	215	870	164	872	130	910	121	961	134	1011	174	1045
676	741	765	560	898	746	979	562	1102	730	298	550	253	528	206	509	158	518	119	550	99	595	113	640	149	674
729	813	799	650	904	815	983	649	1093	822	445	638	413	612	380	590	339	596	307	619	294	658	299	699	323	733
710	809	779	648	879	806	951	652	1046	816	416	634	382	616	350	597	315	607	295	638	288	675	298	712	327	736
727	805	809	624	912	799	988	625	1095	800	436	611	401	589	365	569	324	569	290	589	276	627	275	668	302	699
753	761	820	611	911	752	972	611	1048	765	487	612	457	599	432	580	398	583	371	602	359	632	365	665	387	689
769	787	827	644	940	784	999	640	1098	780	474	612	439	592	402	575	361	581	328	604	312	641	318	681	343	711
704	822	779	670	882	834	952	673	1057	844	405	638	371	636	338	626	307	639	293	671	294	705	308	737	331	762
704	806	777	657	888	810	949	652	1061	816	409	643	372	630	337	613	299	618	277	649	263	685	267	724	291	752
766	803	866	654	942	807	1032	655	1125	828	513	630	489	598	462	570	425	561	389	575	363	604	358	642	369	679
760	801	830	656	922	805	994	661	1102	805	491	620	458	596	424	574	383	573	349	594	332	631	338	671	364	702
681	760	754	610	843	761	920	618	1007	763	412	601	385	582	355	565	321	569	294	588	281	620	285	654	306	681
757	784	829	627	934	783	1009	627	1111	790	454	592	416	586	379	576	342	592	324	626	323	666	339	703	368	732
726	762	784	616	885	753	928	613	1027	752	458	573	428	571	399	563	372	574	360	602	352	631	354	662	369	687
739	787	809	639	902	786	976	637	1069	786	474	605	443	600	414	586	385	595	365	619	356	649	366	680	389	701
734	859	802	709	887	858	962	720	1045	859	455	705	421	681	392	649	355	630	325	654	318	696	307	736	319	776
757	797	841	628	951	794	1024	627	1130	794	469	616	435	590	396	577	356	590	328	619	319	660	333	700	365	729
747	761	811	619	908	753	968	622	1061	766	465	604	428	582	392	560	351	566	320	596	299	633	299	675	333	702
788	758	867	593	977	758	1052	594	1164	757	508	551	469	545	429	536	389	543	360	571	348	610	363	647	391	679
540	602	590	450	704	597	745	452	845	594	261	457	224	439	187	422	146	425	110	444	85	476	74	516	88	553
514	588	571	434	668	585	736	436	825	584	234	404	194	384	153	370	110	381	73	406	47	442	43	486	65	524
502	617	569	471	667	625	717	474	798	628	253	473	225	445	197	418	160	404	123	413	95	440	79	475	81	515
562	592	624	431	731	594	787	439	883	589	299	451	268	422	236	394	194	387	152	397	124	429	114	471	124	511
532	587	584	436	670	554	720	450	783	597	281	416	249	396	217	379	180	381	148	400	129	432	126	467	141	501
562	587	612	415	733	563	776	403	888	539	269	455	236	423	198	398	152	398	115	427	88	465	80	512	95	554
561	557	595	406	703	555	747	406	837	542	306	441	281	409	257	377	219	374	184	394	159	425	144	462	151	499
503	600	558	440	662	603	718	450	811	590	235	421	203	410	173	395	140	404	111	425	95	456	94	491	109	522
517	564	588	410	692	560	755	406	847	552	225	404	187	386	152	367	111	374	76	394	53	428	50	469	72	503
541	623	618	452	704	614	782	446	863	603	277	448	240	421	201	398	156	400	115	417	86	451	74	494	75	540
487	577	546	434	662	584	711	441	801	587	219	422	187	401	156	377	120	373	86	392	63	422	57	460	76	492
523	594	585	441	697	605	757	445	838	613	255	429	226	397	196	365	157	351	118	368	90	402	71	441	73	484
491	576	551	428	664	591	711	435	800	594	236	426	206	400	176	373	136	370	103	391	82	426	74	465	88	502
530	587	589	435	696	585	754	431	833	585	268	420	236	392	201	370	161	371	127	396	101	428	91	468	101	508
522	613	577	457	706	613	745	450	869	606	262	442	224	426	193	398	156	386	116	400	85	429	72	469	81	510
533	582	588	435	695	583	742	442	836	582	253	433	222	400	185	375	140	378	100	398	76	436	63	479	78	520

517	536	562	389	682	530	719	388	822	529	240	390	201	372	164	350	122	345	82	363	56	398	44	438	62	476
542	607	619	455	713	619	785	467	857	633	276	419	244	396	211	373	171	378	138	401	116	433	110	472	129	506
499	591	569	431	681	591	726	430	841	583	198	444	170	413	144	382	105	385	81	418	60	455	46	493	56	532
547	558	599	408	709	557	756	412	866	554	259	405	223	386	188	370	147	372	119	398	97	433	89	472	109	506
537	633	596	454	730	621	770	447	882	610	256	458	216	432	176	413	129	416	88	436	64	477	57	524	68	569
507	579	569	420	669	576	720	420	813	569	258	395	229	372	198	356	161	361	129	379	111	411	106	448	113	484
540	585	592	403	711	575	757	400	847	559	240	396	203	362	158	344	111	356	73	388	50	431	45	479	70	521
539	550	603	408	697	552	757	414	842	553	275	389	243	365	204	352	166	362	132	384	109	416	107	455	125	490
516	576	580	415	688	573	742	424	830	562	270	420	238	395	204	376	164	375	130	398	107	431	95	469	105	508
550	564	593	403	717	558	757	395	844	553	269	396	231	375	195	350	151	349	117	378	91	413	82	456	100	495
538	570	609	417	715	577	776	420	855	577	256	419	224	397	192	372	151	376	117	400	97	435	86	475	100	513
528	591	578	437	689	591	741	434	828	586	231	422	199	395	160	384	120	396	89	424	68	460	64	502	91	533
506	592	575	433	680	600	729	447	815	608	248	436	222	405	196	374	157	361	120	377	89	404	77	443	81	484
562	578	629	433	727	590	787	444	871	593	315	417	284	387	253	356	210	354	169	371	143	404	135	446	151	485
521	566	577	429	681	572	723	429	811	563	283	411	252	383	217	364	176	364	141	385	116	418	104	457	119	494
514	556	581	407	689	565	743	408	830	569	276	396	235	372	201	337	156	323	112	341	77	375	59	420	63	468
534	579	586	423	704	582	742	436	845	579	259	439	225	413	195	385	153	384	114	399	83	429	64	466	72	505
561	592	610	430	728	587	778	438	889	598	279	439	240	426	203	406	164	399	126	415	98	445	89	485	106	521
547	561	595	401	722	559	759	397	870	541	266	441	234	421	204	396	166	381	130	393	103	423	91	462	105	499
524	559	563	425	665	551	705	418	799	538	258	429	229	426	201	418	174	422	163	449	160	478	166	506	187	525
550	610	599	458	707	598	761	455	854	587	274	479	242	467	210	454	177	458	153	482	142	515	148	548	172	572
535	572	594	416	697	548	753	408	843	539	244	462	209	450	175	440	141	452	119	480	109	514	111	550	130	579
585	553	646	405	733	549	800	401	877	545	321	405	293	409	266	415	241	428	227	453	230	480	247	501	273	512
542	627	609	478	693	625	758	479	847	615	276	475	247	456	217	440	183	444	162	471	155	505	166	537	187	564
512	604	579	474	662	589	739	467	818	587	249	487	219	475	189	467	159	475	139	499	137	530	152	558	176	578
515	540	588	404	684	538	749	412	840	536	240	381	209	378	177	379	148	389	123	407	121	438	134	466	156	489
532	564	607	419	696	560	768	423	840	565	257	399	225	402	196	408	167	423	141	441	132	470	147	496	173	513
529	579	589	438	686	571	731	422	836	552	233	459	201	458	170	452	140	452	122	478	114	509	125	537	148	558
542	559	605	396	705	555	754	404	859	561	242	403	205	393	169	384	136	401	103	420	88	453	98	489	128	510
516	584	566	433	668	564	718	427	804	560	252	420	223	421	193	419	166	428	157	453	156	482	162	511	181	532
578	592	638	446	743	591	803	445	907	594	281	465	247	459	213	454	181	466	160	493	155	527	173	555	204	572
530	604	609	475	693	607	757	468	844	600	270	461	240	456	211	452	183	463	161	483	157	513	165	542	185	564
568	558	626	413	710	559	771	425	844	571	291	393	261	381	230	370	197	374	177	398	168	430	175	462	198	485
519	548	580	396	655	543	709	390	805	529	273	409	241	401	210	389	178	395	152	416	144	448	145	481	169	503
518	594	557	459	652	584	697	450	787	579	258	474	231	467	206	456	180	461	158	476	146	501	146	527	160	550
530	514	595	374	687	516	741	373	842	510	261	365	236	368	211	374	191	389	176	409	174	433	185	455	205	471
540	568	610	434	698	568	777	449	847	572	262	407	233	410	203	410	175	419	159	442	154	471	164	498	186	518
522	543	586	417	678	543	731	421	826	548	251	412	222	411	194	414	168	425	154	449	156	476	170	501	193	517
522	639	575	482	676	621	718	463	818	614	260	493	229	476	199	458	163	461	136	483	129	519	131	555	150	584
491	586	536	446	631	572	693	436	775	566	217	458	187	455	156	453	129	461	112	486	112	516	129	541	154	558
555	609	619	482	698	603	754	484	834	604	292	485	262	470	233	460	202	467	177	489	162	519	159	552	176	581
507	542	583	411	653	532	722	408	803	532	260	386	232	384	205	379	184	396	174	422	174	451	181	477	199	498
533	598	580	465	670	589	723	468	806	586	263	451	237	439	209	435	185	446	172	470	167	498	174	525	194	545
501	547	545	414	655	532	687	398	788	517	235	432	209	430	183	428	159	434	148	455	151	481	164	502	184	519
515	523	580	408	653	521	705	409	781	524	280	409	256	402	234	385	210	380	191	399	177	421	176	448	187	472
465	639	499	506	614	618	639	490	739	603	195	540	170	543	145	542	123	549	117	574	123	598	134	620	151	638
498	549	565	429	629	530	687	418	742	507	301	476	274	469	249	458	222	460	202	478	195	505	191	532	207	553
493	600	543	481	621	592	671	478	743	582	282	496	256	484	234	467	206	459	186	478	179	506	177	534	186	560
496	603	562	452	654	585	704	442	805	572	233	478	209	486	184	489	164	503	156	526	162	549	178	569	199	583
761	771	810	625	922	768	967	628	1066	778	492	604	457	588	423	574	388	581	368	613	352	649	351	685	371	717
798	782	839	631	973	761	1007	628	1130	783	504	642	465	621	427	598	384	601	348	626	325	664	331	707	362	738
758	799	801	646	913	789	966	661	1058	808	484	650	451	627	422	595	381	592	353	621	331	658	331	700	356	733
805	776	854	644	941	771	979	642	1066	779	588	651	556	636	528	610	494	596	469	622	443	650	433	686	447	717
732	766	796	620	906	763	956	622	1071	767	448	637	414	619	383	599	347	609	321	636	309	673	315	710	341	737
750	817	825	645	932	819	997	652	1092	828	460	676	429	650	393	629	355	644	325	674	315	715	324	754	356	781
802	818	874	654	981	810	1048	657	1158	821	541	668	504	637	464	614	416	620	376	644	353	686	359	733	386	770
746	745	801	595	907	734	946	604	1052	753	487	600	454	596	423	579	391	570	364	591	349	622	350	657	369	686
775	816	824	680	932	815	975	673	1097	824	495	680	461	667	429	649	393	651	369	680	355	716	358	755	386	783
776	791	824	632	942	785	993	644	1105	824	506	657	476	626	445	601	402	601	366	623	345	660	345	703	368	739
729	784	795	648	893	779	943	653	1048	788	487	645	453	626	422	602	385	593	355	615	336	649	326	686	342	720
792	780	857	633	954	769	1011	632	1114	775	565	639	530	621	496	599	460	586	431	608	420	645	402	680	405	717
764	736	823	598	921	734	966	604	1045	757	515	598	479	587	448	557	409	555	376	580	354	617	355	659	382	691
710	740	762	595	875	734	924	595	1027	757	473	603	437	588	406	564	368	558	334	574	318	609	317	648	332	683
850	783	890	618	1000	774	1064	622	1164	788	563	650	526	630	496	597	458	593	432	630	419	672	420	717	439	749
793	766	863	618	964	767	1027	624	1122	784	512	633	479	617	445	593	405	602	375	632	351	666	358	704	387	729
731	769	792	634	887	769	942	631	1047	782	503	613	471	604	443	582	408	584	380	606	367	638	368	672	388	700
802	804	849	642	960	785	1022	641	1119	805	532	666	500	646	467	625	428	632	397	656	383	693	388	733	410	764
742	744	798	584	897	729	949	592	1048	755	475	610	439	607	410	583	374	579	346	601	331	634	336	670	360	697
766	768	801	629	926	764	956	625	1064	774	500	639	467	626	438	603	405	587	379	608	367	643	364	678	374	712
751	785	817	625	924	776	976	629	1080	786	471	650	436	623	405	587	362	585	324	613	301	654	295	701	319	740
539	561	577	406	696	548	734	399	841	546	270	399	233	383	198	361	161	368	141	404	132	445	138	485	164	516
535	607	592	460	700	603	752	464	869	624	249	465	211	443	172	422	127	418	93	448	71	486	81	530	99	570
547	604	610	435	720	596	776	435	890	615	225	427	186	418	148	407	114	426	97	464	95	504	112	541	141	568
524	561	600	402	698	551	760	400	867	563	258	406	221	379	184	354	139	353	106	382	88	424	89	469	113	507
494	583	532	419	664	572	707	408	850	581	164	362	126	368	92	387	68	418	62	456	70	494	93	525	122	550
537	602	612	438	708	590	789	440	886	602	269	469	232	447	194	423	151	429	117	459	97	499	104	543	135	576
911	934	987	680	1156	918	1249	682	1394	941	473	744	427	705	377	668	316	684	268	723	242	780	263	839	304	886
942	1117	1038	887	1197	1099	1285	880	1461	1129	542	880	493	841	437	816	374	825	319	856	287	910	297	971	340	1019
992	889	1044	656	1221	880	1301	646	1482	889	557	665	501	626	440	601	374	622	332	674	324	742	345	807	394	854
918	913	994	673	1171	903	1242	677	1411	929	483	698	426	669	370	636	305	633	255	672	234	734	256	796	308	838
895	1048	980	818	1166	1053	1226	822	1409	1091	487	849	442	812	393	774	336	758	282	788	251	839	260	898	296	947
914	1139	1006	846	1201	1113	1294	851	1463	1137	406	915	368	848	304	807	229	815	171	865	145	938	156	1013	204	1073
911	878	1038	600	1221	873	1331	612	1501	890	433	641	384	596	328	556	258	558	201	598	175	664	184	734	233	787

941	1086	1028	785	1243	1046	1333	783	1543	1061	428	803	363	768	299	733	228	747	184	805	159	873	162	945	207	1001
921	1120	1048	819	1253	1098	1367	830	1562	1127	363	841	296	810	226	791	158	814	118	874	106	946	131	1015	185	1063
973	897	1085	613	1304	863	1394	593	1587	858	463	647	410	600	357	551	289	562	237	611	213	677	226	746	272	801
889	1052	995	834	1168	1030	1246	822	1401	1044	475	883	420	863	368	841	313	853	273	896	251	950	264	1008	301	1052
872	1086	987	824	1158	1059	1246	812	1399	1074	465	822	412	788	370	734	305	720	246	748	221	809	228	876	261	933
909	846	1055	568	1217	830	1360	569	1514	859	419	615	365	579	312	540	250	537	193	570	163	630	167	696	208	749
914	1051	1038	766	1234	1025	1355	764	1538	1044	429	832	368	790	302	758	229	769	181	821	167	892	186	963	238	1013
924	1105	1019	829	1212	1086	1317	817	1495	1097	419	869	360	833	297	802	230	821	179	870	158	937	160	1007	208	1056
916	1113	1051	849	1228	1100	1324	852	1509	1132	475	878	409	840	341	811	265	807	203	844	168	908	184	978	224	1042
975	1093	1080	807	1275	1058	1377	802	1567	1071	441	837	373	817	307	793	239	805	212	871	202	941	229	1006	274	1063
972	1081	1068	783	1275	1071	1373	791	1572	1097	417	885	423	803	388	727	314	695	250	751	187	810	161	890	194	966
992	1000	1116	686	1295	986	1407	700	1607	1005	436	761	410	682	370	609	286	593	216	640	168	713	155	799	192	874
885	1125	1023	859	1228	1105	1345	835	1512	1103	411	854	341	828	275	793	201	795	149	851	119	921	117	998	165	1054
938	1178	1043	886	1241	1154	1345	885	1545	1176	384	888	316	863	250	826	181	828	137	890	113	961	130	1032	183	1086
800	744	866	602	962	741	1032	600	1127	755	545	617	509	602	475	584	437	590	405	614	387	650	388	689	414	721
525	599	580	433	697	596	747	442	853	630	252	421	213	400	174	374	130	371	99	404	81	446	85	492	112	529
530	578	594	423	705	568	759	421	849	574	241	420	210	389	180	360	138	372	107	402	88	442	97	484	128	515
513	660	572	488	693	651	750	482	847	633	222	503	190	472	153	450	109	453	76	483	63	526	70	570	99	604
537	598	601	420	720	589	779	421	894	601	238	438	199	413	163	388	117	391	80	418	63	461	70	506	100	542
897	867	1001	593	1169	837	1284	604	1423	895	401	599	358	538	315	478	242	494	184	542	148	608	153	682	197	742
951	1132	1073	873	1246	1129	1358	870	1509	1175	399	958	366	889	304	852	229	844	169	883	154	957	178	1027	237	1073
951	1167	1082	885	1301	1154	1405	881	1597	1186	409	852	340	821	266	808	191	821	135	873	111	943	131	1016	181	1074
1058	896	1128	645	1307	896	1401	650	1564	932	530	611	472	573	410	537	340	547	288	594	258	660	266	728	314	781
945	1037	1053	791	1241	1037	1335	808	1492	1076	458	832	411	776	360	726	291	735	225	766	186	820	207	889	260	938
807	782	844	648	960	779	1004	650	1108	778	528	655	498	639	468	621	434	621	412	644	402	677	409	710	429	736
741	806	800	684	912	810	953	689	1063	813	488	667	459	664	429	652	397	649	371	665	361	695	366	727	386	752
797	778	850	643	956	774	1004	644	1108	779	527	641	493	624	464	599	428	602	398	624	382	656	384	694	409	721
533	572	577	415	723	551	766	399	881	555	236	442	204	420	177	391	142	378	106	394	79	420	78	460	95	495
508	520	550	357	693	501	741	339	870	496	197	405	162	380	134	347	94	329	56	348	36	385	39	428	58	465
505	640	578	478	698	642	765	484	878	663	219	511	185	478	163	435	132	400	88	419	60	456	51	504	61	550
530	607	600	460	708	606	771	469	871	618	270	460	237	440	206	415	168	412	132	425	116	460	121	499	140	533
499	636	560	487	689	634	748	487	861	653	215	497	182	482	152	462	116	461	84	479	67	511	73	547	95	577
509	591	571	446	693	586	751	449	847	576	226	453	188	444	152	426	113	415	79	433	63	468	66	507	90	539
504	654	572	490	691	645	756	484	858	648	225	534	198	502	176	466	141	449	104	469	80	503	80	542	108	573
503	593	585	440	689	590	757	445	847	607	252	457	224	429	199	395	163	382	127	402	101	434	97	475	114	513
499	599	563	464	675	590	727	460	842	606	234	467	199	455	170	428	135	422	103	443	90	479	89	517	107	551
503	609	572	451	691	599	751	436	864	601	225	485	194	456	169	420	130	404	89	419	63	454	67	497	89	536
502	625	564	471	678	624	731	460	823	612	234	492	203	466	180	429	148	406	106	414	80	446	77	489	89	532
495	590	570	430	690	585	740	423	854	586	193	487	164	454	142	415	103	406	67	435	37	466	35	511	54	551
964	908	1042	706	1227	895	1287	694	1460	891	562	699	516	685	474	659	426	661	385	689	361	729	371	778	400	816
937	1101	1014	833	1246	1094	1328	827	1545	1127	388	879	341	831	300	770	237	739	173	775	133	832	138	904	168	971
987	1098	1069	823	1307	1081	1387	812	1591	1087	456	838	405	792	354	747	288	733	225	759	200	820	203	888	237	947
963	1147	1027	852	1269	1134	1335	845	1559	1155	403	849	349	812	298	769	233	755	175	786	153	848	156	916	184	976
894	1117	960	897	1162	1106	1228	885	1389	1109	463	929	414	906	377	865	327	854	279	879	260	929	269	982	304	1024
967	883	1101	613	1280	881	1409	627	1594	917	489	659	428	620	370	583	297	579	237	618	203	681	209	751	249	812
1019	1093	1121	791	1331	1069	1421	798	1619	1112	489	851	424	818	368	768	299	786	232	817	200	884	211	956	256	1012
978	1208	1072	929	1282	1190	1384	939	1587	1213	473	966	411	929	348	895	276	899	216	941	191	1009	207	1079	252	1137
773	807	829	643	944	809	995	647	1104	829	515	645	478	617	442	589	396	590	361	620	349	662	361	706	391	740
758	810	806	672	916	812	967	678	1067	841	499	673	462	656	424	643	386	649	362	679	351	717	362	754	391	780
776	802	823	647	948	794	993	643	1098	820	506	648	473	623	436	604	398	617	374	651	360	690	365	730	388	764
774	776	803	625	929	764	970	625	1085	785	486	643	453	616	422	585	377	588	343	619	323	661	327	706	354	741
744	742	790	587	902	730	959	590	1055	751	488	579	448	566	413	541	373	542	344	572	328	611	331	654	354	688
772	834	820	661	941	831	994	665	1114	854	491	685	453	660	419	627	374	633	342	668	325	713	331	759	362	793
770	777	808	608	933	774	985	618	1100	798	493	630	465	592	432	560	385	561	353	594	335	638	336	684	361	724
787	791	836	636	938	786	997	651	1086	819	520	653	487	634	459	603	420	602	388	627	372	665	372	707	390	742
752	754	788	610	912	753	957	619	1070	788	470	631	434	615	398	596	359	596	332	627	316	665	320	705	346	736
797	754	830	611	945	749	985	620	1104	772	536	613	504	605	477	584	447	574	425	599	410	630	408	664	424	693
825	717	841	566	973	706	1001	571	1120	730	553	581	524	565	500	538	466	534	445	563	433	597	430	633	446	663
766	833	791	678	937	818	954	674	1094	839	472	712	443	679	411	650	367	651	332	675	314	714	323	754	351	785
745	751	790	606	901	749	946	617	1045	765	504	599	471	587	438	568	404	565	379	591	362	625	355	660	369	694
762	768	797	623	927	758	960	624	1071	793	480	623	452	603	427	575	393	572	362	592	347	626	350	662	367	694
814	780	832	632	972	773	1004	629	1125	782	501	662	466	637	431	613	388	614	354	640	338	680	356	719	387	751
1026	1046	1099	764	1326	1022	1395	751	1616	1054	523	820	448	806	379	772	307	798	253	851	236	926	255	1000	312	1051
853	1130	951	876	1177	1117	1265	873	1460	1129	351	922	297	888	239	860	176	851	125	887	99	944	116	1005	156	1055
766	774	805	643	909	761	971	629	1062	776	492	663	462	648	435	632	403	635	381	657	372	689	375	720	392	749
730	784	778	648	879	773	924	645	1023	789	488	651	457	643	428	630	397	626	371	640	357	667	357	699	373	725
737	746	795	610	891	738	952	606	1036	760	488	618	457	603	436	577	401	578	377	595	365	627	360	661	364	691
792	780	841	647	948	775	993	647	1080	790	546	652	518	639	494	618	463	615	434	626	416	653	413	684	424	713
769	779	831	628	944	771	1005	636	1088	806	488	652	462	630	442	597	410	583	379	596	363	629	358	666	367	703
711	780	779	641	878	776	944	642	1034	804	459	650	427	633	402	606	366	599	334	618	317	650	312	687	327	720
750	802	801	661	910	784	960	648	1045	797	493	652	466	650	441	635	414	632	396	649	389	677	394	705	406	729
787	790	846	648	957	788	1014	651	1114	821	517	659	488	637	464	611	428	613	401	637	388	670	387	707	406	738
774	773	831	639	940	762	986	630	1075	778	519	625	494	610	469	589	440	593	418	616	403	645	404	678	414	706
713	756	753	629	844	746	888	631	972	751	476	642	449	633	428	609	405	589	377	597	358	621	348	650	355	679
507	549	589	410	689	549	754	414	837	570	250	432	219	418	192	395	158	394	127	412	109	443	104	478	117	510
505	540	568	382	692	520	747	371	849	535	233	416	196	403	164	379	126	372	95	398	75	432	72	471	90	507
506	561	575	407	691	560	759	407	845	575	224	425	186	407	154	379	113	369	73	382	46	415	44	456	63	495
515	585	594	441	702	582	762	445	857	615	258	469	224	445	196	413	156	401	118	418	93	453	87	495	101	534
480	577	540	432	645	562	705	427	793	571	224	433	193	427	167	410	137	400	110	412	94	438	93	469	104	498
536	562	579	418	688	545	733	410	832	560	256	466	226	442	198	411	157	403	118	417	88	445	85	487	102	524
496	613	564	469	662	608	749	471	822	630	249	463	212	453	178	436	143	420	109	433	91	467	87	505	101	541
527	617	574	462	687	606	746	460	847	624	261	489	225	478	192	456	156	444	122	461	97	492	87	530	96	568
509	606	581	455	700	588	751	445	855	600	251	500	216	483	190	452	154	439	119	457	94	489	90	529	97	568

510	581	561	420	685	561	739	414	831	583	230	470	195	446	173	404	133	384	89	401	57	436	49	483	63	527
460	614	521	466	620	607	692	456	783	616	189	471	159	461	131	447	102	436	76	450	65	479	63	510	75	539
523	594	584	435	698	582	755	434	862	604	252	466	219	441	190	410	152	398	114	416	87	449	76	489	88	530
510	556	576	400	692	549	748	393	843	560	243	435	209	415	180	385	144	366	105	376	79	408	68	447	76	487
494	558	574	403	689	536	748	389	845	560	214	414	179	395	143	377	105	372	73	392	56	428	57	467	84	497
495	595	566	443	655	577	725	437	808	608	246	478	215	457	183	437	146	429	114	449	97	482	91	520	107	554
490	561	567	410	674	550	741	415	835	577	232	423	206	389	179	356	139	342	99	360	71	391	60	432	59	474
484	594	567	444	671	591	737	449	817	612	227	477	191	460	161	429	124	417	86	436	64	471	58	514	78	550
539	624	603	478	705	610	771	477	852	626	262	500	234	477	210	450	174	443	138	455	115	485	112	521	131	552
916	1003	988	777	1156	984	1242	773	1368	1008	511	841	466	804	420	766	362	762	309	788	282	841	288	900	323	949
938	877	1023	639	1209	861	1295	641	1414	882	504	687	458	644	413	598	352	587	299	624	267	679	267	744	300	799
863	979	945	761	1099	970	1195	770	1318	992	444	817	409	776	371	734	315	743	262	767	230	813	232	870	260	920
888	830	983	604	1142	820	1254	611	1357	860	426	668	380	625	341	570	273	571	214	603	169	654	154	720	183	779
899	1101	983	863	1161	1073	1247	851	1385	1099	478	917	420	886	377	836	318	819	261	846	225	900	221	965	252	1022
513	579	560	400	677	569	736	398	840	569	178	439	145	422	110	411	74	412	52	440	47	476	59	511	88	534
528	587	591	416	705	583	779	420	876	575	206	403	176	383	142	375	109	389	86	416	74	450	80	483	104	509
538	645	613	461	729	643	796	462	914	626	237	415	194	413	153	403	117	418	101	457	98	499	110	539	135	573
499	611	596	442	682	614	781	451	866	623	205	451	170	438	136	418	98	413	71	439	61	477	61	516	75	552
497	629	567	456	683	631	746	460	854	611	147	444	116	441	87	434	68	455	62	485	64	516	77	543	100	564
575	543	614	370	731	538	792	374	893	538	209	385	171	372	132	367	105	397	97	436	106	476	129	510	159	538
471	570	567	410	653	573	737	428	819	584	197	402	163	394	129	381	96	387	71	412	63	447	74	479	98	506
513	602	572	435	680	611	747	456	837	622	204	417	169	400	134	380	96	383	69	413	60	451	69	488	96	518
544	553	608	396	718	546	776	387	861	534	262	405	226	393	190	380	153	382	127	409	118	446	129	481	157	508
478	586	560	437	654	578	728	429	822	563	211	438	176	427	141	418	105	425	78	447	67	482	73	518	96	547
484	598	545	444	667	596	727	439	844	592	156	435	137	446	115	453	102	472	94	492	92	515	99	535	111	553
390	599	429	480	503	587	555	481	627	578	166	493	142	482	119	470	94	476	75	493	69	519	75	545	93	564
355	425	384	331	463	419	494	338	569	411	161	337	140	333	120	325	99	328	86	346	86	368	93	389	107	405
431	473	456	379	532	474	570	387	640	476	217	361	195	355	176	343	158	355	147	376	142	400	149	422	165	438
321	541	351	443	437	535	479	438	570	529	107	433	87	427	68	412	47	418	32	437	26	460	31	482	48	499
477	573	539	444	623	573	665	443	752	557	223	430	191	425	161	412	132	425	112	451	101	482	103	515	119	542
514	587	571	469	673	573	710	453	795	564	279	477	255	463	229	450	203	459	185	483	176	511	179	540	198	562
477	619	545	501	620	617	682	499	768	617	235	488	208	489	181	492	156	504	135	522	128	548	134	574	154	591
511	582	571	449	650	570	704	445	794	568	281	449	252	439	223	446	191	450	167	471	156	501	158	533	179	557
566	616	616	488	697	609	744	480	822	606	320	453	291	450	262	451	237	465	220	488	212	515	220	542	239	563
516	482	583	363	650	477	712	366	784	483	257	363	228	358	200	351	172	356	155	378	153	407	165	433	186	453
551	602	592	468	689	601	738	464	830	592	290	480	262	462	234	446	206	459	180	478	166	508	169	539	191	563
507	616	567	482	640	614	701	481	774	614	276	497	252	490	228	484	204	493	189	512	187	536	193	560	209	580
495	540	540	418	627	543	673	410	761	531	269	423	243	415	218	403	193	403	171	420	161	445	162	472	175	496
550	631	603	492	704	622	752	474	848	606	291	493	259	478	228	463	194	469	167	491	150	521	148	555	171	580
514	612	560	472	653	608	705	470	802	599	257	444	227	430	196	418	162	422	134	442	125	475	129	509	148	537
479	590	565	467	635	587	730	485	769	601	255	433	226	419	196	408	164	411	136	426	121	455	121	487	138	514
504	664	573	536	646	653	703	532	791	644	271	537	245	523	217	517	195	535	176	557	170	586	175	614	193	636
453	492	520	345	619	482	677	346	760	477	191	365	163	348	138	324	106	313	78	330	66	362	69	396	87	424
554	530	610	398	712	521	774	386	867	519	295	410	269	403	242	401	218	409	198	426	190	450	199	474	217	493
505	575	572	438	679	570	735	431	829	562	272	416	244	413	216	418	191	432	169	450	157	476	157	504	167	530
498	589	557	442	664	575	715	428	819	556	215	448	181	445	148	442	116	453	90	475	80	506	90	537	116	559
527	625	580	485	673	610	723	476	813	599	253	491	231	465	206	442	173	451	153	480	139	512	137	547	154	577
469	566	519	422	631	545	672	404	785	522	183	434	153	430	123	433	96	445	79	469	79	499	93	525	112	549
528	616	600	483	702	616	756	484	847	609	267	479	233	463	198	448	161	461	129	484	107	516	118	551	149	575
525	596	588	459	696	594	748	465	845	594	254	441	226	427	198	414	170	426	150	449	139	478	146	508	170	529
502	575	562	435	641	572	705	439	782	571	254	435	229	423	208	405	183	397	160	411	158	439	162	467	177	489
513	548	572	407	665	526	715	394	810	509	256	423	228	412	201	399	173	403	150	423	139	451	146	480	164	503
516	535	589	410	654	539	728	417	794	547	298	418	274	414	250	411	226	415	212	432	209	455	215	478	228	499
519	591	570	456	665	578	718	456	805	581	240	500	217	487	193	482	172	496	160	520	159	547	171	570	195	584
487	580	545	454	634	566	687	444	762	551	239	472	215	455	191	438	168	447	156	473	150	502	154	531	171	554
525	562	586	436	670	555	723	435	809	552	277	428	252	428	228	425	208	436	196	457	197	481	205	504	223	520
546	557	610	418	680	542	747	411	816	542	312	420	281	418	250	414	222	424	197	443	183	470	186	500	208	521
599	569	639	446	725	562	796	452	873	565	331	466	304	455	280	439	254	451	236	475	228	504	239	532	264	549
610	567	661	449	728	566	782	456	834	565	388	443	362	430	338	412	313	418	299	443	291	472	290	501	299	528
489	561	554	417	642	560	695	426	787	567	274	413	251	394	233	371	203	371	181	388	169	416	172	446	185	472
551	513	577	394	680	499	720	388	813	504	301	414	277	394	253	378	226	388	202	410	187	437	186	468	209	490
568	563	618	418	714	570	772	434	860	591	297	418	273	392	244	370	207	371	180	396	175	433	182	471	210	492
482	605	525	479	632	586	670	464	769	593	226	484	201	480	177	465	152	464	133	480	129	507	134	534	148	559
539	578	606	443	691	571	751	446	835	592	298	451	270	442	246	420	214	416	188	433	176	463	178	494	200	518
518	565	581	409	688	554	744	409	844	560	253	440	218	421	185	399	149	400	124	431	110	468	116	507	146	533
530	597	590	441	708	588	769	446	881	598	222	472	202	445	181	418	143	411	118	439	101	473	98	509	120	540
537	571	604	421	713	560	775	414	865	572	249	455	216	428	185	398	146	384	112	410	95	450	98	493	125	526
483	533	549	401	644	527	687	392	788	533	233	417	208	390	183	364	148	358	120	380	100	410	98	446	119	475
547	535	599	385	695	533	772	388	863	534	250	389	222	367	196	341	161	335	133	358	118	391	122	428	144	457
507	531	595	380	701	539	772	396	868	558	241	362	210	343	176	326	139	332	111	358	99	394	107	431	133	459
341	508	375	404	460	489	481	397	566	494	165	423	139	419	116	407	90	404	67	416	54	439	58	465	78	481
362	493	406	395	489	487	511	384	587	492	193	402	169	391	148	374	121	374	96	386	84	410	87	438	105	458
549	481	600	356	707	479	739	353	833	485	341	360	310	351	283	329	249	332	218	348	207	380	213	412	234	438
479	597	530	454	627	590	670	449	763	598	247	464	214	446	182	424	145	425	124	456	117	494	129	530	156	557
1314	1187	1387	923	1573	1169	1689	920	1869	1200	785	990	732	929	666	885	586	901	524	956	495	1030	514	1108	570	1164
1211	1239	1334	963	1503	1225	1618	963	1793	1248	722	1021	673	965	603	943	532	964	487	1024	469	1096	497	1163	560	1204
1183	1270	1304	993	1469	1261	1574	996	1750	1276	656	1077	610	1020	540	1003	475	1035	437	1097	421	1169	455	1234	516	1276
516	581	601	424	682	586	759	441	846	628	218	450	212	406	187	369	144	356	106	380	86	421	87	467	109	507
526	601	595	434	692	595	761	440	845	609	234	459	213	415	184	376	138	376	99	407	74	449	72	498	98	538
543	534	596	364	716	528	786	371	878	557	205	388	166	365	125	346	83	341	51	373	30	413	39	455	75	481
491	604	562	459	651	613	722	471	799	628	252	459	221	435	189	411	150	411	115	430	95	464	99	503	126	534
561	537	635	398	723	534	787	403	862	551	344	391	312	374	281	352	244	350	211	368	188	399	179	435	198	469
524	460	595	305	700	471	764	315	851	492	275	296	242	268	210	240	166	232	128	250	104	287	105	331	126	369

529	468	609	316	697	471	767	323	848	478	267	308	235	292	204	266	164	267	131	293	116	331	120	372	138	409
563	595	658	452	725	606	797	461	876	627	345	396	310	371	274	350	234	363	205	394	189	433	191	475	212	511
522	501	598	358	676	498	758	360	827	518	269	376	241	343	211	316	170	313	135	338	112	371	110	412	131	447
513	536	589	395	686	524	752	407	845	543	235	410	203	380	173	347	129	347	91	373	67	409	75	453	104	486
459	451	516	297	603	450	661	310	743	480	184	296	149	269	115	240	74	259	44	292	39	335	58	376	93	402
528	552	594	395	695	546	762	395	861	572	246	383	208	358	169	334	122	340	88	371	82	418	96	464	131	497
564	440	638	285	740	436	817	292	901	467	297	278	260	255	223	234	180	237	141	256	123	294	131	335	163	364
507	540	573	400	676	535	747	403	821	547	244	392	210	369	178	342	136	346	104	374	80	411	78	453	103	488
513	518	588	347	678	516	762	353	845	534	223	341	179	325	133	319	90	339	65	379	47	423	56	468	92	497
492	462	557	305	667	450	734	315	835	480	218	319	185	290	155	258	113	256	82	284	67	325	64	367	81	406
517	498	594	335	688	486	769	343	853	495	261	350	229	322	200	287	159	297	130	332	114	376	116	420	141	457
556	504	616	330	734	489	800	331	906	509	255	334	222	298	188	268	141	272	106	303	91	347	101	394	131	430
548	496	604	326	725	483	785	320	882	496	268	339	227	324	190	301	147	305	109	326	90	365	100	408	123	444
579	465	660	295	764	464	843	292	941	483	260	312	235	270	201	236	152	232	112	259	89	303	88	349	118	388
547	613	626	454	731	628	815	471	917	663	250	458	229	412	207	368	159	379	116	405	88	447	96	492	123	535
536	652	609	483	710	654	791	497	854	670	268	498	230	467	188	441	137	446	98	478	83	526	96	573	126	612
1239	1160	1346	922	1517	1146	1633	911	1784	1178	779	965	735	904	703	830	632	801	562	839	514	905	490	982	508	1061
1179	1129	1305	869	1487	1113	1634	873	1759	1137	665	964	644	879	593	813	508	812	434	860	378	927	367	1012	406	1087
1245	1124	1359	869	1530	1115	1656	862	1832	1154	797	941	736	895	691	826	629	783	556	815	516	886	481	959	496	1037
521	519	612	358	709	525	797	371	871	539	249	343	212	310	183	264	132	257	87	286	55	329	51	382	73	431
513	474	599	308	702	482	772	318	858	492	240	312	201	276	167	232	115	233	63	252	31	296	37	350	70	393
519	469	595	340	675	467	746	364	813	496	304	323	271	302	238	281	199	280	166	302	142	333	140	372	157	407
468	568	532	404	615	566	698	426	766	594	203	438	177	404	147	375	106	370	67	385	42	420	37	463	49	504
556	486	614	330	709	486	785	342	863	515	253	362	228	320	202	280	157	282	120	313	93	352	86	399	110	440
493	573	574	426	658	573	738	442	821	604	230	459	204	424	171	395	126	390	84	409	63	450	64	496	87	535
475	510	554	364	623	510	714	382	772	529	249	350	216	329	181	308	141	303	106	323	87	357	87	397	110	429
492	519	569	383	658	523	735	385	813	551	233	375	211	343	191	302	147	292	106	314	77	351	66	395	80	439
569	528	653	386	746	536	816	409	906	573	342	396	308	364	274	333	230	328	188	345	159	381	158	427	178	468
454	505	526	371	612	512	685	388	758	541	222	357	188	337	157	314	117	312	82	332	60	365	57	406	75	441
485	528	576	381	665	532	739	397	827	562	255	397	217	371	183	340	138	331	94	342	65	378	53	423	71	466
527	432	604	277	712	438	778	291	866	458	268	295	234	266	202	231	159	217	119	236	91	275	87	322	103	367
500	481	575	329	674	476	732	327	819	501	254	352	222	319	192	284	147	272	102	288	74	327	63	374	78	418
487	532	549	379	671	530	722	390	819	546	219	396	185	380	156	350	115	345	82	369	63	405	59	447	69	486
491	568	576	420	663	567	741	444	827	607	249	402	214	374	178	352	133	356	98	382	68	417	63	460	82	500
526	431	592	288	695	440	760	309	845	464	258	282	228	259	203	229	164	221	135	243	119	280	112	320	119	358
510	508	599	346	687	501	768	352	841	518	223	376	199	335	179	290	143	271	105	300	73	336	62	382	80	425
688	706	727	616	786	700	828	627	865	710	527	623	505	612	486	593	464	587	441	600	425	620	422	646	432	669
711	690	766	595	827	687	875	597	936	699	537	599	521	578	500	565	475	571	454	588	439	610	436	636	452	656
721	721	771	621	832	717	876	627	944	728	553	635	534	615	516	595	489	590	465	605	452	630	450	659	461	683
715	698	750	598	830	693	865	594	933	707	513	620	499	598	479	580	455	571	431	581	417	604	420	630	433	652
778	677	814	586	889	685	925	586	989	693	606	606	587	588	570	569	544	569	525	586	518	611	518	638	529	663
713	732	743	619	813	715	851	619	916	723	527	630	502	611	480	590	452	598	435	624	425	653	428	683	442	710
692	670	725	583	787	665	819	586	873	676	517	594	505	570	494	547	469	542	448	556	434	578	431	604	440	629
678	683	719	591	781	680	825	591	874	693	517	609	496	595	479	574	454	567	430	579	413	600	407	626	416	651
719	722	762	633	820	728	863	636	915	743	543	650	525	628	510	603	484	595	458	608	438	629	430	656	443	680
828	734	876	647	932	739	977	658	1026	759	655	635	635	613	613	593	583	591	558	608	545	634	547	663	564	687
707	686	757	585	820	684	864	589	910	681	533	616	523	584	507	555	476	546	445	555	426	582	425	614	437	643
762	683	799	583	869	684	910	584	971	696	598	586	573	571	551	550	524	539	496	545	480	569	476	599	484	628
723	679	765	574	841	675	880	581	933	694	527	598	513	571	498	545	470	542	443	556	425	581	424	612	438	639
673	692	710	594	779	692	817	596	869	703	509	608	487	597	470	579	448	570	428	581	417	603	414	627	427	649
774	711	790	600	865	702	896	598	958	705	569	627	558	596	536	572	506	571	479	588	470	619	471	653	489	679
806	683	850	582	915	683	964	583	1024	697	626	588	602	571	580	555	551	555	526	569	514	595	518	623	530	650
765	660	806	563	871	663	905	570	965	678	594	573	574	553	557	528	529	524	502	539	485	563	482	593	495	619
774	727	818	640	881	724	925	642	982	736	597	661	576	647	561	626	536	621	514	633	499	654	497	681	512	702
622	729	663	626	722	722	761	627	821	729	452	645	435	622	415	603	388	605	366	621	353	645	351	673	364	696
719	648	774	551	821	650	872	562	921	668	561	558	545	534	526	514	498	511	474	525	460	549	459	577	469	603
769	694	810	603	882	694	923	605	977	714	596	611	576	592	562	568	538	559	514	570	498	591	494	618	499	645
683	702	732	613	799	699	828	613	886	712	525	618	508	603	493	585	471	579	449	586	435	604	431	627	434	650
1173	1257	1263	1020	1448	1248	1545	1023	1711	1263	718	1069	664	1022	625	955	563	930	499	971	456	1034	442	1110	469	1180
1229	1069	1349	854	1523	1071	1614	863	1760	1105	790	873	742	825	696	774	625	782	559	810	509	860	496	930	526	992
1181	1141	1280	885	1450	1125	1535	892	1673	1146	725	936	706	857	677	785	606	778	551	836	489	887	461	962	478	1039
541	549	629	391	739	557	810	407	890	575	295	385	254	359	219	324	174	306	127	323	92	356	81	404	101	449
533	606	617	442	719	602	801	449	908	643	273	472	237	440	198	413	150	405	106	419	79	458	84	505	103	548
509	611	591	463	697	614	775	474	869	639	248	484	211	458	177	429	133	418	89	428	60	461	55	505	71	547
533	623	627	470	715	625	797	483	888	659	269	495	235	457	204	415	154	401	107	428	63	462	39	510	64	555
521	585	591	438	693	587	763	454	857	608	248	448	222	408	200	364	158	361	113	382	79	418	63	463	77	510
538	634	625	490	738	652	809	504	896	681	267	480	234	447	202	413	155	407	111	423	82	459	73	505	89	547
534	634	633	474	732	632	824	479	910	653	280	489	250	454	218	416	167	413	122	436	92	478	82	528	101	576
526	600	607	433	705	600	782	448	880	625	231	463	235	408	206	358	153	336	101	362	57	402	32	456	42	514
496	698	583	560	682	698	753	572	826	721	250	558	210	540	173	516	129	514	93	540	68	576	65	620	93	654
501	649	595	504	691	654	771	509	865	688	229	499	199	475	170	443	126	436	90	460	67	497	64	540	82	580
544	608	634	452	730	612	815	456	901	635	266	462	227	426	193	380	143	365	96	396	61	441	57	496	82	547
556	672	631	510	747	671	814	527	907	711	254	528	226	492	202	455	157	447	118	472	86	507	78	553	91	596
505	663	582	500	675	677	760	517	835	688	220	515	190	477	167	430	120	417	76	444	45	485	30	534	45	582
513	520	589	373	685	529	757	387	843	544	220	377	195	339	171	296	123	299	75	312	41	346	33	394	47	439
509	610	581	459	688	608	769	461	856	630	234	463	201	432	170	398	124	392	81	410	48	443	36	488	49	533
566	536	630	374	730	538	797	396	887	562	288	357	255	329	222	303	179	303	145	328	128	368	129	410	148	449
558	512	642	365	729	517	822	381	899	558	277	341	241	310	206	276	160	282	121	313	100	356	104	406	133	445
527	628	596	446	709	616	780	462	892	641	233	496	201	458	182	409	140	382	93	403	59	444	47	496	59	549
540	538	613	403	713	541	786	407	873	569	280	408	246	385	216	357	176	356	140	376	121	413	110	454	128	489
549	693	614	552	710	702	799	569	880	737	257	548	237	504	208	466	161	473	124	507	81	529	64	575	86	619
464	636	562	506	643	644	727	511	798	674	217	507	195	472	175	436	136	426	101	450	75	484	57	522	64	562
513	607	584	459	686	605	766	470	839	633	226	472	206	429	182	390	137	377	94	394	65	431	59	478	72	524

502	636	611	493	709	645	786	505	883	659	247	487	212	461	178	430	134	429	98	457	71	494	60	538	80	578
492	665	538	528	646	665	691	532	768	662	216	532	185	516	153	500	118	503	89	524	75	557	75	593	94	624
520	523	583	370	683	517	754	371	840	529	255	378	218	357	186	329	143	324	107	346	87	385	84	429	112	462
526	532	592	371	701	525	764	378	842	541	256	398	222	371	188	348	145	338	105	355	89	396	88	438	111	475
477	471	561	334	670	462	724	338	822	488	210	362	182	337	153	309	112	304	79	328	59	363	64	403	86	438
511	608	599	448	707	616	776	459	875	639	222	477	189	441	162	398	124	375	81	398	52	437	45	487	55	536
517	551	595	397	688	566	779	418	860	603	247	382	219	339	191	295	144	284	97	306	58	343	47	392	56	444
507	491	586	334	680	501	751	341	842	508	229	326	190	301	152	275	107	278	67	299	49	341	52	386	76	424
471	475	557	327	642	478	726	349	795	519	226	323	188	301	158	265	119	243	79	257	50	292	39	337	55	380
508	407	583	277	688	417	738	291	822	436	258	272	234	238	200	215	159	222	124	243	104	279	105	321	123	357
484	502	596	354	673	509	771	383	828	541	230	354	203	321	176	287	135	278	95	293	74	329	67	371	76	411
487	497	575	354	669	512	736	380	815	540	234	331	217	290	193	252	150	244	109	259	75	288	55	328	64	370
423	367	482	241	554	369	624	251	692	377	196	249	169	220	147	188	110	190	80	216	66	252	69	293	86	328
447	392	508	276	585	385	637	285	702	407	229	276	199	257	171	232	136	229	103	247	80	276	71	312	86	345
449	469	512	346	588	473	644	359	713	494	228	360	205	326	179	297	139	295	102	311	75	341	70	380	88	416
532	462	589	320	672	454	724	324	789	465	308	335	282	301	255	269	215	273	177	293	156	329	158	370	181	406
443	600	501	480	576	600	632	492	691	616	230	481	204	459	176	441	144	437	112	449	95	478	91	512	112	540
458	408	520	297	585	406	627	303	687	421	265	297	239	278	210	262	179	265	154	282	140	311	139	343	150	374
475	633	515	493	614	618	649	492	750	620	249	532	221	509	194	483	159	482	131	505	115	538	115	575	134	608
411	495	461	379	556	491	598	381	661	485	212	401	183	390	159	368	128	357	97	368	74	392	70	424	86	452
427	408	472	298	532	417	597	315	659	436	193	317	171	289	149	258	119	243	87	262	67	283	51	316	58	351
454	600	532	470	621	602	690	478	757	618	243	485	217	456	192	427	154	426	120	443	97	474	91	511	111	542
474	577	546	431	635	578	708	443	791	612	241	421	212	385	187	345	148	327	104	342	76	380	63	425	72	472
502	574	556	414	660	569	720	421	806	588	252	432	219	396	192	354	146	344	102	367	69	403	56	450	69	497
463	470	547	332	634	476	700	341	777	498	216	335	193	308	171	275	131	269	96	289	70	319	67	358	89	390
410	386	473	258	555	379	619	268	679	401	176	253	150	231	124	208	90	208	59	226	40	255	36	291	55	321
512	499	587	351	678	482	746	343	842	489	226	408	203	372	175	338	133	351	99	377	76	414	75	456	100	490
498	498	538	378	625	480	666	369	744	479	295	425	267	403	249	371	216	353	179	357	152	383	143	419	152	455
491	566	559	407	670	553	740	410	836	583	217	447	183	407	148	371	96	363	51	389	23	432	27	484	48	532
491	474	558	342	658	464	708	342	803	470	233	385	208	351	175	329	136	332	107	362	90	400	86	441	113	472
481	463	541	312	636	455	705	323	774	469	217	340	193	301	172	256	128	251	84	270	58	310	53	359	71	402
528	542	558	370	691	506	736	340	845	505	254	442	212	417	175	383	127	376	88	401	68	447	70	497	90	542
516	568	562	416	678	553	729	413	835	560	238	479	205	453	174	425	133	415	95	436	78	475	75	518	92	557
540	597	599	451	715	588	768	445	869	586	242	514	207	485	174	455	129	453	93	482	67	520	62	567	93	600
548	611	595	447	707	593	765	434	874	593	266	501	228	471	195	436	150	442	115	477	95	521	93	569	116	612
536	526	594	360	713	509	765	346	885	514	234	413	198	379	159	352	113	354	77	384	54	426	57	473	89	507
497	561	552	414	664	542	709	406	799	538	222	470	181	452	146	423	104	415	68	441	54	483	54	528	71	568
492	649	557	497	667	630	724	483	831	627	245	538	208	512	174	482	129	472	89	490	67	530	61	576	73	620
521	585	574	433	682	551	740	420	838	562	239	505	207	477	182	440	144	428	111	457	81	490	78	535	97	575
525	555	578	414	691	533	739	390	843	512	253	458	217	442	183	420	146	410	112	431	97	469	100	509	114	547
765	751	830	605	915	742	967	630	1028	763	548	627	521	610	492	591	459	596	431	617	416	648	422	682	445	708
688	741	738	604	832	736	882	608	973	756	456	626	428	611	401	594	369	595	345	616	333	647	346	675	367	700
781	713	840	599	927	718	979	604	1048	734	559	602	534	575	511	545	479	536	452	562	435	595	437	632	457	662
715	735	785	613	874	739	931	617	1008	759	484	611	458	588	435	561	399	560	377	589	365	623	370	659	392	687
776	686	804	565	899	669	930	555	1021	680	553	587	529	561	509	533	480	528	462	558	442	587	430	620	441	653
818	674	864	553	954	659	995	547	1078	674	599	558	573	533	545	512	511	512	483	534	471	568	472	604	493	633
756	668	813	528	899	662	967	535	1038	679	505	551	484	519	451	500	415	505	388	531	373	565	378	603	403	630
811	740	868	598	961	736	1017	604	1100	748	571	623	548	588	520	560	480	559	444	581	422	615	418	655	434	693
745	646	801	518	889	642	945	517	1018	655	526	540	504	505	481	471	450	450	419	471	420	510	400	541	384	574
708	730	764	591	856	717	902	588	981	726	496	616	463	601	435	577	402	568	371	588	348	616	347	653	368	682
779	733	830	599	924	724	972	597	1055	744	538	614	518	584	497	556	463	555	432	571	417	604	414	638	431	670
699	719	756	594	847	722	896	598	975	733	485	600	459	578	432	557	400	560	371	579	358	609	359	643	380	669
726	721	795	595	889	719	944	601	1036	741	501	608	471	590	444	569	409	569	384	594	366	623	366	658	385	687
724	726	784	593	871	724	920	598	992	740	513	596	485	583	458	565	425	564	396	581	384	611	388	644	408	672
727	716	795	583	880	708	937	589	1011	725	495	594	467	570	441	546	405	545	376	567	356	598	352	634	372	664
755	791	807	660	896	793	935	667	1010	802	525	678	513	642	489	614	452	608	420	629	401	662	400	700	416	734
686	708	751	575	836	701	898	580	959	723	457	602	449	564	436	529	408	510	378	535	351	562	336	597	340	636
789	696	858	563	961	693	1005	566	1083	704	543	594	517	569	494	541	459	540	430	562	412	593	413	630	433	661
767	742	829	606	919	740	976	606	1070	750	529	620	503	596	476	578	441	581	414	602	400	632	405	666	424	696
734	741	792	602	881	727	931	599	1014	737	485	633	461	605	434	580	399	593	369	614	353	648	359	685	381	715
782	726	851	576	939	714	996	579	1074	733	526	600	501	567	468	535	423	546	388	576	368	616	380	658	415	686
722	722	769	602	859	725	901	607	982	734	506	591	478	568	449	550	417	555	390	579	374	608	376	644	396	674
757	692	839	560	915	691	979	562	1046	703	553	559	529	529	497	508	459	518	425	538	401	569	400	608	420	642
854	801	910	674	1004	800	1063	676	1139	818	617	673	587	647	570	607	533	608	495	629	466	659	459	701	478	739
727	708	793	578	871	707	939	588	1009	723	470	601	450	565	425	533	385	538	350	560	330	594	335	635	358	667
811	747	887	610	956	750	1029	630	1084	793	578	622	568	572	537	533	499	559	461	590	422	622	410	669	444	707
790	789	862	644	937	784	1008	647	1075	787	577	666	548	637	521	606	480	609	444	631	410	657	400	695	416	735
689	819	772	679	840	821	909	697	973	849	470	694	450	662	422	639	384	636	351	652	330	683	330	719	344	755
703	764	757	653	832	760	890	654	961	780	485	642	456	627	428	613	399	622	374	643	359	671	362	703	389	719
1186	781	1227	548	1448	794	1558	521	1731	824	649	576	639	485	596	412	506	434	427	480	367	549	351	636	394	716
1046	1181	1164	955	1333	1176	1446	958	1612	1219	601	969	546	941	490	914	425	927	372	968	337	1023	328	1090	363	1143
1031	1024	1134	771	1320	1014	1413	780	1569	1031	573	802	519	780	473	736	420	707	375	744	341	798	327	857	356	911
952	1096	1055	828	1233	1084	1323	829	1484	1116	476	869	429	806	389	737	313	739	278	810	225	870	209	947	244	1017
1065	960	1210	642	1416	949	1545	661	1744	983	510	693	493	609	415	589	335	609	264	658	242	739	265	821	328	878
1049	1162	1159	847	1384	1166	1482	853	1670	1209	509	850	436	813	366	767	286	782	208	818	166	888	181	972	237	1035
1053	1213	1249	897	1410	1217	1573	898	1728	1207	554	897	502	832	432	783	347	798	279	849	229	919	220	1001	265	1073
1103	1007	1219	706	1454	1023	1545	715	1775	1046	514	714	469	638	391	606	306	626	239	679	223	766	251	848	306	917
920	1150	1030	851	1255	1144	1355	872	1550	1178	381	872	325	831	263	804	196	818	148	867	130	936	130	1006	174	1059
1022	1043	1172	712	1397	1046	1536	728	1699	1071	484	734	411	692	339	644	254	639	181	683	137	758	143	844	196	913
1080	1119	1239	812	1419	1102	1550	799	1757	1134	522	831	472	742	396	675	308	700	245	780	170	839	183	935	252	1011
1030	974	1188	711	1367	969	1511	714	1674	1004	557	736	489	708	427	659	349	651	292	704	259	777	232	850	273	916
1094	1072	1251	768	1432	1072	1608	788	1741	1119	490	793	464	709	426	633	330	658	249	717	200	802	188	901	225	992
980	1134	1087	827	1301	1109	1418	835	1595	1143	438	870	379	815	313	775	234	786	187	848	150	920	147	999	194	1063

1134	1027	1265	715	1501	1012	1588	689	1784	1023	585	769	527	707	469	641	385	620	317	671	283	753	293	837	345	907
1046	921	1184	550	1445	892	1584	565	1791	926	403	602	323	545	244	489	148	504	87	578	43	665	46	759	110	834
1067	800	1218	483	1426	797	1588	492	1756	834	416	534	385	443	363	347	260	366	171	430	107	518	93	626	123	729
1026	935	1160	633	1353	910	1505	645	1672	940	431	686	397	594	327	531	238	561	168	627	118	709	122	805	177	879
973	987	1075	700	1297	953	1406	695	1601	980	410	739	350	697	287	662	216	674	165	720	147	790	170	859	219	914
994	1000	1092	728	1308	980	1421	728	1605	1008	492	771	424	753	357	718	286	727	229	772	191	835	206	905	261	957
1139	1040	1213	726	1468	1042	1543	737	1751	1051	482	805	452	735	411	666	335	658	281	718	249	793	254	874	299	941
1133	1098	1292	797	1469	1090	1608	824	1799	1125	595	787	520	752	446	706	361	714	298	772	249	844	225	924	279	992
1110	1014	1262	750	1481	1040	1601	752	1811	1087	598	750	520	697	460	622	372	636	287	679	252	767	263	863	335	928
1084	1141	1253	839	1434	1140	1592	858	1746	1174	569	869	495	816	428	754	348	730	284	793	229	865	216	951	265	1022
1086	1101	1220	805	1416	1101	1526	834	1685	1129	600	813	528	770	456	718	373	733	300	784	266	863	295	946	353	1006
1104	1037	1224	782	1400	1032	1550	798	1690	1067	580	850	544	794	493	755	428	758	387	811	363	874	377	938	426	984
1047	889	1121	631	1339	850	1410	621	1597	860	540	655	483	633	421	615	356	622	298	653	280	716	291	779	334	827
1070	1385	1199	1096	1366	1356	1485	1071	1650	1351	585	1159	535	1095	467	1063	394	1090	332	1145	296	1219	306	1297	360	1357
1088	1132	1228	810	1461	1090	1564	797	1759	1093	555	884	488	849	417	806	337	804	272	859	222	925	218	1008	261	1079
1096	1228	1210	950	1447	1222	1555	964	1735	1267	564	988	506	952	458	892	388	889	336	945	302	1012	309	1085	359	1143
1026	1085	1192	773	1382	1088	1534	790	1698	1108	503	790	436	735	375	674	287	670	210	714	161	788	156	875	202	951
1112	1045	1253	763	1477	1043	1595	781	1793	1079	624	734	552	699	490	645	409	647	332	676	277	735	257	813	293	885
1010	1141	1160	811	1389	1148	1534	847	1711	1204	381	856	345	760	287	679	186	695	109	767	80	869	129	961	205	1037
1094	1217	1231	937	1452	1217	1597	959	1785	1257	572	948	523	893	467	843	397	849	333	887	296	949	284	1022	311	1091
1063	1153	1221	858	1439	1166	1542	892	1779	1193	553	860	494	817	429	784	356	788	295	826	252	885	254	958	294	1017
1039	814	1181	492	1386	805	1543	502	1703	831	521	510	464	454	409	382	320	400	247	453	191	527	173	616	222	693
1018	832	1129	544	1371	824	1474	549	1711	847	447	565	405	504	355	453	290	493	232	547	187	612	186	688	233	750
1038	832	1194	533	1384	839	1518	552	1703	874	505	499	436	458	360	441	283	464	224	516	181	584	189	661	253	710
1084	655	1199	362	1423	645	1556	391	1730	692	527	399	494	331	467	250	399	254	331	306	271	367	257	451	313	515
532	576	597	445	666	574	737	452	802	607	311	457	284	425	272	383	233	367	194	385	165	418	149	460	153	506
526	610	568	481	663	605	707	478	804	632	282	537	264	508	245	478	212	467	182	487	163	516	162	552	179	584
494	515	562	370	655	509	720	378	808	524	249	396	216	380	184	360	150	352	119	373	101	404	99	441	119	473
577	614	626	487	713	602	761	484	847	601	353	515	322	506	296	485	263	480	236	501	224	532	225	565	245	593
465	502	511	363	594	492	659	364	740	505	212	398	199	362	178	326	141	309	107	329	82	361	75	401	89	439
486	625	544	492	627	617	696	486	784	631	247	519	214	498	183	473	145	471	112	492	91	526	92	565	114	598
480	451	541	297	622	434	693	300	778	441	253	346	216	328	181	307	140	305	107	330	88	366	87	407	109	441
476	479	536	347	634	477	674	353	757	491	247	357	217	335	197	300	161	295	131	320	111	354	105	393	123	426
507	607	561	467	657	603	719	470	807	620	252	498	231	466	206	433	168	414	130	431	104	464	97	505	113	544
555	586	599	460	706	572	756	463	840	594	323	480	294	461	266	438	231	435	200	451	180	479	177	515	191	547
536	668	603	529	689	659	762	520	840	657	296	567	266	541	233	520	194	521	162	545	143	580	135	620	156	652
501	593	558	467	650	587	700	478	788	605	270	485	238	472	211	450	176	447	148	467	128	495	128	530	148	558
545	520	597	380	689	515	739	378	834	529	290	423	259	403	234	370	194	364	161	386	142	422	143	463	163	497
489	589	548	469	642	582	698	477	779	615	241	481	210	468	180	454	146	457	118	477	101	507	105	541	125	568
476	476	523	347	614	473	670	349	776	478	252	382	223	372	199	346	170	331	139	342	127	374	124	409	139	439
450	504	528	378	617	507	681	396	753	538	224	391	190	376	162	349	123	344	87	358	64	388	58	425	74	459
511	531	573	389	672	523	732	387	818	529	260	420	224	394	189	364	147	372	118	407	101	448	99	493	121	532
426	549	497	415	581	562	642	427	719	583	193	398	165	369	144	330	112	304	72	320	47	355	37	398	47	440
512	509	561	379	643	521	703	394	786	538	276	372	247	350	222	320	186	310	150	324	127	354	120	391	134	426
542	531	614	385	719	525	762	402	842	552	276	416	253	374	230	331	185	341	147	370	120	409	115	456	140	497
460	531	514	394	607	527	666	396	751	546	210	420	186	396	167	366	137	350	103	351	89	381	90	416	105	448
471	538	534	400	640	527	682	406	767	562	218	430	195	400	174	369	138	361	109	382	90	415	89	453	111	484
782	735	848	582	955	741	1021	594	1126	738	503	561	467	552	432	542	398	547	374	573	364	607	372	642	395	670
708	729	770	586	876	727	944	596	1028	722	412	566	381	562	349	558	318	569	297	593	296	626	304	659	328	681
823	774	888	629	992	774	1059	641	1142	773	526	609	495	599	463	595	435	607	417	632	415	664	425	695	445	719
777	806	862	645	961	808	1037	649	1179	805	459	643	436	611	402	594	365	602	333	625	316	660	319	698	343	729
702	741	764	606	902	733	950	594	1080	725	390	605	362	587	335	572	304	582	282	606	278	636	290	665	308	692
743	753	817	619	924	770	986	614	1080	770	459	602	429	583	403	558	368	547	334	559	312	588	304	623	316	657
697	752	782	598	887	756	981	611	1069	760	439	560	402	549	365	538	329	543	297	565	270	592	257	628	276	661
772	783	835	613	972	775	1037	614	1160	783	432	618	400	599	366	583	331	586	312	616	309	653	316	688	344	713
770	804	855	657	952	795	1086	666	1135	791	449	603	414	609	377	605	342	612	315	637	300	670	308	704	332	731
744	688	818	550	913	698	997	557	1075	706	426	554	395	530	364	503	326	506	296	533	278	569	278	610	305	641
822	773	881	623	988	779	1062	631	1137	789	497	604	469	571	437	540	392	546	355	572	334	612	343	657	369	694
1259	1203	1365	988	1519	1211	1610	982	1747	1202	825	966	778	956	734	940	691	957	671	998	663	1046	672	1092	703	1128
1316	1265	1440	1041	1551	1272	1676	1062	1808	1274	898	1037	857	1008	814	983	763	992	734	1035	721	1086	724	1137	750	1181
1296	1176	1348	956	1510	1179	1579	959	1703	1168	806	983	763	962	715	951	667	954	637	986	628	1033	643	1075	680	1105
1188	1208	1296	980	1449	1205	1526	985	1685	1189	726	1009	689	1006	651	993	614	1003	601	1039	600	1079	610	1118	636	1146
1266	1191	1359	974	1497	1179	1587	967	1751	1146	835	985	792	976	751	962	708	968	675	996	662	1036	680	1074	715	1101
1325	1069	1433	832	1594	1060	1686	828	1840	1047	884	841	840	813	796	788	761	823	737	869	725	919	739	968	774	1007
1272	1178	1367	963	1509	1172	1608	961	1756	1158	835	959	793	941	751	925	705	929	671	957	656	999	674	1038	710	1065
703	739	753	618	842	734	895	614	978	735	444	614	418	621	391	618	365	627	346	645	340	671	348	696	369	713
772	761	829	625	917	758	973	622	1054	762	488	608	456	605	424	597	394	604	374	629	378	662	392	691	410	717
729	713	821	577	893	721	967	587	1051	724	483	547	458	554	431	554	406	564	392	586	383	610	383	637	399	659
710	764	768	640	854	768	906	637	983	766	449	604	424	609	400	613	381	628	371	651	370	676	380	699	400	714
688	739	767	606	840	736	922	610	985	745	435	593	409	574	383	556	353	564	327	583	308	608	305	639	322	666
787	773	834	642	934	753	984	630	1059	754	554	658	526	645	501	624	472	618	449	636	436	664	432	695	445	723
765	738	827	611	906	741	963	623	1033	755	512	593	482	593	453	584	424	584	410	609	406	638	411	667	430	690
721	721	790	593	880	718	935	600	1011	733	482	579	449	572	419	557	386	552	358	567	344	597	346	630	361	659
743	775	783	604	889	771	953	617	1061	783	406	596	376	570	340	556	301	559	274	585	263	622	274	659	299	688
763	789	834	624	952	784	1018	630	1115	773	456	588	422	583	389	569	356	570	332	596	319	628	319	663	339	692
701	678	759	547	864	675	932	545	1029	687	389	545	356	539	324	524	292	524	268	546	258	579	271	611	297	634
756	689	821	546	918	679	968	553	1040	677	491	533	460	516	425	507	390	511	362	532	344	562	338	598	359	627
824	769	877	620	995	767	1028	618	1153	756	547	584	513	570	478	558	443	565	417	587	403	621	407	655	430	682
825	716	898	573	999	721	1087	585	1166	733	526	574	493	566	458	553	422	561	397	585	388	620	400	654	428	677
786	783	867	628	961	782	1034	641	1121	798	491	614	456	600	420	591	387	603	363	631	350	666	353	703	381	725

x25	y25	x26	y26	x27	y27	x28	y28	x29	y29	x30	y30	PrCoor_x1	PrCoor_y1	PrCoor_x2	PrCoor_y2	PrCoor_x3	PrCoor_y3	PrCoor_x4	PrCoor_y4	PrCoor_x5	PrCoor_y5	PrCoor_x6	PrCoor_y6	PrCoor_x7	PrCoor_y7
447	1364	502	1394	562	1409	620	1398	645	1342	699	1314	3.41E-01	-1.22E-01	2.88E-01	1.42E-02	2.42E-01	-1.31E-01	1.89E-01	2.46E-03	1.39E-01	-1.39E-01	7.83E-02	-3.83E-03	1.85E-02	-1.36E-01
538	1222	589	1256	651	1268	713	1276	746	1230	795	1194	3.48E-01	-1.20E-01	2.99E-01	1.26E-02	2.44E-01	-1.28E-01	1.87E-01	-7.08E-04	1.40E-01	-1.27E-01	5.94E-02	1.34E-03	2.36E-02	-1.31E-01
445	1177	511	1212	580	1231	646	1202	701	1152	766	1116	3.39E-01	-1.22E-01	2.99E-01	1.29E-02	2.37E-01	-1.28E-01	1.90E-01	4.59E-03	1.28E-01	-1.24E-01	6.73E-02	9.14E-03	1.60E-02	-1.16E-01
585	1182	644	1216	708	1245	775	1240	825	1193	886	1162	3.35E-01	-1.18E-01	3.01E-01	1.28E-02	2.35E-01	-1.24E-01	1.93E-01	-5.37E-05	1.24E-01	-1.22E-01	7.32E-02	9.03E-04	1.21E-02	-1.21E-01
565	1249	640	1270	717	1272	777	1225	818	1159	873	1111	3.39E-01	-1.13E-01	2.94E-01	1.72E-02	2.34E-01	-1.22E-01	1.94E-01	1.94E-03	1.27E-01	-1.26E-01	7.45E-02	1.64E-03	2.00E-02	-1.24E-01
564	1383	635	1409	710	1421	771	1411	799	1349	864	1311	3.51E-01	-1.10E-01	2.89E-01	9.48E-03	2.43E-01	-1.29E-01	1.84E-01	-4.69E-03	1.35E-01	-1.38E-01	6.36E-02	-3.04E-03	1.60E-02	-1.32E-01
490	1238	548	1257	607	1271	667	1274	701	1229	750	1196	3.47E-01	-1.15E-01	3.01E-01	2.64E-02	2.37E-01	-1.23E-01	1.86E-01	7.81E-03	1.31E-01	-1.27E-01	6.80E-02	3.64E-03	2.47E-02	-1.26E-01
541	1195	613	1214	685	1215	748	1175	803	1126	868	1093	3.47E-01	-1.08E-01	2.99E-01	9.77E-03	2.39E-01	-1.26E-01	1.88E-01	-8.02E-04	1.26E-01	-1.23E-01	6.95E-02	-4.82E-03	1.61E-02	-1.23E-01
594	1167	655	1197	716	1225	778	1235	813	1181	871	1151	3.42E-01	-1.27E-01	2.94E-01	7.47E-03	2.42E-01	-1.25E-01	1.85E-01	2.47E-03	1.31E-01	-1.34E-01	6.76E-02	5.25E-03	1.78E-02	-1.25E-01
546	1230	612	1254	684	1255	754	1245	806	1200	873	1174	3.32E-01	-1.22E-01	2.95E-01	1.61E-02	2.35E-01	-1.25E-01	1.92E-01	3.96E-03	1.27E-01	-1.27E-01	6.96E-02	4.61E-03	1.64E-02	-1.24E-01
384	1344	446	1360	510	1357	564	1325	586	1268	644	1242	3.28E-01	-1.13E-01	3.13E-01	1.19E-02	2.31E-01	-1.24E-01	2.02E-01	7.87E-03	1.19E-01	-1.23E-01	6.91E-02	1.64E-03	1.48E-03	-1.18E-01
523	1233	585	1247	644	1227	697	1190	751	1157	815	1149	3.57E-01	-1.21E-01	2.93E-01	9.41E-03	2.43E-01	-1.32E-01	1.93E-01	2.88E-03	1.39E-01	-1.32E-01	7.10E-02	-1.97E-03	3.06E-02	-1.29E-01
510	1122	562	1138	618	1137	669	1115	712	1078	762	1054	3.67E-01	-1.16E-01	3.10E-01	1.12E-02	2.62E-01	-1.35E-01	1.92E-01	2.50E-04	1.40E-01	-1.39E-01	5.98E-02	-4.74E-03	1.25E-02	-1.29E-01
488	1242	546	1272	609	1287	671	1272	722	1232	773	1191	3.44E-01	-1.20E-01	3.00E-01	1.08E-02	2.45E-01	-1.34E-01	1.90E-01	-1.45E-03	1.32E-01	-1.35E-01	6.59E-02	8.88E-04	1.76E-02	-1.32E-01
513	1297	586	1326	660	1356	737	1353	746	1283	788	1224	3.48E-01	-1.16E-01	2.93E-01	1.28E-02	2.37E-01	-1.28E-01	1.94E-01	-1.47E-03	1.26E-01	-1.28E-01	6.38E-02	2.42E-03	1.34E-02	-1.21E-01
501	1194	553	1236	614	1266	679	1281	720	1242	742	1182	3.42E-01	-1.21E-01	2.91E-01	1.16E-02	2.40E-01	-1.25E-01	1.88E-01	2.95E-03	1.36E-01	-1.33E-01	6.77E-02	-6.04E-04	2.02E-02	-1.30E-01
519	1275	581	1303	644	1325	705	1308	743	1253	802	1220	3.33E-01	-1.29E-01	2.92E-01	8.68E-03	2.31E-01	-1.27E-01	1.87E-01	5.61E-03	1.28E-01	-1.33E-01	7.24E-02	1.24E-03	1.51E-02	-1.29E-01
592	1312	644	1339	702	1340	745	1312	769	1258	813	1220	3.59E-01	-1.05E-01	3.01E-01	1.63E-02	2.49E-01	-1.19E-01	1.92E-01	-4.29E-03	1.30E-01	-1.31E-01	6.93E-02	-6.84E-03	2.53E-03	-1.29E-01
508	1179	575	1220	648	1242	725	1235	763	1170	822	1125	3.41E-01	-1.16E-01	2.97E-01	2.33E-03	2.41E-01	-1.29E-01	1.91E-01	1.09E-03	1.24E-01	-1.24E-01	6.42E-02	5.78E-03	1.17E-02	-1.20E-01
453	1250	528	1265	606	1267	682	1259	688	1182	744	1136	3.42E-01	-1.14E-01	2.90E-01	2.16E-02	2.41E-01	-1.30E-01	1.81E-01	-4.36E-04	1.26E-01	-1.37E-01	7.29E-02	-5.31E-03	1.73E-02	-1.38E-01
483	1213	552	1245	627	1268	702	1286	742	1225	811	1191	3.33E-01	-1.31E-01	2.99E-01	7.97E-03	2.31E-01	-1.30E-01	1.86E-01	8.21E-03	1.25E-01	-1.26E-01	6.84E-02	1.28E-02	7.96E-03	-1.23E-01
605	1214	677	1227	750	1234	819	1214	839	1143	893	1102	3.48E-01	-1.16E-01	2.96E-01	2.10E-02	2.32E-01	-1.20E-01	1.80E-01	6.61E-03	1.28E-01	-1.21E-01	7.40E-02	5.49E-04	1.47E-02	-1.18E-01
671	1196	734	1220	800	1235	856	1206	899	1154	964	1136	3.35E-01	-1.19E-01	3.02E-01	1.90E-02	2.33E-01	-1.22E-01	1.95E-01	4.34E-03	1.21E-01	-1.29E-01	7.53E-02	2.78E-03	-6.41E-03	-1.21E-01
497	1328	558	1352	621	1369	685	1370	704	1316	742	1264	3.61E-01	-1.12E-01	3.04E-01	2.07E-02	2.44E-01	-1.28E-01	1.92E-01	2.58E-03	1.18E-01	-1.35E-01	6.48E-02	1.78E-03	1.34E-02	-1.29E-01
480	1299	551	1319	624	1329	697	1330	742	1275	791	1224	3.47E-01	-1.11E-01	2.97E-01	1.32E-02	2.42E-01	-1.21E-01	1.96E-01	4.60E-04	1.34E-01	-1.22E-01	7.26E-02	-1.87E-03	1.08E-02	-1.18E-01
531	1241	597	1266	668	1278	728	1246	776	1193	839	1162	3.49E-01	-1.10E-01	2.99E-01	7.06E-03	2.50E-01	-1.24E-01	1.86E-01	-1.43E-03	1.33E-01	-1.23E-01	6.12E-02	-5.07E-03	1.60E-02	-1.20E-01
587	1256	642	1283	702	1292	742	1257	759	1200	808	1167	3.46E-01	-1.16E-01	3.08E-01	1.73E-02	2.45E-01	-1.19E-01	1.94E-01	-2.64E-03	1.36E-01	-1.30E-01	6.48E-02	-3.55E-03	2.31E-02	-1.30E-01
636	1262	688	1268	728	1245	762	1205	803	1172	853	1154	3.63E-01	-9.54E-02	3.05E-01	2.54E-02	2.60E-01	-1.13E-01	1.82E-01	-1.97E-03	1.42E-01	-1.27E-01	6.65E-02	-8.97E-03	1.72E-02	-1.30E-01
479	1255	540	1275	603	1280	653	1239	704	1202	766	1185	3.44E-01	-1.06E-01	3.01E-01	1.94E-02	2.41E-01	-1.24E-01	1.94E-01	3.01E-04	1.33E-01	-1.34E-01	6.78E-02	-2.69E-03	1.01E-02	-1.29E-01
472	1261	535	1275	598	1266	658	1245	718	1221	782	1213	3.45E-01	-1.16E-01	2.99E-01	6.89E-03	2.38E-01	-1.30E-01	1.98E-01	-1.45E-03	1.35E-01	-1.26E-01	7.21E-02	7.20E-03	2.47E-02	-1.27E-01
539	1076	591	1101	646	1121	701	1112	754	1088	811	1074	3.59E-01	-1.09E-01	2.97E-01	6.24E-03	2.49E-01	-1.32E-01	1.81E-01	-3.14E-04	1.35E-01	-1.33E-01	6.87E-02	4.61E-03	2.13E-02	-1.24E-01
498	1224	567	1246	638	1262	707	1254	752	1199	819	1171	3.56E-01	-1.05E-01	2.98E-01	1.04E-02	2.56E-01	-1.21E-01	1.89E-01	-3.22E-03	1.41E-01	-1.20E-01	5.93E-02	2.89E-04	2.66E-02	-1.21E-01
424	1091	479	1118	537	1138	597	1133	637	1087	690	1056	3.66E-01	-1.06E-01	3.04E-01	6.76E-03	2.61E-01	-1.26E-01	1.90E-01	5.20E-04	1.46E-01	-1.29E-01	5.75E-02	2.83E-03	2.37E-02	-1.24E-01
537	1212	601	1243	672	1256	707	1208	760	1161	826	1134	3.48E-01	-1.18E-01	3.01E-01	1.85E-02	2.38E-01	-1.25E-01	1.93E-01	-6.39E-04	1.31E-01	-1.32E-01	6.64E-02	-4.45E-03	1.04E-02	-1.29E-01
437	1176	499	1204	566	1217	621	1187	660	1131	719	1100	3.47E-01	-1.10E-01	2.96E-01	7.51E-03	2.40E-01	-1.17E-01	1.89E-01	-4.52E-03	1.35E-01	-1.22E-01	7.19E-02	3.77E-04	1.67E-02	-1.22E-01
489	1129	536	1144	582	1147	626	1125	667	1098	715	1085	3.56E-01	-1.11E-01	3.05E-01	1.55E-02	2.50E-01	-1.26E-01	1.87E-01	1.40E-03	1.30E-01	-1.35E-01	5.77E-02	7.63E-03	1.00E-02	-1.27E-01
622	1171	682	1201	746	1223	813	1232	855	1185	906	1145	3.55E-01	-1.11E-01	3.01E-01	1.30E-02	2.51E-01	-1.29E-01	1.86E-01	-8.71E-04	1.38E-01	-1.34E-01	5.49E-02	-1.59E-03	1.54E-02	-1.29E-01
403	1286	463	1300	524	1306	561	1265	614	1232	673	1214	3.56E-01	-1.00E-01	3.08E-01	1.69E-02	2.38E-01	-1.22E-01	1.91E-01	2.43E-03	1.23E-01	-1.25E-01	6.69E-02	-3.09E-03	-2.55E-03	-1.21E-01
456	1176	496	1197	539	1205	571	1173	615	1164	660	1164	3.59E-01	-1.24E-01	3.09E-01	7.65E-03	2.48E-01	-1.37E-01	1.91E-01	4.35E-03	1.28E-01	-1.31E-01	5.69E-02	1.24E-02	6.68E-03	-1.17E-01
410	1354	487	1359	563	1353	618	1301	675	1251	746	1220	3.49E-01	-1.01E-01	2.96E-01	1.55E-02	2.44E-01	-1.22E-01	1.98E-01	-4.43E-03	1.36E-01	-1.28E-01	6.78E-02	-2.31E-03	2.10E-02	-1.24E-01
456	1284	520	1308	589	1316	633	1279	670	1226	731	1196	3.48E-01	-1.10E-01	3.02E-01	1.62E-02	2.47E-01	-1.22E-01	1.87E-01	-2.28E-03	1.35E-01	-1.27E-01	6.37E-02	2.10E-03	1.79E-02	-1.25E-01
508	1318	564	1321	614	1297	665	1272	718	1252	775	1244	3.61E-01	-9.81E-02	3.03E-01	1.75E-02	2.49E-01	-1.23E-01	1.83E-01	2.19E-03	1.35E-01	-1.30E-01	6.44E-02	-3.04E-03	1.90E-02	-1.28E-01
624	1218	694	1227	762	1208	825	1173	874	1122	940	1094	3.47E-01	-1.04E-01	2.94E-01	1.34E-02	2.47E-01	-1.21E-01	1.86E-01	3.75E-03	1.35E-01	-1.33E-01	7.58E-02	-1.99E-03	1.92E-02	-1.30E-01
463	1266	521	1277	570	1248	603	1198	642	1152	691	1118	3.49E-01	-9.50E-02	3.08E-01	9.38E-03	2.44E-01	-1.20E-01	1.95E-01	-3.05E-03	1.26E-01	-1.24E-01	7.41E-02	-7.68E-03	5.34E-03	-1.15E-01
560	1339	610	1355	661	1363	705	1343	747	1312	795	1291	3.56E-01	-1.15E-01	2.90E-01	1.84E-02	2.53E-01	-1.31E-01	1.88E-01	1.73E-03	1.42E-01	-1.34E-01	7.11E-02	-2.16E-03	1.68E-02	-1.32E-01
554	1231	604	1257	661	1263	718	1263	776	1267	833	1262	3.57E-01	-1.14E-01	3.03E-01	1.47E-02	2.43E-01	-1.27E-01	1.84E-01	8.73E-03	1.39E-01	-1.33E-01	5.55E-02	6.97E-03	1.49E-02	-1.31E-01
551	1277	609	1301	670	1314	726	1297	759	1245	810	1209	3.52E-01	-1.05E-01	3.00E-01	9.15E-03	2.38E-01	-1.19E-01	1.90E-01	1.68E-04	1.24E-01	-1.26E-01	6.90E-02	-3.04E-03	1.24E-02	-1.27E-01
470	1255	530	1275	593	1284	652	1266	702	1230	764	1216	3.41E-01	-1.18E-01	2.99E-01	9.35E-03	2.38E-01	-1.27E-01	1.95E-01	3.58E-03	1.33E-01	-1.30E-01	7.94E-02	7.24E-04	1.33E-02	-1.21E-01
535	1277	600	1304	671	1317	718	1276	768	1224	837	1207	3.50E-01	-1.15E-01	3.01E-01	1.28E-02	2.46E-01	-1.24E-01	1.94E-01	-6.86E-04	1.38E-01	-1.29E-01	6.37E-02	1.21E-03	1.36E-02	-1.19E-01
420	1297	477	1326	540	1335	582	1292	634	1255	695	1233	3.67E-01	-1.06E-01	3.02E-01	3.22E-03	2.52E-01	-1.25E-01	1.87E-01	-3.28E-03	1.44E-01	-1.32E-01	6.96E-02	-6.96E-03	2.10E-02	-1.19E-01
465	1187	525	1206	587	1214	645	1197	693	1156	748	1125	3.52E-01	-1.01E-01	2.94E-01	9.28E-03	2.47E-01	-1.30E-01	1.79E-01	-7.08E-03	1.36E-01	-1.40E-01	6.47E-02	-9.76E-03	1.81E-02	-1.34E-01
418	1283	472	1304	529	1312	572	1283	608	1241	662	1220	3.53E-01	-1.15E-01	3.02E-01	7.61E-03	2.42E-01	-1.32E-01	1.89E-01	-4.19E-04	1.34E-01	-1.34E-01	6.38E-02	3.03E-03	1.68E-02	-1.36E-01
485	1274	543	1296	604	1305	661	1287	693	1234	739	1194	3.55E-01	-1.11E-01	3.02E-01	1.17E-02	2.47E-01	-1.18E-01	1.90E-01	-2.70E-03	1.39E-01	-1.27E-01	6.88E-02	-1.14E-02	1.20E-02	-1.22E-01
431	1254	494	1273	560	1263	621	1238	678	1204	740	1182	3.52E-01	-9.62E-02	3.03E-01	2.56E-03	2.50E-01	-1.21E-01	1.97E-01	-1.08E-02	1.40E-01	-1.28E-01	5.90E-02	-1.38E-02	2.32E-02	-1.24E-01
611	1285	680	1312	754	1324	828	1324	838	1261	899	1222	3.59E-01	-1.11E-01	2.96E-01	2.85E-03	2.49E-01	-1.26E-01	1.86E-01	-1.73E-03	1.35E-01	-1.35E-01	6.14E-02	-4.77E-03	2.69E-02	-1.30E-01
512	1291	568	1309	626	1319	677	1299	707	1249	757	1222	3.70E-01	-1.03E-01	2.97E-01	4.06E-03	2.51E-01	-1.22E-01	1.95E-01	-6.33E-03	1.40E-01	-1.32E-01	6.55E-02	-1.22E-02	1.45E-02	-1.30E-01
456	1267	527	1278	599	1282	641	1241	672	1177	735	1145	3.56E-01	-1.01E-01	2.97E-01	4.77E-04	2.46E-01	-1.20E-01	1.91E-01	-3.43E-03	1.33E-01	-1.23E-01	6.55E-02	-7.42E-03	1.55E-02	-1.21E-01
418	1167	495	1188	575	1198	625	1137	678	1077	748	1040	3.59E-01	-1.01E-01	2.96E-01	6.58E-03	2.58E-01	-1.12E-01	1.89E-01	-3.55E-03	1.31E-01	-1.17E-01	6.92E-02	-7.39E-03	1.60E-02	-1.15E-01
455	1125	515	1153	578	1175	644	1184	685	1133	739	1098	3.51E-01	-1.12E-01	3.03E-01	5.62E-03	2.67E-01	-1.17E-01	1.94E-01	3.14E-04	1.35E-01	-1.21E-01	6.10E-02	-8.24E-03	1.50E-02	-1.20E-01
570	1317	646	1341	725	1341	788	1303	834	1240	904	1204	3.46E-01	-1.01E-01	2.92E-01	4.26E-03	2.44E-01	-1.17E-01	1.88E-01	-8.18E-03	1.35E-01	-1.22E-01	7.01E-02	-7.18E-03	2.24E-02	-1.19E-01
633	1268	696	1286	760	1296	809	1274	799	1215	842	1169	3.44E-01	-1.02E-01	3.06E-01	1.09E-02	2.39E-01	-1.08E-01	1.95E-01	4.15E-03	1.22E-01	-1.15E-01	6.41E-02	4.10E-04	6.03E-03	-1.11E-01
510	1218	569	1237	630	1249	691	1251	725	1202	779	1171	3.51E-01	-1.06E-01	3.04E-01	8.71E-03	2.41E-01	-1.17E-01	1.90E-01	5.17E-03	1.30E-01	-1.24E-01	5.60E-02	4.10E-03	5.83E-03	-1.19E-01
552	1300	620	1319	689	1337	755	1324	780	1262	837	1222	3.49E-01	-1.09E-01	2.98E-01	3.62E-03	2.46E-01	-9.94E-02	1.86E-01	-2.97E-04	1.29E-01	-1.27E-01	5.91E-02	6.23E-03	8.76E-03	-1.20E-01
551	1183	598	1198	645	1195	671	1154	715	1134	764	1124	3.48E-01	-1.13E-01	3.01E-01	8.22E-04	2.42E-01	-1.20E-01	1.92E-01	4.64E-03	1.33E-01	-1.23E-01	6.54E-02	1.63E-03	1.47E-02	-1.21E-01
478	1169	537	1188	597	1203	656	1204	683	1152	742	1134	3.45E-01	-1.01E-01	3.05E-01	7.21E-03	2.38E-01	-1.15E-01	1.98E-01	-3.81E-04	1.19E-01	-1.17E-01	5.87E-02	1.03E-02	7.83E-03	-1.15E-01
628	1195	673	1215	720	1223	758	1197	783	1156	828	1136	3.44E-01	-1.19E-01	3.04E-01	5.59E-03	2.39E-01	-1.22E-01	1.94E-01	5.11E-03	1.31E-01	-1.26E-01	6.53E-02	1.47E-03	1.45E-02	-1.25E-01
512	1202	566	1224	623	1234	661	1196	697	1151	751	1129	3.62E-01	-1.05E-01	3.00E-01	8.26E-03	2.50E-01	-1.27E-01	1.91E-01	-1.87E-03	1.28E-01	-1.30E-01	5.94E-02	1.14E-03	1.06E-02	-1.21E-01
677	1142	720	1164	768	1171	805	1144	821	1098	851	1064	3.57E-01	-1.06E-01	3.12E-01	1.34E-02	2.44E-01	-1.15E-01	1.93E-01	1.06E-02	1.21E-01	-1.24E-01	6.81E-02	3.82E-03	-8.48E-03	-1.17E-01
147	243	163	251	180	250	194	239	207	229	225	228	3.25E-01	-1.18E-01	3.01E-01	6.47E-03	2.35E-01	-1.25E-01	1.92E-01	-4.61E-03	1.30E-01	-1.25E-01	7.12E-02	-2.85E-04	1.92E-02	-1.18E-01
71	303	89	308	107	306	120	292	135	284	153	280	3.22E-01	-1.07E-01	3.07E-01	7.33E-03	2.30E-01	-1.17E-01	1.97E-01	-1.60E-03	1.26E-01	-1.22E-01	8.03E-02	-6.41E-03	1.99E-02	-1.23E-01
79	299	92	306	105	301	116	290	128	280	143	278	3.43E-01	-1.06E-01	3.07E-01	1.31E-02	2.49E-01	-1.29E-01	1.90E-01	-4.81E-04	1.28E-01	-1.30E-01	6.49E-02	-6.14E-03	-1.91E-04	-1.25E-01
84	314	102	319	120	323	131	309	145	298	163	296	3.42E-01	-1.06E-01	3.06E-01	8.88E-03	2.47E-01	-1.26E-01	1.85E-01	1.59E-03	1.30E-01	-1.28E-01	6.06E-02	-2.15E-04	7.00E-03	-1.24E-01
116	282	132	291	149	293	159	283	166	267	182	261	3.43E-01	-1.06E-01	3.04E-01	1.42E-02	2.41E-01	-1.23E-01	1.88E-01	-8.47E-05	1.34E-01	-1.29E-01	6.62E-02	-5.66E-03	2.54E-02	-1.23E-01
97	292	115	300	135	301	152	294	162	277	177	264	3.37E-01	-1.11E-01	2.95E-01	1.56E-02	2.38E-01	-1.22E-01	1.88E-01	4.62E-03	1.34E-01	-1.27E-01	7.01E-02	-1.98E-03	2.06E-02	-1.35E-01
94	300	111	304	127	302	141	292	154	283	171	279	3.47E-01	-1.04E-01	2.97E-01	4.53E-03	2.43E-01	-1.23E-01	1.93E-01	-5.86E-03	1.24E-01	-1.27E-01	7.84E-02	-8.21E-03	1.24E-02	-1.23E-01
78	303	100	312	123	319	144	315	151	293	161	273	3.29E-01	-1.17E-01	2.98E-01	1.01E-02	2.31E-01	-1.26E-01	1.87E-01	4.81E-03	1.31E-01	-1.28E-01	6.66E-02	3.85E-03	3.29E-02	-1.22E-01
106	294	125	298	145	294	163	286	183	284	203	286	3.30E-01	-1.06E-01	3.03E-01	5.15E-03	2.34E-01	-1.18E-01	1.95E-01	-2.94E-03	1.33E-01	-1.19E-01	7.40E-02	-1.78E-03	2.83E-02	-1.17E-01
124	293	136	298	146	295	156	286	168	280	180	275	3.46E-01	-1.14E-01	3.08E-01	7.51E-03	2.50E-01	-1.24E-01	1.89E-01	4.25E-03	1.40E-01	-1.33E-01	6.73E-02	1.00E-03	2.25E-02	-1.34E-01
85	295	100	300	115	304	130	304	138	291	152	285	3.49E-01	-1.05E-01	3.12E-01	7.18E-03	2.51E-01	-1.18E-01	1.94E-01	5.06E-04	1.35E-01	-1.21E-01	6.54E-02	1.46E-03	9.97E-03	-1.20E-01

98	277	112	284	128	284	142	276	154	265	169	261	3.66E-01	-8.91E-02	2.95E-01	2.87E-03	2.43E-01	-1.24E-01	1.98E-01	-2.99E-03	1.23E-01	-1.27E-01	7.04E-02	-6.13E-03	1.02E-02	-1.15E-01
81	272	94	280	108	280	121	276	135	272	149	269	3.42E-01	-1.22E-01	3.01E-01	8.58E-03	2.42E-01	-1.33E-01	1.93E-01	-1.07E-03	1.32E-01	-1.35E-01	7.02E-02	3.18E-03	1.13E-02	-1.35E-01
133	277	148	282	164	286	180	283	191	271	207	268	3.42E-01	-1.08E-01	3.08E-01	7.42E-03	2.43E-01	-1.15E-01	1.93E-01	1.06E-02	1.37E-01	-1.21E-01	6.31E-02	3.03E-03	2.54E-02	-1.19E-01
130	270	147	277	166	281	181	275	184	257	200	247	3.45E-01	-1.04E-01	3.04E-01	3.42E-03	2.46E-01	-1.19E-01	1.88E-01	2.02E-03	1.42E-01	-1.24E-01	7.01E-02	-3.24E-03	2.15E-02	-1.26E-01
76	311	91	317	107	318	119	310	124	295	138	287	3.36E-01	-1.14E-01	3.00E-01	1.22E-02	2.42E-01	-1.23E-01	1.97E-01	1.58E-03	1.38E-01	-1.28E-01	7.77E-02	2.26E-03	2.48E-02	-1.22E-01
87	282	105	293	125	297	145	291	155	273	174	264	3.33E-01	-1.10E-01	2.97E-01	3.00E-03	2.35E-01	-1.27E-01	1.95E-01	3.36E-03	1.27E-01	-1.25E-01	7.69E-02	-7.16E-03	1.18E-02	-1.23E-01
98	260	113	269	131	269	149	263	165	256	181	247	3.32E-01	-1.11E-01	3.04E-01	9.32E-03	2.34E-01	-1.22E-01	2.00E-01	3.62E-03	1.27E-01	-1.25E-01	6.78E-02	-4.77E-03	1.84E-02	-1.20E-01
92	279	111	286	131	287	151	280	164	265	183	257	3.36E-01	-1.19E-01	3.08E-01	1.45E-02	2.37E-01	-1.27E-01	2.00E-01	2.53E-04	1.30E-01	-1.31E-01	6.79E-02	1.84E-03	2.00E-02	-1.26E-01
145	265	160	268	175	264	183	251	196	243	211	237	3.40E-01	-1.06E-01	3.06E-01	5.69E-03	2.47E-01	-1.17E-01	1.99E-01	1.30E-03	1.30E-01	-1.27E-01	6.22E-02	-3.88E-03	6.36E-03	-1.20E-01
144	366	166	371	188	373	203	362	199	341	217	328	3.42E-01	-1.01E-01	2.97E-01	9.96E-03	2.44E-01	-1.22E-01	1.95E-01	-2.41E-03	1.29E-01	-1.30E-01	7.12E-02	-4.89E-03	1.97E-02	-1.19E-01
122	266	140	276	159	281	179	284	191	269	205	254	3.32E-01	-1.12E-01	2.95E-01	6.70E-03	2.37E-01	-1.27E-01	1.95E-01	2.26E-03	1.28E-01	-1.33E-01	5.88E-02	3.70E-03	9.81E-03	-1.26E-01
139	283	159	288	179	293	193	287	201	268	218	258	3.48E-01	-1.04E-01	2.91E-01	7.89E-03	2.44E-01	-1.22E-01	1.91E-01	-1.11E-03	1.27E-01	-1.22E-01	6.98E-02	-1.96E-03	1.15E-02	-1.17E-01
92	306	109	312	127	313	139	303	154	293	171	289	3.42E-01	-1.15E-01	3.06E-01	8.28E-03	2.41E-01	-1.20E-01	1.91E-01	-2.51E-03	1.38E-01	-1.16E-01	7.07E-02	-4.08E-03	7.68E-03	-1.10E-01
113	279	129	285	146	289	163	290	172	276	188	271	3.41E-01	-1.04E-01	2.99E-01	6.49E-03	2.48E-01	-1.24E-01	1.92E-01	-2.53E-03	1.30E-01	-1.27E-01	6.83E-02	-2.61E-03	2.02E-02	-1.11E-01
115	263	127	271	140	277	149	269	149	256	161	247	3.44E-01	-1.13E-01	3.06E-01	1.10E-02	2.43E-01	-1.20E-01	1.90E-01	2.98E-03	1.32E-01	-1.31E-01	5.95E-02	1.03E-02	1.91E-02	-1.20E-01
101	322	123	328	144	330	155	312	161	290	179	280	3.41E-01	-9.90E-02	2.97E-01	8.62E-03	2.38E-01	-1.23E-01	1.93E-01	-3.37E-03	1.30E-01	-1.27E-01	7.80E-02	-1.17E-02	2.43E-02	-1.18E-01
97	315	112	320	127	325	142	320	152	309	166	302	3.46E-01	-1.07E-01	2.99E-01	1.43E-02	2.50E-01	-1.32E-01	1.87E-01	2.43E-03	1.32E-01	-1.40E-01	6.02E-02	-1.94E-03	1.67E-02	-1.32E-01
124	253	134	260	146	264	158	266	169	263	178	254	3.47E-01	-1.10E-01	3.01E-01	7.79E-03	2.46E-01	-1.29E-01	1.96E-01	-2.92E-03	1.38E-01	-1.27E-01	6.70E-02	-1.66E-03	1.63E-02	-1.19E-01
99	277	114	283	130	287	141	278	148	264	163	258	3.32E-01	-1.09E-01	3.05E-01	9.34E-03	2.40E-01	-1.19E-01	1.97E-01	-1.34E-04	1.29E-01	-1.22E-01	7.73E-02	-3.68E-03	1.38E-02	-1.20E-01
216	481	239	490	264	494	285	485	300	466	321	453	3.44E-01	-1.11E-01	3.00E-01	1.09E-02	2.46E-01	-1.24E-01	1.81E-01	-3.25E-05	1.37E-01	-1.35E-01	6.21E-02	-4.81E-03	2.92E-02	-1.33E-01
167	454	187	457	210	456	230	445	249	432	272	428	3.54E-01	-1.04E-01	3.00E-01	6.55E-03	2.46E-01	-1.17E-01	1.87E-01	3.16E-03	1.41E-01	-1.22E-01	7.26E-02	7.97E-04	2.13E-02	-1.16E-01
241	540	263	545	287	545	302	531	314	511	334	501	3.46E-01	-1.09E-01	3.06E-01	9.45E-03	2.43E-01	-1.17E-01	1.97E-01	2.56E-04	1.34E-01	-1.20E-01	6.33E-02	2.88E-03	1.18E-02	-1.16E-01
240	472	261	480	282	483	297	471	306	453	326	445	3.57E-01	-1.18E-01	3.08E-01	1.21E-02	2.50E-01	-1.24E-01	1.86E-01	6.54E-03	1.48E-01	-1.31E-01	6.19E-02	-5.01E-03	2.98E-02	-1.26E-01
235	487	256	494	277	499	292	489	301	470	320	461	3.58E-01	-1.03E-01	3.03E-01	1.52E-02	2.54E-01	-1.22E-01	1.86E-01	-1.29E-03	1.35E-01	-1.29E-01	6.23E-02	-2.10E-03	9.95E-03	-1.21E-01
195	479	221	485	247	487	274	490	281	468	290	444	3.50E-01	-9.87E-02	3.01E-01	2.20E-02	2.51E-01	-1.18E-01	1.86E-01	6.48E-03	1.29E-01	-1.22E-01	5.32E-02	5.83E-03	1.38E-02	-1.21E-01
178	555	202	568	229	573	245	556	247	529	263	510	3.36E-01	-9.52E-02	2.98E-01	1.31E-02	2.49E-01	-1.16E-01	1.94E-01	3.43E-03	1.43E-01	-1.24E-01	6.81E-02	7.64E-04	2.90E-02	-1.26E-01
198	488	216	493	236	492	250	481	258	465	276	459	3.56E-01	-1.08E-01	2.99E-01	1.40E-02	2.51E-01	-1.27E-01	1.90E-01	4.21E-04	1.39E-01	-1.35E-01	6.28E-02	-1.34E-03	1.17E-02	-1.31E-01
264	516	284	521	306	523	321	513	332	494	349	483	3.44E-01	-1.13E-01	2.96E-01	5.81E-03	2.42E-01	-1.30E-01	1.86E-01	-2.82E-04	1.32E-01	-1.41E-01	6.60E-02	-1.44E-02	1.42E-02	-1.43E-01
229	488	249	494	270	500	287	492	303	479	323	473	3.44E-01	-1.13E-01	3.00E-01	9.33E-03	2.44E-01	-1.31E-01	1.88E-01	-2.14E-04	1.33E-01	-1.28E-01	6.51E-02	3.98E-03	1.38E-02	-1.23E-01
170	405	190	416	213	422	229	410	244	392	266	386	3.36E-01	-1.12E-01	3.02E-01	3.09E-03	2.42E-01	-1.27E-01	1.89E-01	3.21E-03	1.31E-01	-1.28E-01	6.99E-02	4.42E-04	2.24E-02	-1.17E-01
235	493	261	496	287	496	308	481	325	462	349	452	3.28E-01	-1.04E-01	3.01E-01	8.75E-03	2.40E-01	-1.25E-01	1.95E-01	9.38E-04	1.28E-01	-1.27E-01	6.99E-02	1.99E-03	1.43E-02	-1.28E-01
219	464	239	472	260	471	280	464	292	448	307	433	3.40E-01	-1.14E-01	3.05E-01	1.08E-02	2.42E-01	-1.26E-01	1.90E-01	5.33E-03	1.27E-01	-1.32E-01	6.29E-02	1.78E-03	8.60E-03	-1.29E-01
169	507	193	518	217	525	239	517	252	495	266	474	3.49E-01	-1.07E-01	2.96E-01	1.52E-02	2.52E-01	-1.22E-01	1.86E-01	3.31E-04	1.39E-01	-1.28E-01	6.16E-02	-5.09E-03	2.21E-02	-1.24E-01
231	513	250	519	270	519	277	503	282	483	292	466	3.51E-01	-1.07E-01	3.05E-01	9.77E-03	2.47E-01	-1.22E-01	1.95E-01	-7.21E-04	1.36E-01	-1.26E-01	7.08E-02	-3.10E-03	1.66E-02	-1.29E-01
240	439	264	446	287	451	312	452	318	433	332	415	3.51E-01	-1.14E-01	3.02E-01	1.31E-02	2.45E-01	-1.25E-01	1.87E-01	3.42E-03	1.30E-01	-1.32E-01	6.51E-02	-2.28E-04	1.49E-02	-1.23E-01
210	455	230	460	250	457	263	441	280	430	299	422	3.55E-01	-1.01E-01	3.02E-01	1.60E-02	2.58E-01	-1.19E-01	1.86E-01	3.49E-04	1.42E-01	-1.27E-01	6.38E-02	-7.00E-03	2.11E-02	-1.26E-01
182	524	208	528	234	526	259	520	267	495	289	481	3.51E-01	-1.03E-01	3.00E-01	8.53E-03	2.52E-01	-1.09E-01	1.87E-01	1.28E-03	1.38E-01	-1.25E-01	6.21E-02	-1.46E-03	2.13E-02	-1.20E-01
224	307	243	314	263	315	282	308	302	302	322	299	3.55E-01	-1.02E-01	3.00E-01	6.11E-03	2.58E-01	-1.22E-01	1.83E-01	-5.21E-03	1.37E-01	-1.28E-01	5.57E-02	4.17E-03	2.26E-02	-1.19E-01
224	435	242	439	261	438	275	426	290	414	307	408	3.49E-01	-1.08E-01	3.14E-01	5.83E-03	2.54E-01	-1.18E-01	1.94E-01	4.73E-03	1.31E-01	-1.22E-01	6.62E-02	3.51E-03	1.77E-02	-1.22E-01
258	521	279	529	301	535	323	529	334	509	356	501	3.46E-01	-1.11E-01	2.93E-01	5.27E-03	2.43E-01	-1.27E-01	1.84E-01	-5.28E-04	1.36E-01	-1.30E-01	6.40E-02	2.72E-03	1.54E-02	-1.27E-01
93	421	112	426	132	427	145	416	153	398	167	385	3.64E-01	-8.80E-02	3.03E-01	1.22E-02	2.62E-01	-1.14E-01	1.86E-01	-4.03E-03	1.41E-01	-1.26E-01	6.13E-02	-4.58E-03	1.78E-02	-1.23E-01
92	484	112	494	134	500	156	495	160	473	172	456	3.45E-01	-1.19E-01	2.96E-01	3.99E-03	2.50E-01	-1.32E-01	1.82E-01	3.26E-03	1.40E-01	-1.32E-01	6.41E-02	-1.96E-03	1.91E-02	-1.26E-01
232	590	255	592	274	584	288	564	301	546	322	536	3.48E-01	-1.05E-01	3.04E-01	1.26E-02	2.50E-01	-1.11E-01	1.97E-01	-2.99E-04	1.32E-01	-1.27E-01	7.33E-02	-1.24E-02	5.17E-03	-1.20E-01
213	480	236	486	260	484	280	472	297	457	320	449	3.45E-01	-1.03E-01	3.02E-01	1.63E-02	2.45E-01	-1.18E-01	1.86E-01	5.00E-03	1.39E-01	-1.15E-01	6.64E-02	1.58E-03	1.90E-02	-1.19E-01
104	505	123	510	143	510	161	503	164	484	180	473	3.59E-01	-1.03E-01	3.03E-01	1.28E-02	2.56E-01	-1.20E-01	1.88E-01	8.71E-04	1.37E-01	-1.25E-01	6.47E-02	1.01E-04	1.16E-02	-1.22E-01
100	470	121	478	143	474	152	453	159	431	178	422	3.34E-01	-1.12E-01	2.98E-01	4.90E-04	2.41E-01	-1.28E-01	1.88E-01	6.85E-03	1.37E-01	-1.25E-01	7.08E-02	5.56E-03	2.61E-02	-1.25E-01
176	989	207	1010	244	1020	281	1013	311	991	327	957	3.54E-01	-1.06E-01	3.06E-01	6.21E-03	2.57E-01	-1.25E-01	1.89E-01	-3.60E-03	1.44E-01	-1.39E-01	5.96E-02	-6.06E-03	9.75E-03	-1.33E-01
255	1058	290	1070	327	1071	353	1047	368	1013	403	1004	3.47E-01	-1.14E-01	3.00E-01	1.19E-02	2.53E-01	-1.29E-01	1.88E-01	7.75E-03	1.35E-01	-1.37E-01	6.21E-02	1.84E-03	1.06E-02	-1.29E-01
207	1022	255	1039	301	1029	341	998	384	973	431	956	3.41E-01	-1.01E-01	2.98E-01	1.99E-03	2.45E-01	-1.21E-01	1.90E-01	1.79E-03	1.30E-01	-1.17E-01	7.74E-02	-1.80E-03	1.62E-02	-1.08E-01
180	1020	218	1029	258	1033	291	1016	318	989	357	979	3.49E-01	-1.17E-01	3.06E-01	1.49E-02	2.45E-01	-1.29E-01	1.94E-01	6.72E-03	1.35E-01	-1.29E-01	6.65E-02	6.09E-03	1.65E-02	-1.21E-01
199	1040	242	1055	287	1059	324	1037	362	1013	405	1000	3.31E-01	-1.22E-01	3.00E-01	2.91E-03	2.36E-01	-1.23E-01	1.93E-01	4.51E-03	1.26E-01	-1.23E-01	7.77E-02	2.65E-03	1.44E-02	-1.20E-01
287	984	329	996	372	1005	415	1005	418	962	446	933	3.35E-01	-1.13E-01	3.06E-01	-8.62E-06	2.36E-01	-1.21E-01	1.97E-01	1.14E-04	1.32E-01	-1.22E-01	7.34E-02	-1.82E-03	1.65E-02	-1.16E-01
182	1018	222	1039	267	1046	307	1037	294	995	325	966	3.39E-01	-1.19E-01	3.02E-01	7.04E-03	2.42E-01	-1.28E-01	1.89E-01	7.87E-03	1.43E-01	-1.26E-01	6.65E-02	4.19E-03	2.21E-02	-1.26E-01
191	1061	230	1067	265	1059	289	1029	324	1010	362	998	3.61E-01	-9.77E-02	3.03E-01	5.72E-03	2.51E-01	-1.18E-01	1.98E-01	-5.28E-03	1.36E-01	-1.22E-01	6.33E-02	-7.80E-03	1.98E-02	-1.16E-01
436	800	471	811	507	817	536	795	569	781	605	771	3.46E-01	-1.03E-01	2.97E-01	2.19E-03	2.44E-01	-1.20E-01	1.91E-01	1.12E-03	1.30E-01	-1.19E-01	7.36E-02	2.81E-03	1.80E-02	-1.10E-01
130	1122	163	1137	199	1135	231	1117	259	1093	291	1073	3.53E-01	-1.18E-01	3.06E-01	1.47E-02	2.46E-01	-1.35E-01	1.89E-01	3.28E-03	1.30E-01	-1.37E-01	5.72E-02	-3.10E-04	1.96E-02	-1.32E-01
215	1018	262	1030	309	1026	346	996	379	960	421	941	3.35E-01	-9.96E-02	2.98E-01	7.27E-03	2.43E-01	-1.12E-01	1.99E-01	-1.52E-03	1.32E-01	-1.15E-01	7.38E-02	-3.58E-03	1.28E-02	-1.10E-01
240	1075	291	1094	342	1090	367	1043	390	996	442	982	3.28E-01	-1.16E-01	2.90E-01	7.19E-03	2.39E-01	-1.24E-01	1.96E-01	3.59E-03	1.36E-01	-1.27E-01	7.95E-02	1.27E-03	2.04E-02	-1.21E-01
240	989	282	1008	326	1020	369	1014	377	968	399	929	3.39E-01	-1.10E-01	3.01E-01	7.93E-03	2.47E-01	-1.24E-01	1.94E-01	4.96E-04	1.34E-01	-1.27E-01	6.86E-02	4.02E-03	2.20E-02	-1.22E-01
238	1091	282	1106	328	1108	353	1068	382	1032	421	1005	3.55E-01	-1.02E-01	2.99E-01	9.84E-04	2.50E-01	-1.18E-01	1.86E-01	-4.03E-05	1.40E-01	-1.18E-01	6.71E-02	-2.29E-03	1.69E-02	-1.15E-01
197	1044	240	1059	286	1059	328	1041	350	1001	384	970	3.51E-01	-1.02E-01	2.96E-01	1.11E-02	2.53E-01	-1.22E-01	1.95E-01	-1.69E-03	1.37E-01	-1.27E-01	6.69E-02	-4.33E-03	2.09E-02	-1.24E-01
166	766	203	770	240	767	267	741	297	717	332	706	3.58E-01	-1.01E-01	3.05E-01	9.76E-03	2.52E-01	-1.23E-01	1.98E-01	-1.16E-03	1.34E-01	-1.30E-01	6.54E-02	-4.25E-03	1.19E-02	-1.21E-01
178	1156	216	1167	255	1174	276	1153	295	1118	329	1098	3.44E-01	-1.07E-01	2.94E-01	2.54E-03	2.47E-01	-1.19E-01	1.87E-01	-4.74E-03	1.32E-01	-1.28E-01	7.96E-02	-8.70E-03	1.05E-02	-1.20E-01
296	1018	334	1031	368	1012	408	1006	448	1001	488	1005	3.45E-01	-1.14E-01	3.04E-01	9.99E-03	2.48E-01	-1.27E-01	1.94E-01	2.38E-03	1.34E-01	-1.28E-01	6.89E-02	5.63E-03	9.36E-03	-1.13E-01
221	969	261	982	301	988	332	962	359	931	394	913	3.44E-01	-1.06E-01	3.04E-01	5.13E-04	2.42E-01	-1.14E-01	1.99E-01	4.00E-04	1.37E-01	-1.15E-01	7.51E-02	-6.33E-04	1.34E-02	-1.17E-01
196	781	241	800	290	810	340	812	383	788	424	761	3.36E-01	-1.14E-01	2.93E-01	4.70E-03	2.38E-01	-1.26E-01	1.93E-01	6.50E-03	1.26E-01	-1.24E-01	7.09E-02	1.16E-03	1.30E-02	-1.24E-01
237	763	274	780	314	791	342	767	360	731	398	715	3.39E-01	-1.19E-01	3.07E-01	7.04E-03	2.43E-01	-1.29E-01	1.95E-01	-4.93E-03	1.39E-01	-1.33E-01	7.12E-02	-6.06E-03	1.48E-02	-1.23E-01
314	835	353	849	394	845	429	828	445	792	481	775	3.44E-01	-1.17E-01	3.05E-01	1.34E-02	2.40E-01	-1.33E-01	1.98E-01	2.30E-04	1.24E-01	-1.40E-01	7.35E-02	-8.12E-04	5.88E-04	-1.25E-01
142	1045	186	1056	230	1065	266	1041	296	1008	336	988	3.42E-01	-1.05E-01	3.09E-01	7.99E-03	2.48E-01	-1.21E-01	1.99E-01	6.07E-05	1.30E-01	-1.17E-01	7.08E-02	1.60E-03	1.06E-02	-1.15E-01
208	741	248	757	288	771	325	760	336	719	368	691	3.51E-01	-1.03E-01	3.04E-01	8.77E-03	2.42E-01	-1.14E-01	1.88E-01	3.66E-03	1.35E-01	-1.20E-01	6.80E-02	-6.47E-05	1.23E-02	-1.19E-01
82	388	103	396	126	398	146	389	167	381	190	381	3.36E-01	-1.23E-01	3.03E-01	1.17E-02	2.36E-01	-1.28E-01	1.86E-01	7.76E-03	1.23E-01	-1.24E-01	6.58E-02	1.26E-02	7.50E-03	-1.18E-01
158	545	184	547	209	541	218	518	228	494	249	478	3.46E-01	-1.01E-01	2.97E-01	1.23E-02	2.49E-01	-1.15E-01	1.88E-01	1.80E-03	1.41E-01	-1.19E-01	6.71E-02	-5.49E-03	3.30E-02	-1.20E-01
123	551	146	558	169	559	179	544	181	522	201	510	3.52E-01	-1.04E-01	2.97E-01	8.86E-03	2.49E-01	-1.32E-01	1.90E-01	3.82E-03	1.32E-01	-1.31E-01	5.81E-02	5.38E-03	6.28E-03	-1.23E-01
100	378	121	389	145	392	168	391	175	369	191	352	3.32E-01	-1.21E-01	3.00E-01	1.57E-02	2.32E-01	-1.33E-01	1.88E-01	6.99E-03	1.23E-01	-1.34E-01	7.55E-02	2.71E-03	2.96E-03	-1.25E-01
97	532	123	541	150	542	175	529	190	507	202	483	3.40E-01	-1.03E-01	3.03E-01	1.41E-02	2.39E-01	-1.13E-01	1.88E-01	-4.33E-03	1.34E-01	-1.21E-01	7.71E-02	-1.04E-03	2.48E-02	-1.16E-01
115	363	141	376	168	382	190	370	202	344	220	322	3.39E-01	-1.06E-01	2.97E-01	1.12E-02	2.38E-01	-1.25E-01	1.81E-01	4.26E-03	1.34E-01	-1.29E-01	7.16E-02	3.49E-03	2.25E-02	-1.28E-01
115	354	139	363	164	365	187	355	201	334	223	323	3.48E-01	-1.12E-01	2.95E-01	2.39E-03	2.40E-01	-1.12E-01	1.93E-01	-4.61E-03	1.34E-01	-1.16E-01	7.48E-02	-2.85E-03	2.12E-02	-1.05E-01
89	518	111	522	133	517	147	499	160	480	180	472	3.54E-01	-1.03E-01	2.97E-01	4.15E-03	2.41E-01	-1.18E-01	1.88E-01	8.25E-04	1.35E-01	-1.18E-01	7.07E-02	-2.18E-03	1.90E-02	-1.10E-01
97	518	123	528	150	532	172	518	178	491	194	470	3.31E-01	-1.10E-01	2.99E-01	6.94E-03	2.40E-01	-1.16E-01	1.92E-01	3.42E-03	1.37E-01	-1.12E-01	7.05E-02	3.41E-03	2.19E-02	-1.12E-01
84	397	107	400	130	398	148	384	157	362	176	349	3.70E-01	-1.08E-01	3.06E-01	1.29E-02	2.47E-01	-1.15E-01	1.83E-01	1.03E-02	1.33E-01	-1.23E-01	6.42E-02	-2.21E-03	9.81E-03	-1.13E-01
97	553	119	556	141	554	146	534	155	514	173	501	3.40E-01	-1.10E-01	3.04E-01	1.64E-02	2.43E-01	-1.16E-01	1.90E-01	2.02E-03	1.39E-01	-1.23E-01	7.14E-02	-5.22E-03	1.28E-02	-1.23E-01
126	367	146	372	167	374	180	362	190	343	208	333	3.51E-01	-1.07E-01	3.05E-01	1.27E-02	2.52E-01	-1.26E-01	1.86E-01	3.64E-03	1.32E-01	-1.31E-01	6.12E-02	4.02E-03	8.54E-03	-1.18E-01
99	389	122	398	145	399	158	379	173	361	196	353	3.43E-01	-1.15E-01	2.95E-01	5.60E-03	2.40E-01	-1.21E-01	1.86E-01	8.00E-03	1.36E-01	-1.20E-01	6.95E-02	3.28E-03	2.79E-02	-1.11E-01

106	507	129	515	154	517	173	504	178	480	194	461	3.36E-01	-1.06E-01	2.92E-01	-1.18E-03	2.62E-01	-1.16E-01	1.83E-01	-4.54E-03	1.48E-01	-1.23E-01	6.69E-02	2.65E-04	1.94E-02	-1.24E-01
145	514	167	517	190	514	205	497	222	483	244	476	3.46E-01	-1.10E-01	2.89E-01	4.65E-03	2.42E-01	-1.25E-01	1.86E-01	-1.11E-03	1.35E-01	-1.28E-01	7.11E-02	2.43E-03	3.41E-02	-1.21E-01
119	499	140	502	162	501	178	492	188	473	206	463	3.52E-01	-1.19E-01	2.95E-01	1.64E-02	2.42E-01	-1.39E-01	1.81E-01	-2.39E-04	1.32E-01	-1.34E-01	7.03E-02	-7.11E-04	1.68E-02	-1.23E-01
111	546	136	550	158	542	169	521	178	498	199	488	3.28E-01	-1.08E-01	2.89E-01	-2.69E-04	2.36E-01	-1.17E-01	1.95E-01	-3.96E-03	1.29E-01	-1.16E-01	8.21E-02	-4.53E-03	2.85E-02	-1.12E-01
112	363	137	370	163	369	185	356	198	333	219	321	3.41E-01	-1.11E-01	2.93E-01	1.30E-02	2.44E-01	-1.21E-01	1.89E-01	-1.90E-03	1.31E-01	-1.26E-01	6.89E-02	-3.53E-03	2.67E-02	-1.30E-01
129	423	153	430	178	433	200	423	213	401	233	386	3.35E-01	-9.48E-02	3.02E-01	1.07E-02	2.51E-01	-1.12E-01	1.94E-01	1.85E-03	1.43E-01	-1.22E-01	7.32E-02	-3.63E-04	2.91E-02	-1.20E-01
154	252	170	256	185	256	198	248	212	240	228	240	3.43E-01	-1.13E-01	2.99E-01	-1.74E-03	2.40E-01	-1.28E-01	1.96E-01	-3.02E-03	1.32E-01	-1.30E-01	6.73E-02	-5.49E-03	1.31E-02	-1.20E-01
112	273	124	280	136	286	150	287	158	280	165	269	3.44E-01	-1.12E-01	3.04E-01	4.74E-03	2.43E-01	-1.19E-01	2.00E-01	-6.60E-03	1.33E-01	-1.22E-01	5.58E-02	-1.89E-03	1.41E-02	-1.20E-01
146	255	163	260	181	260	196	252	208	238	225	233	3.48E-01	-1.17E-01	2.99E-01	8.09E-03	2.43E-01	-1.22E-01	1.87E-01	-1.03E-03	1.31E-01	-1.24E-01	6.48E-02	-3.79E-03	1.32E-02	-1.16E-01
125	214	138	223	154	225	167	218	181	211	197	209	3.42E-01	-1.16E-01	2.98E-01	1.31E-03	2.41E-01	-1.24E-01	1.87E-01	3.25E-03	1.36E-01	-1.25E-01	5.77E-02	2.62E-03	9.33E-03	-1.22E-01
150	221	162	230	176	235	185	226	196	217	210	212	3.53E-01	-1.16E-01	2.94E-01	3.98E-03	2.49E-01	-1.28E-01	1.83E-01	-5.59E-03	1.34E-01	-1.29E-01	5.84E-02	-6.48E-03	7.82E-03	-1.21E-01
103	266	116	272	131	277	141	267	151	257	166	254	3.58E-01	-1.04E-01	2.96E-01	3.09E-03	2.47E-01	-1.22E-01	1.89E-01	-5.86E-03	1.37E-01	-1.24E-01	5.88E-02	-2.06E-03	1.41E-02	-1.18E-01
106	283	123	288	140	287	154	276	169	269	187	265	3.49E-01	-1.13E-01	2.96E-01	5.45E-03	2.45E-01	-1.24E-01	1.87E-01	-4.20E-03	1.37E-01	-1.25E-01	6.59E-02	-4.76E-03	2.39E-02	-1.17E-01
108	306	124	311	141	312	156	305	168	295	184	289	3.46E-01	-1.04E-01	3.01E-01	3.34E-03	2.41E-01	-1.21E-01	1.90E-01	6.42E-04	1.33E-01	-1.21E-01	6.34E-02	-2.77E-03	1.37E-02	-1.11E-01
160	220	169	225	179	228	190	230	198	224	207	219	3.61E-01	-1.14E-01	3.06E-01	1.29E-02	2.57E-01	-1.28E-01	1.88E-01	3.05E-03	1.30E-01	-1.33E-01	6.26E-02	5.29E-03	3.37E-03	-1.34E-01
89	280	104	289	122	291	135	283	146	269	161	261	3.44E-01	-1.09E-01	3.00E-01	1.11E-02	2.40E-01	-1.24E-01	1.96E-01	2.09E-04	1.27E-01	-1.27E-01	8.89E-02	-8.04E-03	4.23E-03	-1.22E-01
107	294	125	300	143	300	154	286	166	271	182	264	3.33E-01	-1.12E-01	3.00E-01	3.78E-03	2.30E-01	-1.18E-01	1.96E-01	-1.43E-03	1.31E-01	-1.15E-01	7.52E-02	-1.04E-03	1.40E-02	-1.13E-01
154	230	167	235	182	237	195	232	203	221	216	214	3.55E-01	-1.11E-01	3.05E-01	1.41E-02	2.55E-01	-1.25E-01	1.87E-01	1.07E-03	1.37E-01	-1.25E-01	5.71E-02	-5.72E-03	6.22E-03	-1.19E-01
175	331	189	336	203	339	217	338	220	323	229	312	3.41E-01	-1.13E-01	2.95E-01	2.69E-03	2.42E-01	-1.24E-01	1.81E-01	-2.21E-03	1.37E-01	-1.31E-01	7.24E-02	-2.45E-03	1.52E-02	-1.26E-01
153	242	164	247	176	246	187	240	199	235	211	233	3.50E-01	-1.19E-01	3.06E-01	9.72E-03	2.47E-01	-1.28E-01	1.82E-01	-5.35E-04	1.39E-01	-1.29E-01	6.30E-02	-4.94E-03	1.58E-02	-1.28E-01
133	262	148	267	164	271	175	261	187	250	203	245	3.46E-01	-1.09E-01	3.05E-01	1.34E-02	2.47E-01	-1.19E-01	1.90E-01	-1.76E-03	1.34E-01	-1.24E-01	6.26E-02	-1.39E-03	1.74E-02	-1.22E-01
175	199	187	206	199	208	212	205	222	195	235	190	3.56E-01	-1.10E-01	3.02E-01	9.39E-03	2.50E-01	-1.29E-01	1.81E-01	7.71E-03	1.35E-01	-1.31E-01	7.29E-02	-4.66E-03	7.86E-03	-1.28E-01
98	289	114	298	133	301	149	293	164	282	180	272	3.43E-01	-1.02E-01	2.98E-01	7.07E-04	2.44E-01	-1.26E-01	1.89E-01	-6.00E-03	1.54E-01	-1.22E-01	6.82E-02	-3.49E-03	2.48E-02	-1.17E-01
122	535	145	541	168	545	173	527	181	506	201	493	3.38E-01	-1.08E-01	2.99E-01	1.01E-02	2.41E-01	-1.18E-01	1.87E-01	1.70E-03	1.29E-01	-1.27E-01	7.15E-02	-1.11E-03	2.25E-02	-1.18E-01
134	503	157	507	179	510	192	494	204	474	225	464	3.47E-01	-1.09E-01	2.99E-01	9.55E-03	2.36E-01	-1.24E-01	1.90E-01	-4.89E-03	1.30E-01	-1.26E-01	7.63E-02	-3.92E-03	5.90E-03	-1.19E-01
160	570	178	574	197	573	210	561	223	549	241	544	3.44E-01	-1.20E-01	3.06E-01	8.83E-03	2.44E-01	-1.35E-01	1.90E-01	-9.31E-04	1.30E-01	-1.31E-01	6.76E-02	4.13E-03	8.44E-03	-1.21E-01
132	440	152	446	171	449	184	439	190	420	208	410	3.47E-01	-1.01E-01	3.04E-01	2.09E-03	2.44E-01	-1.19E-01	2.04E-01	1.17E-03	1.27E-01	-1.23E-01	6.21E-02	2.29E-03	1.56E-02	-1.16E-01
113	474	135	480	158	484	176	471	190	452	209	439	3.32E-01	-1.10E-01	2.96E-01	6.22E-03	2.41E-01	-1.21E-01	1.92E-01	8.74E-03	1.24E-01	-1.30E-01	6.79E-02	8.83E-03	1.78E-02	-1.16E-01
105	503	126	509	147	500	163	484	184	476	206	470	3.45E-01	-1.06E-01	2.91E-01	9.22E-03	2.46E-01	-1.21E-01	1.90E-01	-4.41E-03	1.32E-01	-1.26E-01	8.10E-02	-6.30E-03	5.64E-03	-1.20E-01
177	409	197	417	218	423	240	427	248	412	260	395	3.43E-01	-1.10E-01	2.99E-01	1.47E-02	2.40E-01	-1.27E-01	1.90E-01	-2.13E-04	1.31E-01	-1.33E-01	5.70E-02	-1.93E-03	1.57E-02	-1.34E-01
113	549	136	556	158	557	173	540	185	522	205	510	3.42E-01	-1.01E-01	2.98E-01	8.37E-03	2.40E-01	-1.30E-01	1.93E-01	-3.74E-03	1.24E-01	-1.30E-01	8.40E-02	-5.03E-03	9.04E-03	-1.16E-01
115	530	135	535	155	533	171	520	186	506	204	495	3.42E-01	-1.08E-01	3.03E-01	1.08E-02	2.34E-01	-1.19E-01	1.90E-01	2.69E-03	1.28E-01	-1.29E-01	6.89E-02	-3.49E-03	2.79E-02	-1.22E-01
165	539	187	547	211	549	232	540	243	520	265	511	3.39E-01	-1.06E-01	2.97E-01	6.47E-03	2.44E-01	-1.15E-01	1.95E-01	-4.62E-03	1.31E-01	-1.26E-01	7.13E-02	-1.04E-02	1.43E-02	-1.25E-01
125	412	154	420	184	423	209	413	225	388	247	370	3.39E-01	-1.09E-01	2.88E-01	5.77E-03	2.43E-01	-1.22E-01	1.86E-01	-1.16E-03	1.35E-01	-1.28E-01	7.84E-02	-2.69E-03	3.14E-02	-1.20E-01
141	390	163	393	184	388	198	373	208	354	225	341	3.41E-01	-1.15E-01	2.91E-01	1.25E-02	2.48E-01	-1.30E-01	1.88E-01	1.85E-03	1.39E-01	-1.39E-01	7.37E-02	-6.38E-03	2.29E-02	-1.40E-01
166	356	179	365	195	368	206	357	218	346	233	340	3.40E-01	-1.18E-01	2.94E-01	3.78E-03	2.41E-01	-1.26E-01	1.92E-01	3.61E-03	1.30E-01	-1.27E-01	7.13E-02	-2.24E-05	2.08E-03	-1.20E-01
172	356	187	363	201	357	210	343	224	334	241	332	3.46E-01	-1.07E-01	2.92E-01	1.99E-03	2.47E-01	-1.26E-01	1.92E-01	-4.07E-03	1.29E-01	-1.31E-01	7.27E-02	-1.94E-04	1.08E-02	-1.23E-01
167	356	183	362	200	364	213	354	220	339	236	333	3.50E-01	-1.12E-01	3.01E-01	1.19E-02	2.55E-01	-1.26E-01	1.87E-01	2.38E-04	1.39E-01	-1.33E-01	6.57E-02	-8.80E-03	1.41E-02	-1.25E-01
153	363	169	370	185	370	198	358	211	347	227	340	3.38E-01	-1.06E-01	3.00E-01	4.63E-03	2.36E-01	-1.29E-01	1.98E-01	2.11E-03	1.21E-01	-1.33E-01	8.12E-02	-1.01E-02	6.21E-03	-1.29E-01
148	344	162	349	177	348	189	337	202	328	217	324	3.49E-01	-9.99E-02	2.91E-01	7.56E-04	2.40E-01	-1.25E-01	1.85E-01	-3.55E-03	1.32E-01	-1.33E-01	7.45E-02	-9.09E-03	1.65E-02	-1.30E-01
164	354	180	357	196	357	207	345	220	336	236	333	3.43E-01	-1.02E-01	2.99E-01	-1.95E-03	2.42E-01	-1.21E-01	1.93E-01	-2.35E-03	1.21E-01	-1.25E-01	7.69E-02	-3.76E-05	4.67E-03	-1.17E-01
162	317	176	323	190	327	204	325	215	315	230	311	3.50E-01	-1.01E-01	3.05E-01	3.33E-03	2.43E-01	-1.25E-01	1.98E-01	-2.84E-03	1.19E-01	-1.29E-01	6.33E-02	-2.96E-03	-2.90E-03	-1.19E-01
156	344	171	349	187	351	201	345	213	335	228	329	3.43E-01	-1.08E-01	3.00E-01	6.51E-03	2.41E-01	-1.21E-01	1.93E-01	-5.52E-03	1.31E-01	-1.22E-01	7.42E-02	-4.80E-03	1.31E-02	-1.19E-01
156	334	173	340	190	343	198	327	208	314	224	308	3.40E-01	-1.07E-01	2.92E-01	1.10E-03	2.34E-01	-1.29E-01	1.92E-01	-4.66E-03	1.23E-01	-1.34E-01	8.65E-02	-5.20E-03	8.47E-03	-1.30E-01
177	348	193	354	209	357	226	356	232	344	247	339	3.30E-01	-1.17E-01	2.94E-01	4.89E-03	2.42E-01	-1.21E-01	1.87E-01	1.71E-03	1.38E-01	-1.27E-01	7.17E-02	2.43E-03	2.29E-02	-1.26E-01
147	368	166	373	186	374	196	363	201	345	219	339	3.45E-01	-1.01E-01	3.01E-01	9.52E-03	2.47E-01	-1.19E-01	1.96E-01	-1.82E-03	1.33E-01	-1.23E-01	6.92E-02	1.88E-03	1.33E-02	-1.12E-01
176	353	191	357	206	354	216	342	227	331	241	326	3.45E-01	-1.16E-01	3.02E-01	5.08E-03	2.36E-01	-1.21E-01	1.92E-01	6.73E-03	1.25E-01	-1.27E-01	7.44E-02	-5.60E-03	8.59E-03	-1.28E-01
194	337	206	342	218	338	228	329	237	320	250	316	3.44E-01	-1.18E-01	3.04E-01	7.44E-03	2.46E-01	-1.20E-01	1.91E-01	-6.89E-03	1.30E-01	-1.23E-01	6.59E-02	2.81E-03	1.02E-02	-1.22E-01
94	333	112	341	130	346	138	335	139	316	154	305	3.42E-01	-1.14E-01	2.98E-01	7.20E-03	2.37E-01	-1.28E-01	1.90E-01	-3.08E-03	1.29E-01	-1.30E-01	6.36E-02	3.66E-03	1.24E-02	-1.23E-01
106	294	120	302	135	305	151	304	155	291	165	280	3.43E-01	-1.17E-01	3.02E-01	5.44E-04	2.32E-01	-1.22E-01	1.89E-01	-2.96E-03	1.19E-01	-1.23E-01	5.69E-02	1.17E-03	2.57E-03	-1.17E-01
102	307	118	311	133	312	139	296	150	284	165	278	3.46E-01	-9.93E-02	3.02E-01	3.48E-03	2.42E-01	-1.18E-01	1.91E-01	-2.19E-03	1.32E-01	-1.20E-01	7.53E-02	-1.07E-02	1.40E-02	-1.20E-01
103	297	117	305	133	308	146	299	159	291	175	289	3.37E-01	-1.19E-01	3.05E-01	3.60E-03	2.42E-01	-1.18E-01	1.90E-01	3.21E-03	1.31E-01	-1.17E-01	6.54E-02	6.20E-03	2.06E-02	-1.15E-01
131	307	148	310	165	311	176	298	186	285	201	278	3.56E-01	-9.09E-02	3.03E-01	7.23E-03	2.48E-01	-1.14E-01	1.90E-01	6.84E-03	1.30E-01	-1.16E-01	6.73E-02	-4.13E-04	1.22E-02	-1.14E-01
101	294	115	301	131	303	142	292	151	279	166	276	3.51E-01	-9.94E-02	2.98E-01	1.96E-03	2.44E-01	-1.18E-01	1.91E-01	-1.00E-02	1.32E-01	-1.19E-01	6.36E-02	-5.98E-03	1.95E-02	-1.14E-01
99	298	117	305	134	301	148	291	161	277	177	270	3.36E-01	-1.05E-01	2.94E-01	6.90E-03	2.43E-01	-1.19E-01	1.84E-01	6.60E-04	1.32E-01	-1.25E-01	6.94E-02	-4.23E-03	2.25E-02	-1.22E-01
111	297	127	304	145	303	159	293	174	284	191	280	3.45E-01	-1.06E-01	2.98E-01	6.89E-03	2.36E-01	-1.21E-01	1.85E-01	-6.20E-03	1.29E-01	-1.27E-01	6.15E-02	3.30E-03	1.96E-02	-1.23E-01
112	292	127	300	143	305	154	293	163	279	178	271	3.45E-01	-1.09E-01	3.01E-01	6.66E-03	2.44E-01	-1.20E-01	1.82E-01	-5.83E-03	1.27E-01	-1.24E-01	6.88E-02	-4.14E-03	1.66E-02	-1.21E-01
139	278	155	288	172	290	179	274	189	259	206	253	3.36E-01	-1.09E-01	2.93E-01	9.38E-03	2.32E-01	-1.17E-01	1.93E-01	-7.95E-04	1.24E-01	-1.18E-01	7.46E-02	-2.24E-03	1.92E-02	-1.17E-01
136	294	151	299	168	298	183	292	196	281	210	275	3.47E-01	-1.06E-01	3.01E-01	4.25E-03	2.41E-01	-1.15E-01	1.94E-01	-2.08E-03	1.34E-01	-1.15E-01	6.01E-02	-1.57E-05	2.09E-02	-1.14E-01
94	313	112	321	131	325	147	318	149	299	165	288	3.32E-01	-1.08E-01	2.99E-01	2.91E-03	2.38E-01	-1.18E-01	1.92E-01	-3.09E-03	1.32E-01	-1.17E-01	6.14E-02	2.15E-03	2.03E-02	-1.15E-01
101	281	116	287	133	288	145	279	154	266	169	258	3.43E-01	-1.11E-01	2.97E-01	2.33E-03	2.43E-01	-1.24E-01	1.88E-01	2.29E-03	1.36E-01	-1.23E-01	6.74E-02	-3.94E-03	1.47E-02	-1.22E-01
125	263	142	270	161	271	173	257	186	244	203	237	3.37E-01	-9.94E-02	3.01E-01	1.07E-02	2.40E-01	-1.11E-01	1.97E-01	-5.86E-03	1.28E-01	-1.17E-01	7.29E-02	-2.58E-03	2.29E-02	-1.16E-01
87	287	101	294	116	298	129	291	138	278	152	272	3.58E-01	-1.05E-01	2.99E-01	5.66E-03	2.49E-01	-1.22E-01	1.85E-01	-2.75E-03	1.38E-01	-1.24E-01	5.87E-02	-5.27E-03	1.13E-02	-1.19E-01
129	300	147	303	165	301	176	286	185	271	203	267	3.41E-01	-9.46E-02	2.94E-01	7.18E-03	2.46E-01	-1.07E-01	1.92E-01	-5.26E-03	1.43E-01	-1.20E-01	6.72E-02	-4.48E-03	2.11E-02	-1.22E-01
118	277	132	286	148	286	158	274	170	265	186	263	3.37E-01	-1.14E-01	3.01E-01	2.12E-03	2.36E-01	-1.18E-01	1.94E-01	6.62E-04	1.26E-01	-1.14E-01	6.73E-02	8.95E-04	1.23E-02	-1.12E-01
143	289	159	295	176	299	192	297	199	281	212	271	3.45E-01	-1.09E-01	2.90E-01	6.17E-03	2.39E-01	-1.15E-01	1.92E-01	-4.69E-03	1.29E-01	-1.22E-01	7.15E-02	-8.01E-03	1.91E-02	-1.21E-01
129	292	143	300	159	305	170	293	179	280	194	272	3.51E-01	-1.02E-01	3.00E-01	3.77E-03	2.43E-01	-1.18E-01	1.94E-01	-2.07E-04	1.34E-01	-1.23E-01	6.54E-02	3.96E-03	2.06E-02	-1.15E-01
114	316	130	323	147	325	159	316	167	301	182	294	3.48E-01	-9.77E-02	2.99E-01	2.89E-03	2.41E-01	-1.13E-01	1.91E-01	-4.33E-04	1.31E-01	-1.16E-01	6.91E-02	-2.51E-03	2.20E-02	-1.12E-01
112	300	128	305	144	305	155	294	167	283	182	278	3.52E-01	-9.38E-02	2.94E-01	6.44E-03	2.40E-01	-1.21E-01	1.92E-01	-4.48E-03	1.31E-01	-1.20E-01	6.79E-02	-4.07E-03	8.52E-03	-1.23E-01
113	288	129	294	146	295	161	286	172	272	188	265	3.52E-01	-9.93E-02	2.99E-01	8.33E-03	2.49E-01	-1.27E-01	1.86E-01	5.15E-03	1.36E-01	-1.30E-01	6.26E-02	-5.90E-03	2.64E-02	-1.32E-01
110	314	127	319	145	322	158	311	168	296	182	286	3.44E-01	-1.04E-01	3.02E-01	6.06E-03	2.44E-01	-1.17E-01	1.93E-01	-1.11E-03	1.29E-01	-1.23E-01	6.33E-02	-6.38E-03	2.19E-02	-1.20E-01
93	299	109	305	125	305	136	295	146	281	160	273	3.52E-01	-1.04E-01	3.01E-01	1.32E-02	2.52E-01	-1.22E-01	1.93E-01	-7.77E-04	1.41E-01	-1.32E-01	7.33E-02	-8.06E-03	1.66E-02	-1.31E-01
97	315	111	322	127	325	136	313	146	300	161	295	3.38E-01	-1.01E-01	3.02E-01	6.17E-03	2.39E-01	-1.16E-01	1.94E-01	1.58E-03	1.34E-01	-1.22E-01	7.58E-02	-5.69E-03	1.70E-02	-1.23E-01
113	280	128	286	143	290	156	282	166	269	179	261	3.45E-01	-1.08E-01	3.07E-01	6.39E-03	2.44E-01	-1.15E-01	1.95E-01	-1.05E-03	1.28E-01	-1.14E-01	5.90E-02	-5.46E-04	2.48E-02	-1.10E-01
111	283	125	287	140	285	152	277	160	265	172	257	3.51E-01	-1.02E-01	3.09E-01	5.86E-03	2.48E-01	-1.17E-01	1.93E-01	2.85E-03	1.28E-01	-1.14E-01	6.00E-02	3.56E-03	1.42E-02	-1.10E-01
112	304	127	309	138	304	147	292	160	284	174	278	3.49E-01	-1.02E-01	3.00E-01	4.45E-03	2.46E-01	-1.17E-01	1.92E-01	4.48E-04	1.39E-01	-1.20E-01	6.48E-02	7.45E-04	2.35E-02	-1.17E-01
102	293	117	299	132	303	143	293	153	281	166	272	3.50E-01	-9.88E-02	2.99E-01	4.39E-03	2.45E-01	-1.19E-01	1.94E-01	-4.50E-03	1.31E-01	-1.26E-01	6.59E-02	-8.31E-03	2.24E-02	-1.21E-01
123	295	139	300	155	300	166	287	179	276	196	272	3.48E-01	-1.02E-01	3.05E-01	7.19E-03	2.46E-01	-1.15E-01	1.92E-01	-1.04E-03	1.37E-01	-1.18E-01	6.36E-02	-1.91E-03	2.02E-02	-1.20E-01
153	330	166	341	182	342	193	330	196	313	207	301	3.45E-01	-1.10E-01	3.17E-01	1.48E-02	2.46E-01	-1.23E-01	1.96E-01	6.54E-04	1.23E-01	-1.33E-01	5.70E-02	4.18E-03	7.44E-03	-1.24E-01
115	277	130	286	147	289	156	276	166	262	183	256	3.40E-01	-1.02E-01	3.04E-01	5.01E-03	2.45E-01	-1.24E-01	1.93E-01	-5.10E-03	1.29E-01	-1.24E-01	6.41E-02	-1.92E-03	1.39E-02	-1.18E-01
159	357	179	361	199	362	217	357	225	338	240	326	3.46E-01	-1.00E-01	2.97E-01	8.98E-03	2.51E-01	-1.20E-01	1.86E-01	-2.15E-04	1.28E-01	-1.25E-01	7.00E-02	7.40E-04	1.15E-02	-1.16E-01
145	336	156	342	169	343	180	339	191	331	203	328	3.64E-01	-1.03E-01	2.99E-01	1.73E-02	2.58E-01	-1.21E-01	1.86E-01	4.75E-03	1.40E-01	-1.30E-01	6.04E-02	-2.52E-03	8.97E-03	-1.26E-01
172	308	190	313	207	317	224	316	237	304	252	295	3.41E-01	-1.04E-01	3.03E-01	1.10E-02	2.45E-01	-1.17E-01	1.88E-01	3.36E-03	1.29E-01	-1.29E-01	6.00E-02	-3.78E-03	8.67E-03	-1.28E-01

161	318	176	324	192	327	203	317	212	303	225	293	3.31E-01	-1.11E-01	3.07E-01	3.38E-03	2.36E-01	-1.16E-01	1.95E-01	-4.03E-04	1.28E-01	-1.15E-01	6.27E-02	3.57E-03	2.13E-02	-1.11E-01
145	266	162	273	180	278	197	275	211	263	226	253	3.36E-01	-1.12E-01	3.05E-01	1.45E-02	2.41E-01	-1.28E-01	1.93E-01	-9.86E-04	1.32E-01	-1.37E-01	5.64E-02	3.86E-03	1.40E-02	-1.31E-01
157	234	172	240	188	244	199	237	209	225	224	219	3.42E-01	-1.09E-01	3.06E-01	9.14E-03	2.44E-01	-1.25E-01	1.96E-01	-1.60E-03	1.24E-01	-1.22E-01	6.13E-02	6.26E-03	3.89E-03	-1.17E-01
166	328	183	335	201	339	214	329	221	312	232	297	3.45E-01	-1.07E-01	3.02E-01	1.08E-02	2.40E-01	-1.22E-01	1.98E-01	-3.30E-03	1.22E-01	-1.22E-01	6.44E-02	-2.65E-04	7.46E-03	-1.18E-01
171	305	186	313	202	319	216	313	228	301	241	289	3.46E-01	-1.10E-01	3.03E-01	7.32E-03	2.50E-01	-1.24E-01	1.89E-01	5.21E-03	1.31E-01	-1.23E-01	5.89E-02	3.20E-03	1.39E-02	-1.23E-01
141	263	157	268	174	270	187	259	194	244	206	232	3.50E-01	-1.04E-01	3.08E-01	6.10E-03	2.45E-01	-1.21E-01	1.95E-01	1.43E-04	1.27E-01	-1.21E-01	6.44E-02	-5.24E-03	3.17E-03	-1.09E-01
74	297	92	306	111	309	127	300	137	283	152	272	3.26E-01	-1.14E-01	3.05E-01	7.86E-03	2.39E-01	-1.24E-01	2.00E-01	8.02E-04	1.33E-01	-1.27E-01	7.58E-02	-2.24E-03	1.55E-02	-1.26E-01
177	327	195	333	213	337	228	328	241	315	257	304	3.48E-01	-9.68E-02	3.06E-01	1.15E-02	2.50E-01	-1.17E-01	1.95E-01	-2.24E-03	1.35E-01	-1.25E-01	6.69E-02	-2.27E-03	1.03E-02	-1.29E-01
128	355	143	362	160	361	169	348	180	335	196	329	3.44E-01	-1.02E-01	3.12E-01	4.48E-03	2.49E-01	-1.13E-01	1.98E-01	-1.02E-03	1.35E-01	-1.20E-01	6.07E-02	2.22E-03	1.32E-02	-1.20E-01
165	354	181	360	198	361	210	356	218	341	233	333	3.44E-01	-1.07E-01	3.07E-01	1.20E-02	2.48E-01	-1.17E-01	1.87E-01	2.32E-03	1.37E-01	-1.24E-01	5.58E-02	-2.49E-03	2.72E-02	-1.27E-01
82	282	99	291	117	294	133	286	147	273	161	260	3.38E-01	-1.01E-01	3.05E-01	-3.80E-04	2.37E-01	-1.17E-01	1.89E-01	2.39E-03	1.29E-01	-1.20E-01	6.24E-02	4.20E-03	2.01E-02	-1.18E-01
118	268	135	274	152	277	164	266	176	254	192	246	3.52E-01	-1.12E-01	3.09E-01	9.96E-03	2.56E-01	-1.30E-01	1.90E-01	-2.60E-03	1.44E-01	-1.34E-01	5.48E-02	2.52E-03	1.83E-02	-1.21E-01
165	361	179	368	194	369	206	362	216	351	228	341	3.56E-01	-9.68E-02	3.12E-01	1.54E-02	2.42E-01	-1.16E-01	1.96E-01	7.55E-03	1.19E-01	-1.25E-01	5.42E-02	-2.39E-04	1.71E-04	-1.23E-01
167	327	183	334	200	337	212	326	223	312	238	304	3.36E-01	-1.03E-01	3.06E-01	-2.39E-03	2.40E-01	-1.16E-01	1.95E-01	-6.63E-04	1.23E-01	-1.20E-01	6.68E-02	2.07E-03	1.59E-02	-1.13E-01
152	322	168	326	184	324	199	318	216	313	233	313	3.43E-01	-1.09E-01	3.02E-01	1.08E-02	2.40E-01	-1.17E-01	1.94E-01	-4.81E-03	1.32E-01	-1.24E-01	7.03E-02	2.26E-03	9.57E-03	-1.20E-01
172	336	187	342	203	345	213	337	218	322	233	315	3.45E-01	-1.08E-01	3.05E-01	1.34E-02	2.48E-01	-1.17E-01	1.92E-01	4.69E-03	1.29E-01	-1.20E-01	6.63E-02	4.33E-03	1.41E-02	-1.15E-01
142	337	156	344	171	348	185	344	188	328	201	319	3.38E-01	-1.04E-01	3.04E-01	1.02E-02	2.45E-01	-1.18E-01	1.98E-01	2.48E-03	1.26E-01	-1.22E-01	5.94E-02	2.71E-03	1.74E-02	-1.15E-01
159	338	176	344	193	349	209	343	224	334	241	329	3.43E-01	-1.02E-01	3.04E-01	6.11E-03	2.42E-01	-1.14E-01	1.88E-01	-1.04E-03	1.35E-01	-1.15E-01	6.86E-02	6.23E-03	2.37E-02	-1.09E-01
177	337	192	342	208	344	220	336	232	325	247	320	3.43E-01	-1.13E-01	3.03E-01	5.28E-03	2.45E-01	-1.21E-01	1.96E-01	-3.25E-04	1.30E-01	-1.30E-01	6.14E-02	-3.78E-04	2.05E-02	-1.20E-01
166	344	183	351	201	356	215	351	220	334	232	320	3.37E-01	-1.11E-01	3.07E-01	4.07E-03	2.39E-01	-1.25E-01	1.94E-01	7.80E-03	1.39E-01	-1.33E-01	6.54E-02	1.03E-03	2.02E-02	-1.16E-01
180	340	195	343	212	345	223	337	233	324	249	321	3.43E-01	-1.04E-01	3.00E-01	1.36E-02	2.41E-01	-1.23E-01	1.90E-01	3.13E-03	1.28E-01	-1.27E-01	6.45E-02	8.74E-03	2.19E-02	-1.24E-01
154	359	168	365	182	366	194	357	204	346	218	342	3.40E-01	-1.15E-01	3.03E-01	9.46E-03	2.44E-01	-1.28E-01	1.92E-01	1.65E-03	1.30E-01	-1.28E-01	6.59E-02	4.57E-03	1.95E-02	-1.23E-01
150	345	166	351	184	352	200	346	215	337	233	335	3.42E-01	-1.05E-01	3.01E-01	-7.36E-04	2.45E-01	-1.21E-01	1.90E-01	5.99E-03	1.40E-01	-1.21E-01	6.30E-02	4.91E-03	2.47E-02	-1.17E-01
164	339	177	347	193	351	202	341	213	332	229	329	3.46E-01	-1.17E-01	3.05E-01	-8.22E-04	2.43E-01	-1.25E-01	1.85E-01	-3.34E-03	1.31E-01	-1.24E-01	5.50E-02	6.90E-03	2.14E-02	-1.17E-01
162	341	179	346	196	349	211	342	224	331	241	328	3.48E-01	-9.83E-02	2.97E-01	1.93E-03	2.42E-01	-1.17E-01	1.87E-01	4.79E-03	1.29E-01	-1.22E-01	6.70E-02	5.39E-03	2.15E-02	-1.18E-01
168	345	183	351	199	348	208	334	218	322	233	315	3.37E-01	-1.25E-01	3.07E-01	1.57E-02	2.41E-01	-1.26E-01	1.92E-01	5.20E-03	1.31E-01	-1.29E-01	7.39E-02	5.39E-03	9.39E-03	-1.17E-01
153	336	170	345	189	350	206	343	218	329	237	324	3.38E-01	-1.09E-01	2.94E-01	-3.57E-03	2.44E-01	-1.17E-01	1.87E-01	1.07E-03	1.38E-01	-1.22E-01	5.93E-02	8.55E-03	3.63E-02	-1.21E-01
217	1015	260	1035	308	1040	350	1027	368	982	408	957	3.46E-01	-1.19E-01	2.98E-01	2.50E-03	2.43E-01	-1.28E-01	1.81E-01	7.35E-04	1.37E-01	-1.23E-01	7.02E-02	5.68E-03	1.86E-02	-1.17E-01
175	1126	221	1135	268	1139	316	1135	323	1091	357	1060	3.43E-01	-1.04E-01	3.03E-01	-1.89E-04	2.44E-01	-1.07E-01	1.89E-01	-2.57E-03	1.41E-01	-1.10E-01	7.03E-02	-3.86E-03	1.71E-02	-1.14E-01
183	1012	217	1019	248	1008	277	991	307	974	341	968	3.57E-01	-9.69E-02	3.13E-01	-1.33E-04	2.52E-01	-1.17E-01	1.94E-01	1.12E-04	1.35E-01	-1.18E-01	6.11E-02	-1.48E-03	1.72E-02	-1.16E-01
291	743	323	748	356	751	381	731	403	709	435	699	3.66E-01	-1.02E-01	2.98E-01	7.48E-03	2.53E-01	-1.15E-01	1.96E-01	-6.20E-03	1.40E-01	-1.20E-01	6.79E-02	-1.12E-02	1.72E-02	-1.20E-01
218	970	261	986	306	988	344	967	372	932	414	922	3.44E-01	-1.04E-01	3.00E-01	8.62E-03	2.48E-01	-1.20E-01	1.91E-01	-5.29E-03	1.30E-01	-1.25E-01	7.21E-02	-3.64E-03	1.04E-02	-1.22E-01
235	967	282	979	331	984	377	978	400	936	430	901	3.44E-01	-9.40E-02	2.99E-01	5.53E-03	2.44E-01	-1.10E-01	2.00E-01	-7.04E-03	1.27E-01	-1.16E-01	6.97E-02	-8.11E-03	1.83E-02	-1.12E-01
266	737	296	750	329	752	354	730	378	707	410	699	3.57E-01	-1.03E-01	3.08E-01	1.65E-03	2.45E-01	-1.11E-01	1.93E-01	-8.50E-03	1.27E-01	-1.18E-01	6.44E-02	-7.67E-03	-6.27E-04	-1.16E-01
288	753	323	765	360	769	395	759	407	724	440	707	3.50E-01	-1.08E-01	3.05E-01	1.42E-02	2.49E-01	-1.23E-01	1.95E-01	-1.05E-03	1.37E-01	-1.31E-01	6.75E-02	-4.68E-03	1.20E-02	-1.24E-01
530	1042	583	1056	637	1063	673	1041	702	995	745	963	3.48E-01	-1.12E-01	2.99E-01	1.80E-02	2.47E-01	-1.18E-01	1.90E-01	3.40E-04	1.36E-01	-1.25E-01	7.17E-02	-3.36E-03	1.70E-02	-1.22E-01
161	996	204	1006	248	1004	289	986	322	956	364	941	3.45E-01	-1.06E-01	2.98E-01	5.27E-03	2.50E-01	-1.24E-01	1.91E-01	-3.14E-03	1.36E-01	-1.29E-01	7.21E-02	-2.40E-03	1.83E-02	-1.23E-01
232	1079	274	1086	317	1087	359	1085	391	1058	426	1034	3.60E-01	-1.00E-01	3.10E-01	1.20E-02	2.47E-01	-1.15E-01	1.94E-01	1.12E-03	1.31E-01	-1.21E-01	6.45E-02	-2.25E-03	4.20E-04	-1.18E-01
316	1129	359	1129	401	1124	429	1094	458	1064	495	1043	3.43E-01	-1.00E-01	2.96E-01	5.28E-03	2.53E-01	-1.19E-01	1.91E-01	-4.52E-03	1.47E-01	-1.29E-01	7.23E-02	-9.62E-03	1.67E-02	-1.30E-01
203	1075	244	1093	288	1098	316	1071	339	1033	375	1009	3.31E-01	-1.11E-01	3.03E-01	2.60E-03	2.36E-01	-1.16E-01	1.99E-01	-1.01E-03	1.26E-01	-1.18E-01	7.15E-02	3.63E-04	3.32E-03	-1.16E-01
200	755	247	769	295	773	337	748	377	717	421	695	3.37E-01	-1.06E-01	2.89E-01	6.39E-03	2.44E-01	-1.13E-01	1.89E-01	1.25E-03	1.38E-01	-1.22E-01	7.57E-02	-4.95E-03	2.51E-02	-1.21E-01
256	1098	296	1103	335	1090	366	1064	402	1044	442	1035	3.48E-01	-1.05E-01	3.05E-01	6.45E-03	2.49E-01	-1.21E-01	1.89E-01	-1.04E-03	1.38E-01	-1.30E-01	6.77E-02	-3.23E-03	1.38E-02	-1.22E-01
243	1018	285	1024	327	1028	369	1032	392	996	431	979	3.57E-01	-9.77E-02	3.08E-01	1.15E-02	2.53E-01	-1.11E-01	1.91E-01	2.50E-04	1.37E-01	-1.22E-01	6.67E-02	-5.21E-03	2.20E-03	-1.17E-01
185	783	232	795	279	786	322	763	366	744	415	736	3.44E-01	-9.66E-02	3.02E-01	3.21E-03	2.59E-01	-1.14E-01	1.96E-01	-7.04E-03	1.33E-01	-1.27E-01	6.18E-02	-2.70E-03	9.14E-03	-1.15E-01
220	1028	258	1046	299	1046	335	1024	372	1003	412	995	3.34E-01	-1.21E-01	3.05E-01	7.46E-03	2.40E-01	-1.31E-01	1.99E-01	4.39E-03	1.28E-01	-1.29E-01	7.37E-02	7.72E-03	2.18E-03	-1.13E-01
232	1031	272	1037	312	1027	348	1004	382	980	419	965	3.52E-01	-1.06E-01	2.98E-01	6.58E-03	2.46E-01	-1.25E-01	1.93E-01	-8.69E-03	1.29E-01	-1.28E-01	7.44E-02	-1.86E-03	1.64E-02	-1.22E-01
158	926	201	950	247	967	288	949	314	909	360	892	3.44E-01	-1.06E-01	2.96E-01	6.09E-03	2.44E-01	-1.23E-01	1.87E-01	-3.71E-03	1.31E-01	-1.25E-01	7.28E-02	-4.82E-03	7.67E-03	-1.19E-01
192	786	244	801	297	803	337	772	361	725	407	699	3.50E-01	-1.11E-01	2.99E-01	1.03E-02	2.52E-01	-1.27E-01	1.85E-01	-1.73E-03	1.39E-01	-1.24E-01	6.43E-02	-4.05E-03	2.38E-02	-1.12E-01
221	1067	271	1085	323	1092	366	1068	381	1018	421	988	3.36E-01	-1.08E-01	3.04E-01	6.92E-03	2.46E-01	-1.11E-01	1.96E-01	-3.17E-03	1.38E-01	-1.18E-01	6.47E-02	-1.36E-03	1.07E-02	-1.14E-01
192	700	240	717	289	724	326	698	360	660	407	642	3.42E-01	-1.06E-01	2.95E-01	4.42E-03	2.41E-01	-1.16E-01	1.83E-01	-2.00E-03	1.38E-01	-1.24E-01	6.97E-02	-8.44E-03	2.91E-02	-1.14E-01
350	764	386	785	424	800	449	782	462	744	496	723	3.60E-01	-1.16E-01	3.06E-01	8.23E-03	2.46E-01	-1.33E-01	1.82E-01	-4.89E-03	1.25E-01	-1.35E-01	5.68E-02	-1.71E-04	5.39E-03	-1.23E-01
359	757	392	775	429	785	446	758	475	734	511	725	3.37E-01	-1.14E-01	3.02E-01	6.47E-03	2.42E-01	-1.27E-01	1.97E-01	-3.87E-03	1.31E-01	-1.33E-01	6.37E-02	-3.16E-03	1.55E-02	-1.19E-01
332	727	367	749	409	756	444	746	471	717	509	701	3.42E-01	-1.15E-01	3.03E-01	2.54E-03	2.45E-01	-1.31E-01	1.86E-01	-6.66E-03	1.33E-01	-1.30E-01	6.66E-02	-2.14E-03	1.65E-02	-1.23E-01
415	708	446	720	478	724	508	711	535	691	568	687	3.53E-01	-1.17E-01	2.99E-01	1.22E-02	2.50E-01	-1.33E-01	1.84E-01	-5.05E-03	1.37E-01	-1.38E-01	6.67E-02	-1.49E-03	1.59E-02	-1.30E-01
376	735	416	747	457	753	487	733	510	700	549	689	3.44E-01	-1.15E-01	2.92E-01	3.18E-03	2.36E-01	-1.27E-01	1.91E-01	1.14E-03	1.31E-01	-1.25E-01	7.48E-02	5.76E-03	2.41E-02	-1.18E-01
361	780	394	792	428	801	459	795	469	766	502	753	3.72E-01	-1.01E-01	2.98E-01	1.09E-02	2.57E-01	-1.36E-01	1.79E-01	2.17E-03	1.28E-01	-1.42E-01	6.27E-02	-5.79E-03	5.83E-03	-1.29E-01
328	765	366	775	405	778	440	769	462	737	498	723	3.60E-01	-9.86E-02	2.92E-01	4.62E-03	2.48E-01	-1.27E-01	1.86E-01	4.22E-03	1.27E-01	-1.33E-01	6.74E-02	1.62E-03	1.20E-02	-1.26E-01
396	708	430	728	466	744	502	741	537	723	575	713	3.53E-01	-1.25E-01	2.99E-01	-4.34E-03	2.40E-01	-1.30E-01	1.85E-01	-1.04E-03	1.35E-01	-1.30E-01	6.84E-02	5.79E-03	1.64E-02	-1.22E-01
398	725	436	743	475	754	516	757	526	727	562	711	3.43E-01	-1.19E-01	3.00E-01	6.59E-03	2.34E-01	-1.28E-01	1.90E-01	3.53E-03	1.29E-01	-1.23E-01	7.15E-02	3.49E-03	1.87E-02	-1.25E-01
334	700	367	713	401	719	435	722	450	695	482	684	3.54E-01	-1.15E-01	3.03E-01	1.53E-02	2.45E-01	-1.29E-01	1.89E-01	-1.29E-03	1.32E-01	-1.34E-01	6.33E-02	-1.99E-03	1.25E-02	-1.29E-01
405	747	443	760	483	761	493	733	493	697	519	667	3.58E-01	-1.03E-01	2.97E-01	8.77E-03	2.47E-01	-1.25E-01	1.84E-01	-1.83E-03	1.36E-01	-1.30E-01	6.84E-02	-9.66E-04	1.59E-02	-1.25E-01
395	703	424	712	454	718	481	705	505	686	534	678	3.49E-01	-1.23E-01	2.98E-01	4.67E-03	2.35E-01	-1.38E-01	1.88E-01	1.07E-02	1.22E-01	-1.34E-01	7.11E-02	7.92E-03	1.26E-02	-1.26E-01
417	716	447	726	478	734	507	734	526	713	556	708	3.65E-01	-1.21E-01	3.02E-01	9.10E-03	2.56E-01	-1.33E-01	1.80E-01	-2.91E-03	1.32E-01	-1.39E-01	5.37E-02	1.90E-03	1.37E-02	-1.34E-01
350	803	390	816	433	822	464	796	486	760	524	745	3.45E-01	-1.13E-01	2.94E-01	8.74E-03	2.46E-01	-1.21E-01	1.90E-01	3.36E-03	1.40E-01	-1.29E-01	7.71E-02	-3.09E-03	2.77E-02	-1.29E-01
399	754	436	775	478	784	515	769	530	730	555	700	3.62E-01	-1.01E-01	2.94E-01	1.55E-03	2.52E-01	-1.31E-01	1.79E-01	-1.70E-03	1.29E-01	-1.35E-01	5.99E-02	5.45E-05	1.97E-02	-1.26E-01
373	719	415	729	458	734	490	717	506	678	546	662	3.51E-01	-1.09E-01	2.92E-01	1.25E-02	2.38E-01	-1.24E-01	1.97E-01	-1.17E-03	1.30E-01	-1.22E-01	7.56E-02	6.37E-04	2.23E-02	-1.21E-01
425	702	460	723	500	735	521	710	535	671	565	645	3.52E-01	-1.11E-01	3.00E-01	6.78E-03	2.43E-01	-1.28E-01	1.84E-01	4.00E-03	1.36E-01	-1.29E-01	6.81E-02	1.24E-03	1.08E-02	-1.22E-01
120	578	161	588	202	588	241	578	266	546	294	518	3.41E-01	-1.10E-01	2.91E-01	1.06E-02	2.41E-01	-1.23E-01	1.87E-01	-2.01E-03	1.38E-01	-1.22E-01	6.87E-02	-5.17E-03	2.96E-02	-1.19E-01
101	551	144	562	189	563	230	545	257	509	290	481	3.39E-01	-1.13E-01	2.94E-01	7.34E-03	2.49E-01	-1.17E-01	1.89E-01	-1.69E-04	1.37E-01	-1.17E-01	6.70E-02	1.76E-03	1.96E-02	-1.09E-01
105	545	139	565	178	572	218	573	248	551	264	515	3.41E-01	-1.16E-01	3.00E-01	-2.01E-04	2.43E-01	-1.24E-01	1.85E-01	-6.72E-03	1.38E-01	-1.21E-01	6.87E-02	-2.56E-03	2.97E-02	-1.17E-01
154	542	194	557	238	561	280	555	299	519	324	484	3.48E-01	-1.19E-01	2.91E-01	6.58E-03	2.52E-01	-1.25E-01	1.85E-01	-3.17E-03	1.41E-01	-1.23E-01	6.28E-02	-3.65E-03	2.95E-02	-1.20E-01
170	525	206	536	243	536	279	526	291	492	304	458	3.62E-01	-1.07E-01	2.84E-01	6.64E-03	2.52E-01	-1.25E-01	1.84E-01	-1.53E-02	1.33E-01	-1.20E-01	6.43E-02	-6.21E-03	2.66E-02	-1.19E-01
132	583	178	591	224	585	269	568	299	535	306	490	3.40E-01	-1.07E-01	2.94E-01	4.32E-03	2.42E-01	-1.18E-01	1.86E-01	-6.62E-03	1.36E-01	-1.16E-01	6.41E-02	2.79E-03	3.23E-02	-1.16E-01
178	528	213	545	253	550	291	539	312	506	326	468	3.48E-01	-1.16E-01	2.95E-01	1.43E-02	2.49E-01	-1.25E-01	1.87E-01	8.08E-04	1.31E-01	-1.24E-01	6.53E-02	-5.27E-03	1.02E-02	-1.21E-01
135	545	167	559	201	565	236	559	256	531	275	502	3.43E-01	-1.12E-01	2.98E-01	1.41E-02	2.46E-01	-1.26E-01	1.88E-01	-1.64E-03	1.33E-01	-1.22E-01	6.69E-02	-4.55E-03	2.20E-02	-1.17E-01
106	525	144	538	185	529	222	510	255	485	295	479	3.40E-01	-1.11E-01	2.91E-01	2.10E-03	2.47E-01	-1.23E-01	1.93E-01	-6.64E-03	1.39E-01	-1.20E-01	7.04E-02	-5.42E-03	3.41E-02	-1.15E-01
104	573	146	589	192	591	236	580	267	548	303	523	3.46E-01	-1.16E-01	2.93E-01	3.78E-03	2.52E-01	-1.27E-01	1.82E-01	-5.34E-03	1.48E-01	-1.29E-01	6.35E-02	2.10E-03	4.49E-02	-1.23E-01
109	513	145	526	183	531	221	522	249	497	284	484	3.45E-01	-1.15E-01	2.99E-01	7.58E-03	2.49E-01	-1.21E-01	1.89E-01	-5.20E-03	1.35E-01	-1.20E-01	6.29E-02	-3.38E-04	1.94E-02	-1.18E-01
98	521	138	539	181	546	225	544	257	520	282	484	3.27E-01	-1.21E-01	2.96E-01	5.60E-03	2.38E-01	-1.25E-01	1.91E-01	-3.75E-03	1.36E-01	-1.18E-01	6.90E-02	1.62E-03	2.69E-02	-1.16E-01
119	528	158	539	199	543	239	539	244	499	250	460	3.45E-01	-1.14E-01	2.95E-01	1.19E-02	2.43E-01	-1.24E-01	1.86E-01	1.34E-03	1.38E-01	-1.22E-01	6.46E-02	-1.89E-03	2.66E-02	-1.20E-01
134	532	176	540	218	541	260	537	279	503	305	474	3.38E-01	-1.20E-01	2.96E-01	9.33E-03	2.38E-01	-1.24E-01	1.92E-01	-5.03E-04	1.38E-01	-1.16E-01	6.66E-02	3.37E-03	2.65E-02	-1.15E-01
107	543	144	561	186	568	228	567	251	531	285	509	3.40E-01	-1.25E-01	3.04E-01	4.12E-03	2.37E-01	-1.26E-01	1.83E-01	-3.75E-03	1.27E-01	-1.23E-01	6.41E-02	1.00E-03	2.01E-02	-1.17E-01
116	542	160	551	206	552	250	548	272	512	303	481	3.46E-01	-1.08E-01	2.88E-01	6.60E-03	2.46E-01	-1.18E-01	1.90E-01	-3.85E-03	1.40E-01	-1.16E-01	7.13E-02	-8.01E-03	3.11E-02	-1.19E-01

99	499	141	508	185	511	228	506	250	470	284	444	3.42E-01	-1.15E-01	2.94E-01	1.15E-02	2.47E-01	-1.16E-01	1.87E-01	-2.06E-03	1.35E-01	-1.22E-01	6.60E-02	-5.65E-04	2.60E-02	-1.21E-01
162	528	200	540	239	547	276	534	301	503	337	490	3.40E-01	-1.17E-01	2.95E-01	9.80E-03	2.44E-01	-1.23E-01	1.85E-01	-6.33E-04	1.34E-01	-1.22E-01	7.01E-02	-4.54E-04	2.21E-02	-1.17E-01
86	559	127	567	168	572	209	571	237	541	265	511	3.45E-01	-1.11E-01	2.90E-01	9.24E-03	2.48E-01	-1.21E-01	1.86E-01	-4.18E-03	1.38E-01	-1.19E-01	6.73E-02	-1.70E-03	2.42E-02	-1.18E-01
145	524	184	533	224	535	261	522	284	489	316	466	3.42E-01	-1.06E-01	2.91E-01	1.23E-02	2.46E-01	-1.17E-01	1.90E-01	-3.94E-03	1.38E-01	-1.15E-01	7.07E-02	-2.15E-03	2.53E-02	-1.14E-01
106	598	151	613	198	613	244	602	273	568	285	523	3.39E-01	-1.11E-01	2.93E-01	5.75E-03	2.45E-01	-1.22E-01	1.84E-01	-4.67E-03	1.38E-01	-1.19E-01	6.83E-02	-7.20E-04	3.01E-02	-1.19E-01
140	509	174	524	210	531	247	526	273	504	288	470	3.34E-01	-1.22E-01	2.97E-01	6.01E-03	2.43E-01	-1.26E-01	1.87E-01	-9.24E-03	1.37E-01	-1.22E-01	6.84E-02	-1.16E-03	2.90E-02	-1.21E-01
112	549	160	560	209	561	258	556	289	522	299	474	3.37E-01	-1.17E-01	2.88E-01	8.32E-03	2.42E-01	-1.21E-01	1.85E-01	-1.86E-03	1.35E-01	-1.20E-01	6.38E-02	-9.56E-04	3.20E-02	-1.16E-01
159	512	198	523	238	525	277	517	306	492	325	456	3.46E-01	-1.13E-01	2.94E-01	9.62E-03	2.45E-01	-1.21E-01	1.88E-01	-2.34E-03	1.34E-01	-1.16E-01	6.91E-02	-2.97E-03	3.01E-02	-1.15E-01
135	532	175	542	215	547	254	539	279	507	306	477	3.43E-01	-1.11E-01	2.92E-01	9.53E-03	2.53E-01	-1.21E-01	1.87E-01	-3.91E-03	1.42E-01	-1.23E-01	6.01E-02	-2.73E-03	3.06E-02	-1.21E-01
136	521	178	536	223	540	266	529	294	495	327	469	3.37E-01	-1.23E-01	2.92E-01	5.10E-03	2.40E-01	-1.26E-01	1.85E-01	-2.27E-03	1.33E-01	-1.23E-01	6.92E-02	9.25E-04	2.68E-02	-1.20E-01
134	536	174	546	216	543	255	530	287	504	319	479	3.45E-01	-1.13E-01	2.97E-01	8.38E-03	2.47E-01	-1.21E-01	1.92E-01	-2.09E-03	1.38E-01	-1.19E-01	6.67E-02	-9.04E-04	2.36E-02	-1.11E-01
128	553	170	560	212	560	254	550	277	521	288	481	3.39E-01	-1.17E-01	2.95E-01	6.14E-03	2.40E-01	-1.25E-01	1.87E-01	7.19E-04	1.34E-01	-1.18E-01	7.70E-02	2.08E-03	1.93E-02	-1.17E-01
110	513	149	527	190	534	231	530	257	498	293	478	3.41E-01	-1.18E-01	2.95E-01	4.07E-03	2.45E-01	-1.23E-01	1.88E-01	-2.50E-03	1.39E-01	-1.23E-01	6.72E-02	-2.21E-03	2.97E-02	-1.18E-01
185	512	223	533	265	542	297	520	311	480	350	464	3.51E-01	-1.17E-01	2.95E-01	3.09E-03	2.44E-01	-1.20E-01	1.80E-01	-2.37E-04	1.37E-01	-1.15E-01	6.40E-02	1.48E-03	3.00E-02	-1.13E-01
150	520	189	534	230	538	269	526	278	486	309	461	3.49E-01	-1.12E-01	2.92E-01	3.98E-03	2.44E-01	-1.20E-01	1.85E-01	-5.34E-03	1.35E-01	-1.18E-01	6.93E-02	-1.18E-03	2.53E-02	-1.15E-01
96	501	142	517	191	518	238	505	268	469	293	427	3.41E-01	-1.13E-01	2.92E-01	6.74E-03	2.43E-01	-1.18E-01	1.77E-01	1.42E-03	1.46E-01	-1.20E-01	6.60E-02	-6.05E-03	3.34E-02	-1.18E-01
105	532	146	543	189	543	225	528	254	496	288	470	3.41E-01	-1.06E-01	2.94E-01	4.63E-03	2.46E-01	-1.15E-01	1.84E-01	-4.53E-03	1.39E-01	-1.15E-01	6.91E-02	-1.09E-02	3.19E-02	-1.12E-01
141	543	181	554	221	551	256	530	286	501	325	488	3.47E-01	-1.10E-01	2.90E-01	6.61E-03	2.50E-01	-1.20E-01	1.85E-01	-1.88E-03	1.43E-01	-1.22E-01	6.90E-02	-6.44E-03	3.44E-02	-1.21E-01
136	525	175	536	215	536	253	525	282	497	318	480	3.63E-01	-1.10E-01	2.97E-01	1.07E-02	2.43E-01	-1.19E-01	1.88E-01	-3.29E-03	1.33E-01	-1.22E-01	7.40E-02	-7.72E-03	2.35E-02	-1.21E-01
213	539	240	548	269	552	289	531	306	507	334	499	3.48E-01	-1.01E-01	3.00E-01	2.71E-03	2.50E-01	-1.22E-01	1.85E-01	-3.54E-03	1.39E-01	-1.33E-01	6.26E-02	-3.96E-03	2.29E-02	-1.29E-01
201	590	235	598	269	602	302	594	325	570	350	546	3.36E-01	-1.08E-01	3.06E-01	5.53E-03	2.50E-01	-1.24E-01	1.88E-01	-2.52E-03	1.42E-01	-1.26E-01	6.72E-02	-3.58E-03	2.74E-02	-1.17E-01
163	590	199	595	235	596	265	580	286	551	300	517	3.44E-01	-9.93E-02	2.90E-01	6.22E-03	2.49E-01	-1.18E-01	1.93E-01	-4.48E-03	1.46E-01	-1.23E-01	7.60E-02	-8.22E-03	2.91E-02	-1.20E-01
300	521	327	528	352	524	368	502	391	486	419	484	3.52E-01	-1.20E-01	2.98E-01	1.00E-02	2.53E-01	-1.34E-01	1.83E-01	4.07E-03	1.32E-01	-1.37E-01	6.25E-02	-1.59E-03	1.26E-02	-1.28E-01
218	581	250	593	284	600	313	587	327	555	353	534	3.34E-01	-1.17E-01	2.97E-01	7.85E-03	2.40E-01	-1.31E-01	1.95E-01	-5.22E-03	1.37E-01	-1.37E-01	7.77E-02	-4.85E-03	1.17E-02	-1.26E-01
203	595	232	609	263	605	284	581	305	557	335	548	3.51E-01	-1.12E-01	3.04E-01	5.85E-03	2.50E-01	-1.20E-01	1.85E-01	-1.99E-03	1.32E-01	-1.22E-01	7.01E-02	-2.03E-03	1.28E-02	-1.15E-01
185	503	215	506	244	493	269	474	296	459	327	454	3.49E-01	-1.10E-01	3.04E-01	9.86E-03	2.45E-01	-1.14E-01	2.00E-01	5.69E-03	1.29E-01	-1.13E-01	7.83E-02	-4.95E-03	8.88E-03	-1.09E-01
202	528	232	538	261	531	291	518	320	506	347	491	3.47E-01	-1.12E-01	3.00E-01	6.36E-03	2.47E-01	-1.26E-01	1.93E-01	4.98E-04	1.33E-01	-1.29E-01	7.43E-02	-1.49E-03	1.57E-02	-1.12E-01
178	570	209	577	240	580	263	566	275	537	301	518	3.44E-01	-1.05E-01	2.97E-01	1.09E-02	2.45E-01	-1.22E-01	1.87E-01	3.19E-03	1.33E-01	-1.30E-01	7.96E-02	-3.49E-03	2.22E-02	-1.25E-01
160	530	197	535	234	531	262	505	288	478	321	461	3.43E-01	-1.04E-01	2.92E-01	1.28E-02	2.45E-01	-1.17E-01	1.94E-01	-6.11E-03	1.33E-01	-1.23E-01	8.05E-02	-6.74E-03	2.40E-02	-1.16E-01
206	548	232	562	259	572	288	577	296	550	319	531	3.37E-01	-1.15E-01	2.96E-01	7.07E-03	2.45E-01	-1.24E-01	1.95E-01	-1.34E-03	1.36E-01	-1.32E-01	6.42E-02	-1.12E-03	1.75E-02	-1.28E-01
237	581	272	586	306	587	323	560	345	535	378	524	3.47E-01	-9.13E-02	3.02E-01	9.25E-03	2.52E-01	-1.21E-01	1.94E-01	-3.17E-03	1.34E-01	-1.27E-01	7.91E-02	-7.84E-03	9.74E-03	-1.17E-01
213	577	242	583	272	578	296	561	315	537	344	529	3.48E-01	-1.00E-01	3.00E-01	5.97E-03	2.48E-01	-1.21E-01	1.94E-01	2.84E-04	1.38E-01	-1.27E-01	7.35E-02	-2.07E-03	1.98E-02	-1.19E-01
228	501	259	514	293	518	321	506	345	483	376	469	3.47E-01	-1.10E-01	2.89E-01	4.34E-03	2.52E-01	-1.30E-01	1.86E-01	-4.37E-03	1.40E-01	-1.37E-01	7.50E-02	-8.55E-03	2.50E-02	-1.27E-01
199	516	231	527	263	534	292	526	305	496	334	481	3.49E-01	-1.20E-01	2.94E-01	1.00E-02	2.47E-01	-1.30E-01	1.80E-01	-3.87E-03	1.38E-01	-1.38E-01	7.16E-02	-8.74E-03	1.91E-02	-1.27E-01
180	567	207	576	234	578	261	574	280	554	299	534	3.43E-01	-1.15E-01	2.93E-01	1.21E-02	2.44E-01	-1.32E-01	1.89E-01	-2.39E-03	1.39E-01	-1.35E-01	7.20E-02	-1.17E-02	2.55E-02	-1.29E-01
228	480	253	485	276	477	299	466	321	454	345	445	3.56E-01	-9.78E-02	3.00E-01	1.13E-02	2.46E-01	-1.29E-01	1.95E-01	-3.46E-03	1.30E-01	-1.33E-01	7.59E-02	-6.03E-03	6.77E-03	-1.24E-01
212	531	239	541	266	538	287	517	309	499	335	485	3.39E-01	-1.06E-01	2.95E-01	1.92E-03	2.40E-01	-1.17E-01	1.91E-01	-4.33E-03	1.35E-01	-1.14E-01	7.48E-02	-2.60E-03	1.34E-02	-1.08E-01
220	527	248	532	273	523	293	502	316	485	343	476	3.41E-01	-1.02E-01	2.96E-01	1.57E-02	2.46E-01	-1.19E-01	2.01E-01	-1.98E-03	1.34E-01	-1.26E-01	8.10E-02	-1.02E-02	1.44E-02	-1.22E-01
182	601	215	616	250	627	282	618	299	586	333	575	3.33E-01	-1.26E-01	2.99E-01	1.30E-02	2.36E-01	-1.28E-01	1.87E-01	1.03E-03	1.33E-01	-1.26E-01	7.31E-02	8.42E-03	1.97E-02	-1.22E-01
183	568	212	576	242	582	272	581	280	556	306	541	3.46E-01	-9.51E-02	3.02E-01	8.83E-03	2.40E-01	-1.24E-01	1.93E-01	-7.35E-04	1.29E-01	-1.31E-01	6.54E-02	-6.80E-03	1.45E-02	-1.28E-01
203	598	236	606	269	603	297	584	322	563	350	550	3.40E-01	-1.10E-01	2.89E-01	7.20E-03	2.42E-01	-1.18E-01	1.94E-01	-7.54E-04	1.43E-01	-1.24E-01	6.97E-02	-3.16E-03	2.30E-02	-1.19E-01
223	514	247	527	275	533	303	537	314	520	337	503	3.56E-01	-1.11E-01	2.92E-01	1.09E-03	2.46E-01	-1.28E-01	1.81E-01	-2.91E-03	1.37E-01	-1.32E-01	6.51E-02	-2.31E-03	2.88E-03	-1.20E-01
220	557	248	561	276	565	301	557	322	540	350	534	3.38E-01	-1.13E-01	2.97E-01	4.43E-03	2.39E-01	-1.23E-01	1.95E-01	3.43E-03	1.30E-01	-1.27E-01	7.10E-02	-4.95E-04	1.97E-02	-1.18E-01
206	532	230	539	255	536	276	521	291	501	314	489	3.57E-01	-9.85E-02	3.04E-01	7.62E-03	2.48E-01	-1.19E-01	1.97E-01	-6.14E-03	1.25E-01	-1.21E-01	6.92E-02	-2.88E-03	4.66E-03	-1.13E-01
209	487	236	489	263	484	289	476	315	466	341	461	3.56E-01	-1.09E-01	2.97E-01	7.47E-03	2.47E-01	-1.26E-01	1.90E-01	4.83E-03	1.36E-01	-1.28E-01	7.51E-02	-4.07E-04	1.84E-02	-1.14E-01
173	649	198	653	223	652	246	646	254	623	273	607	3.48E-01	-1.06E-01	2.99E-01	1.37E-02	2.56E-01	-1.28E-01	1.91E-01	2.96E-04	1.44E-01	-1.39E-01	6.18E-02	-4.06E-03	1.37E-02	-1.39E-01
233	564	260	564	286	554	308	537	331	522	358	516	3.50E-01	-1.03E-01	2.91E-01	1.40E-03	2.59E-01	-1.26E-01	1.80E-01	-3.94E-03	1.56E-01	-1.37E-01	6.17E-02	-1.03E-02	3.49E-02	-1.28E-01
207	580	233	590	261	587	287	574	308	555	336	547	3.41E-01	-1.10E-01	2.97E-01	1.30E-02	2.46E-01	-1.24E-01	1.84E-01	8.75E-03	1.34E-01	-1.29E-01	6.60E-02	-2.93E-03	2.09E-02	-1.25E-01
222	594	247	600	264	586	282	567	305	558	330	555	3.54E-01	-1.04E-01	3.00E-01	1.17E-02	2.54E-01	-1.22E-01	1.94E-01	-4.30E-03	1.32E-01	-1.32E-01	6.50E-02	-3.79E-04	1.17E-02	-1.26E-01
404	738	441	749	479	747	509	729	530	697	565	684	3.49E-01	-9.75E-02	3.00E-01	1.33E-02	2.40E-01	-1.18E-01	1.85E-01	3.35E-03	1.21E-01	-1.25E-01	6.62E-02	-3.68E-03	7.63E-03	-1.21E-01
402	758	446	768	489	777	530	770	549	729	581	701	3.39E-01	-1.01E-01	2.98E-01	8.28E-03	2.31E-01	-1.10E-01	1.89E-01	-2.54E-03	1.23E-01	-1.19E-01	6.73E-02	1.47E-04	8.97E-03	-1.11E-01
393	754	435	765	477	776	519	774	528	737	537	699	3.41E-01	-1.05E-01	3.04E-01	1.94E-02	2.39E-01	-1.16E-01	1.87E-01	8.76E-03	1.22E-01	-1.28E-01	6.67E-02	-1.13E-03	4.27E-03	-1.23E-01
481	735	517	739	553	741	590	743	597	705	624	684	3.40E-01	-1.19E-01	2.97E-01	2.24E-02	2.34E-01	-1.26E-01	1.82E-01	7.56E-03	1.23E-01	-1.36E-01	7.23E-02	4.49E-03	1.64E-02	-1.31E-01
378	746	416	744	455	750	486	736	503	704	512	669	3.41E-01	-9.66E-02	3.03E-01	1.64E-02	2.53E-01	-1.13E-01	1.87E-01	4.42E-04	1.29E-01	-1.24E-01	7.26E-02	-6.41E-03	1.19E-02	-1.21E-01
397	791	439	794	481	793	519	776	530	744	549	716	3.49E-01	-9.70E-02	3.01E-01	1.58E-02	2.53E-01	-1.15E-01	1.89E-01	3.21E-03	1.29E-01	-1.19E-01	6.94E-02	-2.98E-03	8.75E-03	-1.20E-01
426	794	473	809	521	815	562	794	576	748	600	711	3.48E-01	-1.12E-01	2.99E-01	1.64E-02	2.43E-01	-1.23E-01	1.83E-01	9.12E-03	1.29E-01	-1.24E-01	6.95E-02	3.91E-03	8.45E-03	-1.18E-01
401	698	435	706	466	714	498	703	515	673	544	655	3.43E-01	-1.04E-01	2.97E-01	2.15E-02	2.42E-01	-1.15E-01	1.86E-01	3.60E-03	1.26E-01	-1.26E-01	7.27E-02	-1.70E-03	6.01E-03	-1.25E-01
422	797	458	807	497	807	525	790	543	756	574	734	3.45E-01	-1.07E-01	2.96E-01	1.34E-02	2.46E-01	-1.17E-01	1.85E-01	2.21E-03	1.27E-01	-1.24E-01	7.24E-02	1.76E-03	6.10E-03	-1.19E-01
406	758	447	770	491	775	528	765	542	724	577	701	3.64E-01	-1.04E-01	2.98E-01	1.83E-02	2.52E-01	-1.14E-01	1.80E-01	-5.60E-03	1.32E-01	-1.21E-01	5.47E-02	-5.95E-03	1.34E-02	-1.12E-01
375	741	411	757	450	761	488	752	507	720	532	694	3.46E-01	-1.11E-01	3.06E-01	1.44E-02	2.42E-01	-1.14E-01	1.87E-01	4.96E-03	1.20E-01	-1.13E-01	6.87E-02	7.25E-03	8.27E-03	-1.18E-01
435	741	473	753	513	756	549	745	578	718	607	692	3.48E-01	-1.10E-01	2.97E-01	8.18E-03	2.34E-01	-1.18E-01	1.82E-01	8.13E-06	1.18E-01	-1.26E-01	6.75E-02	-2.93E-03	1.94E-02	-1.28E-01
421	707	463	708	501	707	540	709	535	668	560	634	3.47E-01	-1.06E-01	2.92E-01	1.46E-02	2.48E-01	-1.18E-01	1.82E-01	1.01E-03	1.36E-01	-1.28E-01	6.68E-02	-6.56E-03	2.27E-02	-1.22E-01
363	706	401	712	439	708	471	686	495	656	531	644	3.47E-01	-1.06E-01	3.04E-01	1.80E-02	2.41E-01	-1.15E-01	1.83E-01	3.80E-03	1.26E-01	-1.25E-01	5.87E-02	5.16E-03	1.36E-02	-1.19E-01
476	772	521	779	561	776	590	744	606	703	643	680	3.53E-01	-1.04E-01	2.91E-01	1.89E-02	2.50E-01	-1.24E-01	1.83E-01	-2.03E-03	1.33E-01	-1.31E-01	6.66E-02	-6.78E-03	6.99E-03	-1.28E-01
428	742	471	750	513	752	546	728	557	687	590	662	3.57E-01	-9.46E-02	3.01E-01	1.82E-02	2.49E-01	-1.12E-01	1.85E-01	-4.17E-04	1.36E-01	-1.18E-01	6.63E-02	-3.48E-03	1.77E-02	-1.14E-01
415	723	448	737	482	748	509	733	530	704	561	691	3.40E-01	-1.29E-01	3.02E-01	9.43E-03	2.37E-01	-1.28E-01	1.80E-01	7.33E-03	1.22E-01	-1.29E-01	6.28E-02	6.96E-04	1.07E-02	-1.22E-01
445	783	483	792	524	789	558	773	574	737	603	712	3.47E-01	-1.10E-01	3.05E-01	2.03E-02	2.44E-01	-1.23E-01	1.91E-01	7.89E-03	1.19E-01	-1.28E-01	6.70E-02	3.38E-03	-6.70E-04	-1.20E-01
393	715	428	728	464	729	493	707	511	674	537	650	3.51E-01	-1.06E-01	2.93E-01	2.54E-02	2.39E-01	-1.21E-01	1.86E-01	4.53E-03	1.32E-01	-1.30E-01	6.07E-02	-3.39E-03	6.29E-03	-1.25E-01
405	731	441	739	477	741	513	736	529	706	559	686	3.44E-01	-1.11E-01	3.04E-01	2.15E-02	2.38E-01	-1.19E-01	1.83E-01	5.85E-03	1.21E-01	-1.21E-01	6.60E-02	2.36E-03	1.17E-02	-1.14E-01
361	763	407	774	454	778	494	758	516	717	548	686	3.41E-01	-1.05E-01	2.99E-01	1.21E-02	2.40E-01	-1.19E-01	1.89E-01	1.12E-03	1.27E-01	-1.20E-01	7.26E-02	-3.27E-03	1.69E-02	-1.17E-01
202	533	243	540	284	541	298	509	317	472	352	453	3.39E-01	-1.11E-01	2.91E-01	1.50E-02	2.39E-01	-1.31E-01	1.80E-01	9.85E-03	1.30E-01	-1.31E-01	6.68E-02	6.14E-03	2.48E-02	-1.29E-01
139	590	184	594	230	596	270	582	293	542	326	515	3.45E-01	-1.05E-01	2.93E-01	1.67E-02	2.48E-01	-1.17E-01	1.82E-01	5.82E-04	1.37E-01	-1.22E-01	6.92E-02	-4.23E-03	2.40E-02	-1.17E-01
180	581	221	584	261	585	275	550	290	513	325	492	3.45E-01	-1.03E-01	2.99E-01	1.60E-02	2.54E-01	-1.18E-01	1.84E-01	5.01E-03	1.37E-01	-1.27E-01	6.54E-02	-2.20E-03	1.12E-02	-1.16E-01
155	527	199	538	244	547	278	526	295	484	326	457	3.42E-01	-1.09E-01	3.01E-01	1.05E-02	2.41E-01	-1.24E-01	1.85E-01	8.62E-04	1.26E-01	-1.27E-01	7.20E-02	2.05E-03	1.84E-02	-1.17E-01
159	564	197	574	228	561	241	525	218	493	213	458	3.47E-01	-1.09E-01	3.05E-01	2.06E-02	2.52E-01	-1.21E-01	1.77E-01	1.15E-02	1.18E-01	-1.23E-01	5.99E-02	9.42E-03	-6.60E-04	-1.13E-01
177	595	222	602	267	606	290	578	306	535	338	506	3.54E-01	-1.01E-01	3.03E-01	1.32E-02	2.50E-01	-1.19E-01	1.85E-01	3.32E-03	1.35E-01	-1.26E-01	6.29E-02	-2.76E-03	1.73E-02	-1.24E-01
360	914	421	932	481	939	536	913	560	858	569	799	3.66E-01	-9.74E-02	2.96E-01	2.36E-02	2.51E-01	-1.23E-01	1.81E-01	3.47E-03	1.25E-01	-1.30E-01	6.21E-02	-6.54E-03	6.59E-03	-1.24E-01
394	1053	454	1077	515	1095	559	1060	592	1014	632	968	3.43E-01	-1.10E-01	2.99E-01	1.18E-02	2.47E-01	-1.23E-01	1.79E-01	3.47E-03	1.29E-01	-1.25E-01	6.18E-02	1.96E-03	1.32E-02	-1.23E-01
453	891	518	911	587	909	640	865	647	805	647	748	3.62E-01	-1.07E-01	2.92E-01	1.46E-02	2.47E-01	-1.23E-01	1.74E-01	5.48E-03	1.29E-01	-1.25E-01	5.73E-02	-2.12E-03	1.11E-02	-1.17E-01
369	864	434	876	501	883	553	851	566	788	595	735	3.39E-01	-1.01E-01	2.96E-01	1.43E-02	2.46E-01	-1.16E-01	1.82E-01	3.42E-03	1.30E-01	-1.20E-01	6.76E-02	-4.71E-03	1.53E-02	-1.17E-01
344	987	401	1012	462	1022	515	993	551	943	599	910	3.51E-01	-1.12E-01	2.97E-01	1.50E-02	2.48E-01	-1.22E-01	1.81E-01	4.02E-03	1.29E-01	-1.27E-01	5.95E-02	1.24E-03	1.66E-02	-1.23E-01
271	1111	347	1131	425	1134	493	1098	511	1026	517	951	3.52E-01	-1.03E-01	2.94E-01	1.93E-02	2.46E-01	-1.22E-01	1.79E-01	2.39E-03	1.33E-01	-1.27E-01	6.71E-02	-2.88E-03	2.06E-02	-1.21E-01
292	826	362	842	433	851	497	823	533	767	556	702	3.58E-01	-1.02E-01	2.99E-01	1.58E-02	2.53E-01	-1.20E-01	1.83E-01	4.10E-04	1.37E-01	-1.28E-01	6.44E-02	-2.87E-03	1.49E-02	-1.21E-01

272	1034	343	1052	416	1059	471	1026	516	968	575	929	3.55E-01	-1.03E-01	3.01E-01	1.29E-02	2.51E-01	-1.20E-01	1.86E-01	-2.47E-03	1.28E-01	-1.25E-01	5.94E-02	4.89E-04	7.16E-03	-1.13E-01
251	1094	323	1107	395	1107	453	1062	481	996	521	937	3.54E-01	-9.14E-02	3.01E-01	1.51E-02	2.54E-01	-1.15E-01	1.87E-01	-2.98E-03	1.41E-01	-1.20E-01	6.58E-02	-6.08E-03	2.12E-02	-1.18E-01
334	837	401	866	473	880	531	847	549	777	592	725	3.57E-01	-1.05E-01	3.03E-01	1.55E-02	2.57E-01	-1.21E-01	1.79E-01	1.06E-03	1.29E-01	-1.19E-01	6.20E-02	2.85E-03	9.60E-03	-1.17E-01
358	1069	417	1073	474	1057	525	1027	546	972	575	924	3.57E-01	-9.10E-02	3.03E-01	1.39E-02	2.54E-01	-1.14E-01	1.85E-01	1.36E-03	1.36E-01	-1.17E-01	6.52E-02	-5.85E-03	1.75E-02	-1.12E-01
308	982	367	1017	432	1034	493	1014	523	955	563	905	3.50E-01	-1.11E-01	3.00E-01	8.28E-03	2.41E-01	-1.26E-01	1.82E-01	4.87E-03	1.26E-01	-1.27E-01	6.15E-02	7.18E-03	7.76E-03	-1.20E-01
263	786	329	800	396	795	457	770	502	720	558	685	3.57E-01	-9.66E-02	3.02E-01	1.35E-02	2.54E-01	-1.17E-01	1.90E-01	2.98E-03	1.34E-01	-1.23E-01	6.95E-02	-2.76E-03	1.14E-02	-1.18E-01
305	1043	377	1061	447	1052	499	1000	519	929	568	880	3.57E-01	-9.44E-02	3.04E-01	1.48E-02	2.50E-01	-1.21E-01	1.85E-01	2.36E-03	1.32E-01	-1.27E-01	6.36E-02	-4.19E-03	1.22E-02	-1.20E-01
274	1082	343	1095	414	1098	466	1056	510	1006	546	947	3.58E-01	-9.97E-02	3.02E-01	1.49E-02	2.54E-01	-1.15E-01	1.86E-01	-1.73E-03	1.29E-01	-1.15E-01	6.43E-02	-3.82E-03	7.48E-03	-1.09E-01
285	1084	360	1092	435	1103	494	1077	514	1006	567	956	3.54E-01	-1.09E-01	2.94E-01	1.37E-02	2.50E-01	-1.25E-01	1.83E-01	6.05E-04	1.33E-01	-1.29E-01	6.76E-02	7.69E-03	2.77E-02	-1.23E-01
333	1101	404	1106	474	1094	518	1040	536	971	594	935	3.60E-01	-1.02E-01	3.00E-01	1.61E-02	2.52E-01	-1.15E-01	1.87E-01	-3.78E-04	1.38E-01	-1.26E-01	6.34E-02	1.43E-03	6.56E-03	-1.21E-01
266	1015	349	1037	435	1050	506	1023	538	945	587	878	3.53E-01	-1.11E-01	2.92E-01	1.28E-02	2.57E-01	-1.24E-01	1.79E-01	3.85E-03	1.42E-01	-1.35E-01	6.47E-02	-1.71E-03	1.74E-02	-1.30E-01
264	923	347	951	433	965	515	943	557	870	607	805	3.54E-01	-1.01E-01	2.94E-01	1.72E-02	2.49E-01	-1.19E-01	1.81E-01	3.88E-03	1.25E-01	-1.25E-01	6.77E-02	4.01E-03	1.13E-02	-1.19E-01
236	1081	312	1089	389	1092	445	1044	488	983	563	971	3.54E-01	-1.11E-01	2.99E-01	1.28E-02	2.45E-01	-1.24E-01	1.86E-01	8.78E-03	1.34E-01	-1.25E-01	6.82E-02	7.21E-03	1.46E-02	-1.25E-01
254	1111	329	1124	404	1134	452	1091	461	1016	526	980	3.46E-01	-1.09E-01	3.05E-01	2.57E-02	2.48E-01	-1.20E-01	1.83E-01	1.15E-02	1.33E-01	-1.25E-01	6.86E-02	4.00E-03	1.29E-02	-1.20E-01
450	739	489	741	530	736	565	718	586	684	612	658	3.64E-01	-9.65E-02	2.97E-01	1.74E-02	2.46E-01	-1.19E-01	1.80E-01	4.40E-03	1.34E-01	-1.25E-01	6.34E-02	-7.88E-03	1.53E-02	-1.22E-01
153	548	198	562	244	571	281	561	272	519	308	490	3.48E-01	-1.13E-01	2.97E-01	1.76E-02	2.47E-01	-1.20E-01	1.81E-01	4.20E-03	1.35E-01	-1.28E-01	6.04E-02	-3.68E-03	1.66E-02	-1.26E-01
168	535	209	550	253	558	287	540	286	495	307	459	3.49E-01	-1.10E-01	2.98E-01	1.25E-02	2.50E-01	-1.23E-01	1.75E-01	4.81E-03	1.38E-01	-1.26E-01	8.43E-02	-1.21E-02	2.20E-02	-1.22E-01
140	623	184	634	228	645	269	634	276	590	282	546	3.63E-01	-1.08E-01	2.95E-01	1.61E-02	2.57E-01	-1.33E-01	1.80E-01	2.81E-03	1.34E-01	-1.31E-01	6.04E-02	9.91E-04	1.62E-02	-1.26E-01
143	560	189	569	236	576	278	566	299	524	323	485	3.59E-01	-1.08E-01	2.92E-01	1.88E-02	2.51E-01	-1.28E-01	1.80E-01	1.70E-03	1.30E-01	-1.31E-01	6.27E-02	-3.07E-03	1.51E-02	-1.30E-01
264	778	337	801	412	816	486	803	511	737	512	663	3.53E-01	-1.07E-01	2.99E-01	1.31E-02	2.45E-01	-1.24E-01	1.83E-01	7.72E-03	1.27E-01	-1.25E-01	6.56E-02	3.98E-03	1.74E-02	-1.18E-01
307	1103	379	1128	455	1139	522	1112	526	1043	504	970	3.53E-01	-9.57E-02	2.99E-01	1.88E-02	2.55E-01	-1.18E-01	1.86E-01	5.13E-03	1.34E-01	-1.22E-01	6.94E-02	3.13E-03	2.59E-02	-1.24E-01
251	1104	327	1111	404	1111	474	1087	517	1023	579	983	3.41E-01	-1.09E-01	3.00E-01	1.42E-02	2.46E-01	-1.18E-01	1.88E-01	5.69E-03	1.29E-01	-1.19E-01	7.25E-02	3.88E-03	1.18E-02	-1.14E-01
379	811	451	822	519	842	571	809	601	743	658	703	3.50E-01	-1.04E-01	2.96E-01	1.37E-02	2.47E-01	-1.15E-01	1.84E-01	3.42E-03	1.32E-01	-1.21E-01	7.28E-02	5.69E-03	1.59E-02	-1.18E-01
324	974	393	1000	465	1015	530	1003	546	937	528	873	3.53E-01	-1.01E-01	3.02E-01	1.46E-02	2.50E-01	-1.13E-01	1.87E-01	8.16E-03	1.33E-01	-1.20E-01	6.92E-02	-2.68E-03	1.35E-02	-1.20E-01
458	755	490	765	524	769	557	768	578	742	605	721	3.48E-01	-1.08E-01	3.06E-01	1.08E-02	2.46E-01	-1.17E-01	1.87E-01	2.82E-03	1.29E-01	-1.25E-01	5.93E-02	-4.94E-04	1.13E-02	-1.16E-01
414	766	433	772	464	774	496	776	517	753	547	744	3.51E-01	-1.02E-01	3.11E-01	1.44E-02	2.38E-01	-1.10E-01	1.92E-01	3.42E-04	1.19E-01	-1.15E-01	6.37E-02	-1.78E-04	1.31E-03	-1.08E-01
442	739	478	749	516	755	551	746	576	717	610	703	3.43E-01	-1.11E-01	3.05E-01	1.46E-02	2.45E-01	-1.16E-01	1.86E-01	6.40E-03	1.27E-01	-1.20E-01	6.19E-02	-1.16E-03	1.29E-02	-1.09E-01
127	518	164	533	203	544	242	544	278	526	316	512	3.48E-01	-1.19E-01	3.12E-01	1.27E-02	2.41E-01	-1.13E-01	1.82E-01	2.66E-03	1.27E-01	-1.24E-01	6.21E-02	6.49E-03	1.86E-03	-1.16E-01
93	490	134	501	177	510	218	503	251	475	292	462	3.59E-01	-1.02E-01	3.06E-01	1.25E-02	2.47E-01	-1.19E-01	1.83E-01	-5.38E-04	1.29E-01	-1.28E-01	6.63E-02	-5.47E-03	9.25E-03	-1.22E-01
91	587	136	604	184	611	232	607	275	589	305	551	3.53E-01	-1.11E-01	2.98E-01	8.62E-03	2.48E-01	-1.25E-01	1.77E-01	-3.17E-03	1.34E-01	-1.30E-01	6.08E-02	-2.37E-03	1.53E-02	-1.21E-01
173	555	210	568	248	580	287	589	314	562	350	549	3.45E-01	-1.15E-01	2.99E-01	1.20E-02	2.46E-01	-1.22E-01	1.87E-01	-2.61E-03	1.28E-01	-1.27E-01	6.10E-02	-7.10E-03	8.96E-03	-1.21E-01
127	597	161	610	198	611	235	605	271	596	307	590	3.59E-01	-1.07E-01	3.05E-01	1.24E-02	2.48E-01	-1.18E-01	1.84E-01	8.40E-04	1.28E-01	-1.29E-01	5.84E-02	-4.26E-03	7.96E-03	-1.23E-01
125	557	164	568	204	574	244	575	271	546	307	530	3.55E-01	-1.02E-01	3.00E-01	1.24E-02	2.56E-01	-1.11E-01	1.87E-01	-3.09E-03	1.38E-01	-1.26E-01	6.24E-02	-4.66E-03	2.20E-02	-1.20E-01
125	607	163	624	205	632	246	629	284	614	317	588	3.56E-01	-1.11E-01	2.99E-01	1.21E-02	2.54E-01	-1.28E-01	1.82E-01	-2.95E-03	1.35E-01	-1.36E-01	5.80E-02	-3.80E-03	1.26E-02	-1.28E-01
145	540	182	558	223	569	261	562	286	528	320	507	3.62E-01	-1.09E-01	3.04E-01	1.03E-02	2.48E-01	-1.25E-01	1.82E-01	-1.81E-03	1.29E-01	-1.26E-01	5.86E-02	1.77E-03	1.41E-02	-1.22E-01
140	573	177	583	216	590	247	581	274	556	312	548	3.51E-01	-1.11E-01	3.04E-01	9.61E-03	2.48E-01	-1.18E-01	1.85E-01	-1.89E-03	1.32E-01	-1.26E-01	6.40E-02	1.10E-03	1.22E-02	-1.16E-01
121	565	160	586	203	595	246	589	280	561	318	540	3.49E-01	-1.16E-01	3.04E-01	7.29E-03	2.44E-01	-1.23E-01	1.80E-01	1.80E-03	1.28E-01	-1.30E-01	6.06E-02	5.30E-04	7.42E-03	-1.23E-01
119	563	157	584	199	595	242	591	272	560	311	542	3.56E-01	-1.12E-01	2.97E-01	1.15E-02	2.52E-01	-1.25E-01	1.78E-01	1.47E-03	1.30E-01	-1.31E-01	6.42E-02	-1.67E-03	1.50E-02	-1.26E-01
89	579	133	590	178	594	216	571	253	545	298	541	3.64E-01	-1.05E-01	2.97E-01	1.41E-02	2.54E-01	-1.19E-01	1.82E-01	-4.47E-03	1.27E-01	-1.29E-01	6.54E-02	-3.61E-03	1.42E-02	-1.23E-01
443	840	489	859	537	872	581	854	625	832	674	823	3.46E-01	-1.14E-01	3.06E-01	8.25E-03	2.46E-01	-1.19E-01	1.84E-01	1.65E-03	1.23E-01	-1.26E-01	6.49E-02	2.39E-05	3.33E-03	-1.15E-01
220	1021	287	1053	358	1070	430	1064	493	1028	555	991	3.46E-01	-9.40E-02	3.06E-01	8.73E-03	2.43E-01	-1.24E-01	1.84E-01	-2.47E-03	1.30E-01	-1.29E-01	5.98E-02	6.36E-05	1.12E-02	-1.19E-01
288	993	349	1024	416	1041	485	1038	530	993	585	954	3.62E-01	-1.06E-01	3.06E-01	7.15E-03	2.42E-01	-1.25E-01	1.78E-01	-1.64E-03	1.29E-01	-1.29E-01	5.33E-02	3.41E-03	9.79E-03	-1.19E-01
236	1020	295	1053	362	1063	430	1063	496	1050	559	1026	3.52E-01	-1.08E-01	3.07E-01	1.32E-02	2.43E-01	-1.27E-01	1.78E-01	1.72E-03	1.28E-01	-1.30E-01	5.80E-02	2.90E-03	1.42E-02	-1.24E-01
344	1059	392	1085	446	1090	499	1078	546	1051	595	1030	3.58E-01	-1.05E-01	3.09E-01	1.07E-02	2.43E-01	-1.21E-01	1.83E-01	-5.28E-03	1.32E-01	-1.26E-01	5.97E-02	-1.29E-03	5.95E-03	-1.11E-01
312	852	384	870	458	877	516	831	545	763	604	724	3.61E-01	-9.88E-02	3.00E-01	1.34E-02	2.53E-01	-1.19E-01	1.83E-01	-7.28E-04	1.35E-01	-1.23E-01	6.53E-02	-1.73E-03	1.14E-02	-1.15E-01
321	1049	394	1073	468	1084	533	1058	568	991	626	944	3.57E-01	-1.00E-01	2.97E-01	1.34E-02	2.54E-01	-1.21E-01	1.83E-01	4.88E-04	1.30E-01	-1.30E-01	6.46E-02	5.42E-04	9.06E-03	-1.23E-01
315	1175	386	1192	459	1197	526	1173	550	1107	592	1051	3.55E-01	-9.62E-02	2.99E-01	1.57E-02	2.48E-01	-1.15E-01	1.90E-01	6.49E-04	1.28E-01	-1.22E-01	6.51E-02	-9.14E-03	1.65E-02	-1.20E-01
432	762	477	776	522	787	559	764	557	720	566	679	3.49E-01	-1.15E-01	3.03E-01	1.59E-02	2.45E-01	-1.21E-01	1.74E-01	6.54E-03	1.22E-01	-1.28E-01	5.53E-02	-6.07E-04	5.25E-04	-1.23E-01
426	799	466	806	506	807	536	788	552	751	578	721	3.50E-01	-1.02E-01	3.02E-01	1.92E-02	2.39E-01	-1.09E-01	1.82E-01	1.42E-04	1.25E-01	-1.22E-01	6.44E-02	-1.29E-02	-2.76E-03	-1.11E-01
425	782	465	794	507	795	541	778	557	739	585	711	3.60E-01	-1.11E-01	2.98E-01	1.36E-02	2.52E-01	-1.23E-01	1.79E-01	-2.70E-04	1.26E-01	-1.29E-01	5.76E-02	-3.67E-03	1.24E-03	-1.18E-01
396	760	441	773	487	777	528	768	531	721	552	683	3.56E-01	-1.04E-01	2.99E-01	1.58E-02	2.48E-01	-1.16E-01	1.83E-01	2.54E-03	1.26E-01	-1.24E-01	5.90E-02	-4.74E-03	-2.26E-03	-1.16E-01
391	709	432	722	474	729	507	708	524	673	538	635	3.57E-01	-1.09E-01	2.95E-01	1.79E-02	2.50E-01	-1.21E-01	1.84E-01	-2.82E-04	1.23E-01	-1.25E-01	6.27E-02	-5.87E-03	5.70E-03	-1.18E-01
404	816	450	829	497	825	534	796	536	748	546	705	3.62E-01	-9.89E-02	3.00E-01	2.17E-02	2.53E-01	-1.17E-01	1.77E-01	-3.57E-03	1.30E-01	-1.26E-01	5.43E-02	-5.39E-03	-1.96E-03	-1.13E-01
402	747	445	767	493	764	531	741	542	695	560	657	3.61E-01	-1.14E-01	2.94E-01	1.64E-02	2.49E-01	-1.19E-01	1.71E-01	-1.67E-03	1.26E-01	-1.29E-01	5.51E-02	-5.89E-03	6.28E-03	-1.23E-01
422	766	462	776	504	779	539	763	558	725	588	698	3.72E-01	-1.02E-01	2.94E-01	2.36E-02	2.56E-01	-1.16E-01	1.78E-01	-1.05E-03	1.37E-01	-1.30E-01	6.07E-02	-1.72E-02	5.59E-03	-1.24E-01
384	749	425	750	467	751	506	741	524	704	557	681	3.58E-01	-9.65E-02	2.93E-01	1.83E-02	2.53E-01	-1.12E-01	1.84E-01	-6.42E-04	1.34E-01	-1.22E-01	6.17E-02	-1.52E-02	3.48E-03	-1.16E-01
453	711	487	720	520	726	554	726	568	694	594	672	3.52E-01	-1.04E-01	3.03E-01	1.68E-02	2.49E-01	-1.15E-01	1.79E-01	2.51E-03	1.34E-01	-1.27E-01	5.90E-02	-7.95E-03	8.79E-03	-1.21E-01
476	681	511	687	547	692	582	690	595	657	620	634	3.54E-01	-1.01E-01	3.02E-01	1.39E-02	2.46E-01	-1.13E-01	1.81E-01	3.93E-03	1.34E-01	-1.27E-01	5.95E-02	-1.36E-02	8.17E-03	-1.21E-01
388	806	431	817	474	819	515	809	526	768	538	731	3.54E-01	-1.01E-01	2.97E-01	1.80E-02	2.51E-01	-1.23E-01	1.79E-01	-6.54E-05	1.26E-01	-1.27E-01	6.21E-02	-7.60E-03	7.13E-03	-1.17E-01
400	714	437	719	475	718	512	717	532	687	555	661	3.53E-01	-1.10E-01	3.05E-01	1.66E-02	2.50E-01	-1.20E-01	1.79E-01	6.39E-03	1.26E-01	-1.22E-01	6.36E-02	-2.28E-03	6.34E-03	-1.15E-01
396	716	433	725	470	730	507	732	528	705	558	683	3.56E-01	-1.10E-01	3.04E-01	1.48E-02	2.48E-01	-1.19E-01	1.80E-01	1.16E-03	1.29E-01	-1.20E-01	5.87E-02	-3.08E-03	-2.08E-03	-1.11E-01
429	766	471	762	514	759	558	765	567	736	583	698	3.50E-01	-1.01E-01	2.98E-01	1.61E-02	2.46E-01	-1.15E-01	1.81E-01	7.44E-03	1.29E-01	-1.18E-01	6.51E-02	-5.20E-03	9.37E-03	-1.11E-01
386	1071	464	1076	542	1076	600	1037	600	960	639	898	3.53E-01	-1.06E-01	2.90E-01	1.94E-02	2.63E-01	-1.20E-01	1.81E-01	-2.82E-03	1.41E-01	-1.29E-01	6.15E-02	-6.49E-03	1.89E-02	-1.23E-01
207	1091	267	1112	331	1120	392	1109	447	1075	506	1051	3.53E-01	-9.97E-02	3.08E-01	1.13E-02	2.53E-01	-1.16E-01	1.82E-01	-1.82E-03	1.33E-01	-1.25E-01	5.82E-02	-2.92E-03	1.16E-02	-1.17E-01
420	766	451	777	482	778	502	757	526	735	557	724	3.80E-01	-1.01E-01	3.01E-01	1.69E-02	2.53E-01	-1.22E-01	1.81E-01	-2.55E-03	1.35E-01	-1.33E-01	6.10E-02	-3.26E-03	2.95E-02	-1.30E-01
398	745	429	754	460	759	490	752	511	730	542	722	3.66E-01	-1.07E-01	2.99E-01	1.29E-02	2.51E-01	-1.22E-01	1.83E-01	-1.54E-03	1.32E-01	-1.27E-01	6.38E-02	-1.43E-03	8.84E-03	-1.19E-01
393	710	425	721	460	724	491	712	514	687	543	672	3.65E-01	-1.06E-01	2.93E-01	1.41E-02	2.54E-01	-1.19E-01	1.84E-01	-3.24E-03	1.31E-01	-1.32E-01	6.14E-02	-3.65E-03	1.55E-02	-1.25E-01
446	734	476	744	509	746	539	738	567	724	597	714	3.50E-01	-1.12E-01	3.03E-01	1.55E-02	2.44E-01	-1.19E-01	1.83E-01	-2.32E-03	1.26E-01	-1.19E-01	6.50E-02	7.70E-03	1.85E-02	-1.15E-01
394	729	429	746	465	757	501	751	532	728	566	711	3.61E-01	-1.03E-01	3.04E-01	1.54E-02	2.55E-01	-1.16E-01	1.85E-01	-5.30E-03	1.43E-01	-1.24E-01	6.08E-02	-3.98E-03	4.58E-03	-1.14E-01
355	746	388	763	424	775	456	755	482	729	512	711	3.64E-01	-1.13E-01	3.00E-01	1.73E-02	2.54E-01	-1.19E-01	1.79E-01	-1.58E-03	1.33E-01	-1.26E-01	6.23E-02	-2.85E-03	1.07E-02	-1.19E-01
425	749	451	760	478	768	504	756	529	743	556	734	3.58E-01	-1.05E-01	3.04E-01	1.83E-02	2.49E-01	-1.21E-01	1.87E-01	-2.32E-03	1.21E-01	-1.24E-01	6.36E-02	4.56E-03	7.59E-03	-1.19E-01
439	753	471	768	505	780	540	776	549	741	564	711	3.62E-01	-9.71E-02	3.11E-01	1.83E-02	2.52E-01	-1.18E-01	1.85E-01	-3.39E-03	1.31E-01	-1.28E-01	6.26E-02	-7.55E-03	2.97E-03	-1.20E-01
440	726	470	738	501	739	528	723	555	705	585	699	3.47E-01	-1.12E-01	3.05E-01	1.24E-02	2.42E-01	-1.20E-01	1.83E-01	-4.93E-05	1.38E-01	-1.24E-01	6.39E-02	4.85E-03	1.44E-02	-1.17E-01
377	699	406	709	436	716	466	709	491	692	522	689	3.62E-01	-1.18E-01	3.00E-01	1.26E-02	2.44E-01	-1.22E-01	1.82E-01	5.25E-03	1.26E-01	-1.21E-01	6.14E-02	2.83E-03	1.11E-02	-1.16E-01
144	533	179	540	215	540	246	526	278	508	313	500	3.60E-01	-1.02E-01	2.97E-01	1.12E-02	2.57E-01	-1.15E-01	1.83E-01	-2.19E-03	1.48E-01	-1.25E-01	7.01E-02	-5.91E-03	3.02E-02	-1.05E-01
124	527	163	537	202	539	240	529	272	505	307	486	3.66E-01	-1.02E-01	2.96E-01	1.65E-02	2.59E-01	-1.18E-01	1.82E-01	-5.64E-03	1.38E-01	-1.27E-01	5.91E-02	-5.86E-03	1.16E-02	-1.20E-01
97	522	135	539	178	543	218	529	253	503	290	482	3.62E-01	-9.85E-02	2.94E-01	8.54E-03	2.56E-01	-1.12E-01	1.81E-01	-5.11E-03	1.42E-01	-1.21E-01	6.40E-02	-2.77E-03	1.82E-02	-1.15E-01
133	563	172	581	214	587	247	561	270	525	303	498	3.66E-01	-9.86E-02	3.01E-01	1.70E-02	2.57E-01	-1.17E-01	1.80E-01	-6.25E-03	1.34E-01	-1.22E-01	5.91E-02	-4.23E-03	2.12E-02	-1.19E-01
128	519	156	532	186	539	216	531	244	518	275	512	3.70E-01	-1.09E-01	2.98E-01	9.54E-03	2.56E-01	-1.29E-01	1.82E-01	-2.61E-03	1.36E-01	-1.28E-01	6.14E-02	4.39E-03	2.16E-02	-1.18E-01
134	551	173	565	214	568	254	557	285	532	311	500	3.60E-01	-9.09E-02	2.98E-01	2.02E-02	2.55E-01	-1.11E-01	1.83E-01	-6.17E-03	1.41E-01	-1.19E-01	6.25E-02	-4.08E-03	1.92E-02	-1.16E-01
125	568	163	572	201	577	237	571	262	541	297	528	3.78E-01	-1.09E-01	3.09E-01	1.64E-02	2.48E-01	-1.19E-01	1.82E-01	1.46E-03	1.25E-01	-1.20E-01	5.40E-02	2.88E-03	3.49E-03	-1.19E-01
124	596	162	604	201	599	240	594	273	574	307	555	3.65E-01	-1.04E-01	3.00E-01	1.91E-02	2.37E-01	-1.19E-01	1.84E-01	5.30E-03	1.27E-01	-1.23E-01	6.75E-02	-3.65E-03	9.24E-03	-1.16E-01
128	592	167	603	207	604	243	586	276	563	314	551	3.59E-01	-1.03E-01	3.00E-01	8.55E-03	2.57E-01	-1.22E-01	1.84E-01	-2.66E-03	1.34E-01	-1.25E-01	6.14E-02	5.38E-04	2.80E-02	-1.23E-01

96	561	137	584	183	593	227	576	261	543	300	518	3.57E-01	-9.96E-02	3.04E-01	1.11E-02	2.43E-01	-1.04E-01	1.85E-01	-5.02E-03	1.33E-01	-1.27E-01	5.96E-02	-1.52E-03	9.86E-03	-1.17E-01
95	562	123	574	154	578	184	585	213	572	242	561	3.63E-01	-1.09E-01	3.00E-01	1.55E-02	2.62E-01	-1.19E-01	1.80E-01	7.27E-04	1.36E-01	-1.30E-01	5.75E-02	-2.92E-03	1.58E-02	-1.24E-01
120	557	159	572	202	574	241	559	275	534	315	520	3.54E-01	-1.09E-01	2.98E-01	1.27E-02	2.49E-01	-1.20E-01	1.86E-01	-6.02E-03	1.35E-01	-1.24E-01	6.14E-02	-2.50E-03	1.87E-02	-1.21E-01
105	515	143	530	184	537	225	525	256	498	295	485	3.56E-01	-1.07E-01	3.01E-01	1.10E-02	2.53E-01	-1.19E-01	1.85E-01	-6.24E-04	1.38E-01	-1.25E-01	6.38E-02	2.12E-03	1.62E-02	-1.18E-01
117	518	155	531	195	533	226	511	258	488	296	478	3.59E-01	-9.69E-02	3.00E-01	1.32E-02	2.60E-01	-1.21E-01	1.82E-01	-4.32E-03	1.38E-01	-1.27E-01	6.24E-02	-2.54E-03	2.13E-02	-1.19E-01
137	578	174	589	212	589	247	576	275	549	309	533	3.68E-01	-9.37E-02	2.96E-01	1.32E-02	2.56E-01	-1.20E-01	1.82E-01	-1.16E-02	1.44E-01	-1.27E-01	5.82E-02	-5.13E-03	1.54E-02	-1.26E-01
81	509	118	529	161	538	201	531	237	509	278	496	3.55E-01	-1.08E-01	3.00E-01	6.99E-03	2.49E-01	-1.20E-01	1.87E-01	-8.20E-03	1.36E-01	-1.24E-01	6.05E-02	5.80E-04	1.83E-02	-1.14E-01
112	576	149	596	192	602	226	582	256	552	296	537	3.67E-01	-1.06E-01	2.93E-01	1.46E-02	2.55E-01	-1.19E-01	1.80E-01	-8.41E-03	1.38E-01	-1.28E-01	6.18E-02	-6.96E-03	2.23E-02	-1.22E-01
155	581	187	602	225	608	262	601	291	577	326	562	3.63E-01	-1.01E-01	2.94E-01	9.55E-03	2.58E-01	-1.17E-01	1.81E-01	-8.33E-03	1.44E-01	-1.25E-01	6.02E-02	-2.72E-04	2.87E-02	-1.13E-01
373	983	431	998	492	994	547	970	581	922	632	894	3.56E-01	-9.49E-02	2.98E-01	1.78E-02	2.57E-01	-1.17E-01	1.83E-01	-3.38E-03	1.40E-01	-1.27E-01	5.89E-02	-1.16E-02	2.09E-02	-1.22E-01
353	837	414	858	478	869	538	852	568	798	595	746	3.63E-01	-9.28E-02	2.96E-01	1.77E-02	2.59E-01	-1.13E-01	1.80E-01	-6.24E-03	1.38E-01	-1.23E-01	6.23E-02	-4.08E-03	1.76E-02	-1.18E-01
308	951	362	970	419	979	468	950	512	912	564	887	3.70E-01	-9.97E-02	2.96E-01	1.09E-02	2.60E-01	-1.14E-01	1.84E-01	-4.71E-03	1.42E-01	-1.20E-01	6.41E-02	-8.44E-03	2.15E-02	-1.13E-01
241	814	307	827	376	829	439	812	497	774	557	742	3.64E-01	-8.64E-02	2.97E-01	1.63E-02	2.60E-01	-1.05E-01	1.84E-01	-9.41E-03	1.41E-01	-1.17E-01	6.34E-02	-8.10E-03	2.46E-02	-1.12E-01
304	1063	364	1088	430	1097	491	1089	532	1037	590	1008	3.59E-01	-9.56E-02	3.01E-01	1.50E-02	2.59E-01	-1.16E-01	1.81E-01	-8.57E-03	1.37E-01	-1.22E-01	6.27E-02	-3.08E-03	1.96E-02	-1.19E-01
121	549	158	555	189	550	222	534	239	501	269	479	3.41E-01	-1.12E-01	2.97E-01	1.22E-02	2.54E-01	-1.22E-01	1.89E-01	2.57E-04	1.41E-01	-1.33E-01	7.18E-02	-7.89E-03	2.41E-02	-1.32E-01
135	525	170	534	206	539	233	517	265	503	299	493	3.47E-01	-1.08E-01	2.99E-01	7.00E-03	2.49E-01	-1.22E-01	1.90E-01	-2.92E-03	1.33E-01	-1.23E-01	6.28E-02	-2.60E-03	2.21E-02	-1.17E-01
168	600	206	618	247	629	277	617	289	576	317	547	3.34E-01	-1.17E-01	2.97E-01	6.75E-03	2.35E-01	-1.29E-01	1.90E-01	4.77E-04	1.31E-01	-1.33E-01	6.14E-02	5.07E-03	1.44E-02	-1.22E-01
104	577	141	591	180	593	210	570	229	536	259	516	3.49E-01	-1.12E-01	2.94E-01	9.85E-03	2.59E-01	-1.27E-01	1.90E-01	-7.73E-03	1.34E-01	-1.33E-01	6.13E-02	-1.69E-03	2.36E-02	-1.29E-01
127	580	155	592	181	589	200	564	219	540	249	534	3.45E-01	-1.06E-01	2.96E-01	4.17E-03	2.48E-01	-1.24E-01	1.93E-01	-3.73E-03	1.42E-01	-1.27E-01	6.69E-02	3.47E-04	1.80E-02	-1.18E-01
199	544	240	547	280	541	291	507	300	469	334	447	3.41E-01	-8.64E-02	2.93E-01	1.66E-02	2.49E-01	-1.11E-01	1.91E-01	-4.31E-03	1.41E-01	-1.19E-01	6.77E-02	-9.80E-03	2.41E-02	-1.17E-01
124	530	154	549	188	559	220	551	235	518	258	492	3.54E-01	-9.67E-02	2.97E-01	8.88E-03	2.58E-01	-1.17E-01	1.91E-01	-5.17E-03	1.38E-01	-1.29E-01	7.15E-02	-2.05E-02	1.94E-02	-1.24E-01
129	538	166	553	205	563	235	545	253	509	287	494	3.53E-01	-9.68E-02	2.90E-01	5.19E-03	2.49E-01	-1.24E-01	1.84E-01	-9.47E-03	1.37E-01	-1.32E-01	5.56E-02	-1.29E-02	2.87E-02	-1.23E-01
192	523	230	529	267	521	293	493	319	464	352	446	3.42E-01	-1.08E-01	3.00E-01	7.84E-03	2.44E-01	-1.24E-01	1.93E-01	6.61E-03	1.26E-01	-1.27E-01	7.32E-02	-1.66E-03	3.46E-03	-1.24E-01
126	569	161	581	193	564	219	538	245	511	280	501	3.44E-01	-1.09E-01	2.98E-01	5.42E-03	2.48E-01	-1.22E-01	1.88E-01	4.23E-03	1.40E-01	-1.23E-01	6.81E-02	-1.44E-03	2.34E-02	-1.14E-01
133	562	154	565	176	561	196	549	218	542	240	538	3.68E-01	-1.12E-01	3.03E-01	1.20E-02	2.61E-01	-1.28E-01	1.85E-01	9.91E-04	1.40E-01	-1.26E-01	5.68E-02	-4.61E-03	1.18E-02	-1.22E-01
117	575	143	580	169	577	190	562	208	543	232	531	3.43E-01	-9.33E-02	2.94E-01	5.03E-03	2.43E-01	-1.14E-01	1.99E-01	-4.49E-03	1.34E-01	-1.18E-01	7.61E-02	-1.08E-02	1.98E-02	-1.14E-01
125	415	147	419	170	418	183	405	184	383	199	369	3.63E-01	-1.07E-01	3.00E-01	2.13E-02	2.51E-01	-1.17E-01	1.90E-01	3.30E-03	1.36E-01	-1.24E-01	6.27E-02	-4.00E-03	1.02E-02	-1.25E-01
187	447	211	446	234	451	252	441	260	419	279	407	3.54E-01	-1.10E-01	3.01E-01	1.50E-02	2.42E-01	-1.17E-01	1.87E-01	4.63E-03	1.31E-01	-1.19E-01	6.45E-02	-1.59E-03	1.83E-02	-1.14E-01
70	504	94	504	116	512	125	492	135	470	157	461	3.56E-01	-1.07E-01	3.23E-01	1.50E-02	2.38E-01	-1.12E-01	1.87E-01	1.15E-02	1.20E-01	-1.14E-01	6.77E-02	4.68E-04	4.68E-03	-1.06E-01
148	556	181	559	214	559	242	545	264	520	292	503	3.50E-01	-1.08E-01	2.93E-01	9.29E-03	2.52E-01	-1.21E-01	1.87E-01	-4.51E-03	1.42E-01	-1.32E-01	7.22E-02	-7.88E-04	2.28E-02	-1.19E-01
223	579	251	586	280	592	306	586	322	561	346	545	3.46E-01	-1.14E-01	3.07E-01	6.04E-03	2.43E-01	-1.24E-01	1.92E-01	-2.21E-03	1.31E-01	-1.25E-01	6.47E-02	3.27E-03	2.14E-02	-1.19E-01
179	603	206	605	232	597	255	582	278	567	303	558	3.44E-01	-1.05E-01	2.94E-01	9.47E-03	2.54E-01	-1.22E-01	1.88E-01	4.66E-03	1.37E-01	-1.29E-01	8.10E-02	-2.58E-03	1.80E-02	-1.20E-01
209	571	241	577	272	574	295	552	321	532	351	520	3.40E-01	-1.10E-01	2.94E-01	5.90E-03	2.44E-01	-1.28E-01	1.89E-01	-4.98E-04	1.40E-01	-1.28E-01	6.59E-02	8.52E-05	2.53E-02	-1.25E-01
264	578	291	587	319	596	343	587	363	566	388	552	3.29E-01	-1.26E-01	2.96E-01	4.38E-03	2.37E-01	-1.34E-01	1.93E-01	-2.67E-03	1.34E-01	-1.33E-01	7.11E-02	3.74E-03	2.14E-02	-1.21E-01
213	464	241	474	271	478	294	464	309	439	333	422	3.49E-01	-1.02E-01	2.92E-01	3.50E-03	2.51E-01	-1.14E-01	1.93E-01	-8.21E-03	1.50E-01	-1.18E-01	6.68E-02	-8.75E-03	3.87E-02	-1.16E-01
219	579	250	590	283	596	312	588	332	562	361	549	3.37E-01	-1.14E-01	2.95E-01	5.99E-03	2.41E-01	-1.31E-01	1.87E-01	1.23E-03	1.36E-01	-1.28E-01	6.32E-02	1.73E-03	2.40E-02	-1.22E-01
229	595	253	603	277	599	297	582	315	564	338	555	3.51E-01	-1.04E-01	2.98E-01	9.28E-03	2.54E-01	-1.31E-01	1.91E-01	-1.04E-02	1.35E-01	-1.44E-01	7.04E-02	-8.31E-03	1.47E-02	-1.36E-01
199	511	226	518	253	519	264	500	287	484	313	478	3.38E-01	-1.26E-01	3.00E-01	4.43E-03	2.45E-01	-1.35E-01	1.89E-01	-2.44E-03	1.38E-01	-1.35E-01	6.59E-02	1.00E-03	1.75E-02	-1.27E-01
204	590	238	599	272	608	305	602	331	580	363	568	3.40E-01	-1.04E-01	2.90E-01	-6.78E-05	2.38E-01	-1.22E-01	1.85E-01	-2.29E-03	1.35E-01	-1.27E-01	6.96E-02	4.70E-03	2.28E-02	-1.17E-01
175	559	206	574	239	580	270	569	293	544	317	519	3.26E-01	-1.23E-01	2.95E-01	7.41E-03	2.33E-01	-1.28E-01	1.91E-01	3.18E-03	1.24E-01	-1.25E-01	7.16E-02	3.36E-03	1.40E-02	-1.24E-01
163	535	190	551	221	559	251	547	276	527	303	509	3.33E-01	-1.17E-01	2.98E-01	1.09E-03	2.39E-01	-1.14E-01	1.91E-01	-1.62E-03	1.31E-01	-1.22E-01	7.25E-02	4.38E-05	2.57E-02	-1.16E-01
218	651	246	660	274	655	299	640	322	622	348	610	3.41E-01	-1.15E-01	2.96E-01	7.98E-03	2.40E-01	-1.26E-01	1.90E-01	-2.14E-03	1.41E-01	-1.30E-01	7.21E-02	1.12E-03	2.95E-02	-1.22E-01
114	446	143	464	176	473	205	461	223	432	254	419	3.41E-01	-1.07E-01	2.99E-01	8.93E-03	2.50E-01	-1.21E-01	1.92E-01	-2.77E-03	1.48E-01	-1.31E-01	6.75E-02	-3.25E-03	2.92E-02	-1.24E-01
240	507	265	514	292	514	315	503	332	482	350	462	3.61E-01	-1.02E-01	3.05E-01	1.34E-02	2.54E-01	-1.27E-01	1.86E-01	-2.33E-03	1.37E-01	-1.39E-01	6.19E-02	-3.20E-04	1.32E-02	-1.36E-01
188	548	215	557	244	562	271	560	287	537	307	516	3.51E-01	-1.13E-01	3.02E-01	1.49E-03	2.47E-01	-1.19E-01	1.89E-01	-2.26E-03	1.30E-01	-1.25E-01	6.30E-02	-1.24E-03	1.44E-02	-1.18E-01
147	573	179	582	213	586	240	568	259	540	280	516	3.48E-01	-1.12E-01	2.98E-01	1.16E-02	2.50E-01	-1.29E-01	1.90E-01	-3.01E-05	1.33E-01	-1.30E-01	7.00E-02	5.62E-04	1.75E-02	-1.20E-01
184	596	218	606	253	611	286	597	307	572	332	547	3.36E-01	-1.11E-01	2.93E-01	3.02E-03	2.43E-01	-1.30E-01	1.84E-01	-3.23E-03	1.37E-01	-1.27E-01	7.45E-02	2.69E-03	2.33E-02	-1.20E-01
140	558	170	558	193	541	215	519	240	504	270	498	3.50E-01	-1.07E-01	2.97E-01	6.33E-03	2.40E-01	-1.21E-01	1.93E-01	2.60E-03	1.33E-01	-1.23E-01	7.46E-02	-3.49E-03	1.74E-02	-1.11E-01
178	601	217	608	248	588	286	575	303	543	337	527	3.35E-01	-1.05E-01	2.92E-01	1.92E-03	2.42E-01	-1.20E-01	1.91E-01	4.15E-03	1.40E-01	-1.19E-01	7.11E-02	3.00E-03	2.84E-02	-1.17E-01
198	544	228	551	259	555	288	547	303	521	332	510	3.39E-01	-1.06E-01	2.99E-01	3.77E-03	2.45E-01	-1.20E-01	1.89E-01	-3.13E-04	1.32E-01	-1.23E-01	6.42E-02	7.35E-05	1.66E-02	-1.16E-01
198	509	221	524	247	531	271	519	293	501	319	492	3.39E-01	-1.21E-01	3.03E-01	6.83E-03	2.45E-01	-1.24E-01	1.87E-01	9.18E-03	1.30E-01	-1.25E-01	6.38E-02	3.47E-04	1.70E-02	-1.24E-01
190	520	217	532	248	535	275	525	298	504	324	490	3.47E-01	-1.08E-01	2.99E-01	2.84E-03	2.43E-01	-1.23E-01	1.91E-01	-2.45E-03	1.34E-01	-1.24E-01	7.27E-02	6.96E-03	1.55E-02	-1.11E-01
250	509	273	514	297	513	308	494	321	474	343	464	3.57E-01	-1.17E-01	3.06E-01	1.64E-02	2.54E-01	-1.27E-01	1.90E-01	1.23E-03	1.41E-01	-1.37E-01	6.41E-02	-8.87E-04	1.90E-02	-1.41E-01
220	594	246	595	272	588	294	572	316	556	341	546	3.51E-01	-1.13E-01	3.01E-01	1.41E-02	2.54E-01	-1.24E-01	2.00E-01	-2.05E-03	1.43E-01	-1.29E-01	6.70E-02	-4.74E-03	2.04E-02	-1.15E-01
198	563	227	569	256	572	277	552	293	527	316	509	3.48E-01	-9.91E-02	3.01E-01	5.30E-03	2.50E-01	-1.15E-01	1.92E-01	-4.91E-03	1.33E-01	-1.23E-01	6.84E-02	-2.92E-03	1.96E-02	-1.21E-01
245	531	269	537	291	538	306	518	326	504	350	498	3.59E-01	-1.01E-01	3.00E-01	7.07E-03	2.54E-01	-1.22E-01	1.91E-01	-7.91E-03	1.33E-01	-1.30E-01	6.99E-02	-5.44E-03	9.68E-04	-1.19E-01
237	534	267	541	298	541	327	533	347	510	365	484	3.50E-01	-1.17E-01	2.90E-01	1.38E-02	2.50E-01	-1.30E-01	1.81E-01	9.58E-04	1.38E-01	-1.35E-01	6.98E-02	9.01E-04	2.02E-02	-1.28E-01
292	559	321	565	349	557	374	541	392	517	421	511	3.48E-01	-1.18E-01	2.97E-01	1.32E-02	2.50E-01	-1.24E-01	1.87E-01	1.04E-03	1.40E-01	-1.30E-01	6.75E-02	-3.94E-03	1.88E-02	-1.19E-01
321	547	348	554	377	551	397	532	418	511	444	498	3.35E-01	-1.30E-01	2.88E-01	4.25E-03	2.40E-01	-1.29E-01	1.90E-01	2.23E-03	1.39E-01	-1.32E-01	7.06E-02	4.91E-04	2.85E-02	-1.30E-01
206	494	229	513	256	526	284	520	301	495	316	470	3.62E-01	-1.10E-01	2.97E-01	1.32E-02	2.52E-01	-1.25E-01	1.85E-01	8.13E-04	1.15E-01	-1.23E-01	5.85E-02	5.71E-03	-7.99E-03	-1.13E-01
238	502	269	502	301	502	331	498	342	469	361	445	3.63E-01	-1.02E-01	2.95E-01	1.14E-02	2.54E-01	-1.04E-01	1.78E-01	2.46E-03	1.37E-01	-1.14E-01	6.19E-02	-4.70E-03	2.21E-02	-1.24E-01
245	508	282	519	319	527	351	511	345	474	362	442	3.56E-01	-1.05E-01	2.97E-01	2.07E-02	2.48E-01	-1.22E-01	1.88E-01	6.47E-03	1.29E-01	-1.20E-01	7.15E-02	-6.64E-03	1.44E-02	-1.13E-01
172	573	198	584	226	588	251	576	267	554	290	541	3.69E-01	-1.13E-01	3.01E-01	1.11E-02	2.41E-01	-1.15E-01	1.90E-01	7.07E-03	1.23E-01	-1.13E-01	6.21E-02	3.78E-03	1.97E-02	-1.24E-01
228	533	257	544	289	552	316	539	318	507	336	481	3.52E-01	-9.55E-02	3.04E-01	1.16E-02	2.53E-01	-1.15E-01	1.85E-01	-7.03E-04	1.37E-01	-1.22E-01	6.41E-02	-4.13E-04	2.08E-02	-1.14E-01
182	548	222	554	261	554	290	531	312	500	347	487	3.65E-01	-1.02E-01	3.00E-01	1.67E-02	2.45E-01	-1.20E-01	1.88E-01	1.05E-03	1.33E-01	-1.28E-01	6.18E-02	2.97E-03	1.41E-02	-1.22E-01
152	559	187	574	223	583	257	573	266	537	295	516	3.59E-01	-1.03E-01	3.04E-01	1.12E-02	2.52E-01	-1.12E-01	1.86E-01	3.41E-03	1.40E-01	-1.18E-01	5.86E-02	-1.19E-03	2.12E-02	-1.12E-01
163	548	205	559	248	559	290	544	299	515	303	476	3.73E-01	-9.74E-02	2.96E-01	1.49E-02	2.64E-01	-1.14E-01	1.81E-01	3.87E-03	1.28E-01	-1.19E-01	5.78E-02	-2.78E-03	8.05E-03	-1.15E-01
148	496	183	508	218	513	249	501	267	469	297	449	3.52E-01	-1.01E-01	2.95E-01	7.28E-03	2.45E-01	-1.20E-01	1.86E-01	5.40E-05	1.31E-01	-1.24E-01	7.00E-02	1.90E-03	1.34E-02	-1.18E-01
174	478	209	492	245	501	281	495	297	464	321	440	3.55E-01	-1.01E-01	3.01E-01	3.79E-03	2.46E-01	-1.16E-01	1.88E-01	6.88E-03	1.31E-01	-1.22E-01	6.84E-02	9.72E-04	8.97E-03	-1.12E-01
165	480	201	493	239	497	270	475	286	440	310	413	3.58E-01	-1.03E-01	3.12E-01	1.25E-02	2.49E-01	-1.13E-01	1.89E-01	6.17E-03	1.30E-01	-1.13E-01	6.80E-02	4.09E-04	-1.22E-03	-1.07E-01
101	495	127	500	153	501	174	489	177	463	197	445	3.53E-01	-1.08E-01	2.97E-01	1.61E-02	2.45E-01	-1.12E-01	1.83E-01	4.21E-03	1.35E-01	-1.31E-01	5.89E-02	-1.12E-03	1.93E-02	-1.18E-01
128	473	153	485	180	485	200	467	205	440	226	424	3.49E-01	-1.08E-01	2.94E-01	1.20E-02	2.47E-01	-1.19E-01	1.79E-01	3.03E-03	1.34E-01	-1.29E-01	6.03E-02	-2.90E-03	2.95E-02	-1.21E-01
263	457	295	471	329	470	353	449	361	415	384	391	3.53E-01	-1.08E-01	2.95E-01	1.48E-02	2.50E-01	-1.23E-01	1.76E-01	5.53E-04	1.39E-01	-1.25E-01	6.04E-02	-1.03E-03	2.75E-02	-1.19E-01
191	573	229	583	268	586	298	566	285	534	282	501	3.56E-01	-1.10E-01	3.00E-01	1.75E-02	2.40E-01	-1.23E-01	1.84E-01	8.70E-03	1.30E-01	-1.25E-01	6.35E-02	2.22E-03	9.80E-03	-1.18E-01
646	1195	728	1209	811	1206	889	1183	872	1119	854	1053	3.63E-01	-9.59E-02	2.93E-01	1.35E-02	2.62E-01	-1.17E-01	1.79E-01	5.27E-04	1.48E-01	-1.26E-01	6.60E-02	-7.81E-03	2.17E-02	-1.18E-01
630	1231	703	1249	774	1229	834	1182	817	1114	858	1055	3.61E-01	-9.59E-02	2.95E-01	1.45E-02	2.57E-01	-1.20E-01	1.85E-01	3.43E-03	1.41E-01	-1.26E-01	6.71E-02	-6.47E-03	1.39E-02	-1.25E-01
585	1297	659	1292	733	1287	783	1236	767	1169	763	1100	3.57E-01	-9.49E-02	2.90E-01	1.86E-02	2.60E-01	-1.17E-01	1.84E-01	3.37E-03	1.40E-01	-1.25E-01	6.77E-02	-6.73E-03	1.50E-02	-1.24E-01
147	533	189	550	235	548	278	535	286	492	311	460	3.50E-01	-9.95E-02	2.96E-01	1.85E-02	2.53E-01	-1.18E-01	1.81E-01	-5.28E-03	1.38E-01	-1.26E-01	6.52E-02	-3.51E-03	1.89E-02	-1.21E-01
144	558	191	575	237	590	277	565	288	518	311	478	3.45E-01	-1.05E-01	2.97E-01	1.77E-02	2.45E-01	-1.27E-01	1.84E-01	6.61E-03	1.27E-01	-1.29E-01	6.85E-02	4.31E-03	7.06E-03	-1.22E-01
116	499	161	510	205	515	246	498	255	455	256	410	3.64E-01	-9.26E-02	2.97E-01	1.86E-02	2.61E-01	-1.10E-01	1.82E-01	-1.29E-04	1.41E-01	-1.20E-01	5.69E-02	-3.68E-03	1.95E-02	-1.13E-01
160	555	198	568	237	572	273	561	277	521	301	495	3.49E-01	-1.07E-01	2.93E-01	1.83E-02	2.50E-01	-1.19E-01	1.81E-01	9.58E-03	1.29E-01	-1.31E-01	6.45E-02	-3.38E-03	1.82E-02	-1.24E-01
229	493	265	508	302	513	337	499	357	469	373	435	3.54E-01	-1.05E-01	3.00E-01	8.47E-03	2.52E-01	-1.18E-01	1.83E-01	3.46E-03	1.35E-01	-1.20E-01	6.31E-02	3.09E-03	1.72E-02	-1.16E-01
158	398	197	421	240	434	280	430	294	388	302	344	3.52E-01	-1.04E-01	2.97E-01	1.55E-02	2.46E-01	-1.23E-01	1.81E-01	3.52E-03	1.29E-01	-1.25E-01	6.30E-02	-6.91E-04	1.31E-02	-1.19E-01

174	429	213	442	255	444	286	420	300	381	328	352	3.48E-01	-1.03E-01	2.93E-01	9.86E-03	2.51E-01	-1.22E-01	1.84E-01	3.03E-03	1.40E-01	-1.23E-01	6.44E-02	-9.34E-05	3.07E-02	-1.16E-01
242	540	278	562	319	573	347	543	367	505	394	473	3.58E-01	-1.19E-01	2.94E-01	1.18E-02	2.45E-01	-1.28E-01	1.76E-01	7.12E-03	1.24E-01	-1.25E-01	5.91E-02	5.99E-03	1.27E-02	-1.16E-01
167	470	207	484	248	494	287	476	312	455	296	416	3.69E-01	-9.68E-02	2.97E-01	1.38E-02	2.58E-01	-1.18E-01	1.77E-01	6.72E-05	1.40E-01	-1.24E-01	5.99E-02	-3.04E-03	1.80E-02	-1.19E-01
143	510	187	524	232	528	270	508	275	468	283	429	3.60E-01	-8.71E-02	2.86E-01	1.21E-02	2.56E-01	-1.10E-01	1.82E-01	5.26E-04	1.42E-01	-1.16E-01	6.27E-02	-3.19E-03	2.65E-02	-1.14E-01
135	418	180	425	225	425	261	402	245	363	216	329	3.59E-01	-1.00E-01	2.96E-01	2.72E-02	2.47E-01	-1.17E-01	1.80E-01	4.32E-03	1.34E-01	-1.29E-01	6.56E-02	3.49E-04	2.17E-02	-1.28E-01
175	516	222	530	271	534	313	511	310	472	287	429	3.62E-01	-1.00E-01	2.93E-01	1.90E-02	2.51E-01	-1.15E-01	1.82E-01	-1.01E-03	1.30E-01	-1.22E-01	6.28E-02	4.82E-04	1.55E-02	-1.19E-01
202	385	241	404	282	421	323	405	343	371	339	328	3.52E-01	-1.08E-01	2.96E-01	1.17E-02	2.43E-01	-1.18E-01	1.88E-01	-1.27E-03	1.34E-01	-1.20E-01	6.47E-02	-3.92E-03	8.73E-03	-1.12E-01
141	509	179	526	222	534	264	523	271	483	286	443	3.49E-01	-9.78E-02	3.02E-01	9.65E-03	2.51E-01	-1.13E-01	1.88E-01	-6.70E-04	1.35E-01	-1.17E-01	6.03E-02	4.39E-03	2.05E-02	-1.18E-01
137	510	185	514	231	510	270	483	267	441	266	398	3.59E-01	-9.75E-02	2.87E-01	1.76E-02	2.59E-01	-1.15E-01	1.78E-01	4.61E-03	1.41E-01	-1.26E-01	6.74E-02	-7.68E-03	1.67E-02	-1.23E-01
117	427	160	438	204	441	246	432	252	394	255	353	3.53E-01	-1.04E-01	3.00E-01	1.51E-02	2.53E-01	-1.19E-01	1.83E-01	3.39E-03	1.31E-01	-1.26E-01	6.30E-02	-6.96E-03	9.42E-03	-1.24E-01
180	481	222	500	268	500	297	468	289	425	297	381	3.44E-01	-1.01E-01	3.02E-01	1.42E-02	2.48E-01	-1.20E-01	1.88E-01	6.29E-03	1.32E-01	-1.24E-01	6.57E-02	-3.54E-04	1.16E-02	-1.24E-01
165	463	210	481	257	491	293	466	306	420	334	385	3.49E-01	-1.03E-01	2.99E-01	1.53E-02	2.42E-01	-1.24E-01	1.84E-01	2.71E-03	1.33E-01	-1.22E-01	6.28E-02	1.74E-03	1.59E-02	-1.18E-01
160	470	204	477	247	480	288	461	309	424	339	393	3.61E-01	-1.03E-01	2.97E-01	1.70E-02	2.49E-01	-1.23E-01	1.74E-01	4.68E-03	1.33E-01	-1.22E-01	5.92E-02	-2.08E-03	1.36E-02	-1.21E-01
158	418	205	433	253	445	297	430	311	387	317	338	3.64E-01	-1.05E-01	2.86E-01	1.54E-02	2.68E-01	-1.12E-01	1.79E-01	-4.66E-03	1.44E-01	-1.20E-01	6.69E-02	-1.90E-03	2.15E-02	-1.15E-01
156	574	197	602	246	602	294	585	309	540	320	493	3.55E-01	-1.03E-01	2.97E-01	1.15E-02	2.48E-01	-1.15E-01	1.77E-01	4.41E-03	1.34E-01	-1.14E-01	5.80E-02	-2.73E-03	2.43E-02	-1.15E-01
169	639	217	652	267	642	313	622	308	576	317	528	3.71E-01	-8.18E-02	2.92E-01	9.49E-03	2.56E-01	-1.16E-01	1.77E-01	-6.66E-03	1.25E-01	-1.24E-01	5.91E-02	-1.15E-02	1.68E-02	-1.20E-01
563	1120	639	1148	721	1154	800	1146	837	1081	872	1012	3.56E-01	-1.07E-01	2.92E-01	1.02E-02	2.59E-01	-1.24E-01	1.79E-01	-2.24E-03	1.45E-01	-1.32E-01	6.11E-02	1.97E-03	2.91E-02	-1.26E-01
469	1148	550	1181	638	1193	716	1158	755	1082	781	1001	3.56E-01	-9.19E-02	3.01E-01	9.35E-03	2.58E-01	-1.09E-01	1.83E-01	-6.17E-03	1.44E-01	-1.18E-01	6.41E-02	-1.09E-02	2.51E-02	-1.10E-01
556	1093	631	1120	711	1131	790	1118	836	1055	880	993	3.53E-01	-1.06E-01	2.98E-01	1.27E-02	2.59E-01	-1.18E-01	1.76E-01	-3.06E-03	1.39E-01	-1.29E-01	6.12E-02	-6.97E-03	2.95E-02	-1.25E-01
113	467	160	492	212	507	256	489	269	437	296	392	3.65E-01	-1.02E-01	2.94E-01	1.31E-02	2.56E-01	-1.21E-01	1.80E-01	-7.22E-03	1.42E-01	-1.26E-01	5.84E-02	-8.75E-03	1.72E-02	-1.18E-01
117	422	171	438	226	447	282	442	285	401	271	350	3.57E-01	-1.10E-01	2.92E-01	1.71E-02	2.56E-01	-1.26E-01	1.80E-01	5.03E-03	1.35E-01	-1.28E-01	6.59E-02	5.54E-04	1.75E-02	-1.24E-01
188	433	226	444	266	448	299	426	324	400	358	387	3.59E-01	-1.05E-01	2.93E-01	8.97E-03	2.54E-01	-1.13E-01	1.84E-01	-3.66E-03	1.39E-01	-1.21E-01	6.75E-02	-1.42E-02	2.72E-02	-1.13E-01
82	533	122	548	164	556	202	537	235	509	273	490	3.57E-01	-1.13E-01	2.92E-01	1.67E-02	2.60E-01	-1.24E-01	1.78E-01	-3.83E-03	1.50E-01	-1.32E-01	5.87E-02	-3.00E-03	1.65E-02	-1.28E-01
149	466	194	481	242	478	288	466	316	428	346	398	3.60E-01	-9.91E-02	2.86E-01	1.50E-02	2.54E-01	-1.18E-01	1.80E-01	-5.15E-03	1.48E-01	-1.26E-01	6.45E-02	-3.54E-03	2.13E-02	-1.24E-01
126	559	168	573	213	572	253	553	278	516	306	484	3.64E-01	-9.47E-02	2.96E-01	1.60E-02	2.55E-01	-1.15E-01	1.82E-01	7.39E-05	1.45E-01	-1.22E-01	6.62E-02	-7.65E-03	2.45E-02	-1.17E-01
138	458	178	471	218	481	257	469	278	433	292	396	3.58E-01	-1.07E-01	2.93E-01	1.44E-02	2.56E-01	-1.24E-01	1.83E-01	2.80E-03	1.37E-01	-1.28E-01	5.96E-02	-2.99E-03	2.04E-02	-1.21E-01
115	469	158	485	205	494	250	492	269	448	287	410	3.62E-01	-1.03E-01	2.92E-01	1.32E-02	2.46E-01	-1.13E-01	1.82E-01	-2.91E-03	1.35E-01	-1.22E-01	6.35E-02	2.38E-03	1.78E-02	-1.17E-01
213	499	254	520	300	525	338	501	363	463	396	436	3.57E-01	-9.71E-02	2.91E-01	1.12E-02	2.56E-01	-1.14E-01	1.80E-01	-6.33E-03	1.37E-01	-1.21E-01	5.75E-02	-8.54E-03	2.21E-02	-1.13E-01
108	465	145	481	183	494	222	490	244	457	274	430	3.50E-01	-1.07E-01	2.96E-01	1.63E-02	2.52E-01	-1.21E-01	1.83E-01	-2.78E-03	1.36E-01	-1.31E-01	6.21E-02	-5.48E-03	1.83E-02	-1.22E-01
108	494	153	508	199	518	244	513	269	474	302	444	3.56E-01	-1.00E-01	2.89E-01	1.07E-02	2.58E-01	-1.20E-01	1.85E-01	-3.89E-03	1.29E-01	-1.24E-01	6.46E-02	-4.59E-03	2.40E-02	-1.18E-01
140	399	183	418	229	426	267	404	293	363	333	340	3.51E-01	-1.07E-01	2.95E-01	1.68E-02	2.48E-01	-1.24E-01	1.79E-01	3.22E-03	1.35E-01	-1.31E-01	6.49E-02	-1.81E-03	2.45E-02	-1.34E-01
112	452	154	477	201	487	248	482	267	440	294	407	3.58E-01	-1.07E-01	2.90E-01	9.76E-03	2.53E-01	-1.20E-01	1.84E-01	-7.59E-03	1.38E-01	-1.29E-01	6.21E-02	-6.87E-03	2.47E-02	-1.28E-01
105	509	144	523	186	523	224	507	252	475	289	464	3.51E-01	-9.87E-02	2.91E-01	1.41E-02	2.48E-01	-1.16E-01	1.86E-01	-3.65E-03	1.40E-01	-1.27E-01	6.33E-02	-4.56E-03	1.74E-02	-1.20E-01
119	527	160	543	206	547	248	532	278	499	316	478	3.54E-01	-1.04E-01	2.91E-01	1.26E-02	2.52E-01	-1.14E-01	1.81E-01	-4.67E-03	1.39E-01	-1.25E-01	6.87E-02	-1.30E-02	2.26E-02	-1.19E-01
149	382	185	399	226	403	263	394	288	365	326	350	3.49E-01	-1.06E-01	2.95E-01	1.67E-02	2.44E-01	-1.16E-01	1.87E-01	-8.12E-04	1.43E-01	-1.28E-01	6.40E-02	-7.94E-03	1.72E-02	-1.21E-01
118	456	160	478	208	482	249	461	276	422	316	407	3.62E-01	-1.12E-01	2.96E-01	1.04E-02	2.59E-01	-1.23E-01	1.83E-01	2.00E-04	1.40E-01	-1.27E-01	6.38E-02	5.58E-04	2.37E-02	-1.14E-01
453	686	478	694	505	695	531	694	549	679	561	660	3.57E-01	-9.67E-02	2.99E-01	1.94E-02	2.53E-01	-1.17E-01	1.87E-01	1.34E-03	1.32E-01	-1.27E-01	6.64E-02	-6.50E-03	2.86E-02	-1.29E-01
474	671	501	678	527	681	551	671	556	646	576	628	3.55E-01	-1.06E-01	2.95E-01	8.36E-03	2.62E-01	-1.18E-01	1.84E-01	-1.73E-03	1.37E-01	-1.18E-01	6.19E-02	1.84E-03	2.71E-02	-1.19E-01
485	701	510	710	536	715	563	706	575	683	585	658	3.72E-01	-9.86E-02	2.96E-01	1.23E-02	2.52E-01	-1.18E-01	1.71E-01	9.90E-04	1.29E-01	-1.28E-01	5.41E-02	-9.16E-03	7.01E-03	-1.16E-01
450	673	474	683	500	688	526	683	542	663	555	639	3.62E-01	-1.02E-01	2.96E-01	5.76E-03	2.57E-01	-1.11E-01	1.90E-01	-4.47E-03	1.32E-01	-1.16E-01	6.74E-02	-5.87E-03	1.85E-02	-1.14E-01
549	680	571	693	597	697	620	687	623	661	630	637	3.65E-01	-1.17E-01	2.93E-01	1.39E-02	2.60E-01	-1.29E-01	1.77E-01	-2.08E-03	1.34E-01	-1.26E-01	5.40E-02	-2.27E-03	2.17E-02	-1.25E-01
469	725	497	736	527	745	554	739	565	710	573	680	3.56E-01	-9.65E-02	2.94E-01	1.27E-02	2.50E-01	-1.19E-01	1.84E-01	-7.04E-05	1.37E-01	-1.31E-01	5.84E-02	-7.45E-03	1.96E-02	-1.30E-01
459	646	485	653	511	658	537	656	550	633	568	614	3.46E-01	-1.09E-01	3.00E-01	1.82E-02	2.47E-01	-1.21E-01	1.87E-01	9.83E-04	1.26E-01	-1.26E-01	6.71E-02	1.32E-03	6.04E-03	-1.20E-01
438	668	463	680	490	684	516	677	530	654	543	632	3.60E-01	-1.01E-01	2.99E-01	1.51E-02	2.55E-01	-1.21E-01	1.86E-01	-9.91E-04	1.34E-01	-1.29E-01	5.95E-02	-2.21E-03	2.00E-02	-1.21E-01
466	699	489	714	516	718	543	710	566	694	579	669	3.47E-01	-9.99E-02	2.92E-01	1.18E-02	2.56E-01	-1.22E-01	1.83E-01	-3.68E-03	1.31E-01	-1.22E-01	6.42E-02	-7.73E-04	2.04E-02	-1.18E-01
588	703	617	715	646	722	676	714	690	692	690	662	3.53E-01	-1.05E-01	2.96E-01	1.95E-02	2.42E-01	-1.14E-01	1.83E-01	1.78E-04	1.31E-01	-1.22E-01	6.52E-02	-4.85E-03	1.75E-02	-1.21E-01
463	662	494	675	525	685	557	684	576	659	581	631	3.63E-01	-9.79E-02	2.93E-01	1.56E-02	2.53E-01	-1.22E-01	1.79E-01	-2.03E-03	1.36E-01	-1.32E-01	5.86E-02	-1.85E-03	2.36E-02	-1.34E-01
507	647	533	660	562	671	590	671	604	644	619	619	3.57E-01	-1.07E-01	2.90E-01	1.10E-02	2.48E-01	-1.27E-01	1.80E-01	1.27E-04	1.28E-01	-1.37E-01	6.96E-02	-9.69E-03	1.08E-02	-1.27E-01
463	657	492	665	523	668	551	657	563	633	586	619	3.46E-01	-1.02E-01	2.99E-01	1.47E-02	2.50E-01	-1.20E-01	1.87E-01	-1.42E-03	1.34E-01	-1.25E-01	6.19E-02	-4.32E-03	2.67E-02	-1.25E-01
447	664	468	676	493	676	517	670	533	651	547	630	3.62E-01	-1.11E-01	2.96E-01	2.63E-02	2.48E-01	-1.24E-01	1.88E-01	1.42E-03	1.38E-01	-1.29E-01	6.03E-02	-4.23E-05	1.60E-02	-1.31E-01
512	703	544	714	576	721	608	712	614	683	607	653	3.56E-01	-1.03E-01	2.94E-01	1.94E-02	2.49E-01	-1.20E-01	1.77E-01	-3.37E-04	1.33E-01	-1.34E-01	6.30E-02	-4.05E-03	5.80E-03	-1.24E-01
554	666	581	674	609	670	635	657	646	630	668	612	3.62E-01	-1.01E-01	2.94E-01	1.70E-02	2.54E-01	-1.18E-01	1.78E-01	-1.98E-03	1.43E-01	-1.28E-01	6.22E-02	-1.89E-03	1.18E-02	-1.25E-01
514	641	542	654	572	658	599	649	610	620	632	601	3.62E-01	-1.02E-01	2.95E-01	1.57E-02	2.46E-01	-1.11E-01	1.81E-01	-6.12E-03	1.40E-01	-1.25E-01	6.30E-02	-2.29E-03	1.65E-02	-1.20E-01
531	721	557	727	583	725	606	713	626	696	650	686	3.53E-01	-1.01E-01	2.96E-01	1.20E-02	2.51E-01	-1.13E-01	1.88E-01	1.35E-03	1.38E-01	-1.20E-01	7.32E-02	3.41E-03	1.64E-02	-1.13E-01
389	710	415	721	441	719	467	708	481	684	502	667	3.48E-01	-1.09E-01	2.96E-01	1.00E-02	2.50E-01	-1.23E-01	1.80E-01	2.89E-04	1.45E-01	-1.25E-01	6.52E-02	4.84E-04	2.20E-02	-1.18E-01
489	620	515	632	544	634	571	628	582	604	597	582	3.53E-01	-1.08E-01	2.93E-01	1.57E-02	2.53E-01	-1.22E-01	1.85E-01	-2.46E-03	1.44E-01	-1.28E-01	6.78E-02	-1.49E-03	2.27E-02	-1.25E-01
519	664	544	675	571	681	597	676	610	652	627	632	3.61E-01	-1.02E-01	3.01E-01	1.49E-02	2.48E-01	-1.21E-01	1.85E-01	4.47E-03	1.26E-01	-1.28E-01	6.20E-02	5.83E-03	1.09E-02	-1.21E-01
449	668	468	682	491	688	513	684	530	669	550	658	3.59E-01	-1.11E-01	2.98E-01	9.09E-03	2.51E-01	-1.23E-01	1.82E-01	-5.33E-03	1.30E-01	-1.27E-01	6.19E-02	-2.01E-03	1.98E-02	-1.21E-01
524	1232	595	1261	673	1267	739	1236	783	1174	844	1135	3.52E-01	-1.10E-01	2.96E-01	1.66E-02	2.48E-01	-1.18E-01	1.87E-01	-4.95E-04	1.35E-01	-1.22E-01	5.90E-02	-1.97E-03	2.15E-02	-1.20E-01
582	1036	648	1065	718	1071	784	1045	840	1007	886	957	3.54E-01	-1.07E-01	2.96E-01	1.31E-02	2.51E-01	-1.17E-01	1.88E-01	-3.07E-03	1.44E-01	-1.17E-01	6.52E-02	2.23E-03	1.88E-02	-1.11E-01
540	1088	616	1118	694	1135	768	1101	812	1049	853	992	3.57E-01	-1.15E-01	2.93E-01	8.63E-03	2.57E-01	-1.24E-01	1.80E-01	-1.90E-03	1.34E-01	-1.24E-01	6.23E-02	-3.05E-03	2.64E-02	-1.17E-01
139	480	184	501	233	505	281	492	315	456	349	424	3.53E-01	-1.06E-01	2.94E-01	1.35E-02	2.46E-01	-1.19E-01	1.87E-01	-2.34E-03	1.34E-01	-1.21E-01	6.21E-02	-4.08E-03	2.07E-02	-1.11E-01
139	582	183	599	231	610	270	588	292	545	319	505	3.51E-01	-9.92E-02	3.04E-01	1.18E-02	2.50E-01	-1.14E-01	1.87E-01	-8.17E-04	1.34E-01	-1.20E-01	5.71E-02	-3.27E-03	7.74E-03	-1.14E-01
103	578	141	600	186	613	232	613	265	583	279	539	3.54E-01	-1.02E-01	3.03E-01	1.44E-02	2.54E-01	-1.14E-01	1.84E-01	-2.81E-03	1.38E-01	-1.19E-01	6.34E-02	-2.69E-03	1.18E-02	-1.13E-01
109	588	158	607	212	616	265	606	295	561	328	519	3.52E-01	-9.30E-02	2.95E-01	7.36E-03	2.57E-01	-1.07E-01	1.83E-01	-6.10E-03	1.37E-01	-1.12E-01	6.69E-02	-2.95E-03	3.23E-02	-1.12E-01
110	545	154	565	202	576	245	563	276	526	312	495	3.55E-01	-1.08E-01	2.96E-01	1.16E-02	2.49E-01	-1.19E-01	1.81E-01	-1.29E-03	1.37E-01	-1.19E-01	5.67E-02	-5.61E-04	2.08E-02	-1.14E-01
124	580	169	599	214	611	260	600	293	565	335	543	3.65E-01	-9.85E-02	2.94E-01	1.17E-02	2.55E-01	-1.15E-01	1.82E-01	-2.11E-03	1.38E-01	-1.19E-01	6.09E-02	3.49E-03	3.38E-02	-1.13E-01
137	613	186	632	236	639	267	608	305	575	335	533	3.52E-01	-1.02E-01	3.04E-01	1.18E-02	2.53E-01	-1.18E-01	1.85E-01	-2.04E-03	1.37E-01	-1.19E-01	5.99E-02	-6.40E-05	2.37E-02	-1.11E-01
80	559	132	589	192	594	246	579	265	524	299	484	3.51E-01	-1.04E-01	2.95E-01	7.52E-03	2.49E-01	-1.19E-01	1.83E-01	-2.20E-03	1.31E-01	-1.22E-01	6.04E-02	1.68E-03	1.73E-02	-1.18E-01
131	678	173	691	216	695	260	690	278	656	294	619	3.62E-01	-9.95E-02	2.94E-01	1.52E-02	2.58E-01	-1.14E-01	1.86E-01	-1.18E-04	1.35E-01	-1.21E-01	6.29E-02	-3.13E-03	1.61E-02	-1.16E-01
115	608	157	625	199	636	236	620	264	589	300	567	3.66E-01	-1.05E-01	2.94E-01	9.79E-03	2.52E-01	-1.15E-01	1.84E-01	-6.94E-03	1.33E-01	-1.21E-01	7.37E-02	-2.61E-03	1.27E-02	-1.12E-01
121	587	170	617	226	628	274	606	296	553	328	511	3.55E-01	-1.01E-01	2.96E-01	1.36E-02	2.55E-01	-1.11E-01	1.83E-01	-2.22E-03	1.30E-01	-1.15E-01	6.65E-02	-2.68E-03	2.30E-02	-1.14E-01
129	622	173	641	219	651	262	644	288	606	326	583	3.70E-01	-1.02E-01	2.93E-01	1.22E-02	2.59E-01	-1.18E-01	1.83E-01	-4.68E-03	1.39E-01	-1.24E-01	6.62E-02	-7.76E-03	1.90E-02	-1.11E-01
85	615	132	638	183	649	223	627	246	582	278	546	3.57E-01	-1.01E-01	3.02E-01	1.77E-02	2.54E-01	-1.17E-01	1.79E-01	-4.77E-04	1.34E-01	-1.26E-01	6.57E-02	3.39E-03	1.69E-02	-1.21E-01
86	469	132	486	181	497	231	488	265	456	300	428	3.57E-01	-1.03E-01	2.94E-01	1.09E-02	2.56E-01	-1.15E-01	1.82E-01	-3.14E-03	1.43E-01	-1.20E-01	6.63E-02	-2.43E-03	2.31E-02	-1.16E-01
85	563	127	582	174	587	214	565	253	541	297	527	3.55E-01	-1.14E-01	2.98E-01	2.54E-03	2.46E-01	-1.16E-01	1.84E-01	-4.52E-03	1.26E-01	-1.18E-01	6.87E-02	-3.93E-03	2.01E-02	-1.12E-01
181	478	218	500	261	512	301	502	320	462	345	429	3.54E-01	-1.01E-01	2.92E-01	1.49E-02	2.52E-01	-1.21E-01	1.80E-01	-4.23E-03	1.42E-01	-1.26E-01	6.20E-02	-2.82E-04	7.68E-03	-1.20E-01
171	477	217	496	266	507	309	491	306	444	321	402	3.75E-01	-9.00E-02	2.95E-01	1.19E-02	2.64E-01	-1.10E-01	1.78E-01	-8.50E-03	1.40E-01	-1.21E-01	5.30E-02	-6.69E-03	1.64E-02	-1.13E-01
95	587	144	609	196	617	246	603	265	560	288	517	3.63E-01	-1.03E-01	2.93E-01	1.22E-02	2.54E-01	-1.14E-01	1.80E-01	-3.76E-03	1.35E-01	-1.21E-01	6.18E-02	-1.11E-02	2.04E-02	-1.21E-01
160	513	200	522	241	528	279	515	305	482	338	461	3.62E-01	-1.00E-01	2.99E-01	1.85E-02	2.53E-01	-1.14E-01	1.87E-01	-2.62E-03	1.41E-01	-1.22E-01	6.52E-02	-4.72E-03	1.49E-02	-1.18E-01
123	649	170	667	220	674	267	660	297	625	332	599	3.50E-01	-1.06E-01	2.95E-01	1.24E-02	2.47E-01	-1.12E-01	1.87E-01	-2.68E-03	1.44E-01	-1.14E-01	6.43E-02	-1.21E-03	1.76E-02	-1.07E-01
95	591	134	609	176	614	216	602	249	576	283	565	3.64E-01	-1.02E-01	2.97E-01	3.08E-03	2.50E-01	-1.17E-01	1.92E-01	-2.69E-03	1.37E-01	-1.19E-01	6.58E-02	-1.44E-03	2.45E-02	-1.12E-01
102	560	143	584	189	600	232	585	267	553	288	511	3.54E-01	-1.10E-01	2.98E-01	1.21E-02	2.51E-01	-1.18E-01	1.88E-01	-3.92E-03	1.40E-01	-1.19E-01	6.03E-02	3.58E-03	2.64E-02	-1.14E-01

120	600	165	614	211	618	249	597	271	558	304	529	3.55E-01	-9.89E-02	2.99E-01	7.07E-03	2.52E-01	-1.11E-01	1.90E-01	-3.85E-03	1.39E-01	-1.13E-01	6.68E-02	-1.97E-03	1.42E-02	-1.04E-01
123	645	157	659	193	663	224	646	249	621	280	601	3.58E-01	-1.06E-01	2.96E-01	1.47E-02	2.48E-01	-1.18E-01	1.89E-01	-4.15E-03	1.38E-01	-1.21E-01	6.80E-02	-1.66E-03	2.51E-02	-1.21E-01
152	481	192	500	234	505	277	496	295	459	307	419	3.67E-01	-9.60E-02	2.97E-01	1.26E-02	2.53E-01	-1.13E-01	1.80E-01	-4.28E-03	1.28E-01	-1.23E-01	6.47E-02	-2.65E-03	2.12E-02	-1.17E-01
146	502	187	518	230	527	266	509	288	473	309	435	3.54E-01	-8.74E-02	2.95E-01	9.44E-03	2.53E-01	-1.17E-01	1.82E-01	-3.36E-03	1.34E-01	-1.27E-01	6.26E-02	-2.64E-03	1.91E-02	-1.24E-01
120	460	159	475	200	482	234	466	255	431	286	404	3.65E-01	-9.71E-02	2.98E-01	1.42E-02	2.57E-01	-1.11E-01	1.86E-01	-3.62E-03	1.33E-01	-1.16E-01	6.49E-02	-8.03E-03	1.37E-02	-1.11E-01
87	574	133	595	183	604	227	583	260	547	287	510	3.59E-01	-9.75E-02	2.94E-01	9.73E-03	2.57E-01	-1.16E-01	1.82E-01	-5.22E-03	1.36E-01	-1.24E-01	6.28E-02	-2.83E-03	2.59E-02	-1.22E-01
86	486	128	514	178	527	220	499	248	454	285	422	3.47E-01	-1.02E-01	2.97E-01	1.39E-02	2.55E-01	-1.14E-01	1.80E-01	3.08E-03	1.36E-01	-1.21E-01	6.03E-02	1.97E-03	1.94E-02	-1.18E-01
114	451	156	469	201	479	240	460	266	422	300	391	3.53E-01	-1.07E-01	2.94E-01	1.64E-02	2.51E-01	-1.18E-01	1.82E-01	-1.66E-03	1.40E-01	-1.25E-01	6.47E-02	-4.73E-03	1.95E-02	-1.18E-01
91	409	134	428	176	438	216	420	239	380	274	355	3.64E-01	-1.01E-01	2.97E-01	1.34E-02	2.57E-01	-1.17E-01	1.80E-01	-1.24E-03	1.39E-01	-1.29E-01	6.23E-02	-4.54E-03	1.99E-02	-1.17E-01
155	382	195	395	237	402	275	389	292	353	316	320	3.59E-01	-1.03E-01	2.91E-01	1.10E-02	2.45E-01	-1.12E-01	1.84E-01	-4.61E-03	1.38E-01	-1.19E-01	6.26E-02	-4.62E-03	2.06E-02	-1.18E-01
100	446	135	472	176	483	214	468	237	433	268	404	3.67E-01	-9.26E-02	2.97E-01	6.85E-03	2.66E-01	-1.15E-01	1.88E-01	-1.12E-02	1.45E-01	-1.22E-01	6.62E-02	-1.07E-02	2.10E-02	-1.13E-01
96	402	134	426	177	441	220	437	251	406	286	380	3.55E-01	-1.05E-01	2.92E-01	4.75E-03	2.54E-01	-1.18E-01	1.82E-01	-2.91E-03	1.41E-01	-1.24E-01	6.69E-02	-5.10E-03	3.03E-02	-1.19E-01
117	353	154	370	193	379	223	359	221	318	225	279	3.54E-01	-1.02E-01	2.90E-01	1.54E-02	2.56E-01	-1.18E-01	1.86E-01	-3.76E-03	1.37E-01	-1.22E-01	6.12E-02	-1.47E-03	2.04E-02	-1.21E-01
113	370	147	385	185	384	221	373	237	339	262	317	3.46E-01	-9.88E-02	2.97E-01	9.25E-03	2.53E-01	-1.09E-01	1.85E-01	1.15E-03	1.42E-01	-1.15E-01	7.01E-02	-2.75E-03	1.81E-02	-1.15E-01
120	440	160	449	200	450	239	441	253	404	275	373	3.51E-01	-9.65E-02	2.95E-01	1.13E-02	2.54E-01	-1.14E-01	1.83E-01	-5.11E-03	1.40E-01	-1.19E-01	6.42E-02	2.38E-03	2.42E-02	-1.15E-01
213	434	254	448	296	444	337	435	340	395	341	354	3.64E-01	-1.06E-01	2.95E-01	2.06E-02	2.55E-01	-1.28E-01	1.81E-01	5.24E-03	1.33E-01	-1.29E-01	5.78E-02	4.37E-03	8.63E-03	-1.23E-01
138	562	170	577	205	582	238	573	259	545	279	517	3.53E-01	-1.04E-01	2.98E-01	1.89E-02	2.54E-01	-1.15E-01	1.86E-01	2.52E-05	1.28E-01	-1.24E-01	6.33E-02	-5.12E-04	7.51E-03	-1.15E-01
177	393	207	402	240	401	267	385	288	361	316	345	3.51E-01	-1.09E-01	2.91E-01	7.81E-03	2.41E-01	-1.18E-01	1.83E-01	-4.01E-03	1.38E-01	-1.24E-01	7.17E-02	-5.69E-03	2.27E-02	-1.28E-01
161	630	195	644	233	647	263	627	283	595	296	562	3.51E-01	-1.01E-01	2.98E-01	7.75E-03	2.51E-01	-1.11E-01	1.80E-01	-7.60E-03	1.29E-01	-1.20E-01	5.42E-02	-8.59E-04	1.05E-02	-1.18E-01
112	473	142	488	174	494	205	485	229	461	255	440	3.67E-01	-1.00E-01	2.96E-01	8.31E-03	2.57E-01	-1.21E-01	1.83E-01	-2.32E-03	1.51E-01	-1.21E-01	6.30E-02	-2.84E-03	1.79E-02	-1.18E-01
82	379	112	401	149	403	186	398	212	373	242	351	3.57E-01	-1.01E-01	2.88E-01	1.57E-02	2.53E-01	-1.19E-01	1.86E-01	-2.04E-03	1.36E-01	-1.21E-01	6.80E-02	-9.11E-03	1.47E-02	-1.19E-01
143	564	179	580	218	582	255	575	278	547	298	515	3.52E-01	-9.97E-02	3.07E-01	8.77E-03	2.47E-01	-1.18E-01	1.84E-01	-1.09E-03	1.28E-01	-1.24E-01	6.06E-02	-3.07E-03	1.71E-02	-1.19E-01
102	508	144	531	189	545	236	545	251	500	283	467	3.45E-01	-1.08E-01	2.96E-01	3.69E-03	2.42E-01	-1.19E-01	1.82E-01	-3.45E-03	1.32E-01	-1.24E-01	6.11E-02	6.34E-04	2.60E-02	-1.22E-01
105	530	149	554	197	565	242	555	267	515	280	468	3.53E-01	-1.02E-01	2.84E-01	1.40E-02	2.56E-01	-1.19E-01	1.74E-01	-7.34E-03	1.43E-01	-1.27E-01	5.83E-02	-3.44E-03	1.37E-02	-1.17E-01
122	414	156	434	196	438	227	417	254	386	287	378	3.59E-01	-9.80E-02	2.93E-01	9.20E-03	2.55E-01	-1.18E-01	1.85E-01	-4.82E-03	1.40E-01	-1.22E-01	6.10E-02	-1.76E-03	3.07E-02	-1.15E-01
88	335	123	344	156	358	188	352	209	323	237	306	3.50E-01	-1.07E-01	2.96E-01	1.63E-02	2.53E-01	-1.20E-01	1.88E-01	1.40E-03	1.35E-01	-1.26E-01	6.65E-02	-5.21E-03	1.08E-02	-1.20E-01
140	504	184	507	226	518	262	493	284	456	313	425	3.66E-01	-9.68E-02	2.95E-01	8.18E-03	2.56E-01	-1.11E-01	1.86E-01	-2.84E-03	1.47E-01	-1.17E-01	7.34E-02	-3.90E-03	2.21E-02	-1.12E-01
178	483	213	493	251	497	287	504	313	480	331	448	3.44E-01	-1.13E-01	2.95E-01	7.88E-03	2.45E-01	-1.21E-01	1.89E-01	-2.52E-03	1.30E-01	-1.25E-01	6.82E-02	1.82E-03	2.02E-02	-1.16E-01
90	562	140	579	193	587	225	558	251	514	300	497	3.57E-01	-1.05E-01	2.90E-01	1.33E-02	2.50E-01	-1.18E-01	1.83E-01	-4.96E-03	1.38E-01	-1.23E-01	6.57E-02	-7.51E-03	1.79E-02	-1.17E-01
147	497	189	503	231	506	262	482	278	443	305	415	3.67E-01	-9.23E-02	2.93E-01	9.84E-03	2.55E-01	-1.09E-01	1.86E-01	-4.81E-03	1.35E-01	-1.19E-01	6.63E-02	-6.36E-03	1.74E-02	-1.13E-01
109	431	157	440	203	455	244	433	258	393	215	369	3.65E-01	-1.01E-01	2.96E-01	1.37E-02	2.56E-01	-1.19E-01	1.78E-01	-1.80E-03	1.38E-01	-1.28E-01	6.45E-02	-6.00E-03	1.43E-02	-1.26E-01
127	574	174	592	222	593	263	568	285	524	307	483	3.63E-01	-1.08E-01	2.96E-01	1.59E-02	2.53E-01	-1.22E-01	1.82E-01	-3.74E-03	1.33E-01	-1.32E-01	5.95E-02	-6.79E-03	1.63E-02	-1.29E-01
129	579	172	588	215	591	252	573	274	536	298	501	3.55E-01	-1.01E-01	2.96E-01	1.58E-02	2.59E-01	-1.19E-01	1.83E-01	-6.89E-03	1.41E-01	-1.24E-01	5.94E-02	-4.48E-03	1.85E-02	-1.15E-01
133	621	177	633	224	627	268	612	288	574	289	531	3.60E-01	-9.01E-02	2.88E-01	6.63E-03	2.62E-01	-1.08E-01	1.84E-01	-4.35E-03	1.49E-01	-1.13E-01	6.25E-02	1.59E-04	2.50E-02	-1.13E-01
157	639	205	649	255	647	297	629	307	581	335	546	3.60E-01	-1.13E-01	2.89E-01	1.15E-02	2.54E-01	-1.28E-01	1.78E-01	3.14E-03	1.41E-01	-1.33E-01	6.07E-02	2.69E-03	1.47E-02	-1.24E-01
134	526	179	545	227	553	266	529	273	480	294	438	3.64E-01	-9.80E-02	2.97E-01	1.87E-02	2.58E-01	-1.22E-01	1.79E-01	-5.37E-03	1.29E-01	-1.28E-01	5.98E-02	-3.94E-04	8.27E-03	-1.16E-01
111	589	153	596	199	596	243	587	258	547	276	508	3.60E-01	-1.04E-01	2.95E-01	1.53E-02	2.60E-01	-1.17E-01	1.83E-01	-6.70E-04	1.39E-01	-1.24E-01	6.03E-02	-8.96E-04	1.61E-02	-1.22E-01
111	647	154	662	200	672	226	641	258	607	300	591	3.53E-01	-1.02E-01	2.99E-01	6.51E-03	2.51E-01	-1.21E-01	1.84E-01	-8.18E-04	1.35E-01	-1.18E-01	6.28E-02	-1.06E-03	7.53E-03	-1.13E-01
134	600	176	617	219	615	254	587	279	554	308	521	3.54E-01	-1.05E-01	3.00E-01	6.11E-03	2.50E-01	-1.14E-01	1.85E-01	-3.71E-03	1.45E-01	-1.12E-01	6.98E-02	-5.36E-03	2.17E-02	-1.12E-01
142	571	182	579	223	578	259	561	284	529	314	501	3.50E-01	-1.09E-01	2.94E-01	5.85E-03	2.58E-01	-1.20E-01	1.84E-01	2.94E-03	1.33E-01	-1.18E-01	6.60E-02	4.67E-03	2.93E-02	-1.19E-01
477	725	511	734	546	733	571	711	589	681	617	668	3.59E-01	-9.93E-02	2.94E-01	2.00E-02	2.57E-01	-1.18E-01	1.89E-01	-4.55E-03	1.41E-01	-1.27E-01	6.24E-02	-1.17E-02	2.38E-02	-1.23E-01
392	719	423	728	456	727	486	717	503	691	528	675	3.58E-01	-1.06E-01	2.99E-01	1.74E-02	2.53E-01	-1.24E-01	1.86E-01	4.62E-03	1.31E-01	-1.27E-01	5.84E-02	3.21E-04	5.43E-03	-1.22E-01
490	681	527	689	564	696	593	685	602	650	620	619	3.47E-01	-1.07E-01	3.05E-01	1.25E-02	2.56E-01	-1.17E-01	1.88E-01	4.07E-03	1.43E-01	-1.19E-01	6.49E-02	3.30E-03	5.68E-03	-1.18E-01
424	704	458	718	494	721	520	703	529	667	553	644	3.50E-01	-1.06E-01	2.99E-01	1.14E-02	2.46E-01	-1.19E-01	1.91E-01	4.62E-03	1.28E-01	-1.21E-01	7.06E-02	-4.35E-03	6.34E-03	-1.19E-01
468	674	502	685	537	685	572	685	586	654	602	623	3.59E-01	-1.12E-01	2.84E-01	6.73E-03	2.52E-01	-1.30E-01	1.82E-01	-8.69E-04	1.44E-01	-1.18E-01	5.73E-02	-2.70E-03	3.31E-02	-1.24E-01
524	651	558	665	593	675	625	665	634	632	639	597	3.49E-01	-1.01E-01	2.98E-01	4.85E-03	2.51E-01	-1.18E-01	1.81E-01	-4.74E-03	1.37E-01	-1.18E-01	6.32E-02	-3.98E-03	2.08E-02	-1.15E-01
436	647	474	656	512	657	545	638	564	605	582	575	3.53E-01	-1.01E-01	3.03E-01	1.31E-02	2.50E-01	-1.17E-01	1.85E-01	4.56E-03	1.35E-01	-1.21E-01	6.88E-02	-7.04E-03	8.97E-03	-1.17E-01
469	715	508	730	549	729	588	717	601	677	636	659	3.59E-01	-9.91E-02	2.88E-01	1.05E-02	2.56E-01	-1.21E-01	1.76E-01	-2.39E-03	1.38E-01	-1.29E-01	6.15E-02	-1.00E-04	2.18E-02	-1.24E-01
407	606	443	623	483	630	524	628	550	601	561	564	3.49E-01	-1.14E-01	2.98E-01	1.17E-02	2.47E-01	-1.23E-01	1.85E-01	-2.37E-03	1.32E-01	-1.26E-01	6.48E-02	3.19E-03	2.57E-02	-1.17E-01
399	704	434	715	471	720	505	706	516	671	536	646	3.45E-01	-1.06E-01	3.04E-01	1.64E-02	2.44E-01	-1.19E-01	1.91E-01	-7.47E-04	1.31E-01	-1.23E-01	6.55E-02	3.75E-03	1.45E-02	-1.15E-01
455	697	488	712	524	714	559	705	570	670	584	641	3.62E-01	-1.03E-01	2.93E-01	9.46E-03	2.59E-01	-1.29E-01	1.78E-01	-2.25E-03	1.34E-01	-1.22E-01	5.18E-02	-1.90E-03	2.83E-02	-1.24E-01
410	686	441	699	475	702	508	693	517	664	529	637	3.46E-01	-1.11E-01	3.08E-01	2.11E-02	2.42E-01	-1.20E-01	1.87E-01	3.79E-03	1.26E-01	-1.22E-01	6.18E-02	-4.28E-03	7.28E-03	-1.21E-01
414	706	447	717	481	724	513	709	534	682	560	659	3.52E-01	-9.68E-02	2.98E-01	1.22E-02	2.57E-01	-1.16E-01	1.82E-01	-1.50E-03	1.37E-01	-1.26E-01	6.48E-02	-1.06E-02	1.50E-02	-1.25E-01
435	692	466	705	499	709	530	697	546	668	563	639	3.48E-01	-1.15E-01	3.00E-01	1.98E-02	2.40E-01	-1.27E-01	1.88E-01	8.91E-03	1.23E-01	-1.28E-01	6.83E-02	2.92E-03	4.01E-03	-1.26E-01
403	685	438	698	474	705	507	692	519	660	534	631	3.59E-01	-1.07E-01	2.94E-01	9.87E-03	2.54E-01	-1.17E-01	1.88E-01	-5.79E-03	1.40E-01	-1.18E-01	6.40E-02	-1.86E-03	2.27E-02	-1.10E-01
447	758	484	770	521	776	553	759	559	722	578	696	3.52E-01	-1.08E-01	2.93E-01	1.59E-02	2.47E-01	-1.23E-01	1.83E-01	-2.09E-03	1.33E-01	-1.31E-01	6.31E-02	-3.98E-03	1.81E-02	-1.24E-01
363	666	396	685	433	695	472	692	494	663	511	632	3.53E-01	-1.07E-01	3.01E-01	9.98E-03	2.54E-01	-1.24E-01	1.81E-01	-4.80E-03	1.41E-01	-1.32E-01	5.86E-02	-1.79E-03	3.19E-02	-1.30E-01
463	681	498	693	534	691	567	675	581	645	601	616	3.43E-01	-1.03E-01	2.99E-01	1.69E-02	2.51E-01	-1.15E-01	1.89E-01	-2.49E-03	1.47E-01	-1.19E-01	6.79E-02	1.37E-04	1.75E-02	-1.12E-01
452	716	486	727	520	728	548	711	557	677	579	653	3.56E-01	-1.01E-01	2.98E-01	1.13E-02	2.51E-01	-1.17E-01	1.83E-01	7.27E-04	1.42E-01	-1.19E-01	6.71E-02	-8.27E-03	2.57E-02	-1.17E-01
414	733	451	742	488	739	518	720	530	684	560	666	3.52E-01	-1.07E-01	2.99E-01	7.05E-03	2.47E-01	-1.21E-01	1.84E-01	-5.88E-04	1.29E-01	-1.25E-01	7.09E-02	-2.61E-04	2.37E-02	-1.19E-01
458	701	503	711	549	717	589	702	571	662	578	624	3.64E-01	-1.01E-01	2.95E-01	1.53E-02	2.58E-01	-1.20E-01	1.82E-01	-1.53E-03	1.37E-01	-1.23E-01	6.13E-02	-9.26E-05	1.40E-02	-1.16E-01
428	691	461	705	496	714	525	704	525	672	532	639	3.50E-01	-1.07E-01	2.93E-01	1.15E-02	2.44E-01	-1.17E-01	1.83E-01	-8.68E-04	1.29E-01	-1.19E-01	6.71E-02	-8.02E-03	1.33E-02	-1.18E-01
453	662	490	675	527	672	564	663	573	625	598	601	3.47E-01	-1.09E-01	2.97E-01	8.92E-03	2.52E-01	-1.22E-01	1.87E-01	-1.81E-03	1.38E-01	-1.27E-01	6.12E-02	3.05E-03	1.64E-02	-1.17E-01
514	762	556	768	597	774	638	777	649	743	662	710	3.39E-01	-1.10E-01	3.03E-01	1.13E-02	2.39E-01	-1.21E-01	1.89E-01	3.30E-03	1.29E-01	-1.18E-01	6.28E-02	4.04E-03	2.09E-02	-1.18E-01
395	686	434	697	476	699	510	679	515	640	539	614	3.49E-01	-1.01E-01	2.97E-01	1.35E-02	2.52E-01	-1.17E-01	1.88E-01	-2.71E-04	1.37E-01	-1.20E-01	6.86E-02	-1.75E-04	2.59E-02	-1.13E-01
489	730	535	724	575	704	617	724	636	683	601	650	3.55E-01	-1.05E-01	2.87E-01	2.20E-02	2.53E-01	-1.20E-01	1.84E-01	-1.19E-03	1.33E-01	-1.27E-01	6.09E-02	-6.19E-03	2.23E-02	-1.27E-01
448	763	487	780	524	781	563	762	595	740	614	702	3.49E-01	-1.14E-01	3.01E-01	8.39E-03	2.47E-01	-1.26E-01	1.80E-01	3.01E-03	1.36E-01	-1.27E-01	6.41E-02	2.28E-05	2.09E-02	-1.25E-01
376	776	408	793	445	801	479	787	497	757	506	722	3.52E-01	-1.10E-01	3.00E-01	2.09E-02	2.49E-01	-1.25E-01	1.88E-01	-3.86E-03	1.36E-01	-1.30E-01	6.85E-02	-1.81E-03	1.78E-02	-1.25E-01
414	740	444	751	476	759	503	744	511	712	528	694	3.68E-01	-1.08E-01	2.92E-01	9.96E-03	2.45E-01	-1.20E-01	1.74E-01	3.63E-03	1.37E-01	-1.19E-01	6.26E-02	6.65E-04	2.86E-02	-1.24E-01
475	756	566	768	659	765	749	748	734	680	708	601	3.57E-01	-9.39E-02	2.99E-01	9.30E-03	2.50E-01	-1.18E-01	1.81E-01	-4.16E-03	1.42E-01	-1.22E-01	6.56E-02	-8.75E-03	1.77E-02	-1.12E-01
421	1171	484	1187	551	1179	615	1165	663	1128	697	1071	3.66E-01	-8.81E-02	3.02E-01	1.64E-02	2.58E-01	-1.13E-01	1.84E-01	-5.46E-03	1.32E-01	-1.25E-01	6.12E-02	-9.98E-03	1.73E-02	-1.18E-01
407	948	464	970	526	981	579	956	612	903	652	859	3.64E-01	-9.15E-02	2.97E-01	4.33E-03	2.59E-01	-1.12E-01	1.84E-01	-5.92E-03	1.44E-01	-1.21E-01	7.14E-02	-8.80E-03	2.00E-02	-1.15E-01
311	1061	386	1088	465	1096	540	1074	567	1002	550	923	3.57E-01	-1.03E-01	2.91E-01	1.20E-02	2.44E-01	-1.21E-01	1.87E-01	-4.60E-04	1.33E-01	-1.25E-01	6.65E-02	-1.88E-03	1.79E-02	-1.24E-01
409	911	493	927	579	932	633	886	608	804	642	730	3.63E-01	-9.73E-02	3.00E-01	7.44E-03	2.60E-01	-1.15E-01	1.81E-01	-4.86E-03	1.39E-01	-1.16E-01	5.75E-02	-7.72E-04	1.46E-02	-1.09E-01
310	1078	392	1106	479	1115	558	1088	566	1010	617	945	3.65E-01	-1.01E-01	2.95E-01	8.44E-03	2.53E-01	-1.18E-01	1.79E-01	-2.24E-03	1.34E-01	-1.25E-01	6.30E-02	-2.27E-03	1.56E-02	-1.18E-01
333	1123	411	1159	496	1168	571	1132	621	1062	677	1001	3.62E-01	-1.06E-01	2.97E-01	1.09E-02	2.55E-01	-1.19E-01	1.82E-01	-2.14E-03	1.35E-01	-1.22E-01	6.38E-02	-4.33E-03	1.78E-02	-1.16E-01
385	955	469	982	555	1003	634	981	639	895	620	812	3.48E-01	-1.04E-01	3.01E-01	1.34E-02	2.47E-01	-1.11E-01	1.88E-01	2.52E-03	1.29E-01	-1.22E-01	6.79E-02	-1.94E-03	9.49E-03	-1.16E-01
238	1087	307	1101	377	1099	442	1073	475	1012	528	969	3.67E-01	-9.82E-02	2.99E-01	1.65E-02	2.57E-01	-1.19E-01	1.84E-01	3.69E-03	1.28E-01	-1.24E-01	5.64E-02	-2.82E-03	1.43E-03	-1.17E-01
268	962	349	994	436	995	511	954	549	875	596	809	3.53E-01	-1.00E-01	3.02E-01	1.78E-02	2.58E-01	-1.14E-01	1.93E-01	-2.76E-03	1.30E-01	-1.18E-01	5.50E-02	3.50E-03	1.05E-02	-1.15E-01
344	1053	439	1048	539	1067	607	1012	582	916	630	847	3.52E-01	-1.02E-01	3.00E-01	1.37E-02	2.46E-01	-1.15E-01	1.84E-01	-5.61E-04	1.28E-01	-1.23E-01	6.30E-02	8.74E-03	1.59E-02	-1.14E-01
345	951	425	963	502	966	566	931	596	855	659	809	3.70E-01	-8.36E-02	3.01E-01	5.83E-03	2.63E-01	-1.07E-01	1.86E-01	-6.13E-03	1.39E-01	-1.16E-01	6.10E-02	-6.61E-03	2.11E-02	-1.11E-01
311	1041	410	1047	508	1028	600	1011	607	913	667	844	3.56E-01	-9.17E-02	3.00E-01	9.42E-03	2.55E-01	-1.09E-01	1.86E-01	-3.43E-03	1.39E-01	-1.20E-01	6.21E-02	-4.50E-03	2.09E-02	-1.16E-01
267	1098	347	1097	419	1107	492	1101	517	1023	574	977	3.53E-01	-1.03E-01	3.01E-01	1.28E-02	2.46E-01	-1.13E-01	1.89E-01	-1.41E-03	1.36E-01	-1.22E-01	6.23E-02	-3.26E-03	1.24E-02	-1.21E-01

418	955	501	988	590	989	668	955	680	871	725	810	3.52E-01	-1.06E-01	3.05E-01	1.76E-02	2.43E-01	-1.25E-01	1.83E-01	1.07E-03	1.27E-01	-1.26E-01	6.73E-02	1.63E-03	9.74E-03	-1.21E-01
196	878	283	876	370	884	457	891	492	800	550	729	3.52E-01	-1.04E-01	3.00E-01	1.28E-02	2.51E-01	-1.19E-01	1.85E-01	5.43E-04	1.42E-01	-1.22E-01	5.77E-02	-7.03E-04	1.07E-02	-1.23E-01
218	784	324	796	422	768	527	759	546	656	601	575	3.55E-01	-9.48E-02	2.99E-01	9.85E-03	2.54E-01	-1.11E-01	1.84E-01	-2.79E-03	1.38E-01	-1.18E-01	6.24E-02	-3.30E-03	2.18E-02	-1.16E-01
268	912	363	925	461	917	544	887	547	793	606	723	3.73E-01	-8.82E-02	3.00E-01	9.17E-03	2.52E-01	-1.12E-01	1.87E-01	-4.29E-03	1.35E-01	-1.14E-01	6.33E-02	-7.50E-03	1.61E-02	-1.08E-01
286	938	358	955	430	972	479	941	489	871	530	810	3.60E-01	-1.01E-01	2.97E-01	1.00E-02	2.55E-01	-1.17E-01	1.81E-01	5.81E-04	1.34E-01	-1.21E-01	6.81E-02	-5.55E-03	1.40E-02	-1.19E-01
322	997	393	1022	467	1024	536	996	569	928	622	879	3.66E-01	-9.84E-02	2.97E-01	1.79E-02	2.57E-01	-1.15E-01	1.82E-01	-8.01E-03	1.54E-01	-1.24E-01	6.41E-02	-9.58E-03	8.65E-03	-1.21E-01
371	980	449	1004	532	1010	609	985	618	904	653	847	3.51E-01	-9.71E-02	2.90E-01	1.08E-02	2.50E-01	-1.14E-01	1.84E-01	-3.71E-03	1.37E-01	-1.19E-01	6.59E-02	-7.14E-03	1.49E-02	-1.14E-01
345	1047	431	1058	519	1056	599	1025	630	944	659	864	3.67E-01	-9.30E-02	2.94E-01	8.90E-03	2.55E-01	-1.12E-01	1.81E-01	-7.42E-03	1.36E-01	-1.15E-01	6.52E-02	-4.78E-03	2.29E-02	-1.10E-01
425	959	518	935	595	982	664	944	635	851	672	771	3.64E-01	-9.48E-02	2.97E-01	1.47E-02	2.49E-01	-1.13E-01	1.83E-01	-9.25E-04	1.31E-01	-1.15E-01	6.48E-02	-2.06E-03	1.82E-02	-1.09E-01
345	1065	430	1097	515	1116	601	1098	602	1007	616	919	3.54E-01	-9.48E-02	2.98E-01	7.86E-03	2.55E-01	-1.16E-01	1.87E-01	-2.65E-03	1.38E-01	-1.21E-01	6.56E-02	-2.23E-03	2.13E-02	-1.19E-01
435	1042	523	1061	612	1061	698	1043	652	967	648	887	3.69E-01	-9.96E-02	2.88E-01	1.82E-02	2.60E-01	-1.15E-01	1.80E-01	-1.95E-03	1.35E-01	-1.19E-01	6.12E-02	-2.53E-03	1.53E-02	-1.16E-01
491	1002	558	1015	624	1022	688	1014	690	949	712	897	3.72E-01	-8.85E-02	2.91E-01	1.54E-02	2.64E-01	-1.15E-01	1.80E-01	-5.48E-03	1.48E-01	-1.17E-01	6.00E-02	-7.50E-03	2.22E-02	-1.12E-01
384	855	448	874	513	884	573	864	602	807	641	757	3.56E-01	-9.84E-02	2.93E-01	7.94E-03	2.51E-01	-1.13E-01	1.80E-01	-1.01E-03	1.45E-01	-1.18E-01	6.03E-02	-5.60E-03	1.07E-02	-1.14E-01
435	1392	516	1409	597	1397	665	1354	672	1273	727	1217	3.63E-01	-9.65E-02	3.01E-01	3.38E-03	2.47E-01	-1.19E-01	1.80E-01	-2.97E-03	1.40E-01	-1.28E-01	5.79E-02	-5.63E-03	1.43E-02	-1.18E-01
336	1117	417	1135	502	1137	578	1102	599	1025	650	967	3.48E-01	-9.65E-02	3.00E-01	1.07E-02	2.52E-01	-1.18E-01	1.86E-01	-2.56E-03	1.44E-01	-1.25E-01	6.18E-02	-7.88E-04	2.91E-02	-1.24E-01
421	1187	495	1207	572	1212	645	1196	639	1119	648	1048	3.72E-01	-8.00E-02	3.09E-01	1.27E-02	2.55E-01	-1.03E-01	1.83E-01	-9.46E-03	1.32E-01	-1.15E-01	6.80E-02	-1.36E-02	9.02E-03	-1.06E-01
275	999	358	1028	448	1035	531	1006	563	933	616	869	3.54E-01	-1.02E-01	2.97E-01	1.02E-02	2.53E-01	-1.20E-01	1.85E-01	-1.31E-03	1.35E-01	-1.21E-01	6.09E-02	1.11E-03	3.09E-02	-1.19E-01
355	939	426	979	504	1004	578	985	644	938	688	869	3.57E-01	-1.00E-01	2.95E-01	4.52E-03	2.55E-01	-1.10E-01	1.83E-01	-2.95E-03	1.38E-01	-1.14E-01	6.41E-02	-8.46E-03	1.95E-02	-1.08E-01
295	1094	399	1121	505	1133	582	1075	529	982	458	911	3.66E-01	-9.18E-02	2.91E-01	1.26E-02	2.61E-01	-1.14E-01	1.86E-01	-6.01E-03	1.44E-01	-1.21E-01	5.90E-02	-4.47E-03	2.79E-02	-1.14E-01
370	1134	438	1163	512	1181	586	1180	615	1111	666	1058	3.73E-01	-1.02E-01	3.06E-01	1.19E-02	2.48E-01	-1.12E-01	1.83E-01	-1.67E-03	1.29E-01	-1.17E-01	5.44E-02	-5.19E-03	2.03E-02	-1.17E-01
354	1058	424	1083	497	1091	563	1069	603	1005	653	958	3.55E-01	-9.87E-02	3.00E-01	1.48E-02	2.50E-01	-1.15E-01	1.80E-01	5.85E-03	1.34E-01	-1.23E-01	6.24E-02	-1.05E-03	1.45E-02	-1.23E-01
297	744	382	781	472	786	558	755	619	690	653	604	3.73E-01	-1.04E-01	3.00E-01	8.59E-03	2.48E-01	-1.19E-01	1.77E-01	-6.82E-04	1.33E-01	-1.24E-01	6.63E-02	-5.95E-03	1.44E-02	-1.19E-01
308	774	385	782	464	781	536	750	545	672	600	626	3.72E-01	-1.12E-01	3.00E-01	1.39E-02	2.51E-01	-1.11E-01	1.82E-01	5.32E-04	1.28E-01	-1.20E-01	5.25E-02	4.22E-03	1.57E-02	-1.17E-01
316	762	386	797	467	802	539	776	568	709	595	634	3.60E-01	-9.56E-02	2.94E-01	9.81E-03	2.52E-01	-1.19E-01	1.84E-01	-7.87E-04	1.36E-01	-1.23E-01	5.96E-02	-4.64E-03	1.05E-02	-1.17E-01
375	575	452	611	537	629	617	612	614	535	617	460	3.73E-01	-8.82E-02	3.09E-01	7.79E-03	2.56E-01	-1.04E-01	1.86E-01	-6.20E-03	1.30E-01	-1.14E-01	5.93E-02	-3.61E-03	2.17E-03	-1.06E-01
178	542	221	560	265	568	309	559	331	522	352	485	3.54E-01	-1.06E-01	2.91E-01	1.14E-02	2.58E-01	-1.23E-01	1.76E-01	-5.46E-03	1.44E-01	-1.28E-01	6.37E-02	-9.24E-03	3.15E-02	-1.25E-01
210	604	243	619	278	629	313	627	325	593	347	564	3.79E-01	-1.05E-01	2.82E-01	1.41E-02	2.69E-01	-1.25E-01	1.71E-01	-6.36E-03	1.38E-01	-1.32E-01	5.83E-02	-9.28E-03	1.87E-02	-1.25E-01
153	491	188	505	226	507	259	494	273	458	300	432	3.60E-01	-9.66E-02	2.98E-01	1.18E-02	2.52E-01	-1.15E-01	1.88E-01	-5.43E-03	1.37E-01	-1.21E-01	6.99E-02	-1.12E-02	1.18E-02	-1.16E-01
274	609	305	618	339	621	363	604	378	574	404	557	3.61E-01	-9.86E-02	2.97E-01	1.45E-02	2.44E-01	-1.20E-01	1.93E-01	-6.99E-03	1.32E-01	-1.28E-01	6.62E-02	-5.34E-03	9.75E-03	-1.23E-01
122	463	159	482	200	490	240	487	257	453	265	416	3.59E-01	-1.08E-01	2.97E-01	1.41E-02	2.38E-01	-1.23E-01	1.82E-01	8.21E-04	1.36E-01	-1.29E-01	6.48E-02	-7.19E-04	2.08E-02	-1.21E-01
148	617	187	628	227	630	265	619	279	582	306	555	3.57E-01	-9.72E-02	2.89E-01	1.21E-02	2.55E-01	-1.17E-01	1.76E-01	-2.59E-03	1.35E-01	-1.25E-01	7.24E-02	-1.15E-02	2.05E-02	-1.18E-01
145	461	185	471	227	471	261	450	275	412	289	373	3.72E-01	-9.89E-02	2.91E-01	1.26E-02	2.62E-01	-1.19E-01	1.71E-01	-5.52E-03	1.41E-01	-1.28E-01	6.07E-02	-1.46E-02	2.45E-02	-1.23E-01
155	449	193	459	232	469	264	455	274	417	303	392	3.55E-01	-1.07E-01	2.90E-01	1.54E-02	2.53E-01	-1.25E-01	1.79E-01	-4.59E-03	1.34E-01	-1.26E-01	6.55E-02	-2.41E-03	1.82E-02	-1.17E-01
147	569	186	588	227	601	266	596	286	562	303	525	3.52E-01	-1.06E-01	2.97E-01	1.32E-02	2.45E-01	-1.20E-01	1.82E-01	1.75E-03	1.36E-01	-1.31E-01	6.90E-02	-7.47E-03	1.21E-02	-1.24E-01
218	568	251	583	287	585	323	578	347	552	367	522	3.70E-01	-1.02E-01	2.93E-01	1.04E-02	2.63E-01	-1.13E-01	1.88E-01	-1.83E-03	1.30E-01	-1.22E-01	6.41E-02	-3.81E-03	1.55E-02	-1.10E-01
190	669	230	678	269	679	306	665	322	628	348	600	3.70E-01	-9.86E-02	2.93E-01	9.88E-03	2.60E-01	-1.17E-01	1.79E-01	-5.58E-03	1.44E-01	-1.21E-01	6.09E-02	-3.65E-03	1.66E-02	-1.16E-01
180	574	213	585	249	591	282	582	300	553	315	523	3.61E-01	-1.08E-01	2.96E-01	2.28E-02	2.51E-01	-1.11E-01	1.90E-01	3.74E-03	1.35E-01	-1.24E-01	6.39E-02	-2.49E-03	1.76E-02	-1.18E-01
197	518	237	529	278	530	311	506	333	472	366	448	3.62E-01	-9.97E-02	2.97E-01	1.63E-02	2.53E-01	-1.21E-01	1.81E-01	-1.60E-03	1.28E-01	-1.26E-01	6.68E-02	-2.17E-03	1.30E-02	-1.21E-01
155	587	188	595	222	598	244	577	261	547	288	529	3.60E-01	-1.03E-01	2.94E-01	8.86E-03	2.54E-01	-1.12E-01	1.94E-01	-6.69E-03	1.45E-01	-1.19E-01	6.53E-02	-7.88E-03	1.72E-02	-1.07E-01
165	461	197	475	230	483	261	472	278	442	307	426	3.65E-01	-1.10E-01	2.96E-01	2.30E-02	2.55E-01	-1.25E-01	1.83E-01	-5.92E-04	1.33E-01	-1.35E-01	6.08E-02	-4.04E-03	4.67E-03	-1.23E-01
103	483	138	499	176	503	213	495	242	470	270	443	3.59E-01	-9.50E-02	2.94E-01	1.39E-02	2.58E-01	-1.11E-01	1.87E-01	-7.77E-03	1.45E-01	-1.20E-01	6.24E-02	-1.13E-02	2.18E-02	-1.15E-01
159	556	204	560	249	554	283	526	291	481	314	445	3.56E-01	-9.92E-02	2.89E-01	1.08E-02	2.57E-01	-1.14E-01	1.83E-01	-6.24E-03	1.44E-01	-1.21E-01	6.60E-02	-5.16E-03	2.58E-02	-1.19E-01
75	472	111	498	152	512	195	506	218	472	241	437	3.42E-01	-1.11E-01	2.95E-01	1.00E-02	2.46E-01	-1.16E-01	1.82E-01	6.60E-03	1.29E-01	-1.19E-01	6.45E-02	5.17E-03	1.99E-02	-1.11E-01
167	445	203	459	240	468	277	461	283	423	312	401	3.42E-01	-1.11E-01	2.99E-01	1.58E-02	2.47E-01	-1.17E-01	1.85E-01	1.97E-03	1.30E-01	-1.22E-01	6.77E-02	3.28E-03	1.91E-02	-1.15E-01
175	529	223	538	271	535	312	512	315	465	335	429	3.56E-01	-9.23E-02	2.84E-01	1.83E-02	2.54E-01	-1.15E-01	1.87E-01	-7.40E-03	1.38E-01	-1.28E-01	6.66E-02	-3.88E-04	2.13E-02	-1.20E-01
125	477	151	500	183	515	218	519	243	495	246	460	3.59E-01	-1.08E-01	2.97E-01	2.19E-02	2.52E-01	-1.20E-01	1.80E-01	-2.00E-03	1.30E-01	-1.32E-01	6.56E-02	-7.38E-03	8.67E-03	-1.22E-01
145	503	180	519	219	527	250	516	249	478	269	448	3.64E-01	-9.57E-02	2.95E-01	1.72E-02	2.67E-01	-1.19E-01	1.80E-01	-7.79E-04	1.30E-01	-1.24E-01	5.96E-02	-1.04E-02	1.05E-02	-1.13E-01
429	683	465	684	500	691	527	674	541	640	571	629	3.51E-01	-1.01E-01	3.01E-01	6.83E-03	2.38E-01	-1.21E-01	1.92E-01	-4.64E-03	1.34E-01	-1.26E-01	7.24E-02	-7.25E-03	1.12E-02	-1.18E-01
355	698	387	707	420	712	454	712	465	683	484	655	3.61E-01	-9.19E-02	3.02E-01	1.14E-02	2.46E-01	-1.13E-01	1.92E-01	-6.43E-03	1.33E-01	-1.24E-01	6.15E-02	-1.03E-02	1.77E-02	-1.19E-01
474	735	503	747	536	753	567	751	582	724	595	700	3.62E-01	-9.15E-02	3.00E-01	8.84E-03	2.42E-01	-1.14E-01	1.96E-01	-6.08E-03	1.36E-01	-1.22E-01	5.77E-02	-8.22E-03	1.46E-02	-1.19E-01
379	744	417	756	454	761	491	748	521	726	558	712	3.32E-01	-1.14E-01	2.94E-01	6.95E-03	2.41E-01	-1.20E-01	1.89E-01	2.72E-03	1.50E-01	-1.07E-01	7.05E-02	-1.21E-03	4.32E-02	-1.12E-01
338	706	368	718	401	718	426	697	453	678	485	671	3.43E-01	-1.03E-01	3.06E-01	5.93E-03	2.40E-01	-1.13E-01	1.92E-01	2.01E-03	1.32E-01	-1.16E-01	7.39E-02	-5.83E-05	1.45E-02	-1.09E-01
338	685	368	704	401	718	437	715	450	685	482	671	3.42E-01	-1.14E-01	3.00E-01	4.94E-03	2.37E-01	-1.28E-01	1.90E-01	1.38E-04	1.30E-01	-1.27E-01	7.04E-02	-3.87E-04	2.43E-02	-1.12E-01
306	686	341	699	380	700	417	688	448	665	486	658	3.47E-01	-1.15E-01	2.95E-01	3.85E-03	2.38E-01	-1.23E-01	2.00E-01	-5.19E-03	1.29E-01	-1.20E-01	7.85E-02	-6.27E-03	2.04E-02	-1.13E-01
375	733	408	748	443	761	471	741	491	710	522	690	3.53E-01	-9.85E-02	3.06E-01	1.33E-02	2.48E-01	-1.13E-01	1.85E-01	1.44E-03	1.38E-01	-1.20E-01	6.03E-02	1.00E-03	2.34E-02	-1.16E-01
362	753	397	765	433	772	470	771	493	745	525	727	3.33E-01	-1.12E-01	2.99E-01	1.05E-03	2.40E-01	-1.20E-01	1.90E-01	1.13E-03	1.41E-01	-1.15E-01	6.21E-02	5.25E-03	1.65E-02	-1.14E-01
341	662	380	673	422	674	458	656	490	630	525	609	3.42E-01	-1.01E-01	2.98E-01	1.18E-02	2.50E-01	-1.22E-01	1.85E-01	-2.43E-05	1.37E-01	-1.23E-01	7.04E-02	-1.61E-03	3.18E-02	-1.20E-01
407	719	447	740	491	753	533	739	532	694	555	662	3.41E-01	-1.08E-01	2.96E-01	8.20E-03	2.54E-01	-1.18E-01	1.97E-01	-8.74E-04	1.33E-01	-1.23E-01	7.62E-02	-3.67E-03	1.89E-02	-1.12E-01
748	1145	795	1155	843	1163	878	1139	898	1095	930	1062	3.42E-01	-1.10E-01	3.02E-01	8.04E-03	2.37E-01	-1.20E-01	1.90E-01	3.27E-03	1.38E-01	-1.21E-01	7.18E-02	1.19E-03	2.64E-02	-1.20E-01
794	1210	845	1221	897	1220	945	1199	983	1163	1029	1140	3.40E-01	-1.09E-01	2.92E-01	7.91E-03	2.41E-01	-1.24E-01	1.96E-01	2.49E-03	1.54E-01	-1.33E-01	7.41E-02	-7.16E-03	1.52E-02	-1.29E-01
725	1124	772	1134	819	1133	864	1115	893	1076	936	1053	3.44E-01	-9.56E-02	3.01E-01	6.85E-03	2.40E-01	-1.15E-01	1.97E-01	9.16E-04	1.42E-01	-1.21E-01	7.24E-02	-3.09E-03	4.10E-03	-1.13E-01
673	1163	711	1175	751	1175	780	1147	812	1123	851	1111	3.52E-01	-1.00E-01	3.04E-01	1.14E-02	2.53E-01	-1.22E-01	1.91E-01	-3.69E-03	1.35E-01	-1.26E-01	6.88E-02	-3.27E-03	2.77E-02	-1.21E-01
754	1121	797	1132	840	1130	877	1110	914	1088	957	1079	3.50E-01	-1.06E-01	3.01E-01	3.91E-03	2.34E-01	-1.25E-01	2.01E-01	-3.30E-03	1.22E-01	-1.28E-01	9.14E-02	-2.97E-03	2.26E-02	-1.17E-01
818	1033	867	1048	918	1053	946	1014	968	966	1008	936	3.62E-01	-9.69E-02	2.93E-01	9.37E-03	2.54E-01	-1.26E-01	1.86E-01	-5.31E-03	1.37E-01	-1.31E-01	7.02E-02	-2.73E-03	1.36E-02	-1.19E-01
751	1086	793	1101	839	1105	877	1084	919	1067	963	1058	3.54E-01	-1.09E-01	2.95E-01	1.06E-03	2.35E-01	-1.26E-01	1.99E-01	-2.51E-03	1.21E-01	-1.26E-01	8.98E-02	-3.60E-03	2.38E-02	-1.20E-01
394	724	419	734	445	741	471	731	488	711	509	694	3.50E-01	-1.03E-01	3.02E-01	1.24E-02	2.53E-01	-1.20E-01	1.94E-01	-2.72E-03	1.36E-01	-1.27E-01	6.29E-02	-1.23E-03	1.47E-02	-1.19E-01
440	732	471	742	503	746	527	726	515	697	527	669	3.45E-01	-1.10E-01	2.94E-01	1.16E-02	2.48E-01	-1.25E-01	1.85E-01	2.26E-04	1.47E-01	-1.32E-01	6.82E-02	-3.33E-03	2.59E-02	-1.23E-01
422	673	447	670	471	660	496	652	518	638	543	631	3.56E-01	-9.96E-02	3.00E-01	5.10E-03	2.57E-01	-1.22E-01	1.86E-01	2.91E-04	1.45E-01	-1.28E-01	6.60E-02	3.14E-03	1.67E-02	-1.15E-01
424	723	447	734	470	736	490	722	506	702	530	694	3.60E-01	-1.08E-01	2.96E-01	9.18E-03	2.53E-01	-1.30E-01	1.94E-01	-1.39E-03	1.32E-01	-1.29E-01	6.91E-02	-1.96E-03	9.66E-03	-1.21E-01
345	689	371	708	402	711	429	695	452	672	479	654	3.41E-01	-1.13E-01	3.00E-01	6.92E-03	2.49E-01	-1.21E-01	1.95E-01	1.82E-03	1.42E-01	-1.28E-01	7.13E-02	6.05E-03	3.08E-02	-1.26E-01
473	739	503	749	534	752	559	739	562	707	575	681	3.55E-01	-1.13E-01	2.99E-01	3.41E-03	2.49E-01	-1.26E-01	1.94E-01	8.55E-04	1.32E-01	-1.27E-01	5.54E-02	6.04E-03	1.89E-02	-1.16E-01
456	703	485	712	515	712	541	700	552	675	561	654	3.53E-01	-1.01E-01	3.00E-01	1.58E-02	2.54E-01	-1.22E-01	1.89E-01	-3.12E-03	1.38E-01	-1.30E-01	7.07E-02	-1.02E-02	1.54E-02	-1.18E-01
388	679	418	692	449	705	483	704	501	680	505	646	3.55E-01	-1.02E-01	2.95E-01	9.13E-03	2.54E-01	-1.20E-01	1.84E-01	-1.29E-03	1.41E-01	-1.26E-01	6.37E-02	-5.63E-03	2.76E-02	-1.18E-01
332	709	369	725	408	730	437	707	462	678	500	667	3.45E-01	-1.15E-01	2.93E-01	8.34E-03	2.37E-01	-1.20E-01	1.90E-01	-3.47E-03	1.28E-01	-1.20E-01	6.55E-02	6.46E-03	2.45E-02	-1.19E-01
364	715	393	728	427	737	462	739	493	721	526	710	3.56E-01	-1.17E-01	3.00E-01	6.54E-03	2.52E-01	-1.21E-01	2.00E-01	-7.74E-03	1.28E-01	-1.15E-01	6.98E-02	-3.72E-03	8.51E-03	-1.09E-01
330	645	363	655	393	656	423	646	441	617	472	604	3.55E-01	-1.02E-01	3.00E-01	1.31E-02	2.45E-01	-1.13E-01	1.87E-01	-1.19E-03	1.36E-01	-1.23E-01	7.24E-02	-8.35E-04	1.96E-02	-1.21E-01
391	643	425	652	460	657	494	646	518	619	543	600	3.42E-01	-1.11E-01	2.90E-01	1.25E-02	2.43E-01	-1.22E-01	1.88E-01	-1.89E-03	1.39E-01	-1.27E-01	7.28E-02	-1.14E-02	2.08E-02	-1.22E-01
460	704	494	716	529	728	564	726	587	697	620	686	3.34E-01	-1.18E-01	2.96E-01	1.27E-02	2.25E-01	-1.30E-01	1.87E-01	1.58E-03	1.27E-01	-1.24E-01	7.42E-02	-3.15E-03	1.04E-02	-1.25E-01
463	690	499	700	536	707	567	689	587	658	615	633	3.57E-01	-9.27E-02	3.06E-01	1.57E-02	2.64E-01	-1.08E-01	2.03E-01	-7.12E-03	1.30E-01	-1.17E-01	6.96E-02	-1.67E-02	4.91E-03	-1.18E-01
417	736	454	743	492	747	518	732	543	705	577	691	3.40E-01	-1.01E-01	2.97E-01	9.32E-03	2.53E-01	-1.15E-01	1.92E-01	-9.86E-03	1.36E-01	-1.20E-01	7.63E-02	-4.59E-03	1.90E-02	-1.14E-01

PrCoor_x8	PrCoor_y8	PrCoor_x9	PrCoor_y9	PrCoor_x10	PrCoor_y10	PrCoor_x11	PrCoor_y11	PrCoor_x12	PrCoor_y12	PrCoor_x13	PrCoor_y13	PrCoor_x14	PrCoor_y14	PrCoor_x15	PrCoor_y15	PrCoor_x16	PrCoor_y16	PrCoor_x17	PrCoor_y17	PrCoor_x18	PrCoor_y18	PrCoor_x19	PrCoor_y19	PrCoor_x20	PrCoor_y20
-2.04E-02	-1.28E-02	-9.46E-02	-1.11E-01	-1.84E-02	8.47E-02	1.56E-02	-2.40E-04	7.33E-02	9.76E-02	1.24E-01	-4.46E-03	1.81E-01	9.30E-02	2.28E-01	7.17E-03	2.93E-01	1.04E-01	-1.25E-01	-4.02E-02	-1.35E-01	-3.79E-02	-1.45E-01	-3.98E-02	-1.70E-01	-4.09E-02
-2.10E-02	-2.04E-02	-9.69E-02	-9.82E-02	-3.23E-02	7.71E-02	-1.35E-03	1.86E-03	6.62E-02	9.63E-02	1.23E-01	-1.26E-02	1.84E-01	8.98E-02	2.36E-01	-7.20E-04	2.97E-01	1.01E-01	-1.17E-01	-3.52E-02	-1.30E-01	-3.57E-02	-1.45E-01	-3.82E-02	-1.73E-01	-4.05E-02
-4.38E-02	-3.34E-03	-9.59E-02	-9.26E-02	-1.22E-02	8.83E-02	5.17E-03	-3.46E-03	8.73E-02	1.01E-01	1.20E-01	-7.26E-03	1.98E-01	9.95E-02	2.27E-01	-4.12E-03	3.16E-01	9.24E-02	-1.31E-01	-1.30E-03	-1.38E-01	-1.35E-02	-1.46E-01	-3.44E-02	-1.68E-01	-4.62E-02
-3.78E-02	-6.92E-03	-9.08E-02	-9.53E-02	-1.63E-03	7.82E-02	-2.16E-03	-2.01E-04	8.64E-02	9.67E-02	1.14E-01	-5.76E-03	2.00E-01	9.91E-02	2.32E-01	-1.71E-03	3.07E-01	1.03E-01	-1.27E-01	-2.00E-02	-1.42E-01	-2.49E-02	-1.56E-01	-3.70E-02	-1.79E-01	-4.80E-02
-2.74E-02	-1.76E-02	-9.99E-02	-9.66E-02	-4.92E-03	7.48E-02	1.17E-02	-1.15E-02	8.65E-02	9.54E-02	1.19E-01	-9.81E-03	1.94E-01	9.48E-02	2.29E-01	1.37E-03	2.96E-01	1.12E-01	-1.24E-01	-2.39E-02	-1.38E-01	-2.75E-02	-1.53E-01	-3.67E-02	-1.80E-01	-3.79E-02
-4.29E-02	-7.74E-03	-8.77E-02	-1.03E-01	-5.12E-03	7.80E-02	8.02E-03	-8.31E-05	7.75E-02	9.80E-02	1.20E-01	-1.13E-02	1.87E-01	9.63E-02	2.28E-01	-6.81E-03	2.93E-01	1.12E-01	-1.17E-01	-2.52E-02	-1.29E-01	-2.93E-02	-1.42E-01	-3.87E-02	-1.69E-01	-4.25E-02
-5.56E-02	-2.07E-03	-1.09E-01	-1.04E-01	-2.85E-03	8.05E-02	4.63E-03	-5.90E-03	9.96E-02	1.02E-01	1.11E-01	-4.00E-03	2.06E-01	9.53E-02	2.26E-01	-1.60E-03	2.97E-01	9.32E-02	-1.32E-01	-2.40E-02	-1.47E-01	-2.63E-02	-1.63E-01	-3.52E-02	-1.85E-01	-4.43E-02
-3.03E-02	-1.69E-02	-1.03E-01	-9.29E-02	-1.63E-02	8.14E-02	9.19E-03	-1.04E-03	7.65E-02	9.50E-02	1.27E-01	-9.26E-03	1.91E-01	9.90E-02	2.30E-01	-2.50E-03	3.07E-01	1.04E-01	-1.24E-01	-1.39E-02	-1.37E-01	-2.00E-02	-1.51E-01	-3.20E-02	-1.75E-01	-3.61E-02
-2.07E-02	-1.20E-02	-9.81E-02	-9.84E-02	-2.46E-02	7.72E-02	5.84E-03	2.31E-03	8.07E-02	1.00E-01	1.19E-01	-2.05E-03	1.97E-01	1.01E-01	2.27E-01	9.64E-05	3.05E-01	9.82E-02	-1.09E-01	-2.29E-02	-1.24E-01	-2.74E-02	-1.41E-01	-3.89E-02	-1.70E-01	-4.66E-02
-4.99E-02	-3.99E-03	-9.21E-02	-9.84E-02	-4.24E-03	7.86E-02	9.13E-03	-6.81E-04	8.43E-02	9.66E-02	1.19E-01	-9.65E-03	1.97E-01	9.72E-02	2.32E-01	-3.10E-03	3.07E-01	1.13E-01	-1.23E-01	-3.17E-02	-1.35E-01	-3.28E-02	-1.49E-01	-3.89E-02	-1.75E-01	-4.32E-02
-2.10E-02	-1.47E-02	-1.07E-01	-9.14E-02	-3.39E-02	7.69E-02	4.64E-03	-2.30E-03	7.68E-02	1.01E-01	1.29E-01	-5.34E-03	1.98E-01	9.52E-02	2.46E-01	2.19E-03	3.19E-01	1.01E-01	-1.15E-01	-2.90E-02	-1.27E-01	-2.92E-02	-1.42E-01	-3.11E-02	-1.70E-01	-3.20E-02
-1.64E-02	-1.46E-02	-9.53E-02	-1.02E-01	-1.68E-02	8.75E-02	1.03E-02	-2.88E-03	7.99E-02	1.00E-01	1.21E-01	-1.04E-02	1.80E-01	9.57E-02	2.33E-01	-7.70E-04	2.77E-01	9.92E-02	-1.20E-01	-2.01E-02	-1.34E-01	-1.99E-02	-1.47E-01	-2.74E-02	-1.71E-01	-2.88E-02
-3.99E-02	-1.58E-02	-1.03E-01	-8.73E-02	-5.60E-02	7.27E-02	1.61E-04	-8.70E-03	5.89E-02	1.01E-01	1.15E-01	-1.38E-02	1.81E-01	1.00E-01	2.42E-01	-1.16E-02	2.83E-01	1.02E-01	-1.18E-01	-1.79E-02	-1.29E-01	-1.89E-02	-1.40E-01	-2.66E-02	-1.60E-01	-2.72E-02
-3.26E-02	-1.40E-02	-9.94E-02	-9.94E-02	-3.27E-02	8.54E-02	7.75E-03	1.92E-03	7.02E-02	1.08E-01	1.19E-01	-8.12E-03	1.85E-01	1.02E-01	2.33E-01	-5.42E-03	2.94E-01	1.02E-01	-1.20E-01	-2.39E-02	-1.32E-01	-3.00E-02	-1.46E-01	-3.98E-02	-1.69E-01	-4.40E-02
-3.15E-02	-1.57E-02	-9.15E-02	-9.69E-02	-2.06E-02	8.72E-02	-3.04E-03	-2.89E-03	7.41E-02	1.04E-01	1.21E-01	-1.13E-02	1.88E-01	9.96E-02	2.35E-01	-7.08E-03	2.98E-01	1.02E-01	-1.21E-01	-3.22E-02	-1.35E-01	-3.74E-02	-1.49E-01	-5.26E-02	-1.73E-01	-5.81E-02
-1.90E-02	-1.46E-02	-9.87E-02	-1.09E-01	-2.37E-02	7.51E-02	4.73E-03	-2.13E-03	8.09E-02	9.31E-02	1.17E-01	-7.15E-03	2.05E-01	8.51E-02	2.30E-01	-5.05E-04	2.98E-01	1.01E-01	-1.01E-01	9.09E-03	-1.14E-01	-1.47E-03	-1.33E-01	-2.67E-02	-1.66E-01	-5.13E-02
-2.35E-02	-9.04E-03	-1.01E-01	-1.06E-01	-1.65E-02	9.00E-02	1.03E-02	-5.86E-04	8.18E-02	1.07E-01	1.19E-01	2.89E-03	2.05E-01	1.03E-01	2.32E-01	2.13E-03	3.02E-01	1.08E-01	-1.22E-01	-2.61E-02	-1.37E-01	-2.96E-02	-1.51E-01	-4.07E-02	-1.75E-01	-4.41E-02
-3.39E-02	-1.94E-02	-1.19E-01	-9.28E-02	-1.53E-02	8.29E-02	-7.83E-03	-8.39E-03	7.79E-02	9.54E-02	1.18E-01	-1.29E-02	1.88E-01	9.78E-02	2.30E-01	-7.00E-04	2.94E-01	1.08E-01	-1.33E-01	-3.03E-02	-1.45E-01	-3.17E-02	-1.57E-01	-3.70E-02	-1.76E-01	-3.60E-02
-1.67E-02	-1.13E-02	-9.88E-02	-8.80E-02	-2.95E-02	8.52E-02	5.05E-03	-2.21E-03	7.50E-02	1.03E-01	1.21E-01	-5.96E-03	1.92E-01	1.01E-01	2.34E-01	-5.37E-03	3.04E-01	9.36E-02	-1.14E-01	-2.28E-02	-1.30E-01	-2.90E-02	-1.43E-01	-4.81E-02	-1.69E-01	-5.32E-02
-2.53E-02	-1.68E-02	-1.17E-01	-1.07E-01	-1.68E-02	8.80E-02	1.15E-02	-1.06E-02	7.81E-02	1.05E-01	1.18E-01	-1.10E-02	1.89E-01	1.11E-01	2.24E-01	7.49E-04	3.00E-01	1.21E-01	-1.19E-01	-1.54E-02	-1.36E-01	-2.06E-02	-1.58E-01	-3.48E-02	-1.89E-01	-4.51E-02
-2.01E-02	-1.82E-03	-1.04E-01	-1.01E-01	-1.93E-02	9.24E-02	6.06E-03	6.11E-03	7.57E-02	1.09E-01	1.16E-01	1.61E-03	1.97E-01	1.06E-01	2.27E-01	1.99E-03	3.15E-01	9.68E-02	-1.20E-01	-2.53E-02	-1.35E-01	-3.26E-02	-1.51E-01	-4.78E-02	-1.78E-01	-5.35E-02
-3.41E-02	-8.89E-03	-1.01E-01	-1.04E-01	-6.43E-03	8.00E-02	2.29E-02	-6.59E-03	8.54E-02	9.25E-02	1.21E-01	-3.69E-03	1.92E-01	9.28E-02	2.33E-01	1.17E-03	3.04E-01	9.98E-02	-1.25E-01	-1.01E-02	-1.42E-01	-1.41E-02	-1.61E-01	-3.01E-02	-1.86E-01	-4.67E-02
-2.38E-02	-5.30E-03	-1.12E-01	-9.48E-02	-1.82E-03	8.44E-02	8.54E-03	-4.16E-03	8.92E-02	9.81E-02	1.23E-01	-8.63E-03	1.96E-01	9.75E-02	2.35E-01	5.28E-03	3.00E-01	1.05E-01	-1.24E-01	-3.06E-02	-1.37E-01	-3.39E-02	-1.51E-01	-4.14E-02	-1.74E-01	-4.08E-02
-4.56E-02	-8.92E-03	-1.02E-01	-9.88E-02	-1.91E-02	8.13E-02	-6.26E-03	-3.80E-03	8.03E-02	9.90E-02	1.17E-01	-1.53E-02	1.85E-01	1.01E-01	2.34E-01	-6.38E-03	2.93E-01	1.03E-01	-1.30E-01	-1.73E-02	-1.41E-01	-2.34E-02	-1.54E-01	-3.65E-02	-1.77E-01	-4.24E-02
-2.70E-02	-1.08E-02	-1.06E-01	-8.76E-02	-1.84E-02	7.67E-02	4.55E-03	-9.11E-03	8.00E-02	9.72E-02	1.20E-01	-6.90E-03	1.92E-01	9.31E-02	2.30E-01	2.86E-04	2.99E-01	9.68E-02	-1.29E-01	-2.36E-02	-1.43E-01	-2.94E-02	-1.57E-01	-4.05E-02	-1.80E-01	-4.39E-02
-2.52E-02	-1.68E-02	-9.48E-02	-8.81E-02	-2.17E-02	8.10E-02	-5.89E-04	-3.18E-03	7.48E-02	9.63E-02	1.14E-01	-1.09E-02	1.93E-01	9.28E-02	2.30E-01	-2.06E-03	3.09E-01	9.71E-02	-1.21E-01	-1.98E-02	-1.34E-01	-2.47E-02	-1.46E-01	-3.68E-02	-1.69E-01	-3.96E-02
-2.88E-02	-1.67E-02	-1.01E-01	-9.96E-02	-2.11E-02	7.63E-02	5.88E-04	-9.02E-03	7.58E-02	9.20E-02	1.20E-01	-1.15E-02	1.87E-01	9.49E-02	2.34E-01	-5.96E-04	2.97E-01	1.00E-01	-1.27E-01	-1.37E-02	-1.39E-01	-1.84E-02	-1.52E-01	-2.84E-02	-1.74E-01	-3.61E-02
-4.89E-02	-8.90E-03	-1.15E-01	-9.84E-02	-1.67E-02	7.42E-02	6.99E-03	-1.71E-02	8.50E-02	8.36E-02	1.11E-01	-2.11E-02	1.89E-01	8.84E-02	2.30E-01	-6.00E-03	3.03E-01	9.72E-02	-1.36E-01	-2.32E-02	-1.44E-01	-1.74E-02	-1.54E-01	-1.86E-02	-1.73E-01	-2.23E-02
-2.14E-02	-1.35E-02	-1.02E-01	-9.47E-02	-2.05E-02	7.26E-02	1.14E-02	-4.10E-03	8.54E-02	8.86E-02	1.23E-01	-1.75E-02	1.95E-01	9.28E-02	2.39E-01	-5.93E-03	2.88E-01	1.05E-01	-1.22E-01	-2.39E-02	-1.36E-01	-2.54E-02	-1.49E-01	-3.23E-02	-1.72E-01	-3.49E-02
-2.61E-02	-1.02E-02	-1.02E-01	-9.40E-02	-2.41E-02	7.80E-02	9.08E-04	1.20E-03	7.84E-02	1.01E-01	1.20E-01	-1.96E-04	1.87E-01	9.66E-02	2.33E-01	3.23E-04	2.94E-01	9.56E-02	-1.24E-01	-3.51E-02	-1.38E-01	-3.18E-02	-1.52E-01	-3.58E-02	-1.76E-01	-3.75E-02
-2.12E-02	-1.83E-03	-1.00E-01	-9.97E-02	-2.47E-02	8.30E-02	9.91E-03	7.53E-03	7.06E-02	9.23E-02	1.20E-01	-5.36E-03	2.00E-01	9.64E-02	2.31E-01	-7.40E-03	2.90E-01	8.49E-02	-1.20E-01	-2.15E-02	-1.31E-01	-2.76E-02	-1.44E-01	-3.20E-02	-1.68E-01	-3.45E-02
-2.49E-02	-8.69E-03	-9.34E-02	-9.50E-02	-2.59E-02	6.64E-02	7.09E-03	2.80E-03	6.79E-02	7.63E-02	1.21E-01	-9.77E-03	1.96E-01	8.15E-02	2.36E-01	-3.77E-03	2.93E-01	8.44E-02	-1.22E-01	-9.02E-03	-1.35E-01	-1.63E-02	-1.48E-01	-3.04E-02	-1.72E-01	-3.90E-02
-2.12E-02	-1.16E-02	-9.95E-02	-8.86E-02	-2.12E-02	6.38E-02	8.25E-03	2.78E-03	6.23E-02	8.74E-02	1.12E-01	-1.01E-02	1.76E-01	8.44E-02	2.38E-01	-9.03E-03	2.83E-01	8.75E-02	-1.18E-01	-2.70E-02	-1.31E-01	-2.67E-02	-1.46E-01	-2.96E-02	-1.72E-01	-3.20E-02
-1.70E-02	-1.78E-02	-1.09E-01	-1.01E-01	-2.01E-02	8.21E-02	1.25E-02	-3.73E-03	8.43E-02	9.34E-02	1.19E-01	-1.01E-02	1.91E-01	9.26E-02	2.30E-01	4.50E-05	2.93E-01	1.07E-01	-1.16E-01	-6.21E-03	-1.33E-01	-1.06E-02	-1.50E-01	-2.85E-02	-1.77E-01	-4.15E-02
-1.88E-02	-1.12E-02	-9.92E-02	-9.51E-02	-2.51E-02	7.14E-02	1.17E-02	-7.52E-03	6.32E-02	8.59E-02	1.24E-01	-8.28E-03	1.91E-01	8.50E-02	2.64E-01	5.14E-03	2.90E-01	9.08E-02	-1.21E-01	-2.20E-02	-1.35E-01	-2.48E-02	-1.49E-01	-3.23E-02	-1.74E-01	-3.74E-02
-1.87E-02	-1.07E-02	-1.16E-01	-1.02E-01	-3.11E-02	7.62E-02	6.21E-03	1.25E-03	6.86E-02	9.67E-02	1.24E-01	-1.11E-02	1.93E-01	9.70E-02	2.29E-01	-7.29E-03	3.05E-01	9.84E-02	-1.29E-01	-3.46E-02	-1.39E-01	-2.94E-02	-1.51E-01	-2.92E-02	-1.71E-01	-3.08E-02
-2.36E-02	-1.35E-02	-9.93E-02	-9.33E-02	-3.72E-02	7.73E-02	-5.54E-04	8.78E-05	6.88E-02	9.65E-02	1.07E-01	-1.46E-02	1.84E-01	9.80E-02	2.33E-01	-1.03E-02	2.92E-01	9.75E-02	-1.17E-01	-3.35E-02	-1.31E-01	-3.70E-02	-1.47E-01	-4.41E-02	-1.73E-01	-4.49E-02
-2.46E-02	-1.78E-02	-1.07E-01	-8.61E-02	-2.73E-02	7.08E-02	4.97E-03	-7.01E-03	7.62E-02	8.92E-02	1.20E-01	-1.03E-02	2.04E-01	9.25E-02	2.38E-01	-4.98E-03	3.05E-01	9.57E-02	-1.25E-01	-2.88E-02	-1.38E-01	-2.59E-02	-1.50E-01	-2.94E-02	-1.72E-01	-3.13E-02
-4.49E-02	-5.83E-03	-1.01E-01	-7.84E-02	-4.07E-02	8.77E-02	-1.65E-02	2.77E-03	7.60E-02	1.04E-01	1.18E-01	-7.04E-03	1.93E-01	8.95E-02	2.41E-01	-8.37E-03	3.16E-01	8.69E-02	-1.32E-01	-3.77E-02	-1.33E-01	-3.94E-02	-1.32E-01	-4.24E-02	-1.44E-01	-4.22E-02
-2.65E-02	-1.48E-02	-1.00E-01	-9.25E-02	-1.60E-02	7.49E-02	1.26E-02	-4.77E-03	8.16E-02	8.86E-02	1.19E-01	-1.23E-02	1.85E-01	8.74E-02	2.36E-01	-3.13E-03	2.91E-01	9.90E-02	-1.26E-01	-1.36E-02	-1.40E-01	-1.95E-02	-1.54E-01	-3.11E-02	-1.76E-01	-3.58E-02
-1.88E-02	-1.33E-02	-1.00E-01	-1.02E-01	-1.49E-02	8.20E-02	6.72E-03	-2.70E-03	7.69E-02	9.23E-02	1.26E-01	-1.27E-02	1.86E-01	9.12E-02	2.34E-01	-1.14E-02	3.01E-01	9.54E-02	-1.11E-01	-1.00E-02	-1.25E-01	-1.46E-02	-1.43E-01	-2.65E-02	-1.71E-01	-3.69E-02
-3.30E-02	-1.63E-02	-1.01E-01	-1.01E-01	-2.20E-02	7.49E-02	5.93E-03	-1.01E-02	8.33E-02	8.77E-02	1.14E-01	-1.59E-02	1.95E-01	9.06E-02	2.32E-01	-8.24E-03	2.96E-01	9.84E-02	-1.23E-01	-2.85E-02	-1.34E-01	-2.40E-02	-1.46E-01	-2.40E-02	-1.69E-01	-2.29E-02
-1.86E-02	-1.50E-02	-1.03E-01	-1.05E-01	-1.47E-02	7.43E-02	3.03E-02	-5.97E-03	7.51E-02	8.47E-02	1.26E-01	-6.20E-03	1.84E-01	8.80E-02	2.35E-01	2.54E-04	2.85E-01	9.46E-02	-1.17E-01	-1.56E-02	-1.35E-01	-1.80E-02	-1.55E-01	-2.83E-02	-1.83E-01	-3.83E-02
-2.15E-02	-2.22E-02	-1.17E-01	-7.41E-02	-3.37E-02	7.31E-02	4.15E-03	-1.18E-02	7.46E-02	9.18E-02	1.22E-01	-1.31E-02	1.97E-01	9.64E-02	2.44E-01	-6.88E-04	3.06E-01	9.60E-02	-1.37E-01	-3.99E-02	-1.45E-01	-3.58E-02	-1.55E-01	-3.97E-02	-1.70E-01	-3.66E-02
-3.47E-02	-7.85E-03	-1.13E-01	-1.02E-01	-2.14E-02	8.35E-02	1.72E-02	-4.14E-03	6.98E-02	9.34E-02	1.24E-01	-1.25E-02	1.81E-01	9.59E-02	2.30E-01	-2.91E-03	2.92E-01	1.01E-01	-1.34E-01	-3.45E-02	-1.43E-01	-3.02E-02	-1.53E-01	-3.00E-02	-1.73E-01	-3.21E-02
-2.36E-02	-9.21E-03	-1.15E-01	-1.04E-01	-2.74E-02	7.21E-02	1.46E-03	1.57E-03	7.14E-02	9.19E-02	1.15E-01	-9.82E-03	1.85E-01	9.04E-02	2.37E-01	-4.23E-03	3.02E-01	9.91E-02	-1.20E-01	-2.90E-02	-1.35E-01	-2.88E-02	-1.54E-01	-3.46E-02	-1.79E-01	-3.75E-02
-1.80E-02	-2.00E-02	-1.05E-01	-9.21E-02	-2.60E-02	7.84E-02	7.37E-03	-5.89E-03	7.14E-02	9.38E-02	1.20E-01	-4.07E-03	1.92E-01	8.74E-02	2.35E-01	9.54E-04	3.03E-01	9.53E-02	-1.25E-01	-3.89E-02	-1.37E-01	-3.95E-02	-1.50E-01	-4.40E-02	-1.73E-01	-4.24E-02
-1.84E-02	-1.64E-03	-9.75E-02	-1.01E-01	-2.85E-02	7.17E-02	1.89E-02	7.05E-03	8.62E-02	9.50E-02	1.20E-01	2.64E-03	1.97E-01	8.98E-02	2.28E-01	6.00E-03	3.06E-01	8.68E-02	-1.16E-01	-7.52E-03	-1.32E-01	-1.24E-02	-1.46E-01	-3.05E-02	-1.70E-01	-4.26E-02
-2.04E-02	-1.19E-02	-1.03E-01	-8.95E-02	-3.03E-02	7.99E-02	4.51E-03	2.31E-04	7.30E-02	9.34E-02	1.23E-01	-1.18E-02	1.89E-01	8.80E-02	2.35E-01	-3.26E-03	2.93E-01	9.35E-02	-1.20E-01	-3.35E-02	-1.34E-01	-3.16E-02	-1.48E-01	-3.73E-02	-1.71E-01	-3.92E-02
-2.86E-02	-2.08E-02	-1.00E-01	-8.24E-02	-3.31E-02	6.32E-02	3.29E-03	-9.07E-03	6.09E-02	8.88E-02	1.25E-01	-1.38E-02	1.80E-01	8.97E-02	2.38E-01	-9.57E-03	2.89E-01	9.86E-02	-1.19E-01	-3.44E-02	-1.29E-01	-3.06E-02	-1.39E-01	-3.07E-02	-1.62E-01	-3.00E-02
-1.68E-02	-1.23E-02	-8.66E-02	-1.03E-01	-2.15E-02	5.69E-02	1.71E-02	7.02E-03	6.23E-02	9.18E-02	1.25E-01	-5.53E-03	1.83E-01	9.53E-02	2.34E-01	-7.77E-04	3.00E-01	1.01E-01	-1.15E-01	-2.19E-02	-1.27E-01	-2.53E-02	-1.42E-01	-2.97E-02	-1.70E-01	-3.45E-02
-1.31E-02	-1.51E-02	-1.14E-01	-9.94E-02	-4.14E-02	8.48E-02	1.43E-02	2.67E-03	6.93E-02	1.07E-01	1.20E-01	-7.60E-03	1.89E-01	1.01E-01	2.29E-01	-5.60E-03	3.04E-01	1.00E-01	-1.14E-01	-2.59E-02	-1.27E-01	-2.70E-02	-1.43E-01	-3.04E-02	-1.68E-01	-3.11E-02
-2.55E-02	-1.95E-02	-1.01E-01	-9.32E-02	-2.64E-02	6.67E-02	4.97E-03	-7.53E-03	6.92E-02	9.15E-02	1.06E-01	-5.95E-03	1.93E-01	9.34E-02	2.29E-01	1.03E-04	3.06E-01	1.02E-01	-1.22E-01	-3.07E-02	-1.34E-01	-3.01E-02	-1.48E-01	-3.18E-02	-1.74E-01	-3.15E-02
-2.23E-02	-2.06E-02	-9.09E-02	-9.62E-02	-3.10E-02	5.83E-02	3.47E-03	-5.42E-03	7.62E-02	8.52E-02	1.09E-01	-9.68E-03	1.86E-01	9.42E-02	2.37E-01	-2.46E-03	3.00E-01	9.83E-02	-1.15E-01	-2.15E-02	-1.28E-01	-2.29E-02	-1.40E-01	-3.06E-02	-1.64E-01	-3.04E-02
-2.58E-02	-1.44E-02	-8.63E-02	-9.53E-02	-3.64E-02	7.41E-02	7.51E-04	1.00E-03	7.25E-02	9.31E-02	1.08E-01	-8.05E-03	1.90E-01	9.77E-02	2.31E-01	-2.50E-03	2.98E-01	9.71E-02	-1.14E-01	-1.51E-02	-1.28E-01	-1.94E-02	-1.43E-01	-3.08E-02	-1.71E-01	-3.78E-02
-3.01E-02	-2.06E-02	-1.00E-01	-9.94E-02	-2.10E-02	5.67E-02	4.23E-03	-4.61E-03	6.97E-02	9.09E-02	1.16E-01	-9.72E-03	1.85E-01	9.86E-02	2.28E-01	4.77E-04	2.92E-01	1.03E-01	-1.27E-01	-2.61E-02	-1.37E-01	-2.41E-02	-1.48E-01	-2.57E-02	-1.71E-01	-2.69E-02
-3.21E-02	-1.64E-02	-9.36E-02	-9.41E-02	-2.04E-02	7.75E-02	2.13E-03	-8.19E-03	8.35E-02	9.78E-02	1.10E-01	-6.45E-03	1.99E-01	9.77E-02	2.24E-01	-1.60E-03	3.07E-01	9.59E-02	-1.18E-01	-2.94E-02	-1.30E-01	-2.76E-02	-1.44E-01	-2.89E-02	-1.71E-01	-3.37E-02
-3.39E-02	-1.37E-02	-1.03E-01	-8.51E-02	-8.08E-03	7.07E-02	1.34E-03	-1.11E-02	8.25E-02	8.76E-02	1.16E-01	-8.90E-03	1.90E-01	9.16E-02	2.31E-01	-3.18E-03	3.01E-01	9.23E-02	-1.25E-01	-1.61E-02	-1.38E-01	-1.95E-02	-1.52E-01	-2.85E-02	-1.77E-01	-2.94E-02
-2.87E-02	-1.32E-02	-1.01E-01	-9.35E-02	-3.10E-02	7.17E-02	-1.29E-03	-2.23E-03	6.78E-02	8.61E-02	1.16E-01	-1.03E-02	1.84E-01	8.92E-02	2.36E-01	-4.46E-03	2.96E-01	9.04E-02	-1.17E-01	-3.03E-02	-1.30E-01	-3.04E-02	-1.45E-01	-3.77E-02	-1.71E-01	-3.81E-02
-1.40E-02	-1.82E-02	-9.46E-02	-9.51E-02	-1.61E-02	7.72E-02	1.69E-02	-3.78E-03	8.69E-02	9.03E-02	1.15E-01	-8.82E-03	1.93E-01	9.06E-02	2.27E-01	-2.75E-03	3.03E-01	9.30E-02	-1.15E-01	-1.62E-02	-1.31E-01	-2.05E-02	-1.46E-01	-3.74E-02	-1.72E-01	-4.80E-02
-4.01E-02	-1.41E-02	-1.06E-01	-8.83E-02	-1.26E-02	7.02E-02	-4.10E-03	-9.23E-03	9.29E-02	8.84E-02	1.13E-01	-1.33E-02	2.02E-01	8.84E-02	2.49E-01	3.91E-03	3.11E-01	9.57E-02	-1.39E-01	-1.21E-02	-1.46E-01	-2.32E-02	-1.56E-01	-3.44E-02	-1.78E-01	-3.81E-02
-2.52E-02	-8.16E-03	-1.14E-01	-8.94E-02	-2.55E-02	6.77E-02	4.05E-03	4.17E-03	7.49E-02	8.93E-02	1.20E-01	-6.74E-03	1.97E-01	9.40E-02	2.36E-01	-2.17E-03	3.19E-01	9.98E-02	-1.25E-01	-2.23E-02	-1.39E-01	-2.37E-02	-1.56E-01	-2.92E-02	-1.81E-01	-3.14E-02
-2.27E-02	-9.59E-03	-1.07E-01	-8.97E-02	-1.93E-02	7.76E-02	3.76E-03	3.07E-04	7.86E-02	9.63E-02	1.16E-01	-1.07E-02	1.97E-01	9.26E-02	2.35E-01	-8.70E-03	3.13E-01	9.96E-02	-1.23E-01	-2.66E-02	-1.36E-01	-2.96E-02	-1.52E-01	-3.56E-02	-1.79E-01	-3.78E-02
-3.47E-02	-2.06E-02	-1.20E-01	-8.49E-02	-2.81E-02	8.45E-02	-6.20E-03	-9.50E-03	7.80E-02	9.71E-02	1.18E-01	-4.30E-03	2.01E-01	1.02E-01	2.40E-01	3.16E-03	3.10E-01	9.81E-02	-1.36E-01	-3.31E-02	-1.47E-01	-2.96E-02	-1.57E-01	-3.30E-02	-1.73E-01	-3.24E-02
-3.22E-02	-6.06E-03	-1.07E-01	-8.35E-02	-1.63E-02	7.30E-02	-5.99E-03	5.10E-03	9.20E-02	9.15E-02	1.16E-01	-6.49E-03	2.09E-01	9.07E-02	2.35E-01	-2.04E-03	3.21E-01	9.55E-02	-1.33E-01	-2.06E-02	-1.46E-01	-2.40E-02	-1.60E-01	-3.08E-02	-1.82E-01	-3.62E-02
-1.90E-02	-1.33E-02	-1.13E-01	-9.68E-02	-2.71E-02	8.42E-02	5.86E-03	-2.13E-03	7.88E-02	9.91E-02	1.21E-01	-8.60E-03	2.00E-01	1.02E-01	2.36E-01	1.25E-03	3.06E-01	1.01E-01	-1.30E-01	-2.47E-02	-1.42E-01	-2.46E-02	-1.55E-01	-2.71E-02	-1.75E-01	-3.14E-02
-2.48E-02	-9.98E-03	-1.01E-01	-9.33E-02	-2.26E-02	7.91E-02	4.99E-03	-2.20E-03	7.80E-02	9.42E-02	1.15E-01	-1.14E-02	1.92E-01	9.37E-02	2.35E-01	-2.30E-03	3.00E-01	9.80E-02	-1.26E-01	-1.92E-02	-1.38E-01	-2.11E-02	-1.51E-01	-2.63E-02	-1.72E-01	-3.01E-02
-4.29E-02	-9.74E-03	-1.17E-01	-8.33E-02	-1.82E-02	7.39E-02	8.54E-03	-3.35E-03	7.69E-02	8.03E-02	1.19E-01	-1.05E-02	1.95E-01	8.98E-02	2.46E-01	3.74E-03	3.17E-01	9.70E-02	-1.30E-01	-1.65E-02	-1.42E-01	-1.69E-02	-1.57E-01	-2.46E-02	-1.78E-01	-3.17E-02
-2.69E-02	-1.14E-02	-9.98E-02	-9.81E-02	-9.97E-03	9.08E-02	2.57E-03	-1.62E-03	8.67E-02	1.07E-01	1.20E-01	-5.96E-03	1.95E-01	1.06E-01	2.32E-01	-8.87E-03	3.00E-01	1.00E-01	-1.21E-01	-2.24E-02	-1.33E-01	-3.26E-02	-1.47E-01	-4.20E-02	-1.74E-01	-4.79E-02
-2.08E-02	-7.52E-03	-9.81E-02	-9.94E-02	-2.07E-02	8.43E-02	2.71E-02	-2.95E-03	8.33E-02	1.01E-01	1.25E-01	-9.80E-03	2.03E-01	9.41E-02	2.29E-01	-4.78E-03	3.01E-01	9.52E-02	-1.18E-01	-1.93E-02	-1.32E-01	-2.55E-02	-1.45E-01	-3.78E-02	-1.70E-01	-3.99E-02
-3.12E-02	-2.11E-02	-1.09E-01	-9.26E-02	-1.69E-02	7.84E-02	4.28E-03	-4.65E-03	7.30E-02	1.03E-01	1.21E-01	-7.96E-03	1.91E-01	1.05E-01	2.34E-01	-4.55E-03	3.07E-01	1.03E-01	-1.24E-01	-1.62E-02	-1.38E-01	-2.15E-02	-1.53E-01	-2.48E-02	-1.78E-01	-3.16E-02
-3.75E-02	-1.55E-02	-1.00E-01	-9.68E-02	-1.13E-02	8.47E-02	6.66E-03	-6.54E-03	7.85E-02	1.01E-01	1.15E-01	-9.13E-03	1.85E-01	1.05E-01	2.38E-01	-1.43E-02	2.93E-01	9.60E-02	-1.15E-01	-2.49E-02	-1.29E-01	-3.16E-02	-1.43E-01	-4.10E-02	-1.71E-01	-4.22E-02
-2.95E-02	-1.71E-02	-9.05E-02	-9.67E-02	-1.62E-02	8.29E-02	9.70E-03	-8.46E-03	7.37E-02	8.86E-02	1.24E-01	-1.16E-02	1.87E-01	9.56E-02	2.36E-01	-3.08E-03	2.91E-01	8.96E-02	-1.18E-01	-1.69E-02	-1.31E-01	-2.38E-02	-1.50E-01	-2.96E-02	-1.78E-01	-4.23E-02
-2.88E-02	-1.44E-02	-9.47E-02	-1.15E-01	-1.47E-02	8.32E-02	5.13E-03	-1.12E-02	8.42E-02	9.56E-02	1.22E-01	-1.20E-02	1.88E-01	9.77E-02	2.28E-01	-8.42E-03	2.90E-01	1.01E-01	-1.16E-01	-1.05E-02	-1.29E-01	-2.26E-02	-1.45E-01	-4.42E-02	-1.73E-01	-6.00E-02
-2.31E-02	-1.99E-02	-1.00E-01	-9.36E-02	-1.52E-02	8.08E-02	7.04E-03	-2.20E-03	7.77E-02	9.78E-02	1.20E-01	-5.47E-03	1.88E-01	9.86E-02	2.30E-01	2.31E-03	2.98E-01	9.80E-02	-1.23E-01	-2.46E-02	-1.39E-01	-2.62E-02	-1.52E-01	-3.64E-02	-1.76E-01	-3.62E-02
-1.64E-02	-2.48E-02	-8.00E-02	-1.02E-01	-1.25E-02	8.33E-02	9.05E-03	-1.52E-02	8.04E-02	9.58E-02	1.21E-01	-1.26E-02	1.90E-01	9.85E-02	2.38E-01	-6.27E-03	2.92E-01	9.75E-02	-1.12E-01	-5.59E-03	-1.27E-01	-1.59E-02	-1.41E-01	-4.29E-02	-1.66E-01	-6.42E-02
-5.89E-02	-8.51E-04	-8.89E-02	-9.93E-02	-1.17E-02	7.65E-02	-1.06E-02	1.13E-03	8.49E-02	9.25E-02	1.15E-01	-8.03E-03	2.05E-01	9.94E-02	2.36E-01	-6.50E-03	3.03E-01	9.66E-02	-1.29E-01	-3.06E-03	-1.42E-01	-1.54E-02	-1.51E-01	-3.61E-02	-1.75E-01	-4.33E-02
-2.88E-02	-1.54E-02	-1.01E-01	-1.02E-01	-2.87E-02	8.40E-02	2.72E-03	-8.95E-03	6.46E-02	8.99E-02	1.20E-01	-4.29E-03	1.82E-01	9.90E-02	2.41E-01	-4.05E-03	3.01E-01	9.93E-02	-1.21E-01	-2.12E-02	-1.33E-01	-1.90E-02	-1.48E-01	-1.87E-02	-1.72E-01	-2.13E-02
-2.64E-02	-5.42E-03	-1.13E-01	-8.52E-02	-3.06E-02	5.95E-02	1.83E-03	-7.42E-05	7.39E-02	8.32E-02	1.31E-01	-1.32E-02	1.87E-01	8.83E-02	2.46E-01	-1.20E-02	2.91E-01	1.05E-01	-1.26E-01	-1.13E-02	-1.39E-01	-1.87E-02	-1.55E-01	-2.71E-02	-1.80E-01	-3.13E-02

-3.13E-02	-1.07E-02	-1.03E-01	-8.28E-02	-3.08E-02	7.32E-02	6.91E-03	-2.01E-03	6.97E-02	9.17E-02	1.24E-01	-1.40E-02	1.99E-01	9.61E-02	2.38E-01	-3.23E-03	3.01E-01	8.55E-02	-1.27E-01	-2.16E-02	-1.39E-01	-2.33E-02	-1.51E-01	-2.70E-02	-1.73E-01	-2.81E-02
-2.40E-02	-1.05E-02	-1.10E-01	-9.87E-02	-2.77E-02	7.40E-02	8.29E-03	3.87E-03	7.76E-02	1.06E-01	1.22E-01	-6.10E-03	1.89E-01	9.74E-02	2.41E-01	-5.82E-04	2.90E-01	1.14E-01	-1.21E-01	-2.84E-02	-1.34E-01	-2.86E-02	-1.49E-01	-2.93E-02	-1.74E-01	-3.04E-02
-5.48E-02	-9.73E-03	-9.54E-02	-1.02E-01	-1.04E-02	7.55E-02	-4.52E-03	-4.07E-03	6.65E-02	8.53E-02	1.21E-01	-7.17E-03	1.96E-01	8.89E-02	2.46E-01	-1.42E-02	3.00E-01	9.09E-02	-1.27E-01	-1.29E-02	-1.38E-01	-1.90E-02	-1.50E-01	-3.13E-02	-1.73E-01	-3.66E-02
-3.51E-02	-1.48E-02	-9.84E-02	-9.91E-02	-1.82E-02	7.80E-02	2.07E-03	-7.92E-03	8.05E-02	9.58E-02	1.14E-01	-7.87E-03	1.90E-01	9.71E-02	2.36E-01	-1.03E-02	2.90E-01	9.45E-02	-1.23E-01	-1.33E-02	-1.34E-01	-2.06E-02	-1.48E-01	-3.37E-02	-1.74E-01	-4.04E-02
-2.99E-02	2.24E-04	-9.23E-02	-8.53E-02	-2.40E-02	7.31E-02	1.38E-02	-3.99E-04	8.34E-02	8.82E-02	1.25E-01	-8.51E-03	1.94E-01	8.45E-02	2.34E-01	-6.46E-03	2.96E-01	9.95E-02	-1.20E-01	-1.74E-02	-1.31E-01	-2.29E-02	-1.43E-01	-3.18E-02	-1.70E-01	-3.60E-02
-2.68E-02	-1.31E-02	-9.84E-02	-8.93E-02	-1.63E-02	9.02E-02	1.15E-02	9.92E-04	7.50E-02	1.00E-01	1.19E-01	-5.85E-03	1.90E-01	1.01E-01	2.40E-01	-2.98E-03	3.01E-01	9.64E-02	-1.09E-01	-2.08E-02	-1.23E-01	-2.79E-02	-1.43E-01	-3.54E-02	-1.77E-01	-4.12E-02
-6.07E-02	5.10E-03	-1.02E-01	-8.95E-02	-1.52E-02	8.02E-02	7.41E-03	1.84E-04	8.31E-02	1.00E-01	1.24E-01	-9.81E-03	1.97E-01	9.81E-02	2.40E-01	1.13E-03	3.02E-01	9.20E-02	-1.33E-01	-1.35E-02	-1.41E-01	-2.58E-02	-1.52E-01	-3.92E-02	-1.75E-01	-4.78E-02
-2.92E-02	-1.85E-02	-1.07E-01	-9.47E-02	-2.22E-02	8.66E-02	-3.14E-03	-9.30E-03	6.88E-02	1.01E-01	1.25E-01	-3.48E-03	1.79E-01	1.02E-01	2.40E-01	-7.84E-03	2.95E-01	1.03E-01	-1.17E-01	-8.67E-03	-1.32E-01	-1.67E-02	-1.46E-01	-3.62E-02	-1.72E-01	-4.31E-02
-3.81E-02	-1.88E-02	-1.13E-01	-9.48E-02	-2.35E-02	7.97E-02	2.05E-03	-1.50E-02	7.28E-02	9.71E-02	1.28E-01	-1.54E-02	1.99E-01	1.02E-01	2.43E-01	-2.84E-03	2.97E-01	1.03E-01	-1.23E-01	-2.52E-02	-1.35E-01	-2.69E-02	-1.47E-01	-2.96E-02	-1.71E-01	-2.95E-02
-3.46E-02	-2.35E-02	-9.93E-02	-9.53E-02	-9.88E-03	7.19E-02	3.03E-03	-8.45E-03	8.16E-02	9.29E-02	1.23E-01	-1.22E-02	1.94E-01	9.91E-02	2.35E-01	-2.48E-03	2.92E-01	1.08E-01	-1.18E-01	-2.01E-02	-1.32E-01	-2.27E-02	-1.50E-01	-3.11E-02	-1.81E-01	-3.83E-02
-1.72E-02	-1.66E-02	-9.80E-02	-1.05E-01	-1.60E-02	8.21E-02	1.85E-03	-8.12E-04	7.67E-02	1.00E-01	1.16E-01	-7.09E-03	1.84E-01	9.79E-02	2.37E-01	-6.20E-03	2.95E-01	1.10E-01	-1.11E-01	-2.92E-02	-1.27E-01	-3.83E-02	-1.48E-01	-4.79E-02	-1.82E-01	-5.84E-02
-2.04E-02	-1.37E-02	-1.00E-01	-8.74E-02	-1.06E-02	7.24E-02	7.88E-03	-8.42E-03	7.96E-02	9.08E-02	1.25E-01	-1.03E-02	1.89E-01	9.71E-02	2.42E-01	-8.06E-03	2.90E-01	1.05E-01	-1.16E-01	-1.60E-02	-1.31E-01	-2.25E-02	-1.49E-01	-3.33E-02	-1.81E-01	-3.88E-02
-2.94E-02	-1.35E-02	-1.00E-01	-8.88E-02	-1.78E-02	8.13E-02	3.50E-03	-5.55E-03	8.23E-02	1.01E-01	1.17E-01	-1.06E-02	2.00E-01	1.02E-01	2.33E-01	-6.38E-03	2.96E-01	9.52E-02	-1.22E-01	-2.55E-02	-1.37E-01	-2.80E-02	-1.53E-01	-3.27E-02	-1.78E-01	-3.86E-02
-2.46E-02	-7.68E-03	-9.62E-02	-8.59E-02	-2.98E-02	7.52E-02	5.51E-03	-2.64E-03	7.46E-02	9.14E-02	1.27E-01	-8.20E-03	1.89E-01	9.02E-02	2.43E-01	-1.08E-02	2.99E-01	9.21E-02	-1.26E-01	-8.27E-03	-1.35E-01	-2.05E-02	-1.49E-01	-3.50E-02	-1.75E-01	-4.71E-02
-3.87E-02	-1.60E-02	-1.07E-01	-9.12E-02	-2.32E-02	7.83E-02	-3.97E-03	-7.77E-03	7.81E-02	1.06E-01	1.15E-01	-1.19E-02	2.03E-01	1.00E-01	2.35E-01	-1.30E-02	3.08E-01	9.96E-02	-1.27E-01	-2.01E-02	-1.40E-01	-2.35E-02	-1.55E-01	-3.38E-02	-1.78E-01	-4.33E-02
-2.74E-02	-2.37E-02	-9.08E-02	-9.05E-02	-1.63E-02	8.09E-02	1.23E-02	-8.87E-03	7.39E-02	8.88E-02	1.14E-01	-1.11E-02	1.86E-01	9.58E-02	2.33E-01	2.40E-03	2.99E-01	1.00E-01	-1.22E-01	-1.23E-02	-1.35E-01	-2.11E-02	-1.52E-01	-3.47E-02	-1.82E-01	-4.66E-02
-2.94E-02	-1.51E-02	-1.10E-01	-9.82E-02	-1.99E-02	7.46E-02	4.55E-03	5.54E-03	7.30E-02	1.01E-01	1.19E-01	-1.07E-02	1.88E-01	1.07E-01	2.34E-01	-5.66E-03	3.02E-01	1.02E-01	-1.30E-01	-3.63E-03	-1.40E-01	-1.44E-02	-1.55E-01	-2.55E-02	-1.80E-01	-3.30E-02
-1.30E-02	-1.20E-02	-1.01E-01	-9.19E-02	-3.09E-02	6.18E-02	1.30E-02	5.23E-03	7.80E-02	9.10E-02	1.09E-01	-7.26E-03	1.92E-01	9.71E-02	2.46E-01	-5.34E-03	2.98E-01	9.71E-02	-1.22E-01	-1.51E-02	-1.34E-01	-2.06E-02	-1.48E-01	-2.90E-02	-1.74E-01	-3.15E-02
-4.23E-02	-1.16E-02	-1.01E-01	-8.85E-02	-1.11E-02	8.30E-02	2.93E-03	-9.01E-03	8.53E-02	9.83E-02	1.24E-01	-1.13E-02	1.96E-01	1.06E-01	2.36E-01	-1.35E-02	3.00E-01	1.01E-01	-1.27E-01	-1.10E-02	-1.39E-01	-1.89E-02	-1.53E-01	-3.17E-02	-1.78E-01	-4.10E-02
-3.72E-02	-1.00E-02	-8.38E-02	-1.13E-01	-2.83E-02	8.12E-02	1.20E-02	-2.79E-03	7.27E-02	9.17E-02	1.20E-01	-1.04E-02	1.89E-01	9.31E-02	2.31E-01	-1.45E-02	2.95E-01	9.78E-02	-1.18E-01	-4.32E-03	-1.27E-01	-1.58E-02	-1.40E-01	-3.51E-02	-1.66E-01	-4.80E-02
-1.45E-02	-9.59E-03	-1.02E-01	-8.88E-02	-2.07E-02	7.24E-02	1.29E-02	-1.41E-03	6.83E-02	8.28E-02	1.26E-01	-6.14E-03	1.85E-01	8.31E-02	2.34E-01	-7.61E-04	2.99E-01	8.35E-02	-1.26E-01	-1.29E-02	-1.39E-01	-2.06E-02	-1.53E-01	-3.05E-02	-1.77E-01	-3.33E-02
-3.47E-02	-1.52E-02	-9.66E-02	-8.71E-02	-2.18E-02	7.16E-02	-3.26E-03	-6.01E-03	7.33E-02	9.04E-02	1.23E-01	-9.23E-03	1.92E-01	9.15E-02	2.43E-01	-3.12E-03	3.06E-01	9.90E-02	-1.24E-01	-1.41E-02	-1.39E-01	-1.64E-02	-1.53E-01	-2.53E-02	-1.77E-01	-3.25E-02
-4.47E-02	-1.11E-02	-1.01E-01	-9.49E-02	-2.86E-02	8.03E-02	-1.19E-03	-1.08E-02	7.49E-02	9.23E-02	1.17E-01	-1.03E-02	1.82E-01	9.54E-02	2.32E-01	-1.28E-02	2.92E-01	9.68E-02	-1.20E-01	-1.73E-02	-1.33E-01	-1.79E-02	-1.46E-01	-3.02E-02	-1.68E-01	-3.58E-02
-3.74E-02	-1.08E-03	-9.80E-02	-9.20E-02	-2.18E-02	7.47E-02	7.15E-03	-1.76E-03	7.44E-02	8.31E-02	1.22E-01	-7.61E-03	1.80E-01	8.33E-02	2.34E-01	-5.06E-03	2.98E-01	9.45E-02	-1.28E-01	-2.30E-02	-1.42E-01	-2.40E-02	-1.54E-01	-3.28E-02	-1.77E-01	-3.82E-02
-6.09E-02	-5.85E-03	-1.08E-01	-9.39E-02	-2.79E-03	7.50E-02	-1.15E-02	-4.12E-03	8.23E-02	9.05E-02	1.17E-01	-1.33E-02	1.91E-01	9.13E-02	2.39E-01	-1.44E-03	2.98E-01	9.01E-02	-1.34E-01	-2.57E-02	-1.46E-01	-3.42E-02	-1.61E-01	-4.77E-02	-1.86E-01	-5.11E-02
-3.03E-02	-1.45E-02	-1.04E-01	-9.57E-02	-1.42E-02	7.54E-02	5.77E-04	-8.49E-03	8.42E-02	9.06E-02	1.18E-01	-1.19E-02	1.88E-01	9.05E-02	2.37E-01	-5.66E-03	2.84E-01	9.41E-02	-1.30E-01	-4.13E-02	-1.44E-01	-4.30E-02	-1.59E-01	-4.78E-02	-1.86E-01	-4.41E-02
-3.44E-02	-1.26E-02	-1.05E-01	-9.56E-02	-3.00E-02	8.30E-02	3.84E-03	-7.26E-03	6.29E-02	9.82E-02	1.22E-01	-1.72E-02	1.81E-01	9.55E-02	2.39E-01	-1.05E-02	2.98E-01	9.91E-02	-1.31E-01	-1.75E-02	-1.41E-01	-2.27E-02	-1.50E-01	-3.35E-02	-1.67E-01	-3.75E-02
-1.60E-02	-2.50E-02	-9.96E-02	-1.12E-01	-2.21E-02	6.80E-02	1.66E-02	-8.12E-03	6.91E-02	9.70E-02	1.12E-01	-6.25E-03	1.85E-01	1.03E-01	2.31E-01	1.97E-03	2.98E-01	1.20E-01	-1.11E-01	-1.30E-02	-1.21E-01	-2.32E-02	-1.36E-01	-3.40E-02	-1.66E-01	-3.71E-02
-2.21E-02	-9.93E-03	-9.96E-02	-9.37E-02	-2.19E-02	8.13E-02	1.25E-02	1.54E-03	7.29E-02	9.77E-02	1.20E-01	-9.98E-03	1.85E-01	8.97E-02	2.34E-01	-1.02E-02	3.01E-01	1.02E-01	-1.19E-01	-2.21E-02	-1.31E-01	-3.00E-02	-1.45E-01	-3.73E-02	-1.71E-01	-3.87E-02
-1.85E-02	-1.16E-02	-8.86E-02	-7.98E-02	-2.50E-02	8.70E-02	7.90E-03	-5.07E-03	7.91E-02	1.01E-01	1.18E-01	-1.14E-02	1.90E-01	9.33E-02	2.41E-01	-7.74E-03	2.93E-01	8.82E-02	-1.17E-01	-3.17E-02	-1.31E-01	-3.58E-02	-1.44E-01	-4.61E-02	-1.68E-01	-4.82E-02
-1.49E-02	-1.54E-02	-9.82E-02	-9.92E-02	-1.02E-02	7.84E-02	1.40E-02	4.43E-04	7.73E-02	8.78E-02	1.27E-01	-6.79E-03	1.90E-01	9.71E-02	2.36E-01	-3.97E-03	2.95E-01	9.86E-02	-1.19E-01	-1.27E-02	-1.34E-01	-2.20E-02	-1.46E-01	-4.15E-02	-1.72E-01	-4.73E-02
-2.40E-02	-1.99E-02	-1.02E-01	-1.04E-01	-1.45E-02	8.60E-02	6.23E-03	-4.35E-03	7.17E-02	9.30E-02	1.22E-01	-7.93E-03	1.90E-01	9.96E-02	2.37E-01	6.61E-03	2.94E-01	1.05E-01	-1.19E-01	-2.83E-02	-1.34E-01	-3.09E-02	-1.50E-01	-3.90E-02	-1.77E-01	-4.44E-02
-2.45E-02	-1.92E-02	-9.71E-02	-9.38E-02	-2.82E-02	8.00E-02	-7.13E-04	-1.17E-03	6.80E-02	9.09E-02	1.17E-01	-1.40E-02	1.82E-01	8.62E-02	2.32E-01	-3.38E-03	2.97E-01	8.87E-02	-1.17E-01	-2.81E-02	-1.27E-01	-3.81E-02	-1.42E-01	-4.71E-02	-1.71E-01	-5.00E-02
-2.27E-02	-1.96E-02	-9.82E-02	-9.54E-02	-3.11E-02	7.66E-02	4.25E-03	-8.24E-03	6.56E-02	9.12E-02	1.23E-01	-9.40E-03	1.87E-01	9.44E-02	2.35E-01	-6.94E-03	2.95E-01	1.02E-01	-1.31E-01	-2.17E-02	-1.41E-01	-2.43E-02	-1.51E-01	-3.17E-02	-1.72E-01	-3.50E-02
-3.64E-02	-1.90E-02	-1.02E-01	-1.01E-01	-2.37E-02	7.46E-02	-1.90E-03	-7.67E-03	6.67E-02	9.33E-02	1.22E-01	-4.03E-03	1.85E-01	9.49E-02	2.34E-01	5.89E-04	2.96E-01	1.02E-01	-1.22E-01	-2.55E-02	-1.33E-01	-3.35E-02	-1.50E-01	-4.24E-02	-1.78E-01	-5.05E-02
-3.80E-02	-1.16E-02	-1.01E-01	-9.12E-02	-2.41E-02	7.81E-02	1.08E-02	-9.89E-03	7.11E-02	8.88E-02	1.22E-01	-2.20E-02	1.82E-01	8.94E-02	2.36E-01	-1.17E-02	2.95E-01	9.33E-02	-1.22E-01	-1.55E-02	-1.35E-01	-1.72E-02	-1.50E-01	-2.49E-02	-1.74E-01	-2.90E-02
-3.29E-02	-2.07E-02	-9.45E-02	-9.30E-02	-1.71E-02	6.67E-02	5.47E-04	-8.12E-03	7.49E-02	8.87E-02	1.19E-01	-1.33E-02	1.91E-01	9.22E-02	2.29E-01	-8.07E-03	2.97E-01	9.53E-02	-1.24E-01	-2.05E-02	-1.36E-01	-2.77E-02	-1.50E-01	-3.96E-02	-1.76E-01	-4.57E-02
-2.91E-02	-9.51E-03	-9.79E-02	-8.73E-02	-1.39E-02	7.50E-02	5.15E-03	6.67E-03	7.71E-02	9.17E-02	1.13E-01	-1.18E-02	1.95E-01	9.37E-02	2.26E-01	-8.70E-03	3.02E-01	8.66E-02	-1.27E-01	-1.99E-02	-1.39E-01	-2.50E-02	-1.51E-01	-3.15E-02	-1.73E-01	-3.37E-02
-4.48E-02	-9.34E-03	-9.82E-02	-8.68E-02	-3.43E-02	7.54E-02	-4.14E-03	-5.65E-03	6.57E-02	9.20E-02	1.19E-01	-1.94E-02	1.87E-01	9.01E-02	2.54E-01	-1.01E-02	2.97E-01	9.10E-02	-1.28E-01	-2.76E-02	-1.37E-01	-2.43E-02	-1.46E-01	-2.68E-02	-1.65E-01	-2.79E-02
-2.38E-02	-8.56E-03	-9.67E-02	-9.37E-02	-1.82E-02	8.11E-02	4.67E-03	-3.06E-03	7.79E-02	9.80E-02	1.24E-01	-9.54E-03	1.93E-01	9.45E-02	2.33E-01	-6.94E-03	2.94E-01	1.05E-01	-1.18E-01	-2.12E-02	-1.32E-01	-2.77E-02	-1.48E-01	-3.45E-02	-1.77E-01	-3.90E-02
-5.79E-02	-7.85E-03	-9.84E-02	-8.26E-02	-3.02E-02	7.41E-02	-2.63E-03	-1.71E-02	7.03E-02	8.42E-02	1.17E-01	-2.29E-02	1.96E-01	8.58E-02	2.38E-01	-2.06E-02	2.99E-01	9.40E-02	-1.27E-01	-2.07E-02	-1.36E-01	-2.14E-02	-1.46E-01	-2.80E-02	-1.66E-01	-3.01E-02
-3.64E-02	-1.38E-02	-9.33E-02	-9.75E-02	-2.72E-02	7.50E-02	1.59E-03	6.19E-05	7.29E-02	9.54E-02	1.15E-01	-3.58E-03	1.81E-01	9.90E-02	2.38E-01	-1.28E-03	2.94E-01	1.06E-01	-1.23E-01	-2.16E-02	-1.36E-01	-2.85E-02	-1.50E-01	-4.22E-02	-1.76E-01	-5.05E-02
-1.80E-02	-1.77E-02	-1.08E-01	-8.72E-02	-2.83E-02	7.11E-02	1.30E-02	-8.88E-03	6.75E-02	8.62E-02	1.21E-01	-1.38E-02	1.89E-01	9.18E-02	2.36E-01	1.77E-03	2.98E-01	9.96E-02	-1.20E-01	-1.86E-02	-1.35E-01	-2.30E-02	-1.51E-01	-3.24E-02	-1.76E-01	-3.68E-02
-4.58E-02	-8.55E-03	-1.04E-01	-9.14E-02	-9.12E-03	7.46E-02	-6.92E-03	-1.01E-02	8.12E-02	8.49E-02	1.21E-01	-9.37E-03	1.90E-01	8.69E-02	2.38E-01	-4.03E-03	3.03E-01	8.81E-02	-1.24E-01	-2.54E-02	-1.38E-01	-2.94E-02	-1.51E-01	-3.65E-02	-1.76E-01	-3.59E-02
-2.70E-02	-1.14E-02	-1.14E-01	-9.15E-02	-1.51E-02	6.69E-02	6.84E-03	-2.72E-03	7.83E-02	8.71E-02	1.16E-01	-1.26E-02	1.88E-01	9.12E-02	2.39E-01	-2.19E-03	2.93E-01	9.40E-02	-1.26E-01	-1.59E-02	-1.39E-01	-1.96E-02	-1.56E-01	-2.96E-02	-1.80E-01	-3.77E-02
-9.81E-03	-7.52E-03	-9.40E-02	-1.01E-01	-2.26E-02	8.64E-02	2.05E-02	1.98E-03	7.46E-02	9.59E-02	1.21E-01	-3.13E-03	1.83E-01	9.73E-02	2.40E-01	-6.33E-03	2.91E-01	9.43E-02	-1.19E-01	-3.01E-02	-1.32E-01	-3.42E-02	-1.46E-01	-4.23E-02	-1.74E-01	-4.59E-02
-2.21E-02	-2.20E-02	-1.10E-01	-8.90E-02	-4.25E-02	7.58E-02	7.96E-03	-5.53E-03	5.54E-02	9.57E-02	1.17E-01	-1.55E-02	1.82E-01	9.79E-02	2.38E-01	-6.57E-03	2.91E-01	1.05E-01	-1.23E-01	-2.29E-02	-1.37E-01	-2.51E-02	-1.52E-01	-3.59E-02	-1.74E-01	-4.00E-02
-5.02E-02	-1.65E-02	-9.67E-02	-9.73E-02	-2.93E-02	8.98E-02	-2.99E-03	-1.41E-02	7.00E-02	1.03E-01	1.23E-01	-1.26E-02	1.91E-01	9.76E-02	2.33E-01	-5.43E-03	3.03E-01	9.74E-02	-1.29E-01	-2.23E-02	-1.41E-01	-2.36E-02	-1.51E-01	-3.46E-02	-1.69E-01	-4.26E-02
-2.09E-02	-1.45E-02	-1.03E-01	-8.59E-02	-1.15E-02	8.09E-02	8.03E-03	-4.83E-03	8.29E-02	9.33E-02	1.26E-01	-3.21E-03	1.96E-01	9.05E-02	2.37E-01	-2.75E-03	2.97E-01	8.40E-02	-1.24E-01	-1.03E-02	-1.40E-01	-1.82E-02	-1.55E-01	-3.23E-02	-1.81E-01	-4.14E-02
-3.26E-02	-1.16E-02	-1.05E-01	-9.19E-02	-2.48E-02	7.22E-02	4.89E-04	-4.13E-03	6.45E-02	8.70E-02	1.24E-01	-5.98E-03	1.87E-01	9.35E-02	2.39E-01	-8.78E-05	3.01E-01	9.77E-02	-1.26E-01	-1.62E-02	-1.40E-01	-2.04E-02	-1.55E-01	-2.55E-02	-1.78E-01	-3.07E-02
-1.39E-02	-1.11E-02	-1.02E-01	-8.93E-02	-1.02E-02	8.48E-02	1.74E-02	9.19E-04	8.15E-02	9.80E-02	1.23E-01	-1.89E-03	1.92E-01	9.56E-02	2.37E-01	9.00E-04	2.99E-01	9.57E-02	-1.16E-01	-2.26E-02	-1.31E-01	-2.95E-02	-1.46E-01	-3.93E-02	-1.73E-01	-3.98E-02
-3.96E-02	-2.06E-02	-9.90E-02	-8.79E-02	-3.28E-02	7.96E-02	2.57E-03	-5.78E-03	7.28E-02	1.05E-01	1.21E-01	-2.77E-03	1.92E-01	1.00E-01	2.40E-01	-1.26E-03	2.97E-01	9.44E-02	-1.27E-01	-2.92E-02	-1.41E-01	-3.47E-02	-1.55E-01	-4.68E-02	-1.79E-01	-5.28E-02
-2.88E-02	-1.08E-02	-8.91E-02	-9.55E-02	-2.31E-02	7.89E-02	9.51E-03	6.86E-05	7.33E-02	1.05E-01	1.25E-01	-3.82E-03	1.90E-01	9.83E-02	2.40E-01	-7.10E-03	2.93E-01	9.07E-02	-1.26E-01	-1.59E-02	-1.41E-01	-1.83E-02	-1.57E-01	-3.10E-02	-1.80E-01	-4.63E-02
-1.27E-02	-1.47E-02	-9.57E-02	-8.36E-02	-3.98E-02	6.86E-02	7.34E-03	-2.48E-04	6.83E-02	8.34E-02	1.22E-01	-1.22E-02	1.89E-01	8.17E-02	2.42E-01	-6.95E-03	3.02E-01	8.48E-02	-1.19E-01	-2.32E-02	-1.32E-01	-2.07E-02	-1.45E-01	-2.20E-02	-1.69E-01	-2.53E-02
-2.28E-02	-1.16E-02	-9.99E-02	-8.55E-02	-1.67E-02	7.78E-02	4.90E-03	2.95E-03	8.57E-02	8.75E-02	1.28E-01	-6.85E-04	1.98E-01	8.54E-02	2.42E-01	1.70E-03	3.06E-01	8.19E-02	-1.26E-01	-2.23E-02	-1.40E-01	-2.36E-02	-1.53E-01	-2.94E-02	-1.75E-01	-3.37E-02
-3.53E-02	-1.82E-02	-1.03E-01	-9.75E-02	-3.23E-02	8.97E-02	1.02E-03	-1.06E-02	6.48E-02	1.04E-01	1.22E-01	-1.52E-02	1.78E-01	1.07E-01	2.38E-01	-1.13E-02	2.96E-01	1.06E-01	-1.28E-01	-3.13E-02	-1.39E-01	-2.89E-02	-1.50E-01	-3.28E-02	-1.70E-01	-3.68E-02
-2.43E-02	-2.13E-02	-1.06E-01	-8.84E-02	-1.48E-02	7.26E-02	9.86E-03	-9.18E-03	8.19E-02	8.82E-02	1.27E-01	-9.69E-03	1.95E-01	9.00E-02	2.44E-01	-2.16E-03	2.96E-01	9.50E-02	-1.31E-01	-1.38E-02	-1.46E-01	-2.09E-02	-1.59E-01	-3.35E-02	-1.82E-01	-3.63E-02
-2.58E-02	-1.15E-02	-9.55E-02	-9.20E-02	-1.98E-02	8.79E-02	1.80E-02	-1.95E-03	8.24E-02	9.97E-02	1.27E-01	-9.80E-03	1.94E-01	9.44E-02	2.37E-01	-4.01E-03	2.89E-01	9.65E-02	-1.26E-01	-1.37E-02	-1.37E-01	-2.54E-02	-1.48E-01	-4.58E-02	-1.70E-01	-5.30E-02
-1.98E-02	-2.11E-02	-9.95E-02	-9.12E-02	-3.01E-02	7.45E-02	7.33E-03	-6.39E-03	6.87E-02	9.17E-02	1.23E-01	-7.58E-03	1.87E-01	9.42E-02	2.41E-01	1.08E-04	3.04E-01	9.27E-02	-1.20E-01	-1.53E-02	-1.32E-01	-2.15E-02	-1.47E-01	-3.62E-02	-1.73E-01	-4.49E-02
-1.62E-02	-1.08E-02	-9.84E-02	-8.31E-02	-2.03E-02	7.63E-02	9.34E-03	-8.14E-04	9.10E-02	8.50E-02	1.20E-01	-8.67E-03	1.90E-01	8.45E-02	2.37E-01	-2.33E-03	2.85E-01	8.28E-02	-1.28E-01	-2.67E-02	-1.42E-01	-2.97E-02	-1.54E-01	-3.85E-02	-1.76E-01	-4.25E-02
-3.10E-02	-2.44E-02	-1.06E-01	-8.82E-02	-2.36E-02	6.60E-02	3.82E-03	-1.19E-02	6.58E-02	9.51E-02	1.26E-01	-1.49E-02	1.81E-01	9.75E-02	2.39E-01	-5.19E-03	2.89E-01	9.70E-02	-1.14E-01	-2.78E-02	-1.29E-01	-2.92E-02	-1.47E-01	-3.25E-02	-1.77E-01	-3.38E-02
-3.46E-02	-1.37E-02	-1.10E-01	-8.41E-02	-2.57E-02	7.34E-02	2.56E-03	-5.36E-03	7.04E-02	8.57E-02	1.22E-01	-1.12E-02	1.85E-01	9.03E-02	2.34E-01	-2.64E-03	2.98E-01	9.59E-02	-1.32E-01	-2.20E-02	-1.44E-01	-2.43E-02	-1.55E-01	-2.93E-02	-1.74E-01	-3.33E-02
-2.21E-02	-2.24E-02	-1.08E-01	-8.82E-02	-2.53E-02	7.07E-02	3.20E-03	-6.51E-03	7.33E-02	9.53E-02	1.23E-01	-1.04E-02	1.91E-01	9.23E-02	2.58E-01	1.34E-02	2.99E-01	1.00E-01	-1.26E-01	-2.38E-02	-1.39E-01	-2.34E-02	-1.54E-01	-2.84E-02	-1.78E-01	-3.20E-02
-3.66E-02	-1.15E-02	-1.04E-01	-8.11E-02	-3.80E-02	7.11E-02	7.29E-03	-2.07E-03	7.04E-02	9.08E-02	1.27E-01	-8.48E-03	1.95E-01	9.25E-02	2.40E-01	-1.86E-03	3.04E-01	9.71E-02	-1.28E-01	-2.22E-02	-1.41E-01	-2.60E-02	-1.53E-01	-3.04E-02	-1.74E-01	-3.27E-02
-1.87E-02	-1.79E-02	-1.13E-01	-8.69E-02	-3.14E-02	7.49E-02	7.05E-03	-2.32E-03	7.49E-02	9.06E-02	1.34E-01	-3.73E-03	1.94E-01	9.10E-02	2.45E-01	9.93E-04	3.01E-01	8.76E-02	-1.28E-01	-1.56E-02	-1.42E-01	-1.85E-02	-1.57E-01	-2.52E-02	-1.80E-01	-2.83E-02
-2.91E-02	-1.63E-02	-9.99E-02	-9.90E-02	-1.56E-02	8.37E-02	-1.64E-03	-5.95E-03	7.18E-02	1.00E-01	1.16E-01	-6.64E-03	1.85E-01	9.53E-02	2.49E-01	2.35E-03	2.98E-01	9.62E-02	-1.18E-01	-3.10E-02	-1.33E-01	-3.81E-02	-1.50E-01	-4.70E-02	-1.80E-01	-4.96E-02
-2.03E-02	-2.31E-02	-1.01E-01	-9.10E-02	-3.02E-02	8.12E-02	1.89E-02	-5.43E-03	6.29E-02	9.61E-02	1.28E-01	-4.13E-03	1.80E-01	9.89E-02	2.39E-01	3.02E-03	2.96E-01	1.05E-01	-1.20E-01	-1.81E-02	-1.32E-01	-2.28E-02	-1.48E-01	-2.83E-02	-1.73E-01	-3.66E-02
-1.65E-02	-1.77E-02	-1.13E-01	-8.54E-02	-1.44E-02	8.26E-02	9.50E-03	-5.47E-03	6.90E-02	1.04E-01	1.22E-01	-8.26E-03	1.84E-01	1.05E-01	2.38E-01	1.66E-03	2.87E-01	1.13E-01	-1.25E-01	-2.83E-02	-1.40E-01	-2.95E-02	-1.56E-01	-3.53E-02	-1.81E-01	-3.88E-02
-2.89E-02	-1.89E-02	-9.81E-02	-8.47E-02	-1.76E-02	8.07E-02	-1.95E-03	-9.82E-03	6.62E-02	8.82E-02	1.27E-01	-8.78E-03	1.85E-01	8.67E-02	2.43E-01	-8.44E-03	2.99E-01	8.75E-02	-1.19E-01	-2.74E-02	-1.31E-01	-3.24E-02	-1.43E-01	-3.89E-02	-1.68E-01	-4.10E-02
-3.97E-02	-1.80E-02	-1.09E-01	-8.72E-02	-1.35E-02	7.65E-02	1.33E-03	-9.68E-03	8.02E-02	9.20E-02	1.23E-01	-7.44E-03	1.91E-01	9.04E-02	2.37E-01	6.32E-04	3.12E-01	8.86E-02	-1.24E-01	-2.21E-02	-1.36E-01	-2.41E-02	-1.51E-01	-3.10E-02	-1.76E-01	-3.38E-02
-3.14E-02	-7.18E-03	-9.18E-02	-9.96E-02	-1.01E-02	8.76E-02	-4.10E-03	-2.60E-03	8.35E-02	9.77E-02	1.16E-01	-5.25E-03	1.95E-01	9.75E-02	2.30E-01	-5.52E-03	3.11E-01	1.00E-01	-1.18E-01	-2.61E-02	-1.32E-01	-3.24E-02	-1.48E-01	-3.90E-02	-1.75E-01	-4.65E-02
-2.83E-02	-1.59E-02	-8.53E-02	-9.75E-02	-1.93E-02	7.44E-02	1.05E-03	-5.94E-03	7.16E-02	8.72E-02	1.19E-01	-1.25E-02	1.89E-01	8.56E-02	2.35E-01	-7.08E-03	2.98E-01	8.89E-02	-1.17E-01	-1.69E-02	-1.32E-01	-2.11E-02	-1.48E-01	-3.02E-02	-1.77E-01	-3.70E-02
-4.04E-02	-7.08E-05	-1.07E-01	-9.99E-02	-2.02E-02	8.24E-02	9.53E-03	5.85E-04	7.44E-02	9.82E-02	1.14E-01	-6.26E-03	1.95E-01	9.68E-02	2.35E-01	-1.19E-02	2.96E-01	1.00E-01	-1.22E-01	-2.12E-02	-1.34E-01	-2.81E-02	-1.49E-01	-3.82E-02	-1.76E-01	-4.63E-02
-1.60E-02	-6.52E-03	-1.08E-01	-1.05E-01	-8.85E-03	9.20E-02	1.15E-02	-2.18E-04	9.23E-02	1.09E-01	1.19E-01	5.78E-04	1.97E-01	1.10E-01	2.29E-01	-2.89E-03	2.96E-01	1.06E-01	-1.23E-01	-7.34E-03	-1.37E-01	-1.68E-02	-1.52E-01	-3.79E-02	-1.81E-01	-5.24E-02
-1.52E-02	-1.83E-02	-8.53E-02	-8.86E-02	-1.93E-02	7.68E-02	1.31E-02	-1.06E-02	8.32E-02	9.46E-02	1.21E-01	-1.30E-02	1.93E-01	9.22E-02	2.29E-01	-1.55E-02	2.98E-01	8.45E-02	-1.16E-01	-1.11E-03	-1.28E-01	-1.11E-02	-1.43E-01	-3.19E-02	-1.71E-01	-4.96E-02
-2.61E-02	-1.28E-02	-8.64E-02	-1.07E-01	-1.83E-02	7.79E-02	6.56E-03	-9.65E-03	7.83E-02	9.90E-02	1.19E-01	-6.81E-03	1.89E-01	9.49E-02	2.28E-01	-6.04E-03	3.08E-01	8.91E-02	-1.12E-01	-6.77E-03	-1.25E-01	-1.63E-02	-1.41E-01	-3.63E-02	-1.71E-01	-5.18E-02
-1.51E-02	-1.21E-02	-8.81E-02	-8.53E-02	-2.11E-02	7.68E-02	1.10E-02	-3.57E-03	7.98E-02	1.00E-01	1.19E-01	-7.45E-03	1.90E-01	8.53E-02	2.32E-01	-7.01E-03	2.97E-01	8.36E-02	-1.16E-01	-1.26E-02	-1.28E-01	-2.31E-02	-1.45E-01	-3.54E-02	-1.78E-01	-4.55E-02
-1.76E-02	-1.55E-02	-1.08E-01	-7.86E-02	-2.61E-02	6.78E-02	1.38E-02	-4.09E-03	7.53E-02	9.09E-02	1.21E-01	-1.96E-03	1.93E-01	8.86E-02	2.32E-01	-2.72E-03	3.09E-01	9.04E-02	-1.19E-01	-2.68E-02	-1.34E-01	-2.88E-02	-1.51E-01	-3.33E-02	-1.79E-01	-3.52E-02
-1.27E-02	-2.38E-02	-8.50E-02	-8.96E-02	-2.08E-02	7.16E-02	7.26E-03	-8.53E-03	7.51E-02	9.01E-02	1.22E-01	-1.29E-02	1.93E-01	8.68E-02	2.38E-01	-6.99E-03	3.01E-01	9.45E-02	-1.15E-01	-1.99E-02	-1.29E-01	-2.64E-02	-1.44E-01	-4.30E-02	-1.75E-01	-4.91E-02
-3.07E-02	-1.68E-02	-1.07E-01	-8.57E-02	-3.38E-02	7.71E-02	1.87E-03	-1.08E-02	6.81E-02	9.54E-02	1.16E-01	-1.11E-02	1.88E-01	9.26E-02	2.34E-01	-1.52E-02	3.05E-01	9.25E-02	-1.18E-01	-1.10E-02	-1.34E-01	-1.32E-02	-1.51E-01	-2.58E-02	-1.77E-01	-3.47E-02
-3.55E-02	-8.74E-03	-1.07E-01	-9.62E-02	-2.26E-02	7.41E-02	2.88E-04	-6.09E-03	7.12E-02	8.57E-02	1.21E-01	-5.15E-03	1.92E-01	9.00E-02	2.34E-01	-5.22E-03	3.08E-01	1.05E-01	-1.20E-01	-3.22E-02	-1.33E-01	-3.25E-02	-1.48E-01	-3.99E-02	-1.72E-01	-3.91E-02
-1.92E-02	-1.55E-02	-1.02E-01	-9.05E-02	-3.16E-02	7.78E-02	7.04E-03	-1.93E-03	7.00E-02	9.80E-02	1.17E-01	-9.15E-03	1.91E-01	9.86E-02	2.33E-01	-1.19E-02	3.01E-01	9.14E-02	-1.21E-01	-2.93E-02	-1.34E-01	-2.89E-02	-1.48E-01	-3.66E-02	-1.71E-01	-3.78E-02
-9.71E-03	-8.62E-03	-8.82E-02	-8.68E-02	-1.73E-02	7.36E-02	1.47E-02	-7.56E-04	7.45E-02	8.94E-02	1.21E-01	-8.39E-03	1.96E-01	8.88E-02	2.35E-01	-7.80E-03	3.04E-01	8.95E-02	-1.15E-01	-1.67E-02	-1.32E-01	-1.82E-02	-1.46E-01	-3.40E-02	-1.73E-01	-4.24E-02

-2.17E-02	-1.57E-02	-9.04E-02	-1.01E-01	-2.15E-02	8.92E-02	7.80E-03	-4.98E-03	8.19E-02	9.43E-02	1.17E-01	-8.10E-03	1.89E-01	9.11E-02	2.30E-01	-1.10E-02	2.93E-01	8.97E-02	-1.19E-01	-1.39E-02	-1.33E-01	-2.15E-02	-1.44E-01	-4.37E-02	-1.68E-01	-5.45E-02
-1.64E-02	-1.46E-02	-8.69E-02	-9.40E-02	-2.37E-02	7.55E-02	1.47E-02	-4.47E-03	7.79E-02	9.32E-02	1.25E-01	-1.71E-03	1.89E-01	9.82E-02	2.25E-01	-5.87E-03	2.99E-01	9.23E-02	-1.16E-01	-3.10E-02	-1.31E-01	-2.93E-02	-1.47E-01	-3.40E-02	-1.76E-01	-3.91E-02
-2.76E-02	-8.07E-03	-1.03E-01	-9.94E-02	-1.25E-02	8.15E-02	1.17E-02	1.06E-03	7.54E-02	9.82E-02	1.15E-01	-3.10E-04	1.89E-01	1.06E-01	2.23E-01	-1.25E-04	2.97E-01	1.08E-01	-1.33E-01	-1.03E-02	-1.45E-01	-1.95E-02	-1.58E-01	-3.53E-02	-1.79E-01	-4.59E-02
-5.14E-03	-1.85E-02	-8.64E-02	-8.55E-02	-1.85E-02	7.74E-02	2.80E-02	-2.82E-03	7.83E-02	9.47E-02	1.27E-01	-7.64E-03	1.99E-01	1.00E-01	2.33E-01	-2.53E-03	3.01E-01	9.86E-02	-1.36E-01	-2.46E-02	-1.49E-01	-2.99E-02	-1.59E-01	-3.94E-02	-1.81E-01	-3.96E-02
-2.40E-02	-2.00E-02	-9.64E-02	-1.04E-01	-2.14E-02	7.82E-02	1.00E-02	-1.08E-02	7.67E-02	1.00E-01	1.20E-01	-1.28E-02	1.92E-01	1.02E-01	2.24E-01	-8.74E-03	2.98E-01	1.03E-01	-1.10E-01	-9.18E-03	-1.28E-01	-1.17E-02	-1.46E-01	-3.05E-02	-1.77E-01	-4.45E-02
-2.73E-02	-2.08E-02	-1.01E-01	-8.42E-02	-2.41E-02	7.00E-02	4.53E-03	-1.16E-02	7.85E-02	8.60E-02	1.24E-01	-1.23E-02	1.96E-01	8.52E-02	2.36E-01	-5.95E-03	2.92E-01	8.23E-02	-1.27E-01	-2.74E-02	-1.41E-01	-2.97E-02	-1.55E-01	-3.82E-02	-1.78E-01	-3.90E-02
-3.18E-02	-1.04E-02	-9.85E-02	-8.13E-02	-2.74E-02	8.04E-02	-5.91E-03	7.41E-03	7.60E-02	1.05E-01	1.15E-01	-7.04E-03	1.97E-01	1.02E-01	2.33E-01	3.82E-03	3.08E-01	9.81E-02	-1.30E-01	-3.17E-02	-1.42E-01	-3.28E-02	-1.52E-01	-3.77E-02	-1.71E-01	-4.01E-02
-4.15E-02	-1.17E-02	-1.03E-01	-9.25E-02	-2.97E-02	7.96E-02	-1.23E-02	-2.75E-03	7.53E-02	9.77E-02	1.15E-01	-8.05E-03	1.97E-01	9.35E-02	2.42E-01	-4.00E-03	3.12E-01	1.04E-01	-1.33E-01	-3.32E-02	-1.42E-01	-3.52E-02	-1.51E-01	-3.77E-02	-1.71E-01	-3.80E-02
-1.67E-02	-1.75E-02	-9.82E-02	-8.80E-02	-2.02E-02	7.87E-02	5.27E-03	2.76E-03	7.95E-02	1.02E-01	1.15E-01	-6.63E-03	1.94E-01	9.67E-02	2.31E-01	-1.39E-03	3.04E-01	1.01E-01	-1.18E-01	-2.77E-02	-1.34E-01	-2.48E-02	-1.52E-01	-3.03E-02	-1.79E-01	-3.75E-02
-2.15E-02	-1.73E-02	-1.01E-01	-8.78E-02	-2.76E-02	8.62E-02	3.22E-03	2.91E-03	7.73E-02	1.01E-01	1.16E-01	-5.89E-03	1.99E-01	9.85E-02	2.40E-01	-7.94E-03	3.12E-01	9.52E-02	-1.18E-01	-3.04E-02	-1.31E-01	-3.24E-02	-1.44E-01	-3.46E-02	-1.68E-01	-3.55E-02
-1.99E-02	-2.00E-02	-1.01E-01	-8.12E-02	-3.76E-02	8.98E-02	5.23E-03	-4.56E-03	7.61E-02	1.05E-01	1.10E-01	-1.52E-02	1.95E-01	9.90E-02	2.36E-01	-4.86E-03	3.12E-01	1.04E-01	-1.23E-01	-2.71E-02	-1.34E-01	-3.06E-02	-1.46E-01	-3.56E-02	-1.68E-01	-3.76E-02
-3.20E-02	-2.09E-02	-1.03E-01	-7.44E-02	-3.15E-02	7.64E-02	-1.48E-03	-9.52E-04	8.38E-02	9.61E-02	1.17E-01	-1.68E-02	2.02E-01	9.93E-02	2.24E-01	-9.29E-03	3.06E-01	9.47E-02	-1.28E-01	-3.54E-02	-1.39E-01	-3.77E-02	-1.47E-01	-4.05E-02	-1.66E-01	-3.77E-02
-1.25E-02	-2.15E-02	-9.48E-02	-8.98E-02	-1.86E-02	7.80E-02	1.07E-02	4.61E-04	7.49E-02	9.68E-02	1.19E-01	-7.44E-03	1.89E-01	9.50E-02	2.31E-01	-4.14E-03	3.01E-01	9.96E-02	-1.21E-01	-2.87E-02	-1.35E-01	-2.65E-02	-1.48E-01	-2.57E-02	-1.75E-01	-2.52E-02
-2.09E-02	-1.33E-02	-9.48E-02	-7.95E-02	-2.52E-02	7.65E-02	8.51E-03	-1.49E-03	7.60E-02	8.79E-02	1.22E-01	-7.80E-03	2.01E-01	8.86E-02	2.36E-01	-3.70E-03	3.11E-01	9.12E-02	-1.22E-01	-2.59E-02	-1.36E-01	-2.80E-02	-1.50E-01	-3.38E-02	-1.73E-01	-3.97E-02
-2.59E-02	-1.40E-02	-1.14E-01	-8.71E-02	-3.48E-02	6.99E-02	7.16E-03	-1.17E-03	5.97E-02	9.06E-02	1.23E-01	-1.22E-02	1.85E-01	9.72E-02	2.36E-01	-8.21E-04	3.00E-01	9.96E-02	-1.41E-01	-3.27E-02	-1.48E-01	-3.06E-02	-1.54E-01	-3.21E-02	-1.71E-01	-3.28E-02
-1.74E-02	-1.36E-02	-1.01E-01	-9.07E-02	-1.86E-02	7.01E-02	1.96E-02	2.85E-03	7.54E-02	9.28E-02	1.23E-01	-3.57E-03	1.88E-01	9.71E-02	2.31E-01	2.03E-03	2.99E-01	9.22E-02	-1.20E-01	-2.18E-02	-1.33E-01	-2.50E-02	-1.49E-01	-3.24E-02	-1.76E-01	-3.80E-02
-1.27E-02	-1.70E-02	-9.93E-02	-8.44E-02	-1.34E-02	7.56E-02	7.22E-03	-3.16E-03	8.46E-02	9.46E-02	1.24E-01	-5.04E-03	1.99E-01	9.26E-02	2.41E-01	2.07E-03	3.06E-01	9.92E-02	-1.20E-01	-2.36E-02	-1.36E-01	-2.57E-02	-1.52E-01	-3.09E-02	-1.80E-01	-3.65E-02
-1.58E-02	-1.83E-02	-1.18E-01	-7.97E-02	-3.28E-02	6.66E-02	2.83E-03	-6.66E-03	7.07E-02	9.12E-02	1.21E-01	-8.50E-03	1.87E-01	9.20E-02	2.35E-01	3.01E-03	3.08E-01	1.01E-01	-1.30E-01	-3.79E-02	-1.42E-01	-2.99E-02	-1.56E-01	-3.36E-02	-1.77E-01	-3.39E-02
-1.67E-02	-1.28E-02	-1.05E-01	-1.01E-01	-2.81E-02	7.20E-02	4.76E-03	7.15E-03	6.98E-02	9.86E-02	1.32E-01	1.29E-04	1.99E-01	1.00E-01	2.32E-01	-2.00E-04	3.12E-01	1.06E-01	-1.26E-01	-2.44E-02	-1.37E-01	-2.83E-02	-1.51E-01	-3.25E-02	-1.77E-01	-3.62E-02
-3.13E-02	-1.12E-02	-1.03E-01	-1.01E-01	-2.42E-02	8.73E-02	-2.60E-04	-1.27E-03	8.18E-02	1.01E-01	1.13E-01	-5.61E-03	1.92E-01	9.94E-02	2.31E-01	-2.63E-03	3.06E-01	1.01E-01	-1.27E-01	-3.20E-02	-1.37E-01	-2.59E-02	-1.47E-01	-2.72E-02	-1.68E-01	-2.90E-02
-3.96E-02	-1.37E-02	-9.60E-02	-9.62E-02	-2.41E-02	7.33E-02	2.12E-03	1.46E-03	7.43E-02	8.29E-02	1.19E-01	-1.48E-02	1.92E-01	8.76E-02	2.33E-01	-3.63E-03	3.13E-01	1.00E-01	-1.20E-01	-2.04E-02	-1.33E-01	-2.04E-02	-1.47E-01	-2.73E-02	-1.71E-01	-3.08E-02
-2.29E-02	-7.54E-03	-1.06E-01	-9.06E-02	-2.80E-02	6.95E-02	2.06E-02	-5.19E-03	6.44E-02	9.42E-02	1.22E-01	-4.09E-03	1.87E-01	9.29E-02	2.40E-01	-2.24E-03	3.03E-01	9.48E-02	-1.29E-01	-1.28E-02	-1.42E-01	-1.61E-02	-1.56E-01	-2.76E-02	-1.75E-01	-3.61E-02
-1.67E-02	-1.20E-02	-9.91E-02	-8.98E-02	-2.76E-02	7.55E-02	2.20E-02	-1.26E-03	6.77E-02	9.27E-02	1.21E-01	-7.17E-03	1.98E-01	8.87E-02	2.31E-01	-1.07E-02	2.87E-01	8.95E-02	-1.18E-01	-2.27E-02	-1.32E-01	-2.82E-02	-1.47E-01	-3.70E-02	-1.73E-01	-4.03E-02
-3.76E-02	-1.04E-02	-9.76E-02	-1.00E-01	-1.19E-02	8.61E-02	7.08E-03	-8.79E-03	7.86E-02	9.75E-02	1.14E-01	-1.10E-02	1.94E-01	1.00E-01	2.30E-01	-4.11E-03	3.07E-01	9.56E-02	-1.29E-01	-2.79E-02	-1.42E-01	-2.97E-02	-1.55E-01	-3.86E-02	-1.80E-01	-4.29E-02
-1.50E-02	-9.71E-03	-1.08E-01	-9.47E-02	-2.30E-02	8.35E-02	1.50E-02	-1.15E-03	7.29E-02	9.91E-02	1.23E-01	-2.60E-03	1.87E-01	9.50E-02	2.27E-01	-8.44E-03	3.02E-01	1.05E-01	-1.23E-01	-3.25E-02	-1.39E-01	-3.20E-02	-1.55E-01	-3.99E-02	-1.81E-01	-4.49E-02
-2.54E-02	-2.42E-03	-1.06E-01	-9.50E-02	-2.73E-02	8.53E-02	5.82E-03	3.38E-03	7.48E-02	1.04E-01	1.16E-01	-3.38E-03	1.91E-01	9.99E-02	2.35E-01	-8.59E-03	3.00E-01	1.05E-01	-1.25E-01	-2.87E-02	-1.37E-01	-3.01E-02	-1.48E-01	-3.45E-02	-1.69E-01	-3.84E-02
-3.98E-02	-1.82E-02	-1.06E-01	-8.75E-02	-2.61E-02	7.92E-02	-3.32E-03	-6.13E-03	7.68E-02	9.98E-02	1.13E-01	-1.06E-02	2.02E-01	9.78E-02	2.41E-01	-5.25E-03	3.05E-01	9.37E-02	-1.25E-01	-3.38E-02	-1.37E-01	-2.95E-02	-1.50E-01	-3.14E-02	-1.73E-01	-3.37E-02
-3.69E-02	1.35E-03	-9.56E-02	-9.69E-02	-1.10E-02	8.99E-02	9.22E-03	-5.52E-03	8.86E-02	1.04E-01	1.13E-01	-1.31E-02	2.04E-01	1.01E-01	2.33E-01	-1.22E-02	3.06E-01	9.64E-02	-1.26E-01	-2.96E-02	-1.40E-01	-3.26E-02	-1.53E-01	-4.13E-02	-1.77E-01	-4.90E-02
-3.66E-02	-8.63E-03	-1.00E-01	-9.11E-02	-1.77E-02	8.49E-02	5.42E-03	-3.70E-03	8.14E-02	9.75E-02	1.24E-01	-9.43E-03	1.96E-01	9.86E-02	2.31E-01	-4.57E-03	3.00E-01	1.01E-01	-1.27E-01	-3.08E-02	-1.42E-01	-3.00E-02	-1.55E-01	-3.15E-02	-1.80E-01	-3.76E-02
-3.26E-02	-1.15E-02	-1.00E-01	-1.10E-01	-1.71E-02	7.85E-02	-2.77E-03	-1.36E-03	8.76E-02	9.74E-02	1.14E-01	-7.95E-03	1.92E-01	1.08E-01	2.28E-01	-2.15E-03	3.00E-01	1.05E-01	-1.31E-01	-2.05E-02	-1.43E-01	-2.85E-02	-1.59E-01	-3.68E-02	-1.83E-01	-4.74E-02
-3.62E-02	-6.39E-03	-9.23E-02	-9.13E-02	-1.66E-02	8.73E-02	8.65E-03	-6.15E-03	7.73E-02	9.73E-02	1.31E-01	-1.28E-02	1.92E-01	9.51E-02	2.37E-01	-8.67E-03	2.91E-01	1.01E-01	-1.23E-01	-2.19E-02	-1.36E-01	-2.73E-02	-1.48E-01	-3.48E-02	-1.73E-01	-4.21E-02
-2.85E-02	-5.11E-03	-9.73E-02	-9.43E-02	-2.37E-02	8.20E-02	9.24E-03	-7.62E-03	7.99E-02	9.52E-02	1.14E-01	-9.05E-03	1.93E-01	9.81E-02	2.32E-01	-5.01E-03	3.12E-01	9.38E-02	-1.24E-01	-3.28E-02	-1.36E-01	-3.14E-02	-1.48E-01	-3.54E-02	-1.71E-01	-3.59E-02
-4.63E-02	-1.39E-02	-9.94E-02	-9.69E-02	-9.24E-03	8.68E-02	-2.54E-03	-6.10E-03	8.66E-02	9.77E-02	1.16E-01	-7.68E-03	1.95E-01	9.37E-02	2.33E-01	-3.19E-03	2.96E-01	1.05E-01	-1.29E-01	-1.20E-02	-1.41E-01	-2.09E-02	-1.55E-01	-3.54E-02	-1.79E-01	-4.51E-02
-4.65E-02	-6.48E-03	-8.95E-02	-9.66E-02	-6.20E-03	7.81E-02	1.13E-02	-4.23E-03	8.81E-02	9.31E-02	1.24E-01	-3.51E-03	1.93E-01	9.46E-02	2.33E-01	2.63E-03	2.90E-01	9.31E-02	-1.25E-01	-1.01E-02	-1.39E-01	-1.85E-02	-1.54E-01	-3.64E-02	-1.81E-01	-5.09E-02
-2.99E-02	-1.93E-02	-1.05E-01	-1.10E-01	-1.21E-02	8.58E-02	5.93E-03	-1.30E-02	8.07E-02	1.10E-01	1.15E-01	-7.47E-03	1.89E-01	1.09E-01	2.26E-01	-2.78E-03	2.88E-01	1.14E-01	-1.20E-01	-2.12E-02	-1.35E-01	-2.25E-02	-1.53E-01	-2.88E-02	-1.79E-01	-3.68E-02
-2.06E-02	-1.37E-02	-1.05E-01	-9.24E-02	-1.82E-02	8.17E-02	9.00E-03	1.49E-03	8.03E-02	9.78E-02	1.22E-01	5.30E-03	2.00E-01	9.89E-02	2.37E-01	-1.48E-03	3.10E-01	9.88E-02	-1.20E-01	-2.44E-02	-1.31E-01	-3.01E-02	-1.44E-01	-3.37E-02	-1.70E-01	-3.50E-02
-3.04E-02	-1.15E-02	-9.71E-02	-9.30E-02	-2.99E-02	8.80E-02	2.92E-03	8.63E-04	8.21E-02	1.01E-01	1.22E-01	-1.74E-03	1.95E-01	9.80E-02	2.33E-01	6.55E-04	3.06E-01	8.63E-02	-1.23E-01	-2.63E-02	-1.34E-01	-3.25E-02	-1.44E-01	-3.99E-02	-1.66E-01	-4.26E-02
-2.52E-02	-1.76E-02	-1.05E-01	-9.74E-02	-2.61E-02	7.86E-02	3.74E-03	-8.07E-03	7.00E-02	9.80E-02	1.17E-01	-1.23E-02	1.85E-01	1.00E-01	2.30E-01	-4.63E-03	2.94E-01	1.01E-01	-1.24E-01	-1.72E-02	-1.37E-01	-2.19E-02	-1.50E-01	-3.36E-02	-1.72E-01	-3.75E-02
-3.01E-02	-1.95E-02	-9.95E-02	-9.38E-02	-1.92E-02	8.68E-02	5.31E-04	-6.73E-03	7.31E-02	1.02E-01	1.17E-01	-7.92E-03	1.93E-01	1.04E-01	2.37E-01	-9.87E-04	2.99E-01	1.05E-01	-1.18E-01	-3.78E-02	-1.31E-01	-3.84E-02	-1.43E-01	-4.49E-02	-1.65E-01	-4.15E-02
-2.09E-02	-2.60E-02	-1.08E-01	-9.69E-02	-2.92E-02	8.16E-02	7.01E-03	-1.16E-02	7.31E-02	9.83E-02	1.19E-01	-8.84E-03	1.93E-01	1.02E-01	2.52E-01	2.99E-03	2.98E-01	9.97E-02	-1.16E-01	-3.05E-02	-1.29E-01	-2.86E-02	-1.44E-01	-3.18E-02	-1.69E-01	-3.25E-02
-2.59E-02	-7.27E-03	-1.04E-01	-9.94E-02	-2.08E-02	8.73E-02	2.86E-03	-3.18E-04	8.20E-02	1.03E-01	1.22E-01	-2.64E-03	1.99E-01	9.23E-02	2.37E-01	-8.51E-04	3.00E-01	9.40E-02	-1.25E-01	-2.81E-02	-1.38E-01	-3.13E-02	-1.50E-01	-3.68E-02	-1.74E-01	-3.73E-02
-2.88E-02	-1.80E-02	-1.08E-01	-8.96E-02	-3.10E-02	8.88E-02	-4.08E-03	8.90E-04	8.21E-02	1.03E-01	1.20E-01	-1.86E-03	2.01E-01	1.00E-01	2.31E-01	4.18E-04	3.15E-01	8.93E-02	-1.24E-01	-2.52E-02	-1.36E-01	-2.93E-02	-1.50E-01	-3.33E-02	-1.73E-01	-3.71E-02
-2.22E-02	-2.18E-02	-1.06E-01	-8.30E-02	-2.48E-02	7.37E-02	2.36E-03	-1.07E-02	7.68E-02	9.34E-02	1.23E-01	-7.45E-03	1.98E-01	9.93E-02	2.38E-01	-1.75E-03	3.06E-01	9.96E-02	-1.22E-01	-2.83E-02	-1.34E-01	-2.93E-02	-1.48E-01	-3.51E-02	-1.70E-01	-3.27E-02
-1.82E-02	-1.38E-02	-1.01E-01	-1.01E-01	-2.01E-02	9.25E-02	1.35E-02	1.00E-03	8.01E-02	1.12E-01	1.24E-01	-9.27E-04	1.91E-01	1.04E-01	2.33E-01	-1.54E-03	2.99E-01	9.84E-02	-1.16E-01	-2.75E-02	-1.29E-01	-3.18E-02	-1.43E-01	-3.72E-02	-1.71E-01	-3.95E-02
-2.60E-02	-1.13E-02	-1.06E-01	-9.72E-02	-2.48E-02	7.59E-02	1.13E-02	4.07E-03	8.35E-02	9.65E-02	1.21E-01	-4.78E-03	2.00E-01	9.85E-02	2.31E-01	-2.91E-03	3.05E-01	1.03E-01	-1.25E-01	-2.77E-02	-1.38E-01	-3.16E-02	-1.54E-01	-3.71E-02	-1.80E-01	-4.14E-02
-4.22E-02	-2.03E-02	-9.71E-02	-8.39E-02	-2.45E-02	7.20E-02	-5.92E-04	-1.26E-02	6.98E-02	9.21E-02	1.21E-01	-1.01E-02	1.98E-01	9.33E-02	2.35E-01	-5.04E-03	3.05E-01	8.99E-02	-1.24E-01	-2.48E-02	-1.35E-01	-3.13E-02	-1.47E-01	-4.08E-02	-1.71E-01	-4.38E-02
-4.70E-02	-1.10E-02	-1.03E-01	-1.01E-01	-1.74E-02	8.84E-02	4.60E-03	-1.05E-02	7.61E-02	1.01E-01	1.20E-01	-3.78E-03	1.92E-01	1.05E-01	2.35E-01	1.73E-04	3.04E-01	1.06E-01	-1.29E-01	-2.16E-02	-1.40E-01	-2.70E-02	-1.50E-01	-3.41E-02	-1.72E-01	-3.79E-02
-2.13E-02	-1.93E-02	-1.06E-01	-8.90E-02	-3.03E-02	8.41E-02	8.54E-03	-3.64E-03	7.07E-02	1.03E-01	1.25E-01	-7.54E-03	1.96E-01	1.03E-01	2.36E-01	-5.63E-03	3.06E-01	1.02E-01	-1.35E-01	-3.22E-02	-1.44E-01	-2.67E-02	-1.53E-01	-2.86E-02	-1.71E-01	-3.03E-02
-2.42E-02	-1.40E-02	-9.80E-02	-9.16E-02	-1.66E-02	8.86E-02	3.90E-03	2.10E-03	7.68E-02	1.03E-01	1.18E-01	-8.55E-03	1.95E-01	1.01E-01	2.34E-01	-6.77E-03	2.99E-01	1.04E-01	-1.24E-01	-1.61E-02	-1.32E-01	-2.90E-02	-1.44E-01	-4.49E-02	-1.70E-01	-5.20E-02
-3.70E-02	-8.45E-03	-1.08E-01	-8.91E-02	-2.22E-02	8.21E-02	-3.59E-03	5.24E-03	8.04E-02	9.87E-02	1.33E-01	5.29E-03	2.00E-01	1.06E-01	2.37E-01	-6.83E-03	3.11E-01	1.08E-01	-1.26E-01	-3.82E-02	-1.36E-01	-4.44E-02	-1.48E-01	-5.05E-02	-1.73E-01	-5.13E-02
-4.74E-02	-1.67E-02	-1.06E-01	-9.07E-02	-1.85E-02	7.70E-02	2.99E-03	-1.26E-02	7.94E-02	9.60E-02	1.25E-01	-9.44E-03	1.97E-01	9.91E-02	2.38E-01	-2.35E-03	3.03E-01	1.01E-01	-1.22E-01	-2.41E-02	-1.35E-01	-2.39E-02	-1.50E-01	-2.87E-02	-1.75E-01	-3.16E-02
-3.68E-02	-1.73E-02	-9.02E-02	-9.27E-02	-2.15E-02	7.71E-02	-1.77E-02	-2.99E-03	8.06E-02	9.92E-02	1.18E-01	-1.08E-03	2.05E-01	9.62E-02	2.37E-01	-7.91E-03	3.13E-01	9.14E-02	-1.24E-01	-2.06E-02	-1.38E-01	-2.45E-02	-1.50E-01	-3.56E-02	-1.71E-01	-4.26E-02
-6.09E-02	-1.59E-02	-1.09E-01	-7.89E-02	-1.64E-02	7.28E-02	-8.25E-03	-1.37E-02	8.08E-02	8.91E-02	1.24E-01	-6.34E-03	1.99E-01	8.82E-02	2.38E-01	-4.61E-03	3.08E-01	8.45E-02	-1.24E-01	-3.03E-02	-1.37E-01	-3.19E-02	-1.51E-01	-3.50E-02	-1.76E-01	-3.56E-02
-3.49E-02	-1.83E-02	-1.02E-01	-8.35E-02	-2.05E-02	8.22E-02	-6.03E-04	3.03E-03	8.07E-02	9.16E-02	1.21E-01	-3.66E-03	2.05E-01	9.05E-02	2.29E-01	-2.89E-03	3.06E-01	8.31E-02	-1.26E-01	-2.37E-02	-1.38E-01	-3.07E-02	-1.50E-01	-4.26E-02	-1.70E-01	-4.86E-02
-3.55E-02	-2.13E-02	-9.23E-02	-9.13E-02	-1.44E-02	8.11E-02	2.60E-03	-7.43E-03	8.77E-02	9.54E-02	1.20E-01	-5.25E-03	2.01E-01	9.21E-02	2.36E-01	-3.07E-03	3.02E-01	9.69E-02	-1.16E-01	-2.87E-02	-1.29E-01	-3.26E-02	-1.43E-01	-3.82E-02	-1.71E-01	-3.93E-02
-1.61E-02	-1.29E-02	-9.56E-02	-9.67E-02	-1.80E-02	8.28E-02	1.22E-02	9.02E-04	7.78E-02	9.60E-02	1.21E-01	-7.07E-03	2.02E-01	9.50E-02	2.25E-01	-5.15E-03	3.07E-01	9.41E-02	-1.12E-01	-2.19E-02	-1.27E-01	-2.59E-02	-1.44E-01	-3.50E-02	-1.73E-01	-3.93E-02
-3.55E-02	-2.29E-02	-8.85E-02	-8.84E-02	-1.55E-02	8.12E-02	-2.89E-04	-5.68E-03	8.14E-02	9.60E-02	1.14E-01	-1.17E-02	1.97E-01	1.01E-01	2.37E-01	-6.60E-03	2.99E-01	9.87E-02	-1.22E-01	-2.09E-02	-1.31E-01	-3.08E-02	-1.41E-01	-4.18E-02	-1.65E-01	-4.05E-02
-4.41E-02	-2.26E-02	-1.03E-01	-8.99E-02	-9.33E-03	8.47E-02	6.62E-03	-6.74E-03	8.46E-02	9.51E-02	1.23E-01	-3.91E-03	1.96E-01	9.81E-02	2.43E-01	-3.86E-03	3.07E-01	9.71E-02	-1.27E-01	-2.80E-02	-1.40E-01	-3.35E-02	-1.54E-01	-4.45E-02	-1.78E-01	-4.96E-02
-5.56E-02	-1.59E-02	-9.60E-02	-8.60E-02	-5.66E-03	7.75E-02	-1.85E-03	3.04E-03	8.12E-02	9.08E-02	1.16E-01	-1.13E-02	1.96E-01	9.15E-02	2.42E-01	3.93E-04	3.06E-01	8.82E-02	-1.27E-01	-2.10E-02	-1.37E-01	-2.86E-02	-1.49E-01	-3.67E-02	-1.72E-01	-4.26E-02
-3.27E-02	-1.91E-02	-9.40E-02	-9.17E-02	-2.08E-02	7.60E-02	3.38E-03	-1.27E-04	8.42E-02	9.44E-02	1.22E-01	-6.65E-03	1.99E-01	1.02E-01	2.37E-01	-6.24E-03	3.02E-01	9.26E-02	-1.20E-01	-2.66E-02	-1.31E-01	-3.53E-02	-1.45E-01	-4.84E-02	-1.72E-01	-5.02E-02
-3.11E-02	-1.10E-02	-9.52E-02	-9.38E-02	-1.91E-02	9.09E-02	1.16E-02	-8.41E-03	7.49E-02	1.01E-01	1.22E-01	-1.13E-02	1.94E-01	9.38E-02	2.33E-01	-8.28E-03	2.90E-01	9.79E-02	-1.24E-01	-2.53E-02	-1.36E-01	-3.24E-02	-1.49E-01	-4.50E-02	-1.71E-01	-5.10E-02
-4.36E-02	-1.88E-02	-9.25E-02	-9.22E-02	-1.33E-02	8.12E-02	8.84E-04	-8.96E-03	8.36E-02	9.11E-02	1.21E-01	-1.21E-02	2.02E-01	8.66E-02	2.34E-01	-6.86E-03	3.02E-01	9.09E-02	-1.25E-01	-9.79E-03	-1.37E-01	-1.91E-02	-1.48E-01	-3.58E-02	-1.73E-01	-4.41E-02
-3.31E-02	-2.11E-02	-9.56E-02	-9.32E-02	-2.03E-02	7.59E-02	-1.09E-05	-8.75E-03	7.58E-02	9.46E-02	1.13E-01	-1.46E-02	1.94E-01	9.58E-02	2.30E-01	-5.49E-04	3.06E-01	9.42E-02	-1.18E-01	-2.71E-02	-1.29E-01	-2.96E-02	-1.43E-01	-3.39E-02	-1.68E-01	-3.70E-02
-4.11E-02	-2.11E-02	-8.78E-02	-9.13E-02	-8.19E-03	7.36E-02	3.85E-04	-7.58E-03	8.73E-02	8.76E-02	1.21E-01	-1.25E-02	1.94E-01	8.57E-02	2.38E-01	-1.82E-03	2.92E-01	8.97E-02	-1.26E-01	-1.39E-02	-1.39E-01	-2.26E-02	-1.50E-01	-3.83E-02	-1.74E-01	-4.19E-02
-3.15E-02	-1.75E-02	-1.10E-01	-8.83E-02	-2.03E-02	8.22E-02	-1.73E-03	-3.84E-03	7.84E-02	1.02E-01	1.22E-01	1.47E-04	2.06E-01	9.48E-02	2.40E-01	-2.77E-03	3.16E-01	9.62E-02	-1.23E-01	-2.19E-02	-1.37E-01	-2.63E-02	-1.53E-01	-3.41E-02	-1.77E-01	-3.77E-02
-3.88E-02	-1.88E-02	-9.67E-02	-9.02E-02	-2.03E-02	7.80E-02	6.84E-03	-8.91E-03	8.12E-02	9.76E-02	1.17E-01	-8.44E-03	1.97E-01	9.51E-02	2.30E-01	-6.51E-04	3.02E-01	1.04E-01	-1.21E-01	-3.27E-02	-1.36E-01	-3.33E-02	-1.51E-01	-4.22E-02	-1.79E-01	-4.58E-02
-4.55E-02	-1.76E-02	-9.57E-02	-8.79E-02	-1.71E-02	7.44E-02	-3.60E-03	-4.09E-03	7.50E-02	9.19E-02	1.24E-01	-1.23E-02	1.99E-01	8.64E-02	2.40E-01	-4.32E-03	3.02E-01	9.31E-02	-1.23E-01	-2.29E-02	-1.36E-01	-2.28E-02	-1.52E-01	-2.83E-02	-1.76E-01	-3.77E-02
-3.96E-02	-1.91E-02	-9.76E-02	-8.18E-02	-1.10E-02	7.79E-02	-3.83E-03	-4.29E-03	8.05E-02	9.18E-02	1.17E-01	-8.85E-03	2.01E-01	8.98E-02	2.32E-01	-5.49E-03	3.11E-01	8.66E-02	-1.26E-01	-2.76E-02	-1.39E-01	-2.93E-02	-1.54E-01	-3.36E-02	-1.79E-01	-4.03E-02
-2.26E-02	-1.92E-02	-1.04E-01	-1.00E-01	-1.91E-02	8.15E-02	1.04E-02	-4.55E-03	8.00E-02	9.67E-02	1.20E-01	-9.40E-03	1.95E-01	9.19E-02	2.29E-01	-8.49E-03	3.06E-01	9.43E-02	-1.22E-01	-2.43E-02	-1.35E-01	-2.80E-02	-1.49E-01	-3.64E-02	-1.73E-01	-3.56E-02
-4.96E-02	-2.26E-02	-9.28E-02	-8.92E-02	-1.67E-02	7.52E-02	-8.97E-03	-1.12E-02	7.53E-02	9.36E-02	1.15E-01	-1.10E-02	1.89E-01	9.68E-02	2.37E-01	-5.29E-03	2.99E-01	9.87E-02	-1.20E-01	-1.71E-02	-1.31E-01	-2.24E-02	-1.45E-01	-2.82E-02	-1.72E-01	-3.29E-02
-4.83E-02	-2.17E-02	-9.96E-02	-9.14E-02	-1.97E-02	7.14E-02	-4.19E-03	-9.13E-03	7.69E-02	9.02E-02	1.24E-01	-6.62E-03	1.93E-01	9.49E-02	2.43E-01	2.26E-03	2.98E-01	1.01E-01	-1.21E-01	-2.74E-02	-1.35E-01	-2.85E-02	-1.51E-01	-3.32E-02	-1.79E-01	-3.79E-02
-3.51E-02	-1.65E-02	-1.04E-01	-9.35E-02	-2.62E-02	6.74E-02	4.86E-03	-1.50E-03	6.96E-02	8.24E-02	1.18E-01	-6.92E-03	1.85E-01	9.24E-02	2.35E-01	-3.87E-03	2.93E-01	9.94E-02	-1.23E-01	-1.18E-02	-1.38E-01	-1.54E-02	-1.51E-01	-3.04E-02	-1.74E-01	-3.75E-02
-2.84E-02	-2.43E-02	-1.01E-01	-8.97E-02	-1.90E-02	7.40E-02	2.64E-03	-1.20E-02	8.38E-02	9.40E-02	1.24E-01	-8.98E-03	2.02E-01	9.57E-02	2.32E-01	-3.01E-03	3.00E-01	9.47E-02	-1.27E-01	-2.20E-02	-1.39E-01	-2.69E-02	-1.52E-01	-3.22E-02	-1.77E-01	-3.69E-02
-4.34E-02	-1.91E-02	-9.59E-02	-8.63E-02	-2.74E-02	7.14E-02	-1.03E-03	-2.23E-03	7.34E-02	9.42E-02	1.15E-01	-1.04E-02	2.02E-01	8.92E-02	2.38E-01	-4.15E-03	3.12E-01	8.94E-02	-1.21E-01	-2.13E-02	-1.34E-01	-2.46E-02	-1.49E-01	-3.29E-02	-1.74E-01	-4.06E-02
-4.38E-02	-1.44E-02	-1.07E-01	-8.42E-02	-1.96E-02	6.69E-02	-6.59E-03	-2.33E-03	7.44E-02	8.88E-02	1.22E-01	-1.29E-02	2.02E-01	9.35E-02	2.40E-01	-4.31E-03	3.15E-01	8.81E-02	-1.26E-01	-1.65E-02	-1.39E-01	-1.89E-02	-1.51E-01	-3.31E-02	-1.72E-01	-3.81E-02
-2.71E-02	-2.02E-02	-1.01E-01	-8.37E-02	-2.55E-02	7.31E-02	-1.60E-03	-4.75E-03	7.63E-02	8.42E-02	1.23E-01	-1.07E-02	1.99E-01	8.81E-02	2.40E-01	-3.92E-03	3.04E-01	9.35E-02	-1.25E-01	-2.42E-02	-1.37E-01	-2.53E-02	-1.50E-01	-2.81E-02	-1.74E-01	-3.00E-02
-3.29E-02	-1.60E-02	-9.90E-02	-9.38E-02	-2.10E-02	7.93E-02	5.48E-03	-6.13E-03	8.37E-02	9.52E-02	1.18E-01	-1.02E-02	1.98E-01	9.25E-02	2.29E-01	-2.98E-03	2.99E-01	9.44E-02	-1.23E-01	-2.40E-02	-1.35E-01	-2.84E-02	-1.47E-01	-3.36E-02	-1.72E-01	-3.70E-02
-2.86E-02	-1.76E-02	-1.00E-01	-9.09E-02	-1.53E-02	6.97E-02	-1.87E-03	-5.79E-03	7.28E-02	8.27E-02	1.22E-01	-1.32E-02	1.94E-01	8.80E-02	2.33E-01	-2.12E-03	2.97E-01	9.64E-02	-1.23E-01	-2.49E-02	-1.38E-01	-2.78E-02	-1.51E-01	-3.65E-02	-1.75E-01	-3.79E-02
-3.39E-02	-1.42E-02	-1.05E-01	-9.24E-02	-4.54E-02	8.74E-02	-2.69E-03	-7.61E-03	5.90E-02	1.04E-01	1.29E-01	-1.11E-02	1.89E-01	9.82E-02	2.46E-01	-1.18E-02	3.05E-01	8.95E-02	-1.17E-01	-1.77E-02	-1.24E-01	-2.73E-02	-1.36E-01	-3.82E-02	-1.62E-01	-4.26E-02
-3.78E-02	-6.61E-03	-9.90E-02	-8.88E-02	-2.50E-02	8.60E-02	5.10E-03	-2.31E-03	7.55E-02	1.01E-01	1.22E-01	-1.09E-02	1.95E-01	9.28E-02	2.37E-01	-8.85E-03	3.04E-01	9.09E-02	-1.25E-01	-1.79E-02	-1.36E-01	-2.67E-02	-1.48E-01	-3.89E-02	-1.71E-01	-4.48E-02
-2.96E-02	-7.03E-03	-1.03E-01	-8.60E-02	-1.31E-02	8.65E-02	1.07E-03	-9.15E-03	9.03E-02	9.95E-02	1.19E-01	-1.14E-02	1.89E-01	9.70E-02	2.30E-01	-6.01E-03	2.94E-01	9.33E-02	-1.21E-01	-3.00E-02	-1.36E-01	-3.40E-02	-1.52E-01	-4.43E-02	-1.80E-01	-4.54E-02
-2.83E-02	-7.97E-03	-1.10E-01	-8.98E-02	-2.53E-02	6.60E-02	9.82E-03	-1.94E-03	8.23E-02	8.80E-02	1.07E-01	-1.28E-02	1.92E-01	8.82E-02	2.35E-01	-6.80E-03	3.05E-01	8.99E-02	-1.29E-01	-1.90E-02	-1.42E-01	-2.01E-02	-1.55E-01	-2.14E-02	-1.75E-01	-2.74E-02
-4.04E-02	-1.99E-02	-1.18E-01	-9.82E-02	-1.30E-02	7.53E-02	-7.10E-03	-1.23E-02	7.76E-02	9.75E-02	1.15E-01	-9.65E-03	1.96E-01	1.00E-01	2.35E-01	2.42E-03	3.03E-01	1.08E-01	-1.29E-01	-1.50E-02	-1.43E-01	-2.36E-02	-1.61E-01	-3.67E-02	-1.87E-01	-4.27E-02

-3.53E-02	-1.86E-02	-1.01E-01	-8.70E-02	-1.39E-02	8.78E-02	1.67E-03	-2.34E-03	8.82E-02	9.68E-02	1.28E-01	-1.04E-02	2.02E-01	9.39E-02	2.46E-01	-5.22E-03	3.03E-01	9.63E-02	-1.24E-01	-2.35E-02	-1.37E-01	-2.76E-02	-1.52E-01	-2.99E-02	-1.78E-01	-3.69E-02
-3.89E-02	-1.49E-02	-1.03E-01	-1.06E-01	-1.90E-02	8.08E-02	-2.16E-03	-1.30E-03	7.48E-02	1.03E-01	1.16E-01	-8.29E-03	1.86E-01	9.48E-02	2.45E-01	-2.79E-03	2.85E-01	1.08E-01	-1.22E-01	-3.04E-02	-1.36E-01	-3.57E-02	-1.51E-01	-4.43E-02	-1.76E-01	-4.47E-02
-3.28E-02	-1.09E-02	-1.04E-01	-8.24E-02	-2.26E-02	8.37E-02	-2.84E-03	-2.04E-03	8.36E-02	1.07E-01	1.16E-01	-1.16E-02	1.99E-01	9.70E-02	2.41E-01	-1.15E-02	3.04E-01	9.53E-02	-1.24E-01	-2.39E-02	-1.36E-01	-3.00E-02	-1.50E-01	-3.69E-02	-1.74E-01	-4.25E-02
-3.46E-02	-1.42E-02	-1.03E-01	-8.47E-02	-7.80E-03	8.33E-02	-4.27E-03	-4.53E-03	8.87E-02	9.97E-02	1.17E-01	-1.42E-02	1.97E-01	1.01E-01	2.34E-01	-3.71E-03	2.98E-01	1.06E-01	-1.26E-01	-3.17E-02	-1.39E-01	-3.40E-02	-1.53E-01	-3.77E-02	-1.80E-01	-3.93E-02
-2.85E-02	-2.18E-02	-1.09E-01	-8.32E-02	-2.67E-02	7.21E-02	2.48E-03	-3.22E-03	6.34E-02	9.18E-02	1.26E-01	-1.06E-02	1.90E-01	9.25E-02	2.45E-01	-1.80E-03	3.00E-01	9.19E-02	-1.17E-01	-2.94E-02	-1.31E-01	-3.33E-02	-1.46E-01	-4.27E-02	-1.72E-01	-4.22E-02
-4.53E-02	-1.37E-02	-1.10E-01	-7.54E-02	-3.71E-02	7.31E-02	2.48E-04	-6.58E-03	8.01E-02	8.97E-02	1.25E-01	-8.01E-03	1.97E-01	9.32E-02	2.42E-01	-3.16E-03	3.05E-01	9.64E-02	-1.23E-01	-2.74E-02	-1.35E-01	-3.34E-02	-1.47E-01	-4.23E-02	-1.70E-01	-4.37E-02
-2.12E-02	-1.45E-02	-1.01E-01	-9.93E-02	-2.96E-02	7.61E-02	1.63E-02	-8.30E-03	7.01E-02	1.01E-01	1.33E-01	-7.80E-03	1.85E-01	9.66E-02	2.43E-01	-2.08E-03	2.92E-01	9.97E-02	-1.14E-01	-1.65E-02	-1.28E-01	-2.23E-02	-1.44E-01	-3.59E-02	-1.72E-01	-4.26E-02
-2.92E-02	-1.68E-02	-1.13E-01	-9.54E-02	-1.76E-02	6.60E-02	2.86E-03	-1.32E-02	7.44E-02	9.07E-02	1.20E-01	-1.48E-02	1.87E-01	9.03E-02	2.38E-01	-4.77E-03	2.89E-01	9.73E-02	-1.25E-01	-8.14E-03	-1.35E-01	-1.97E-02	-1.49E-01	-3.37E-02	-1.75E-01	-3.98E-02
-2.31E-02	-2.09E-02	-1.05E-01	-8.81E-02	-2.82E-02	6.70E-02	2.60E-03	-6.35E-03	7.49E-02	9.43E-02	1.15E-01	-1.38E-02	1.95E-01	9.46E-02	2.39E-01	-6.91E-03	3.06E-01	8.98E-02	-1.16E-01	-2.35E-02	-1.29E-01	-2.36E-02	-1.44E-01	-3.13E-02	-1.69E-01	-3.15E-02
-2.71E-02	-2.14E-02	-9.24E-02	-8.62E-02	-2.79E-02	7.27E-02	-8.91E-03	-1.02E-02	8.14E-02	9.40E-02	1.17E-01	-1.48E-02	1.98E-01	9.43E-02	2.38E-01	-7.75E-03	2.96E-01	9.13E-02	-1.19E-01	-2.21E-02	-1.32E-01	-2.37E-02	-1.46E-01	-3.11E-02	-1.72E-01	-3.31E-02
-1.93E-02	-1.92E-02	-1.01E-01	-8.44E-02	-2.95E-02	8.13E-02	8.96E-03	-3.37E-03	7.74E-02	1.00E-01	1.26E-01	-3.69E-03	1.98E-01	9.73E-02	2.40E-01	-1.40E-02	3.04E-01	8.34E-02	-1.14E-01	-3.46E-02	-1.27E-01	-3.79E-02	-1.44E-01	-4.07E-02	-1.73E-01	-4.31E-02
-2.56E-02	-1.57E-02	-9.98E-02	-8.91E-02	-3.47E-02	8.10E-02	-6.46E-03	-2.62E-03	7.01E-02	1.00E-01	1.14E-01	-9.62E-03	1.86E-01	9.19E-02	2.36E-01	-1.17E-02	2.90E-01	9.89E-02	-1.16E-01	-2.34E-02	-1.29E-01	-2.59E-02	-1.43E-01	-2.90E-02	-1.68E-01	-2.96E-02
-4.89E-02	-1.32E-02	-1.13E-01	-8.54E-02	-2.32E-02	7.83E-02	-8.93E-03	-9.29E-03	8.24E-02	9.77E-02	1.16E-01	-2.08E-02	1.97E-01	9.52E-02	2.45E-01	-7.84E-03	3.10E-01	9.53E-02	-1.29E-01	-2.74E-02	-1.40E-01	-2.81E-02	-1.52E-01	-3.28E-02	-1.72E-01	-3.34E-02
-3.25E-02	-2.05E-02	-9.26E-02	-9.02E-02	-2.37E-02	7.68E-02	1.19E-05	-7.54E-03	7.57E-02	9.66E-02	1.26E-01	-8.98E-03	2.00E-01	9.38E-02	2.42E-01	-1.06E-02	3.07E-01	9.33E-02	-1.15E-01	-2.36E-02	-1.26E-01	-3.02E-02	-1.39E-01	-3.84E-02	-1.66E-01	-4.14E-02
-3.48E-02	-9.40E-03	-1.05E-01	-9.56E-02	-1.54E-02	8.47E-02	-2.64E-04	-5.49E-03	8.45E-02	1.03E-01	1.18E-01	-8.20E-03	2.00E-01	9.81E-02	2.30E-01	-6.47E-03	3.04E-01	9.47E-02	-1.23E-01	-1.36E-02	-1.36E-01	-2.12E-02	-1.50E-01	-3.41E-02	-1.74E-01	-3.74E-02
-3.53E-02	-1.27E-02	-9.89E-02	-9.38E-02	-1.88E-02	7.62E-02	-1.39E-03	-5.20E-03	8.01E-02	9.45E-02	1.23E-01	-1.29E-02	1.99E-01	9.50E-02	2.39E-01	-9.68E-03	3.01E-01	8.50E-02	-1.22E-01	-2.72E-02	-1.34E-01	-2.88E-02	-1.48E-01	-3.08E-02	-1.74E-01	-3.72E-02
-3.43E-02	-1.35E-02	-1.03E-01	-9.05E-02	-9.63E-03	7.33E-02	-1.99E-03	-2.80E-03	9.38E-02	1.01E-01	1.20E-01	-1.17E-02	1.97E-01	9.16E-02	2.36E-01	-5.71E-03	3.06E-01	9.39E-02	-1.28E-01	-2.83E-02	-1.38E-01	-3.25E-02	-1.49E-01	-3.81E-02	-1.73E-01	-3.83E-02
-5.62E-02	4.60E-03	-8.64E-02	-9.56E-02	-1.07E-02	7.57E-02	-2.01E-03	-3.98E-03	8.88E-02	9.13E-02	1.20E-01	-8.79E-03	1.99E-01	8.83E-02	2.32E-01	-1.06E-02	3.04E-01	8.03E-02	-1.26E-01	-2.63E-02	-1.40E-01	-2.74E-02	-1.51E-01	-3.60E-02	-1.75E-01	-3.86E-02
-2.82E-02	-9.19E-03	-8.48E-02	-9.38E-02	-2.71E-02	7.68E-02	4.38E-04	-7.85E-04	7.58E-02	9.46E-02	1.17E-01	-1.13E-02	1.94E-01	9.24E-02	2.43E-01	-6.18E-03	3.02E-01	9.38E-02	-1.19E-01	-1.39E-02	-1.33E-01	-1.90E-02	-1.44E-01	-3.28E-02	-1.67E-01	-4.03E-02
-3.62E-02	-1.13E-02	-9.30E-02	-8.69E-02	-2.06E-02	7.80E-02	-2.70E-03	-2.33E-03	7.57E-02	1.01E-01	1.21E-01	-1.15E-02	1.93E-01	9.73E-02	2.43E-01	-1.00E-02	2.95E-01	9.63E-02	-1.18E-01	-1.86E-02	-1.27E-01	-2.66E-02	-1.42E-01	-3.64E-02	-1.69E-01	-4.58E-02
-3.80E-02	-1.60E-02	-1.03E-01	-9.86E-02	-5.24E-03	7.75E-02	-6.57E-03	-5.81E-03	8.16E-02	9.71E-02	1.19E-01	-1.00E-02	1.96E-01	9.33E-02	2.36E-01	-6.89E-03	3.02E-01	9.54E-02	-1.24E-01	-2.16E-02	-1.38E-01	-2.62E-02	-1.54E-01	-3.52E-02	-1.79E-01	-4.29E-02
-2.78E-02	-4.78E-03	-1.03E-01	-8.95E-02	-2.79E-02	8.62E-02	6.55E-03	-1.26E-03	7.77E-02	9.80E-02	1.25E-01	-4.10E-03	1.95E-01	9.78E-02	2.37E-01	-8.15E-03	3.00E-01	9.12E-02	-1.31E-01	-2.45E-02	-1.42E-01	-2.79E-02	-1.52E-01	-3.61E-02	-1.70E-01	-3.96E-02
-2.76E-02	-4.12E-03	-9.21E-02	-9.19E-02	-1.96E-02	7.60E-02	6.53E-03	5.00E-03	8.15E-02	9.42E-02	1.23E-01	-1.22E-02	1.94E-01	8.61E-02	2.45E-01	-1.25E-02	2.93E-01	8.46E-02	-1.16E-01	-1.44E-02	-1.29E-01	-1.99E-02	-1.43E-01	-3.19E-02	-1.70E-01	-3.67E-02
-4.17E-02	-3.32E-03	-8.91E-02	-8.96E-02	-2.24E-02	7.59E-02	1.38E-02	1.39E-02	7.89E-02	9.96E-02	1.16E-01	-4.99E-03	1.98E-01	9.25E-02	2.33E-01	-9.24E-03	3.07E-01	8.86E-02	-1.21E-01	-2.51E-02	-1.30E-01	-3.25E-02	-1.38E-01	-4.09E-02	-1.60E-01	-4.44E-02
-3.54E-02	-6.07E-03	-8.63E-02	-9.83E-02	-5.99E-03	7.89E-02	5.25E-03	-1.19E-03	8.68E-02	9.36E-02	1.17E-01	-6.92E-03	1.94E-01	8.53E-02	2.38E-01	-7.59E-03	3.00E-01	8.27E-02	-1.22E-01	-2.53E-02	-1.35E-01	-2.97E-02	-1.49E-01	-3.80E-02	-1.76E-01	-4.53E-02
-3.34E-02	-2.50E-03	-1.02E-01	-9.36E-02	-1.58E-02	8.34E-02	6.88E-03	1.46E-03	8.61E-02	1.01E-01	1.24E-01	-1.42E-03	1.96E-01	8.97E-02	2.36E-01	1.09E-03	2.97E-01	1.02E-01	-1.26E-01	-2.37E-02	-1.39E-01	-2.59E-02	-1.53E-01	-3.21E-02	-1.77E-01	-3.88E-02
-2.04E-02	-5.21E-03	-7.93E-02	-9.89E-02	-1.75E-02	7.46E-02	1.14E-03	5.69E-03	8.70E-02	9.21E-02	1.23E-01	-5.53E-03	1.94E-01	8.47E-02	2.40E-01	-9.50E-03	3.00E-01	7.88E-02	-1.15E-01	-4.11E-03	-1.27E-01	-1.50E-02	-1.40E-01	-3.64E-02	-1.66E-01	-5.27E-02
-2.59E-02	-1.38E-02	-9.91E-02	-9.36E-02	-1.73E-02	8.35E-02	1.54E-03	-4.34E-03	7.95E-02	1.01E-01	1.20E-01	1.20E-03	1.89E-01	9.95E-02	2.33E-01	-6.74E-03	2.99E-01	9.86E-02	-1.19E-01	-1.53E-02	-1.35E-01	-1.94E-02	-1.50E-01	-3.52E-02	-1.77E-01	-4.40E-02
-2.43E-02	-1.67E-02	-9.61E-02	-8.91E-02	-1.58E-02	7.93E-02	3.69E-03	-2.01E-03	7.90E-02	9.56E-02	1.19E-01	-1.03E-02	1.95E-01	8.89E-02	2.38E-01	-7.95E-03	3.07E-01	8.32E-02	-1.30E-01	-1.19E-02	-1.42E-01	-1.93E-02	-1.56E-01	-3.08E-02	-1.81E-01	-4.19E-02
-2.42E-02	-1.50E-02	-1.10E-01	-8.20E-02	-3.26E-02	7.48E-02	-1.52E-03	-4.17E-03	6.46E-02	8.33E-02	1.23E-01	-1.15E-02	1.89E-01	8.33E-02	2.43E-01	-3.34E-03	3.09E-01	8.68E-02	-1.29E-01	-2.38E-02	-1.40E-01	-2.29E-02	-1.51E-01	-2.58E-02	-1.70E-01	-2.59E-02
-2.79E-02	-1.97E-02	-1.05E-01	-8.75E-02	-2.68E-02	6.43E-02	3.24E-03	-7.35E-03	6.94E-02	8.06E-02	1.30E-01	-7.96E-03	1.80E-01	8.45E-02	2.38E-01	-1.95E-04	2.96E-01	9.36E-02	-1.36E-01	-2.53E-02	-1.47E-01	-2.35E-02	-1.55E-01	-2.40E-02	-1.75E-01	-2.67E-02
-1.45E-02	-1.64E-02	-1.11E-01	-9.17E-02	-1.65E-02	7.89E-02	1.19E-02	4.36E-05	7.33E-02	9.33E-02	1.24E-01	3.21E-04	1.87E-01	9.26E-02	2.36E-01	6.14E-04	2.98E-01	8.97E-02	-1.20E-01	-1.60E-02	-1.34E-01	-2.40E-02	-1.52E-01	-2.93E-02	-1.81E-01	-3.47E-02
-2.68E-02	-1.41E-02	-9.66E-02	-8.31E-02	-8.88E-03	6.82E-02	3.85E-03	-2.95E-03	7.57E-02	8.42E-02	1.23E-01	-9.19E-03	1.90E-01	8.48E-02	2.38E-01	1.48E-05	2.92E-01	9.29E-02	-1.32E-01	-2.71E-02	-1.47E-01	-3.27E-02	-1.62E-01	-4.36E-02	-1.88E-01	-4.78E-02
-3.96E-02	-1.96E-02	-1.20E-01	-8.42E-02	-1.94E-02	7.94E-02	-7.94E-03	-5.10E-03	8.11E-02	9.88E-02	1.22E-01	-4.30E-03	1.95E-01	9.48E-02	2.37E-01	-1.23E-03	3.08E-01	9.84E-02	-1.40E-01	-3.36E-02	-1.50E-01	-2.96E-02	-1.59E-01	-3.35E-02	-1.75E-01	-3.40E-02
-3.95E-02	-1.87E-02	-1.04E-01	-8.73E-02	-3.00E-02	7.53E-02	5.21E-03	-9.43E-03	6.87E-02	9.17E-02	1.22E-01	-8.50E-03	1.87E-01	9.98E-02	2.38E-01	-5.94E-03	3.01E-01	9.79E-02	-1.24E-01	-2.30E-02	-1.36E-01	-2.34E-02	-1.50E-01	-2.54E-02	-1.74E-01	-3.11E-02
-3.20E-02	-1.19E-02	-1.00E-01	-9.24E-02	-1.13E-02	7.33E-02	2.63E-04	-5.54E-03	7.48E-02	8.67E-02	1.19E-01	-6.08E-03	1.88E-01	8.87E-02	2.33E-01	-5.27E-03	2.90E-01	9.61E-02	-1.25E-01	-1.76E-02	-1.41E-01	-2.37E-02	-1.57E-01	-3.70E-02	-1.82E-01	-4.75E-02
-2.01E-02	-2.39E-02	-1.04E-01	-8.79E-02	-2.59E-02	8.26E-02	3.78E-03	-8.19E-03	7.28E-02	9.83E-02	1.25E-01	-6.72E-03	1.86E-01	9.41E-02	2.38E-01	-1.61E-03	2.93E-01	9.58E-02	-1.15E-01	-2.65E-02	-1.31E-01	-2.64E-02	-1.48E-01	-3.07E-02	-1.76E-01	-3.37E-02
-2.61E-02	-1.87E-02	-1.18E-01	-9.06E-02	-1.75E-02	6.44E-02	2.22E-03	-6.06E-03	6.48E-02	8.58E-02	1.15E-01	-6.10E-03	1.85E-01	9.48E-02	2.41E-01	-2.71E-03	2.96E-01	9.41E-02	-1.27E-01	-1.38E-02	-1.43E-01	-1.89E-02	-1.61E-01	-3.00E-02	-1.88E-01	-3.42E-02
-2.33E-02	-1.53E-02	-1.00E-01	-9.59E-02	-2.00E-02	7.93E-02	6.64E-03	-3.92E-03	6.66E-02	9.37E-02	1.26E-01	-7.04E-03	1.81E-01	8.94E-02	2.40E-01	-9.93E-04	2.90E-01	9.37E-02	-1.22E-01	-2.14E-02	-1.36E-01	-2.24E-02	-1.52E-01	-2.76E-02	-1.77E-01	-3.22E-02
-3.26E-02	-1.74E-02	-1.13E-01	-9.07E-02	-5.92E-03	8.76E-02	9.11E-03	-3.08E-03	8.51E-02	1.05E-01	1.20E-01	-4.38E-03	2.00E-01	9.88E-02	2.39E-01	9.17E-05	2.96E-01	1.02E-01	-1.35E-01	-2.67E-02	-1.48E-01	-3.31E-02	-1.61E-01	-4.18E-02	-1.83E-01	-4.62E-02
-2.20E-02	-1.54E-02	-1.08E-01	-9.48E-02	-2.16E-05	7.66E-02	3.52E-04	-6.81E-03	8.87E-02	8.97E-02	1.23E-01	-4.83E-03	1.91E-01	9.16E-02	2.36E-01	2.44E-03	2.94E-01	9.46E-02	-1.32E-01	-3.57E-03	-1.44E-01	-1.62E-02	-1.59E-01	-3.34E-02	-1.86E-01	-4.32E-02
-3.29E-02	-1.24E-02	-9.97E-02	-8.98E-02	-3.23E-02	7.71E-02	1.78E-02	-1.46E-03	7.29E-02	9.36E-02	1.24E-01	-6.22E-03	1.90E-01	9.30E-02	2.35E-01	-3.05E-03	3.03E-01	9.17E-02	-1.24E-01	-1.65E-02	-1.36E-01	-1.91E-02	-1.49E-01	-2.47E-02	-1.71E-01	-2.59E-02
-3.03E-02	-1.64E-02	-1.16E-01	-9.33E-02	-1.06E-02	6.94E-02	4.73E-03	-5.78E-03	6.44E-02	8.68E-02	1.22E-01	-7.69E-03	1.80E-01	9.13E-02	2.41E-01	-3.85E-03	2.96E-01	9.04E-02	-1.27E-01	-1.71E-02	-1.44E-01	-1.90E-02	-1.63E-01	-2.67E-02	-1.91E-01	-3.33E-02
-3.21E-02	-1.17E-02	-1.07E-01	-8.38E-02	-2.32E-02	7.17E-02	3.01E-03	-2.25E-03	7.39E-02	9.03E-02	1.24E-01	-1.22E-02	1.87E-01	9.36E-02	2.44E-01	-3.02E-03	3.00E-01	8.82E-02	-1.19E-01	-1.96E-02	-1.32E-01	-2.71E-02	-1.46E-01	-3.31E-02	-1.72E-01	-3.33E-02
-2.43E-02	-2.44E-02	-9.74E-02	-8.63E-02	-3.78E-02	8.94E-02	1.70E-04	-9.07E-03	6.92E-02	1.06E-01	1.28E-01	2.13E-04	1.96E-01	1.01E-01	2.43E-01	-4.14E-03	3.06E-01	9.11E-02	-1.12E-01	-2.48E-02	-1.26E-01	-2.65E-02	-1.41E-01	-3.42E-02	-1.66E-01	-3.83E-02
-3.09E-02	-1.77E-02	-9.99E-02	-9.11E-02	-2.44E-02	7.34E-02	1.01E-02	-1.28E-02	7.37E-02	9.53E-02	1.32E-01	-3.36E-03	1.89E-01	1.00E-01	2.33E-01	-2.37E-03	2.92E-01	1.00E-01	-1.22E-01	-2.41E-02	-1.34E-01	-2.55E-02	-1.47E-01	-2.74E-02	-1.71E-01	-2.87E-02
-3.37E-02	-1.82E-02	-1.04E-01	-8.85E-02	-2.19E-02	8.12E-02	4.49E-03	-5.17E-03	7.35E-02	9.84E-02	1.17E-01	-1.01E-03	1.92E-01	9.81E-02	2.33E-01	2.21E-03	3.12E-01	9.31E-02	-1.23E-01	-1.50E-02	-1.40E-01	-2.05E-02	-1.53E-01	-3.78E-02	-1.77E-01	-4.41E-02
-3.40E-02	-2.05E-02	-8.66E-02	-8.27E-02	-2.51E-02	7.68E-02	3.00E-03	-1.13E-02	6.00E-02	8.98E-02	1.16E-01	-8.48E-03	1.88E-01	9.07E-02	2.34E-01	-1.05E-02	2.97E-01	1.02E-01	-1.15E-01	-2.58E-02	-1.29E-01	-3.01E-02	-1.42E-01	-4.08E-02	-1.68E-01	-4.08E-02
-2.26E-02	-1.97E-02	-1.06E-01	-8.44E-02	-2.99E-02	7.57E-02	1.17E-04	-6.02E-03	6.84E-02	8.67E-02	1.19E-01	-3.19E-03	1.96E-01	9.09E-02	2.39E-01	-4.19E-03	3.11E-01	9.38E-02	-1.20E-01	-1.49E-02	-1.35E-01	-2.17E-02	-1.52E-01	-3.65E-02	-1.80E-01	-4.53E-02
-1.57E-02	-2.46E-02	-9.90E-02	-8.65E-02	-2.23E-02	7.25E-02	1.25E-02	-8.31E-03	7.67E-02	8.85E-02	1.25E-01	-4.49E-03	1.92E-01	9.39E-02	2.37E-01	-6.88E-04	2.98E-01	8.83E-02	-1.17E-01	-1.57E-02	-1.32E-01	-2.36E-02	-1.48E-01	-3.60E-02	-1.77E-01	-3.99E-02
-3.50E-02	-1.29E-02	-1.00E-01	-9.42E-02	-3.18E-02	7.79E-02	-9.63E-03	2.12E-04	7.02E-02	9.65E-02	1.16E-01	-7.07E-03	1.82E-01	9.92E-02	2.34E-01	-6.15E-03	3.03E-01	1.05E-01	-1.10E-01	-1.77E-02	-1.21E-01	-2.78E-02	-1.35E-01	-4.52E-02	-1.63E-01	-5.08E-02
-2.42E-02	-1.47E-02	-1.03E-01	-9.08E-02	-2.18E-02	8.67E-02	-9.61E-04	-4.69E-04	7.34E-02	9.83E-02	1.20E-01	-8.63E-03	1.86E-01	9.73E-02	2.35E-01	-4.86E-03	2.97E-01	1.05E-01	-1.21E-01	-2.03E-02	-1.37E-01	-2.82E-02	-1.51E-01	-4.33E-02	-1.74E-01	-4.95E-02
-3.13E-02	-1.63E-02	-9.26E-02	-8.92E-02	-2.87E-02	8.34E-02	1.13E-03	-2.06E-03	6.93E-02	1.05E-01	1.20E-01	-7.83E-03	1.85E-01	1.01E-01	2.32E-01	-7.35E-03	2.99E-01	1.02E-01	-1.12E-01	-1.56E-02	-1.26E-01	-2.42E-02	-1.41E-01	-3.77E-02	-1.70E-01	-4.50E-02
-2.53E-02	-1.03E-02	-1.02E-01	-9.86E-02	-1.74E-02	8.00E-02	2.52E-03	2.67E-03	7.00E-02	9.95E-02	1.21E-01	-7.36E-03	1.84E-01	9.58E-02	2.30E-01	-4.64E-03	2.82E-01	1.08E-01	-1.19E-01	-1.26E-02	-1.34E-01	-1.88E-02	-1.49E-01	-3.38E-02	-1.74E-01	-3.80E-02
-2.26E-02	-1.14E-02	-8.82E-02	-9.32E-02	-1.02E-02	8.19E-02	8.39E-03	1.55E-03	8.00E-02	9.58E-02	1.18E-01	1.55E-03	1.92E-01	9.32E-02	2.31E-01	-1.74E-03	2.96E-01	9.00E-02	-1.15E-01	-1.81E-02	-1.28E-01	-2.62E-02	-1.43E-01	-3.95E-02	-1.72E-01	-4.49E-02
-4.05E-02	-1.24E-02	-1.13E-01	-9.32E-02	-2.68E-02	8.27E-02	-1.66E-02	9.18E-04	6.60E-02	9.04E-02	1.16E-01	-7.75E-03	1.82E-01	9.97E-02	2.28E-01	-4.35E-03	2.95E-01	1.08E-01	-1.27E-01	-3.16E-02	-1.42E-01	-3.18E-02	-1.56E-01	-4.05E-02	-1.78E-01	-4.36E-02
-2.86E-02	-1.28E-02	-1.07E-01	-9.59E-02	-2.68E-02	8.10E-02	8.97E-04	-3.08E-03	7.00E-02	8.96E-02	1.19E-01	-5.65E-03	1.89E-01	9.48E-02	2.30E-01	-6.43E-03	3.00E-01	1.01E-01	-1.18E-01	-1.99E-02	-1.34E-01	-2.53E-02	-1.51E-01	-3.78E-02	-1.77E-01	-4.43E-02
-2.08E-02	-1.02E-02	-9.64E-02	-9.00E-02	-3.08E-02	8.85E-02	5.92E-03	3.47E-03	6.19E-02	9.84E-02	1.22E-01	-3.99E-03	1.78E-01	9.42E-02	2.32E-01	-9.73E-03	3.00E-01	1.01E-01	-1.12E-01	-6.19E-03	-1.22E-01	-1.94E-02	-1.37E-01	-3.82E-02	-1.65E-01	-4.93E-02
-3.26E-02	-9.25E-03	-9.80E-02	-1.00E-01	-1.78E-02	8.11E-02	-1.98E-04	2.10E-03	7.15E-02	9.90E-02	1.16E-01	-1.21E-03	1.82E-01	9.86E-02	2.28E-01	-9.72E-04	3.05E-01	9.52E-02	-1.16E-01	-1.92E-02	-1.30E-01	-2.91E-02	-1.47E-01	-4.50E-02	-1.78E-01	-5.27E-02
-1.67E-02	-1.50E-02	-1.04E-01	-9.75E-02	-2.13E-02	7.55E-02	9.02E-03	-1.60E-03	6.56E-02	9.61E-02	1.17E-01	-7.96E-03	1.78E-01	9.74E-02	2.32E-01	-1.76E-03	2.92E-01	9.95E-02	-1.21E-01	-1.55E-02	-1.33E-01	-2.47E-02	-1.48E-01	-3.81E-02	-1.74E-01	-4.18E-02
-2.28E-02	-1.98E-02	-9.71E-02	-9.65E-02	-2.37E-02	8.22E-02	2.73E-03	-8.21E-03	7.00E-02	9.47E-02	1.16E-01	-5.27E-03	1.83E-01	9.44E-02	2.31E-01	-4.86E-03	2.96E-01	9.93E-02	-1.23E-01	-2.84E-02	-1.36E-01	-3.00E-02	-1.51E-01	-4.03E-02	-1.77E-01	-4.41E-02
-1.63E-02	-1.09E-02	-9.99E-02	-9.42E-02	-2.11E-02	8.85E-02	4.39E-03	2.69E-03	7.52E-02	1.07E-01	1.19E-01	-1.17E-03	1.93E-01	1.01E-01	2.26E-01	-2.14E-03	2.99E-01	1.02E-01	-1.22E-01	-3.57E-02	-1.37E-01	-3.62E-02	-1.51E-01	-4.43E-02	-1.73E-01	-4.86E-02
-3.14E-02	-7.09E-03	-1.10E-01	-9.61E-02	-2.70E-02	8.09E-02	-6.80E-03	1.50E-03	6.29E-02	1.03E-01	1.13E-01	-5.78E-04	1.78E-01	1.03E-01	2.30E-01	-1.23E-03	2.95E-01	1.04E-01	-1.23E-01	-2.59E-02	-1.38E-01	-2.75E-02	-1.53E-01	-3.84E-02	-1.75E-01	-4.03E-02
-1.73E-02	-1.62E-02	-9.57E-02	-9.89E-02	-1.53E-02	8.58E-02	1.21E-02	-5.70E-03	7.70E-02	9.56E-02	1.20E-01	-7.20E-03	1.80E-01	9.16E-02	2.28E-01	-3.31E-03	2.87E-01	8.88E-02	-1.15E-01	-2.24E-03	-1.31E-01	-1.13E-02	-1.49E-01	-3.19E-02	-1.78E-01	-5.13E-02
-3.71E-02	-1.80E-02	-9.35E-02	-9.24E-02	-1.80E-02	8.35E-02	-7.40E-04	-1.62E-03	5.89E-02	9.68E-02	1.14E-01	-9.15E-03	1.82E-01	9.70E-02	2.30E-01	-7.80E-03	2.95E-01	9.89E-02	-1.21E-01	-2.07E-02	-1.32E-01	-3.17E-02	-1.45E-01	-4.58E-02	-1.72E-01	-5.03E-02
-2.39E-02	-1.71E-02	-9.42E-02	-9.44E-02	-1.05E-02	7.90E-02	2.52E-03	-9.64E-03	7.68E-02	9.27E-02	1.20E-01	-3.36E-03	1.86E-01	8.75E-02	2.27E-01	-1.14E-03	2.89E-01	9.65E-02	-1.14E-01	-1.39E-02	-1.29E-01	-2.27E-02	-1.45E-01	-4.00E-02	-1.75E-01	-4.76E-02
-2.30E-02	-1.68E-02	-1.00E-01	-8.42E-02	-2.39E-02	8.50E-02	2.34E-03	-9.28E-03	6.53E-02	9.80E-02	1.15E-01	-6.51E-03	1.85E-01	9.76E-02	2.32E-01	-6.28E-03	3.03E-01	9.66E-02	-1.12E-01	-3.23E-02	-1.27E-01	-3.41E-02	-1.44E-01	-4.12E-02	-1.73E-01	-4.43E-02
-1.87E-02	-3.39E-02	-9.10E-02	-9.38E-02	-1.15E-02	7.97E-02	-6.03E-04	-1.47E-02	8.42E-02	9.19E-02	1.23E-01	-9.27E-03	1.95E-01	9.40E-02	2.28E-01	-2.72E-03	2.91E-01	9.67E-02	-9.97E-02	-1.56E-02	-1.13E-01	-2.32E-02	-1.32E-01	-3.78E-02	-1.67E-01	-4.33E-02
-2.21E-02	-2.32E-02	-9.19E-02	-7.84E-02	-1.29E-02	7.97E-02	-5.86E-04	-7.26E-03	8.41E-02	9.40E-02	1.22E-01	-9.21E-03	1.87E-01	9.14E-02	2.32E-01	-8.41E-03	2.92E-01	9.03E-02	-1.04E-01	-2.81E-02	-1.19E-01	-3.64E-02	-1.36E-01	-4.99E-02	-1.70E-01	-5.08E-02
-2.56E-02	-2.51E-02	-8.25E-02	-8.85E-02	-2.17E-02	7.70E-02	5.99E-03	-1.17E-02	7.81E-02	9.52E-02	1.23E-01	-9.55E-03	1.95E-01	9.81E-02	2.28E-01	-9.89E-03	2.88E-01	9.80E-02	-1.01E-01	-2.85E-03	-1.12E-01	-1.33E-02	-1.29E-01	-3.30E-02	-1.62E-01	-4.99E-02
-1.70E-02	-2.59E-02	-8.76E-02	-9.24E-02	-2.13E-02	7.82E-02	4.40E-03	-1.29E-02	7.46E-02	9.52E-02	1.17E-01	-1.25E-02	1.88E-01	9.76E-02	2.27E-01	-6.15E-03	2.90E-01	9.53E-02	-1.01E-01	-1.22E-03	-1.12E-01	-1.20E-02	-1.29E-01	-3.27E-02	-1.65E-01	-4.50E-02
-1.24E-02	-2.75E-02	-8.47E-02	-9.30E-02	-1.89E-02	9.85E-02	1.19E-03	-1.06E-02	8.67E-02	1.05E-01	1.22E-01	-1.07E-02	1.90E-01	7.62E-02	2.25E-01	-4.04E-03	2.77E-01	1.05E-01	-1.08E-01	-1.71E-02	-1.20E-01	-2.46E-02	-1.37E-01	-3.95E-02	-1.70E-01	-4.61E-02
-2.50E-02	-2.81E-02	-8.44E-02	-9.58E-02	-1.60E-02	7.89E-02	-2.11E-03	-1.78E-02	7.81E-02	9.90E-02	1.22E-01	-1.05E-02	1.92E-01	9.47E-02	2.31E-01	-7.43E-03	2.95E-01	8.93E-02	-1.05E-01	-7.15E-03	-1.15E-01	-2.02E-02	-1.30E-01	-4.19E-02	-1.65E-01	-5.56E-02
-2.67E-02	-3.89E-02	-9.45E-02	-9.02E-02	-1.11E-02	8.43E-02	-1.41E-02	-2.09E-02	8.43E-02	9.72E-02	1.16E-01	-1.44E-02	1.91E-01	1.02E-01	2.30E-01	-8.07E-03	2.92E-01	9.76E-02	-1.02E-01	-4.54E-04	-1.11E-01	-1.35E-02	-1.22E-01	-4.14E-02	-1.53E-01	-5.61E-02
-2.23E-02	-2.31E-02	-9.08E-02	-9.03E-02	-2.45E-02	8.53E-02	-8.99E-04	-1.39E-02	7.75E-02	9.96E-02	1.17E-01	-1.45E-02	1.91E-01	1.02E-01	2.31E-01	-7.33E-03	2.97E-01	9.26E-02	-1.14E-01	-2.82E-02	-1.27E-01	-3.22E-02	-1.42E-01	-4.52E-02	-1.69E-01	-4.65E-02
-1.85E-02	-2.04E-02	-8.26E-02	-8.80E-02	-1.62E-02	7.91E-02	6.45E-03	-3.26E-03	7.72E-02	9.29E-02	1.25E-01	-5.85E-03	1.90E-01	9.21E-02	2.33E-01	-6.65E-03	2.90E-01	8.86E-02	-1.10E-01	-1.36E-02	-1.24E-01	-2.03E-02	-1.38E-01	-3.51E-02	-1.68E-01	-3.93E-02
-1.32E-02	-2.24E-02	-7.10E-02	-9.36E-02	-1.91E-02	8.34E-02	6.69E-03	-1.16E-02	7.28E-02	9.77E-02	1.21E-01	-1.15E-02	1.77E-01	9.16E-02	2.26E-01	-1.57E-02	2.78E-01	8.43E-02	-9.62E-02	-1.32E-02	-1.08E-01	-2.27E-02	-1.23E-01	-3.96E-02	-1.58E-01	-4.70E-02
-2.34E-02	-1.85E-02	-9.62E-02	-8.89E-02	-1.92E-02	7.69E-02	-1.65E-04	-2.41E-03	7.67E-02	9.35E-02	1.15E-01	-6.35E-03	1.98E-01	9.56E-02	2.30E-01	-4.04E-03	2.94E-01	9.56E-02	-1.11E-01	-9.64E-03	-1.26E-01	-1.82E-02	-1.42E-01	-3.47E-02	-1.72E-01	-4.39E-02
-2.31E-02	-2.03E-02	-8.24E-02	-9.65E-02	-8.46E-03	8.05E-02	7.54E-03	-1.01E-02	8.01E-02	9.58E-02	1.18E-01	-8.31E-03	1.97E-01	9.86E-02	2.33E-01	-1.01E-02	2.92E-01	1.00E-01	-1.04E-01	-7.79E-03	-1.15E-01	-1.94E-02	-1.31E-01	-4.08E-02	-1.64E-01	-5.80E-02
-2.20E-02	-2.57E-02	-8.54E-02	-9.91E-02	-2.27E-02	7.74E-02	8.15E-03	-1.35E-02	7.36E-02	9.32E-02	1.14E-01	-1.28E-02	1.97E-01	1.01E-01	2.28E-01	-1.02E-02	2.94E-01	1.02E-01	-1.10E-01	-9.75E-03	-1.21E-01	-1.87E-02	-1.36E-01	-3.94E-02	-1.68E-01	-5.17E-02
-3.44E-02	-1.77E-02	-7.86E-02	-9.57E-02	-1.63E-02	8.33E-02	4.95E-03	-7.27E-03	7.90E-02	9.88E-02	1.18E-01	-8.13E-03	1.95E-01	9.52E-02	2.33E-01	-1.32E-02	2.91E-01	9.33E-02	-1.06E-01	-1.42E-02	-1.17E-01	-2.45E-02	-1.32E-01	-4.24E-02	-1.63E-01	-5.38E-02
-1.89E-02	-2.79E-02	-7.65E-02	-8.80E-02	-2.00E-02	8.30E-02	-4.93E-03	-1.06E-02	7.62E-02	1.01E-01	1.13E-01	-2.13E-03	1.99E-01	1.02E-01	2.25E-01	-6.24E-03	3.07E-01	9.78E-02	-9.61E-02	-1.30E-02	-1.12E-01	-1.77E-02	-1.29E-01	-3.50E-02	-1.62E-01	-5.06E-02
-9.02E-03	-2.20E-02	-8.65E-02	-9.59E-02	-1.40E-02	8.03E-02	1.40E-02	-1.01E-02	8.17E-02	9.01E-02	1.18E-01	-9.19E-03	1.91E-01	8.97E-02	2.21E-01	-5.54E-03	2.86E-01	8.81E-02	-1.08E-01	-8.24E-03	-1.18E-01	-2.14E-02	-1.34E-01	-4.16E-02	-1.69E-01	-4.88E-02

-2.71E-02	-2.52E-02	-9.23E-02	-9.96E-02	-1.10E-02	8.08E-02	4.86E-03	-1.06E-02	8.40E-02	9.26E-02	1.18E-01	-5.67E-03	1.96E-01	9.22E-02	2.24E-01	-2.93E-03	2.90E-01	9.45E-02	-9.99E-02	-1.20E-02	-1.15E-01	-1.90E-02	-1.36E-01	-3.51E-02	-1.72E-01	-4.57E-02
-2.10E-02	-1.63E-02	-9.43E-02	-8.80E-02	-1.56E-02	7.60E-02	5.47E-03	-8.70E-03	7.73E-02	9.78E-02	1.22E-01	-8.99E-03	1.94E-01	9.75E-02	2.35E-01	-9.02E-03	2.92E-01	1.00E-01	-1.12E-01	-1.65E-02	-1.26E-01	-2.51E-02	-1.41E-01	-4.15E-02	-1.70E-01	-4.65E-02
-3.02E-02	-1.88E-02	-8.87E-02	-9.82E-02	-1.54E-02	8.21E-02	6.30E-03	-1.52E-02	7.38E-02	9.12E-02	1.22E-01	-1.19E-02	1.93E-01	9.46E-02	2.25E-01	-9.61E-03	2.97E-01	9.24E-02	-1.20E-01	-1.03E-02	-1.31E-01	-2.27E-02	-1.42E-01	-4.60E-02	-1.65E-01	-5.82E-02
-2.61E-02	-2.04E-02	-8.83E-02	-9.19E-02	-1.12E-02	7.82E-02	6.34E-03	-1.18E-02	8.30E-02	8.97E-02	1.20E-01	-1.17E-02	1.93E-01	9.05E-02	2.26E-01	-7.54E-03	3.00E-01	8.99E-02	-1.11E-01	-1.68E-02	-1.24E-01	-2.44E-02	-1.41E-01	-3.76E-02	-1.71E-01	-4.62E-02
-2.05E-02	-2.23E-02	-8.31E-02	-9.12E-02	-1.94E-02	8.65E-02	7.87E-03	-1.54E-02	7.23E-02	9.93E-02	1.15E-01	-1.27E-02	1.95E-01	9.70E-02	2.25E-01	-1.24E-02	2.92E-01	9.41E-02	-1.01E-01	-1.94E-02	-1.14E-01	-2.93E-02	-1.31E-01	-4.56E-02	-1.68E-01	-5.24E-02
-2.34E-02	-2.10E-02	-8.19E-02	-9.58E-02	-1.76E-02	9.08E-02	-1.43E-03	-8.23E-03	7.28E-02	1.08E-01	1.16E-01	-8.34E-03	1.90E-01	1.04E-01	2.26E-01	-9.77E-03	2.95E-01	9.76E-02	-1.10E-01	-2.35E-02	-1.21E-01	-3.33E-02	-1.35E-01	-4.84E-02	-1.65E-01	-5.27E-02
-1.98E-02	-2.79E-02	-8.25E-02	-9.65E-02	-7.81E-03	9.07E-02	1.26E-03	-1.48E-02	7.95E-02	1.05E-01	1.18E-01	-1.21E-02	1.91E-01	1.02E-01	2.25E-01	-1.02E-02	2.80E-01	9.51E-02	-1.11E-01	-2.46E-02	-1.22E-01	-3.90E-02	-1.38E-01	-5.77E-02	-1.73E-01	-6.35E-02
-2.64E-02	-2.06E-02	-9.22E-02	-9.05E-02	-1.57E-02	7.91E-02	2.64E-03	-1.21E-02	7.55E-02	9.18E-02	1.21E-01	-8.67E-03	1.87E-01	9.31E-02	2.30E-01	-4.49E-03	2.90E-01	9.38E-02	-1.11E-01	-2.19E-02	-1.23E-01	-3.31E-02	-1.39E-01	-4.72E-02	-1.71E-01	-4.88E-02
-2.07E-02	-2.79E-02	-8.93E-02	-9.26E-02	-2.15E-02	8.05E-02	3.15E-03	-1.58E-02	6.99E-02	9.73E-02	1.16E-01	-1.57E-02	1.91E-01	9.61E-02	2.30E-01	-8.54E-03	2.91E-01	8.91E-02	-1.03E-01	-1.38E-02	-1.14E-01	-2.43E-02	-1.32E-01	-4.03E-02	-1.65E-01	-4.95E-02
-1.81E-02	-2.46E-02	-8.61E-02	-9.43E-02	-1.18E-02	8.14E-02	1.13E-03	-1.04E-02	8.31E-02	1.03E-01	1.16E-01	-5.50E-03	1.96E-01	1.03E-01	2.28E-01	-6.81E-03	2.83E-01	1.02E-01	-1.03E-01	-1.83E-02	-1.19E-01	-2.71E-02	-1.37E-01	-4.57E-02	-1.69E-01	-5.70E-02
-1.77E-02	-2.77E-02	-9.65E-02	-8.16E-02	-1.76E-02	7.69E-02	4.68E-03	-1.75E-02	7.49E-02	8.92E-02	1.23E-01	-1.15E-02	1.92E-01	9.52E-02	2.34E-01	-8.24E-03	2.85E-01	9.63E-02	-1.11E-01	-1.29E-02	-1.24E-01	-2.23E-02	-1.39E-01	-4.05E-02	-1.69E-01	-4.61E-02
-3.01E-02	-2.23E-02	-8.70E-02	-9.13E-02	-1.43E-02	8.30E-02	5.74E-03	-1.06E-02	8.27E-02	9.90E-02	1.19E-01	-5.05E-03	1.92E-01	1.02E-01	2.31E-01	-4.34E-03	2.87E-01	1.01E-01	-1.17E-01	-2.11E-02	-1.26E-01	-3.32E-02	-1.40E-01	-4.79E-02	-1.70E-01	-5.20E-02
-2.54E-02	-2.81E-02	-9.01E-02	-9.08E-02	-1.95E-02	7.88E-02	-5.49E-03	-1.80E-02	7.77E-02	9.97E-02	1.20E-01	-1.24E-02	1.98E-01	9.94E-02	2.27E-01	-7.86E-03	2.92E-01	1.00E-01	-1.06E-01	5.85E-04	-1.17E-01	-1.12E-02	-1.32E-01	-3.32E-02	-1.63E-01	-4.88E-02
-1.72E-02	-2.45E-02	-8.30E-02	-9.22E-02	-2.21E-02	7.69E-02	-1.57E-04	-1.23E-02	7.80E-02	9.39E-02	1.18E-01	-1.07E-02	1.94E-01	9.43E-02	2.29E-01	-1.06E-02	2.95E-01	8.95E-02	-1.02E-01	-6.70E-03	-1.15E-01	-1.72E-02	-1.29E-01	-3.93E-02	-1.63E-01	-4.95E-02
-1.54E-02	-2.57E-02	-8.69E-02	-8.68E-02	-2.02E-02	8.21E-02	-2.62E-04	-1.28E-02	7.82E-02	9.53E-02	1.17E-01	-6.74E-03	1.96E-01	9.69E-02	2.25E-01	-9.24E-03	2.92E-01	8.81E-02	-1.00E-01	-1.49E-02	-1.11E-01	-2.65E-02	-1.28E-01	-4.35E-02	-1.63E-01	-5.26E-02
-1.10E-02	-3.18E-02	-8.92E-02	-8.89E-02	-1.84E-02	7.87E-02	1.07E-02	-1.59E-02	7.56E-02	9.37E-02	1.17E-01	-8.91E-03	1.94E-01	9.48E-02	2.26E-01	-1.28E-02	2.90E-01	9.35E-02	-8.99E-02	-7.66E-03	-1.07E-01	-1.45E-02	-1.27E-01	-3.70E-02	-1.66E-01	-5.41E-02
-1.85E-02	-2.50E-02	-8.84E-02	-9.57E-02	-1.69E-02	8.00E-02	1.06E-02	-1.31E-02	8.68E-02	9.05E-02	1.21E-01	-1.48E-02	2.01E-01	9.15E-02	2.26E-01	-6.94E-03	2.96E-01	8.88E-02	-9.91E-02	-2.18E-03	-1.12E-01	-1.10E-02	-1.27E-01	-3.20E-02	-1.61E-01	-4.03E-02
-1.64E-02	-2.21E-02	-8.68E-02	-9.12E-02	-2.23E-02	8.28E-02	7.07E-03	-1.10E-02	8.33E-02	9.20E-02	1.15E-01	-1.36E-02	1.92E-01	8.87E-02	2.25E-01	-8.40E-03	2.97E-01	9.59E-02	-1.01E-01	-7.80E-03	-1.16E-01	-1.21E-02	-1.36E-01	-2.43E-02	-1.69E-01	-3.46E-02
-2.16E-02	-2.63E-02	-1.00E-01	-9.63E-02	-2.11E-02	8.36E-02	3.89E-04	-1.65E-02	7.70E-02	9.24E-02	1.15E-01	-1.14E-02	1.93E-01	9.76E-02	2.23E-01	-7.97E-03	2.91E-01	9.12E-02	-1.04E-01	2.74E-03	-1.18E-01	-6.58E-03	-1.38E-01	-2.55E-02	-1.69E-01	-4.07E-02
-2.15E-02	-1.26E-02	-1.01E-01	-8.99E-02	-1.92E-02	8.41E-02	6.17E-03	-1.07E-03	7.82E-02	9.46E-02	1.17E-01	-8.62E-03	1.90E-01	9.66E-02	2.29E-01	-5.76E-03	2.96E-01	9.43E-02	-1.24E-01	-2.34E-02	-1.39E-01	-2.56E-02	-1.55E-01	-3.36E-02	-1.79E-01	-4.14E-02
-3.05E-02	-2.23E-02	-8.99E-02	-8.66E-02	-2.00E-02	7.50E-02	4.10E-03	-8.34E-03	7.11E-02	9.46E-02	1.16E-01	-1.25E-02	1.86E-01	9.58E-02	2.34E-01	-4.42E-03	2.93E-01	9.71E-02	-1.21E-01	-1.77E-02	-1.35E-01	-2.43E-02	-1.50E-01	-3.64E-02	-1.77E-01	-4.40E-02
-3.39E-02	-2.01E-02	-8.86E-02	-9.58E-02	-2.38E-02	7.82E-02	1.37E-02	-1.41E-02	7.19E-02	8.66E-02	1.27E-01	-1.51E-02	1.86E-01	8.57E-02	2.38E-01	-5.32E-03	2.87E-01	9.35E-02	-1.18E-01	-1.70E-02	-1.30E-01	-2.34E-02	-1.44E-01	-3.66E-02	-1.71E-01	-4.28E-02
-2.38E-02	-1.83E-02	-1.02E-01	-9.06E-02	-2.92E-02	8.11E-02	2.39E-03	-8.62E-03	6.68E-02	1.00E-01	1.22E-01	-9.19E-03	1.80E-01	1.06E-01	2.40E-01	-3.00E-03	2.90E-01	1.12E-01	-1.26E-01	-2.87E-02	-1.39E-01	-2.82E-02	-1.50E-01	-2.94E-02	-1.73E-01	-2.86E-02
-2.26E-02	-1.19E-02	-1.04E-01	-9.66E-02	-2.52E-02	8.78E-02	1.18E-02	-3.14E-03	6.88E-02	1.04E-01	1.23E-01	-3.77E-03	1.80E-01	1.07E-01	2.33E-01	1.49E-03	2.94E-01	1.05E-01	-1.23E-01	-1.61E-02	-1.36E-01	-2.56E-02	-1.51E-01	-4.03E-02	-1.77E-01	-4.87E-02
-1.92E-02	-1.34E-02	-1.06E-01	-8.41E-02	-3.30E-02	7.80E-02	1.02E-02	-5.64E-03	6.70E-02	8.73E-02	1.25E-01	-5.61E-03	1.80E-01	8.56E-02	2.45E-01	-6.39E-04	2.97E-01	9.41E-02	-1.22E-01	-1.69E-02	-1.36E-01	-2.44E-02	-1.51E-01	-3.44E-02	-1.76E-01	-3.82E-02
-4.59E-02	-1.28E-02	-9.77E-02	-8.46E-02	-2.29E-02	7.52E-02	4.89E-04	-1.32E-02	7.21E-02	9.00E-02	1.25E-01	-6.87E-03	1.93E-01	8.93E-02	2.40E-01	-5.25E-04	3.04E-01	8.85E-02	-1.24E-01	-2.46E-02	-1.36E-01	-2.66E-02	-1.49E-01	-2.99E-02	-1.73E-01	-2.76E-02
-2.72E-02	-1.21E-02	-1.01E-01	-7.84E-02	-1.64E-02	7.52E-02	5.36E-03	-3.97E-05	6.83E-02	9.23E-02	1.26E-01	-9.41E-03	1.86E-01	9.42E-02	2.42E-01	-1.82E-03	2.89E-01	1.02E-01	-1.24E-01	-3.40E-02	-1.37E-01	-3.38E-02	-1.50E-01	-3.50E-02	-1.74E-01	-2.77E-02
-3.78E-03	-1.26E-02	-9.75E-02	-8.93E-02	-1.99E-02	7.07E-02	2.58E-02	-8.63E-03	7.59E-02	8.90E-02	1.29E-01	-5.96E-03	1.87E-01	9.54E-02	2.31E-01	-6.31E-03	2.95E-01	9.36E-02	-1.24E-01	-1.85E-02	-1.39E-01	-2.00E-02	-1.55E-01	-2.58E-02	-1.81E-01	-3.41E-02
-2.11E-02	-1.98E-02	-9.71E-02	-8.38E-02	-1.72E-02	7.63E-02	1.63E-02	-1.26E-02	8.17E-02	9.20E-02	1.27E-01	-1.61E-02	1.91E-01	9.25E-02	2.27E-01	-7.85E-03	2.94E-01	9.95E-02	-1.16E-01	-1.85E-02	-1.31E-01	-2.30E-02	-1.45E-01	-3.44E-02	-1.71E-01	-3.03E-02
-2.45E-02	-1.56E-02	-8.75E-02	-1.02E-01	-3.51E-02	8.42E-02	2.21E-03	-7.36E-03	6.96E-02	1.03E-01	1.17E-01	-1.12E-02	1.89E-01	9.65E-02	2.35E-01	-6.90E-03	2.94E-01	1.01E-01	-1.26E-01	-3.95E-02	-1.39E-01	-3.96E-02	-1.52E-01	-4.48E-02	-1.77E-01	-5.11E-02
-3.91E-02	-1.01E-02	-1.10E-01	-8.71E-02	-2.06E-02	7.06E-02	7.83E-03	-1.20E-02	7.35E-02	8.40E-02	1.22E-01	-1.28E-02	1.87E-01	9.20E-02	2.35E-01	-4.82E-03	2.99E-01	1.03E-01	-1.23E-01	-1.86E-02	-1.37E-01	-2.24E-02	-1.53E-01	-3.02E-02	-1.78E-01	-3.20E-02
-1.32E-02	-1.46E-02	-1.06E-01	-8.92E-02	-3.02E-02	7.49E-02	1.39E-02	-5.82E-03	6.61E-02	8.77E-02	1.27E-01	-5.87E-03	1.86E-01	9.30E-02	2.36E-01	-8.21E-03	2.97E-01	9.07E-02	-1.23E-01	-2.26E-02	-1.36E-01	-2.54E-02	-1.50E-01	-3.19E-02	-1.74E-01	-3.02E-02
-2.89E-02	-1.43E-02	-9.15E-02	-9.46E-02	-1.50E-02	8.05E-02	1.00E-02	-3.95E-03	7.96E-02	9.88E-02	1.20E-01	-7.79E-03	1.83E-01	9.80E-02	2.27E-01	-6.09E-04	2.82E-01	1.05E-01	-1.25E-01	-1.87E-02	-1.39E-01	-2.43E-02	-1.53E-01	-3.33E-02	-1.78E-01	-4.02E-02
-6.03E-03	-1.81E-02	-9.13E-02	-9.48E-02	-2.86E-02	8.01E-02	2.09E-02	-5.88E-03	6.81E-02	1.02E-01	1.25E-01	-1.15E-02	1.73E-01	1.06E-01	2.24E-01	-8.03E-03	2.90E-01	1.05E-01	-1.13E-01	-2.06E-02	-1.28E-01	-2.51E-02	-1.44E-01	-3.71E-02	-1.74E-01	-4.14E-02
-1.47E-02	-1.43E-02	-9.17E-02	-9.48E-02	-1.92E-02	7.62E-02	2.06E-02	-7.79E-03	7.85E-02	9.73E-02	1.19E-01	-6.54E-03	1.85E-01	9.93E-02	2.31E-01	-3.31E-03	2.92E-01	1.05E-01	-1.18E-01	-1.68E-02	-1.31E-01	-2.06E-02	-1.44E-01	-3.19E-02	-1.70E-01	-3.56E-02
-2.29E-02	-2.42E-02	-1.14E-01	-8.29E-02	-2.77E-02	7.89E-02	7.65E-03	-1.53E-02	6.82E-02	9.40E-02	1.22E-01	-9.18E-03	1.86E-01	1.01E-01	2.32E-01	-4.77E-03	3.03E-01	1.02E-01	-1.29E-01	-2.77E-02	-1.40E-01	-2.74E-02	-1.50E-01	-2.97E-02	-1.68E-01	-2.52E-02
-2.73E-02	-2.13E-02	-1.07E-01	-8.33E-02	-2.47E-02	7.74E-02	1.38E-02	-7.66E-03	7.83E-02	8.95E-02	1.31E-01	-8.66E-03	1.95E-01	9.07E-02	2.54E-01	3.63E-03	3.04E-01	9.48E-02	-1.25E-01	-3.11E-02	-1.38E-01	-2.95E-02	-1.51E-01	-3.19E-02	-1.75E-01	-3.31E-02
-4.24E-02	-2.02E-02	-1.06E-01	-9.04E-02	-1.74E-02	7.24E-02	9.02E-03	-1.53E-02	7.17E-02	8.67E-02	1.25E-01	-6.55E-03	1.90E-01	9.26E-02	2.35E-01	1.97E-03	3.02E-01	1.02E-01	-1.29E-01	-2.30E-02	-1.42E-01	-2.47E-02	-1.53E-01	-2.76E-02	-1.77E-01	-2.91E-02
-2.53E-02	-1.23E-02	-9.52E-02	-9.69E-02	-1.22E-02	8.73E-02	6.84E-03	-8.93E-03	7.22E-02	1.05E-01	1.21E-01	-9.51E-03	1.88E-01	1.00E-01	2.29E-01	-1.51E-02	2.95E-01	1.04E-01	-1.15E-01	-2.02E-02	-1.29E-01	-2.96E-02	-1.45E-01	-4.59E-02	-1.74E-01	-5.34E-02
-3.85E-02	-1.42E-02	-1.03E-01	-9.71E-02	-1.74E-02	7.80E-02	5.12E-03	-6.35E-03	7.31E-02	9.41E-02	1.20E-01	-1.03E-02	1.83E-01	9.51E-02	2.42E-01	-4.80E-03	2.94E-01	1.03E-01	-1.28E-01	-2.78E-02	-1.42E-01	-3.10E-02	-1.57E-01	-3.68E-02	-1.83E-01	-4.07E-02
-1.87E-02	-1.62E-02	-8.47E-02	-9.21E-02	-1.64E-02	6.92E-02	1.36E-02	-7.14E-03	8.03E-02	8.56E-02	1.33E-01	-8.77E-03	1.89E-01	8.58E-02	2.36E-01	-2.77E-03	2.92E-01	9.10E-02	-1.15E-01	-1.73E-02	-1.27E-01	-2.48E-02	-1.40E-01	-3.65E-02	-1.67E-01	-4.14E-02
-2.90E-02	-1.33E-02	-1.04E-01	-8.35E-02	-3.39E-02	7.48E-02	1.77E-02	-1.85E-03	6.12E-02	9.54E-02	1.28E-01	-5.98E-03	1.79E-01	9.41E-02	2.39E-01	-2.02E-03	2.99E-01	1.01E-01	-1.29E-01	-4.06E-02	-1.43E-01	-4.20E-02	-1.56E-01	-5.20E-02	-1.76E-01	-5.38E-02
-2.69E-02	-7.75E-03	-9.63E-02	-9.63E-02	-1.64E-02	8.41E-02	7.18E-03	1.07E-03	8.12E-02	9.86E-02	1.21E-01	-6.41E-03	1.91E-01	9.42E-02	2.35E-01	-1.15E-03	2.99E-01	9.45E-02	-1.31E-01	-2.39E-02	-1.43E-01	-3.12E-02	-1.54E-01	-3.99E-02	-1.75E-01	-4.51E-02
-3.91E-02	-1.20E-02	-1.07E-01	-9.31E-02	-2.93E-02	7.75E-02	2.42E-03	-9.17E-03	7.34E-02	9.40E-02	1.20E-01	-7.99E-03	1.97E-01	9.51E-02	2.34E-01	-8.72E-03	3.04E-01	9.44E-02	-1.28E-01	-1.98E-02	-1.41E-01	-2.22E-02	-1.54E-01	-2.70E-02	-1.77E-01	-3.21E-02
-3.02E-02	-7.20E-03	-9.75E-02	-8.61E-02	-2.22E-02	6.75E-02	1.91E-02	-7.19E-03	7.28E-02	8.91E-02	1.29E-01	-6.22E-03	1.88E-01	8.93E-02	2.33E-01	-3.64E-03	2.95E-01	9.36E-02	-1.22E-01	-9.56E-03	-1.38E-01	-1.33E-02	-1.52E-01	-2.63E-02	-1.75E-01	-3.66E-02
-3.42E-02	-1.93E-02	-1.10E-01	-1.06E-01	-2.57E-02	6.90E-02	-4.80E-03	-1.36E-02	6.91E-02	9.47E-02	1.17E-01	-5.90E-03	1.91E-01	1.01E-01	2.30E-01	2.84E-03	2.93E-01	1.08E-01	-1.30E-01	-2.54E-02	-1.44E-01	-2.59E-02	-1.59E-01	-3.09E-02	-1.81E-01	-3.60E-02
-1.35E-02	-1.65E-02	-8.04E-02	-9.93E-02	-3.42E-02	7.65E-02	2.97E-02	-6.15E-03	5.49E-02	8.76E-02	1.31E-01	-1.06E-02	1.75E-01	8.87E-02	2.42E-01	-3.51E-03	2.79E-01	8.39E-02	-1.11E-01	-5.21E-03	-1.26E-01	-1.08E-02	-1.42E-01	-2.40E-02	-1.70E-01	-3.30E-02
-2.08E-02	-2.02E-02	-8.95E-02	-1.04E-01	-1.98E-02	8.02E-02	5.89E-03	-1.15E-02	7.17E-02	9.41E-02	1.22E-01	-1.08E-02	1.87E-01	9.30E-02	2.38E-01	-7.33E-03	2.98E-01	8.98E-02	-1.13E-01	-9.78E-03	-1.29E-01	-1.71E-02	-1.47E-01	-3.35E-02	-1.74E-01	-4.79E-02
-4.70E-02	-1.40E-02	-1.06E-01	-8.71E-02	-3.76E-02	7.89E-02	-1.23E-03	-1.36E-02	6.14E-02	9.55E-02	1.28E-01	-1.52E-02	1.86E-01	9.73E-02	2.39E-01	-8.83E-03	3.05E-01	1.02E-01	-1.31E-01	-2.78E-02	-1.42E-01	-2.48E-02	-1.53E-01	-2.83E-02	-1.73E-01	-2.96E-02
-2.48E-02	-1.31E-02	-9.76E-02	-9.77E-02	-1.90E-02	8.14E-02	9.18E-03	-8.31E-03	8.07E-02	9.29E-02	1.15E-01	-1.27E-02	1.97E-01	8.99E-02	2.29E-01	-1.14E-02	3.01E-01	9.63E-02	-1.14E-01	-2.61E-02	-1.29E-01	-3.30E-02	-1.46E-01	-4.57E-02	-1.74E-01	-5.36E-02
-2.93E-02	-1.07E-02	-9.15E-02	-1.03E-01	-8.59E-03	7.96E-02	1.05E-02	-6.43E-03	8.20E-02	9.01E-02	1.16E-01	-1.08E-02	2.01E-01	8.25E-02	2.30E-01	-6.42E-03	3.07E-01	1.03E-01	-1.12E-01	-1.60E-02	-1.27E-01	-2.49E-02	-1.45E-01	-4.32E-02	-1.78E-01	-5.24E-02
-3.02E-02	-1.51E-02	-1.05E-01	-9.41E-02	-1.45E-02	7.84E-02	3.18E-03	-1.84E-02	8.09E-02	9.53E-02	1.13E-01	-1.51E-02	1.93E-01	8.92E-02	2.32E-01	-3.08E-03	2.98E-01	1.04E-01	-1.16E-01	-1.46E-02	-1.32E-01	-2.41E-02	-1.48E-01	-4.82E-02	-1.78E-01	-6.17E-02
-1.03E-02	-9.10E-03	-9.09E-02	-9.95E-02	-1.64E-02	8.27E-02	2.40E-02	-6.14E-03	7.70E-02	9.89E-02	1.19E-01	-1.08E-02	1.91E-01	9.62E-02	2.24E-01	-1.11E-02	2.95E-01	1.04E-01	-1.03E-01	-7.79E-03	-1.21E-01	-1.44E-02	-1.41E-01	-3.52E-02	-1.72E-01	-5.64E-02
-1.68E-02	-1.49E-02	-1.02E-01	-9.55E-02	-2.03E-02	7.34E-02	1.37E-02	-1.34E-02	6.95E-02	8.74E-02	1.19E-01	-1.19E-02	1.90E-01	9.11E-02	2.30E-01	-5.18E-03	3.05E-01	9.93E-02	-1.23E-01	-1.16E-02	-1.37E-01	-1.92E-02	-1.52E-01	-3.70E-02	-1.79E-01	-4.17E-02
-3.50E-02	-1.67E-02	-1.02E-01	-9.55E-02	-1.78E-02	7.59E-02	7.28E-03	-2.55E-02	6.60E-02	9.04E-02	1.19E-01	-2.29E-02	1.87E-01	9.53E-02	2.34E-01	-1.48E-02	2.94E-01	1.04E-01	-1.25E-01	-9.38E-03	-1.36E-01	-2.08E-02	-1.49E-01	-3.99E-02	-1.76E-01	-4.41E-02
-2.59E-02	-9.43E-03	-9.14E-02	-9.17E-02	-2.11E-02	7.78E-02	1.29E-02	-1.76E-02	6.37E-02	9.15E-02	1.15E-01	-1.61E-02	1.83E-01	8.96E-02	2.30E-01	-1.08E-02	3.00E-01	1.00E-01	-1.07E-01	-1.31E-02	-1.19E-01	-2.54E-02	-1.35E-01	-4.55E-02	-1.71E-01	-5.30E-02
-7.51E-03	-1.40E-02	-1.04E-01	-9.79E-02	-2.37E-02	8.17E-02	2.06E-02	-4.67E-03	7.80E-02	9.28E-02	1.21E-01	-1.81E-02	1.98E-01	8.63E-02	2.28E-01	-9.93E-03	3.05E-01	1.02E-01	-1.13E-01	-1.77E-02	-1.31E-01	-1.88E-02	-1.52E-01	-3.03E-02	-1.81E-01	-4.16E-02
-1.25E-02	-1.74E-02	-1.08E-01	-9.07E-02	-2.13E-02	7.59E-02	1.28E-02	-7.57E-03	7.83E-02	8.79E-02	1.18E-01	-7.47E-03	1.90E-01	9.26E-02	2.26E-01	-7.22E-03	3.08E-01	1.05E-01	-1.17E-01	-1.89E-02	-1.33E-01	-2.53E-02	-1.51E-01	-3.99E-02	-1.79E-01	-4.72E-02
-3.15E-02	-1.82E-02	-9.49E-02	-7.85E-02	-1.53E-02	7.55E-02	-9.48E-04	-1.69E-02	7.44E-02	8.64E-02	1.07E-01	-2.41E-02	1.90E-01	8.19E-02	2.25E-01	-1.61E-02	3.03E-01	1.09E-01	-1.14E-01	-6.20E-03	-1.25E-01	-1.88E-02	-1.39E-01	-3.93E-02	-1.71E-01	-4.91E-02
-1.23E-02	-1.47E-02	-9.84E-02	-9.48E-02	-1.81E-02	7.79E-02	1.35E-02	-1.18E-02	7.09E-02	8.89E-02	1.19E-01	-1.12E-02	1.92E-01	8.51E-02	2.28E-01	-7.57E-03	3.06E-01	9.17E-02	-1.07E-01	-1.32E-02	-1.23E-01	-2.10E-02	-1.43E-01	-3.88E-02	-1.75E-01	-5.28E-02
-6.51E-03	-2.04E-02	-9.54E-02	-9.34E-02	-2.40E-02	8.33E-02	1.69E-02	-4.98E-03	6.72E-02	9.68E-02	1.18E-01	-1.13E-02	1.88E-01	9.17E-02	2.33E-01	-9.10E-03	3.07E-01	9.92E-02	-9.90E-02	-1.24E-02	-1.16E-01	-2.10E-02	-1.38E-01	-3.76E-02	-1.72E-01	-5.43E-02
-1.18E-02	-1.76E-02	-9.57E-02	-9.84E-02	-1.32E-02	7.67E-02	1.67E-02	-1.07E-02	7.43E-02	9.08E-02	1.19E-01	-1.22E-02	1.92E-01	9.02E-02	2.26E-01	-6.89E-03	2.85E-01	1.08E-01	-1.11E-01	-1.39E-02	-1.29E-01	-1.69E-02	-1.47E-01	-3.83E-02	-1.81E-01	-4.96E-02
-1.79E-02	-1.08E-02	-9.48E-02	-1.01E-01	-2.48E-02	7.46E-02	1.19E-02	-6.47E-03	7.25E-02	9.12E-02	1.11E-01	-1.66E-02	1.95E-01	8.62E-02	2.31E-01	-1.72E-02	3.08E-01	1.03E-01	-1.04E-01	-9.62E-03	-1.21E-01	-1.52E-02	-1.41E-01	-3.27E-02	-1.75E-01	-4.37E-02
-2.24E-02	-1.58E-02	-9.40E-02	-1.02E-01	-1.27E-02	8.65E-02	9.27E-03	-1.57E-02	7.77E-02	9.42E-02	1.11E-01	-1.78E-02	1.81E-01	9.28E-02	2.30E-01	-9.45E-03	2.93E-01	1.08E-01	-1.15E-01	-6.38E-03	-1.31E-01	-1.41E-02	-1.47E-01	-3.73E-02	-1.73E-01	-5.42E-02
-2.04E-02	-1.71E-02	-9.71E-02	-8.70E-02	-2.05E-02	7.24E-02	1.01E-02	-1.95E-02	7.17E-02	8.20E-02	1.20E-01	-1.79E-02	1.88E-01	8.37E-02	2.31E-01	-1.29E-02	2.95E-01	9.62E-02	-1.18E-01	-9.85E-03	-1.33E-01	-1.65E-02	-1.48E-01	-3.42E-02	-1.76E-01	-3.93E-02
-2.46E-02	-8.75E-03	-9.33E-02	-1.01E-01	-1.80E-02	9.19E-02	1.09E-02	-5.27E-03	6.64E-02	9.83E-02	1.15E-01	-5.73E-03	1.87E-01	9.95E-02	2.31E-01	-6.81E-03	3.11E-01	1.11E-01	-1.09E-01	-2.43E-02	-1.26E-01	-2.76E-02	-1.42E-01	-4.44E-02	-1.70E-01	-5.19E-02
-3.79E-02	-9.89E-03	-1.06E-01	-9.23E-02	-1.38E-02	8.03E-02	3.87E-03	-1.50E-02	7.31E-02	9.60E-02	1.14E-01	-1.71E-02	1.87E-01	8.98E-02	2.37E-01	-9.81E-03	2.99E-01	1.11E-01	-1.13E-01	-1.48E-02	-1.26E-01	-2.44E-02	-1.42E-01	-4.29E-02	-1.72E-01	-4.86E-02
-1.96E-02	-2.14E-02	-9.97E-02	-9.72E-02	6.14E-04	6.89E-02	7.14E-03	-1.42E-02	7.61E-02	9.47E-02	1.23E-01	-1.99E-02	1.90E-01	8.96E-02	2.33E-01	-8.40E-03	2.99E-01	1.14E-01	-1.14E-01	-1.31E-02	-1.32E-01	-1.29E-02	-1.49E-01	-2.99E-02	-1.79E-01	-4.13E-02
-1.91E-02	-1.29E-02	-1.04E-01	-9.65E-02	-1.06E-02	7.96E-02	9.23E-03	-7.58E-03	8.28E-02	9.14E-02	1.12E-01	-1.20E-02	2.03E-01	9.20E-02	2.28E-01	-1.15E-02	3.06E-01	1.03E-01	-1.14E-01	-1.13E-02	-1.31E-01	-1.75E-02	-1.52E-01	-3.48E-02	-1.81E-01	-5.25E-02
-3.18E-02	-7.98E-03	-9.06E-02	-9.29E-02	-1.58E-02	8.23E-02	1.29E-02	-1.46E-02	7.37E-02	9.06E-02	1.22E-01	-1.59E-02	1.91E-01	8.82E-02	2.29E-01	-9.75E-03	2.95E-01	9.83E-02	-1.12E-01	-6.50E-03	-1.27E-01	-1.71E-02	-1.41E-01	-4.22E-02	-1.71E-01	-5.64E-02
-3.59E-02	4.11E-03	-8.43E-02	-1.12E-01	-1.70E-02	9.27E-02	7.64E-03	-4.96E-03	7.89E-02	1.03E-01	1.10E-01	-1.03E-02	1.94E-01	9.62E-02	2.25E-01	-1.24E-02	3.01E-01	9.75E-02	-1.15E-01	-2.14E-02	-1.32E-01	-2.80E-02	-1.48E-01	-4.61E-02	-1.76E-01	-5.93E-02
-2.40E-02	-1.48E-02	-8.82E-02	-9.43E-02	-2.18E-02	7.66E-02	1.53E-02	-1.29E-02	7.74E-02	8.45E-02	1.17E-01	-1.29E-02	1.87E-01	8.33E-02	2.23E-01	-8.87E-03	3.00E-01	9.88E-02	-1.12E-01	-1.26E-02	-1.27E-01	-2.17E-02	-1.46E-01	-3.67E-02	-1.79E-01	-4.94E-02
-4.00E-02	-1.05E-02	-9.16E-02	-9.45E-02	-1.48E-02	8.27E-02	8.71E-03	-1.54E-02	7.78E-02	9.21E-02	1.20E-01	-1.53E-02	1.89E-01	8.89E-02	2.26E-01	-1.34E-02	2.97E-01	1.03E-01	-1.26E-01	-2.48E-02	-1.40E-01	-2.81E-02	-1.54E-01	-4.04E-02	-1.78E-01	-4.68E-02
-2.88E-02	-1.20E-02	-9.13E-02	-9.28E-02	-2.19E-02	8.59E-02	1.23E-02	-1.12E-02	6.76E-02	9.50E-02	1.21E-01	-1.12E-02	1.85E-01	9.05E-02	2.29E-01	-1.04E-02	2.99E-01	1.01E-01	-1.10E-01	-1.29E-02	-1.23E-01	-2.35E-02	-1.41E-01	-4.27E-02	-1.73E-01	-5.56E-02
-5.15E-02	-5.78E-03	-1.04E-01	-9.08E-02	-5.81E-03	9.30E-02	-9.73E-03	-8.41E-03	8.29E-02	1.00E-01	1.12E-01	-7.54E-03	1.96E-01	9.64E-02	2.28E-01	-1.13E-02	3.19E-01	1.06E-01	-1.31E-01	-5.20E-02	-1.40E-01	-5.42E-02	-1.51E-01	-6.01E-02	-1.77E-01	-5.78E-02
-2.54E-02	-1.31E-02	-9.61E-02	-9.55E-02	-2.08E-02	8.05E-02	5.70E-03	-1.16E-02	6.31E-02	8.67E-02	1.18E-01	-1.92E-02	1.76E-01	8.44E-02	2.35E-01	-1.20E-02	2.94E-01	9.82E-02	-1.10E-01	-1.01E-02	-1.24E-01	-1.92E-02	-1.40E-01	-3.81E-02	-1.71E-01	-4.65E-02
-3.82E-02	-1.48E-02	-1.05E-01	-9.39E-02	-1.37E-02	7.77E-02	1.45E-03	-1.66E-02	7.27E-02	9.00E-02	1.15E-01	-2.24E-02	1.84E-01	9.04E-02	2.33E-01	-1.34E-02	2.91E-01	1.08E-01	-1.19E-01	-9.53E-03	-1.31E-01	-2.10E-02	-1.44E-01	-4.26E-02	-1.73E-01	-4.80E-02
-3.30E-02	-9.47E-03	-8.46E-02	-9.50E-02	-1.48E-02	8.32E-02	3.93E-03	-8.81E-03	7.18E-02	9.41E-02	1.16E-01	-1.05E-02	1.88E-01	8.65E-02	2.29E-01	-1.31E-02	3.08E-01	1.01E-01	-1.10E-01	-1.49E-02	-1.21E-01	-2.58E-02	-1.35E-01	-4.12E-02	-1.67E-01	-4.68E-02
-2.81E-02	-1.12E-02	-9.19E-02	-9.39E-02	-1.52E-02	8.97E-02	3.04E-03	-9.84E-03	7.67E-02	9.09E-02	1.07E-01	-1.44E-02	1.82E-01	9.28E-02	2.25E-01	-1.22E-02	3.01E-01	1.04E-01	-1.17E-01	-2.31E-02	-1.29E-01	-3.47E-02	-1.45E-01	-5.34E-02	-1.78E-01	-6.05E-02
-2.60E-02	-1.51E-02	-9.48E-02	-9.43E-02	-1.30E-02	7.92E-02	1.74E-02	-1.25E-02	7.78E-02	9.35E-02	1.16E-01	-1.53E-02	1.94E-01	9.21E-02	2.29E-01	-1.04E-02	3.03E-01	1.07E-01	-1.19E-01	-1.03E-02	-1.33E-01	-1.79E-02	-1.52E-01	-3.44E-02	-1.86E-01	-4.60E-02
-3.18E-02	-1.46E-02	-8.99E-02	-9.52E-02	-1.66E-02	7.81E-02	5.20E-03	-1.40E-02	7.14E-02	8.89E-02	1.11E-01	-1.59E-02	1.95E-01	9.17E-02	2.23E-01	-1.36E-02	3.06E-01	1.09E-01	-1.14E-01	-2.68E-03	-1.27E-01	-1.32E-02	-1.44E-01	-3.04E-02	-1.74E-01	-4.56E-02
-2.19E-02	-1.98E-02	-9.24E-02	-9.18E-02	-1.59E-02	8.06E-02	1.18E-02	-1.90E-02	7.25E-02	9.43E-02	1.14E-01	-2.01E-02	1.87E-01	8.94E-02	2.29E-01	-1.28E-02	2.90E-01	1.04E-01	-1.25E-01	-4.00E-03	-1.29E-01	-2.01E-02	-1.41E-01	-4.23E-02	-1.73E-01	-5.45E-02
-4.10E-02	-1.41E-02	-8.73E-02	-9.67E-02	-2.51E-02	7.89E-02	6.35E-03	-1.52E-02	6.36E-02	8.98E-02	1.15E-01	-1.80E-02	1.85E-01	9.00E-02	2.30E-01	-1.13E-02	2.94E-01	9.86E-02	-1.22E-01	-6.20E-03	-1.32E-01	-1.67E-02	-1.45E-01	-3.46E-02	-1.74E-01	-4.47E-02

-2.60E-02	-1.28E-02	-1.00E-01	-8.70E-02	-1.79E-02	8.30E-02	5.67E-04	-8.19E-03	7.43E-02	9.58E-02	1.12E-01	-1.60E-02	1.89E-01	8.59E-02	2.27E-01	-1.16E-02	3.01E-01	9.66E-02	-1.14E-01	-1.94E-02	-1.29E-01	-2.83E-02	-1.45E-01	-4.46E-02	-1.73E-01	-5.25E-02
-3.59E-02	-1.43E-02	-9.68E-02	-8.68E-02	-2.71E-02	7.48E-02	3.08E-03	-1.63E-02	6.99E-02	8.64E-02	1.18E-01	-1.87E-02	1.87E-01	8.20E-02	2.30E-01	-1.17E-02	2.96E-01	9.53E-02	-1.25E-01	-1.79E-02	-1.37E-01	-2.53E-02	-1.52E-01	-3.69E-02	-1.79E-01	-4.27E-02
-3.32E-02	-1.27E-02	-9.41E-02	-9.00E-02	-1.97E-02	8.55E-02	2.68E-03	-1.01E-02	6.71E-02	9.35E-02	1.14E-01	-1.21E-02	1.93E-01	8.57E-02	2.31E-01	-1.50E-02	3.00E-01	8.80E-02	-1.22E-01	-8.63E-03	-1.33E-01	-1.93E-02	-1.44E-01	-4.07E-02	-1.70E-01	-5.08E-02
-3.39E-02	-2.18E-02	-1.02E-01	-8.60E-02	-2.26E-02	6.49E-02	1.10E-02	-1.60E-02	6.41E-02	8.07E-02	1.21E-01	-1.43E-02	1.92E-01	8.18E-02	2.36E-01	-9.83E-03	2.97E-01	9.73E-02	-1.18E-01	-1.26E-02	-1.31E-01	-1.86E-02	-1.48E-01	-3.29E-02	-1.77E-01	-3.87E-02
-1.98E-02	-6.36E-03	-1.02E-01	-9.46E-02	-2.85E-02	9.16E-02	1.22E-02	-3.74E-03	6.69E-02	1.04E-01	1.17E-01	-1.13E-02	1.92E-01	9.19E-02	2.31E-01	-1.67E-02	2.98E-01	9.83E-02	-1.12E-01	-1.20E-02	-1.29E-01	-2.06E-02	-1.44E-01	-4.42E-02	-1.75E-01	-5.75E-02
-3.22E-02	-2.14E-02	-1.05E-01	-8.77E-02	-2.39E-02	7.59E-02	3.33E-03	-1.62E-02	6.58E-02	8.75E-02	1.23E-01	-1.69E-02	1.83E-01	8.37E-02	2.39E-01	-1.43E-02	2.95E-01	9.69E-02	-1.18E-01	-3.75E-03	-1.31E-01	-1.24E-02	-1.47E-01	-2.84E-02	-1.75E-01	-3.63E-02
-3.52E-02	-1.50E-02	-9.72E-02	-9.07E-02	-2.51E-02	7.76E-02	5.77E-03	-1.81E-02	6.17E-02	8.76E-02	1.16E-01	-1.77E-02	1.84E-01	8.55E-02	2.36E-01	-1.08E-02	2.99E-01	1.00E-01	-1.17E-01	-7.27E-03	-1.29E-01	-1.75E-02	-1.44E-01	-3.42E-02	-1.74E-01	-4.38E-02
-3.11E-02	-2.43E-02	-1.02E-01	-8.17E-02	-2.66E-02	7.68E-02	4.24E-03	-1.57E-02	7.56E-02	8.80E-02	1.18E-01	-1.72E-02	1.88E-01	8.68E-02	2.34E-01	-1.54E-02	2.98E-01	9.72E-02	-1.15E-01	-1.46E-02	-1.28E-01	-2.42E-02	-1.44E-01	-4.07E-02	-1.73E-01	-4.50E-02
-1.83E-02	-1.21E-02	-9.06E-02	-9.33E-02	-2.41E-02	7.77E-02	1.41E-02	-8.77E-03	6.25E-02	8.62E-02	1.18E-01	-1.54E-02	1.85E-01	8.48E-02	2.25E-01	-1.10E-02	2.94E-01	1.01E-01	-1.07E-01	-1.09E-02	-1.20E-01	-2.00E-02	-1.40E-01	-3.36E-02	-1.75E-01	-4.39E-02
-3.39E-02	-1.24E-02	-1.06E-01	-9.05E-02	-1.99E-02	7.72E-02	1.06E-02	-1.25E-02	7.15E-02	8.95E-02	1.18E-01	-1.40E-02	1.84E-01	8.46E-02	2.29E-01	-7.81E-03	2.92E-01	9.73E-02	-1.20E-01	-2.01E-02	-1.35E-01	-2.54E-02	-1.53E-01	-3.75E-02	-1.80E-01	-4.78E-02
-3.43E-02	-7.60E-03	-8.34E-02	-9.62E-02	-2.19E-02	7.97E-02	1.22E-02	-1.21E-02	7.23E-02	9.22E-02	1.09E-01	-1.85E-02	1.85E-01	8.92E-02	2.22E-01	-1.48E-02	2.96E-01	9.96E-02	-1.34E-01	1.77E-02	-1.29E-01	2.49E-03	-1.32E-01	-2.84E-02	-1.53E-01	-5.63E-02
-2.53E-02	-1.51E-02	-9.55E-02	-8.95E-02	-1.25E-02	8.51E-02	9.56E-03	-1.80E-02	7.50E-02	9.19E-02	1.20E-01	-2.21E-02	1.85E-01	8.67E-02	2.26E-01	-1.72E-02	2.98E-01	9.34E-02	-1.27E-01	5.29E-03	-1.31E-01	-1.14E-02	-1.38E-01	-4.07E-02	-1.66E-01	-5.87E-02
-1.69E-02	-5.74E-03	-9.18E-02	-1.00E-01	-2.98E-02	7.86E-02	1.44E-02	-5.37E-03	6.08E-02	8.99E-02	1.15E-01	-6.25E-03	1.87E-01	8.70E-02	2.34E-01	-1.09E-02	2.92E-01	9.00E-02	-1.10E-01	-1.60E-02	-1.27E-01	-2.22E-02	-1.45E-01	-3.62E-02	-1.74E-01	-4.56E-02
-3.40E-02	-7.69E-03	-9.59E-02	-9.87E-02	-1.29E-02	7.91E-02	1.38E-02	-1.13E-02	7.84E-02	9.21E-02	1.18E-01	-1.30E-02	1.88E-01	8.51E-02	2.27E-01	-1.17E-02	2.98E-01	9.47E-02	-1.20E-01	-1.59E-02	-1.35E-01	-2.07E-02	-1.52E-01	-3.45E-02	-1.80E-01	-4.62E-02
-3.05E-02	-1.63E-02	-1.05E-01	-9.34E-02	-2.06E-02	7.77E-02	7.87E-03	-1.96E-02	6.64E-02	8.37E-02	1.18E-01	-1.47E-02	1.81E-01	8.67E-02	2.35E-01	-1.08E-02	2.97E-01	1.02E-01	-1.10E-01	-1.42E-02	-1.25E-01	-2.08E-02	-1.43E-01	-3.66E-02	-1.75E-01	-4.08E-02
-2.70E-02	-1.75E-02	-9.76E-02	-1.03E-01	-1.59E-02	8.08E-02	-3.48E-03	-1.53E-02	7.49E-02	9.66E-02	1.10E-01	-1.53E-02	1.90E-01	9.26E-02	2.22E-01	-1.13E-02	2.95E-01	1.13E-01	-1.10E-01	-1.94E-02	-1.26E-01	-2.69E-02	-1.46E-01	-4.44E-02	-1.79E-01	-5.70E-02
-3.27E-02	-1.31E-02	-8.83E-02	-9.73E-02	-2.08E-02	8.58E-02	7.68E-03	-1.37E-02	7.13E-02	9.74E-02	1.15E-01	-6.74E-03	1.89E-01	9.09E-02	2.26E-01	-7.84E-03	2.86E-01	9.52E-02	-1.23E-01	-9.26E-03	-1.34E-01	-2.09E-02	-1.45E-01	-4.51E-02	-1.71E-01	-5.37E-02
-3.11E-02	-1.24E-02	-1.03E-01	-9.35E-02	-2.86E-02	8.72E-02	2.67E-03	-7.98E-03	6.63E-02	9.94E-02	1.11E-01	-1.07E-02	1.84E-01	9.98E-02	2.27E-01	-8.40E-03	2.85E-01	9.35E-02	-1.18E-01	-1.31E-02	-1.28E-01	-2.59E-02	-1.44E-01	-4.51E-02	-1.76E-01	-5.48E-02
-2.77E-02	-1.98E-02	-1.06E-01	-9.70E-02	-1.56E-02	8.45E-02	5.41E-03	-1.57E-02	6.75E-02	9.71E-02	1.13E-01	-1.27E-02	1.83E-01	9.67E-02	2.24E-01	-6.94E-03	2.91E-01	1.09E-01	-1.15E-01	-1.19E-02	-1.30E-01	-2.18E-02	-1.47E-01	-4.11E-02	-1.80E-01	-4.81E-02
-3.62E-02	-1.12E-02	-9.74E-02	-9.65E-02	-8.29E-03	8.13E-02	2.21E-03	-1.44E-02	7.50E-02	9.79E-02	1.16E-01	-1.35E-02	1.85E-01	8.47E-02	2.31E-01	-1.01E-02	2.88E-01	1.07E-01	-1.27E-01	-8.77E-03	-1.37E-01	-2.33E-02	-1.46E-01	-5.15E-02	-1.73E-01	-6.01E-02
-3.10E-02	-1.19E-02	-1.06E-01	-9.30E-02	-1.39E-02	7.01E-02	1.11E-02	-2.11E-02	7.30E-02	8.00E-02	1.24E-01	-1.70E-02	1.86E-01	8.37E-02	2.33E-01	-1.34E-02	2.86E-01	1.06E-01	-1.35E-01	4.12E-03	-1.37E-01	-1.13E-02	-1.52E-01	-3.11E-02	-1.85E-01	-4.46E-02
-1.97E-02	-9.72E-03	-9.35E-02	-8.99E-02	-1.73E-02	7.90E-02	7.56E-03	-8.74E-03	7.10E-02	8.98E-02	1.18E-01	-9.46E-03	1.95E-01	8.64E-02	2.32E-01	-9.77E-03	2.99E-01	9.87E-02	-1.20E-01	-2.34E-02	-1.33E-01	-3.13E-02	-1.50E-01	-3.92E-02	-1.80E-01	-4.32E-02
-2.05E-02	-9.50E-03	-1.03E-01	-8.99E-02	-7.52E-03	7.98E-02	8.30E-03	-6.46E-03	9.27E-02	8.89E-02	1.17E-01	-1.06E-02	1.91E-01	8.58E-02	2.25E-01	-1.20E-02	2.92E-01	9.68E-02	-1.18E-01	-1.67E-02	-1.32E-01	-2.56E-02	-1.49E-01	-4.14E-02	-1.79E-01	-4.72E-02
-2.76E-02	-1.58E-02	-9.64E-02	-9.82E-02	-1.37E-02	7.35E-02	5.14E-03	-1.84E-02	7.19E-02	8.33E-02	1.16E-01	-1.57E-02	1.91E-01	8.41E-02	2.30E-01	-8.07E-03	2.93E-01	1.01E-01	-1.24E-01	-7.96E-04	-1.34E-01	-1.32E-02	-1.45E-01	-3.75E-02	-1.77E-01	-4.45E-02
-4.44E-02	-1.29E-02	-1.05E-01	-9.74E-02	-2.02E-02	7.64E-02	-8.48E-03	-1.25E-03	8.05E-02	8.86E-02	1.13E-01	-9.44E-03	1.94E-01	9.26E-02	2.32E-01	-1.43E-03	3.04E-01	9.80E-02	-1.22E-01	-1.71E-02	-1.36E-01	-2.55E-02	-1.52E-01	-4.06E-02	-1.79E-01	-5.00E-02
-2.09E-02	-1.46E-02	-1.06E-01	-9.26E-02	-1.94E-02	7.01E-02	2.52E-03	-4.57E-03	7.27E-02	8.39E-02	1.18E-01	-7.96E-03	2.02E-01	8.82E-02	2.34E-01	-3.04E-03	3.16E-01	9.16E-02	-1.18E-01	-2.31E-02	-1.34E-01	-2.41E-02	-1.52E-01	-3.25E-02	-1.82E-01	-3.84E-02
-3.72E-02	-1.41E-02	-1.04E-01	-9.43E-02	-1.35E-02	7.77E-02	-1.53E-03	-6.81E-03	7.83E-02	8.97E-02	1.19E-01	-8.07E-03	1.94E-01	8.91E-02	2.31E-01	-5.11E-03	3.05E-01	9.50E-02	-1.17E-01	-1.42E-02	-1.33E-01	-2.16E-02	-1.49E-01	-4.10E-02	-1.77E-01	-4.80E-02
-3.78E-02	-1.53E-02	-1.04E-01	-9.09E-02	-1.53E-02	7.97E-02	-1.93E-03	-1.10E-03	7.47E-02	9.36E-02	1.10E-01	-7.38E-03	2.00E-01	8.82E-02	2.35E-01	-9.54E-03	3.04E-01	9.78E-02	-1.14E-01	-4.69E-03	-1.29E-01	-1.53E-02	-1.45E-01	-3.62E-02	-1.73E-01	-5.07E-02
-3.91E-02	-1.51E-02	-1.07E-01	-9.49E-02	-1.73E-02	7.65E-02	-6.40E-03	-5.40E-03	7.31E-02	9.12E-02	1.08E-01	-8.83E-03	1.90E-01	8.92E-02	2.29E-01	-9.84E-03	3.02E-01	9.56E-02	-1.16E-01	2.25E-03	-1.31E-01	-9.21E-03	-1.47E-01	-3.28E-02	-1.75E-01	-5.08E-02
-2.04E-02	-1.65E-02	-9.88E-02	-9.68E-02	-1.95E-02	7.98E-02	7.26E-03	-3.85E-03	6.36E-02	8.96E-02	1.10E-01	-1.23E-02	1.85E-01	9.13E-02	2.28E-01	-8.03E-03	2.99E-01	1.05E-01	-1.16E-01	7.59E-03	-1.32E-01	-5.58E-03	-1.48E-01	-3.49E-02	-1.72E-01	-6.26E-02
-2.79E-02	-1.16E-02	-9.99E-02	-9.71E-02	-1.78E-02	7.82E-02	8.01E-03	1.88E-04	6.40E-02	9.09E-02	1.15E-01	-1.03E-02	1.88E-01	9.26E-02	2.33E-01	-1.74E-03	3.01E-01	1.03E-01	-1.15E-01	-1.47E-02	-1.31E-01	-2.41E-02	-1.48E-01	-4.21E-02	-1.80E-01	-4.99E-02
-3.60E-02	-1.47E-02	-1.08E-01	-1.01E-01	-2.06E-02	7.68E-02	5.40E-04	-5.97E-03	6.53E-02	8.55E-02	1.09E-01	-1.08E-02	1.90E-01	8.93E-02	2.33E-01	-5.72E-03	3.03E-01	1.06E-01	-1.18E-01	-1.34E-02	-1.33E-01	-2.08E-02	-1.51E-01	-3.54E-02	-1.78E-01	-4.04E-02
-2.14E-02	-1.69E-02	-1.06E-01	-9.12E-02	-2.14E-02	7.07E-02	3.67E-03	-3.32E-03	6.83E-02	8.46E-02	1.13E-01	-9.00E-03	1.89E-01	8.67E-02	2.32E-01	-1.79E-03	2.91E-01	8.46E-02	-1.14E-01	-1.45E-02	-1.32E-01	-1.90E-02	-1.55E-01	-3.13E-02	-1.86E-01	-4.28E-02
-3.27E-02	-1.49E-02	-1.02E-01	-9.44E-02	-2.75E-02	8.06E-02	1.52E-03	-1.19E-02	6.05E-02	9.42E-02	1.11E-01	-1.18E-02	1.84E-01	9.44E-02	2.32E-01	-9.74E-03	2.94E-01	1.02E-01	-1.20E-01	5.87E-03	-1.32E-01	-8.29E-03	-1.44E-01	-3.43E-02	-1.69E-01	-5.28E-02
-2.48E-02	-1.61E-02	-1.01E-01	-9.13E-02	-2.41E-02	8.09E-02	3.27E-03	-6.71E-03	5.93E-02	9.05E-02	1.15E-01	-1.43E-02	1.86E-01	8.76E-02	2.32E-01	-1.17E-02	2.94E-01	9.84E-02	-1.13E-01	-1.14E-03	-1.26E-01	-1.29E-02	-1.40E-01	-3.63E-02	-1.68E-01	-5.25E-02
-3.64E-02	-8.33E-03	-9.63E-02	-9.68E-02	-2.02E-02	7.57E-02	2.24E-03	-1.28E-03	6.71E-02	8.46E-02	1.15E-01	-7.37E-03	1.89E-01	8.31E-02	2.29E-01	-5.77E-03	3.05E-01	9.86E-02	-1.13E-01	-1.41E-02	-1.31E-01	-1.93E-02	-1.47E-01	-3.75E-02	-1.75E-01	-4.90E-02
-2.73E-02	-8.26E-03	-9.82E-02	-9.72E-02	-1.79E-02	8.46E-02	1.38E-02	-7.74E-05	6.22E-02	9.29E-02	1.13E-01	-8.46E-03	1.86E-01	9.31E-02	2.31E-01	-1.18E-02	3.00E-01	1.01E-01	-1.16E-01	1.33E-03	-1.30E-01	-1.17E-02	-1.44E-01	-3.71E-02	-1.73E-01	-5.51E-02
-3.51E-02	-1.28E-02	-1.03E-01	-9.97E-02	-1.82E-02	8.16E-02	7.01E-03	-5.97E-03	6.95E-02	9.72E-02	1.13E-01	-7.05E-03	1.89E-01	9.84E-02	2.27E-01	-1.27E-02	2.88E-01	9.18E-02	-1.12E-01	3.72E-03	-1.27E-01	-7.95E-03	-1.43E-01	-3.45E-02	-1.72E-01	-5.64E-02
-3.27E-02	-9.80E-03	-9.98E-02	-8.86E-02	-2.00E-02	6.94E-02	5.75E-03	-8.63E-03	6.70E-02	8.30E-02	1.20E-01	-1.47E-02	1.90E-01	8.76E-02	2.28E-01	-1.24E-02	2.93E-01	9.47E-02	-1.19E-01	6.21E-03	-1.31E-01	-7.73E-03	-1.40E-01	-3.41E-02	-1.64E-01	-4.99E-02
-2.63E-02	-1.31E-02	-1.11E-01	-9.33E-02	-2.25E-02	7.67E-02	1.88E-03	-2.67E-05	7.29E-02	9.29E-02	1.16E-01	-2.07E-03	1.99E-01	9.35E-02	2.34E-01	-1.48E-03	3.11E-01	9.76E-02	-1.15E-01	-1.78E-02	-1.31E-01	-2.30E-02	-1.48E-01	-3.68E-02	-1.75E-01	-4.08E-02
-3.32E-02	-1.15E-02	-1.00E-01	-9.60E-02	-2.16E-02	7.75E-02	-1.27E-03	-5.54E-03	8.00E-02	8.67E-02	1.11E-01	-1.25E-02	1.95E-01	8.70E-02	2.28E-01	-1.18E-02	3.06E-01	1.02E-01	-1.23E-01	-1.08E-03	-1.36E-01	-1.40E-02	-1.49E-01	-3.76E-02	-1.75E-01	-5.46E-02
-3.25E-02	-8.80E-03	-1.01E-01	-9.02E-02	-2.39E-02	8.19E-02	-1.06E-03	1.30E-03	7.74E-02	9.42E-02	1.11E-01	-7.45E-03	1.97E-01	9.08E-02	2.29E-01	-8.57E-03	3.02E-01	9.57E-02	-1.18E-01	-7.58E-03	-1.30E-01	-1.89E-02	-1.45E-01	-3.71E-02	-1.74E-01	-4.86E-02
-4.73E-02	-8.16E-03	-1.05E-01	-9.85E-02	-2.13E-02	7.84E-02	-3.68E-04	-4.38E-03	8.34E-02	9.78E-02	1.09E-01	-1.02E-02	1.96E-01	9.51E-02	2.22E-01	-1.07E-02	3.03E-01	1.05E-01	-1.20E-01	-1.53E-02	-1.35E-01	-2.53E-02	-1.52E-01	-4.19E-02	-1.79E-01	-5.14E-02
-4.58E-02	-8.35E-03	-1.03E-01	-8.77E-02	-2.25E-02	7.38E-02	1.48E-03	-2.57E-03	7.37E-02	8.96E-02	1.10E-01	-1.02E-02	1.98E-01	9.12E-02	2.33E-01	-9.07E-03	3.02E-01	9.82E-02	-1.20E-01	-7.72E-03	-1.37E-01	-1.48E-02	-1.51E-01	-3.41E-02	-1.76E-01	-4.61E-02
-2.10E-02	-1.59E-02	-1.01E-01	-8.72E-02	-2.24E-02	7.11E-02	6.33E-03	-1.40E-02	6.51E-02	8.15E-02	1.18E-01	-1.84E-02	1.82E-01	8.37E-02	2.33E-01	-1.04E-02	3.00E-01	1.00E-01	-1.12E-01	-6.84E-03	-1.25E-01	-1.57E-02	-1.42E-01	-3.18E-02	-1.73E-01	-4.09E-02
-2.39E-02	-1.20E-02	-8.79E-02	-9.25E-02	-2.51E-02	7.48E-02	1.09E-02	-1.13E-02	7.44E-02	8.78E-02	1.16E-01	-2.11E-02	1.89E-01	8.39E-02	2.26E-01	-1.38E-02	2.94E-01	1.04E-01	-1.16E-01	-9.00E-03	-1.31E-01	-1.60E-02	-1.42E-01	-3.63E-02	-1.70E-01	-4.02E-02
-2.78E-02	-1.85E-02	-1.11E-01	-9.27E-02	-2.20E-02	7.54E-02	5.37E-03	-1.39E-02	7.18E-02	8.75E-02	1.12E-01	-1.64E-02	1.87E-01	8.62E-02	2.30E-01	-6.92E-03	3.02E-01	1.00E-01	-1.15E-01	-1.32E-02	-1.28E-01	-2.25E-02	-1.47E-01	-3.86E-02	-1.79E-01	-4.48E-02
-2.22E-02	-1.94E-02	-1.03E-01	-9.62E-02	-2.22E-02	8.63E-02	3.22E-03	-1.57E-02	6.94E-02	1.01E-01	1.10E-01	-1.43E-02	1.90E-01	1.03E-01	2.27E-01	-1.07E-02	3.03E-01	1.18E-01	-1.12E-01	-1.45E-02	-1.26E-01	-2.52E-02	-1.45E-01	-4.80E-02	-1.80E-01	-5.94E-02
-1.53E-02	-1.82E-02	-1.09E-01	-8.51E-02	-8.11E-03	6.85E-02	1.73E-02	-1.21E-02	7.34E-02	8.57E-02	1.12E-01	-1.60E-02	1.91E-01	9.06E-02	2.32E-01	-8.09E-03	3.03E-01	1.15E-01	-1.17E-01	-2.19E-02	-1.32E-01	-3.04E-02	-1.52E-01	-4.35E-02	-1.85E-01	-5.03E-02
-2.62E-02	-1.21E-02	-9.92E-02	-9.17E-02	-1.34E-02	8.43E-02	-3.25E-03	-7.14E-03	6.97E-02	9.35E-02	1.06E-01	-1.39E-02	1.90E-01	9.19E-02	2.25E-01	-1.27E-02	2.95E-01	1.14E-01	-1.16E-01	-2.07E-02	-1.29E-01	-3.25E-02	-1.44E-01	-5.09E-02	-1.72E-01	-5.84E-02
-2.60E-02	-1.72E-02	-9.44E-02	-8.22E-02	-9.06E-03	8.32E-02	-8.49E-04	-1.39E-02	8.18E-02	8.96E-02	1.07E-01	-1.51E-02	1.91E-01	8.64E-02	2.24E-01	-9.50E-03	2.99E-01	1.06E-01	-1.15E-01	-1.31E-02	-1.29E-01	-2.45E-02	-1.43E-01	-4.91E-02	-1.74E-01	-6.10E-02
-2.88E-02	-2.01E-02	-1.06E-01	-9.00E-02	-1.90E-02	8.48E-02	-1.74E-03	-1.02E-02	7.40E-02	9.69E-02	1.09E-01	-1.34E-02	1.87E-01	9.09E-02	2.31E-01	-8.57E-03	2.97E-01	1.08E-01	-1.07E-01	-2.40E-02	-1.25E-01	-2.83E-02	-1.45E-01	-4.71E-02	-1.77E-01	-5.72E-02
-2.05E-02	-2.27E-02	-1.02E-01	-8.60E-02	-1.85E-02	8.27E-02	2.95E-03	-1.94E-02	7.26E-02	9.31E-02	1.08E-01	-2.21E-02	1.86E-01	9.40E-02	2.25E-01	-1.64E-02	3.02E-01	1.12E-01	-1.13E-01	-1.18E-02	-1.29E-01	-2.07E-02	-1.44E-01	-4.56E-02	-1.75E-01	-5.65E-02
-2.94E-02	-2.40E-02	-1.04E-01	-9.08E-02	-1.07E-02	8.03E-02	3.58E-03	-1.72E-02	7.44E-02	9.84E-02	1.03E-01	-1.74E-02	1.87E-01	9.84E-02	2.25E-01	-8.26E-03	3.01E-01	1.17E-01	-1.14E-01	-6.36E-03	-1.24E-01	-2.15E-02	-1.38E-01	-4.81E-02	-1.70E-01	-6.25E-02
-2.76E-02	-2.15E-02	-9.83E-02	-9.35E-02	-1.49E-02	7.61E-02	-3.56E-03	-1.79E-02	7.56E-02	8.81E-02	1.10E-01	-2.15E-02	1.82E-01	8.45E-02	2.24E-01	-1.10E-02	2.87E-01	1.08E-01	-1.13E-01	-9.44E-03	-1.29E-01	-1.68E-02	-1.44E-01	-3.87E-02	-1.73E-01	-4.92E-02
-2.27E-02	-1.97E-02	-1.04E-01	-8.53E-02	-1.04E-02	6.82E-02	4.69E-03	-1.70E-02	7.78E-02	8.06E-02	1.08E-01	-1.95E-02	1.90E-01	8.49E-02	2.26E-01	-7.94E-03	3.00E-01	1.14E-01	-1.17E-01	-1.47E-02	-1.33E-01	-2.25E-02	-1.52E-01	-3.74E-02	-1.83E-01	-4.71E-02
-2.50E-02	-1.95E-02	-1.02E-01	-9.39E-02	-1.47E-02	7.90E-02	1.48E-03	-1.32E-02	7.90E-02	9.15E-02	1.05E-01	-1.65E-02	1.91E-01	8.90E-02	2.23E-01	-8.35E-03	3.11E-01	1.08E-01	-1.17E-01	-1.76E-02	-1.35E-01	-2.05E-02	-1.53E-01	-3.54E-02	-1.79E-01	-4.92E-02
-3.55E-02	-2.13E-02	-1.01E-01	-9.76E-02	-1.44E-02	7.81E-02	-5.92E-03	-1.53E-02	8.40E-02	9.59E-02	9.92E-02	-1.87E-02	1.97E-01	9.04E-02	2.21E-01	-1.18E-02	3.08E-01	1.11E-01	-1.20E-01	-1.29E-02	-1.36E-01	-2.04E-02	-1.51E-01	-4.14E-02	-1.76E-01	-5.45E-02
-3.77E-02	-7.23E-03	-1.07E-01	-8.60E-02	-1.63E-02	7.85E-02	3.19E-03	-1.45E-02	8.05E-02	9.40E-02	1.04E-01	-1.28E-02	2.00E-01	9.05E-02	2.18E-01	-8.94E-03	3.08E-01	1.11E-01	-1.19E-01	-2.25E-03	-1.29E-01	-1.61E-02	-1.43E-01	-4.08E-02	-1.75E-01	-5.26E-02
-2.73E-02	-1.70E-02	-9.69E-02	-9.53E-02	-1.70E-02	8.47E-02	2.24E-03	-1.31E-02	7.32E-02	9.26E-02	1.08E-01	-1.73E-02	1.92E-01	9.14E-02	2.26E-01	-8.63E-03	3.01E-01	1.04E-01	-1.09E-01	-2.33E-02	-1.26E-01	-2.83E-02	-1.45E-01	-4.23E-02	-1.76E-01	-5.27E-02
-3.67E-02	-1.17E-02	-1.01E-01	-8.25E-02	-1.07E-02	8.30E-02	-6.36E-03	-8.09E-03	8.18E-02	9.23E-02	1.07E-01	-1.25E-02	2.01E-01	8.50E-02	2.25E-01	-1.19E-02	3.05E-01	1.10E-01	-1.22E-01	-1.23E-02	-1.36E-01	-2.14E-02	-1.49E-01	-4.12E-02	-1.76E-01	-5.15E-02
-2.84E-02	-1.43E-02	-1.06E-01	-8.87E-02	-7.83E-04	7.75E-02	-7.83E-04	-1.24E-02	8.78E-02	8.76E-02	1.07E-01	-1.34E-02	1.97E-01	9.02E-02	2.26E-01	-7.49E-03	3.02E-01	1.03E-01	-1.22E-01	-8.24E-03	-1.36E-01	-1.96E-02	-1.53E-01	-3.97E-02	-1.86E-01	-4.96E-02
-1.41E-02	-2.38E-02	-9.78E-02	-9.65E-02	-2.04E-02	7.74E-02	6.08E-03	-1.51E-02	6.90E-02	8.67E-02	1.08E-01	-1.71E-02	1.84E-01	8.98E-02	2.21E-01	-9.97E-03	2.92E-01	1.14E-01	-1.12E-01	-1.95E-02	-1.29E-01	-2.29E-02	-1.47E-01	-4.06E-02	-1.79E-01	-4.36E-02
-3.08E-02	-1.37E-02	-9.51E-02	-9.44E-02	-2.24E-02	7.18E-02	2.65E-03	-4.08E-03	6.85E-02	8.18E-02	1.12E-01	-1.38E-02	1.94E-01	8.38E-02	2.33E-01	-8.30E-03	3.02E-01	9.44E-02	-1.20E-01	-8.97E-03	-1.33E-01	-1.88E-02	-1.50E-01	-3.09E-02	-1.78E-01	-4.15E-02
-2.16E-02	-1.06E-02	-1.03E-01	-8.43E-02	-3.05E-02	7.48E-02	8.99E-03	-3.20E-03	7.59E-02	8.05E-02	1.09E-01	-1.22E-02	1.79E-01	7.68E-02	2.29E-01	-1.58E-02	2.89E-01	9.35E-02	-1.17E-01	-1.00E-02	-1.30E-01	-1.73E-02	-1.45E-01	-3.12E-02	-1.70E-01	-3.77E-02
-2.17E-02	-1.13E-02	-9.60E-02	-9.34E-02	-2.07E-02	7.52E-02	4.40E-03	-3.69E-03	7.30E-02	9.06E-02	1.13E-01	-1.33E-02	1.88E-01	8.50E-02	2.25E-01	-1.28E-02	2.99E-01	1.00E-01	-1.11E-01	-1.66E-02	-1.27E-01	-2.04E-02	-1.45E-01	-3.03E-02	-1.75E-01	-3.86E-02
-3.03E-02	-1.26E-02	-9.62E-02	-9.80E-02	-1.60E-02	7.43E-02	9.24E-03	-1.04E-02	7.04E-02	8.91E-02	1.15E-01	-1.18E-02	1.86E-01	8.49E-02	2.33E-01	-1.29E-02	2.94E-01	1.03E-01	-1.14E-01	-9.38E-03	-1.30E-01	-1.51E-02	-1.42E-01	-3.58E-02	-1.68E-01	-4.32E-02
-2.65E-02	-3.19E-03	-9.55E-02	-9.16E-02	-2.19E-02	7.91E-02	9.93E-03	7.03E-04	7.89E-02	8.76E-02	1.17E-01	-1.55E-02	2.00E-01	8.38E-02	2.35E-01	-1.55E-02	3.02E-01	9.55E-02	-1.12E-01	-1.17E-02	-1.27E-01	-1.77E-02	-1.42E-01	-3.39E-02	-1.71E-01	-4.11E-02
-4.64E-02	-6.88E-03	-1.04E-01	-8.70E-02	-1.37E-02	7.41E-02	-3.81E-04	-7.10E-03	7.16E-02	8.40E-02	1.14E-01	-2.00E-02	1.92E-01	7.79E-02	2.33E-01	-1.52E-02	2.91E-01	1.01E-01	-1.22E-01	-2.37E-03	-1.36E-01	-1.24E-02	-1.48E-01	-3.45E-02	-1.72E-01	-4.94E-02
-1.73E-02	-1.38E-02	-1.00E-01	-8.96E-02	-2.54E-02	7.28E-02	1.16E-02	-1.01E-02	6.75E-02	8.31E-02	1.16E-01	-1.46E-02	1.85E-01	8.09E-02	2.32E-01	-1.33E-02	2.95E-01	1.01E-01	-1.10E-01	-9.33E-03	-1.25E-01	-1.63E-02	-1.41E-01	-3.56E-02	-1.69E-01	-4.66E-02
-2.53E-02	-9.59E-03	-9.95E-02	-9.26E-02	-2.49E-02	8.25E-02	7.99E-04	-6.19E-03	7.47E-02	9.61E-02	1.15E-01	-1.17E-02	1.98E-01	8.38E-02	2.37E-01	-2.01E-02	3.01E-01	9.51E-02	-1.21E-01	-2.15E-02	-1.37E-01	-2.17E-02	-1.52E-01	-3.19E-02	-1.75E-01	-3.97E-02
-2.57E-02	-1.51E-02	-1.01E-01	-9.12E-02	-2.47E-02	7.33E-02	5.87E-03	-1.49E-02	6.84E-02	8.29E-02	1.10E-01	-1.64E-02	1.88E-01	8.21E-02	2.28E-01	-1.37E-02	2.97E-01	1.06E-01	-1.20E-01	-9.97E-03	-1.33E-01	-1.85E-02	-1.45E-01	-3.88E-02	-1.71E-01	-4.73E-02
-2.23E-02	-8.67E-03	-1.01E-01	-8.71E-02	-2.82E-02	8.12E-02	1.17E-02	-2.95E-03	7.45E-02	9.30E-02	1.17E-01	-8.47E-03	2.00E-01	8.35E-02	2.33E-01	-1.64E-02	3.02E-01	9.46E-02	-1.19E-01	-1.68E-02	-1.33E-01	-2.44E-02	-1.46E-01	-4.01E-02	-1.68E-01	-4.63E-02
-1.94E-02	-4.52E-04	-9.11E-02	-9.67E-02	-1.69E-02	8.11E-02	1.19E-02	3.66E-03	8.53E-02	9.26E-02	1.18E-01	-1.20E-02	1.93E-01	8.43E-02	2.29E-01	-1.04E-02	2.99E-01	8.84E-02	-1.10E-01	-1.23E-03	-1.28E-01	-6.06E-03	-1.46E-01	-2.52E-02	-1.71E-01	-4.55E-02
-2.45E-02	-4.81E-03	-8.58E-02	-8.84E-02	-2.88E-02	7.74E-02	2.44E-02	-5.35E-03	6.23E-02	7.28E-02	1.19E-01	-2.30E-02	1.88E-01	7.31E-02	2.33E-01	-1.61E-02	2.90E-01	8.78E-02	-1.15E-01	-8.46E-03	-1.30E-01	-1.39E-02	-1.43E-01	-2.93E-02	-1.69E-01	-3.74E-02
-2.07E-02	-1.36E-02	-9.44E-02	-8.52E-02	-1.58E-02	7.50E-02	7.95E-03	-5.57E-03	6.25E-02	8.39E-02	1.12E-01	-1.73E-02	1.87E-01	7.96E-02	2.30E-01	-1.61E-02	2.90E-01	9.70E-02	-1.11E-01	-1.08E-02	-1.28E-01	-1.69E-02	-1.45E-01	-3.35E-02	-1.73E-01	-4.62E-02
-2.55E-02	-8.86E-03	-8.98E-02	-8.79E-02	-1.24E-02	7.81E-02	1.16E-02	-8.34E-03	7.14E-02	8.10E-02	1.15E-01	-1.77E-02	1.90E-01	7.99E-02	2.33E-01	-1.81E-02	2.88E-01	8.92E-02	-1.09E-01	-5.36E-03	-1.26E-01	-1.15E-02	-1.43E-01	-2.87E-02	-1.74E-01	-4.11E-02
-2.48E-02	-1.51E-02	-9.12E-02	-8.59E-02	-2.63E-02	7.08E-02	9.79E-03	-1.33E-02	6.56E-02	7.70E-02	1.18E-01	-1.92E-02	1.90E-01	7.43E-02	2.29E-01	-1.72E-02	2.93E-01	9.56E-02	-1.06E-01	6.71E-04	-1.20E-01	-7.41E-03	-1.35E-01	-2.87E-02	-1.66E-01	-4.47E-02
-2.05E-02	-2.58E-03	-8.96E-02	-1.03E-01	-2.88E-02	8.14E-02	1.36E-02	4.05E-03	6.86E-02	9.13E-02	1.12E-01	-1.16E-02	1.86E-01	8.14E-02	2.30E-01	-1.44E-02	2.91E-01	8.85E-02	-1.13E-01	-1.24E-02	-1.29E-01	-1.45E-02	-1.47E-01	-2.58E-02	-1.73E-01	-3.64E-02
-4.24E-02	-9.63E-03	-9.73E-02	-8.83E-02	-8.12E-03	7.01E-02	6.00E-03	-1.06E-02	8.26E-02	7.91E-02	1.18E-01	-2.01E-02	1.90E-01	7.28E-02	2.26E-01	-2.00E-02	2.90E-01	8.86E-02	-1.10E-01	1.53E-03	-1.23E-01	-9.11E-03	-1.38E-01	-3.24E-02	-1.71E-01	-4.63E-02
-1.46E-02	-7.61E-03	-1.04E-01	-9.42E-02	-3.09E-02	7.19E-02	2.03E-02	-4.75E-03	6.69E-02	8.60E-02	1.12E-01	-1.31E-02	1.80E-01	8.07E-02	2.38E-01	-1.38E-02	2.90E-01	9.39E-02	-1.03E-01	-1.36E-02	-1.21E-01	-1.74E-02	-1.45E-01	-2.79E-02	-1.76E-01	-4.18E-02
-1.24E-02	-9.77E-03	-9.45E-02	-9.19E-02	-1.85E-02	7.08E-02	2.52E-02	-8.85E-03	7.68E-02	8.37E-02	1.13E-01	-2.09E-02	1.86E-01	8.08E-02	2.31E-01	-1.73E-02	2.95E-01	9.77E-02	-1.01E-01	-1.14E-02	-1.19E-01	-1.63E-02	-1.39E-01	-3.15E-02	-1.71E-01	-4.52E-02
-1.31E-02	-1.01E-02	-9.56E-02	-8.76E-02	-3.05E-02	7.74E-02	9.29E-03	-3.34E-03	6.18E-02	8.18E-02	1.16E-01	-1.62E-02	1.90E-01	7.76E-02	2.30E-01	-1.59E-02	2.93E-01	9.20E-02	-1.06E-01	2.47E-04	-1.23E-01	-7.41E-03	-1.39E-01	-2.91E-02	-1.67E-01	-4.45E-02

-2.51E-02	-8.30E-03	-9.28E-02	-9.06E-02	-1.79E-02	7.63E-02	9.95E-03	-6.19E-03	7.60E-02	8.28E-02	1.14E-01	-2.25E-02	1.92E-01	7.43E-02	2.32E-01	-2.16E-02	2.89E-01	9.27E-02	-1.07E-01	1.62E-03	-1.24E-01	-8.57E-03	-1.36E-01	-3.65E-02	-1.65E-01	-5.76E-02
-2.94E-02	-9.91E-03	-9.82E-02	-1.03E-01	-2.25E-02	7.73E-02	5.22E-03	-3.50E-03	6.92E-02	8.86E-02	1.14E-01	-1.34E-02	1.81E-01	8.64E-02	2.33E-01	-1.75E-02	2.94E-01	9.54E-02	-1.17E-01	-1.55E-02	-1.32E-01	-2.05E-02	-1.51E-01	-3.04E-02	-1.77E-01	-4.13E-02
-1.53E-02	-6.67E-03	-9.32E-02	-9.03E-02	-2.56E-02	7.93E-02	1.31E-02	-2.79E-03	7.23E-02	8.71E-02	1.15E-01	-1.73E-02	1.88E-01	8.15E-02	2.28E-01	-1.58E-02	2.97E-01	9.81E-02	-1.06E-01	-1.15E-03	-1.20E-01	-1.16E-02	-1.36E-01	-3.27E-02	-1.67E-01	-4.77E-02
-2.31E-02	-4.98E-03	-9.17E-02	-9.87E-02	-2.22E-02	8.14E-02	7.27E-03	-4.99E-03	7.07E-02	8.47E-02	1.16E-01	-1.72E-02	1.91E-01	8.26E-02	2.30E-01	-1.94E-02	2.90E-01	9.19E-02	-1.04E-01	1.23E-03	-1.20E-01	-7.32E-03	-1.38E-01	-2.77E-02	-1.69E-01	-4.57E-02
-2.13E-02	-8.71E-03	-9.25E-02	-8.43E-02	-2.10E-02	7.50E-02	1.28E-02	-5.72E-03	6.45E-02	8.76E-02	1.17E-01	-1.15E-02	1.90E-01	7.50E-02	2.29E-01	-1.92E-02	2.90E-01	9.16E-02	-1.15E-01	-7.26E-03	-1.29E-01	-1.53E-02	-1.46E-01	-2.70E-02	-1.75E-01	-3.71E-02
-2.34E-02	-2.66E-03	-9.98E-02	-9.48E-02	-2.44E-02	7.88E-02	1.85E-02	-5.26E-03	6.40E-02	8.36E-02	1.18E-01	-2.19E-02	1.78E-01	7.53E-02	2.31E-01	-2.18E-02	2.85E-01	1.01E-01	-1.09E-01	-5.95E-03	-1.22E-01	-1.61E-02	-1.41E-01	-3.17E-02	-1.72E-01	-4.41E-02
-1.88E-02	-5.54E-03	-9.10E-02	-8.49E-02	-2.28E-02	7.76E-02	1.35E-02	-5.59E-03	6.76E-02	8.48E-02	1.16E-01	-1.58E-02	1.89E-01	7.48E-02	2.31E-01	-1.51E-02	2.95E-01	9.01E-02	-1.04E-01	-2.29E-03	-1.15E-01	-1.61E-02	-1.30E-01	-3.84E-02	-1.59E-01	-5.47E-02
-1.54E-02	-1.06E-02	-8.42E-02	-9.13E-02	-2.64E-02	7.30E-02	2.50E-02	-5.11E-03	6.06E-02	7.82E-02	1.18E-01	-2.04E-02	1.85E-01	7.89E-02	2.31E-01	-1.46E-02	2.82E-01	9.49E-02	-1.09E-01	-3.34E-03	-1.26E-01	-1.01E-02	-1.42E-01	-2.95E-02	-1.71E-01	-4.51E-02
-1.79E-02	-4.48E-03	-9.10E-02	-8.39E-02	-2.48E-02	7.49E-02	8.81E-03	-3.07E-03	7.37E-02	8.26E-02	1.18E-01	-1.73E-02	1.87E-01	7.31E-02	2.33E-01	-1.79E-02	2.88E-01	8.42E-02	-1.16E-01	-2.53E-03	-1.29E-01	-1.26E-02	-1.42E-01	-3.16E-02	-1.71E-01	-4.19E-02
-3.64E-02	-1.67E-02	-1.01E-01	-8.63E-02	-1.89E-02	7.63E-02	8.16E-03	-1.41E-02	7.32E-02	8.60E-02	1.12E-01	-1.96E-02	1.87E-01	8.24E-02	2.33E-01	-1.57E-02	2.87E-01	9.85E-02	-1.15E-01	-1.74E-05	-1.28E-01	-1.13E-02	-1.43E-01	-3.14E-02	-1.74E-01	-4.22E-02
-2.16E-02	-1.47E-02	-9.52E-02	-9.24E-02	-1.68E-02	7.79E-02	4.40E-03	-7.64E-03	7.29E-02	8.37E-02	1.12E-01	-2.10E-02	1.93E-01	7.85E-02	2.32E-01	-1.82E-02	2.83E-01	8.92E-02	-1.18E-01	-3.36E-03	-1.30E-01	-1.46E-02	-1.45E-01	-3.62E-02	-1.74E-01	-5.07E-02
-2.93E-02	-1.35E-02	-9.77E-02	-8.42E-02	-2.17E-02	7.22E-02	7.42E-03	-1.23E-02	7.40E-02	7.92E-02	1.14E-01	-2.14E-02	1.84E-01	7.71E-02	2.30E-01	-1.50E-02	2.85E-01	8.93E-02	-1.19E-01	3.95E-05	-1.30E-01	-1.27E-02	-1.39E-01	-3.43E-02	-1.66E-01	-3.92E-02
-2.73E-02	-1.51E-02	-9.35E-02	-8.60E-02	-1.77E-02	6.61E-02	9.16E-03	-1.48E-02	7.94E-02	7.18E-02	1.20E-01	-2.38E-02	1.87E-01	6.83E-02	2.35E-01	-2.01E-02	2.78E-01	8.58E-02	-1.16E-01	1.62E-03	-1.30E-01	-1.01E-02	-1.41E-01	-3.44E-02	-1.69E-01	-4.29E-02
-2.65E-02	-1.27E-02	-9.36E-02	-8.99E-02	-2.11E-02	7.57E-02	3.11E-03	-1.33E-02	7.16E-02	8.46E-02	1.13E-01	-1.88E-02	1.87E-01	7.68E-02	2.29E-01	-1.95E-02	2.86E-01	9.20E-02	-1.11E-01	-3.68E-03	-1.28E-01	-1.17E-02	-1.44E-01	-3.40E-02	-1.74E-01	-4.98E-02
-3.90E-02	-9.20E-03	-1.07E-01	-9.74E-02	-2.10E-02	8.46E-02	1.11E-02	-1.15E-02	8.05E-02	9.86E-02	1.20E-01	-1.30E-02	1.86E-01	1.01E-01	2.32E-01	-5.21E-03	2.89E-01	1.09E-01	-1.26E-01	-7.88E-03	-1.37E-01	-1.60E-02	-1.53E-01	-2.61E-02	-1.79E-01	-3.51E-02
-3.84E-02	-1.01E-02	-9.15E-02	-8.68E-02	-1.25E-02	8.40E-02	4.61E-03	-2.80E-03	7.87E-02	9.72E-02	1.18E-01	-1.12E-02	1.91E-01	9.38E-02	2.37E-01	-9.60E-03	2.99E-01	8.80E-02	-1.26E-01	-1.76E-02	-1.36E-01	-2.63E-02	-1.46E-01	-3.70E-02	-1.67E-01	-4.12E-02
-3.10E-02	-1.28E-02	-8.85E-02	-9.74E-02	-2.39E-02	9.15E-02	-1.07E-03	3.61E-03	6.73E-02	1.05E-01	1.17E-01	-1.00E-02	1.88E-01	1.06E-01	2.33E-01	-6.74E-03	3.05E-01	9.84E-02	-1.19E-01	-4.43E-02	-1.35E-01	-4.43E-02	-1.50E-01	-5.41E-02	-1.78E-01	-6.14E-02
-2.33E-02	-1.33E-02	-8.61E-02	-9.44E-02	-3.25E-02	7.44E-02	1.87E-03	-2.91E-03	6.56E-02	8.94E-02	1.28E-01	-1.40E-02	1.79E-01	9.35E-02	2.42E-01	-6.06E-03	2.93E-01	1.01E-01	-1.14E-01	-1.33E-02	-1.28E-01	-1.79E-02	-1.44E-01	-2.99E-02	-1.70E-01	-4.11E-02
-3.64E-02	-1.31E-02	-1.05E-01	-9.67E-02	-2.45E-02	9.18E-02	3.12E-03	-2.58E-03	7.89E-02	9.66E-02	1.24E-01	-9.86E-03	1.94E-01	9.98E-02	2.35E-01	-5.55E-03	3.00E-01	8.92E-02	-1.36E-01	-2.16E-02	-1.48E-01	-2.24E-02	-1.57E-01	-3.28E-02	-1.73E-01	-3.94E-02
-4.13E-02	-1.15E-02	-9.88E-02	-9.56E-02	-7.80E-03	7.72E-02	1.38E-02	-1.19E-02	8.94E-02	8.78E-02	1.20E-01	-2.05E-02	1.88E-01	8.95E-02	2.32E-01	-1.24E-02	2.91E-01	9.47E-02	-1.37E-01	-2.39E-02	-1.51E-01	-3.05E-02	-1.64E-01	-4.47E-02	-1.86E-01	-4.95E-02
-2.31E-02	-1.67E-02	-9.50E-02	-9.45E-02	-3.72E-02	7.54E-02	6.76E-03	-5.63E-03	6.02E-02	8.68E-02	1.23E-01	-1.94E-02	1.81E-01	8.82E-02	2.36E-01	-8.04E-03	2.91E-01	9.50E-02	-1.22E-01	-2.35E-02	-1.36E-01	-2.61E-02	-1.50E-01	-3.56E-02	-1.76E-01	-4.01E-02
-3.05E-02	-1.67E-02	-9.24E-02	-9.38E-02	-9.20E-03	8.46E-02	1.62E-03	-4.37E-03	8.17E-02	9.53E-02	1.16E-01	-1.39E-02	1.90E-01	9.70E-02	2.29E-01	-4.75E-03	2.91E-01	1.00E-01	-1.22E-01	-1.64E-02	-1.37E-01	-2.23E-02	-1.52E-01	-3.53E-02	-1.78E-01	-4.39E-02
-2.13E-02	-6.97E-03	-1.08E-01	-9.27E-02	-2.26E-02	7.95E-02	2.03E-02	-7.78E-03	7.17E-02	9.93E-02	1.23E-01	-6.15E-03	1.92E-01	1.02E-01	2.39E-01	-5.02E-03	2.91E-01	1.00E-01	-1.16E-01	-1.50E-02	-1.32E-01	-2.13E-02	-1.51E-01	-3.24E-02	-1.80E-01	-3.98E-02
-1.59E-02	-1.98E-02	-9.16E-02	-8.44E-02	-2.96E-02	8.08E-02	7.62E-03	-1.12E-02	6.51E-02	9.08E-02	1.26E-01	-8.28E-03	1.86E-01	9.11E-02	2.41E-01	-8.28E-03	3.01E-01	8.63E-02	-1.13E-01	-1.89E-02	-1.26E-01	-2.43E-02	-1.42E-01	-3.35E-02	-1.70E-01	-3.71E-02
-3.09E-02	-1.55E-02	-1.12E-01	-6.71E-02	-3.36E-02	6.93E-02	-5.75E-03	-3.13E-03	7.25E-02	8.89E-02	1.18E-01	-1.02E-02	1.93E-01	9.34E-02	2.37E-01	-7.65E-03	3.09E-01	9.65E-02	-1.37E-01	-2.85E-02	-1.43E-01	-2.46E-02	-1.50E-01	-2.82E-02	-1.62E-01	-2.65E-02
-2.70E-02	-2.07E-02	-1.06E-01	-8.82E-02	-1.26E-02	8.17E-02	1.35E-02	-1.03E-02	7.99E-02	9.67E-02	1.22E-01	-1.22E-02	1.86E-01	9.09E-02	2.39E-01	-5.25E-03	3.01E-01	8.85E-02	-1.23E-01	-1.26E-02	-1.37E-01	-1.97E-02	-1.52E-01	-3.37E-02	-1.77E-01	-3.76E-02
-2.49E-02	-1.22E-02	-1.04E-01	-9.63E-02	-2.57E-02	7.58E-02	4.13E-03	-1.03E-03	7.65E-02	8.70E-02	1.13E-01	-1.07E-02	1.91E-01	8.72E-02	2.29E-01	3.42E-03	3.04E-01	8.52E-02	-1.23E-01	-1.79E-02	-1.37E-01	-2.03E-02	-1.52E-01	-3.06E-02	-1.78E-01	-3.69E-02
-3.53E-02	-6.99E-03	-9.56E-02	-9.59E-02	-1.51E-02	8.03E-02	3.79E-03	-4.21E-03	8.78E-02	8.97E-02	1.13E-01	-7.12E-03	1.92E-01	8.99E-02	2.30E-01	1.74E-04	3.03E-01	9.11E-02	-1.33E-01	-2.37E-02	-1.48E-01	-2.65E-02	-1.60E-01	-4.20E-02	-1.79E-01	-4.69E-02
-3.83E-02	-9.48E-03	-1.04E-01	-8.52E-02	-2.95E-02	7.64E-02	4.46E-03	-3.17E-03	8.13E-02	9.22E-02	1.12E-01	-2.98E-03	1.94E-01	8.76E-02	2.36E-01	-6.43E-03	3.24E-01	8.33E-02	-1.26E-01	-1.53E-02	-1.40E-01	-1.82E-02	-1.51E-01	-3.32E-02	-1.73E-01	-3.85E-02
-3.21E-02	-1.69E-02	-8.79E-02	-9.55E-02	-2.25E-02	6.99E-02	1.04E-03	-9.23E-03	7.45E-02	9.27E-02	1.27E-01	-5.49E-03	1.88E-01	9.74E-02	2.25E-01	-2.15E-03	2.89E-01	8.93E-02	-1.18E-01	-2.67E-02	-1.34E-01	-2.86E-02	-1.47E-01	-4.19E-02	-1.72E-01	-4.56E-02
-2.11E-02	-1.06E-02	-8.79E-02	-9.48E-02	-2.76E-02	7.68E-02	6.39E-03	-3.02E-03	6.17E-02	8.93E-02	1.18E-01	-3.40E-03	1.94E-01	9.00E-02	2.33E-01	-5.84E-03	2.95E-01	9.20E-02	-1.23E-01	-1.93E-02	-1.35E-01	-2.82E-02	-1.47E-01	-4.27E-02	-1.69E-01	-4.91E-02
-2.01E-02	-2.40E-02	-1.02E-01	-9.04E-02	-2.91E-02	6.89E-02	9.29E-03	-1.62E-02	6.90E-02	8.69E-02	1.28E-01	-5.31E-03	1.84E-01	9.05E-02	2.38E-01	-1.96E-03	3.02E-01	9.59E-02	-1.20E-01	-2.69E-02	-1.33E-01	-2.74E-02	-1.46E-01	-3.01E-02	-1.69E-01	-2.59E-02
-1.64E-02	-1.70E-02	-8.36E-02	-8.79E-02	-2.03E-02	7.52E-02	1.40E-02	-7.56E-03	6.68E-02	9.43E-02	1.23E-01	-1.07E-02	1.81E-01	9.23E-02	2.32E-01	-6.56E-03	2.98E-01	9.87E-02	-1.13E-01	-2.69E-02	-1.25E-01	-3.53E-02	-1.39E-01	-3.50E-02	-1.69E-01	-3.77E-02
-2.60E-02	-8.93E-03	-8.31E-02	-9.36E-02	-1.21E-02	9.08E-02	1.14E-03	2.04E-03	7.83E-02	1.04E-01	1.24E-01	-1.06E-03	1.88E-01	1.02E-01	2.31E-01	-3.85E-03	2.92E-01	1.03E-01	-1.22E-01	-3.89E-02	-1.34E-01	-4.15E-02	-1.45E-01	-4.84E-02	-1.69E-01	-5.01E-02
-4.07E-02	-2.07E-02	-7.93E-02	-9.70E-02	-1.79E-02	7.02E-02	6.11E-03	-1.14E-02	7.44E-02	8.42E-02	1.31E-01	-9.28E-03	1.82E-01	8.33E-02	2.34E-01	-3.85E-03	2.88E-01	9.13E-02	-1.30E-01	-1.70E-02	-1.43E-01	-1.93E-02	-1.55E-01	-2.66E-02	-1.80E-01	-3.44E-02
-2.56E-02	-1.60E-02	-8.65E-02	-9.89E-02	-1.37E-02	7.87E-02	2.00E-03	-3.89E-03	7.92E-02	9.35E-02	1.18E-01	-9.62E-03	1.88E-01	9.99E-02	2.33E-01	-5.19E-03	2.99E-01	1.00E-01	-1.20E-01	-1.59E-02	-1.32E-01	-2.55E-02	-1.43E-01	-4.32E-02	-1.67E-01	-4.57E-02
-2.33E-02	-2.28E-02	-1.11E-01	-8.94E-02	-3.29E-02	7.46E-02	7.52E-03	-4.52E-03	6.60E-02	9.70E-02	1.25E-01	-1.19E-02	1.78E-01	1.04E-01	2.38E-01	-4.51E-03	2.91E-01	1.12E-01	-1.21E-01	-1.73E-02	-1.34E-01	-2.22E-02	-1.47E-01	-3.11E-02	-1.70E-01	-3.23E-02
-1.55E-02	-8.33E-03	-8.89E-02	-9.23E-02	-3.21E-02	8.12E-02	1.13E-02	1.55E-04	7.53E-02	9.72E-02	1.20E-01	-4.21E-03	1.87E-01	1.07E-01	2.34E-01	-3.98E-03	3.02E-01	1.04E-01	-1.11E-01	-1.42E-02	-1.25E-01	-1.88E-02	-1.40E-01	-2.98E-02	-1.66E-01	-3.78E-02
-2.16E-02	-1.57E-02	-9.05E-02	-9.46E-02	-1.09E-02	8.72E-02	1.20E-02	-6.92E-03	7.49E-02	9.74E-02	1.21E-01	-5.23E-03	1.92E-01	9.73E-02	2.34E-01	-1.23E-02	3.01E-01	9.14E-02	-1.15E-01	-1.80E-02	-1.29E-01	-2.58E-02	-1.45E-01	-4.01E-02	-1.73E-01	-4.55E-02
-1.85E-02	-5.27E-03	-9.53E-02	-9.91E-02	-1.71E-02	9.11E-02	1.02E-02	3.02E-03	8.22E-02	1.05E-01	1.20E-01	-2.78E-03	1.90E-01	1.04E-01	2.33E-01	-2.29E-03	3.06E-01	9.93E-02	-1.15E-01	-2.87E-02	-1.29E-01	-3.60E-02	-1.46E-01	-4.75E-02	-1.76E-01	-5.17E-02
-1.33E-02	-1.83E-02	-9.19E-02	-9.16E-02	-2.51E-02	7.50E-02	9.81E-03	-6.78E-03	6.61E-02	9.23E-02	1.29E-01	-6.16E-03	1.87E-01	8.51E-02	2.58E-01	2.64E-03	2.92E-01	9.18E-02	-1.13E-01	-2.28E-02	-1.27E-01	-2.93E-02	-1.43E-01	-3.89E-02	-1.72E-01	-4.14E-02
-2.29E-02	-1.25E-02	-8.79E-02	-1.04E-01	-2.25E-02	8.41E-02	7.02E-03	-9.20E-04	6.20E-02	9.38E-02	1.25E-01	-8.30E-03	1.79E-01	9.14E-02	2.32E-01	-5.54E-03	2.99E-01	9.08E-02	-1.24E-01	-2.15E-02	-1.36E-01	-3.03E-02	-1.45E-01	-4.46E-02	-1.64E-01	-4.62E-02
-3.89E-02	-9.83E-03	-9.66E-02	-8.95E-02	-2.88E-02	7.44E-02	9.18E-03	-6.32E-03	6.92E-02	9.39E-02	1.21E-01	-1.20E-02	1.91E-01	8.99E-02	2.36E-01	-8.14E-03	2.94E-01	8.91E-02	-1.19E-01	-4.02E-03	-1.33E-01	-1.20E-02	-1.49E-01	-2.94E-02	-1.74E-01	-4.41E-02
-1.87E-02	-1.31E-02	-1.01E-01	-8.95E-02	-3.12E-02	6.37E-02	6.53E-03	3.04E-03	6.42E-02	8.96E-02	1.14E-01	-6.26E-03	1.83E-01	9.18E-02	2.37E-01	-6.02E-03	3.00E-01	9.90E-02	-1.23E-01	-1.50E-02	-1.34E-01	-1.96E-02	-1.46E-01	-2.54E-02	-1.69E-01	-2.58E-02
-2.26E-02	-1.89E-02	-9.11E-02	-6.83E-02	-4.03E-02	6.83E-02	1.21E-04	-9.98E-03	5.96E-02	9.28E-02	1.17E-01	-9.98E-03	1.94E-01	9.61E-02	2.43E-01	-8.68E-03	3.10E-01	9.61E-02	-1.14E-01	-3.91E-02	-1.24E-01	-4.24E-02	-1.36E-01	-4.62E-02	-1.60E-01	-4.04E-02
-1.45E-02	-1.15E-02	-9.59E-02	-8.23E-02	-3.08E-02	8.04E-02	1.07E-02	-4.01E-03	7.06E-02	9.27E-02	1.22E-01	-5.45E-03	1.87E-01	9.46E-02	2.33E-01	-4.10E-03	2.96E-01	9.23E-02	-1.16E-01	-2.53E-02	-1.30E-01	-2.79E-02	-1.45E-01	-3.45E-02	-1.72E-01	-3.46E-02
-3.38E-02	-4.33E-03	-8.51E-02	-9.49E-02	-1.71E-02	8.83E-02	1.43E-02	-2.65E-03	7.64E-02	9.99E-02	1.22E-01	-4.85E-03	1.88E-01	9.41E-02	2.32E-01	-6.19E-03	2.96E-01	9.12E-02	-1.27E-01	-1.29E-02	-1.37E-01	-2.61E-02	-1.46E-01	-4.74E-02	-1.68E-01	-5.90E-02
-2.82E-02	-2.26E-02	-9.52E-02	-7.77E-02	-1.44E-02	7.66E-02	2.98E-03	-1.09E-02	7.35E-02	9.70E-02	1.22E-01	-4.03E-03	1.94E-01	9.52E-02	2.35E-01	-4.42E-03	3.08E-01	9.12E-02	-1.24E-01	-2.32E-02	-1.37E-01	-2.74E-02	-1.49E-01	-3.18E-02	-1.73E-01	-3.28E-02
-1.46E-02	-1.59E-02	-8.67E-02	-8.46E-02	-2.56E-02	7.22E-02	1.32E-02	-8.63E-03	7.09E-02	8.78E-02	1.25E-01	-6.28E-03	1.96E-01	9.12E-02	2.37E-01	-2.49E-03	3.00E-01	8.90E-02	-1.14E-01	-1.57E-02	-1.28E-01	-2.32E-02	-1.43E-01	-3.82E-02	-1.73E-01	-3.89E-02
-2.73E-02	-8.49E-03	-9.76E-02	-9.45E-02	-2.41E-02	7.92E-02	1.10E-02	-2.71E-03	7.26E-02	9.52E-02	1.19E-01	-6.00E-03	1.99E-01	9.33E-02	2.37E-01	-1.95E-03	3.08E-01	9.30E-02	-1.28E-01	-1.85E-02	-1.40E-01	-2.49E-02	-1.52E-01	-3.80E-02	-1.73E-01	-4.14E-02
-2.99E-02	-7.11E-03	-9.99E-02	-9.94E-02	-1.87E-02	9.13E-02	2.49E-03	-6.32E-05	7.66E-02	1.02E-01	1.24E-01	-1.14E-02	1.89E-01	9.88E-02	2.40E-01	-8.90E-03	3.03E-01	9.72E-02	-1.24E-01	-9.70E-03	-1.39E-01	-1.57E-02	-1.55E-01	-2.97E-02	-1.78E-01	-4.05E-02
-3.34E-02	-1.30E-02	-9.65E-02	-8.29E-02	-1.44E-02	7.79E-02	3.17E-03	-2.57E-03	6.98E-02	9.74E-02	1.26E-01	-7.69E-03	1.89E-01	9.12E-02	2.37E-01	-7.54E-03	3.03E-01	8.84E-02	-1.21E-01	-1.79E-02	-1.34E-01	-2.43E-02	-1.48E-01	-3.69E-02	-1.73E-01	-4.32E-02
-2.15E-02	-2.16E-02	-1.09E-01	-9.61E-02	-3.82E-02	7.33E-02	8.30E-03	-1.05E-02	5.92E-02	8.94E-02	1.24E-01	-1.29E-02	1.74E-01	9.70E-02	2.40E-01	-2.80E-03	2.91E-01	1.08E-01	-1.20E-01	-1.52E-02	-1.32E-01	-1.78E-02	-1.46E-01	-2.24E-02	-1.70E-01	-2.51E-02
-3.51E-02	-1.09E-02	-9.94E-02	-8.14E-02	-2.81E-02	7.16E-02	2.42E-03	-5.11E-03	7.08E-02	8.45E-02	1.22E-01	-1.48E-02	1.84E-01	8.73E-02	2.36E-01	-1.88E-03	2.91E-01	1.02E-01	-1.36E-01	-9.96E-03	-1.46E-01	-1.86E-02	-1.54E-01	-2.93E-02	-1.72E-01	-3.36E-02
-3.01E-02	-1.57E-02	-9.74E-02	-9.42E-02	-2.27E-02	7.84E-02	6.75E-03	-9.79E-03	6.73E-02	9.39E-02	1.23E-01	-6.54E-03	1.89E-01	9.10E-02	2.40E-01	-6.47E-03	2.96E-01	8.61E-02	-1.31E-01	-9.63E-03	-1.42E-01	-1.91E-02	-1.53E-01	-3.68E-02	-1.74E-01	-4.66E-02
-2.05E-02	-1.24E-02	-1.08E-01	-7.88E-02	-3.31E-02	7.70E-02	7.55E-03	1.00E-03	7.01E-02	9.26E-02	1.24E-01	-8.19E-03	1.89E-01	9.17E-02	2.36E-01	-4.52E-03	3.02E-01	9.38E-02	-1.28E-01	-2.48E-02	-1.41E-01	-2.49E-02	-1.52E-01	-3.11E-02	-1.72E-01	-3.22E-02
-1.83E-02	-1.42E-02	-1.00E-01	-9.62E-02	-2.30E-02	7.65E-02	1.53E-02	-7.96E-03	6.61E-02	9.89E-02	1.28E-01	-8.64E-03	1.75E-01	9.63E-02	2.39E-01	-4.56E-03	2.85E-01	1.06E-01	-1.13E-01	-2.82E-02	-1.28E-01	-3.01E-02	-1.45E-01	-3.77E-02	-1.75E-01	-3.62E-02
-1.63E-02	-2.11E-02	-1.01E-01	-9.48E-02	-9.72E-03	7.41E-02	4.00E-03	-5.95E-03	7.66E-02	8.83E-02	1.16E-01	-6.35E-03	1.77E-01	9.09E-02	2.40E-01	8.48E-03	2.93E-01	1.03E-01	-1.28E-01	-1.04E-02	-1.43E-01	-1.69E-02	-1.54E-01	-3.30E-02	-1.75E-01	-3.64E-02
-1.20E-02	-1.10E-02	-9.12E-02	-1.02E-01	-1.22E-02	8.72E-02	1.38E-02	-3.08E-04	7.85E-02	9.80E-02	1.28E-01	-6.84E-03	1.85E-01	1.00E-01	2.36E-01	2.66E-03	2.80E-01	1.02E-01	-1.18E-01	-1.95E-02	-1.33E-01	-2.67E-02	-1.47E-01	-4.45E-02	-1.71E-01	-5.45E-02
-3.49E-02	-7.39E-03	-8.79E-02	-9.82E-02	-3.02E-02	9.12E-02	-3.11E-03	-7.76E-03	6.47E-02	1.01E-01	1.14E-01	-2.15E-02	1.93E-01	9.51E-02	2.32E-01	-1.86E-02	3.14E-01	9.62E-02	-1.20E-01	-1.58E-02	-1.32E-01	-2.41E-02	-1.43E-01	-4.40E-02	-1.66E-01	-5.38E-02
-2.42E-02	-1.72E-02	-8.37E-02	-9.50E-02	-2.19E-02	7.47E-02	6.69E-03	-1.24E-02	8.12E-02	8.55E-02	1.08E-01	-1.35E-02	1.90E-01	7.89E-02	2.28E-01	-1.28E-02	3.02E-01	8.84E-02	-1.25E-01	-7.69E-03	-1.35E-01	-1.79E-02	-1.45E-01	-3.67E-02	-1.70E-01	-4.21E-02
-3.03E-02	-1.37E-02	-9.95E-02	-8.82E-02	-1.51E-02	7.89E-02	3.51E-03	-1.50E-02	7.87E-02	9.30E-02	1.12E-01	-1.73E-02	1.88E-01	9.36E-02	2.28E-01	-1.02E-02	2.99E-01	1.05E-01	-1.29E-01	-7.27E-03	-1.39E-01	-1.85E-02	-1.53E-01	-3.68E-02	-1.83E-01	-4.66E-02
-2.28E-02	-1.31E-02	-1.05E-01	-8.29E-02	-2.48E-02	7.91E-02	6.54E-03	-6.61E-03	7.48E-02	9.12E-02	1.15E-01	-7.63E-03	1.96E-01	8.29E-02	2.32E-01	-1.30E-02	3.05E-01	9.48E-02	-1.24E-01	-1.73E-02	-1.40E-01	-1.96E-02	-1.54E-01	-3.15E-02	-1.78E-01	-3.81E-02
-3.00E-02	-1.60E-02	-9.65E-02	-8.83E-02	-2.45E-02	7.87E-02	7.57E-03	-1.71E-02	7.04E-02	8.89E-02	1.22E-01	-1.74E-02	1.89E-01	8.23E-02	2.36E-01	-1.60E-02	3.02E-01	9.78E-02	-1.19E-01	-8.97E-03	-1.34E-01	-1.14E-02	-1.48E-01	-2.75E-02	-1.76E-01	-3.79E-02
-2.38E-02	-1.48E-02	-9.93E-02	-9.11E-02	-1.81E-02	7.83E-02	1.03E-02	-8.82E-03	6.37E-02	8.89E-02	1.14E-01	-1.82E-02	1.83E-01	8.73E-02	2.28E-01	-1.23E-02	2.92E-01	9.72E-02	-1.18E-01	-8.41E-03	-1.33E-01	-1.73E-02	-1.50E-01	-3.45E-02	-1.77E-01	-4.58E-02
-3.52E-02	-1.19E-02	-9.77E-02	-8.37E-02	-2.07E-02	7.57E-02	4.44E-03	-1.06E-02	7.31E-02	8.38E-02	1.14E-01	-1.88E-02	1.91E-01	7.96E-02	2.33E-01	-1.37E-02	3.05E-01	8.80E-02	-1.29E-01	-2.09E-03	-1.37E-01	-1.35E-02	-1.48E-01	-3.32E-02	-1.69E-01	-4.69E-02
-3.44E-02	-2.04E-02	-9.88E-02	-9.40E-02	-1.94E-02	7.28E-02	3.43E-03	-1.45E-02	7.03E-02	8.51E-02	1.18E-01	-1.35E-02	1.88E-01	8.16E-02	2.32E-01	-1.45E-02	2.89E-01	9.28E-02	-1.19E-01	1.44E-03	-1.32E-01	-8.63E-03	-1.49E-01	-3.00E-02	-1.79E-01	-4.91E-02
-1.82E-02	-9.68E-03	-9.19E-02	-9.65E-02	-2.52E-02	7.78E-02	8.40E-03	-4.83E-03	7.14E-02	9.02E-02	1.23E-01	-9.74E-03	1.94E-01	8.69E-02	2.28E-01	-1.55E-02	3.04E-01	9.26E-02	-1.18E-01	-6.58E-05	-1.29E-01	-1.21E-02	-1.42E-01	-3.36E-02	-1.70E-01	-4.86E-02
-3.73E-02	-1.26E-02	-1.00E-01	-8.41E-02	-1.41E-02	7.63E-02	2.30E-03	-1.34E-02	8.19E-02	9.27E-02	1.18E-01	-1.17E-02	1.85E-01	9.10E-02	2.38E-01	-9.94E-03	3.02E-01	9.14E-02	-1.25E-01	-8.21E-03	-1.38E-01	-1.73E-02	-1.51E-01	-3.56E-02	-1.77E-01	-4.83E-02
-2.98E-02	-1.58E-02	-1.07E-01	-8.12E-02	-2.89E-02	7.59E-02	4.02E-04	-1.38E-02	6.27E-02	8.71E-02	1.17E-01	-1.66E-02	1.93E-01	8.64E-02	2.36E-01	-1.14E-02	3.05E-01	9.39E-02	-1.20E-01	-1.76E-02	-1.33E-01	-2.51E-02	-1.48E-01	-3.79E-02	-1.76E-01	-4.22E-02
-2.50E-02	-1.88E-02	-9.90E-02	-9.05E-02	-2.21E-02	8.26E-02	5.93E-03	-1.09E-02	7.08E-02	9.37E-02	1.16E-01	-1.21E-02	1.97E-01	8.45E-02	2.27E-01	-9.90E-03	3.08E-01	9.92E-02	-1.06E-01	-1.10E-02	-1.22E-01	-1.32E-02	-1.43E-01	-2.70E-02	-1.77E-01	-3.81E-02
-2.44E-02	-1.30E-02	-9.19E-02	-9.81E-02	-2.34E-02	8.67E-02	7.20E-03	-1.61E-02	6.66E-02	9.28E-02	1.16E-01	-8.44E-03	2.00E-01	9.08E-02	2.27E-01	-1.64E-02	3.03E-01	9.94E-02	-1.07E-01	-8.35E-03	-1.23E-01	-1.42E-02	-1.39E-01	-3.33E-02	-1.72E-01	-4.22E-02
-2.34E-02	-1.79E-02	-9.66E-02	-9.51E-02	-2.72E-02	8.37E-02	6.80E-03	-1.31E-02	6.43E-02	9.15E-02	1.09E-01	-1.26E-02	1.97E-01	9.26E-02	2.26E-01	-1.27E-02	3.03E-01	9.99E-02	-1.08E-01	-1.38E-02	-1.25E-01	-1.68E-02	-1.42E-01	-3.59E-02	-1.74E-01	-4.19E-02
-2.69E-02	-1.59E-02	-1.08E-01	-9.54E-02	-1.64E-02	9.04E-02	8.84E-04	-1.61E-02	6.63E-02	9.84E-02	1.13E-01	-1.57E-02	1.87E-01	9.84E-02	2.27E-01	-1.44E-02	2.97E-01	1.10E-01	-1.17E-01	-1.85E-02	-1.32E-01	-2.67E-02	-1.51E-01	-4.74E-02	-1.84E-01	-6.09E-02
-3.15E-02	-1.68E-02	-8.91E-02	-8.22E-02	-2.00E-02	7.69E-02	5.77E-03	-1.24E-02	7.63E-02	8.05E-02	1.10E-01	-1.87E-02	1.76E-01	7.93E-02	2.25E-01	-1.33E-02	2.88E-01	9.75E-02	-1.21E-01	-6.30E-03	-1.30E-01	-2.01E-02	-1.42E-01	-4.33E-02	-1.75E-01	-5.34E-02
-2.73E-02	-2.25E-02	-1.10E-01	-9.44E-02	-2.70E-02	8.12E-02	9.01E-03	-1.98E-02	6.31E-02	8.85E-02	1.17E-01	-1.67E-02	1.77E-01	8.92E-02	2.28E-01	-1.07E-02	2.90E-01	1.04E-01	-1.23E-01	-7.06E-03	-1.30E-01	-2.18E-02	-1.45E-01	-3.86E-02	-1.77E-01	-4.56E-02
-2.29E-02	-2.47E-02	-9.50E-02	-9.45E-02	-2.07E-02	7.37E-02	1.56E-02	-2.09E-02	6.82E-02	8.45E-02	1.24E-01	-1.96E-02	1.80E-01	8.96E-02	2.29E-01	-1.04E-02	2.89E-01	1.04E-01	-1.31E-01	-6.49E-03	-1.36E-01	-2.14E-02	-1.48E-01	-3.96E-02	-1.77E-01	-4.67E-02
-2.44E-02	-1.80E-02	-9.86E-02	-9.46E-02	-1.56E-02	7.90E-02	1.25E-02	-1.94E-02	7.03E-02	8.77E-02	1.21E-01	-2.07E-02	1.82E-01	8.48E-02	2.28E-01	-1.53E-02	2.93E-01	1.07E-01	-1.34E-01	1.09E-02	-1.34E-01	-5.22E-03	-1.43E-01	-3.22E-02	-1.72E-01	-5.22E-02
-3.35E-02	-6.77E-03	-9.42E-02	-9.51E-02	-1.45E-02	8.51E-02	7.37E-03	-1.42E-02	7.55E-02	9.49E-02	1.21E-01	-1.73E-02	1.87E-01	9.05E-02	2.33E-01	-1.36E-02	2.90E-01	9.96E-02	-1.21E-01	1.96E-04	-1.27E-01	-1.65E-02	-1.37E-01	-4.60E-02	-1.66E-01	-6.24E-02
-2.71E-02	-2.35E-02	-9.20E-02	-8.35E-02	-1.71E-02	7.10E-02	-3.00E-04	-1.55E-02	8.39E-02	8.29E-02	1.17E-01	-2.05E-02	1.89E-01	7.97E-02	2.32E-01	-1.57E-02	2.88E-01	9.78E-02	-1.22E-01	-6.90E-03	-1.33E-01	-1.47E-02	-1.52E-01	-2.73E-02	-1.83E-01	-4.04E-02
-2.10E-02	-1.61E-02	-8.72E-02	-9.70E-02	-1.87E-02	7.77E-02	1.22E-02	-1.75E-02	6.77E-02	9.19E-02	1.16E-01	-1.73E-02	1.86E-01	9.37E-02	2.34E-01	-1.33E-02	2.96E-01	1.00E-01	-1.13E-01	-7.95E-03	-1.26E-01	-1.70E-02	-1.41E-01	-3.57E-02	-1.74E-01	-4.49E-02
-1.41E-02	-1.59E-02	-8.99E-02	-9.27E-02	-2.42E-02	8.07E-02	1.25E-02	-1.52E-02	6.34E-02	9.04E-02	1.16E-01	-1.66E-02	1.86E-01	8.46E-02	2.32E-01	-1.61E-02	2.92E-01	9.44E-02	-1.04E-01	-1.56E-02	-1.19E-01	-2.21E-02	-1.37E-01	-3.87E-02	-1.71E-01	-4.72E-02
-2.79E-02	-1.79E-02	-9.13E-02	-9.96E-02	-1.72E-02	7.86E-02	1.96E-03	-1.65E-02	6.87E-02	9.19E-02	1.13E-01	-1.75E-02	1.91E-01	9.39E-02	2.30E-01	-1.60E-02	2.96E-01	1.04E-01	-1.09E-01	-1.35E-02	-1.22E-01	-2.32E-02	-1.39E-01	-4.33E-02	-1.75E-01	-5.62E-02

-1.80E-02	-8.86E-03	-9.56E-02	-9.22E-02	-2.38E-02	7.83E-02	8.32E-03	-1.70E-02	6.90E-02	9.17E-02	1.23E-01	-1.52E-02	1.86E-01	9.14E-02	2.33E-01	-1.26E-02	2.91E-01	9.42E-02	-1.16E-01	-1.59E-02	-1.31E-01	-2.21E-02	-1.47E-01	-3.99E-02	-1.77E-01	-4.74E-02
-2.01E-02	-1.56E-02	-8.70E-02	-9.21E-02	-2.18E-02	8.80E-02	4.84E-03	-8.52E-03	6.37E-02	9.81E-02	1.23E-01	-1.26E-02	1.82E-01	9.46E-02	2.24E-01	-1.59E-02	2.94E-01	9.92E-02	-1.09E-01	-3.11E-02	-1.24E-01	-4.05E-02	-1.40E-01	-5.94E-02	-1.69E-01	-6.51E-02
-2.24E-02	-1.15E-02	-8.75E-02	-9.65E-02	-2.65E-02	7.42E-02	1.19E-02	-1.40E-02	6.50E-02	8.33E-02	1.18E-01	-1.93E-02	1.75E-01	8.01E-02	2.32E-01	-1.90E-02	2.83E-01	9.32E-02	-1.16E-01	-4.18E-03	-1.26E-01	-1.68E-02	-1.41E-01	-3.93E-02	-1.73E-01	-5.29E-02
-1.92E-02	-1.60E-02	-7.77E-02	-9.41E-02	-2.50E-02	7.32E-02	1.54E-02	-1.69E-02	7.10E-02	8.03E-02	1.20E-01	-1.69E-02	1.88E-01	6.91E-02	2.31E-01	-1.16E-02	2.97E-01	7.92E-02	-1.20E-01	-6.00E-04	-1.32E-01	-1.06E-02	-1.44E-01	-3.44E-02	-1.75E-01	-4.77E-02
-2.20E-02	-2.03E-02	-9.70E-02	-1.06E-01	-1.74E-02	8.56E-02	8.11E-03	-1.72E-02	7.57E-02	9.45E-02	1.15E-01	-1.89E-02	1.81E-01	9.14E-02	2.21E-01	-1.19E-02	2.84E-01	1.11E-01	-1.28E-01	-1.43E-02	-1.42E-01	-2.44E-02	-1.57E-01	-5.14E-02	-1.88E-01	-5.55E-02
-1.63E-02	-2.45E-02	-9.28E-02	-8.94E-02	-1.48E-02	7.99E-02	5.45E-03	-1.45E-02	6.82E-02	9.34E-02	1.12E-01	-1.26E-02	1.81E-01	8.86E-02	2.25E-01	-1.34E-02	2.93E-01	1.05E-01	-1.23E-01	-1.90E-02	-1.36E-01	-2.81E-02	-1.54E-01	-4.74E-02	-1.90E-01	-5.51E-02
-2.27E-02	-1.44E-02	-9.12E-02	-9.28E-02	-2.63E-02	7.96E-02	3.08E-03	-1.33E-02	6.79E-02	9.14E-02	1.17E-01	-1.40E-02	1.87E-01	8.74E-02	2.38E-01	-1.06E-02	2.96E-01	1.07E-01	-1.14E-01	-1.61E-02	-1.27E-01	-2.46E-02	-1.45E-01	-4.11E-02	-1.79E-01	-4.68E-02
-1.77E-02	-1.66E-02	-9.41E-02	-9.33E-02	-2.96E-02	7.67E-02	2.52E-03	-6.32E-03	7.13E-02	8.43E-02	1.17E-01	-1.34E-02	1.89E-01	8.00E-02	2.38E-01	-1.21E-02	2.90E-01	8.77E-02	-1.12E-01	-1.73E-02	-1.27E-01	-2.61E-02	-1.42E-01	-4.69E-02	-1.74E-01	-5.50E-02
-2.33E-02	-1.96E-02	-9.96E-02	-9.31E-02	-2.31E-02	7.91E-02	6.65E-03	-2.91E-02	7.03E-02	8.98E-02	1.21E-01	-1.93E-02	1.77E-01	9.11E-02	2.33E-01	-1.27E-02	2.84E-01	1.05E-01	-1.14E-01	-2.88E-02	-1.27E-01	-3.58E-02	-1.46E-01	-4.79E-02	-1.79E-01	-4.96E-02
-1.30E-02	-1.83E-02	-9.46E-02	-9.37E-02	-2.18E-02	7.45E-02	9.20E-03	-1.41E-02	6.70E-02	9.07E-02	1.12E-01	-1.47E-02	1.85E-01	8.36E-02	2.31E-01	-7.00E-03	2.98E-01	1.05E-01	-1.17E-01	-7.06E-03	-1.29E-01	-1.73E-02	-1.43E-01	-4.10E-02	-1.74E-01	-5.45E-02
-1.92E-02	-1.95E-02	-9.64E-02	-1.01E-01	-3.03E-02	8.29E-02	1.78E-02	-1.66E-02	6.03E-02	9.38E-02	1.16E-01	-1.88E-02	1.80E-01	8.79E-02	2.38E-01	-1.08E-02	2.95E-01	9.65E-02	-1.16E-01	-1.31E-02	-1.30E-01	-2.37E-02	-1.41E-01	-5.15E-02	-1.69E-01	-6.24E-02
-3.50E-02	-1.04E-02	-8.53E-02	-9.59E-02	-1.14E-02	8.76E-02	8.39E-03	-1.09E-02	7.36E-02	9.54E-02	1.10E-01	-1.61E-02	1.87E-01	8.44E-02	2.28E-01	-1.70E-02	2.97E-01	9.57E-02	-1.20E-01	-1.06E-02	-1.31E-01	-2.32E-02	-1.43E-01	-4.59E-02	-1.72E-01	-5.84E-02
-1.50E-02	-2.12E-02	-9.83E-02	-9.51E-02	-1.47E-02	7.86E-02	6.38E-03	-1.25E-02	7.08E-02	9.35E-02	1.12E-01	-1.66E-02	1.87E-01	8.92E-02	2.30E-01	-1.62E-02	2.90E-01	1.01E-01	-1.09E-01	-1.60E-02	-1.26E-01	-2.17E-02	-1.44E-01	-3.87E-02	-1.78E-01	-4.37E-02
-2.52E-02	-1.40E-02	-9.37E-02	-9.09E-02	-1.70E-02	7.58E-02	1.26E-02	-1.31E-02	6.97E-02	8.68E-02	1.16E-01	-1.63E-02	1.81E-01	8.35E-02	2.25E-01	-2.08E-02	2.87E-01	9.23E-02	-1.24E-01	-2.27E-04	-1.31E-01	-1.41E-02	-1.43E-01	-3.74E-02	-1.74E-01	-5.11E-02
-3.39E-02	-1.93E-02	-8.95E-02	-8.99E-02	-2.43E-02	7.64E-02	4.15E-03	-1.94E-02	6.88E-02	8.29E-02	1.12E-01	-2.09E-02	1.86E-01	8.45E-02	2.32E-01	-1.83E-02	3.05E-01	9.86E-02	-1.25E-01	-2.43E-03	-1.34E-01	-1.89E-02	-1.39E-01	-5.06E-02	-1.64E-01	-5.91E-02
-2.28E-02	-2.34E-02	-8.27E-02	-9.46E-02	-1.59E-02	7.68E-02	9.11E-03	-2.03E-02	6.60E-02	8.94E-02	1.13E-01	-2.33E-02	1.82E-01	8.96E-02	2.34E-01	-1.52E-02	2.77E-01	9.93E-02	-1.13E-01	-1.13E-02	-1.25E-01	-2.23E-02	-1.42E-01	-4.33E-02	-1.79E-01	-5.40E-02
-2.66E-02	-7.47E-03	-8.36E-02	-9.88E-02	-1.74E-02	7.76E-02	1.11E-02	-4.78E-03	6.80E-02	8.39E-02	1.11E-01	-1.03E-02	1.79E-01	7.92E-02	2.25E-01	-1.38E-02	2.84E-01	9.28E-02	-1.14E-01	4.89E-03	-1.25E-01	-8.65E-03	-1.34E-01	-3.92E-02	-1.63E-01	-6.11E-02
-3.04E-02	-1.32E-02	-8.02E-02	-8.91E-02	-2.08E-02	7.12E-02	1.46E-02	-1.45E-02	6.62E-02	7.29E-02	1.16E-01	-2.25E-02	1.81E-01	7.02E-02	2.38E-01	-1.76E-02	2.82E-01	8.18E-02	-1.23E-01	6.01E-03	-1.24E-01	-1.14E-02	-1.29E-01	-4.06E-02	-1.58E-01	-5.79E-02
-2.67E-02	-8.56E-03	-9.55E-02	-9.01E-02	-1.57E-02	7.28E-02	1.71E-02	-1.14E-02	6.58E-02	8.12E-02	1.13E-01	-1.62E-02	1.76E-01	8.11E-02	2.28E-01	-1.54E-02	2.93E-01	9.97E-02	-1.05E-01	4.85E-03	-1.21E-01	-5.48E-03	-1.38E-01	-3.28E-02	-1.67E-01	-5.80E-02
-1.82E-02	-1.74E-02	-8.69E-02	-9.64E-02	-2.48E-02	8.16E-02	1.42E-02	-8.44E-03	6.45E-02	8.82E-02	1.14E-01	-1.35E-02	1.79E-01	8.47E-02	2.27E-01	-1.27E-02	2.79E-01	8.70E-02	-1.08E-01	-8.70E-03	-1.24E-01	-1.85E-02	-1.37E-01	-4.58E-02	-1.68E-01	-6.09E-02
-2.73E-02	-1.38E-02	-9.62E-02	-9.77E-02	-1.96E-02	8.15E-02	8.56E-03	-1.08E-02	6.35E-02	9.17E-02	1.15E-01	-1.36E-02	1.82E-01	9.42E-02	2.24E-01	-9.65E-03	2.80E-01	9.84E-02	-1.10E-01	-6.46E-03	-1.25E-01	-1.78E-02	-1.39E-01	-4.72E-02	-1.77E-01	-5.69E-02
-1.10E-02	-2.38E-02	-8.01E-02	-9.29E-02	-2.05E-02	7.59E-02	1.33E-02	-1.12E-02	6.40E-02	8.42E-02	1.14E-01	-1.68E-02	1.81E-01	7.44E-02	2.29E-01	-6.89E-03	2.86E-01	8.88E-02	-1.05E-01	-1.40E-02	-1.21E-01	-2.22E-02	-1.38E-01	-3.76E-02	-1.70E-01	-4.69E-02
-3.76E-02	-7.98E-03	-8.90E-02	-9.66E-02	-2.07E-02	8.25E-02	7.66E-03	-1.33E-02	7.42E-02	8.68E-02	1.17E-01	-2.64E-02	1.75E-01	8.42E-02	2.31E-01	-1.27E-02	2.79E-01	1.02E-01	-1.09E-01	-2.27E-04	-1.19E-01	-1.40E-02	-1.32E-01	-3.57E-02	-1.64E-01	-4.78E-02
-2.70E-02	-1.21E-02	-9.14E-02	-9.73E-02	-1.86E-02	7.78E-02	7.11E-03	-1.46E-02	7.88E-02	8.18E-02	1.16E-01	-2.13E-02	1.79E-01	8.04E-02	2.28E-01	-1.50E-02	2.81E-01	9.80E-02	-1.21E-01	2.94E-03	-1.31E-01	-1.30E-02	-1.40E-01	-4.22E-02	-1.67E-01	-5.65E-02
-4.02E-02	-8.18E-03	-8.96E-02	-9.33E-02	-2.56E-02	7.91E-02	1.58E-02	-1.61E-02	6.46E-02	7.59E-02	1.17E-01	-2.50E-02	1.77E-01	7.35E-02	2.29E-01	-1.65E-02	2.88E-01	9.22E-02	-1.15E-01	2.17E-03	-1.24E-01	-1.14E-02	-1.39E-01	-3.30E-02	-1.72E-01	-4.59E-02
-3.13E-02	-1.41E-02	-9.30E-02	-9.33E-02	-2.52E-02	8.04E-02	7.23E-03	-1.30E-02	6.44E-02	9.38E-02	1.17E-01	-1.69E-02	1.74E-01	8.85E-02	2.36E-01	-9.45E-03	2.85E-01	9.71E-02	-1.09E-01	-1.62E-02	-1.23E-01	-2.44E-02	-1.42E-01	-3.95E-02	-1.77E-01	-5.02E-02
-3.35E-02	-1.38E-02	-9.12E-02	-8.96E-02	-2.20E-02	8.15E-02	6.26E-03	-1.14E-02	7.00E-02	8.48E-02	1.17E-01	-1.28E-02	1.85E-01	8.04E-02	2.33E-01	-1.86E-02	2.94E-01	9.31E-02	-1.16E-01	-3.86E-03	-1.27E-01	-1.68E-02	-1.37E-01	-4.47E-02	-1.66E-01	-6.02E-02
-1.91E-02	-2.38E-02	-8.78E-02	-8.59E-02	-1.83E-02	7.70E-02	1.08E-02	-1.50E-02	6.11E-02	7.89E-02	1.14E-01	-2.36E-02	1.85E-01	7.75E-02	2.29E-01	-1.40E-02	2.98E-01	9.70E-02	-1.03E-01	-4.45E-03	-1.16E-01	-1.59E-02	-1.33E-01	-3.80E-02	-1.69E-01	-5.03E-02
-1.70E-02	-1.49E-02	-9.00E-02	-9.38E-02	-2.36E-02	8.09E-02	1.54E-02	-6.64E-03	6.54E-02	8.26E-02	1.14E-01	-1.69E-02	1.81E-01	8.33E-02	2.30E-01	-8.94E-03	2.88E-01	1.00E-01	-1.08E-01	-1.87E-02	-1.23E-01	-2.65E-02	-1.41E-01	-4.35E-02	-1.74E-01	-5.18E-02
-2.87E-02	-1.27E-02	-8.57E-02	-9.70E-02	-1.92E-02	7.75E-02	1.14E-02	-1.36E-02	5.91E-02	8.05E-02	1.19E-01	-2.07E-02	1.81E-01	8.05E-02	2.30E-01	-1.24E-02	2.92E-01	9.83E-02	-9.93E-02	-5.03E-03	-1.15E-01	-1.40E-02	-1.33E-01	-3.52E-02	-1.71E-01	-4.84E-02
-2.44E-02	-1.37E-02	-9.77E-02	-1.03E-01	-1.92E-02	8.19E-02	7.05E-03	-1.41E-02	6.61E-02	8.54E-02	1.15E-01	-1.69E-02	1.87E-01	8.77E-02	2.29E-01	-9.24E-03	2.89E-01	9.95E-02	-1.05E-01	-2.20E-03	-1.19E-01	-1.33E-02	-1.38E-01	-3.65E-02	-1.71E-01	-5.28E-02
-2.46E-02	-1.12E-02	-8.73E-02	-9.99E-02	-2.21E-02	7.69E-02	1.20E-02	-1.01E-02	6.45E-02	8.53E-02	1.16E-01	-1.71E-02	1.82E-01	8.31E-02	2.23E-01	-1.73E-02	2.80E-01	1.01E-01	-1.01E-01	-3.94E-03	-1.14E-01	-1.67E-02	-1.30E-01	-4.22E-02	-1.66E-01	-5.90E-02
-1.46E-02	-2.07E-02	-8.31E-02	-9.51E-02	-2.00E-02	7.68E-02	1.18E-02	-9.69E-03	7.01E-02	8.29E-02	1.13E-01	-2.04E-02	1.95E-01	8.25E-02	2.30E-01	-1.21E-02	2.95E-01	9.40E-02	-1.11E-01	-1.06E-02	-1.28E-01	-1.70E-02	-1.42E-01	-3.65E-02	-1.71E-01	-4.82E-02
-1.25E-02	-2.20E-02	-8.21E-02	-9.47E-02	-1.97E-02	7.59E-02	1.38E-02	-1.42E-02	6.29E-02	8.55E-02	1.14E-01	-1.79E-02	1.79E-01	7.74E-02	2.26E-01	-8.93E-03	2.91E-01	9.72E-02	-1.08E-01	-1.60E-02	-1.21E-01	-2.65E-02	-1.37E-01	-4.34E-02	-1.69E-01	-5.05E-02
-9.71E-03	-1.84E-02	-8.64E-02	-9.57E-02	-1.82E-02	7.86E-02	1.43E-02	-8.00E-03	7.31E-02	8.33E-02	1.13E-01	-1.75E-02	1.90E-01	8.32E-02	2.30E-01	-9.36E-03	2.94E-01	9.41E-02	-1.17E-01	-9.15E-03	-1.32E-01	-1.81E-02	-1.46E-01	-3.79E-02	-1.73E-01	-5.04E-02
-2.73E-02	-7.81E-03	-8.51E-02	-9.27E-02	-1.89E-02	8.27E-02	9.99E-03	-9.75E-03	6.63E-02	8.93E-02	1.20E-01	-1.69E-02	1.80E-01	8.08E-02	2.29E-01	-1.68E-02	2.79E-01	8.82E-02	-1.21E-01	1.09E-02	-1.31E-01	-3.56E-03	-1.41E-01	-3.23E-02	-1.64E-01	-5.65E-02
-3.38E-02	-1.10E-02	-9.65E-02	-9.06E-02	-1.44E-02	7.89E-02	8.29E-03	-8.74E-03	7.33E-02	8.34E-02	1.18E-01	-1.91E-02	1.85E-01	7.65E-02	2.33E-01	-6.67E-03	2.75E-01	8.78E-02	-1.10E-01	-1.09E-02	-1.27E-01	-1.77E-02	-1.46E-01	-3.82E-02	-1.77E-01	-5.25E-02
-2.04E-02	-9.63E-03	-8.78E-02	-8.68E-02	-2.97E-02	7.36E-02	9.82E-03	-5.84E-03	6.29E-02	8.39E-02	1.19E-01	-1.55E-02	1.83E-01	7.94E-02	2.32E-01	-1.48E-02	2.97E-01	9.04E-02	-1.19E-01	-8.43E-03	-1.27E-01	-2.16E-02	-1.37E-01	-4.04E-02	-1.64E-01	-4.68E-02
-2.22E-02	-1.38E-02	-9.81E-02	-8.95E-02	-2.59E-02	7.94E-02	9.57E-03	-9.42E-03	6.52E-02	8.73E-02	1.19E-01	-1.83E-02	1.83E-01	8.37E-02	2.30E-01	-1.14E-02	3.01E-01	9.60E-02	-1.12E-01	-4.81E-03	-1.24E-01	-1.68E-02	-1.39E-01	-4.02E-02	-1.70E-01	-5.47E-02
-3.57E-02	-7.08E-03	-8.90E-02	-8.48E-02	-2.36E-02	7.12E-02	8.37E-03	-1.26E-02	7.74E-02	8.44E-02	1.14E-01	-1.54E-02	1.93E-01	8.07E-02	2.29E-01	-1.80E-02	2.96E-01	9.59E-02	-1.24E-01	3.90E-03	-1.30E-01	-9.17E-03	-1.44E-01	-2.79E-02	-1.72E-01	-4.59E-02
-4.23E-02	-1.39E-02	-7.26E-02	-1.16E-01	-2.83E-02	8.48E-02	-8.78E-03	-1.64E-02	6.58E-02	7.81E-02	1.10E-01	-1.75E-02	1.84E-01	9.31E-02	2.29E-01	-1.11E-02	2.91E-01	1.07E-01	-1.15E-01	-8.21E-03	-1.25E-01	-1.84E-02	-1.36E-01	-4.21E-02	-1.63E-01	-5.66E-02
-4.02E-02	-1.85E-02	-9.59E-02	-1.02E-01	-2.26E-02	8.90E-02	2.51E-03	-1.70E-02	7.21E-02	9.56E-02	1.10E-01	-2.00E-02	1.77E-01	8.42E-02	2.23E-01	-1.34E-02	2.84E-01	9.87E-02	-1.15E-01	-2.19E-02	-1.31E-01	-3.41E-02	-1.45E-01	-6.08E-02	-1.72E-01	-7.22E-02
-3.22E-02	-5.43E-03	-9.79E-02	-9.75E-02	-1.56E-02	8.45E-02	8.65E-03	-9.02E-03	8.02E-02	9.01E-02	1.18E-01	-1.17E-02	1.91E-01	8.39E-02	2.28E-01	-8.47E-03	2.92E-01	9.65E-02	-1.24E-01	1.82E-03	-1.33E-01	-1.41E-02	-1.40E-01	-4.40E-02	-1.67E-01	-6.15E-02
-2.78E-02	-8.95E-03	-9.77E-02	-9.84E-02	-1.58E-02	7.35E-02	9.51E-03	-1.45E-02	6.82E-02	8.23E-02	1.16E-01	-1.71E-02	1.81E-01	8.21E-02	2.32E-01	-1.40E-02	2.82E-01	9.90E-02	-1.06E-01	-3.39E-03	-1.21E-01	-1.21E-02	-1.37E-01	-3.61E-02	-1.69E-01	-5.15E-02
-3.17E-02	-1.22E-02	-8.48E-02	-1.01E-01	-7.81E-03	7.57E-02	1.23E-02	-1.39E-02	7.73E-02	7.98E-02	1.22E-01	-1.67E-02	1.86E-01	8.45E-02	2.31E-01	-1.52E-02	2.88E-01	9.90E-02	-1.13E-01	5.16E-03	-1.25E-01	-7.53E-03	-1.37E-01	-3.52E-02	-1.67E-01	-5.41E-02
-3.12E-02	-1.22E-02	-9.09E-02	-9.40E-02	-1.53E-02	7.74E-02	1.16E-02	-1.68E-02	7.36E-02	8.53E-02	1.20E-01	-1.13E-02	1.86E-01	8.42E-02	2.29E-01	-5.73E-03	2.88E-01	9.98E-02	-1.18E-01	-1.04E-02	-1.30E-01	-2.31E-02	-1.47E-01	-4.59E-02	-1.81E-01	-5.90E-02
-4.78E-02	-2.71E-03	-8.63E-02	-1.14E-01	-2.11E-02	7.57E-02	9.00E-03	-9.57E-03	6.22E-02	8.79E-02	1.16E-01	-1.83E-02	1.81E-01	8.66E-02	2.28E-01	-1.32E-02	2.76E-01	8.42E-02	-1.21E-01	1.25E-02	-1.25E-01	-6.20E-03	-1.33E-01	-3.99E-02	-1.67E-01	-6.13E-02
-2.82E-02	-9.25E-03	-8.90E-02	-1.03E-01	-2.25E-02	8.33E-02	1.14E-02	-7.35E-03	7.22E-02	9.22E-02	1.10E-01	-1.20E-02	1.84E-01	9.27E-02	2.26E-01	-1.15E-02	2.90E-01	1.05E-01	-9.90E-02	-7.85E-03	-1.15E-01	-1.59E-02	-1.36E-01	-3.71E-02	-1.73E-01	-5.60E-02
-2.55E-02	-1.29E-02	-8.20E-02	-9.11E-02	-1.59E-02	8.02E-02	1.36E-02	-1.15E-02	7.44E-02	8.57E-02	1.15E-01	-2.02E-02	1.93E-01	7.97E-02	2.30E-01	-1.52E-02	2.85E-01	9.72E-02	-1.23E-01	7.75E-03	-1.31E-01	-7.18E-03	-1.38E-01	-3.48E-02	-1.65E-01	-5.09E-02
-4.13E-02	-1.37E-02	-8.91E-02	-9.76E-02	-2.92E-02	8.01E-02	-6.63E-03	-1.36E-02	7.01E-02	9.05E-02	1.12E-01	-1.89E-02	1.89E-01	9.08E-02	2.31E-01	-1.63E-02	2.89E-01	1.03E-01	-1.13E-01	-3.89E-03	-1.29E-01	-9.93E-03	-1.46E-01	-2.93E-02	-1.75E-01	-4.68E-02
-4.36E-02	-6.39E-03	-9.99E-02	-9.33E-02	-8.19E-03	8.58E-02	8.72E-03	-1.68E-02	8.65E-02	9.58E-02	1.11E-01	-2.02E-02	1.83E-01	9.21E-02	2.23E-01	-1.55E-02	2.81E-01	1.01E-01	-1.24E-01	-5.98E-03	-1.27E-01	-2.51E-02	-1.36E-01	-5.51E-02	-1.70E-01	-7.24E-02
-2.24E-02	-1.32E-02	-9.05E-02	-9.60E-02	-1.95E-02	7.93E-02	8.79E-03	-1.34E-02	7.11E-02	8.65E-02	1.14E-01	-1.54E-02	1.81E-01	8.55E-02	2.29E-01	-1.55E-02	2.90E-01	9.85E-02	-1.11E-01	-7.31E-03	-1.24E-01	-1.63E-02	-1.40E-01	-3.47E-02	-1.72E-01	-4.36E-02
-3.53E-02	-9.76E-03	-8.28E-02	-9.24E-02	-1.24E-02	7.37E-02	1.57E-02	-1.46E-02	7.43E-02	8.27E-02	1.15E-01	-2.21E-02	1.87E-01	8.22E-02	2.20E-01	-1.80E-02	2.88E-01	9.49E-02	-1.11E-01	-4.12E-03	-1.24E-01	-1.51E-02	-1.36E-01	-4.13E-02	-1.67E-01	-5.75E-02
-3.08E-02	-8.30E-03	-9.39E-02	-8.48E-02	-2.48E-02	6.93E-02	1.43E-02	-8.48E-03	7.29E-02	7.55E-02	1.22E-01	-1.71E-02	1.88E-01	7.45E-02	2.37E-01	-1.27E-02	2.96E-01	8.95E-02	-1.16E-01	7.79E-04	-1.32E-01	-7.97E-03	-1.44E-01	-3.08E-02	-1.72E-01	-4.46E-02
-1.85E-02	-1.53E-02	-8.90E-02	-9.06E-02	-1.79E-02	8.08E-02	9.15E-03	-1.59E-02	7.02E-02	9.44E-02	1.17E-01	-1.85E-02	1.81E-01	8.87E-02	2.26E-01	-1.55E-02	2.90E-01	9.83E-02	-1.16E-01	-1.61E-03	-1.25E-01	-1.67E-02	-1.37E-01	-4.14E-02	-1.67E-01	-5.35E-02
-2.04E-02	-1.17E-02	-9.13E-02	-9.84E-02	-2.30E-02	7.62E-02	8.85E-03	-1.96E-02	6.32E-02	8.87E-02	1.19E-01	-2.02E-02	1.75E-01	8.58E-02	2.26E-01	-1.31E-02	2.85E-01	1.00E-01	-1.14E-01	-1.97E-03	-1.24E-01	-1.65E-02	-1.37E-01	-4.08E-02	-1.70E-01	-5.43E-02
-3.24E-02	-9.44E-03	-9.59E-02	-9.21E-02	-2.15E-02	7.79E-02	5.67E-03	-7.34E-03	7.37E-02	8.26E-02	1.14E-01	-1.46E-02	1.93E-01	7.92E-02	2.33E-01	-1.59E-02	2.94E-01	9.75E-02	-1.11E-01	1.53E-04	-1.25E-01	-9.71E-03	-1.38E-01	-3.53E-02	-1.67E-01	-5.34E-02
-1.79E-02	-6.82E-03	-8.96E-02	-9.47E-02	-2.37E-02	7.85E-02	1.31E-02	-1.57E-03	6.71E-02	8.65E-02	1.20E-01	-1.25E-02	1.95E-01	8.20E-02	2.27E-01	-1.35E-02	2.92E-01	9.55E-02	-1.09E-01	-4.88E-03	-1.21E-01	-1.49E-02	-1.35E-01	-3.52E-02	-1.65E-01	-4.76E-02
-2.85E-02	-8.66E-03	-8.94E-02	-9.66E-02	-1.58E-02	7.78E-02	1.26E-02	-7.23E-03	7.30E-02	8.24E-02	1.12E-01	-1.43E-02	1.86E-01	8.15E-02	2.29E-01	-1.02E-02	2.94E-01	9.03E-02	-1.12E-01	3.93E-04	-1.28E-01	-1.10E-02	-1.40E-01	-3.84E-02	-1.68E-01	-5.91E-02
-1.80E-02	-1.16E-02	-8.63E-02	-8.36E-02	-1.91E-02	7.19E-02	1.29E-02	-8.93E-03	7.06E-02	7.42E-02	1.19E-01	-1.45E-02	1.91E-01	7.42E-02	2.28E-01	-1.16E-02	2.89E-01	8.76E-02	-1.10E-01	-5.28E-03	-1.23E-01	-1.73E-02	-1.34E-01	-4.02E-02	-1.64E-01	-4.61E-02
-3.32E-02	-3.19E-03	-8.71E-02	-9.27E-02	-1.77E-02	8.33E-02	6.99E-03	-1.19E-02	7.32E-02	9.11E-02	1.14E-01	-1.79E-02	1.87E-01	8.34E-02	2.23E-01	-1.58E-02	2.82E-01	9.15E-02	-1.21E-01	5.54E-03	-1.25E-01	-1.38E-02	-1.29E-01	-4.81E-02	-1.51E-01	-6.96E-02
-1.88E-02	-1.15E-02	-8.68E-02	-9.42E-02	-1.45E-02	8.01E-02	1.74E-02	-8.76E-03	6.40E-02	8.96E-02	1.13E-01	-1.53E-02	1.91E-01	8.61E-02	2.30E-01	-1.29E-02	2.88E-01	9.11E-02	-1.01E-01	-4.63E-03	-1.18E-01	-1.23E-02	-1.38E-01	-3.26E-02	-1.74E-01	-5.02E-02
-1.95E-02	-1.64E-02	-1.01E-01	-8.82E-02	-1.99E-02	7.18E-02	1.37E-02	-1.30E-02	6.25E-02	8.17E-02	1.17E-01	-2.28E-02	1.81E-01	7.97E-02	2.34E-01	-1.78E-02	3.02E-01	1.06E-01	-1.03E-01	-4.60E-03	-1.15E-01	-1.65E-02	-1.35E-01	-3.59E-02	-1.73E-01	-4.82E-02
-3.17E-02	-9.54E-03	-9.37E-02	-9.07E-02	-2.88E-02	6.83E-02	1.20E-02	-1.13E-02	6.37E-02	7.45E-02	1.17E-01	-2.04E-02	1.84E-01	7.69E-02	2.35E-01	-1.29E-02	2.95E-01	9.34E-02	-1.04E-01	-7.92E-03	-1.18E-01	-1.71E-02	-1.37E-01	-3.69E-02	-1.73E-01	-5.05E-02
-2.72E-02	-1.35E-02	-8.31E-02	-9.17E-02	-2.15E-02	7.79E-02	8.23E-03	-1.21E-02	6.70E-02	7.56E-02	1.21E-01	-2.39E-02	1.82E-01	7.12E-02	2.29E-01	-2.10E-02	2.92E-01	8.72E-02	-1.04E-01	2.77E-03	-1.17E-01	-9.65E-03	-1.33E-01	-3.65E-02	-1.66E-01	-5.51E-02
-2.87E-02	-9.43E-03	-8.58E-02	-9.59E-02	-1.77E-02	7.78E-02	1.35E-02	-8.58E-03	7.27E-02	8.18E-02	1.15E-01	-1.69E-02	1.86E-01	7.83E-02	2.28E-01	-1.12E-02	2.94E-01	8.75E-02	-1.11E-01	-6.63E-04	-1.22E-01	-1.51E-02	-1.30E-01	-4.46E-02	-1.59E-01	-5.87E-02
-3.50E-02	-8.57E-03	-9.32E-02	-8.44E-02	-2.14E-02	7.34E-02	2.31E-03	-1.04E-02	6.69E-02	7.55E-02	1.14E-01	-1.83E-02	1.92E-01	7.64E-02	2.28E-01	-1.91E-02	2.91E-01	8.63E-02	-1.09E-01	-6.19E-03	-1.23E-01	-1.74E-02	-1.38E-01	-3.83E-02	-1.70E-01	-4.81E-02
-2.70E-02	-1.35E-02	-8.18E-02	-9.05E-02	-2.66E-02	7.62E-02	1.37E-02	-1.51E-02	5.64E-02	8.16E-02	1.16E-01	-2.03E-02	1.81E-01	7.72E-02	2.36E-01	-2.02E-02	2.92E-01	8.75E-02	-1.05E-01	-5.24E-03	-1.17E-01	-1.80E-02	-1.29E-01	-4.26E-02	-1.61E-01	-5.48E-02
-3.24E-02	-6.46E-03	-9.21E-02	-9.35E-02	-1.65E-02	8.03E-02	9.90E-03	-9.01E-03	7.31E-02	8.55E-02	1.19E-01	-2.09E-02	1.85E-01	8.02E-02	2.28E-01	-1.68E-02	2.94E-01	9.06E-02	-1.15E-01	8.54E-03	-1.10E-01	-1.02E-02	-1.17E-01	-4.25E-02	-1.48E-01	-6.95E-02
-1.09E-02	-2.34E-02	-8.91E-02	-9.77E-02	-2.96E-02	7.26E-02	9.51E-03	-1.28E-02	6.03E-02	7.72E-02	1.18E-01	-1.64E-02	1.86E-01	7.62E-02	2.33E-01	-9.28E-03	2.83E-01	9.09E-02	-1.07E-01	-1.59E-02	-1.23E-01	-2.21E-02	-1.42E-01	-3.87E-02	-1.77E-01	-4.84E-02
-3.16E-02	-7.42E-03	-8.98E-02	-9.18E-02	-2.96E-02	7.79E-02	8.94E-03	-1.28E-02	6.30E-02	8.01E-02	1.19E-01	-1.46E-02	1.84E-01	7.85E-02	2.31E-01	-1.58E-02	2.96E-01	9.58E-02	-1.14E-01	-8.66E-03	-1.28E-01	-1.80E-02	-1.42E-01	-3.74E-02	-1.69E-01	-4.92E-02
-2.63E-02	-1.48E-02	-9.64E-02	-8.65E-02	-1.89E-02	7.63E-02	1.60E-02	-9.87E-03	6.70E-02	7.77E-02	1.19E-01	-1.94E-02	1.81E-01	7.70E-02	2.30E-01	-2.00E-02	2.86E-01	8.81E-02	-1.06E-01	-7.16E-03	-1.21E-01	-1.90E-02	-1.38E-01	-4.65E-02	-1.71E-01	-6.66E-02
-4.09E-02	-7.61E-03	-9.07E-02	-8.45E-02	-2.35E-02	7.43E-02	8.48E-03	-1.09E-02	7.23E-02	8.02E-02	1.14E-01	-2.08E-02	1.89E-01	7.47E-02	2.26E-01	-1.50E-02	2.88E-01	9.50E-02	-1.16E-01	-2.17E-04	-1.27E-01	-1.24E-02	-1.38E-01	-3.63E-02	-1.64E-01	-5.06E-02
-2.26E-02	-1.38E-02	-8.62E-02	-9.14E-02	-1.60E-02	7.17E-02	1.02E-02	-1.46E-02	7.45E-02	8.38E-02	1.17E-01	-2.18E-02	1.82E-01	8.59E-02	2.29E-01	-1.81E-02	2.83E-01	8.65E-02	-1.11E-01	1.72E-03	-1.24E-01	-1.07E-02	-1.34E-01	-3.98E-02	-1.64E-01	-5.89E-02
-2.91E-02	-5.39E-03	-9.28E-02	-8.88E-02	-1.70E-02	7.59E-02	1.25E-02	-1.02E-02	7.53E-02	8.03E-02	1.18E-01	-1.55E-02	1.85E-01	7.99E-02	2.25E-01	-1.25E-02	2.85E-01	8.38E-02	-1.15E-01	1.88E-05	-1.27E-01	-1.48E-02	-1.35E-01	-4.26E-02	-1.65E-01	-4.91E-02
-2.14E-02	-7.86E-03	-7.75E-02	-8.49E-02	-1.83E-02	8.40E-02	1.41E-02	-5.01E-03	7.30E-02	8.47E-02	1.16E-01	-1.22E-02	1.86E-01	8.04E-02	2.35E-01	-1.41E-02	2.93E-01	9.14E-02	-1.04E-01	-3.74E-03	-1.17E-01	-1.49E-02	-1.30E-01	-3.63E-02	-1.61E-01	-4.90E-02
-3.11E-02	-1.05E-02	-9.07E-02	-9.09E-02	-1.64E-02	8.33E-02	-3.00E-03	-1.20E-02	7.79E-02	9.07E-02	1.15E-01	-2.00E-02	1.88E-01	8.59E-02	2.27E-01	-1.16E-02	2.94E-01	9.60E-02	-1.15E-01	-1.85E-02	-1.28E-01	-2.85E-02	-1.43E-01	-4.70E-02	-1.73E-01	-5.68E-02
-4.03E-02	-1.68E-02	-8.70E-02	-8.93E-02	-1.88E-02	7.54E-02	4.69E-03	-1.63E-02	6.89E-02	7.90E-02	1.15E-01	-1.77E-02	1.77E-01	7.40E-02	2.29E-01	-1.61E-02	2.86E-01	9.17E-02	-1.16E-01	-1.55E-02	-1.29E-01	-2.47E-02	-1.43E-01	-4.75E-02	-1.73E-01	-5.74E-02
-3.81E-02	-8.76E-03	-9.16E-02	-9.72E-02	-1.61E-02	7.99E-02	8.07E-03	-1.70E-02	7.03E-02	8.87E-02	1.12E-01	-2.37E-02	1.82E-01	8.07E-02	2.26E-01	-1.45E-02	2.95E-01	9.55E-02	-1.11E-01	8.22E-03	-1.25E-01	-4.07E-03	-1.35E-01	-3.54E-02	-1.63E-01	-6.12E-02
-3.33E-02	-1.19E-02	-9.92E-02	-9.17E-02	-1.71E-02	7.47E-02	5.78E-03	-1.39E-02	6.72E-02	7.68E-02	1.15E-01	-1.49E-02	1.84E-01	7.69E-02	2.31E-01	-1.41E-02	2.92E-01	9.41E-02	-1.09E-01	-8.03E-03	-1.24E-01	-1.64E-02	-1.39E-01	-3.62E-02	-1.70E-01	-4.46E-02
-1.37E-02	-1.47E-02	-8.73E-02	-8.35E-02	-1.56E-02	7.01E-02	1.33E-02	-8.98E-03	7.91E-02	7.65E-02	1.14E-01	-1.72E-02	1.83E-01	7.45E-02	2.34E-01	-1.52E-02	2.94E-01	8.87E-02	-1.15E-01	-2.57E-03	-1.23E-01	-2.00E-02	-1.34E-01	-4.74E-02	-1.62E-01	-5.70E-02
-2.38E-02	-8.93E-03	-8.64E-02	-9.09E-02	-2.62E-02	7.48E-02	1.51E-02	-1.16E-02	5.97E-02	7.78E-02	1.20E-01	-1.55E-02	1.82E-01	7.48E-02	2.33E-01	-1.99E-02	2.89E-01	8.79E-02	-1.15E-01	1.46E-03	-1.25E-01	-1.23E-02	-1.36E-01	-3.73E-02	-1.61E-01	-5.31E-02
-3.93E-02	-7.09E-03	-8.66E-02	-9.04E-02	-2.38E-02	7.89E-02	1.23E-03	-4.93E-03	7.34E-02	8.13E-02	1.15E-01	-1.70E-02	1.86E-01	7.51E-02	2.34E-01	-1.50E-02	2.86E-01	8.90E-02	-1.17E-01	1.47E-03	-1.24E-01	-1.39E-02	-1.34E-01	-4.10E-02	-1.64E-01	-5.89E-02

-1.24E-02	-1.55E-02	-8.63E-02	-8.17E-02	-3.30E-02	7.48E-02	1.35E-02	-7.81E-03	5.71E-02	7.83E-02	1.22E-01	-1.60E-02	1.89E-01	7.79E-02	2.34E-01	-1.35E-02	3.00E-01	8.17E-02	-1.10E-01	-9.15E-03	-1.24E-01	-1.78E-02	-1.39E-01	-3.74E-02	-1.69E-01	-4.84E-02
-2.55E-02	-9.41E-03	-9.23E-02	-8.94E-02	-2.81E-02	7.78E-02	9.75E-03	-7.22E-03	8.28E-02	8.30E-02	1.18E-01	-1.50E-02	1.94E-01	8.54E-02	2.28E-01	-9.80E-03	2.82E-01	8.51E-02	-1.14E-01	-1.70E-02	-1.28E-01	-2.46E-02	-1.43E-01	-3.77E-02	-1.72E-01	-4.39E-02
-2.66E-02	-1.20E-02	-9.26E-02	-9.76E-02	-2.43E-02	7.64E-02	1.11E-02	-1.01E-02	6.97E-02	8.86E-02	1.13E-01	-1.65E-02	1.81E-01	8.42E-02	2.30E-01	-1.60E-02	2.89E-01	9.22E-02	-1.12E-01	-1.05E-02	-1.28E-01	-1.78E-02	-1.45E-01	-3.77E-02	-1.79E-01	-4.96E-02
-2.70E-02	-1.67E-02	-8.57E-02	-9.57E-02	-2.34E-02	8.11E-02	8.30E-03	-1.65E-02	7.23E-02	8.70E-02	1.18E-01	-2.20E-02	1.91E-01	8.29E-02	2.34E-01	-1.67E-02	2.87E-01	9.43E-02	-1.11E-01	-4.92E-03	-1.23E-01	-1.54E-02	-1.41E-01	-3.24E-02	-1.76E-01	-4.68E-02
-3.89E-02	-1.88E-02	-9.85E-02	-8.57E-02	-2.14E-02	6.88E-02	3.11E-03	-1.30E-02	6.15E-02	7.56E-02	1.21E-01	-1.61E-02	1.93E-01	7.28E-02	2.32E-01	-1.06E-02	2.96E-01	9.31E-02	-1.18E-01	-3.15E-03	-1.30E-01	-1.40E-02	-1.44E-01	-3.42E-02	-1.72E-01	-4.69E-02
-4.69E-02	-1.11E-02	-9.66E-02	-9.32E-02	-1.81E-02	7.52E-02	1.60E-03	-1.70E-02	6.76E-02	8.08E-02	1.20E-01	-1.87E-02	1.88E-01	8.33E-02	2.29E-01	-1.42E-02	2.92E-01	9.53E-02	-1.12E-01	3.73E-03	-1.25E-01	-8.25E-03	-1.40E-01	-3.47E-02	-1.68E-01	-5.78E-02
-3.35E-02	-8.62E-03	-8.56E-02	-9.59E-02	-2.03E-02	8.29E-02	5.62E-03	-1.47E-02	7.89E-02	8.15E-02	1.16E-01	-2.07E-02	1.87E-01	7.77E-02	2.32E-01	-2.17E-02	2.97E-01	8.75E-02	-1.03E-01	-3.29E-03	-1.14E-01	-1.62E-02	-1.27E-01	-4.32E-02	-1.58E-01	-6.03E-02
-1.51E-02	-1.66E-02	-8.86E-02	-8.48E-02	-2.40E-02	7.39E-02	1.99E-02	-1.31E-02	7.12E-02	8.67E-02	1.19E-01	-1.60E-02	1.83E-01	8.99E-02	2.26E-01	-1.45E-02	2.87E-01	9.14E-02	-1.11E-01	-1.45E-02	-1.26E-01	-2.34E-02	-1.42E-01	-4.12E-02	-1.75E-01	-4.79E-02
-1.79E-02	-1.13E-02	-8.76E-02	-9.38E-02	-2.32E-02	7.68E-02	1.19E-02	-1.40E-02	6.78E-02	8.52E-02	1.16E-01	-1.81E-02	1.81E-01	7.68E-02	2.29E-01	-1.38E-02	2.85E-01	9.47E-02	-1.04E-01	-7.39E-04	-1.21E-01	-6.67E-03	-1.41E-01	-2.71E-02	-1.74E-01	-4.72E-02
-1.59E-02	-1.40E-02	-9.33E-02	-9.38E-02	-2.57E-02	7.38E-02	1.16E-02	-8.56E-03	6.64E-02	7.99E-02	1.17E-01	-1.52E-02	1.96E-01	8.25E-02	2.28E-01	-9.06E-03	2.92E-01	9.27E-02	-1.16E-01	-1.05E-02	-1.25E-01	-2.54E-02	-1.38E-01	-4.61E-02	-1.69E-01	-5.17E-02
-2.69E-02	-1.42E-02	-8.55E-02	-8.80E-02	-4.06E-02	7.21E-02	1.30E-02	-1.41E-02	5.98E-02	7.82E-02	1.22E-01	-2.31E-02	1.81E-01	7.13E-02	2.36E-01	-1.51E-02	2.82E-01	8.25E-02	-1.11E-01	-1.21E-03	-1.22E-01	-1.18E-02	-1.35E-01	-3.29E-02	-1.64E-01	-4.61E-02
-2.43E-02	-1.20E-02	-8.50E-02	-9.04E-02	-2.08E-02	7.64E-02	9.45E-03	-1.13E-02	6.97E-02	8.81E-02	1.15E-01	-1.44E-02	1.91E-01	8.14E-02	2.23E-01	-1.20E-02	2.90E-01	8.65E-02	-1.13E-01	1.51E-03	-1.20E-01	-1.34E-02	-1.30E-01	-3.86E-02	-1.60E-01	-5.18E-02
-2.49E-02	-1.11E-02	-8.25E-02	-9.71E-02	-1.81E-02	8.38E-02	1.43E-02	-1.32E-02	6.87E-02	8.44E-02	1.16E-01	-1.93E-02	1.76E-01	8.48E-02	2.32E-01	-1.25E-02	2.89E-01	9.00E-02	-1.18E-01	-1.00E-02	-1.31E-01	-2.16E-02	-1.41E-01	-5.04E-02	-1.70E-01	-6.54E-02
-3.04E-02	-1.19E-02	-9.15E-02	-9.19E-02	-1.53E-02	7.49E-02	1.41E-02	-3.93E-03	7.77E-02	7.93E-02	1.26E-01	-1.72E-02	1.91E-01	7.10E-02	2.32E-01	-1.22E-02	2.88E-01	8.68E-02	-1.04E-01	-1.19E-02	-1.18E-01	-1.95E-02	-1.35E-01	-3.98E-02	-1.69E-01	-5.16E-02
-1.98E-02	-1.78E-02	-8.63E-02	-9.43E-02	-1.68E-02	7.56E-02	9.68E-03	-1.36E-02	7.31E-02	8.26E-02	1.19E-01	-1.98E-02	1.86E-01	8.02E-02	2.27E-01	-1.46E-02	2.88E-01	9.22E-02	-1.11E-01	2.84E-03	-1.20E-01	-1.14E-02	-1.33E-01	-3.80E-02	-1.68E-01	-5.04E-02
-3.98E-02	-5.37E-03	-1.00E-01	-9.81E-02	-1.33E-02	7.78E-02	6.92E-03	-1.73E-02	6.98E-02	9.71E-02	1.16E-01	-1.73E-02	1.83E-01	9.08E-02	2.25E-01	-1.40E-02	2.77E-01	9.97E-02	-1.11E-01	-5.40E-03	-1.22E-01	-1.99E-02	-1.34E-01	-5.02E-02	-1.68E-01	-6.07E-02
-1.69E-02	-1.60E-02	-8.93E-02	-8.67E-02	-9.80E-03	7.63E-02	1.32E-02	-1.18E-02	7.28E-02	8.66E-02	1.19E-01	-1.76E-02	1.87E-01	8.31E-02	2.32E-01	-1.08E-02	2.85E-01	9.38E-02	-1.13E-01	-9.70E-03	-1.25E-01	-2.05E-02	-1.42E-01	-3.66E-02	-1.77E-01	-4.68E-02
-1.96E-02	-1.29E-02	-8.62E-02	-9.77E-02	-1.85E-02	8.11E-02	1.91E-02	-6.54E-03	7.01E-02	8.75E-02	1.27E-01	-1.50E-02	1.87E-01	8.55E-02	2.26E-01	-9.62E-03	2.81E-01	9.91E-02	-1.08E-01	-1.46E-02	-1.21E-01	-2.53E-02	-1.37E-01	-4.22E-02	-1.70E-01	-5.04E-02
-2.96E-02	-1.60E-02	-8.81E-02	-9.56E-02	-1.79E-02	8.01E-02	6.92E-03	-9.68E-03	7.21E-02	9.15E-02	1.15E-01	-2.29E-02	1.89E-01	8.82E-02	2.27E-01	-1.48E-02	3.03E-01	9.90E-02	-1.10E-01	-8.13E-03	-1.23E-01	-1.97E-02	-1.37E-01	-4.42E-02	-1.68E-01	-5.96E-02
-2.26E-02	-1.28E-02	-8.83E-02	-9.16E-02	-3.10E-02	8.22E-02	2.36E-02	-9.00E-03	6.42E-02	8.13E-02	1.10E-01	-1.71E-02	1.89E-01	8.10E-02	2.27E-01	-1.23E-02	2.79E-01	7.82E-02	-1.04E-01	-3.76E-03	-1.22E-01	-8.98E-03	-1.40E-01	-2.74E-02	-1.72E-01	-4.32E-02
-2.50E-02	-1.70E-02	-9.07E-02	-9.24E-02	-1.70E-02	7.45E-02	7.08E-03	-1.55E-02	9.00E-02	7.45E-02	1.25E-01	-1.94E-02	1.79E-01	7.91E-02	2.31E-01	-8.55E-03	2.86E-01	9.16E-02	-1.09E-01	4.16E-03	-1.21E-01	-9.38E-03	-1.37E-01	-3.49E-02	-1.66E-01	-5.68E-02
-2.43E-02	-2.55E-03	-9.33E-02	-8.95E-02	-2.70E-02	7.85E-02	1.55E-02	-1.73E-02	5.49E-02	8.59E-02	1.15E-01	-1.73E-02	1.86E-01	8.54E-02	2.39E-01	-1.29E-02	2.93E-01	9.62E-02	-1.12E-01	-1.95E-03	-1.24E-01	-1.48E-02	-1.37E-01	-3.97E-02	-1.69E-01	-5.05E-02
-1.70E-02	-1.09E-02	-7.96E-02	-1.02E-01	-2.35E-02	8.47E-02	7.48E-03	-8.55E-03	6.94E-02	9.29E-02	1.12E-01	-1.49E-02	1.84E-01	8.43E-02	2.28E-01	-1.58E-02	2.96E-01	9.93E-02	-1.05E-01	-4.42E-03	-1.19E-01	-1.73E-02	-1.34E-01	-4.45E-02	-1.68E-01	-6.60E-02
-3.91E-02	-9.74E-03	-8.68E-02	-1.02E-01	-1.07E-02	8.06E-02	1.32E-02	-1.15E-02	7.64E-02	9.13E-02	1.12E-01	-2.14E-02	1.87E-01	8.54E-02	2.27E-01	-1.90E-02	2.89E-01	9.64E-02	-1.01E-01	-4.23E-03	-1.14E-01	-1.66E-02	-1.28E-01	-4.69E-02	-1.62E-01	-6.63E-02
-3.16E-02	-1.05E-02	-8.50E-02	-8.76E-02	-2.26E-02	7.94E-02	1.07E-02	-1.15E-02	6.70E-02	8.48E-02	1.19E-01	-1.85E-02	1.89E-01	7.97E-02	2.29E-01	-2.04E-02	2.93E-01	8.75E-02	-1.17E-01	1.81E-03	-1.29E-01	-9.32E-03	-1.40E-01	-3.19E-02	-1.66E-01	-4.47E-02
-3.27E-02	-1.56E-02	-9.43E-02	-9.36E-02	-1.63E-02	8.11E-02	8.61E-03	-1.06E-02	7.33E-02	9.02E-02	1.20E-01	-1.24E-02	1.88E-01	8.13E-02	2.36E-01	-7.96E-03	2.86E-01	9.57E-02	-1.15E-01	-9.22E-03	-1.28E-01	-1.93E-02	-1.42E-01	-3.83E-02	-1.71E-01	-4.65E-02
-3.45E-02	-8.80E-03	-8.27E-02	-9.01E-02	-2.29E-02	7.11E-02	1.87E-02	-1.72E-02	6.47E-02	7.82E-02	1.21E-01	-1.87E-02	1.77E-01	7.32E-02	2.29E-01	-1.79E-02	2.88E-01	8.43E-02	-1.25E-01	5.57E-03	-1.32E-01	-9.44E-03	-1.38E-01	-3.68E-02	-1.62E-01	-4.68E-02
-1.68E-02	1.06E-03	-8.29E-02	-1.02E-01	-2.09E-02	8.49E-02	1.90E-02	-3.93E-03	7.19E-02	8.81E-02	1.16E-01	-1.47E-02	1.85E-01	8.13E-02	2.29E-01	-1.34E-02	2.90E-01	8.89E-02	-1.02E-01	9.18E-03	-1.18E-01	-2.97E-03	-1.32E-01	-3.42E-02	-1.67E-01	-5.99E-02
-3.10E-02	-1.59E-02	-9.27E-02	-8.70E-02	-1.94E-02	7.43E-02	1.08E-02	-1.93E-02	6.84E-02	8.25E-02	1.15E-01	-1.84E-02	1.84E-01	7.73E-02	2.31E-01	-1.33E-02	2.90E-01	9.94E-02	-1.05E-01	1.22E-03	-1.17E-01	-1.40E-02	-1.32E-01	-4.00E-02	-1.66E-01	-5.71E-02
-2.92E-02	-8.65E-03	-8.72E-02	-8.67E-02	-3.13E-02	7.30E-02	1.04E-02	-1.27E-02	6.58E-02	7.32E-02	1.20E-01	-1.98E-02	1.88E-01	7.35E-02	2.30E-01	-1.31E-02	2.93E-01	8.43E-02	-1.18E-01	-3.20E-03	-1.25E-01	-1.84E-02	-1.37E-01	-3.98E-02	-1.67E-01	-5.40E-02
-2.18E-02	-1.59E-02	-9.05E-02	-1.01E-01	-1.26E-02	8.24E-02	3.69E-03	-1.38E-02	7.09E-02	9.09E-02	1.13E-01	-1.59E-02	1.81E-01	8.52E-02	2.29E-01	-8.19E-03	2.78E-01	9.51E-02	-1.16E-01	3.77E-03	-1.26E-01	-1.08E-02	-1.33E-01	-4.43E-02	-1.66E-01	-6.09E-02
-3.86E-02	-9.14E-03	-9.15E-02	-1.02E-01	-2.24E-02	8.27E-02	6.24E-03	-1.30E-02	6.94E-02	9.06E-02	1.08E-01	-1.82E-02	1.80E-01	8.49E-02	2.27E-01	-1.84E-02	2.80E-01	1.01E-01	-9.85E-02	-4.40E-03	-1.14E-01	-1.54E-02	-1.30E-01	-4.21E-02	-1.65E-01	-6.14E-02
-1.67E-02	-9.84E-03	-9.76E-02	-8.46E-02	-1.60E-02	7.57E-02	1.43E-02	-9.56E-03	7.25E-02	8.02E-02	1.14E-01	-2.05E-02	1.84E-01	8.08E-02	2.28E-01	-1.14E-02	2.91E-01	9.60E-02	-1.11E-01	8.85E-04	-1.24E-01	-1.07E-02	-1.41E-01	-3.30E-02	-1.73E-01	-5.00E-02
-3.00E-02	-1.89E-03	-9.37E-02	-8.93E-02	-1.85E-02	6.57E-02	1.15E-02	-1.77E-02	7.36E-02	7.23E-02	1.21E-01	-1.94E-02	1.88E-01	7.70E-02	2.32E-01	-1.31E-02	2.89E-01	8.50E-02	-1.16E-01	2.91E-05	-1.27E-01	-1.12E-02	-1.42E-01	-3.49E-02	-1.74E-01	-4.93E-02
-2.99E-02	-7.20E-03	-8.98E-02	-9.77E-02	-2.28E-02	8.15E-02	9.08E-03	-8.36E-03	6.90E-02	8.69E-02	1.13E-01	-1.75E-02	1.75E-01	8.80E-02	2.27E-01	-1.22E-02	2.86E-01	1.02E-01	-1.08E-01	-8.83E-03	-1.22E-01	-2.14E-02	-1.34E-01	-4.93E-02	-1.64E-01	-6.31E-02
-1.94E-02	-1.46E-02	-9.79E-02	-9.08E-02	-2.06E-02	7.59E-02	1.28E-02	-1.35E-02	7.10E-02	8.49E-02	1.17E-01	-1.61E-02	1.83E-01	8.43E-02	2.25E-01	-1.51E-02	2.91E-01	9.73E-02	-1.12E-01	-3.44E-03	-1.22E-01	-1.64E-02	-1.38E-01	-3.88E-02	-1.70E-01	-5.16E-02
-3.18E-02	-1.43E-02	-9.43E-02	-9.49E-02	-1.87E-02	7.50E-02	7.04E-03	-1.06E-02	7.11E-02	7.92E-02	1.21E-01	-1.56E-02	1.86E-01	8.11E-02	2.29E-01	-7.10E-03	2.78E-01	9.04E-02	-1.07E-01	-7.00E-03	-1.24E-01	-1.49E-02	-1.42E-01	-3.74E-02	-1.75E-01	-5.54E-02
-2.79E-02	-7.23E-03	-9.33E-02	-9.36E-02	-2.58E-02	7.45E-02	1.00E-02	-1.05E-02	6.66E-02	8.49E-02	1.19E-01	-1.53E-02	1.87E-01	8.09E-02	2.33E-01	-1.63E-02	2.99E-01	8.73E-02	-9.61E-02	-3.47E-03	-1.11E-01	-1.50E-02	-1.29E-01	-3.78E-02	-1.64E-01	-5.44E-02
-2.09E-02	-1.33E-02	-9.37E-02	-8.87E-02	-2.15E-02	8.02E-02	1.42E-02	-1.35E-02	7.18E-02	8.22E-02	1.19E-01	-1.71E-02	1.84E-01	7.12E-02	2.33E-01	-1.36E-02	2.89E-01	9.02E-02	-1.14E-01	6.73E-03	-1.27E-01	-5.32E-03	-1.39E-01	-3.28E-02	-1.64E-01	-5.36E-02
-1.61E-02	-4.43E-03	-8.82E-02	-9.76E-02	-2.66E-02	7.75E-02	1.72E-02	-2.26E-03	6.99E-02	8.36E-02	1.19E-01	-8.86E-03	1.86E-01	8.30E-02	2.32E-01	-1.12E-02	2.93E-01	8.20E-02	-1.09E-01	-7.18E-03	-1.25E-01	-1.58E-02	-1.44E-01	-3.34E-02	-1.75E-01	-4.97E-02
-2.54E-02	-2.01E-02	-9.64E-02	-9.00E-02	-2.21E-02	7.74E-02	1.64E-02	-1.47E-02	6.32E-02	9.06E-02	1.15E-01	-2.93E-02	1.86E-01	8.23E-02	2.28E-01	-9.77E-03	2.79E-01	9.88E-02	-1.15E-01	-9.59E-03	-1.29E-01	-1.82E-02	-1.45E-01	-3.51E-02	-1.74E-01	-4.08E-02
-2.94E-02	-1.00E-02	-9.92E-02	-9.23E-02	-1.76E-02	7.69E-02	8.97E-03	-1.03E-02	6.68E-02	8.88E-02	1.11E-01	-2.15E-02	1.84E-01	8.87E-02	2.29E-01	-1.43E-02	2.99E-01	1.09E-01	-1.19E-01	-1.14E-02	-1.33E-01	-1.93E-02	-1.49E-01	-3.48E-02	-1.77E-01	-4.34E-02
-1.50E-02	-1.70E-02	-1.05E-01	-9.13E-02	-1.33E-02	7.14E-02	5.28E-03	-1.09E-02	6.56E-02	8.68E-02	1.12E-01	-9.68E-03	1.87E-01	8.75E-02	2.28E-01	-8.72E-03	2.89E-01	9.81E-02	-1.22E-01	-7.22E-04	-1.35E-01	-1.30E-02	-1.51E-01	-3.85E-02	-1.80E-01	-5.72E-02
-1.50E-02	-1.64E-02	-1.04E-01	-8.81E-02	-2.04E-02	8.10E-02	1.15E-02	-8.07E-03	6.27E-02	8.75E-02	1.17E-01	-1.08E-02	1.89E-01	8.84E-02	2.33E-01	-9.69E-03	2.97E-01	1.02E-01	-1.23E-01	-8.03E-03	-1.37E-01	-1.87E-02	-1.51E-01	-4.16E-02	-1.77E-01	-5.48E-02
-2.13E-02	-8.11E-03	-8.24E-02	-1.03E-01	-1.52E-02	8.62E-02	7.77E-03	-4.66E-03	7.40E-02	9.22E-02	1.05E-01	-1.33E-02	1.84E-01	8.34E-02	2.17E-01	-1.57E-02	2.91E-01	9.93E-02	-1.18E-01	-6.56E-03	-1.30E-01	-2.01E-02	-1.43E-01	-4.77E-02	-1.67E-01	-6.80E-02
-2.09E-02	-1.86E-02	-9.29E-02	-9.50E-02	-2.23E-02	8.41E-02	8.11E-03	-8.24E-03	6.98E-02	9.20E-02	1.13E-01	-1.11E-02	1.88E-01	8.27E-02	2.26E-01	-1.29E-02	2.94E-01	9.88E-02	-1.16E-01	-1.37E-02	-1.27E-01	-2.64E-02	-1.43E-01	-4.80E-02	-1.76E-01	-6.02E-02
-2.88E-02	-1.37E-02	-1.13E-01	-9.14E-02	-1.83E-02	8.12E-02	9.25E-03	-1.52E-02	7.01E-02	8.92E-02	1.16E-01	-1.85E-02	1.81E-01	8.68E-02	2.36E-01	-1.07E-02	2.89E-01	1.02E-01	-1.23E-01	-5.46E-03	-1.29E-01	-2.15E-02	-1.44E-01	-4.14E-02	-1.75E-01	-5.06E-02
-3.07E-02	-1.38E-02	-9.08E-02	-9.98E-02	-2.45E-02	7.91E-02	1.04E-02	-1.53E-02	7.06E-02	8.90E-02	1.14E-01	-1.91E-02	1.85E-01	8.59E-02	2.27E-01	-1.46E-02	2.91E-01	9.50E-02	-1.12E-01	-1.05E-04	-1.20E-01	-1.53E-02	-1.33E-01	-4.03E-02	-1.65E-01	-5.34E-02
-3.40E-02	-3.08E-03	-8.57E-02	-9.90E-02	-1.93E-02	8.23E-02	1.16E-02	-6.36E-03	6.98E-02	8.96E-02	1.13E-01	-1.57E-02	1.87E-01	8.41E-02	2.31E-01	-1.87E-02	2.92E-01	9.26E-02	-1.11E-01	6.51E-03	-1.21E-01	-9.09E-03	-1.37E-01	-3.80E-02	-1.69E-01	-6.55E-02
-2.91E-02	-1.83E-02	-1.07E-01	-9.64E-02	-1.92E-02	8.13E-02	8.07E-03	-1.97E-02	6.68E-02	9.46E-02	1.19E-01	-1.75E-02	1.89E-01	9.00E-02	2.32E-01	-1.44E-02	2.92E-01	1.02E-01	-1.03E-01	-7.92E-03	-1.20E-01	-1.48E-02	-1.41E-01	-3.58E-02	-1.75E-01	-5.17E-02
-2.65E-02	-1.55E-02	-8.07E-02	-9.88E-02	-2.72E-02	8.01E-02	1.43E-03	-1.26E-02	7.26E-02	9.28E-02	1.12E-01	-1.44E-02	1.88E-01	8.46E-02	2.25E-01	-1.70E-02	2.93E-01	9.96E-02	-1.20E-01	-3.73E-04	-1.29E-01	-1.31E-02	-1.37E-01	-3.79E-02	-1.66E-01	-5.04E-02
-1.83E-02	-2.12E-02	-1.07E-01	-9.64E-02	-1.68E-02	8.02E-02	8.66E-03	-1.41E-02	6.50E-02	9.12E-02	1.14E-01	-1.39E-02	1.90E-01	9.50E-02	2.32E-01	-9.23E-03	2.98E-01	1.05E-01	-1.15E-01	-1.12E-02	-1.27E-01	-2.22E-02	-1.43E-01	-4.29E-02	-1.75E-01	-4.97E-02
-1.95E-02	-1.68E-02	-9.79E-02	-9.65E-02	-2.35E-02	7.53E-02	9.83E-03	-1.16E-02	5.93E-02	8.00E-02	1.15E-01	-1.67E-02	1.86E-01	8.07E-02	2.30E-01	-9.93E-03	2.99E-01	9.98E-02	-1.13E-01	-1.08E-02	-1.28E-01	-1.92E-02	-1.44E-01	-3.72E-02	-1.72E-01	-4.70E-02
-2.40E-02	-1.27E-02	-1.08E-01	-9.18E-02	-2.35E-02	8.25E-02	1.17E-02	-1.19E-02	6.55E-02	9.55E-02	1.19E-01	-1.64E-02	1.90E-01	9.65E-02	2.34E-01	-9.60E-03	2.93E-01	1.12E-01	-1.11E-01	-1.88E-02	-1.27E-01	-2.57E-02	-1.46E-01	-4.22E-02	-1.79E-01	-4.92E-02
-2.81E-02	-1.37E-02	-8.09E-02	-9.29E-02	-2.20E-02	7.51E-02	1.32E-02	-1.32E-02	6.69E-02	8.70E-02	1.19E-01	-1.70E-02	1.86E-01	8.00E-02	2.30E-01	-1.31E-02	2.88E-01	9.25E-02	-1.15E-01	-6.93E-03	-1.26E-01	-1.65E-02	-1.39E-01	-3.71E-02	-1.68E-01	-5.01E-02
-2.01E-02	-2.16E-02	-9.11E-02	-9.98E-02	-1.39E-02	8.36E-02	1.52E-02	-1.23E-02	7.33E-02	9.45E-02	1.15E-01	-1.58E-02	1.91E-01	9.40E-02	2.22E-01	-1.20E-02	2.87E-01	9.97E-02	-1.21E-01	3.65E-03	-1.24E-01	-1.28E-02	-1.34E-01	-3.97E-02	-1.65E-01	-5.78E-02
-3.85E-02	-5.89E-03	-8.77E-02	-9.60E-02	-2.17E-02	8.59E-02	2.50E-03	-1.41E-02	6.33E-02	8.95E-02	1.13E-01	-1.93E-02	1.84E-01	8.08E-02	2.32E-01	-1.82E-02	2.84E-01	9.61E-02	-1.24E-01	8.28E-03	-1.27E-01	-8.45E-03	-1.34E-01	-3.94E-02	-1.54E-01	-6.63E-02
-2.84E-02	-1.81E-02	-9.20E-02	-8.79E-02	-1.67E-02	7.65E-02	9.93E-03	-1.50E-02	6.61E-02	8.22E-02	1.21E-01	-1.86E-02	1.98E-01	8.29E-02	2.34E-01	-1.38E-02	2.91E-01	9.35E-02	-1.21E-01	-3.14E-04	-1.32E-01	-1.08E-02	-1.43E-01	-3.43E-02	-1.70E-01	-4.75E-02
-1.51E-02	-1.97E-02	-8.89E-02	-9.16E-02	-2.65E-02	7.57E-02	8.96E-03	-1.74E-02	6.58E-02	8.79E-02	1.14E-01	-1.77E-02	1.84E-01	8.60E-02	2.28E-01	-1.80E-02	3.01E-01	9.35E-02	-1.19E-01	-6.26E-03	-1.29E-01	-1.68E-02	-1.42E-01	-3.44E-02	-1.71E-01	-4.28E-02
-3.49E-02	-5.06E-03	-8.88E-02	-8.73E-02	-2.06E-02	8.14E-02	1.47E-02	-7.93E-03	7.01E-02	9.01E-02	1.21E-01	-1.65E-02	1.86E-01	8.52E-02	2.30E-01	-1.31E-02	2.90E-01	9.85E-02	-1.21E-01	-4.85E-03	-1.31E-01	-1.89E-02	-1.39E-01	-4.36E-02	-1.64E-01	-4.98E-02
-3.00E-02	-1.21E-02	-9.05E-02	-9.21E-02	-1.34E-02	8.33E-02	5.43E-03	-1.36E-02	6.55E-02	9.07E-02	1.18E-01	-1.89E-02	1.81E-01	8.41E-02	2.25E-01	-1.47E-02	2.81E-01	9.99E-02	-1.22E-01	-5.74E-03	-1.31E-01	-2.06E-02	-1.43E-01	-4.84E-02	-1.75E-01	-5.76E-02
-2.41E-02	-2.05E-02	-9.58E-02	-9.92E-02	-1.41E-02	8.35E-02	1.03E-02	-1.29E-02	7.36E-02	9.17E-02	1.14E-01	-1.17E-02	1.91E-01	9.37E-02	2.27E-01	-7.96E-03	2.97E-01	1.01E-01	-1.13E-01	-2.00E-02	-1.24E-01	-3.07E-02	-1.40E-01	-5.06E-02	-1.73E-01	-5.89E-02
-2.27E-02	-1.95E-02	-7.67E-02	-9.26E-02	-2.96E-02	8.23E-02	1.35E-02	-1.41E-02	5.72E-02	9.21E-02	1.20E-01	-1.53E-02	1.84E-01	8.80E-02	2.32E-01	-1.66E-02	2.89E-01	9.48E-02	-1.09E-01	-1.01E-02	-1.18E-01	-2.41E-02	-1.29E-01	-4.64E-02	-1.60E-01	-5.21E-02
-3.40E-02	-2.06E-02	-9.15E-02	-9.80E-02	-1.19E-02	8.70E-02	5.72E-03	-1.28E-02	7.37E-02	9.01E-02	1.15E-01	-9.35E-03	1.90E-01	8.72E-02	2.34E-01	-9.95E-03	2.95E-01	9.93E-02	-1.12E-01	-5.99E-03	-1.29E-01	-1.62E-02	-1.37E-01	-4.74E-02	-1.66E-01	-5.92E-02
-2.46E-02	-1.49E-02	-9.34E-02	-8.49E-02	-1.27E-02	7.64E-02	1.27E-02	-1.31E-02	7.28E-02	8.29E-02	1.22E-01	-1.69E-02	1.83E-01	8.06E-02	2.33E-01	-1.08E-02	2.88E-01	9.14E-02	-1.24E-01	3.94E-03	-1.32E-01	-1.16E-02	-1.40E-01	-3.98E-02	-1.69E-01	-5.08E-02
-1.94E-02	-1.75E-02	-9.24E-02	-1.01E-01	-1.85E-02	8.37E-02	1.88E-02	-1.64E-02	6.44E-02	8.71E-02	1.18E-01	-2.24E-02	1.77E-01	8.34E-02	2.29E-01	-1.29E-02	2.79E-01	1.11E-01	-1.22E-01	-4.70E-04	-1.25E-01	-2.50E-02	-1.32E-01	-6.40E-02	-1.61E-01	-6.71E-02
-2.14E-02	-1.18E-02	-7.89E-02	-9.88E-02	-1.94E-02	8.37E-02	1.25E-02	-9.08E-03	6.29E-02	9.25E-02	1.20E-01	-1.85E-02	1.76E-01	9.00E-02	2.32E-01	-1.48E-02	2.83E-01	9.37E-02	-1.00E-01	-4.38E-03	-1.13E-01	-1.69E-02	-1.25E-01	-4.37E-02	-1.57E-01	-5.45E-02
-1.89E-02	-1.95E-02	-1.00E-01	-9.91E-02	-2.44E-02	8.23E-02	8.60E-03	-1.64E-02	6.11E-02	8.99E-02	1.23E-01	-2.14E-02	1.79E-01	8.79E-02	2.30E-01	-1.06E-02	2.84E-01	1.07E-01	-1.13E-01	-2.60E-03	-1.20E-01	-1.69E-02	-1.35E-01	-3.83E-02	-1.68E-01	-4.86E-02
-1.80E-02	-1.89E-02	-8.30E-02	-9.67E-02	-2.03E-02	7.42E-02	7.78E-03	-8.94E-03	7.21E-02	8.43E-02	1.17E-01	-1.11E-02	1.82E-01	7.96E-02	2.31E-01	-1.15E-02	2.93E-01	9.54E-02	-1.15E-01	-1.77E-02	-1.29E-01	-2.46E-02	-1.42E-01	-4.03E-02	-1.71E-01	-4.44E-02
-8.12E-03	-1.77E-02	-8.72E-02	-8.54E-02	-2.28E-02	7.99E-02	1.25E-02	-1.06E-02	8.23E-02	8.12E-02	9.75E-02	-9.15E-03	1.84E-01	8.51E-02	2.25E-01	-2.08E-02	2.93E-01	9.56E-02	-1.26E-01	3.86E-03	-1.26E-01	-1.67E-02	-1.28E-01	-5.17E-02	-1.57E-01	-6.08E-02
-2.71E-02	-2.33E-02	-8.72E-02	-9.29E-02	-1.69E-02	6.32E-02	-1.32E-03	-1.45E-02	6.50E-02	7.56E-02	1.15E-01	-1.50E-02	1.82E-01	7.67E-02	2.30E-01	-1.08E-02	2.95E-01	9.72E-02	-1.14E-01	-1.55E-02	-1.27E-01	-2.27E-02	-1.41E-01	-3.67E-02	-1.71E-01	-4.04E-02
-2.39E-02	-1.63E-02	-9.08E-02	-9.44E-02	-1.75E-02	8.02E-02	4.35E-03	-1.16E-02	7.21E-02	8.65E-02	1.14E-01	-1.73E-02	1.90E-01	8.16E-02	2.28E-01	-1.44E-02	2.92E-01	8.78E-02	-1.16E-01	-3.01E-03	-1.32E-01	-7.70E-03	-1.49E-01	-2.41E-02	-1.74E-01	-4.19E-02
-1.74E-02	-2.04E-02	-9.54E-02	-1.01E-01	-1.43E-02	7.82E-02	8.93E-03	-1.33E-02	7.02E-02	9.18E-02	1.14E-01	-1.63E-02	1.85E-01	8.94E-02	2.24E-01	-1.35E-02	2.86E-01	1.05E-01	-1.21E-01	-4.82E-03	-1.33E-01	-1.97E-02	-1.44E-01	-5.19E-02	-1.68E-01	-7.05E-02
-3.37E-02	-1.27E-02	-9.51E-02	-8.68E-02	-2.50E-02	8.30E-02	2.29E-03	-1.34E-02	6.30E-02	8.87E-02	1.14E-01	-2.23E-02	1.86E-01	8.49E-02	2.31E-01	-1.56E-02	3.00E-01	9.68E-02	-1.31E-01	-4.77E-03	-1.27E-01	-2.34E-02	-1.35E-01	-4.10E-02	-1.66E-01	-4.59E-02
-2.28E-02	-1.84E-02	-9.35E-02	-8.99E-02	-2.50E-02	8.27E-02	-6.63E-04	-8.60E-03	7.58E-02	9.04E-02	1.13E-01	-1.82E-02	1.91E-01	9.08E-02	2.24E-01	-1.70E-02	2.89E-01	1.05E-01	-1.11E-01	-1.52E-02	-1.26E-01	-2.18E-02	-1.41E-01	-3.96E-02	-1.74E-01	-4.23E-02
-2.43E-02	-1.63E-02	-9.99E-02	-9.44E-02	-3.13E-02	8.01E-02	8.18E-03	-1.43E-02	5.95E-02	9.30E-02	1.23E-01	-1.51E-02	1.80E-01	9.18E-02	2.33E-01	-1.71E-02	2.87E-01	8.61E-02	-1.11E-01	-1.00E-02	-1.21E-01	-2.38E-02	-1.35E-01	-4.39E-02	-1.64E-01	-5.01E-02
-3.08E-02	-1.87E-02	-1.04E-01	-9.37E-02	-1.45E-02	8.00E-02	-2.79E-03	-1.71E-02	7.19E-02	8.76E-02	1.13E-01	-1.45E-02	1.92E-01	9.51E-02	2.25E-01	-9.47E-03	3.01E-01	1.05E-01	-1.27E-01	-1.59E-02	-1.32E-01	-3.26E-02	-1.46E-01	-5.13E-02	-1.79E-01	-5.61E-02
-3.43E-02	-1.39E-02	-1.11E-01	-9.13E-02	-1.80E-02	8.12E-02	1.06E-03	-1.73E-02	7.20E-02	8.86E-02	1.13E-01	-1.86E-02	1.93E-01	8.82E-02	2.30E-01	-9.33E-03	2.99E-01	1.02E-01	-1.21E-01	-1.44E-02	-1.32E-01	-2.42E-02	-1.47E-01	-3.76E-02	-1.74E-01	-4.18E-02
-2.50E-02	-1.67E-02	-1.04E-01	-8.95E-02	-1.78E-02	7.84E-02	6.55E-03	-1.47E-02	6.77E-02	8.84E-02	1.16E-01	-2.07E-02	1.91E-01	8.82E-02	2.35E-01	-1.66E-02	2.90E-01	9.55E-02	-1.10E-01	-1.13E-02	-1.24E-01	-1.89E-02	-1.43E-01	-3.55E-02	-1.76E-01	-4.43E-02
-1.34E-02	-1.28E-02	-9.12E-02	-8.92E-02	-2.63E-02	7.90E-02	1.13E-02	-6.04E-03	7.15E-02	9.25E-02	1.24E-01	-1.16E-02	1.86E-01	8.53E-02	2.28E-01	-1.73E-02	3.00E-01	9.46E-02	-1.18E-01	-2.03E-03	-1.25E-01	-1.96E-02	-1.38E-01	-4.85E-02	-1.71E-01	-5.90E-02
-2.25E-02	-1.86E-02	-9.49E-02	-8.87E-02	-3.01E-02	7.31E-02	1.34E-02	-1.51E-02	6.09E-02	7.54E-02	1.17E-01	-1.89E-02	1.82E-01	7.34E-02	2.33E-01	-1.80E-02	2.91E-01	8.57E-02	-1.09E-01	-9.55E-03	-1.25E-01	-1.46E-02	-1.43E-01	-3.19E-02	-1.74E-01	-4.31E-02
-2.93E-02	-1.88E-02	-8.99E-02	-9.00E-02	-2.20E-02	7.98E-02	5.92E-03	-1.57E-02	7.13E-02	8.29E-02	1.19E-01	-1.86E-02	1.82E-01	7.91E-02	2.37E-01	-1.60E-02	2.84E-01	9.11E-02	-1.30E-01	-1.98E-03	-1.35E-01	-1.87E-02	-1.38E-01	-4.87E-02	-1.62E-01	-5.57E-02
-2.95E-02	-1.46E-02	-9.93E-02	-9.28E-02	-2.80E-02	7.65E-02	4.07E-03	-1.35E-02	7.38E-02	8.93E-02	1.16E-01	-1.89E-02	1.89E-01	8.43E-02	2.34E-01	-1.20E-02	2.93E-01	1.00E-01	-1.16E-01	-1.16E-02	-1.27E-01	-2.41E-02	-1.42E-01	-4.29E-02	-1.71E-01	-5.32E-02

-1.53E-02	-1.19E-02	-1.00E-01	-9.82E-02	-2.44E-02	8.15E-02	1.12E-02	-1.08E-02	6.72E-02	9.53E-02	1.16E-01	-1.07E-02	1.94E-01	9.40E-02	2.27E-01	-1.62E-02	2.91E-01	1.01E-01	-1.19E-01	6.89E-04	-1.30E-01	-1.20E-02	-1.46E-01	-3.67E-02	-1.76E-01	-5.43E-02
-3.26E-02	-1.46E-02	-1.00E-01	-9.43E-02	-2.48E-02	7.95E-02	4.45E-03	-1.08E-02	7.12E-02	8.92E-02	1.16E-01	-1.84E-02	1.89E-01	8.54E-02	2.34E-01	-9.29E-03	2.91E-01	9.95E-02	-1.14E-01	-1.23E-02	-1.28E-01	-2.25E-02	-1.43E-01	-4.28E-02	-1.72E-01	-5.26E-02
-2.66E-02	-1.90E-02	-9.15E-02	-8.89E-02	-1.93E-02	8.12E-02	5.90E-03	-1.53E-02	7.29E-02	8.17E-02	1.19E-01	-1.69E-02	1.84E-01	7.96E-02	2.33E-01	-1.54E-02	2.86E-01	8.99E-02	-1.29E-01	1.54E-03	-1.36E-01	-1.58E-02	-1.36E-01	-4.95E-02	-1.58E-01	-5.88E-02
-2.92E-02	-1.63E-02	-1.00E-01	-8.81E-02	-1.71E-02	8.08E-02	1.19E-02	-1.46E-02	7.04E-02	8.30E-02	1.15E-01	-1.70E-02	1.79E-01	7.50E-02	2.29E-01	-1.28E-02	2.85E-01	8.51E-02	-1.27E-01	4.61E-06	-1.30E-01	-1.85E-02	-1.39E-01	-4.66E-02	-1.66E-01	-5.61E-02
-3.46E-02	-1.97E-02	-9.82E-02	-9.09E-02	-1.91E-02	8.05E-02	6.26E-03	-1.64E-02	7.39E-02	8.96E-02	1.15E-01	-1.08E-02	1.90E-01	8.30E-02	2.33E-01	-6.93E-03	2.97E-01	9.78E-02	-1.22E-01	-8.26E-03	-1.33E-01	-1.73E-02	-1.46E-01	-3.37E-02	-1.74E-01	-4.13E-02
-2.38E-02	-1.77E-02	-9.95E-02	-1.05E-01	-2.49E-02	7.54E-02	1.24E-02	-1.28E-02	6.54E-02	8.00E-02	1.07E-01	-1.67E-02	1.80E-01	8.00E-02	2.27E-01	-9.18E-03	2.88E-01	9.69E-02	-1.12E-01	-1.49E-02	-1.28E-01	-1.90E-02	-1.45E-01	-3.36E-02	-1.76E-01	-3.92E-02
-2.04E-02	-2.05E-02	-9.78E-02	-8.86E-02	-9.24E-03	7.56E-02	6.04E-03	-1.61E-02	8.63E-02	8.96E-02	1.14E-01	-1.81E-02	2.00E-01	9.29E-02	2.28E-01	-1.16E-02	2.97E-01	9.83E-02	-1.38E-01	3.21E-03	-1.43E-01	-1.12E-02	-1.50E-01	-3.73E-02	-1.73E-01	-5.40E-02
-2.04E-02	-1.89E-02	-8.91E-02	-9.35E-02	-2.38E-02	6.75E-02	1.23E-02	-1.25E-02	7.18E-02	8.45E-02	1.23E-01	-1.64E-02	1.83E-01	7.99E-02	2.28E-01	-9.23E-03	2.93E-01	8.96E-02	-1.09E-01	-1.58E-02	-1.23E-01	-2.12E-02	-1.40E-01	-3.77E-02	-1.72E-01	-4.55E-02
-1.60E-02	-2.13E-02	-9.86E-02	-8.73E-02	-2.05E-02	7.16E-02	9.63E-03	-1.32E-02	6.37E-02	8.02E-02	1.13E-01	-1.16E-02	1.91E-01	8.55E-02	2.29E-01	-1.42E-02	3.04E-01	9.84E-02	-1.14E-01	-5.32E-03	-1.30E-01	-1.29E-02	-1.44E-01	-4.05E-02	-1.79E-01	-4.71E-02
-1.26E-02	-1.45E-02	-9.25E-02	-9.79E-02	-2.80E-02	7.55E-02	1.45E-02	-1.14E-02	6.39E-02	8.46E-02	1.20E-01	-1.80E-02	1.82E-01	8.28E-02	2.34E-01	-1.30E-02	2.88E-01	9.31E-02	-1.11E-01	-5.24E-03	-1.25E-01	-1.39E-02	-1.44E-01	-3.51E-02	-1.76E-01	-5.41E-02
-2.34E-02	-1.99E-02	-9.22E-02	-9.90E-02	-2.08E-02	7.62E-02	9.81E-03	-1.61E-02	6.49E-02	8.92E-02	1.13E-01	-2.02E-02	1.86E-01	8.83E-02	2.26E-01	-1.04E-02	2.86E-01	9.78E-02	-1.15E-01	-1.56E-02	-1.29E-01	-2.31E-02	-1.46E-01	-4.50E-02	-1.82E-01	-5.15E-02
-3.92E-02	-1.59E-02	-9.24E-02	-8.78E-02	-2.77E-02	7.03E-02	1.00E-02	-1.61E-02	6.72E-02	7.63E-02	1.15E-01	-2.20E-02	1.82E-01	7.56E-02	2.41E-01	-1.45E-02	2.94E-01	9.03E-02	-1.35E-01	1.62E-03	-1.41E-01	-1.10E-02	-1.51E-01	-3.00E-02	-1.76E-01	-4.35E-02
-2.01E-02	-1.40E-02	-9.08E-02	-9.12E-02	-1.14E-02	7.82E-02	7.07E-03	-9.06E-03	7.89E-02	8.81E-02	1.15E-01	-1.24E-02	1.96E-01	8.02E-02	2.30E-01	-8.92E-03	2.98E-01	9.08E-02	-1.16E-01	-1.79E-02	-1.29E-01	-2.43E-02	-1.45E-01	-3.42E-02	-1.77E-01	-3.85E-02
-2.62E-02	-1.80E-02	-9.38E-02	-8.95E-02	-2.42E-02	8.34E-02	1.22E-02	-1.30E-02	6.25E-02	9.21E-02	1.20E-01	-1.46E-02	1.77E-01	8.99E-02	2.31E-01	-1.56E-02	2.85E-01	9.64E-02	-1.16E-01	-8.89E-03	-1.23E-01	-2.49E-02	-1.35E-01	-4.64E-02	-1.65E-01	-5.22E-02
-1.96E-02	-1.87E-02	-9.64E-02	-8.53E-02	-3.25E-02	7.22E-02	1.12E-02	-1.10E-02	6.50E-02	8.73E-02	1.20E-01	-1.73E-02	1.91E-01	8.26E-02	2.33E-01	-1.32E-02	2.91E-01	9.12E-02	-1.08E-01	-9.51E-03	-1.21E-01	-1.76E-02	-1.39E-01	-3.41E-02	-1.70E-01	-4.43E-02
-2.80E-02	-2.21E-02	-9.93E-02	-9.30E-02	-2.27E-02	6.92E-02	2.68E-03	-1.09E-02	6.67E-02	7.91E-02	1.08E-01	-2.11E-02	1.93E-01	7.72E-02	2.33E-01	-1.59E-02	2.97E-01	9.36E-02	-1.25E-01	-7.98E-03	-1.39E-01	-1.45E-02	-1.52E-01	-3.64E-02	-1.78E-01	-4.84E-02
-1.24E-02	-1.62E-02	-9.57E-02	-9.43E-02	-2.61E-02	7.33E-02	1.14E-02	-7.44E-03	6.48E-02	8.83E-02	1.18E-01	-1.68E-02	1.83E-01	8.67E-02	2.32E-01	-1.36E-02	2.89E-01	9.10E-02	-1.11E-01	-6.07E-03	-1.25E-01	-1.62E-02	-1.41E-01	-3.80E-02	-1.73E-01	-4.73E-02
-2.84E-02	-1.50E-02	-8.96E-02	-9.06E-02	-1.98E-02	7.73E-02	4.99E-03	-1.14E-02	6.72E-02	8.37E-02	1.12E-01	-1.47E-02	1.92E-01	7.84E-02	2.29E-01	-1.29E-02	3.01E-01	8.66E-02	-1.04E-01	-1.66E-02	-1.20E-01	-2.19E-02	-1.36E-01	-4.04E-02	-1.68E-01	-4.61E-02
-3.69E-02	-2.02E-02	-8.57E-02	-1.02E-01	-1.94E-02	7.95E-02	6.60E-04	-1.52E-02	6.41E-02	8.52E-02	1.09E-01	-2.07E-02	1.84E-01	8.42E-02	2.28E-01	-1.26E-02	2.87E-01	9.90E-02	-1.38E-01	8.03E-05	-1.42E-01	-1.65E-02	-1.50E-01	-4.76E-02	-1.79E-01	-6.04E-02
-3.06E-02	-1.64E-02	-9.63E-02	-8.62E-02	-2.90E-02	7.25E-02	-1.51E-03	-1.07E-02	6.50E-02	8.08E-02	1.10E-01	-1.65E-02	1.88E-01	7.86E-02	2.37E-01	-1.13E-02	3.03E-01	9.02E-02	-1.17E-01	-8.65E-03	-1.26E-01	-1.98E-02	-1.38E-01	-3.90E-02	-1.65E-01	-4.60E-02
-3.66E-02	-1.81E-02	-1.05E-01	-9.33E-02	-2.37E-02	7.40E-02	5.99E-03	-1.66E-02	6.55E-02	8.61E-02	1.18E-01	-1.72E-02	1.96E-01	8.75E-02	2.29E-01	-8.12E-03	3.13E-01	9.40E-02	-1.13E-01	-1.11E-02	-1.24E-01	-1.98E-02	-1.40E-01	-3.33E-02	-1.70E-01	-3.84E-02
-2.59E-02	-1.60E-02	-8.90E-02	-9.37E-02	-2.01E-02	8.34E-02	7.09E-03	-1.41E-02	6.06E-02	9.12E-02	1.08E-01	-1.72E-02	1.77E-01	8.77E-02	2.30E-01	-1.43E-02	2.84E-01	9.60E-02	-1.14E-01	-1.03E-02	-1.27E-01	-2.22E-02	-1.37E-01	-4.78E-02	-1.65E-01	-5.45E-02
-2.61E-02	-1.41E-02	-9.03E-02	-9.63E-02	-2.05E-02	7.84E-02	-1.73E-03	-8.46E-03	6.85E-02	8.72E-02	1.07E-01	-1.16E-02	1.90E-01	8.51E-02	2.26E-01	-9.17E-03	3.07E-01	9.37E-02	-1.27E-01	-5.79E-03	-1.35E-01	-1.90E-02	-1.42E-01	-4.27E-02	-1.61E-01	-4.59E-02
-3.52E-02	-1.35E-02	-9.54E-02	-8.46E-02	-1.45E-02	8.15E-02	1.07E-02	-1.19E-02	6.90E-02	8.61E-02	1.20E-01	-1.66E-02	1.87E-01	8.63E-02	2.31E-01	-1.22E-02	2.96E-01	9.63E-02	-1.14E-01	-2.39E-02	-1.25E-01	-3.26E-02	-1.40E-01	-4.42E-02	-1.70E-01	-4.63E-02
-2.76E-02	-1.52E-02	-1.08E-01	-8.54E-02	-1.87E-02	7.36E-02	-7.20E-04	-1.03E-02	7.02E-02	8.24E-02	1.08E-01	-2.19E-02	1.89E-01	7.54E-02	2.34E-01	-1.53E-02	2.98E-01	8.88E-02	-1.28E-01	-2.11E-03	-1.37E-01	-1.64E-02	-1.41E-01	-4.58E-02	-1.64E-01	-5.66E-02
-2.22E-02	-1.67E-02	-8.51E-02	-9.59E-02	-1.51E-02	8.00E-02	1.46E-02	-1.15E-02	6.91E-02	8.60E-02	1.14E-01	-1.79E-02	1.73E-01	7.94E-02	2.23E-01	-1.78E-02	2.80E-01	9.97E-02	-1.07E-01	2.05E-03	-1.22E-01	-1.02E-02	-1.30E-01	-4.35E-02	-1.60E-01	-6.52E-02
-3.58E-02	-2.22E-02	-8.46E-02	-9.89E-02	-1.81E-02	7.00E-02	-4.66E-03	-1.84E-02	6.83E-02	8.00E-02	1.09E-01	-2.11E-02	1.78E-01	8.36E-02	2.21E-01	-1.58E-02	2.90E-01	1.13E-01	-1.23E-01	7.83E-03	-1.31E-01	-6.21E-03	-1.40E-01	-3.28E-02	-1.66E-01	-5.27E-02
-2.18E-02	-2.21E-02	-9.79E-02	-8.72E-02	-2.32E-02	7.36E-02	8.72E-03	-1.60E-02	6.55E-02	8.51E-02	1.18E-01	-1.92E-02	1.83E-01	8.43E-02	2.34E-01	-9.90E-03	2.94E-01	9.86E-02	-1.11E-01	-7.27E-03	-1.25E-01	-1.43E-02	-1.44E-01	-2.91E-02	-1.76E-01	-4.27E-02
-2.39E-02	-1.33E-02	-9.31E-02	-9.53E-02	-2.73E-02	8.29E-02	8.94E-03	-7.36E-03	7.06E-02	8.65E-02	1.19E-01	-1.53E-02	1.84E-01	8.55E-02	2.31E-01	-8.95E-03	2.95E-01	9.34E-02	-1.08E-01	-9.96E-03	-1.25E-01	-1.44E-02	-1.42E-01	-3.26E-02	-1.72E-01	-4.62E-02
-2.77E-02	-1.45E-02	-9.10E-02	-9.86E-02	-1.64E-02	8.30E-02	6.83E-03	-1.09E-02	7.54E-02	9.21E-02	1.13E-01	-1.66E-02	1.77E-01	8.56E-02	2.29E-01	-1.42E-02	2.91E-01	9.73E-02	-1.22E-01	7.73E-03	-1.27E-01	-8.59E-03	-1.37E-01	-3.85E-02	-1.66E-01	-6.31E-02
-2.31E-02	-2.08E-02	-9.78E-02	-9.54E-02	-2.03E-02	7.67E-02	2.43E-02	-1.01E-02	6.77E-02	8.34E-02	1.17E-01	-1.54E-02	1.75E-01	8.26E-02	2.32E-01	-1.43E-02	2.94E-01	9.92E-02	-1.10E-01	-6.14E-03	-1.24E-01	-1.56E-02	-1.42E-01	-3.68E-02	-1.75E-01	-4.86E-02
-2.28E-02	-2.36E-02	-8.44E-02	-9.98E-02	-2.50E-02	7.76E-02	1.09E-02	-2.21E-02	6.07E-02	8.68E-02	1.16E-01	-2.39E-02	1.68E-01	8.36E-02	2.30E-01	-1.16E-02	2.84E-01	9.92E-02	-1.02E-01	-6.71E-03	-1.16E-01	-1.46E-02	-1.35E-01	-3.34E-02	-1.71E-01	-4.75E-02
-1.53E-02	-1.62E-02	-9.01E-02	-9.49E-02	-1.32E-02	7.40E-02	1.09E-02	-5.29E-03	6.76E-02	9.02E-02	1.14E-01	-1.45E-02	1.93E-01	8.79E-02	2.24E-01	-1.04E-02	2.90E-01	9.85E-02	-1.14E-01	-5.42E-03	-1.31E-01	-1.45E-02	-1.42E-01	-4.19E-02	-1.70E-01	-5.79E-02
-3.46E-02	-1.14E-02	-9.86E-02	-9.78E-02	-1.86E-02	8.45E-02	1.17E-02	-1.21E-02	7.26E-02	8.75E-02	1.14E-01	-1.48E-02	1.83E-01	8.64E-02	2.30E-01	-1.07E-02	2.93E-01	1.01E-01	-1.14E-01	4.13E-03	-1.22E-01	-1.03E-02	-1.38E-01	-3.64E-02	-1.68E-01	-5.83E-02
-3.38E-02	-1.81E-02	-9.61E-02	-8.90E-02	-1.83E-02	7.53E-02	5.02E-03	-1.13E-02	6.89E-02	8.07E-02	1.07E-01	-1.64E-02	1.87E-01	7.39E-02	2.29E-01	-9.77E-03	2.91E-01	9.46E-02	-1.09E-01	-8.29E-03	-1.23E-01	-1.71E-02	-1.39E-01	-3.64E-02	-1.71E-01	-4.73E-02
-2.11E-02	-1.19E-02	-9.02E-02	-9.22E-02	-2.59E-02	7.47E-02	9.28E-03	-1.43E-02	6.28E-02	8.12E-02	1.18E-01	-1.76E-02	1.75E-01	8.18E-02	2.35E-01	-1.66E-02	2.86E-01	8.75E-02	-1.08E-01	-4.34E-03	-1.19E-01	-1.63E-02	-1.35E-01	-3.56E-02	-1.67E-01	-4.68E-02
-2.49E-02	-2.18E-02	-9.95E-02	-9.05E-02	-2.13E-02	6.76E-02	5.77E-03	-1.61E-02	6.84E-02	8.07E-02	1.16E-01	-1.75E-02	1.86E-01	7.95E-02	2.28E-01	-5.36E-03	2.95E-01	9.71E-02	-1.11E-01	-1.02E-02	-1.28E-01	-1.57E-02	-1.45E-01	-3.41E-02	-1.77E-01	-4.44E-02
-1.38E-02	-1.88E-02	-1.03E-01	-8.68E-02	-1.86E-02	7.82E-02	9.86E-03	-1.37E-02	7.39E-02	8.28E-02	1.19E-01	-1.94E-02	1.82E-01	8.53E-02	2.25E-01	-1.48E-02	2.89E-01	1.02E-01	-1.12E-01	-5.44E-04	-1.28E-01	-1.04E-02	-1.42E-01	-3.60E-02	-1.73E-01	-5.00E-02
-1.77E-02	-1.84E-02	-9.17E-02	-7.95E-02	-2.36E-02	6.24E-02	1.39E-02	-1.38E-02	7.29E-02	7.41E-02	1.20E-01	-1.55E-02	1.89E-01	7.19E-02	2.33E-01	-6.41E-03	2.92E-01	9.96E-02	-1.12E-01	-1.27E-02	-1.26E-01	-1.85E-02	-1.41E-01	-3.10E-02	-1.71E-01	-3.73E-02
-2.35E-02	-1.62E-02	-1.06E-01	-9.24E-02	-2.89E-02	7.58E-02	5.99E-03	-1.26E-02	6.54E-02	8.38E-02	1.10E-01	-1.68E-02	1.76E-01	8.88E-02	2.27E-01	-7.27E-03	3.05E-01	1.02E-01	-1.09E-01	-3.47E-03	-1.27E-01	-9.40E-03	-1.44E-01	-3.08E-02	-1.73E-01	-4.89E-02
-2.98E-02	-1.92E-02	-8.54E-02	-8.65E-02	-2.15E-02	6.68E-02	7.40E-03	-1.47E-02	6.33E-02	7.26E-02	1.19E-01	-2.21E-02	1.87E-01	7.25E-02	2.33E-01	-1.09E-02	2.89E-01	9.35E-02	-1.06E-01	-8.15E-03	-1.23E-01	-1.31E-02	-1.39E-01	-3.21E-02	-1.72E-01	-4.30E-02
-1.46E-02	-1.99E-02	-8.26E-02	-8.88E-02	-2.38E-02	7.60E-02	1.04E-02	-1.63E-02	6.53E-02	8.13E-02	1.16E-01	-1.81E-02	1.81E-01	8.23E-02	2.30E-01	-1.28E-02	2.86E-01	9.27E-02	-1.10E-01	-9.03E-03	-1.24E-01	-1.97E-02	-1.39E-01	-4.50E-02	-1.70E-01	-5.85E-02
-2.99E-02	-1.38E-02	-9.08E-02	-9.62E-02	-9.30E-03	8.33E-02	7.85E-03	-1.47E-02	7.49E-02	9.13E-02	1.18E-01	-1.47E-02	1.92E-01	8.90E-02	2.27E-01	-1.70E-02	2.97E-01	9.40E-02	-1.12E-01	-3.86E-03	-1.27E-01	-1.51E-02	-1.45E-01	-4.20E-02	-1.75E-01	-6.77E-02
-2.18E-02	-1.47E-02	-9.19E-02	-9.24E-02	-2.37E-02	7.92E-02	6.00E-03	-1.20E-02	8.57E-02	8.94E-02	1.17E-01	-1.52E-02	1.89E-01	9.15E-02	2.29E-01	-1.14E-02	3.02E-01	9.68E-02	-1.07E-01	-4.68E-03	-1.22E-01	-1.27E-02	-1.38E-01	-3.46E-02	-1.72E-01	-4.93E-02
-2.43E-02	-2.12E-02	-9.31E-02	-9.13E-02	-1.68E-02	7.78E-02	1.19E-02	-1.32E-02	7.04E-02	8.18E-02	1.20E-01	-2.22E-02	1.96E-01	7.61E-02	2.25E-01	-1.14E-02	2.83E-01	9.41E-02	-1.19E-01	2.39E-03	-1.28E-01	-1.42E-02	-1.34E-01	-4.87E-02	-1.61E-01	-6.08E-02
-3.59E-02	-1.11E-02	-9.71E-02	-9.97E-02	-2.56E-02	7.53E-02	5.64E-03	-1.36E-02	7.20E-02	8.82E-02	1.15E-01	-1.67E-02	1.85E-01	8.66E-02	2.32E-01	-1.36E-02	2.96E-01	1.03E-01	-1.19E-01	4.20E-05	-1.32E-01	-1.11E-02	-1.46E-01	-3.61E-02	-1.76E-01	-5.49E-02
-1.82E-02	-1.51E-02	-9.78E-02	-8.24E-02	-2.34E-02	8.14E-02	1.43E-02	-1.38E-02	6.86E-02	8.38E-02	1.15E-01	-2.06E-02	1.96E-01	7.46E-02	2.27E-01	-1.69E-02	2.92E-01	1.00E-01	-1.23E-01	3.72E-03	-1.33E-01	-8.28E-03	-1.44E-01	-3.34E-02	-1.71E-01	-5.04E-02
-1.80E-02	-2.01E-02	-1.08E-01	-8.53E-02	-2.67E-02	8.12E-02	7.19E-03	-4.49E-03	7.19E-02	9.54E-02	1.17E-01	-8.67E-03	1.89E-01	9.94E-02	2.34E-01	-6.15E-04	3.06E-01	9.72E-02	-1.18E-01	-2.27E-02	-1.33E-01	-2.62E-02	-1.50E-01	-3.45E-02	-1.78E-01	-3.81E-02
-2.79E-02	-2.68E-02	-1.07E-01	-8.79E-02	-2.40E-02	7.42E-02	3.38E-03	-8.63E-03	7.24E-02	8.74E-02	1.18E-01	-1.00E-02	1.88E-01	8.95E-02	2.38E-01	4.41E-04	2.93E-01	8.92E-02	-1.29E-01	-3.12E-02	-1.42E-01	-3.33E-02	-1.56E-01	-3.93E-02	-1.82E-01	-3.84E-02
-2.72E-02	-2.57E-02	-1.07E-01	-8.61E-02	-2.13E-02	7.39E-02	2.42E-03	-9.51E-03	7.33E-02	8.81E-02	1.19E-01	-1.17E-02	1.90E-01	8.93E-02	2.37E-01	-2.22E-03	2.94E-01	8.97E-02	-1.31E-01	-2.82E-02	-1.43E-01	-3.31E-02	-1.56E-01	-4.06E-02	-1.80E-01	-4.12E-02
-4.97E-02	-1.01E-02	-8.46E-02	-9.70E-02	-1.31E-02	7.36E-02	5.31E-03	-8.69E-03	7.04E-02	9.13E-02	1.22E-01	-1.03E-02	1.85E-01	9.03E-02	2.31E-01	-9.96E-03	3.22E-01	8.59E-02	-1.30E-01	-6.71E-03	-1.37E-01	-1.94E-02	-1.45E-01	-3.43E-02	-1.69E-01	-4.11E-02
-2.67E-02	-1.66E-02	-9.85E-02	-8.81E-02	-1.80E-02	7.44E-02	7.03E-03	-4.98E-03	7.09E-02	8.59E-02	1.15E-01	-1.32E-03	2.02E-01	8.52E-02	2.37E-01	-4.99E-03	3.19E-01	8.40E-02	-1.31E-01	-1.04E-02	-1.42E-01	-1.84E-02	-1.52E-01	-3.15E-02	-1.72E-01	-3.59E-02
-1.25E-02	-1.38E-02	-7.73E-02	-9.05E-02	-2.26E-02	6.83E-02	1.84E-02	4.35E-03	7.81E-02	8.58E-02	1.24E-01	-3.94E-03	1.98E-01	9.35E-02	2.36E-01	-1.04E-02	3.00E-01	9.06E-02	-1.12E-01	-8.38E-03	-1.24E-01	-1.47E-02	-1.38E-01	-3.00E-02	-1.66E-01	-4.32E-02
-9.79E-03	-1.49E-02	-9.20E-02	-7.45E-02	-3.43E-02	7.45E-02	2.23E-02	2.60E-03	6.45E-02	9.24E-02	1.16E-01	-8.19E-03	1.86E-01	9.09E-02	2.44E-01	-4.13E-03	3.03E-01	8.96E-02	-1.05E-01	-2.51E-02	-1.19E-01	-2.89E-02	-1.36E-01	-3.69E-02	-1.65E-01	-3.86E-02
-2.81E-02	-1.92E-02	-1.01E-01	-8.80E-02	-2.12E-02	7.84E-02	-7.63E-03	-7.66E-03	7.51E-02	8.71E-02	1.13E-01	-1.51E-02	1.95E-01	8.27E-02	2.35E-01	-1.40E-02	3.09E-01	8.79E-02	-1.29E-01	-1.29E-02	-1.41E-01	-2.05E-02	-1.55E-01	-3.15E-02	-1.78E-01	-3.95E-02
-2.96E-02	-1.28E-02	-9.63E-02	-8.55E-02	-1.44E-02	8.18E-02	6.66E-03	-1.18E-03	7.25E-02	8.88E-02	1.26E-01	-1.15E-03	1.85E-01	8.50E-02	2.69E-01	6.65E-03	2.98E-01	8.44E-02	-1.24E-01	-3.83E-02	-1.36E-01	-3.51E-02	-1.50E-01	-3.83E-02	-1.77E-01	-4.03E-02
-2.22E-02	-1.03E-02	-8.69E-02	-9.50E-02	-1.24E-02	6.82E-02	1.04E-02	-5.27E-03	7.71E-02	7.88E-02	1.24E-01	-9.43E-03	1.85E-01	8.49E-02	2.38E-01	-5.29E-03	2.88E-01	8.97E-02	-1.26E-01	-6.16E-03	-1.39E-01	-1.58E-02	-1.51E-01	-3.36E-02	-1.76E-01	-4.29E-02
-2.48E-02	-1.15E-02	-9.69E-02	-8.50E-02	-1.16E-02	7.57E-02	-3.12E-03	-2.65E-03	8.51E-02	8.80E-02	1.20E-01	-7.47E-03	1.90E-01	8.88E-02	2.34E-01	-5.73E-03	2.84E-01	9.24E-02	-1.22E-01	-1.24E-02	-1.33E-01	-2.48E-02	-1.45E-01	-4.67E-02	-1.73E-01	-5.40E-02
-1.65E-02	-1.67E-02	-9.73E-02	-9.42E-02	-1.68E-02	7.90E-02	1.27E-02	-6.84E-03	7.07E-02	9.17E-02	1.22E-01	-7.73E-03	1.92E-01	9.76E-02	2.36E-01	-8.51E-03	2.98E-01	9.53E-02	-1.30E-01	-2.24E-02	-1.43E-01	-2.49E-02	-1.56E-01	-3.52E-02	-1.78E-01	-4.03E-02
-1.44E-02	-1.30E-02	-9.53E-02	-9.49E-02	-1.38E-02	7.37E-02	1.39E-02	-6.44E-03	6.60E-02	9.25E-02	1.24E-01	-1.12E-02	1.75E-01	9.61E-02	2.33E-01	-1.11E-03	2.94E-01	9.74E-02	-1.28E-01	-1.38E-02	-1.40E-01	-2.29E-02	-1.52E-01	-3.66E-02	-1.76E-01	-4.43E-02
-2.36E-02	-1.90E-02	-1.04E-01	-8.67E-02	-2.51E-02	6.96E-02	4.98E-03	-1.45E-02	9.84E-02	9.05E-02	1.27E-01	-1.20E-02	1.99E-01	9.63E-02	2.36E-01	-5.88E-03	2.90E-01	9.51E-02	-1.28E-01	-1.04E-02	-1.40E-01	-1.81E-02	-1.55E-01	-2.54E-02	-1.81E-01	-3.18E-02
-2.31E-02	-1.82E-02	-1.03E-01	-8.94E-02	-2.88E-02	7.51E-02	1.25E-03	-8.67E-03	7.32E-02	8.73E-02	1.28E-01	-1.44E-02	1.93E-01	9.22E-02	2.33E-01	-6.55E-03	3.01E-01	9.06E-02	-1.33E-01	-1.48E-02	-1.46E-01	-1.60E-02	-1.57E-01	-2.31E-02	-1.75E-01	-2.83E-02
-2.40E-02	-5.93E-03	-1.04E-01	-8.97E-02	-2.12E-02	7.98E-02	4.63E-03	3.01E-03	7.42E-02	9.72E-02	1.25E-01	-3.32E-03	1.85E-01	9.84E-02	2.34E-01	1.29E-04	3.06E-01	9.03E-02	-1.25E-01	-1.36E-02	-1.39E-01	-1.71E-02	-1.53E-01	-2.54E-02	-1.76E-01	-2.89E-02
-4.11E-02	-1.17E-02	-1.01E-01	-9.06E-02	-2.04E-02	8.48E-02	5.97E-03	-7.05E-03	6.58E-02	9.26E-02	1.18E-01	-1.25E-02	1.87E-01	9.24E-02	2.32E-01	-1.06E-02	2.98E-01	9.01E-02	-1.29E-01	-1.63E-02	-1.41E-01	-2.48E-02	-1.51E-01	-4.31E-02	-1.67E-01	-4.87E-02
-2.46E-02	-6.28E-03	-1.05E-01	-8.57E-02	-2.13E-02	7.90E-02	7.91E-03	1.15E-03	7.60E-02	9.71E-02	1.23E-01	-2.28E-03	1.87E-01	9.73E-02	2.36E-01	-1.59E-04	3.03E-01	9.39E-02	-1.26E-01	-1.09E-02	-1.39E-01	-1.71E-02	-1.53E-01	-2.60E-02	-1.76E-01	-3.02E-02
-2.48E-02	-1.89E-02	-9.87E-02	-9.25E-02	-2.21E-02	6.39E-02	6.73E-03	-5.77E-04	7.51E-02	8.39E-02	1.22E-01	-9.69E-03	1.86E-01	8.81E-02	2.36E-01	-4.51E-03	2.95E-01	9.69E-02	-1.24E-01	-3.10E-02	-1.37E-01	-2.70E-02	-1.50E-01	-3.17E-02	-1.75E-01	-3.17E-02
-1.79E-02	-2.06E-02	-9.98E-02	-8.81E-02	-2.07E-02	7.71E-02	1.27E-02	-6.12E-03	8.01E-02	9.41E-02	1.26E-01	-4.93E-03	1.87E-01	9.59E-02	2.33E-01	-3.22E-03	2.89E-01	1.03E-01	-1.26E-01	-2.67E-02	-1.39E-01	-2.76E-02	-1.54E-01	-3.57E-02	-1.83E-01	-3.95E-02
-2.24E-02	-2.69E-02	-9.62E-02	-8.06E-02	-3.92E-02	7.75E-02	-1.00E-03	-1.32E-02	6.28E-02	8.86E-02	1.29E-01	-1.48E-02	1.86E-01	9.16E-02	2.39E-01	-9.92E-03	3.04E-01	9.11E-02	-1.24E-01	-3.12E-02	-1.35E-01	-2.68E-02	-1.46E-01	-2.88E-02	-1.68E-01	-2.89E-02
-3.37E-02	-1.81E-02	-1.17E-01	-7.88E-02	-2.97E-02	7.47E-02	4.69E-04	-5.56E-03	7.47E-02	9.49E-02	1.23E-01	-3.46E-03	1.90E-01	1.00E-01	2.33E-01	-3.93E-03	2.93E-01	1.00E-01	-1.32E-01	-3.65E-02	-1.43E-01	-3.44E-02	-1.54E-01	-3.71E-02	-1.73E-01	-3.50E-02
-2.93E-02	-1.82E-02	-8.65E-02	-8.94E-02	-3.18E-02	7.44E-02	6.74E-03	-1.63E-02	7.22E-02	9.06E-02	1.27E-01	-8.76E-03	1.84E-01	8.52E-02	2.41E-01	-9.05E-03	2.90E-01	8.88E-02	-1.17E-01	-1.14E-02	-1.28E-01	-1.91E-02	-1.38E-01	-3.48E-02	-1.61E-01	-3.96E-02
-1.79E-02	-1.24E-02	-9.73E-02	-8.94E-02	-3.62E-02	8.37E-02	-9.47E-04	4.12E-03	7.33E-02	9.79E-02	1.18E-01	-6.36E-03	1.94E-01	8.92E-02	2.41E-01	-8.44E-03	2.96E-01	9.64E-02	-1.11E-01	-7.66E-03	-1.24E-01	-1.33E-02	-1.40E-01	-3.12E-02	-1.69E-01	-4.55E-02
-3.04E-02	-2.20E-02	-1.05E-01	-9.30E-02	-2.24E-02	7.51E-02	8.31E-03	-1.17E-02	7.54E-02	8.93E-02	1.24E-01	-1.10E-02	1.87E-01	9.16E-02	2.32E-01	-1.58E-03	2.87E-01	1.03E-01	-1.24E-01	-2.51E-02	-1.39E-01	-2.37E-02	-1.57E-01	-3.00E-02	-1.83E-01	-3.82E-02
-1.70E-02	-2.73E-02	-9.52E-02	-9.32E-02	-2.94E-02	4.51E-02	1.72E-02	-5.10E-03	6.79E-02	8.74E-02	1.21E-01	-9.96E-03	1.89E-01	8.56E-02	2.31E-01	-4.14E-03	2.89E-01	9.75E-02	-1.14E-01	-2.17E-02	-1.29E-01	-2.37E-02	-1.49E-01	-3.45E-02	-1.81E-01	-4.35E-02
-1.55E-02	-1.10E-02	-9.14E-02	-8.72E-02	-5.66E-03	8.16E-02	1.24E-02	-1.98E-03	9.46E-02	9.73E-02	1.18E-01	-1.34E-02	1.89E-01	9.26E-02	2.28E-01	-7.56E-03	3.00E-01	9.78E-02	-1.25E-01	-1.30E-02	-1.36E-01	-2.41E-02	-1.49E-01	-3.54E-02	-1.76E-01	-4.35E-02
-3.16E-02	-1.11E-02	-9.35E-02	-8.72E-02	-2.14E-02	8.02E-02	-3.24E-03	-1.70E-03	7.25E-02	9.70E-02	1.17E-01	-6.98E-03	1.92E-01	9.37E-02	2.33E-01	-3.43E-03	2.94E-01	8.66E-02	-1.21E-01	-2.91E-02	-1.36E-01	-3.03E-02	-1.50E-01	-3.85E-02	-1.74E-01	-4.52E-02
-1.39E-02	-1.29E-02	-1.12E-01	-9.21E-02	-1.13E-02	7.26E-02	9.73E-03	-8.52E-04	7.92E-02	8.15E-02	1.21E-01	-4.65E-03	1.88E-01	8.29E-02	2.36E-01	-2.30E-03	2.98E-01	9.44E-02	-1.26E-01	-1.38E-02	-1.42E-01	-1.64E-02	-1.59E-01	-2.66E-02	-1.84E-01	-3.20E-02
-7.45E-03	-9.07E-03	-9.42E-02	-9.35E-02	-2.54E-02	8.33E-02	3.58E-02	-2.27E-03	7.78E-02	9.48E-02	1.25E-01	-8.13E-03	1.95E-01	8.84E-02	2.32E-01	-2.33E-03	2.83E-01	8.76E-02	-1.13E-01	-1.92E-02	-1.25E-01	-2.75E-02	-1.41E-01	-3.68E-02	-1.71E-01	-4.11E-02
-1.64E-02	-1.11E-02	-1.06E-01	-9.27E-02	-1.10E-02	8.89E-02	1.03E-02	2.80E-03	7.80E-02	1.04E-01	1.17E-01	-1.05E-03	1.99E-01	1.04E-01	2.24E-01	-6.38E-04	3.11E-01	9.86E-02	-1.16E-01	-3.04E-02	-1.31E-01	-3.73E-02	-1.48E-01	-4.83E-02	-1.77E-01	-5.07E-02
-2.56E-02	-2.39E-02	-1.02E-01	-8.73E-02	-2.35E-02	5.86E-02	1.38E-02	-1.23E-02	6.71E-02	7.93E-02	1.16E-01	-1.29E-02	1.80E-01	8.41E-02	2.38E-01	-3.43E-03	2.88E-01	9.33E-02	-1.26E-01	-1.56E-02	-1.40E-01	-1.88E-02	-1.55E-01	-2.86E-02	-1.80E-01	-3.14E-02
-2.27E-02	-2.31E-02	-1.02E-01	-8.34E-02	-1.56E-02	7.32E-02	1.43E-02	-1.35E-02	7.29E-02	8.98E-02	1.26E-01	-1.28E-02	1.88E-01	8.85E-02	2.36E-01	-4.85E-03	2.94E-01	9.84E-02	-1.22E-01	-2.06E-02	-1.36E-01	-2.67E-02	-1.51E-01	-3.64E-02	-1.76E-01	-3.81E-02

PrCoor_x21	PrCoor_y21	PrCoor_x22	PrCoor_y22	PrCoor_x23	PrCoor_y23	PrCoor_x24	PrCoor_y24	PrCoor_x25	PrCoor_y25	PrCoor_x26	PrCoor_y26	PrCoor_x27	PrCoor_y27	PrCoor_x28	PrCoor_y28	PrCoor_x29	PrCoor_y29	PrCoor_x30	PrCoor_y30
-1.94E-01	-2.78E-02	-2.06E-01	-4.01E-03	-2.07E-01	2.37E-02	-1.91E-01	4.68E-02	-1.67E-01	6.41E-02	-1.41E-01	7.63E-02	-1.15E-01	7.80E-02	-1.01E-01	6.70E-02	-9.68E-02	4.97E-02	-8.16E-02	4.36E-02
-1.95E-01	-2.80E-02	-2.06E-01	-4.48E-03	-2.06E-01	2.23E-02	-1.89E-01	4.42E-02	-1.63E-01	6.17E-02	-1.38E-01	7.38E-02	-1.13E-01	7.39E-02	-1.00E-01	6.79E-02	-9.66E-02	5.21E-02	-8.40E-02	4.43E-02
-1.93E-01	-3.97E-02	-2.08E-01	-1.98E-02	-2.07E-01	8.14E-03	-1.94E-01	3.59E-02	-1.70E-01	5.59E-02	-1.43E-01	6.66E-02	-1.16E-01	6.57E-02	-1.00E-01	4.93E-02	-8.96E-02	3.73E-02	-7.38E-02	2.90E-02
-1.99E-01	-3.77E-02	-2.11E-01	-1.64E-02	-2.09E-01	1.06E-02	-1.92E-01	3.63E-02	-1.64E-01	5.50E-02	-1.35E-01	6.90E-02	-1.09E-01	7.28E-02	-9.48E-02	5.91E-02	-8.87E-02	4.64E-02	-7.46E-02	4.02E-02
-2.06E-01	-2.46E-02	-2.21E-01	-2.40E-03	-2.19E-01	2.35E-02	-1.99E-01	4.63E-02	-1.68E-01	6.23E-02	-1.36E-01	7.16E-02	-1.07E-01	6.87E-02	-8.96E-02	4.92E-02	-7.94E-02	3.16E-02	-6.45E-02	2.02E-02
-1.98E-01	-3.13E-02	-2.16E-01	-8.44E-03	-2.17E-01	1.97E-02	-1.98E-01	4.41E-02	-1.68E-01	6.27E-02	-1.37E-01	7.43E-02	-1.08E-01	7.62E-02	-9.39E-02	6.27E-02	-9.01E-02	4.59E-02	-7.50E-02	4.15E-02
-2.00E-01	-3.30E-02	-2.05E-01	-8.90E-03	-2.03E-01	1.98E-02	-1.85E-01	4.28E-02	-1.57E-01	5.58E-02	-1.27E-01	6.26E-02	-1.02E-01	6.47E-02	-9.18E-02	5.29E-02	-9.14E-02	3.85E-02	-8.16E-02	3.40E-02
-1.98E-01	-2.60E-02	-2.10E-01	-5.74E-03	-2.10E-01	1.94E-02	-1.95E-01	4.18E-02	-1.70E-01	5.75E-02	-1.42E-01	6.47E-02	-1.15E-01	6.22E-02	-9.70E-02	4.68E-02	-8.39E-02	3.49E-02	-6.63E-02	2.65E-02
-1.93E-01	-3.31E-02	-2.08E-01	-9.19E-03	-2.11E-01	1.78E-02	-1.95E-01	4.02E-02	-1.67E-01	5.52E-02	-1.40E-01	6.74E-02	-1.18E-01	7.42E-02	-1.07E-01	6.47E-02	-1.02E-01	4.75E-02	-8.66E-02	4.19E-02
-2.00E-01	-3.12E-02	-2.15E-01	-7.94E-03	-2.14E-01	2.06E-02	-1.97E-01	4.58E-02	-1.69E-01	6.16E-02	-1.37E-01	6.79E-02	-1.07E-01	6.28E-02	-9.07E-02	5.18E-02	-8.48E-02	4.06E-02	-7.24E-02	3.79E-02
-1.92E-01	-2.03E-02	-2.05E-01	-6.16E-04	-2.04E-01	2.26E-02	-1.90E-01	4.28E-02	-1.69E-01	5.67E-02	-1.45E-01	6.15E-02	-1.23E-01	5.80E-02	-1.08E-01	4.48E-02	-1.01E-01	2.76E-02	-8.47E-02	2.25E-02
-1.94E-01	-1.76E-02	-2.08E-01	1.38E-03	-2.09E-01	2.35E-02	-1.96E-01	4.28E-02	-1.76E-01	5.79E-02	-1.52E-01	6.62E-02	-1.28E-01	5.98E-02	-1.07E-01	4.56E-02	-9.06E-02	3.56E-02	-6.77E-02	3.25E-02
-1.79E-01	-1.59E-02	-1.91E-01	3.30E-03	-1.94E-01	2.53E-02	-1.84E-01	4.37E-02	-1.68E-01	5.69E-02	-1.48E-01	6.26E-02	-1.27E-01	6.11E-02	-1.09E-01	5.16E-02	-9.68E-02	4.06E-02	-8.23E-02	3.43E-02
-1.91E-01	-3.19E-02	-2.05E-01	-9.65E-03	-2.04E-01	1.78E-02	-1.89E-01	4.31E-02	-1.66E-01	6.20E-02	-1.40E-01	7.23E-02	-1.15E-01	7.24E-02	-9.78E-02	6.09E-02	-8.77E-02	4.91E-02	-7.59E-02	3.98E-02
-1.92E-01	-3.89E-02	-2.09E-01	-9.37E-03	-2.13E-01	2.28E-02	-1.93E-01	4.78E-02	-1.61E-01	6.57E-02	-1.27E-01	7.75E-02	-1.01E-01	8.27E-02	-9.20E-02	7.03E-02	-9.46E-02	4.74E-02	-8.40E-02	3.57E-02
-1.94E-01	-4.81E-02	-2.11E-01	-2.39E-02	-2.13E-01	1.16E-02	-1.99E-01	4.45E-02	-1.69E-01	6.53E-02	-1.39E-01	7.51E-02	-1.18E-01	7.59E-02	-1.10E-01	6.45E-02	-1.09E-01	4.80E-02	-1.04E-01	3.17E-02
-1.98E-01	-3.26E-02	-2.09E-01	-1.11E-02	-2.06E-01	1.56E-02	-1.91E-01	4.02E-02	-1.65E-01	5.57E-02	-1.38E-01	6.79E-02	-1.13E-01	7.16E-02	-9.81E-02	5.84E-02	-9.01E-02	4.30E-02	-7.44E-02	3.64E-02
-1.95E-01	-2.28E-02	-2.04E-01	-1.79E-03	-2.02E-01	2.21E-02	-1.83E-01	4.06E-02	-1.57E-01	5.71E-02	-1.31E-01	7.13E-02	-1.06E-01	7.07E-02	-9.08E-02	5.66E-02	-8.38E-02	3.99E-02	-7.06E-02	3.07E-02
-1.94E-01	-3.82E-02	-2.12E-01	-1.38E-02	-2.15E-01	1.52E-02	-1.97E-01	4.07E-02	-1.68E-01	6.15E-02	-1.39E-01	7.54E-02	-1.12E-01	7.66E-02	-9.61E-02	6.47E-02	-9.03E-02	4.50E-02	-7.54E-02	3.55E-02
-2.11E-01	-3.35E-02	-2.17E-01	-7.15E-03	-2.10E-01	2.53E-02	-1.89E-01	5.22E-02	-1.55E-01	6.37E-02	-1.20E-01	6.77E-02	-9.24E-02	6.57E-02	-8.18E-02	5.35E-02	-8.57E-02	3.04E-02	-6.91E-02	2.19E-02
-2.02E-01	-4.10E-02	-2.14E-01	-1.64E-02	-2.13E-01	1.28E-02	-1.92E-01	3.76E-02	-1.61E-01	5.53E-02	-1.28E-01	6.61E-02	-1.01E-01	7.11E-02	-8.93E-02	6.25E-02	-8.67E-02	4.32E-02	-7.09E-02	3.77E-02
-2.06E-01	-3.34E-02	-2.18E-01	-7.45E-03	-2.17E-01	2.23E-02	-1.96E-01	4.62E-02	-1.64E-01	5.91E-02	-1.29E-01	6.42E-02	-1.00E-01	6.28E-02	-8.70E-02	4.69E-02	-8.59E-02	2.63E-02	-7.02E-02	1.82E-02
-1.97E-01	-2.72E-02	-2.11E-01	-5.74E-03	-2.12E-01	1.87E-02	-1.95E-01	3.85E-02	-1.69E-01	5.46E-02	-1.40E-01	6.50E-02	-1.13E-01	6.71E-02	-9.61E-02	5.21E-02	-8.58E-02	3.80E-02	-6.70E-02	3.36E-02
-1.98E-01	-3.29E-02	-2.06E-01	-1.11E-02	-2.01E-01	1.69E-02	-1.82E-01	4.21E-02	-1.54E-01	5.86E-02	-1.24E-01	6.83E-02	-1.01E-01	7.09E-02	-9.17E-02	5.99E-02	-9.30E-02	4.19E-02	-8.40E-02	3.23E-02
-2.01E-01	-3.04E-02	-2.13E-01	-7.05E-03	-2.12E-01	2.03E-02	-1.93E-01	4.35E-02	-1.63E-01	5.89E-02	-1.30E-01	6.55E-02	-1.02E-01	6.65E-02	-8.88E-02	5.72E-02	-8.60E-02	4.16E-02	-7.58E-02	3.17E-02
-1.92E-01	-2.80E-02	-2.05E-01	-4.99E-03	-2.09E-01	2.17E-02	-1.96E-01	4.34E-02	-1.72E-01	5.79E-02	-1.45E-01	6.74E-02	-1.19E-01	6.82E-02	-1.02E-01	5.39E-02	-9.04E-02	4.00E-02	-7.28E-02	3.21E-02
-1.93E-01	-2.62E-02	-2.02E-01	-6.20E-03	-1.98E-01	1.86E-02	-1.82E-01	4.16E-02	-1.62E-01	6.03E-02	-1.39E-01	7.06E-02	-1.18E-01	7.08E-02	-1.07E-01	5.57E-02	-1.02E-01	3.86E-02	-8.63E-02	2.98E-02
-1.87E-01	-1.15E-02	-1.95E-01	7.03E-03	-1.94E-01	2.82E-02	-1.83E-01	4.65E-02	-1.64E-01	5.80E-02	-1.43E-01	6.43E-02	-1.23E-01	5.70E-02	-1.09E-01	4.03E-02	-9.58E-02	2.91E-02	-7.83E-02	2.23E-02
-1.93E-01	-2.37E-02	-2.05E-01	-4.03E-03	-2.03E-01	2.03E-02	-1.91E-01	4.38E-02	-1.72E-01	6.10E-02	-1.48E-01	6.88E-02	-1.24E-01	6.90E-02	-1.08E-01	5.39E-02	-9.35E-02	4.32E-02	-7.29E-02	3.73E-02
-1.95E-01	-2.44E-02	-2.06E-01	-2.85E-03	-2.06E-01	2.22E-02	-1.94E-01	4.40E-02	-1.72E-01	5.73E-02	-1.46E-01	6.34E-02	-1.19E-01	5.88E-02	-9.76E-02	5.09E-02	-8.38E-02	4.60E-02	-6.48E-02	4.46E-02
-1.90E-01	-2.46E-02	-2.02E-01	-4.30E-03	-2.04E-01	1.95E-02	-1.93E-01	3.95E-02	-1.72E-01	5.22E-02	-1.48E-01	6.10E-02	-1.25E-01	6.38E-02	-1.09E-01	5.48E-02	-9.83E-02	4.80E-02	-8.21E-02	4.35E-02
-1.94E-01	-2.67E-02	-2.09E-01	-4.65E-03	-2.11E-01	2.09E-02	-1.97E-01	4.26E-02	-1.72E-01	5.63E-02	-1.44E-01	6.44E-02	-1.18E-01	6.70E-02	-1.03E-01	5.72E-02	-9.67E-02	4.38E-02	-7.96E-02	3.87E-02
-1.94E-01	-2.04E-02	-2.07E-01	-2.09E-04	-2.07E-01	2.29E-02	-1.92E-01	4.23E-02	-1.67E-01	5.70E-02	-1.42E-01	6.73E-02	-1.19E-01	6.99E-02	-1.06E-01	5.95E-02	-9.98E-02	4.63E-02	-8.61E-02	3.93E-02
-1.97E-01	-3.40E-02	-2.05E-01	-1.24E-02	-2.03E-01	1.56E-02	-1.89E-01	4.12E-02	-1.66E-01	5.86E-02	-1.43E-01	6.90E-02	-1.23E-01	7.16E-02	-1.09E-01	5.28E-02	-9.22E-02	3.97E-02	-6.96E-02	3.16E-02
-1.95E-01	-2.49E-02	-2.09E-01	-3.54E-03	-2.09E-01	2.11E-02	-1.94E-01	4.20E-02	-1.70E-01	5.81E-02	-1.44E-01	6.95E-02	-1.20E-01	7.11E-02	-1.04E-01	5.72E-02	-9.43E-02	4.23E-02	-7.71E-02	3.49E-02
-1.85E-01	-1.88E-02	-1.92E-01	3.48E-04	-1.92E-01	2.18E-02	-1.80E-01	3.94E-02	-1.65E-01	5.43E-02	-1.45E-01	6.17E-02	-1.24E-01	6.25E-02	-1.08E-01	5.35E-02	-9.55E-02	4.54E-02	-7.73E-02	4.07E-02
-1.97E-01	-3.10E-02	-2.12E-01	-1.01E-02	-2.08E-01	1.57E-02	-1.88E-01	4.19E-02	-1.63E-01	6.49E-02	-1.32E-01	7.57E-02	-1.06E-01	8.04E-02	-9.16E-02	7.28E-02	-8.58E-02	5.60E-02	-7.35E-02	4.71E-02
-1.90E-01	-2.07E-02	-2.00E-01	-2.86E-03	-2.00E-01	1.84E-02	-1.87E-01	3.79E-02	-1.68E-01	5.40E-02	-1.45E-01	6.19E-02	-1.23E-01	6.58E-02	-1.08E-01	5.16E-02	-9.26E-02	4.24E-02	-7.16E-02	3.65E-02
-1.60E-01	-3.05E-02	-1.75E-01	-1.02E-02	-1.83E-01	1.44E-02	-1.81E-01	3.68E-02	-1.72E-01	5.42E-02	-1.56E-01	6.57E-02	-1.39E-01	7.22E-02	-1.27E-01	6.12E-02	-1.13E-01	5.61E-02	-9.29E-02	5.50E-02
-1.98E-01	-2.24E-02	-2.14E-01	-1.39E-03	-2.15E-01	2.28E-02	-2.00E-01	4.44E-02	-1.74E-01	5.95E-02	-1.44E-01	6.51E-02	-1.15E-01	6.39E-02	-9.64E-02	4.70E-02	-8.09E-02	3.60E-02	-5.99E-02	2.91E-02
-1.93E-01	-2.79E-02	-2.02E-01	-4.60E-03	-2.03E-01	2.32E-02	-1.92E-01	4.64E-02	-1.72E-01	5.98E-02	-1.50E-01	6.56E-02	-1.29E-01	6.48E-02	-1.15E-01	4.89E-02	-1.05E-01	3.46E-02	-8.63E-02	2.71E-02
-1.92E-01	-1.26E-02	-2.02E-01	7.16E-03	-2.03E-01	2.97E-02	-1.92E-01	4.76E-02	-1.72E-01	5.74E-02	-1.50E-01	6.01E-02	-1.27E-01	5.07E-02	-1.07E-01	4.32E-02	-9.36E-02	3.98E-02	-7.46E-02	3.88E-02
-2.04E-01	-2.48E-02	-2.16E-01	-4.02E-03	-2.02E-01	1.94E-02	-1.90E-01	5.01E-02	-1.76E-01	7.30E-02	-1.51E-01	7.35E-02	-1.22E-01	6.40E-02	-9.95E-02	4.90E-02	-8.61E-02	3.46E-02	-6.62E-02	2.76E-02
-1.81E-01	-2.10E-02	-1.92E-01	6.26E-04	-1.90E-01	2.43E-02	-1.79E-01	4.67E-02	-1.64E-01	6.46E-02	-1.46E-01	7.47E-02	-1.27E-01	7.11E-02	-1.12E-01	5.27E-02	-9.86E-02	3.57E-02	-8.14E-02	2.35E-02
-1.89E-01	-1.97E-02	-1.97E-01	1.16E-03	-1.99E-01	2.44E-02	-1.86E-01	4.19E-02	-1.65E-01	5.43E-02	-1.41E-01	6.32E-02	-1.17E-01	6.58E-02	-1.01E-01	5.58E-02	-8.97E-02	4.77E-02	-7.46E-02	4.21E-02
-1.95E-01	-2.67E-02	-1.98E-01	-4.57E-03	-1.97E-01	2.09E-02	-1.83E-01	4.16E-02	-1.62E-01	5.50E-02	-1.38E-01	6.07E-02	-1.14E-01	5.82E-02	-9.88E-02	5.76E-02	-9.19E-02	5.83E-02	-7.74E-02	5.67E-02
-1.93E-01	-2.77E-02	-2.06E-01	-5.13E-03	-2.05E-01	2.13E-02	-1.89E-01	4.51E-02	-1.67E-01	6.50E-02	-1.40E-01	7.48E-02	-1.14E-01	7.63E-02	-9.82E-02	6.44E-02	-9.07E-02	4.80E-02	-7.64E-02	3.91E-02
-1.91E-01	-3.08E-02	-2.05E-01	-7.92E-03	-2.07E-01	1.88E-02	-1.96E-01	4.12E-02	-1.76E-01	5.46E-02	-1.52E-01	5.90E-02	-1.28E-01	5.86E-02	-1.11E-01	4.85E-02	-9.95E-02	3.94E-02	-8.09E-02	3.66E-02
-1.90E-01	-2.55E-02	-2.03E-01	-4.13E-03	-2.04E-01	2.04E-02	-1.91E-01	4.17E-02	-1.71E-01	5.90E-02	-1.46E-01	6.96E-02	-1.23E-01	7.36E-02	-1.07E-01	5.87E-02	-9.27E-02	4.41E-02	-7.23E-02	3.91E-02
-1.83E-01	-1.72E-02	-1.97E-01	3.07E-03	-2.01E-01	2.59E-02	-1.90E-01	4.49E-02	-1.72E-01	5.97E-02	-1.52E-01	7.05E-02	-1.32E-01	7.34E-02	-1.17E-01	5.88E-02	-1.02E-01	4.86E-02	-8.32E-02	4.20E-02
-1.96E-01	-2.32E-02	-2.09E-01	5.70E-05	-2.11E-01	2.69E-02	-1.97E-01	4.90E-02	-1.73E-01	6.33E-02	-1.46E-01	6.95E-02	-1.21E-01	6.83E-02	-1.04E-01	5.68E-02	-9.51E-02	4.59E-02	-7.96E-02	3.82E-02
-1.86E-01	-1.90E-02	-1.95E-01	1.34E-03	-1.95E-01	2.41E-02	-1.85E-01	4.26E-02	-1.67E-01	5.45E-02	-1.48E-01	6.16E-02	-1.29E-01	6.34E-02	-1.14E-01	5.22E-02	-1.02E-01	3.89E-02	-8.47E-02	3.30E-02
-1.97E-01	-2.23E-02	-2.04E-01	-1.83E-03	-2.01E-01	2.41E-02	-1.88E-01	4.69E-02	-1.64E-01	6.04E-02	-1.39E-01	6.89E-02	-1.16E-01	6.84E-02	-1.02E-01	5.67E-02	-9.65E-02	4.20E-02	-8.41E-02	3.33E-02
-1.89E-01	-1.70E-02	-2.04E-01	5.23E-03	-2.09E-01	3.00E-02	-1.98E-01	5.00E-02	-1.77E-01	6.26E-02	-1.54E-01	6.78E-02	-1.29E-01	6.18E-02	-1.10E-01	5.20E-02	-9.67E-02	4.38E-02	-7.85E-02	3.79E-02
-1.97E-01	-2.94E-02	-2.10E-01	-7.97E-03	-2.10E-01	1.85E-02	-1.91E-01	4.19E-02	-1.66E-01	6.06E-02	-1.37E-01	6.87E-02	-1.12E-01	7.00E-02	-1.01E-01	6.29E-02	-1.01E-01	4.36E-02	-8.48E-02	3.68E-02
-1.91E-01	-1.69E-02	-2.01E-01	1.08E-03	-1.96E-01	2.33E-02	-1.84E-01	4.52E-02	-1.64E-01	5.95E-02	-1.41E-01	6.70E-02	-1.19E-01	6.82E-02	-1.07E-01	5.77E-02	-1.02E-01	4.56E-02	-8.68E-02	3.93E-02
-1.93E-01	-2.22E-02	-2.05E-01	3.43E-04	-2.07E-01	2.67E-02	-1.94E-01	4.81E-02	-1.71E-01	6.06E-02	-1.45E-01	6.56E-02	-1.21E-01	6.63E-02	-1.07E-01	4.95E-02	-9.76E-02	3.22E-02	-8.03E-02	2.56E-02
-2.01E-01	-1.97E-02	-2.15E-01	-1.70E-03	-2.12E-01	2.08E-02	-1.96E-01	4.18E-02	-1.69E-01	5.45E-02	-1.40E-01	6.33E-02	-1.15E-01	6.46E-02	-1.01E-01	4.50E-02	-8.70E-02	3.11E-02	-6.79E-02	2.21E-02
-1.94E-01	-2.69E-02	-2.03E-01	-3.49E-03	-2.05E-01	2.28E-02	-1.89E-01	4.43E-02	-1.65E-01	5.98E-02	-1.38E-01	6.87E-02	-1.16E-01	7.28E-02	-1.05E-01	6.46E-02	-1.01E-01	4.94E-02	-8.82E-02	4.28E-02
-1.95E-01	-3.40E-02	-2.08E-01	-6.24E-03	-2.11E-01	2.63E-02	-2.02E-01	5.33E-02	-1.79E-01	6.79E-02	-1.52E-01	7.38E-02	-1.25E-01	6.99E-02	-1.07E-01	5.35E-02	-9.58E-02	3.72E-02	-7.63E-02	2.89E-02
-2.02E-01	-2.77E-02	-2.15E-01	-7.89E-03	-2.10E-01	1.57E-02	-1.88E-01	3.63E-02	-1.57E-01	5.29E-02	-1.25E-01	6.32E-02	-9.88E-02	6.47E-02	-9.00E-02	5.15E-02	-9.45E-02	3.35E-02	-8.15E-02	2.40E-02
-2.00E-01	-1.96E-02	-2.11E-01	-2.28E-03	-2.06E-01	1.72E-02	-1.86E-01	3.42E-02	-1.59E-01	4.75E-02	-1.31E-01	5.55E-02	-1.07E-01	5.80E-02	-9.47E-02	4.99E-02	-9.12E-02	3.57E-02	-7.76E-02	3.00E-02
-2.01E-01	-2.42E-02	-2.14E-01	-2.68E-03	-2.11E-01	2.22E-02	-1.93E-01	4.31E-02	-1.63E-01	5.29E-02	-1.32E-01	6.17E-02	-1.06E-01	6.44E-02	-9.32E-02	5.16E-02	-8.98E-02	3.33E-02	-7.54E-02	2.60E-02
-1.86E-01	-2.05E-02	-1.96E-01	-1.83E-03	-1.96E-01	1.95E-02	-1.83E-01	3.67E-02	-1.62E-01	4.99E-02	-1.39E-01	6.06E-02	-1.18E-01	6.45E-02	-1.05E-01	4.86E-02	-8.92E-02	3.95E-02	-6.68E-02	3.46E-02
-2.00E-01	-2.70E-02	-2.09E-01	-9.35E-03	-2.06E-01	1.21E-02	-1.87E-01	3.10E-02	-1.61E-01	4.66E-02	-1.31E-01	5.49E-02	-1.06E-01	5.84E-02	-9.39E-02	4.98E-02	-9.16E-02	3.54E-02	-7.59E-02	3.25E-02
-1.88E-01	-2.06E-02	-1.98E-01	-3.60E-03	-1.94E-01	1.59E-02	-1.81E-01	3.50E-02	-1.63E-01	5.12E-02	-1.42E-01	6.08E-02	-1.22E-01	6.29E-02	-1.08E-01	5.15E-02	-9.99E-02	3.71E-02	-8.36E-02	3.00E-02
-1.91E-01	-2.05E-02	-2.01E-01	-1.91E-03	-2.02E-01	2.01E-02	-1.89E-01	3.74E-02	-1.66E-01	4.97E-02	-1.42E-01	6.05E-02	-1.20E-01	6.45E-02	-1.06E-01	5.03E-02	-9.49E-02	3.68E-02	-7.64E-02	2.99E-02
-1.94E-01	-2.45E-02	-1.98E-01	-7.57E-03	-1.91E-01	1.50E-02	-1.76E-01	3.73E-02	-1.56E-01	5.35E-02	-1.35E-01	6.16E-02	-1.15E-01	5.93E-02	-1.05E-01	4.40E-02	-1.01E-01	2.80E-02	-9.17E-02	1.88E-02
-1.98E-01	-3.74E-02	-2.14E-01	-1.56E-02	-2.14E-01	1.24E-02	-1.98E-01	3.94E-02	-1.73E-01	6.06E-02	-1.46E-01	7.52E-02	-1.18E-01	7.31E-02	-9.73E-02	5.59E-02	-8.12E-02	4.41E-02	-5.77E-02	4.32E-02
-1.96E-01	-2.83E-02	-2.12E-01	-5.52E-03	-2.16E-01	2.14E-02	-2.03E-01	4.40E-02	-1.79E-01	6.05E-02	-1.53E-01	6.89E-02	-1.27E-01	6.65E-02	-1.09E-01	4.67E-02	-8.93E-02	3.62E-02	-6.55E-02	3.04E-02
-1.95E-01	-2.40E-02	-2.03E-01	-6.52E-03	-1.99E-01	1.63E-02	-1.85E-01	3.84E-02	-1.62E-01	5.35E-02	-1.42E-01	6.69E-02	-1.20E-01	6.09E-02	-1.02E-01	4.50E-02	-8.79E-02	3.45E-02	-6.59E-02	3.28E-02
-1.99E-01	-3.07E-02	-2.15E-01	-8.85E-03	-2.13E-01	1.90E-02	-1.98E-01	4.59E-02	-1.70E-01	6.19E-02	-1.40E-01	7.10E-02	-1.15E-01	7.52E-02	-9.94E-02	5.41E-02	-8.16E-02	4.16E-02	-5.78E-02	3.96E-02
-2.00E-01	-3.30E-02	-2.13E-01	-1.01E-02	-2.13E-01	1.88E-02	-1.96E-01	4.42E-02	-1.68E-01	6.23E-02	-1.41E-01	7.68E-02	-1.16E-01	7.77E-02	-1.01E-01	5.84E-02	-9.20E-02	3.85E-02	-7.14E-02	3.23E-02
-1.94E-01	-4.13E-02	-2.10E-01	-6.68E-03	-2.21E-01	2.92E-02	-2.06E-01	5.72E-02	-1.74E-01	7.39E-02	-1.41E-01	8.38E-02	-1.10E-01	7.79E-02	-9.13E-02	5.90E-02	-8.35E-02	3.84E-02	-6.75E-02	2.67E-02
-2.00E-01	-2.68E-02	-2.11E-01	-6.92E-03	-2.11E-01	1.87E-02	-1.97E-01	4.15E-02	-1.73E-01	5.99E-02	-1.45E-01	6.94E-02	-1.17E-01	6.66E-02	-9.56E-02	5.18E-02	-7.93E-02	4.17E-02	-5.71E-02	3.76E-02
-1.88E-01	-4.26E-02	-2.18E-01	-1.57E-02	-2.28E-01	2.07E-02	-2.12E-01	5.44E-02	-1.78E-01	7.52E-02	-1.40E-01	8.40E-02	-1.09E-01	8.46E-02	-9.62E-02	6.59E-02	-9.67E-02	4.01E-02	-8.73E-02	2.13E-02
-2.04E-01	-3.34E-02	-2.24E-01	-1.28E-02	-2.25E-01	1.41E-02	-2.07E-01	3.99E-02	-1.75E-01	5.68E-02	-1.39E-01	6.16E-02	-1.05E-01	5.00E-02	-8.04E-02	4.06E-02	-6.63E-02	4.35E-02	-4.78E-02	4.64E-02
-1.91E-01	-1.47E-02	-1.97E-01	1.94E-03	-1.96E-01	2.32E-02	-1.85E-01	4.17E-02	-1.67E-01	5.28E-02	-1.49E-01	6.23E-02	-1.29E-01	5.86E-02	-1.13E-01	4.43E-02	-9.65E-02	3.66E-02	-7.77E-02	2.91E-02
-2.00E-01	-2.37E-02	-2.08E-01	-6.00E-03	-2.05E-01	1.65E-02	-1.88E-01	3.65E-02	-1.61E-01	4.89E-02	-1.34E-01	5.84E-02	-1.10E-01	6.25E-02	-9.82E-02	5.43E-02	-9.42E-02	4.02E-02	-7.84E-02	3.56E-02

-1.94E-01	-1.96E-02	-2.06E-01	-3.81E-03	-2.06E-01	1.61E-02	-1.92E-01	3.52E-02	-1.69E-01	5.05E-02	-1.45E-01	6.32E-02	-1.20E-01	6.28E-02	-1.01E-01	5.07E-02	-8.88E-02	3.88E-02	-7.04E-02	3.40E-02
-1.93E-01	-2.25E-02	-2.00E-01	-5.19E-03	-1.98E-01	1.69E-02	-1.85E-01	3.70E-02	-1.66E-01	5.26E-02	-1.46E-01	6.31E-02	-1.22E-01	6.03E-02	-1.04E-01	5.34E-02	-9.13E-02	5.01E-02	-7.39E-02	4.66E-02
-1.97E-01	-2.88E-02	-2.09E-01	-8.79E-03	-2.07E-01	1.70E-02	-1.93E-01	4.05E-02	-1.67E-01	5.57E-02	-1.38E-01	6.36E-02	-1.12E-01	6.49E-02	-9.81E-02	5.23E-02	-9.20E-02	4.13E-02	-7.65E-02	4.05E-02
-1.99E-01	-3.00E-02	-2.12E-01	-7.43E-03	-2.12E-01	2.02E-02	-1.93E-01	4.36E-02	-1.65E-01	6.08E-02	-1.35E-01	7.15E-02	-1.10E-01	7.22E-02	-9.76E-02	5.68E-02	-9.64E-02	3.70E-02	-7.94E-02	2.97E-02
-1.96E-01	-2.66E-02	-2.12E-01	-5.96E-03	-2.14E-01	1.95E-02	-1.98E-01	4.18E-02	-1.73E-01	5.98E-02	-1.46E-01	6.96E-02	-1.21E-01	6.76E-02	-1.06E-01	5.05E-02	-1.01E-01	3.19E-02	-8.42E-02	2.54E-02
-2.07E-01	-3.53E-02	-2.22E-01	-1.57E-02	-2.20E-01	1.13E-02	-1.98E-01	3.75E-02	-1.71E-01	6.19E-02	-1.41E-01	7.50E-02	-1.12E-01	7.31E-02	-9.37E-02	5.74E-02	-8.63E-02	3.73E-02	-6.85E-02	3.11E-02
-1.98E-01	-3.93E-02	-2.11E-01	-1.70E-02	-2.12E-01	1.16E-02	-1.94E-01	3.72E-02	-1.68E-01	5.92E-02	-1.39E-01	7.08E-02	-1.08E-01	6.30E-02	-8.70E-02	5.13E-02	-7.67E-02	4.51E-02	-6.50E-02	3.79E-02
-1.96E-01	-2.97E-02	-2.13E-01	-6.71E-03	-2.16E-01	2.02E-02	-1.99E-01	4.28E-02	-1.70E-01	5.84E-02	-1.40E-01	6.73E-02	-1.11E-01	6.39E-02	-9.14E-02	5.04E-02	-8.08E-02	3.54E-02	-6.25E-02	2.97E-02
-1.91E-01	-1.77E-02	-2.05E-01	-2.77E-04	-2.00E-01	1.99E-02	-1.86E-01	4.18E-02	-1.66E-01	5.94E-02	-1.44E-01	6.70E-02	-1.23E-01	6.43E-02	-1.09E-01	4.53E-02	-9.11E-02	3.50E-02	-7.04E-02	2.73E-02
-2.06E-01	-2.81E-02	-2.17E-01	-4.63E-03	-2.14E-01	2.43E-02	-1.93E-01	4.81E-02	-1.62E-01	6.34E-02	-1.31E-01	7.19E-02	-1.05E-01	7.10E-02	-9.58E-02	5.24E-02	-9.91E-02	3.03E-02	-8.00E-02	2.33E-02
-2.08E-01	-4.52E-02	-2.21E-01	-1.72E-02	-2.18E-01	1.80E-02	-1.98E-01	4.93E-02	-1.62E-01	6.82E-02	-1.27E-01	8.26E-02	-9.80E-02	8.43E-02	-8.44E-02	7.32E-02	-8.15E-02	5.21E-02	-6.90E-02	4.02E-02
-2.10E-01	-2.66E-02	-2.27E-01	-5.70E-03	-2.25E-01	1.89E-02	-2.01E-01	4.02E-02	-1.68E-01	5.92E-02	-1.33E-01	6.75E-02	-1.04E-01	7.11E-02	-8.92E-02	5.31E-02	-8.31E-02	3.26E-02	-6.46E-02	2.42E-02
-1.98E-01	-2.82E-02	-2.09E-01	-6.90E-03	-2.10E-01	1.92E-02	-1.97E-01	4.17E-02	-1.72E-01	5.37E-02	-1.44E-01	6.37E-02	-1.18E-01	6.49E-02	-9.87E-02	4.88E-02	-8.23E-02	3.85E-02	-6.12E-02	3.38E-02
-1.97E-01	-3.69E-02	-2.11E-01	-1.39E-02	-2.12E-01	1.55E-02	-1.99E-01	4.22E-02	-1.71E-01	5.73E-02	-1.39E-01	6.59E-02	-1.11E-01	6.87E-02	-9.61E-02	6.04E-02	-9.19E-02	4.34E-02	-7.34E-02	3.90E-02
-1.94E-01	-3.50E-02	-1.98E-01	-1.42E-02	-1.94E-01	1.31E-02	-1.74E-01	3.70E-02	-1.54E-01	6.01E-02	-1.32E-01	7.23E-02	-1.12E-01	7.50E-02	-1.05E-01	5.83E-02	-1.05E-01	3.85E-02	-9.03E-02	3.02E-02
-2.07E-01	-3.44E-02	-2.24E-01	-9.64E-03	-2.21E-01	2.03E-02	-1.98E-01	4.69E-02	-1.67E-01	6.97E-02	-1.33E-01	7.97E-02	-1.05E-01	7.99E-02	-9.24E-02	5.42E-02	-8.50E-02	2.90E-02	-6.29E-02	1.83E-02
-2.01E-01	-2.63E-02	-2.09E-01	-9.65E-03	-2.04E-01	1.22E-02	-1.85E-01	3.29E-02	-1.59E-01	5.03E-02	-1.32E-01	6.05E-02	-1.08E-01	6.44E-02	-9.35E-02	5.21E-02	-8.51E-02	4.09E-02	-6.81E-02	3.45E-02
-1.96E-01	-2.48E-02	-2.03E-01	-6.62E-03	-2.00E-01	1.67E-02	-1.86E-01	3.79E-02	-1.63E-01	5.17E-02	-1.41E-01	6.07E-02	-1.20E-01	6.12E-02	-1.11E-01	5.67E-02	-1.07E-01	4.99E-02	-9.83E-02	3.91E-02
-2.00E-01	-3.45E-02	-2.07E-01	-1.39E-02	-2.07E-01	1.44E-02	-1.92E-01	4.01E-02	-1.66E-01	5.63E-02	-1.38E-01	6.68E-02	-1.13E-01	6.92E-02	-9.98E-02	5.13E-02	-9.14E-02	3.41E-02	-7.27E-02	2.80E-02
-1.92E-01	-3.93E-02	-2.04E-01	-1.22E-02	-2.08E-01	2.17E-02	-1.98E-01	5.09E-02	-1.75E-01	6.84E-02	-1.47E-01	7.61E-02	-1.21E-01	7.56E-02	-1.04E-01	6.04E-02	-9.37E-02	4.59E-02	-7.67E-02	3.86E-02
-2.00E-01	-2.23E-02	-2.13E-01	-1.92E-03	-2.13E-01	2.29E-02	-2.01E-01	4.49E-02	-1.77E-01	5.59E-02	-1.48E-01	6.09E-02	-1.19E-01	5.83E-02	-9.84E-02	4.50E-02	-8.42E-02	3.53E-02	-6.22E-02	3.18E-02
-1.98E-01	-2.31E-02	-2.09E-01	-3.07E-03	-2.09E-01	2.09E-02	-1.94E-01	3.99E-02	-1.68E-01	5.24E-02	-1.40E-01	6.10E-02	-1.14E-01	6.11E-02	-9.84E-02	4.49E-02	-8.80E-02	2.98E-02	-6.98E-02	2.31E-02
-1.89E-01	-2.59E-02	-1.98E-01	-4.93E-03	-1.97E-01	2.13E-02	-1.85E-01	4.52E-02	-1.66E-01	6.13E-02	-1.44E-01	6.88E-02	-1.23E-01	6.90E-02	-1.10E-01	5.40E-02	-1.01E-01	3.86E-02	-8.46E-02	3.42E-02
-1.97E-01	-2.63E-02	-2.11E-01	-6.16E-03	-2.08E-01	1.77E-02	-1.92E-01	4.19E-02	-1.64E-01	5.69E-02	-1.35E-01	6.78E-02	-1.09E-01	7.20E-02	-9.43E-02	5.63E-02	-8.61E-02	3.95E-02	-6.92E-02	3.39E-02
-2.11E-01	-3.53E-02	-2.24E-01	-8.16E-03	-2.17E-01	2.31E-02	-1.90E-01	4.88E-02	-1.50E-01	6.19E-02	-1.09E-01	6.95E-02	-7.90E-02	7.10E-02	-7.11E-02	6.02E-02	-7.84E-02	3.81E-02	-7.29E-02	2.59E-02
-2.10E-01	-2.50E-02	-2.19E-01	5.03E-03	-2.18E-01	3.65E-02	-1.93E-01	5.03E-02	-1.57E-01	6.21E-02	-1.26E-01	7.92E-02	-9.85E-02	8.09E-02	-8.61E-02	5.91E-02	-8.38E-02	3.41E-02	-7.05E-02	2.29E-02
-1.84E-01	-2.50E-02	-1.98E-01	-4.88E-03	-1.95E-01	1.85E-02	-1.81E-01	4.14E-02	-1.64E-01	6.15E-02	-1.42E-01	7.01E-02	-1.19E-01	6.95E-02	-1.03E-01	5.68E-02	-9.51E-02	4.18E-02	-7.73E-02	3.69E-02
-1.94E-01	-2.73E-02	-2.09E-01	-5.15E-03	-2.08E-01	2.33E-02	-1.93E-01	4.95E-02	-1.71E-01	6.81E-02	-1.45E-01	7.51E-02	-1.21E-01	7.60E-02	-1.06E-01	6.10E-02	-9.60E-02	4.59E-02	-7.72E-02	3.77E-02
-1.96E-01	-3.03E-02	-2.10E-01	-1.13E-02	-2.09E-01	1.41E-02	-1.95E-01	3.89E-02	-1.72E-01	5.70E-02	-1.44E-01	6.66E-02	-1.19E-01	7.19E-02	-1.01E-01	5.96E-02	-8.80E-02	4.93E-02	-6.86E-02	4.50E-02
-1.92E-01	-3.50E-02	-2.09E-01	-1.18E-02	-2.14E-01	1.65E-02	-2.01E-01	4.17E-02	-1.76E-01	6.14E-02	-1.49E-01	7.60E-02	-1.23E-01	8.13E-02	-1.05E-01	6.51E-02	-9.13E-02	4.79E-02	-7.03E-02	4.23E-02
-1.97E-01	-3.21E-02	-2.15E-01	-6.05E-03	-2.21E-01	2.35E-02	-2.05E-01	4.79E-02	-1.79E-01	6.52E-02	-1.47E-01	7.06E-02	-1.17E-01	6.92E-02	-9.67E-02	5.18E-02	-8.26E-02	3.80E-02	-6.03E-02	3.17E-02
-1.98E-01	-3.07E-02	-2.07E-01	-4.60E-03	-2.06E-01	2.59E-02	-1.90E-01	5.13E-02	-1.66E-01	6.75E-02	-1.38E-01	7.39E-02	-1.11E-01	6.72E-02	-9.44E-02	5.44E-02	-8.71E-02	3.89E-02	-7.54E-02	2.86E-02
-1.98E-01	-3.65E-02	-2.14E-01	-1.01E-02	-2.12E-01	2.31E-02	-1.95E-01	5.38E-02	-1.67E-01	7.42E-02	-1.36E-01	8.46E-02	-1.10E-01	8.48E-02	-9.47E-02	6.70E-02	-8.83E-02	4.66E-02	-7.89E-02	3.26E-02
-1.91E-01	-2.26E-02	-2.03E-01	-1.39E-03	-2.02E-01	2.32E-02	-1.85E-01	4.46E-02	-1.61E-01	6.14E-02	-1.36E-01	7.24E-02	-1.15E-01	7.48E-02	-1.05E-01	5.64E-02	-9.80E-02	3.60E-02	-8.76E-02	2.18E-02
-1.98E-01	-3.58E-02	-2.08E-01	-7.37E-03	-2.09E-01	2.52E-02	-1.90E-01	5.10E-02	-1.59E-01	6.63E-02	-1.26E-01	7.30E-02	-9.98E-02	7.55E-02	-8.94E-02	6.65E-02	-9.08E-02	4.68E-02	-8.04E-02	3.56E-02
-1.96E-01	-2.13E-02	-2.04E-01	-2.28E-03	-2.04E-01	2.22E-02	-1.91E-01	4.39E-02	-1.70E-01	5.98E-02	-1.46E-01	6.75E-02	-1.23E-01	6.47E-02	-1.07E-01	4.67E-02	-8.98E-02	3.72E-02	-7.02E-02	2.98E-02
-1.97E-01	-2.81E-02	-2.12E-01	5.85E-04	-2.16E-01	3.22E-02	-2.00E-01	5.63E-02	-1.70E-01	6.80E-02	-1.37E-01	7.15E-02	-1.06E-01	6.66E-02	-9.12E-02	5.37E-02	-8.98E-02	3.45E-02	-7.29E-02	2.74E-02
-1.95E-01	-2.41E-02	-2.08E-01	-5.88E-03	-2.09E-01	1.66E-02	-1.96E-01	3.71E-02	-1.72E-01	5.25E-02	-1.45E-01	6.17E-02	-1.18E-01	5.97E-02	-9.92E-02	5.05E-02	-8.74E-02	4.73E-02	-6.88E-02	4.45E-02
-1.86E-01	-1.84E-02	-1.97E-01	-6.97E-04	-2.00E-01	2.05E-02	-1.88E-01	3.87E-02	-1.70E-01	5.49E-02	-1.47E-01	6.31E-02	-1.25E-01	6.33E-02	-1.08E-01	5.00E-02	-9.44E-02	3.95E-02	-7.74E-02	3.38E-02
-2.03E-01	-2.99E-02	-2.18E-01	-1.07E-02	-2.15E-01	1.42E-02	-1.96E-01	3.87E-02	-1.68E-01	5.77E-02	-1.37E-01	6.97E-02	-1.09E-01	7.28E-02	-9.33E-02	5.92E-02	-8.61E-02	4.21E-02	-6.74E-02	3.85E-02
-1.87E-01	-1.87E-02	-2.01E-01	1.20E-03	-2.04E-01	2.44E-02	-1.90E-01	4.37E-02	-1.66E-01	5.81E-02	-1.41E-01	6.69E-02	-1.17E-01	6.74E-02	-1.04E-01	5.21E-02	-9.63E-02	3.66E-02	-8.48E-02	2.88E-02
-2.00E-01	-3.92E-02	-2.12E-01	-1.58E-02	-2.05E-01	1.44E-02	-1.86E-01	4.53E-02	-1.58E-01	6.87E-02	-1.28E-01	8.14E-02	-1.03E-01	8.22E-02	-9.17E-02	6.83E-02	-9.21E-02	4.72E-02	-8.10E-02	3.55E-02
-1.96E-01	-2.37E-02	-2.06E-01	6.00E-04	-2.06E-01	2.95E-02	-1.94E-01	5.39E-02	-1.71E-01	6.57E-02	-1.45E-01	7.16E-02	-1.21E-01	6.48E-02	-1.03E-01	4.35E-02	-8.80E-02	2.65E-02	-6.62E-02	1.84E-02
-2.02E-01	-2.40E-02	-2.17E-01	-3.78E-03	-2.15E-01	2.10E-02	-1.99E-01	4.45E-02	-1.70E-01	5.98E-02	-1.39E-01	6.83E-02	-1.10E-01	6.32E-02	-9.03E-02	4.79E-02	-7.77E-02	3.78E-02	-6.04E-02	3.38E-02
-1.97E-01	-2.75E-02	-2.01E-01	-4.88E-03	-1.97E-01	2.33E-02	-1.84E-01	4.75E-02	-1.62E-01	6.19E-02	-1.36E-01	6.60E-02	-1.13E-01	6.26E-02	-1.03E-01	5.02E-02	-1.02E-01	3.39E-02	-8.77E-02	2.75E-02
-1.97E-01	-2.94E-02	-2.14E-01	-2.54E-03	-2.15E-01	2.79E-02	-1.97E-01	5.25E-02	-1.69E-01	6.93E-02	-1.41E-01	8.05E-02	-1.16E-01	7.75E-02	-1.05E-01	5.20E-02	-9.66E-02	2.67E-02	-7.63E-02	1.71E-02
-1.90E-01	-2.74E-02	-1.99E-01	-5.94E-03	-1.95E-01	2.01E-02	-1.78E-01	4.43E-02	-1.58E-01	6.33E-02	-1.36E-01	7.39E-02	-1.14E-01	7.25E-02	-9.98E-02	6.24E-02	-9.28E-02	5.18E-02	-8.75E-02	3.90E-02
-1.83E-01	-3.15E-02	-1.94E-01	-1.02E-02	-1.93E-01	1.60E-02	-1.81E-01	4.09E-02	-1.63E-01	6.11E-02	-1.40E-01	7.22E-02	-1.18E-01	7.38E-02	-1.03E-01	5.99E-02	-9.42E-02	4.44E-02	-7.61E-02	4.22E-02
-2.02E-01	-3.18E-02	-2.14E-01	-1.04E-02	-2.16E-01	1.55E-02	-2.00E-01	3.74E-02	-1.74E-01	5.41E-02	-1.48E-01	6.63E-02	-1.19E-01	6.09E-02	-9.75E-02	4.60E-02	-8.06E-02	3.89E-02	-5.83E-02	3.32E-02
-1.96E-01	-2.25E-02	-2.05E-01	-5.38E-03	-2.03E-01	1.62E-02	-1.89E-01	3.66E-02	-1.65E-01	5.10E-02	-1.39E-01	5.93E-02	-1.15E-01	6.11E-02	-9.81E-02	4.99E-02	-8.75E-02	4.03E-02	-6.94E-02	3.79E-02
-2.00E-01	-2.96E-02	-2.15E-01	-9.92E-03	-2.17E-01	1.43E-02	-2.02E-01	3.60E-02	-1.77E-01	5.28E-02	-1.49E-01	6.47E-02	-1.21E-01	6.62E-02	-1.01E-01	5.32E-02	-8.58E-02	4.55E-02	-6.46E-02	4.15E-02
-2.00E-01	-3.97E-02	-2.13E-01	-1.55E-02	-2.09E-01	1.41E-02	-1.88E-01	4.16E-02	-1.58E-01	6.32E-02	-1.25E-01	7.44E-02	-9.90E-02	7.97E-02	-8.86E-02	7.06E-02	-9.05E-02	4.87E-02	-7.61E-02	3.94E-02
-1.98E-01	-3.57E-02	-2.09E-01	-1.38E-02	-2.02E-01	1.42E-02	-1.86E-01	4.21E-02	-1.58E-01	5.87E-02	-1.31E-01	7.18E-02	-1.07E-01	7.09E-02	-9.99E-02	5.90E-02	-1.08E-01	3.85E-02	-9.20E-02	3.02E-02
-1.88E-01	-1.58E-02	-1.99E-01	1.81E-03	-1.99E-01	2.31E-02	-1.90E-01	4.25E-02	-1.75E-01	5.59E-02	-1.56E-01	6.23E-02	-1.36E-01	6.16E-02	-1.21E-01	4.69E-02	-1.04E-01	3.90E-02	-8.19E-02	3.38E-02
-1.92E-01	-2.08E-02	-2.05E-01	-1.31E-03	-2.08E-01	2.02E-02	-1.97E-01	3.75E-02	-1.75E-01	4.93E-02	-1.50E-01	5.67E-02	-1.26E-01	5.89E-02	-1.10E-01	4.49E-02	-9.45E-02	3.80E-02	-7.39E-02	3.12E-02
-1.86E-01	-2.58E-02	-1.95E-01	-5.89E-03	-1.94E-01	1.82E-02	-1.81E-01	4.03E-02	-1.63E-01	5.92E-02	-1.42E-01	7.11E-02	-1.19E-01	7.04E-02	-1.01E-01	6.00E-02	-8.79E-02	5.02E-02	-7.29E-02	4.26E-02
-2.05E-01	-2.52E-02	-2.18E-01	-5.00E-03	-2.18E-01	1.92E-02	-2.00E-01	3.92E-02	-1.71E-01	5.46E-02	-1.40E-01	6.58E-02	-1.10E-01	6.45E-02	-8.99E-02	4.83E-02	-7.63E-02	3.48E-02	-5.54E-02	2.71E-02
-1.95E-01	-3.77E-02	-2.15E-01	-1.16E-02	-2.18E-01	1.93E-02	-2.00E-01	4.59E-02	-1.73E-01	6.81E-02	-1.43E-01	8.08E-02	-1.16E-01	7.93E-02	-1.02E-01	5.36E-02	-8.96E-02	3.12E-02	-6.45E-02	2.54E-02
-1.95E-01	-3.28E-02	-2.07E-01	-8.48E-03	-2.06E-01	2.09E-02	-1.89E-01	4.63E-02	-1.62E-01	6.32E-02	-1.35E-01	7.27E-02	-1.13E-01	7.34E-02	-1.05E-01	6.03E-02	-1.06E-01	3.97E-02	-9.59E-02	2.64E-02
-1.95E-01	-3.06E-02	-2.07E-01	-8.67E-03	-2.09E-01	1.82E-02	-1.95E-01	4.22E-02	-1.71E-01	6.06E-02	-1.45E-01	7.37E-02	-1.21E-01	7.76E-02	-1.07E-01	5.92E-02	-9.13E-02	4.40E-02	-7.13E-02	3.31E-02
-2.02E-01	-2.24E-02	-2.14E-01	-1.65E-04	-2.13E-01	2.64E-02	-1.93E-01	4.93E-02	-1.67E-01	6.65E-02	-1.38E-01	7.41E-02	-1.11E-01	6.93E-02	-9.52E-02	5.44E-02	-8.84E-02	3.70E-02	-7.50E-02	2.77E-02
-1.90E-01	-2.41E-02	-1.99E-01	-5.77E-03	-1.97E-01	1.76E-02	-1.85E-01	4.00E-02	-1.67E-01	5.88E-02	-1.43E-01	6.59E-02	-1.19E-01	6.61E-02	-1.03E-01	5.23E-02	-8.90E-02	4.25E-02	-6.95E-02	3.73E-02
-1.96E-01	-2.23E-02	-2.03E-01	-3.49E-03	-2.01E-01	1.99E-02	-1.87E-01	4.04E-02	-1.64E-01	5.42E-02	-1.40E-01	6.38E-02	-1.19E-01	6.85E-02	-1.06E-01	5.49E-02	-9.62E-02	4.00E-02	-7.84E-02	3.22E-02
-1.93E-01	-2.33E-02	-2.04E-01	-5.81E-03	-2.02E-01	1.60E-02	-1.89E-01	3.68E-02	-1.67E-01	5.16E-02	-1.45E-01	6.06E-02	-1.22E-01	5.00E-02	-9.94E-02	4.78E-02	-8.47E-02	4.64E-02	-6.56E-02	4.87E-02
-1.98E-01	-2.08E-02	-2.04E-01	-4.69E-03	-2.01E-01	1.63E-02	-1.87E-01	3.57E-02	-1.66E-01	5.08E-02	-1.43E-01	5.90E-02	-1.22E-01	6.14E-02	-1.07E-01	4.78E-02	-9.53E-02	3.45E-02	-7.73E-02	2.57E-02
-2.06E-01	-3.93E-02	-2.18E-01	-1.56E-02	-2.16E-01	1.44E-02	-1.95E-01	4.14E-02	-1.64E-01	6.20E-02	-1.31E-01	7.40E-02	-1.01E-01	7.67E-02	-8.51E-02	6.87E-02	-7.90E-02	5.76E-02	-6.68E-02	5.07E-02
-1.92E-01	-2.69E-02	-2.02E-01	-6.61E-03	-1.99E-01	1.92E-02	-1.88E-01	4.37E-02	-1.67E-01	5.87E-02	-1.44E-01	6.83E-02	-1.23E-01	7.15E-02	-1.10E-01	5.61E-02	-1.01E-01	3.85E-02	-8.27E-02	3.11E-02
-2.00E-01	-2.97E-02	-2.06E-01	-1.07E-02	-2.02E-01	1.46E-02	-1.86E-01	3.91E-02	-1.62E-01	5.78E-02	-1.37E-01	6.98E-02	-1.10E-01	6.79E-02	-9.22E-02	5.71E-02	-8.50E-02	4.19E-02	-6.70E-02	3.74E-02
-1.91E-01	-2.76E-02	-2.06E-01	-5.08E-03	-2.07E-01	2.22E-02	-1.98E-01	4.63E-02	-1.75E-01	5.85E-02	-1.48E-01	6.77E-02	-1.23E-01	7.16E-02	-1.06E-01	5.74E-02	-9.35E-02	4.52E-02	-7.54E-02	3.88E-02
-1.98E-01	-2.27E-02	-2.10E-01	-3.80E-03	-2.07E-01	1.84E-02	-1.88E-01	3.76E-02	-1.61E-01	5.31E-02	-1.34E-01	6.40E-02	-1.11E-01	6.71E-02	-1.01E-01	5.29E-02	-9.93E-02	3.50E-02	-8.64E-02	2.69E-02
-1.99E-01	-3.68E-02	-2.10E-01	-1.39E-02	-2.12E-01	1.49E-02	-1.98E-01	4.08E-02	-1.74E-01	5.85E-02	-1.44E-01	6.74E-02	-1.15E-01	6.51E-02	-9.42E-02	5.20E-02	-7.89E-02	4.67E-02	-5.80E-02	4.67E-02
-2.04E-01	-2.36E-02	-2.19E-01	2.28E-03	-2.22E-01	3.11E-02	-2.04E-01	5.16E-02	-1.73E-01	6.49E-02	-1.41E-01	7.31E-02	-1.14E-01	7.07E-02	-9.96E-02	4.56E-02	-8.56E-02	2.48E-02	-6.43E-02	1.44E-02
-1.97E-01	-3.57E-02	-2.06E-01	-1.16E-02	-2.04E-01	1.93E-02	-1.87E-01	4.63E-02	-1.60E-01	6.43E-02	-1.32E-01	7.46E-02	-1.09E-01	7.51E-02	-9.75E-02	5.41E-02	-9.25E-02	3.29E-02	-7.44E-02	2.71E-02
-2.04E-01	-4.31E-02	-2.14E-01	-1.82E-02	-2.11E-01	1.36E-02	-1.90E-01	4.20E-02	-1.60E-01	6.15E-02	-1.29E-01	6.99E-02	-1.02E-01	6.64E-02	-9.09E-02	5.36E-02	-9.23E-02	3.26E-02	-7.86E-02	2.16E-02
-1.98E-01	-3.68E-02	-2.20E-01	-1.04E-02	-2.27E-01	2.03E-02	-2.08E-01	4.74E-02	-1.79E-01	6.85E-02	-1.46E-01	7.57E-02	-1.17E-01	6.98E-02	-9.88E-02	5.15E-02	-9.06E-02	3.31E-02	-8.16E-02	1.61E-02
-1.99E-01	-4.07E-02	-2.18E-01	-1.42E-02	-2.19E-01	1.91E-02	-2.00E-01	4.88E-02	-1.70E-01	7.03E-02	-1.40E-01	8.16E-02	-1.14E-01	7.92E-02	-9.94E-02	5.83E-02	-9.26E-02	3.70E-02	-7.89E-02	2.32E-02
-2.07E-01	-3.95E-02	-2.20E-01	-1.72E-02	-2.18E-01	1.45E-02	-2.05E-01	4.60E-02	-1.78E-01	6.63E-02	-1.47E-01	7.48E-02	-1.17E-01	7.16E-02	-9.71E-02	5.51E-02	-8.75E-02	3.66E-02	-6.79E-02	2.83E-02
-2.01E-01	-2.41E-02	-2.13E-01	-3.73E-03	-2.10E-01	2.18E-02	-1.95E-01	4.60E-02	-1.71E-01	6.25E-02	-1.45E-01	7.00E-02	-1.20E-01	6.56E-02	-1.03E-01	4.58E-02	-8.87E-02	2.92E-02	-6.74E-02	2.31E-02
-2.05E-01	-3.53E-02	-2.23E-01	-8.68E-03	-2.24E-01	2.39E-02	-2.05E-01	5.22E-02	-1.72E-01	6.85E-02	-1.39E-01	8.07E-02	-1.11E-01	7.90E-02	-9.61E-02	5.88E-02	-9.26E-02	3.46E-02	-7.83E-02	1.98E-02
-1.97E-01	-2.40E-02	-2.08E-01	-2.51E-03	-2.07E-01	2.34E-02	-1.91E-01	4.56E-02	-1.66E-01	5.84E-02	-1.38E-01	6.30E-02	-1.13E-01	5.93E-02	-9.64E-02	4.22E-02	-8.79E-02	2.38E-02	-7.09E-02	1.59E-02
-1.95E-01	-2.20E-02	-2.12E-01	1.96E-03	-2.10E-01	2.82E-02	-1.93E-01	5.09E-02	-1.65E-01	6.38E-02	-1.36E-01	7.19E-02	-1.14E-01	7.37E-02	-1.03E-01	5.05E-02	-8.95E-02	3.01E-02	-7.02E-02	2.06E-02
-1.92E-01	-2.43E-02	-2.05E-01	-2.86E-03	-2.04E-01	2.22E-02	-1.90E-01	4.48E-02	-1.68E-01	6.02E-02	-1.41E-01	6.81E-02	-1.18E-01	7.03E-02	-1.02E-01	5.45E-02	-9.24E-02	3.67E-02	-7.48E-02	2.85E-02
-1.98E-01	-3.18E-02	-2.12E-01	-8.39E-03	-2.13E-01	2.07E-02	-2.00E-01	4.65E-02	-1.76E-01	6.26E-02	-1.50E-01	7.18E-02	-1.26E-01	7.10E-02	-1.13E-01	4.85E-02	-9.84E-02	3.07E-02	-7.52E-02	2.23E-02

-1.91E-01	-3.83E-02	-2.08E-01	-9.10E-03	-2.15E-01	2.44E-02	-1.99E-01	5.25E-02	-1.73E-01	7.24E-02	-1.43E-01	8.14E-02	-1.17E-01	7.92E-02	-1.02E-01	6.01E-02	-9.79E-02	3.71E-02	-8.29E-02	2.43E-02
-1.99E-01	-2.56E-02	-2.12E-01	-8.60E-04	-2.15E-01	2.78E-02	-2.03E-01	5.14E-02	-1.79E-01	6.47E-02	-1.51E-01	7.09E-02	-1.24E-01	6.89E-02	-1.05E-01	4.95E-02	-8.63E-02	3.62E-02	-6.31E-02	3.02E-02
-1.97E-01	-3.31E-02	-2.10E-01	-8.97E-03	-2.10E-01	1.93E-02	-1.93E-01	4.27E-02	-1.66E-01	5.94E-02	-1.34E-01	6.66E-02	-1.04E-01	6.52E-02	-8.67E-02	5.05E-02	-7.88E-02	3.48E-02	-6.06E-02	2.80E-02
-2.06E-01	-2.62E-02	-2.22E-01	-5.10E-03	-2.18E-01	1.79E-02	-1.98E-01	4.08E-02	-1.71E-01	6.39E-02	-1.41E-01	7.65E-02	-1.13E-01	7.37E-02	-9.66E-02	5.15E-02	-8.75E-02	2.91E-02	-6.54E-02	1.87E-02
-2.03E-01	-3.28E-02	-2.19E-01	-7.84E-03	-2.17E-01	2.33E-02	-2.00E-01	5.10E-02	-1.72E-01	6.90E-02	-1.42E-01	7.41E-02	-1.13E-01	6.81E-02	-9.48E-02	5.00E-02	-8.51E-02	3.10E-02	-6.61E-02	2.32E-02
-1.99E-01	-2.42E-02	-2.13E-01	-1.14E-03	-2.15E-01	2.42E-02	-1.97E-01	4.41E-02	-1.71E-01	6.17E-02	-1.41E-01	7.15E-02	-1.14E-01	7.23E-02	-9.68E-02	5.85E-02	-8.84E-02	4.21E-02	-7.29E-02	3.30E-02
-1.89E-01	-2.95E-02	-2.02E-01	-1.04E-02	-2.04E-01	1.32E-02	-1.92E-01	3.48E-02	-1.71E-01	5.34E-02	-1.44E-01	6.47E-02	-1.17E-01	6.70E-02	-9.72E-02	5.61E-02	-8.28E-02	4.85E-02	-6.16E-02	4.93E-02
-1.91E-01	-2.66E-02	-2.03E-01	-9.06E-03	-1.97E-01	1.16E-02	-1.79E-01	3.40E-02	-1.55E-01	5.38E-02	-1.30E-01	6.77E-02	-1.09E-01	7.28E-02	-9.88E-02	6.40E-02	-9.73E-02	5.26E-02	-9.27E-02	4.41E-02
-1.99E-01	-2.76E-02	-2.08E-01	-6.14E-03	-2.07E-01	2.04E-02	-1.94E-01	4.38E-02	-1.71E-01	5.76E-02	-1.44E-01	6.50E-02	-1.18E-01	6.26E-02	-9.95E-02	4.88E-02	-8.77E-02	3.45E-02	-6.79E-02	3.02E-02
-1.90E-01	-2.56E-02	-2.04E-01	-8.30E-03	-2.04E-01	1.36E-02	-1.92E-01	3.49E-02	-1.71E-01	5.09E-02	-1.50E-01	6.53E-02	-1.26E-01	6.74E-02	-1.07E-01	5.61E-02	-9.13E-02	4.82E-02	-7.01E-02	4.55E-02
-1.88E-01	-2.93E-02	-1.96E-01	-1.17E-02	-1.93E-01	1.21E-02	-1.84E-01	3.68E-02	-1.65E-01	5.36E-02	-1.44E-01	6.80E-02	-1.26E-01	7.59E-02	-1.12E-01	6.32E-02	-9.71E-02	5.01E-02	-7.77E-02	4.21E-02
-1.87E-01	-2.67E-02	-2.00E-01	-8.06E-03	-2.00E-01	1.57E-02	-1.89E-01	3.94E-02	-1.69E-01	5.74E-02	-1.44E-01	6.94E-02	-1.22E-01	7.69E-02	-1.07E-01	6.26E-02	-9.38E-02	4.88E-02	-7.33E-02	4.42E-02
-1.99E-01	-1.83E-02	-2.11E-01	-2.78E-03	-2.10E-01	1.79E-02	-1.96E-01	3.85E-02	-1.75E-01	5.54E-02	-1.50E-01	6.45E-02	-1.24E-01	6.36E-02	-1.05E-01	4.85E-02	-8.82E-02	3.98E-02	-6.51E-02	3.39E-02
-1.91E-01	-2.61E-02	-2.05E-01	-4.71E-03	-2.09E-01	1.97E-02	-1.98E-01	4.02E-02	-1.75E-01	5.40E-02	-1.49E-01	6.33E-02	-1.23E-01	6.40E-02	-1.04E-01	5.33E-02	-9.14E-02	4.31E-02	-7.29E-02	3.77E-02
-1.84E-01	-2.21E-02	-1.92E-01	-4.07E-03	-1.88E-01	1.75E-02	-1.78E-01	3.84E-02	-1.57E-01	5.07E-02	-1.35E-01	6.16E-02	-1.13E-01	6.58E-02	-1.01E-01	6.29E-02	-9.74E-02	5.47E-02	-8.60E-02	4.82E-02
-1.98E-01	-2.84E-02	-2.09E-01	-7.67E-03	-2.09E-01	1.78E-02	-1.93E-01	4.03E-02	-1.70E-01	5.78E-02	-1.46E-01	7.05E-02	-1.22E-01	6.99E-02	-1.05E-01	5.50E-02	-9.42E-02	4.03E-02	-7.66E-02	3.20E-02
-2.02E-01	-2.76E-02	-2.13E-01	-8.45E-03	-2.12E-01	1.60E-02	-1.96E-01	3.86E-02	-1.72E-01	5.69E-02	-1.45E-01	6.77E-02	-1.21E-01	6.82E-02	-1.05E-01	4.94E-02	-9.16E-02	3.35E-02	-7.01E-02	2.52E-02
-1.91E-01	-2.07E-02	-1.96E-01	2.54E-04	-1.95E-01	2.42E-02	-1.83E-01	4.38E-02	-1.62E-01	5.47E-02	-1.39E-01	6.37E-02	-1.17E-01	6.49E-02	-1.02E-01	5.55E-02	-9.31E-02	4.28E-02	-7.77E-02	3.61E-02
-1.96E-01	-2.47E-02	-2.04E-01	-2.85E-03	-2.02E-01	2.23E-02	-1.84E-01	4.24E-02	-1.59E-01	5.68E-02	-1.33E-01	6.50E-02	-1.12E-01	6.64E-02	-1.04E-01	5.56E-02	-1.05E-01	3.74E-02	-9.35E-02	2.76E-02
-1.86E-01	-1.98E-02	-1.92E-01	-1.44E-03	-1.94E-01	2.02E-02	-1.82E-01	3.76E-02	-1.68E-01	5.32E-02	-1.50E-01	6.22E-02	-1.29E-01	6.02E-02	-1.12E-01	5.04E-02	-9.82E-02	4.45E-02	-7.92E-02	4.09E-02
-1.94E-01	-2.35E-02	-2.04E-01	-5.27E-03	-2.04E-01	1.81E-02	-1.89E-01	3.90E-02	-1.69E-01	5.62E-02	-1.44E-01	6.46E-02	-1.23E-01	6.78E-02	-1.09E-01	5.20E-02	-9.50E-02	4.06E-02	-7.60E-02	3.56E-02
-1.89E-01	-2.62E-02	-1.95E-01	-6.89E-03	-1.90E-01	1.71E-02	-1.78E-01	4.06E-02	-1.65E-01	5.84E-02	-1.45E-01	6.38E-02	-1.24E-01	6.18E-02	-1.09E-01	5.20E-02	-1.02E-01	4.06E-02	-8.61E-02	3.45E-02
-1.98E-01	-3.01E-02	-2.10E-01	-8.80E-03	-2.10E-01	1.84E-02	-1.97E-01	4.41E-02	-1.75E-01	6.40E-02	-1.50E-01	7.47E-02	-1.24E-01	7.36E-02	-1.05E-01	5.89E-02	-9.09E-02	4.71E-02	-7.46E-02	3.67E-02
-2.03E-01	-3.12E-02	-2.14E-01	-8.96E-03	-2.12E-01	1.82E-02	-1.91E-01	3.96E-02	-1.61E-01	5.81E-02	-1.30E-01	7.10E-02	-1.07E-01	7.97E-02	-9.63E-02	5.96E-02	-8.49E-02	3.88E-02	-6.52E-02	2.96E-02
-1.99E-01	-3.24E-02	-2.09E-01	-9.51E-03	-2.06E-01	1.84E-02	-1.90E-01	4.42E-02	-1.66E-01	6.37E-02	-1.38E-01	7.18E-02	-1.13E-01	7.61E-02	-9.76E-02	5.75E-02	-8.42E-02	3.93E-02	-6.34E-02	3.20E-02
-1.86E-01	-2.74E-02	-1.98E-01	-7.82E-03	-1.99E-01	1.61E-02	-1.87E-01	3.85E-02	-1.69E-01	5.64E-02	-1.46E-01	6.53E-02	-1.23E-01	6.65E-02	-1.05E-01	5.30E-02	-9.14E-02	4.12E-02	-7.16E-02	3.66E-02
-1.91E-01	-2.16E-02	-2.02E-01	-1.20E-03	-2.02E-01	2.19E-02	-1.87E-01	3.99E-02	-1.64E-01	5.47E-02	-1.39E-01	6.38E-02	-1.16E-01	6.64E-02	-1.03E-01	5.21E-02	-9.69E-02	3.47E-02	-8.13E-02	2.91E-02
-1.96E-01	-3.68E-02	-2.06E-01	-1.17E-02	-2.07E-01	1.89E-02	-1.94E-01	4.53E-02	-1.69E-01	6.23E-02	-1.41E-01	7.05E-02	-1.14E-01	7.13E-02	-9.85E-02	5.33E-02	-8.71E-02	3.77E-02	-7.26E-02	2.96E-02
-1.98E-01	-2.67E-02	-2.09E-01	-5.56E-03	-2.08E-01	2.03E-02	-1.94E-01	4.37E-02	-1.69E-01	5.85E-02	-1.43E-01	6.96E-02	-1.17E-01	6.20E-02	-9.72E-02	4.46E-02	-7.80E-02	3.88E-02	-5.58E-02	3.38E-02
-2.01E-01	-3.57E-02	-2.11E-01	-1.29E-02	-2.05E-01	1.47E-02	-1.83E-01	4.01E-02	-1.52E-01	5.91E-02	-1.21E-01	7.02E-02	-9.68E-02	7.55E-02	-8.86E-02	6.77E-02	-8.98E-02	5.06E-02	-8.08E-02	4.00E-02
-1.97E-01	-3.24E-02	-2.11E-01	-1.12E-02	-2.12E-01	1.60E-02	-1.97E-01	4.09E-02	-1.71E-01	5.94E-02	-1.43E-01	7.19E-02	-1.16E-01	7.44E-02	-9.94E-02	5.62E-02	-8.59E-02	4.03E-02	-6.75E-02	3.24E-02
-1.94E-01	-2.42E-02	-2.06E-01	-3.33E-03	-2.05E-01	2.22E-02	-1.93E-01	4.60E-02	-1.72E-01	6.17E-02	-1.47E-01	6.94E-02	-1.22E-01	6.66E-02	-1.04E-01	5.07E-02	-8.91E-02	3.78E-02	-7.31E-02	2.88E-02
-2.01E-01	-3.60E-02	-2.13E-01	-1.43E-02	-2.11E-01	1.39E-02	-1.93E-01	4.06E-02	-1.66E-01	6.10E-02	-1.35E-01	7.19E-02	-1.06E-01	7.06E-02	-8.87E-02	5.57E-02	-8.15E-02	3.99E-02	-6.36E-02	3.56E-02
-2.05E-01	-4.11E-02	-2.20E-01	-1.55E-02	-2.18E-01	1.77E-02	-2.00E-01	4.82E-02	-1.71E-01	6.86E-02	-1.37E-01	7.49E-02	-1.06E-01	7.22E-02	-8.95E-02	5.37E-02	-8.26E-02	3.62E-02	-6.84E-02	2.71E-02
-1.98E-01	-2.46E-02	-2.08E-01	-1.88E-03	-2.03E-01	2.53E-02	-1.87E-01	5.08E-02	-1.65E-01	6.85E-02	-1.40E-01	7.30E-02	-1.15E-01	6.70E-02	-9.84E-02	4.83E-02	-8.74E-02	3.10E-02	-7.08E-02	2.15E-02
-1.93E-01	-2.74E-02	-2.02E-01	-8.96E-03	-2.02E-01	1.51E-02	-1.89E-01	3.69E-02	-1.68E-01	5.41E-02	-1.48E-01	6.73E-02	-1.27E-01	6.94E-02	-1.13E-01	5.33E-02	-9.86E-02	3.99E-02	-7.90E-02	3.15E-02
-1.88E-01	-3.15E-02	-1.99E-01	-8.36E-03	-2.01E-01	2.04E-02	-1.90E-01	4.49E-02	-1.69E-01	6.14E-02	-1.49E-01	7.49E-02	-1.28E-01	7.16E-02	-1.13E-01	5.16E-02	-9.56E-02	3.96E-02	-7.15E-02	3.67E-02
-1.93E-01	-2.58E-02	-2.04E-01	-4.42E-03	-2.03E-01	2.14E-02	-1.88E-01	4.46E-02	-1.65E-01	6.12E-02	-1.39E-01	7.07E-02	-1.15E-01	7.10E-02	-1.00E-01	5.53E-02	-9.23E-02	3.80E-02	-7.31E-02	3.21E-02
-1.89E-01	-2.53E-02	-2.07E-01	-2.28E-03	-2.12E-01	2.32E-02	-1.97E-01	4.37E-02	-1.72E-01	6.12E-02	-1.46E-01	7.49E-02	-1.20E-01	7.59E-02	-1.01E-01	5.85E-02	-8.36E-02	4.52E-02	-6.37E-02	3.74E-02
-1.89E-01	-1.91E-02	-2.00E-01	2.60E-03	-2.01E-01	2.74E-02	-1.90E-01	4.81E-02	-1.72E-01	6.16E-02	-1.51E-01	6.92E-02	-1.29E-01	6.78E-02	-1.12E-01	5.17E-02	-9.57E-02	3.94E-02	-7.45E-02	3.31E-02
-1.98E-01	-2.84E-02	-2.08E-01	-9.85E-03	-2.06E-01	1.50E-02	-1.92E-01	3.91E-02	-1.71E-01	5.90E-02	-1.45E-01	6.72E-02	-1.19E-01	6.88E-02	-1.01E-01	5.14E-02	-8.46E-02	3.95E-02	-6.17E-02	3.51E-02
-1.91E-01	-2.89E-02	-1.97E-01	-1.02E-02	-1.98E-01	1.31E-02	-1.84E-01	3.31E-02	-1.65E-01	5.09E-02	-1.41E-01	6.05E-02	-1.18E-01	6.42E-02	-1.03E-01	5.66E-02	-9.40E-02	4.61E-02	-7.76E-02	4.29E-02
-1.92E-01	-2.14E-02	-2.04E-01	-2.94E-03	-2.03E-01	1.98E-02	-1.92E-01	4.13E-02	-1.72E-01	5.57E-02	-1.47E-01	6.38E-02	-1.23E-01	6.47E-02	-1.06E-01	5.42E-02	-9.49E-02	4.42E-02	-7.75E-02	3.82E-02
-1.95E-01	-2.93E-02	-2.08E-01	-8.69E-03	-2.07E-01	1.73E-02	-1.92E-01	4.15E-02	-1.69E-01	6.07E-02	-1.44E-01	7.12E-02	-1.23E-01	7.65E-02	-1.11E-01	5.65E-02	-9.64E-02	3.84E-02	-7.40E-02	3.09E-02
-2.00E-01	-2.99E-02	-2.11E-01	-8.91E-03	-2.07E-01	1.66E-02	-1.90E-01	3.99E-02	-1.65E-01	5.66E-02	-1.36E-01	6.58E-02	-1.10E-01	6.75E-02	-9.65E-02	6.05E-02	-9.25E-02	4.58E-02	-7.65E-02	4.28E-02
-1.94E-01	-3.05E-02	-2.08E-01	-5.73E-03	-2.09E-01	2.43E-02	-1.95E-01	5.00E-02	-1.69E-01	6.50E-02	-1.39E-01	7.29E-02	-1.12E-01	7.34E-02	-9.90E-02	5.48E-02	-9.33E-02	3.52E-02	-7.41E-02	3.03E-02
-1.94E-01	-2.70E-02	-2.06E-01	-6.36E-03	-2.02E-01	1.91E-02	-1.90E-01	4.54E-02	-1.67E-01	5.94E-02	-1.40E-01	6.89E-02	-1.14E-01	6.65E-02	-9.71E-02	4.76E-02	-8.23E-02	3.32E-02	-6.29E-02	2.65E-02
-1.87E-01	-1.96E-02	-1.98E-01	-2.59E-03	-1.96E-01	1.68E-02	-1.83E-01	3.61E-02	-1.65E-01	5.32E-02	-1.46E-01	6.66E-02	-1.25E-01	6.52E-02	-1.07E-01	5.10E-02	-9.41E-02	3.60E-02	-7.40E-02	2.84E-02
-1.96E-01	-3.97E-02	-2.13E-01	-1.46E-02	-2.12E-01	1.67E-02	-1.93E-01	4.48E-02	-1.62E-01	6.47E-02	-1.32E-01	7.73E-02	-1.07E-01	8.13E-02	-9.83E-02	5.97E-02	-9.70E-02	3.56E-02	-7.89E-02	2.46E-02
-1.95E-01	-3.87E-02	-2.05E-01	-1.41E-02	-2.03E-01	1.68E-02	-1.86E-01	4.40E-02	-1.59E-01	6.39E-02	-1.30E-01	7.51E-02	-1.04E-01	7.37E-02	-9.26E-02	6.30E-02	-9.34E-02	4.40E-02	-8.37E-02	3.57E-02
-1.97E-01	-2.15E-02	-2.09E-01	-2.14E-03	-2.07E-01	2.12E-02	-1.88E-01	4.07E-02	-1.64E-01	5.85E-02	-1.38E-01	6.69E-02	-1.17E-01	6.90E-02	-1.08E-01	4.72E-02	-9.25E-02	3.13E-02	-7.18E-02	2.37E-02
-1.89E-01	-3.02E-02	-2.02E-01	-8.65E-03	-2.04E-01	1.72E-02	-1.94E-01	4.02E-02	-1.71E-01	5.43E-02	-1.47E-01	6.59E-02	-1.22E-01	6.66E-02	-1.06E-01	5.12E-02	-9.10E-02	4.15E-02	-7.01E-02	3.79E-02
-1.98E-01	-2.34E-02	-2.09E-01	-1.09E-03	-2.09E-01	2.58E-02	-1.94E-01	4.81E-02	-1.67E-01	6.00E-02	-1.37E-01	6.58E-02	-1.12E-01	6.52E-02	-9.72E-02	4.49E-02	-8.40E-02	2.97E-02	-6.71E-02	2.33E-02
-1.88E-01	-3.67E-02	-2.01E-01	-1.42E-02	-2.01E-01	1.42E-02	-1.88E-01	4.18E-02	-1.70E-01	6.49E-02	-1.46E-01	7.66E-02	-1.23E-01	7.79E-02	-1.08E-01	6.07E-02	-9.73E-02	4.39E-02	-7.66E-02	3.91E-02
-1.98E-01	-2.78E-02	-2.13E-01	-3.68E-03	-2.17E-01	2.51E-02	-2.02E-01	4.88E-02	-1.76E-01	6.53E-02	-1.49E-01	7.61E-02	-1.21E-01	6.86E-02	-1.00E-01	5.13E-02	-8.60E-02	3.61E-02	-6.72E-02	2.89E-02
-1.98E-01	-2.82E-02	-2.12E-01	-5.37E-03	-2.14E-01	2.15E-02	-2.01E-01	4.40E-02	-1.77E-01	5.91E-02	-1.51E-01	6.91E-02	-1.25E-01	6.53E-02	-1.04E-01	4.91E-02	-8.70E-02	3.91E-02	-6.49E-02	3.39E-02
-1.95E-01	-3.11E-02	-2.12E-01	-9.75E-03	-2.13E-01	1.84E-02	-1.98E-01	4.43E-02	-1.70E-01	6.20E-02	-1.41E-01	7.58E-02	-1.17E-01	8.00E-02	-1.03E-01	5.98E-02	-9.13E-02	4.07E-02	-7.22E-02	3.03E-02
-2.00E-01	-3.76E-02	-2.10E-01	-1.34E-02	-2.08E-01	1.74E-02	-1.90E-01	4.48E-02	-1.62E-01	6.34E-02	-1.35E-01	7.92E-02	-1.12E-01	8.21E-02	-1.02E-01	5.88E-02	-8.90E-02	3.81E-02	-6.68E-02	2.97E-02
-1.95E-01	-3.07E-02	-2.10E-01	-8.02E-03	-2.12E-01	1.97E-02	-1.97E-01	4.44E-02	-1.72E-01	6.14E-02	-1.42E-01	6.85E-02	-1.13E-01	6.20E-02	-9.52E-02	4.89E-02	-8.63E-02	3.90E-02	-7.20E-02	3.37E-02
-2.01E-01	-3.59E-02	-2.20E-01	-1.11E-02	-2.19E-01	1.91E-02	-1.98E-01	4.69E-02	-1.67E-01	6.79E-02	-1.35E-01	7.98E-02	-1.06E-01	7.95E-02	-9.30E-02	6.16E-02	-9.30E-02	3.82E-02	-7.50E-02	2.95E-02
-1.93E-01	-3.83E-02	-2.05E-01	-1.21E-02	-2.08E-01	2.05E-02	-1.97E-01	4.89E-02	-1.72E-01	6.59E-02	-1.45E-01	7.77E-02	-1.17E-01	7.81E-02	-9.85E-02	6.11E-02	-8.60E-02	4.43E-02	-6.78E-02	3.66E-02
-1.98E-01	-3.36E-02	-2.14E-01	-1.02E-02	-2.17E-01	1.85E-02	-2.00E-01	4.27E-02	-1.73E-01	6.28E-02	-1.44E-01	7.41E-02	-1.17E-01	7.30E-02	-1.00E-01	5.08E-02	-8.46E-02	3.49E-02	-6.32E-02	2.59E-02
-1.91E-01	-2.64E-02	-2.02E-01	-3.54E-03	-2.03E-01	2.39E-02	-1.92E-01	4.69E-02	-1.69E-01	6.04E-02	-1.45E-01	7.08E-02	-1.22E-01	7.19E-02	-1.07E-01	5.71E-02	-9.79E-02	4.17E-02	-8.09E-02	3.53E-02
-2.03E-01	-2.94E-02	-2.20E-01	-4.63E-03	-2.22E-01	2.59E-02	-2.05E-01	5.29E-02	-1.75E-01	6.91E-02	-1.41E-01	7.58E-02	-1.10E-01	7.14E-02	-9.44E-02	4.74E-02	-8.26E-02	2.83E-02	-5.88E-02	2.33E-02
-1.97E-01	-2.98E-02	-2.05E-01	-1.17E-02	-2.03E-01	1.23E-02	-1.87E-01	3.44E-02	-1.65E-01	5.32E-02	-1.43E-01	6.70E-02	-1.21E-01	6.70E-02	-1.06E-01	4.90E-02	-9.02E-02	3.68E-02	-6.74E-02	3.36E-02
-2.03E-01	-3.21E-02	-2.17E-01	-7.54E-03	-2.16E-01	2.17E-02	-1.96E-01	4.69E-02	-1.65E-01	6.44E-02	-1.31E-01	7.42E-02	-1.03E-01	7.56E-02	-8.89E-02	6.16E-02	-8.60E-02	4.10E-02	-7.21E-02	3.29E-02
-1.94E-01	-2.81E-02	-2.06E-01	-8.13E-03	-2.05E-01	1.66E-02	-1.88E-01	3.91E-02	-1.65E-01	5.76E-02	-1.41E-01	7.04E-02	-1.20E-01	7.30E-02	-1.07E-01	5.42E-02	-9.54E-02	3.81E-02	-7.80E-02	2.98E-02
-1.99E-01	-3.06E-02	-2.11E-01	-1.04E-02	-2.10E-01	1.54E-02	-1.94E-01	3.97E-02	-1.68E-01	5.82E-02	-1.40E-01	7.09E-02	-1.13E-01	7.20E-02	-9.77E-02	5.59E-02	-8.91E-02	3.91E-02	-7.17E-02	3.24E-02
-1.97E-01	-2.54E-02	-2.08E-01	-4.73E-03	-2.08E-01	2.12E-02	-1.94E-01	4.41E-02	-1.72E-01	6.10E-02	-1.45E-01	7.02E-02	-1.20E-01	7.03E-02	-1.03E-01	5.29E-02	-8.77E-02	3.92E-02	-6.61E-02	3.22E-02
-1.97E-01	-2.40E-02	-2.11E-01	-3.72E-03	-2.11E-01	2.23E-02	-1.95E-01	4.60E-02	-1.69E-01	6.23E-02	-1.40E-01	7.11E-02	-1.13E-01	6.75E-02	-9.63E-02	5.03E-02	-8.59E-02	3.54E-02	-6.89E-02	2.91E-02
-2.02E-01	-2.71E-02	-2.12E-01	-4.27E-03	-2.10E-01	2.39E-02	-1.93E-01	4.79E-02	-1.65E-01	6.20E-02	-1.36E-01	7.13E-02	-1.10E-01	7.20E-02	-9.48E-02	5.28E-02	-8.33E-02	3.63E-02	-6.79E-02	2.75E-02
-1.94E-01	-2.62E-02	-2.06E-01	-4.76E-03	-2.04E-01	2.15E-02	-1.89E-01	4.60E-02	-1.66E-01	6.28E-02	-1.41E-01	7.21E-02	-1.17E-01	7.00E-02	-1.01E-01	5.25E-02	-9.01E-02	3.72E-02	-7.26E-02	2.85E-02
-1.98E-01	-2.67E-02	-2.09E-01	-7.38E-03	-2.06E-01	1.69E-02	-1.91E-01	4.16E-02	-1.67E-01	5.83E-02	-1.41E-01	7.10E-02	-1.18E-01	7.47E-02	-1.05E-01	5.63E-02	-9.28E-02	3.98E-02	-7.14E-02	3.23E-02
-1.96E-01	-3.07E-02	-2.07E-01	-9.06E-03	-2.07E-01	1.78E-02	-1.92E-01	4.15E-02	-1.67E-01	5.82E-02	-1.41E-01	6.85E-02	-1.17E-01	7.01E-02	-1.02E-01	5.39E-02	-9.17E-02	3.93E-02	-7.63E-02	3.14E-02
-1.91E-01	-2.73E-02	-2.01E-01	-6.40E-03	-1.98E-01	1.93E-02	-1.84E-01	4.32E-02	-1.66E-01	6.10E-02	-1.43E-01	6.56E-02	-1.20E-01	5.95E-02	-1.05E-01	4.55E-02	-9.76E-02	3.26E-02	-8.38E-02	2.49E-02
-1.94E-01	-1.96E-02	-2.04E-01	-3.95E-04	-2.05E-01	2.29E-02	-1.91E-01	4.27E-02	-1.69E-01	5.84E-02	-1.47E-01	6.91E-02	-1.27E-01	6.38E-02	-1.12E-01	4.50E-02	-9.46E-02	3.43E-02	-7.37E-02	2.51E-02
-1.95E-01	-2.69E-02	-2.06E-01	-5.69E-03	-2.06E-01	2.08E-02	-1.91E-01	4.45E-02	-1.69E-01	6.18E-02	-1.43E-01	7.08E-02	-1.20E-01	7.33E-02	-1.07E-01	5.57E-02	-9.43E-02	4.01E-02	-7.91E-02	2.93E-02
-1.98E-01	-2.57E-02	-2.10E-01	-3.30E-03	-2.10E-01	2.40E-02	-1.95E-01	4.79E-02	-1.71E-01	6.39E-02	-1.43E-01	7.28E-02	-1.17E-01	7.13E-02	-1.02E-01	5.11E-02	-8.63E-02	3.78E-02	-6.35E-02	3.20E-02
-1.86E-01	-3.30E-02	-1.94E-01	-8.66E-03	-1.93E-01	2.27E-02	-1.83E-01	5.01E-02	-1.61E-01	6.55E-02	-1.42E-01	7.78E-02	-1.23E-01	7.37E-02	-1.11E-01	5.40E-02	-1.07E-01	3.16E-02	-9.46E-02	2.01E-02
-1.94E-01	-3.55E-02	-2.08E-01	-1.52E-02	-2.07E-01	1.12E-02	-1.90E-01	3.69E-02	-1.66E-01	5.87E-02	-1.40E-01	7.46E-02	-1.17E-01	7.87E-02	-1.03E-01	5.88E-02	-8.99E-02	4.13E-02	-6.89E-02	3.48E-02
-2.05E-01	-3.06E-02	-2.20E-01	-5.07E-03	-2.20E-01	2.45E-02	-1.99E-01	4.82E-02	-1.67E-01	6.42E-02	-1.32E-01	7.01E-02	-1.01E-01	6.80E-02	-8.49E-02	5.27E-02	-8.10E-02	3.22E-02	-6.72E-02	2.44E-02
-1.89E-01	-1.93E-02	-1.95E-01	-3.07E-03	-1.93E-01	1.70E-02	-1.82E-01	3.58E-02	-1.64E-01	5.00E-02	-1.44E-01	6.00E-02	-1.23E-01	5.95E-02	-1.09E-01	5.16E-02	-9.99E-02	4.42E-02	-8.37E-02	4.04E-02
-2.07E-01	-3.16E-02	-2.16E-01	-9.73E-03	-2.10E-01	1.69E-02	-1.88E-01	3.99E-02	-1.55E-01	5.40E-02	-1.22E-01	6.46E-02	-9.39E-02	6.84E-02	-7.90E-02	5.70E-02	-7.32E-02	4.45E-02	-5.93E-02	3.83E-02

-1.98E-01	-2.90E-02	-2.08E-01	-1.05E-02	-2.06E-01	1.38E-02	-1.91E-01	3.71E-02	-1.68E-01	5.56E-02	-1.42E-01	6.62E-02	-1.19E-01	6.82E-02	-1.05E-01	5.14E-02	-9.43E-02	3.50E-02	-7.96E-02	2.48E-02
-2.01E-01	-3.06E-02	-2.17E-01	-9.01E-03	-2.12E-01	1.68E-02	-1.92E-01	4.20E-02	-1.62E-01	6.16E-02	-1.30E-01	7.43E-02	-1.02E-01	7.72E-02	-8.39E-02	6.43E-02	-7.48E-02	5.16E-02	-6.16E-02	4.42E-02
-1.92E-01	-3.34E-02	-2.01E-01	-1.27E-02	-2.04E-01	1.33E-02	-1.89E-01	3.45E-02	-1.67E-01	5.42E-02	-1.41E-01	6.56E-02	-1.17E-01	7.01E-02	-1.01E-01	5.65E-02	-9.00E-02	4.32E-02	-7.22E-02	3.74E-02
-2.04E-01	-2.99E-02	-2.13E-01	-1.05E-02	-2.07E-01	1.52E-02	-1.88E-01	4.16E-02	-1.60E-01	6.09E-02	-1.31E-01	7.28E-02	-1.06E-01	7.37E-02	-9.33E-02	5.42E-02	-8.65E-02	3.45E-02	-7.64E-02	2.30E-02
-1.94E-01	-2.99E-02	-2.06E-01	-8.03E-03	-2.05E-01	1.89E-02	-1.88E-01	4.35E-02	-1.65E-01	6.38E-02	-1.40E-01	7.46E-02	-1.17E-01	7.66E-02	-1.02E-01	6.10E-02	-9.12E-02	4.71E-02	-7.92E-02	3.61E-02
-1.91E-01	-3.21E-02	-2.02E-01	-9.16E-03	-2.01E-01	1.99E-02	-1.88E-01	4.63E-02	-1.65E-01	6.47E-02	-1.39E-01	7.31E-02	-1.15E-01	7.27E-02	-1.01E-01	5.50E-02	-9.21E-02	3.67E-02	-7.97E-02	2.68E-02
-1.97E-01	-2.98E-02	-2.13E-01	-6.94E-03	-2.12E-01	2.15E-02	-1.96E-01	4.72E-02	-1.70E-01	6.41E-02	-1.43E-01	7.41E-02	-1.19E-01	7.21E-02	-1.02E-01	5.49E-02	-9.35E-02	3.62E-02	-7.78E-02	2.60E-02
-1.98E-01	-2.80E-02	-2.12E-01	-6.17E-03	-2.11E-01	2.02E-02	-1.93E-01	4.35E-02	-1.66E-01	6.18E-02	-1.38E-01	7.07E-02	-1.12E-01	7.13E-02	-9.62E-02	5.45E-02	-8.47E-02	4.23E-02	-6.81E-02	3.23E-02
-1.91E-01	-2.02E-02	-2.03E-01	-4.29E-04	-2.02E-01	2.34E-02	-1.89E-01	4.46E-02	-1.69E-01	5.93E-02	-1.48E-01	6.84E-02	-1.29E-01	6.67E-02	-1.16E-01	4.84E-02	-1.02E-01	3.33E-02	-8.19E-02	2.61E-02
-1.97E-01	-2.40E-02	-2.10E-01	-5.40E-03	-2.09E-01	1.81E-02	-1.92E-01	4.04E-02	-1.69E-01	5.99E-02	-1.42E-01	6.98E-02	-1.17E-01	6.96E-02	-1.02E-01	5.73E-02	-9.49E-02	4.16E-02	-7.82E-02	3.50E-02
-1.96E-01	-2.98E-02	-2.12E-01	-7.74E-03	-2.11E-01	1.97E-02	-1.97E-01	4.56E-02	-1.73E-01	6.36E-02	-1.47E-01	7.45E-02	-1.21E-01	7.30E-02	-1.03E-01	5.73E-02	-9.00E-02	4.24E-02	-7.77E-02	3.05E-02
-1.91E-01	-1.99E-02	-2.04E-01	-1.31E-03	-2.06E-01	2.08E-02	-1.92E-01	3.96E-02	-1.69E-01	5.39E-02	-1.44E-01	6.40E-02	-1.22E-01	6.80E-02	-1.06E-01	5.33E-02	-9.14E-02	4.03E-02	-7.32E-02	3.26E-02
-1.91E-01	-2.27E-02	-2.01E-01	-4.57E-03	-1.97E-01	1.75E-02	-1.82E-01	3.89E-02	-1.60E-01	5.45E-02	-1.36E-01	6.54E-02	-1.13E-01	6.36E-02	-9.78E-02	4.99E-02	-8.82E-02	3.78E-02	-7.73E-02	2.93E-02
-1.92E-01	-3.15E-02	-2.06E-01	-8.62E-03	-2.10E-01	1.99E-02	-1.97E-01	4.50E-02	-1.74E-01	6.25E-02	-1.49E-01	7.31E-02	-1.26E-01	7.43E-02	-1.10E-01	5.62E-02	-9.71E-02	4.04E-02	-7.88E-02	3.18E-02
-1.97E-01	-2.76E-02	-2.10E-01	-8.06E-03	-2.08E-01	1.69E-02	-1.95E-01	4.07E-02	-1.72E-01	5.67E-02	-1.45E-01	6.08E-02	-1.18E-01	5.33E-02	-9.68E-02	4.28E-02	-8.27E-02	3.91E-02	-6.29E-02	3.87E-02
-1.94E-01	-2.60E-02	-2.05E-01	-4.50E-03	-2.05E-01	2.11E-02	-1.91E-01	4.30E-02	-1.67E-01	5.78E-02	-1.41E-01	6.73E-02	-1.18E-01	6.89E-02	-1.06E-01	5.27E-02	-9.98E-02	3.47E-02	-8.31E-02	2.87E-02
-1.96E-01	-2.80E-02	-2.07E-01	-7.42E-03	-2.05E-01	1.86E-02	-1.88E-01	4.17E-02	-1.63E-01	5.92E-02	-1.37E-01	6.94E-02	-1.13E-01	6.95E-02	-1.03E-01	5.55E-02	-1.03E-01	3.73E-02	-8.97E-02	3.00E-02
-2.03E-01	-3.05E-02	-2.17E-01	-1.14E-02	-2.17E-01	1.35E-02	-1.99E-01	3.73E-02	-1.72E-01	5.65E-02	-1.40E-01	6.59E-02	-1.11E-01	6.75E-02	-9.33E-02	5.25E-02	-8.15E-02	4.42E-02	-6.67E-02	4.02E-02
-1.89E-01	-3.11E-02	-1.99E-01	-7.97E-03	-2.03E-01	1.96E-02	-1.95E-01	4.29E-02	-1.75E-01	5.78E-02	-1.51E-01	6.61E-02	-1.28E-01	6.73E-02	-1.11E-01	5.37E-02	-9.84E-02	4.29E-02	-7.91E-02	3.80E-02
-1.92E-01	-3.28E-02	-2.11E-01	-9.74E-03	-2.11E-01	1.90E-02	-1.94E-01	4.52E-02	-1.67E-01	6.36E-02	-1.38E-01	7.26E-02	-1.14E-01	7.35E-02	-1.03E-01	5.65E-02	-1.01E-01	3.60E-02	-8.92E-02	2.52E-02
-1.98E-01	-3.27E-02	-2.06E-01	-1.03E-02	-2.04E-01	1.83E-02	-1.89E-01	4.45E-02	-1.68E-01	6.46E-02	-1.40E-01	6.99E-02	-1.14E-01	6.99E-02	-9.88E-02	5.33E-02	-8.87E-02	3.92E-02	-6.87E-02	3.58E-02
-1.89E-01	-3.02E-02	-1.98E-01	-1.10E-02	-2.00E-01	1.37E-02	-1.87E-01	3.55E-02	-1.69E-01	5.54E-02	-1.46E-01	6.79E-02	-1.22E-01	7.03E-02	-1.05E-01	5.73E-02	-9.33E-02	4.37E-02	-7.47E-02	3.80E-02
-1.96E-01	-2.81E-02	-2.11E-01	-6.96E-03	-2.13E-01	1.94E-02	-2.02E-01	4.23E-02	-1.79E-01	5.51E-02	-1.52E-01	6.27E-02	-1.25E-01	5.97E-02	-1.06E-01	4.82E-02	-9.38E-02	4.04E-02	-7.48E-02	3.93E-02
-1.84E-01	-3.30E-02	-2.02E-01	-1.11E-02	-2.09E-01	1.48E-02	-1.96E-01	3.62E-02	-1.74E-01	5.59E-02	-1.50E-01	7.05E-02	-1.28E-01	7.58E-02	-1.13E-01	5.95E-02	-9.88E-02	4.76E-02	-7.86E-02	4.34E-02
-1.99E-01	-3.31E-02	-2.10E-01	-6.90E-03	-2.14E-01	2.38E-02	-2.03E-01	4.87E-02	-1.77E-01	6.13E-02	-1.46E-01	6.82E-02	-1.17E-01	6.78E-02	-9.93E-02	5.23E-02	-8.72E-02	4.06E-02	-6.81E-02	3.75E-02
-1.95E-01	-3.01E-02	-2.00E-01	-9.22E-03	-1.98E-01	1.84E-02	-1.86E-01	4.32E-02	-1.66E-01	5.94E-02	-1.44E-01	6.88E-02	-1.23E-01	6.55E-02	-1.09E-01	4.52E-02	-9.53E-02	2.91E-02	-7.63E-02	2.05E-02
-1.92E-01	-4.16E-02	-2.10E-01	-1.62E-02	-2.13E-01	1.75E-02	-2.05E-01	4.84E-02	-1.83E-01	6.78E-02	-1.55E-01	7.46E-02	-1.28E-01	7.31E-02	-1.11E-01	5.60E-02	-1.01E-01	4.03E-02	-7.96E-02	3.48E-02
-2.01E-01	-3.44E-02	-2.14E-01	-1.34E-02	-2.11E-01	1.34E-02	-1.91E-01	3.83E-02	-1.66E-01	5.98E-02	-1.38E-01	7.09E-02	-1.11E-01	6.94E-02	-9.58E-02	5.55E-02	-9.09E-02	3.72E-02	-7.37E-02	3.05E-02
-2.02E-01	-3.14E-02	-2.14E-01	-9.84E-03	-2.12E-01	1.64E-02	-1.94E-01	3.96E-02	-1.65E-01	5.55E-02	-1.34E-01	6.22E-02	-1.07E-01	6.39E-02	-9.64E-02	5.49E-02	-9.78E-02	3.65E-02	-8.30E-02	2.74E-02
-1.86E-01	-1.55E-02	-1.96E-01	6.78E-04	-1.96E-01	1.98E-02	-1.85E-01	3.76E-02	-1.70E-01	5.22E-02	-1.51E-01	6.03E-02	-1.30E-01	5.61E-02	-1.11E-01	4.70E-02	-9.81E-02	3.99E-02	-7.80E-02	3.72E-02
-1.90E-01	-1.67E-02	-1.97E-01	1.80E-03	-1.99E-01	2.43E-02	-1.89E-01	4.22E-02	-1.69E-01	5.50E-02	-1.45E-01	6.15E-02	-1.21E-01	6.49E-02	-1.05E-01	5.36E-02	-9.42E-02	4.27E-02	-7.52E-02	3.70E-02
-2.03E-01	-2.59E-02	-2.14E-01	-6.97E-03	-2.12E-01	1.69E-02	-1.95E-01	3.83E-02	-1.69E-01	5.53E-02	-1.42E-01	6.55E-02	-1.15E-01	6.45E-02	-9.68E-02	5.08E-02	-8.61E-02	3.66E-02	-6.53E-02	3.28E-02
-2.11E-01	-3.36E-02	-2.25E-01	-9.53E-03	-2.22E-01	1.90E-02	-2.00E-01	4.44E-02	-1.64E-01	6.16E-02	-1.27E-01	7.30E-02	-9.49E-02	7.50E-02	-7.92E-02	6.29E-02	-7.61E-02	4.50E-02	-6.39E-02	3.42E-02
-1.91E-01	-2.42E-02	-2.00E-01	-7.98E-03	-1.94E-01	1.09E-02	-1.81E-01	3.30E-02	-1.60E-01	5.08E-02	-1.37E-01	6.42E-02	-1.13E-01	6.82E-02	-9.73E-02	5.61E-02	-8.57E-02	4.49E-02	-6.66E-02	4.03E-02
-1.92E-01	-2.09E-02	-2.03E-01	-2.42E-03	-2.01E-01	2.00E-02	-1.86E-01	4.07E-02	-1.63E-01	5.58E-02	-1.38E-01	6.41E-02	-1.14E-01	6.40E-02	-1.01E-01	5.35E-02	-9.77E-02	3.76E-02	-8.29E-02	3.30E-02
-2.01E-01	-3.53E-02	-2.14E-01	-1.15E-02	-2.13E-01	1.76E-02	-1.96E-01	4.39E-02	-1.66E-01	6.12E-02	-1.35E-01	7.30E-02	-1.07E-01	7.60E-02	-9.01E-02	5.96E-02	-7.98E-02	4.45E-02	-6.29E-02	3.62E-02
-1.98E-01	-2.24E-02	-2.09E-01	-1.06E-03	-2.10E-01	2.35E-02	-1.96E-01	4.38E-02	-1.73E-01	5.86E-02	-1.46E-01	6.52E-02	-1.20E-01	6.28E-02	-1.00E-01	5.18E-02	-8.69E-02	4.01E-02	-6.83E-02	3.53E-02
-2.09E-01	-2.40E-02	-2.17E-01	-5.99E-03	-2.09E-01	1.62E-02	-1.89E-01	3.79E-02	-1.58E-01	5.09E-02	-1.26E-01	5.82E-02	-9.78E-02	6.00E-02	-8.31E-02	5.35E-02	-7.79E-02	4.26E-02	-6.38E-02	3.60E-02
-1.98E-01	-2.01E-02	-2.11E-01	9.86E-04	-2.12E-01	2.51E-02	-1.96E-01	4.52E-02	-1.73E-01	6.07E-02	-1.45E-01	6.57E-02	-1.18E-01	6.51E-02	-9.95E-02	4.87E-02	-8.39E-02	3.69E-02	-6.40E-02	2.94E-02
-2.01E-01	-3.46E-02	-2.13E-01	-1.29E-02	-2.11E-01	1.33E-02	-1.92E-01	3.74E-02	-1.62E-01	5.48E-02	-1.32E-01	7.02E-02	-1.05E-01	7.39E-02	-8.83E-02	5.68E-02	-7.70E-02	4.01E-02	-5.96E-02	3.05E-02
-2.09E-01	-3.36E-02	-2.22E-01	-1.23E-02	-2.18E-01	1.49E-02	-1.98E-01	4.06E-02	-1.70E-01	6.13E-02	-1.38E-01	6.95E-02	-1.07E-01	6.64E-02	-8.82E-02	4.88E-02	-7.51E-02	3.81E-02	-5.51E-02	2.86E-02
-1.94E-01	-1.82E-02	-2.06E-01	-2.49E-03	-2.03E-01	1.81E-02	-1.91E-01	3.99E-02	-1.72E-01	5.54E-02	-1.50E-01	6.18E-02	-1.26E-01	5.66E-02	-1.06E-01	4.25E-02	-9.01E-02	3.48E-02	-6.94E-02	3.13E-02
-2.10E-01	-2.45E-02	-2.15E-01	-5.18E-03	-2.09E-01	1.87E-02	-1.89E-01	3.93E-02	-1.58E-01	4.99E-02	-1.25E-01	5.70E-02	-9.80E-02	6.21E-02	-8.55E-02	5.44E-02	-8.30E-02	3.89E-02	-6.66E-02	3.60E-02
-1.97E-01	-2.14E-02	-2.13E-01	-2.39E-03	-2.13E-01	2.11E-02	-2.00E-01	4.31E-02	-1.74E-01	5.66E-02	-1.47E-01	6.40E-02	-1.19E-01	5.73E-02	-9.69E-02	4.54E-02	-8.14E-02	4.00E-02	-6.16E-02	3.77E-02
-1.88E-01	-2.79E-02	-1.99E-01	-6.44E-03	-2.04E-01	1.83E-02	-1.94E-01	3.90E-02	-1.75E-01	5.40E-02	-1.53E-01	6.42E-02	-1.30E-01	6.39E-02	-1.10E-01	5.12E-02	-9.28E-02	4.13E-02	-7.18E-02	3.71E-02
-1.94E-01	-1.83E-02	-2.08E-01	2.05E-04	-2.07E-01	2.30E-02	-1.96E-01	4.43E-02	-1.74E-01	5.77E-02	-1.48E-01	6.43E-02	-1.23E-01	5.93E-02	-1.02E-01	4.74E-02	-8.80E-02	3.90E-02	-6.97E-02	3.34E-02
-2.00E-01	-3.09E-02	-2.16E-01	-1.07E-02	-2.15E-01	1.29E-02	-1.94E-01	3.37E-02	-1.64E-01	5.15E-02	-1.34E-01	6.82E-02	-1.08E-01	7.34E-02	-9.00E-02	5.78E-02	-7.97E-02	4.08E-02	-5.98E-02	3.58E-02
-1.98E-01	-2.79E-02	-2.16E-01	-3.50E-03	-2.18E-01	2.64E-02	-2.03E-01	5.23E-02	-1.74E-01	6.75E-02	-1.42E-01	7.65E-02	-1.13E-01	7.47E-02	-9.49E-02	5.50E-02	-8.47E-02	3.46E-02	-6.65E-02	2.62E-02
-2.02E-01	-3.41E-02	-2.14E-01	-1.05E-02	-2.13E-01	1.79E-02	-1.94E-01	4.16E-02	-1.65E-01	5.91E-02	-1.35E-01	7.14E-02	-1.09E-01	7.20E-02	-9.32E-02	5.63E-02	-8.72E-02	3.62E-02	-6.93E-02	2.73E-02
-2.03E-01	-2.74E-02	-2.20E-01	-6.61E-03	-2.21E-01	1.86E-02	-2.03E-01	4.17E-02	-1.75E-01	6.05E-02	-1.45E-01	7.17E-02	-1.17E-01	7.33E-02	-9.82E-02	5.60E-02	-8.39E-02	4.06E-02	-6.19E-02	3.35E-02
-1.90E-01	-4.08E-02	-2.05E-01	-1.65E-02	-2.05E-01	1.50E-02	-1.87E-01	4.41E-02	-1.63E-01	6.89E-02	-1.37E-01	8.17E-02	-1.15E-01	8.53E-02	-1.03E-01	6.62E-02	-9.57E-02	4.52E-02	-7.79E-02	3.70E-02
-1.95E-01	-3.68E-02	-2.08E-01	-1.28E-02	-2.10E-01	1.57E-02	-1.93E-01	3.87E-02	-1.66E-01	5.80E-02	-1.38E-01	7.36E-02	-1.16E-01	8.11E-02	-1.01E-01	6.14E-02	-8.32E-02	4.70E-02	-5.87E-02	4.10E-02
-1.97E-01	-3.73E-02	-2.11E-01	-1.42E-02	-2.15E-01	1.41E-02	-1.97E-01	3.85E-02	-1.71E-01	6.12E-02	-1.44E-01	7.49E-02	-1.16E-01	7.43E-02	-9.57E-02	6.13E-02	-8.45E-02	4.63E-02	-6.68E-02	4.04E-02
-1.97E-01	-2.79E-02	-2.11E-01	-7.66E-03	-2.11E-01	1.71E-02	-1.94E-01	3.93E-02	-1.69E-01	5.70E-02	-1.42E-01	6.69E-02	-1.14E-01	6.63E-02	-9.50E-02	5.33E-02	-8.22E-02	4.38E-02	-6.19E-02	4.29E-02
-2.01E-01	-3.46E-02	-2.19E-01	-1.18E-02	-2.22E-01	1.71E-02	-2.05E-01	4.33E-02	-1.78E-01	6.35E-02	-1.46E-01	7.22E-02	-1.17E-01	7.30E-02	-9.86E-02	5.43E-02	-8.69E-02	3.69E-02	-6.51E-02	3.22E-02
-1.96E-01	-3.13E-02	-2.04E-01	-8.92E-03	-1.99E-01	1.85E-02	-1.82E-01	4.37E-02	-1.54E-01	6.14E-02	-1.25E-01	7.18E-02	-9.88E-02	7.53E-02	-8.49E-02	6.40E-02	-8.08E-02	4.67E-02	-6.48E-02	4.38E-02
-1.98E-01	-2.86E-02	-2.12E-01	-3.14E-03	-2.13E-01	2.55E-02	-1.97E-01	4.82E-02	-1.67E-01	5.95E-02	-1.35E-01	6.72E-02	-1.06E-01	6.63E-02	-8.81E-02	5.37E-02	-7.97E-02	3.72E-02	-6.20E-02	3.30E-02
-1.93E-01	-4.04E-02	-2.11E-01	-1.94E-02	-2.14E-01	9.90E-03	-2.01E-01	3.82E-02	-1.75E-01	5.78E-02	-1.46E-01	6.94E-02	-1.19E-01	7.33E-02	-9.94E-02	6.20E-02	-8.63E-02	5.15E-02	-6.79E-02	4.62E-02
-2.07E-01	-4.16E-02	-2.23E-01	-1.70E-02	-2.21E-01	1.38E-02	-1.98E-01	4.11E-02	-1.64E-01	6.13E-02	-1.28E-01	7.36E-02	-9.78E-02	7.63E-02	-8.41E-02	6.71E-02	-8.32E-02	4.63E-02	-6.53E-02	4.15E-02
-1.99E-01	-3.05E-02	-2.11E-01	-8.23E-03	-2.10E-01	1.87E-02	-1.93E-01	4.22E-02	-1.65E-01	5.89E-02	-1.34E-01	6.76E-02	-1.07E-01	6.96E-02	-9.35E-02	6.30E-02	-9.07E-02	4.66E-02	-7.26E-02	4.25E-02
-2.00E-01	-2.98E-02	-2.11E-01	-4.16E-03	-2.07E-01	2.67E-02	-1.88E-01	5.36E-02	-1.59E-01	6.87E-02	-1.29E-01	7.89E-02	-1.06E-01	8.00E-02	-9.76E-02	5.74E-02	-9.56E-02	3.22E-02	-8.15E-02	1.96E-02
-1.88E-01	-3.36E-02	-2.00E-01	-8.62E-03	-2.03E-01	2.01E-02	-1.93E-01	4.42E-02	-1.71E-01	5.94E-02	-1.45E-01	6.80E-02	-1.19E-01	7.09E-02	-1.00E-01	5.87E-02	-8.63E-02	4.75E-02	-6.66E-02	4.27E-02
-1.94E-01	-2.67E-02	-2.06E-01	-5.67E-03	-2.04E-01	1.83E-02	-1.86E-01	3.89E-02	-1.59E-01	5.39E-02	-1.29E-01	6.30E-02	-1.03E-01	6.73E-02	-8.80E-02	5.97E-02	-8.13E-02	4.74E-02	-6.56E-02	4.68E-02
-2.00E-01	-4.21E-02	-2.06E-01	-1.20E-02	-2.14E-01	2.31E-02	-2.03E-01	5.24E-02	-1.77E-01	6.99E-02	-1.46E-01	7.62E-02	-1.18E-01	7.46E-02	-1.01E-01	5.24E-02	-9.00E-02	3.18E-02	-6.74E-02	2.33E-02
-2.01E-01	-3.82E-02	-2.17E-01	-1.34E-02	-2.17E-01	1.74E-02	-1.94E-01	4.47E-02	-1.62E-01	6.89E-02	-1.30E-01	8.49E-02	-1.01E-01	8.46E-02	-8.45E-02	6.71E-02	-8.01E-02	4.57E-02	-6.78E-02	3.33E-02
-2.03E-01	-3.08E-02	-2.24E-01	-3.10E-03	-2.30E-01	2.67E-02	-2.10E-01	4.86E-02	-1.75E-01	6.41E-02	-1.39E-01	7.23E-02	-1.06E-01	7.21E-02	-8.84E-02	5.24E-02	-8.10E-02	3.11E-02	-5.92E-02	2.55E-02
-1.98E-01	-3.14E-02	-2.14E-01	-9.30E-03	-2.10E-01	1.80E-02	-1.91E-01	4.43E-02	-1.63E-01	6.44E-02	-1.36E-01	7.95E-02	-1.13E-01	8.32E-02	-1.00E-01	6.19E-02	-9.15E-02	3.85E-02	-7.59E-02	2.70E-02
-2.00E-01	-3.03E-02	-2.22E-01	-3.55E-03	-2.28E-01	2.81E-02	-2.12E-01	5.48E-02	-1.83E-01	7.05E-02	-1.49E-01	7.36E-02	-1.18E-01	6.92E-02	-1.01E-01	5.60E-02	-9.33E-02	3.87E-02	-7.93E-02	2.68E-02
-2.03E-01	-3.44E-02	-2.27E-01	-7.09E-03	-2.31E-01	2.64E-02	-2.13E-01	5.58E-02	-1.82E-01	7.53E-02	-1.46E-01	8.01E-02	-1.13E-01	7.42E-02	-9.20E-02	5.50E-02	-8.13E-02	3.49E-02	-6.60E-02	2.32E-02
-1.96E-01	-4.22E-02	-2.19E-01	-1.52E-02	-2.27E-01	1.97E-02	-2.14E-01	5.08E-02	-1.82E-01	6.96E-02	-1.47E-01	7.60E-02	-1.16E-01	7.16E-02	-1.01E-01	6.11E-02	-9.69E-02	4.43E-02	-9.24E-02	2.68E-02
-2.00E-01	-3.84E-02	-2.21E-01	-1.25E-02	-2.26E-01	2.23E-02	-2.11E-01	5.34E-02	-1.80E-01	7.18E-02	-1.45E-01	7.58E-02	-1.15E-01	7.20E-02	-9.97E-02	5.85E-02	-9.68E-02	3.69E-02	-8.46E-02	2.12E-02
-2.02E-01	-3.32E-02	-2.22E-01	-5.30E-03	-2.23E-01	2.92E-02	-2.06E-01	5.88E-02	-1.74E-01	7.58E-02	-1.40E-01	7.83E-02	-1.10E-01	7.08E-02	-9.52E-02	5.50E-02	-9.54E-02	3.08E-02	-8.96E-02	1.41E-02
-1.99E-01	-4.03E-02	-2.26E-01	-1.01E-02	-2.33E-01	2.82E-02	-2.17E-01	6.13E-02	-1.83E-01	7.94E-02	-1.44E-01	7.98E-02	-1.09E-01	7.02E-02	-9.01E-02	5.50E-02	-8.49E-02	3.74E-02	-8.33E-02	1.84E-02
-1.88E-01	-4.35E-02	-2.14E-01	-1.40E-02	-2.26E-01	2.21E-02	-2.12E-01	5.50E-02	-1.81E-01	7.65E-02	-1.46E-01	8.42E-02	-1.15E-01	7.84E-02	-9.93E-02	6.06E-02	-9.50E-02	3.98E-02	-8.78E-02	2.07E-02
-1.96E-01	-3.24E-02	-2.13E-01	-6.72E-03	-2.14E-01	2.44E-02	-1.98E-01	5.16E-02	-1.71E-01	6.95E-02	-1.40E-01	7.70E-02	-1.13E-01	7.46E-02	-9.83E-02	6.12E-02	-9.34E-02	4.26E-02	-8.51E-02	2.96E-02
-1.98E-01	-2.84E-02	-2.19E-01	-5.78E-03	-2.24E-01	2.23E-02	-2.11E-01	4.70E-02	-1.86E-01	6.43E-02	-1.57E-01	7.25E-02	-1.28E-01	6.37E-02	-1.04E-01	4.94E-02	-8.80E-02	3.78E-02	-6.53E-02	3.56E-02
-1.94E-01	-3.65E-02	-2.19E-01	-9.04E-03	-2.27E-01	2.74E-02	-2.20E-01	5.84E-02	-1.93E-01	7.55E-02	-1.60E-01	8.08E-02	-1.29E-01	7.61E-02	-1.07E-01	6.26E-02	-9.60E-02	4.54E-02	-7.92E-02	3.46E-02
-2.00E-01	-3.16E-02	-2.19E-01	-8.04E-03	-2.22E-01	2.01E-02	-2.06E-01	4.41E-02	-1.76E-01	5.96E-02	-1.44E-01	6.72E-02	-1.14E-01	6.48E-02	-9.41E-02	5.19E-02	-8.34E-02	3.81E-02	-6.52E-02	3.23E-02
-1.96E-01	-4.60E-02	-2.19E-01	-1.48E-02	-2.30E-01	2.21E-02	-2.16E-01	5.43E-02	-1.86E-01	7.38E-02	-1.48E-01	7.71E-02	-1.15E-01	7.32E-02	-9.71E-02	6.09E-02	-9.21E-02	4.41E-02	-8.42E-02	2.84E-02
-2.00E-01	-3.80E-02	-2.20E-01	-6.90E-03	-2.23E-01	2.97E-02	-2.04E-01	5.96E-02	-1.69E-01	7.56E-02	-1.32E-01	7.70E-02	-1.03E-01	7.33E-02	-9.43E-02	5.86E-02	-1.02E-01	3.33E-02	-9.93E-02	1.43E-02
-1.96E-01	-3.81E-02	-2.22E-01	-8.81E-03	-2.29E-01	2.75E-02	-2.16E-01	5.76E-02	-1.84E-01	7.19E-02	-1.45E-01	7.30E-02	-1.12E-01	6.95E-02	-9.44E-02	5.74E-02	-9.19E-02	3.56E-02	-7.97E-02	2.31E-02
-1.94E-01	-4.05E-02	-2.18E-01	-1.60E-02	-2.23E-01	1.70E-02	-2.09E-01	4.75E-02	-1.82E-01	6.77E-02	-1.49E-01	7.40E-02	-1.19E-01	7.26E-02	-1.03E-01	6.12E-02	-9.81E-02	4.07E-02	-8.09E-02	3.16E-02
-2.04E-01	-3.67E-02	-2.26E-01	-5.97E-03	-2.35E-01	2.93E-02	-2.20E-01	5.75E-02	-1.85E-01	7.12E-02	-1.46E-01	7.42E-02	-1.12E-01	7.10E-02	-9.32E-02	5.94E-02	-8.90E-02	3.78E-02	-7.38E-02	2.38E-02

-2.06E-01	-3.37E-02	-2.27E-01	-5.65E-03	-2.33E-01	2.63E-02	-2.13E-01	5.22E-02	-1.79E-01	6.69E-02	-1.41E-01	7.04E-02	-1.09E-01	6.88E-02	-9.24E-02	5.55E-02	-8.76E-02	3.47E-02	-7.13E-02	2.54E-02
-1.98E-01	-3.19E-02	-2.18E-01	-5.84E-03	-2.23E-01	2.46E-02	-2.07E-01	4.99E-02	-1.78E-01	6.53E-02	-1.45E-01	7.14E-02	-1.15E-01	6.96E-02	-9.63E-02	5.29E-02	-8.67E-02	3.54E-02	-6.75E-02	2.86E-02
-1.89E-01	-4.00E-02	-2.09E-01	-8.12E-03	-2.21E-01	2.60E-02	-2.09E-01	5.41E-02	-1.80E-01	7.05E-02	-1.44E-01	7.33E-02	-1.12E-01	7.50E-02	-9.49E-02	6.44E-02	-8.98E-02	4.72E-02	-7.74E-02	3.47E-02
-2.00E-01	-3.05E-02	-2.20E-01	-2.57E-03	-2.27E-01	2.84E-02	-2.11E-01	5.27E-02	-1.80E-01	6.64E-02	-1.46E-01	7.20E-02	-1.15E-01	6.85E-02	-9.71E-02	5.25E-02	-8.89E-02	3.46E-02	-7.29E-02	2.51E-02
-2.04E-01	-4.13E-02	-2.25E-01	-1.07E-02	-2.30E-01	2.80E-02	-2.15E-01	6.05E-02	-1.80E-01	7.73E-02	-1.42E-01	8.24E-02	-1.10E-01	7.50E-02	-9.17E-02	6.01E-02	-8.73E-02	3.99E-02	-8.47E-02	2.27E-02
-1.97E-01	-4.05E-02	-2.16E-01	-1.17E-02	-2.20E-01	2.46E-02	-2.07E-01	5.56E-02	-1.76E-01	7.24E-02	-1.41E-01	7.96E-02	-1.11E-01	7.62E-02	-9.55E-02	6.23E-02	-9.10E-02	4.56E-02	-8.74E-02	3.07E-02
-2.08E-01	-4.59E-02	-2.33E-01	-1.21E-02	-2.39E-01	2.79E-02	-2.16E-01	6.14E-02	-1.76E-01	8.19E-02	-1.31E-01	8.56E-02	-9.41E-02	8.15E-02	-7.68E-02	6.79E-02	-7.56E-02	4.62E-02	-7.44E-02	2.73E-02
-2.04E-01	-3.32E-02	-2.27E-01	-6.32E-03	-2.29E-01	2.74E-02	-2.11E-01	5.66E-02	-1.76E-01	7.29E-02	-1.38E-01	7.74E-02	-1.05E-01	7.18E-02	-8.70E-02	5.75E-02	-8.12E-02	4.06E-02	-7.58E-02	2.54E-02
-1.96E-01	-3.45E-02	-2.18E-01	-4.92E-03	-2.26E-01	2.94E-02	-2.13E-01	5.75E-02	-1.82E-01	7.25E-02	-1.46E-01	7.63E-02	-1.16E-01	7.46E-02	-9.77E-02	5.97E-02	-9.04E-02	4.02E-02	-7.75E-02	2.66E-02
-2.00E-01	-4.02E-02	-2.24E-01	-1.16E-02	-2.31E-01	2.27E-02	-2.14E-01	5.24E-02	-1.81E-01	7.18E-02	-1.45E-01	8.03E-02	-1.12E-01	7.70E-02	-9.15E-02	6.18E-02	-8.20E-02	4.33E-02	-6.56E-02	3.26E-02
-1.97E-01	-3.19E-02	-2.16E-01	-3.79E-03	-2.23E-01	2.78E-02	-2.10E-01	5.34E-02	-1.82E-01	6.81E-02	-1.50E-01	7.21E-02	-1.19E-01	6.53E-02	-9.68E-02	5.24E-02	-8.48E-02	3.91E-02	-6.93E-02	2.73E-02
-2.03E-01	-3.49E-02	-2.26E-01	-4.92E-03	-2.32E-01	2.87E-02	-2.10E-01	5.50E-02	-1.74E-01	7.19E-02	-1.34E-01	7.47E-02	-1.01E-01	6.96E-02	-8.38E-02	5.55E-02	-8.13E-02	3.61E-02	-7.88E-02	2.01E-02
-1.94E-01	-3.70E-02	-2.19E-01	-1.24E-02	-2.24E-01	2.15E-02	-2.13E-01	5.13E-02	-1.83E-01	6.71E-02	-1.48E-01	7.17E-02	-1.17E-01	7.00E-02	-9.84E-02	5.66E-02	-9.14E-02	3.77E-02	-7.32E-02	2.81E-02
-1.98E-01	-3.92E-02	-2.21E-01	-1.27E-02	-2.27E-01	2.14E-02	-2.11E-01	5.18E-02	-1.79E-01	7.11E-02	-1.48E-01	8.27E-02	-1.20E-01	8.02E-02	-1.04E-01	5.73E-02	-9.83E-02	3.31E-02	-7.55E-02	2.45E-02
-1.98E-01	-3.86E-02	-2.22E-01	-8.84E-03	-2.31E-01	2.63E-02	-2.14E-01	5.62E-02	-1.82E-01	7.54E-02	-1.46E-01	8.16E-02	-1.15E-01	7.69E-02	-9.88E-02	5.94E-02	-9.83E-02	3.36E-02	-7.99E-02	2.15E-02
-2.02E-01	-3.99E-02	-2.29E-01	-8.84E-03	-2.37E-01	3.04E-02	-2.23E-01	6.32E-02	-1.88E-01	7.97E-02	-1.49E-01	8.17E-02	-1.15E-01	7.25E-02	-9.42E-02	5.45E-02	-8.65E-02	3.18E-02	-7.73E-02	1.29E-02
-1.96E-01	-2.88E-02	-2.21E-01	-2.79E-03	-2.31E-01	2.76E-02	-2.18E-01	5.30E-02	-1.90E-01	6.75E-02	-1.57E-01	7.06E-02	-1.27E-01	6.47E-02	-1.07E-01	4.78E-02	-9.60E-02	3.06E-02	-7.93E-02	1.86E-02
-1.99E-01	-2.41E-02	-2.20E-01	-1.95E-03	-2.24E-01	2.55E-02	-2.10E-01	4.89E-02	-1.84E-01	6.27E-02	-1.56E-01	6.81E-02	-1.28E-01	6.20E-02	-1.07E-01	4.50E-02	-9.17E-02	3.01E-02	-7.05E-02	2.42E-02
-1.97E-01	-3.09E-02	-2.16E-01	-7.25E-03	-2.21E-01	2.13E-02	-2.05E-01	4.62E-02	-1.79E-01	6.20E-02	-1.48E-01	6.59E-02	-1.20E-01	6.19E-02	-1.00E-01	5.00E-02	-8.95E-02	3.67E-02	-7.12E-02	3.05E-02
-1.94E-01	-2.95E-02	-2.03E-01	-6.36E-03	-2.06E-01	2.07E-02	-1.92E-01	4.25E-02	-1.68E-01	5.90E-02	-1.42E-01	7.00E-02	-1.18E-01	7.30E-02	-1.03E-01	5.59E-02	-8.96E-02	4.03E-02	-6.79E-02	3.61E-02
-2.02E-01	-3.22E-02	-2.17E-01	-8.75E-03	-2.18E-01	1.95E-02	-2.00E-01	4.41E-02	-1.71E-01	6.32E-02	-1.38E-01	7.11E-02	-1.08E-01	7.17E-02	-9.09E-02	5.96E-02	-8.29E-02	4.58E-02	-6.98E-02	3.56E-02
-1.97E-01	-2.85E-02	-2.14E-01	-1.13E-03	-2.18E-01	3.05E-02	-2.04E-01	5.57E-02	-1.75E-01	6.75E-02	-1.43E-01	7.43E-02	-1.16E-01	7.25E-02	-9.94E-02	5.61E-02	-9.14E-02	4.02E-02	-8.46E-02	2.62E-02
-1.95E-01	-1.75E-02	-2.07E-01	1.10E-03	-2.08E-01	2.28E-02	-1.92E-01	3.93E-02	-1.66E-01	5.21E-02	-1.39E-01	6.42E-02	-1.13E-01	6.62E-02	-9.66E-02	4.89E-02	-7.98E-02	3.80E-02	-5.59E-02	3.75E-02
-2.00E-01	-3.67E-02	-2.13E-01	-1.29E-02	-2.11E-01	1.69E-02	-1.94E-01	4.50E-02	-1.64E-01	6.28E-02	-1.34E-01	7.42E-02	-1.07E-01	7.57E-02	-9.06E-02	5.96E-02	-8.38E-02	3.98E-02	-6.79E-02	2.98E-02
-1.98E-01	-2.83E-02	-2.11E-01	-8.99E-03	-2.09E-01	1.53E-02	-1.92E-01	3.82E-02	-1.68E-01	5.80E-02	-1.43E-01	7.29E-02	-1.19E-01	7.21E-02	-1.00E-01	5.40E-02	-8.45E-02	3.89E-02	-6.17E-02	3.41E-02
-2.00E-01	-1.97E-02	-2.12E-01	-2.48E-03	-2.10E-01	2.07E-02	-1.96E-01	4.37E-02	-1.72E-01	5.98E-02	-1.46E-01	6.52E-02	-1.20E-01	5.48E-02	-9.88E-02	3.90E-02	-8.21E-02	3.06E-02	-6.20E-02	2.78E-02
-2.01E-01	-1.68E-02	-2.16E-01	-9.89E-05	-2.16E-01	1.98E-02	-1.99E-01	3.78E-02	-1.73E-01	5.42E-02	-1.44E-01	6.50E-02	-1.15E-01	5.78E-02	-9.22E-02	4.82E-02	-7.80E-02	4.28E-02	-6.21E-02	3.45E-02
-1.99E-01	-2.22E-02	-2.11E-01	-2.27E-03	-2.10E-01	2.11E-02	-1.95E-01	4.19E-02	-1.71E-01	5.59E-02	-1.44E-01	6.46E-02	-1.19E-01	6.67E-02	-1.05E-01	5.34E-02	-9.75E-02	3.71E-02	-8.03E-02	2.84E-02
-2.01E-01	-1.93E-02	-2.20E-01	5.41E-04	-2.23E-01	2.40E-02	-2.05E-01	4.37E-02	-1.80E-01	6.26E-02	-1.50E-01	6.91E-02	-1.21E-01	6.57E-02	-1.01E-01	4.64E-02	-8.50E-02	3.12E-02	-6.46E-02	2.19E-02
-1.96E-01	-3.85E-02	-2.04E-01	-1.29E-02	-2.04E-01	1.80E-02	-1.86E-01	4.41E-02	-1.59E-01	6.41E-02	-1.31E-01	7.97E-02	-1.06E-01	8.67E-02	-9.59E-02	7.87E-02	-9.47E-02	5.81E-02	-8.02E-02	5.03E-02
-2.00E-01	-1.95E-02	-2.15E-01	8.46E-04	-2.14E-01	2.41E-02	-1.96E-01	4.33E-02	-1.68E-01	5.61E-02	-1.37E-01	6.35E-02	-1.12E-01	6.59E-02	-9.68E-02	4.57E-02	-8.13E-02	3.18E-02	-5.99E-02	2.69E-02
-1.97E-01	-1.98E-02	-2.08E-01	-1.54E-04	-2.07E-01	2.41E-02	-1.95E-01	4.57E-02	-1.73E-01	5.95E-02	-1.48E-01	6.67E-02	-1.22E-01	6.31E-02	-1.03E-01	4.90E-02	-9.04E-02	3.38E-02	-6.96E-02	2.89E-02
-2.00E-01	-2.90E-02	-2.14E-01	-6.45E-03	-2.16E-01	2.08E-02	-2.00E-01	4.39E-02	-1.71E-01	6.01E-02	-1.41E-01	7.15E-02	-1.11E-01	7.11E-02	-9.16E-02	5.66E-02	-7.96E-02	4.32E-02	-6.18E-02	3.59E-02
-2.01E-01	-3.04E-02	-2.13E-01	-5.53E-03	-2.17E-01	2.26E-02	-2.00E-01	4.43E-02	-1.71E-01	6.00E-02	-1.41E-01	7.21E-02	-1.14E-01	7.60E-02	-9.69E-02	6.41E-02	-8.98E-02	4.50E-02	-7.03E-02	3.87E-02
-1.95E-01	-2.49E-02	-2.09E-01	-3.13E-03	-2.09E-01	2.29E-02	-1.95E-01	4.55E-02	-1.72E-01	6.10E-02	-1.45E-01	6.64E-02	-1.21E-01	6.50E-02	-1.07E-01	5.59E-02	-1.02E-01	4.37E-02	-9.01E-02	3.37E-02
-1.88E-01	-1.38E-02	-2.00E-01	3.00E-03	-2.01E-01	2.26E-02	-1.90E-01	4.06E-02	-1.70E-01	5.36E-02	-1.48E-01	6.25E-02	-1.23E-01	5.78E-02	-1.02E-01	4.90E-02	-8.79E-02	4.17E-02	-6.87E-02	3.54E-02
-1.95E-01	-2.14E-02	-2.07E-01	-1.79E-03	-2.07E-01	2.15E-02	-1.94E-01	4.16E-02	-1.72E-01	5.60E-02	-1.48E-01	6.67E-02	-1.24E-01	6.57E-02	-1.07E-01	4.91E-02	-9.06E-02	3.65E-02	-7.20E-02	2.68E-02
-1.98E-01	-1.90E-02	-2.09E-01	2.80E-05	-2.09E-01	2.37E-02	-1.94E-01	4.47E-02	-1.69E-01	5.91E-02	-1.43E-01	6.82E-02	-1.17E-01	6.22E-02	-9.78E-02	4.47E-02	-8.20E-02	3.46E-02	-6.17E-02	2.91E-02
-2.01E-01	-4.39E-02	-2.13E-01	-1.77E-02	-2.16E-01	1.50E-02	-2.01E-01	4.33E-02	-1.70E-01	6.15E-02	-1.40E-01	7.68E-02	-1.11E-01	8.14E-02	-9.41E-02	6.61E-02	-8.60E-02	4.78E-02	-6.53E-02	4.54E-02
-2.05E-01	-2.91E-02	-2.17E-01	-8.17E-03	-2.12E-01	1.71E-02	-1.91E-01	4.02E-02	-1.59E-01	5.63E-02	-1.25E-01	6.87E-02	-9.72E-02	7.43E-02	-8.39E-02	6.55E-02	-8.17E-02	4.78E-02	-6.60E-02	4.37E-02
-1.94E-01	-2.83E-02	-2.13E-01	-3.20E-03	-2.19E-01	2.60E-02	-2.08E-01	5.05E-02	-1.84E-01	6.67E-02	-1.56E-01	7.34E-02	-1.28E-01	6.88E-02	-1.08E-01	5.33E-02	-9.33E-02	4.19E-02	-7.34E-02	3.39E-02
-1.94E-01	-4.01E-02	-2.03E-01	-1.51E-02	-2.03E-01	1.57E-02	-1.87E-01	4.28E-02	-1.60E-01	6.34E-02	-1.31E-01	7.82E-02	-1.04E-01	8.24E-02	-8.98E-02	7.87E-02	-8.59E-02	6.31E-02	-7.31E-02	5.63E-02
-1.95E-01	-3.26E-02	-2.08E-01	-9.38E-03	-2.11E-01	1.83E-02	-1.98E-01	4.19E-02	-1.73E-01	5.77E-02	-1.42E-01	6.38E-02	-1.13E-01	6.67E-02	-9.36E-02	5.66E-02	-8.24E-02	4.67E-02	-6.32E-02	4.39E-02
-1.95E-01	-2.41E-02	-2.03E-01	-6.95E-03	-2.01E-01	1.53E-02	-1.86E-01	3.66E-02	-1.64E-01	5.46E-02	-1.40E-01	6.50E-02	-1.16E-01	6.31E-02	-9.82E-02	5.04E-02	-8.69E-02	3.79E-02	-7.03E-02	3.21E-02
-1.93E-01	-2.30E-02	-2.08E-01	-1.61E-03	-2.09E-01	2.37E-02	-1.98E-01	4.52E-02	-1.77E-01	5.69E-02	-1.49E-01	5.64E-02	-1.22E-01	4.95E-02	-1.01E-01	4.20E-02	-8.94E-02	3.75E-02	-7.19E-02	3.48E-02
-1.95E-01	-2.45E-02	-1.99E-01	-2.62E-03	-1.93E-01	2.41E-02	-1.79E-01	4.78E-02	-1.56E-01	6.26E-02	-1.30E-01	6.88E-02	-1.07E-01	6.87E-02	-9.51E-02	5.97E-02	-9.22E-02	4.56E-02	-7.86E-02	3.97E-02
-1.96E-01	-2.25E-02	-2.09E-01	3.13E-03	-2.18E-01	2.97E-02	-2.07E-01	5.03E-02	-1.85E-01	6.40E-02	-1.59E-01	6.79E-02	-1.31E-01	6.11E-02	-1.08E-01	4.75E-02	-8.93E-02	3.98E-02	-6.60E-02	3.85E-02
-1.95E-01	-3.66E-02	-2.06E-01	-9.31E-03	-2.10E-01	2.31E-02	-1.99E-01	5.07E-02	-1.77E-01	6.82E-02	-1.51E-01	7.47E-02	-1.23E-01	6.80E-02	-1.02E-01	5.43E-02	-8.84E-02	4.25E-02	-6.73E-02	3.88E-02
-1.92E-01	-1.97E-02	-2.01E-01	-2.16E-03	-1.98E-01	1.93E-02	-1.82E-01	3.95E-02	-1.60E-01	5.64E-02	-1.40E-01	6.89E-02	-1.20E-01	6.09E-02	-1.01E-01	4.61E-02	-8.40E-02	4.17E-02	-6.29E-02	4.16E-02
-1.95E-01	-3.57E-02	-2.12E-01	-6.14E-03	-2.17E-01	2.73E-02	-2.03E-01	5.46E-02	-1.75E-01	7.20E-02	-1.44E-01	7.95E-02	-1.14E-01	7.43E-02	-9.31E-02	5.57E-02	-8.15E-02	3.68E-02	-6.13E-02	3.12E-02
-2.11E-01	-3.88E-02	-2.33E-01	-1.31E-02	-2.33E-01	1.85E-02	-2.09E-01	4.62E-02	-1.71E-01	6.50E-02	-1.31E-01	7.42E-02	-9.93E-02	7.72E-02	-8.29E-02	6.01E-02	-7.86E-02	3.71E-02	-6.21E-02	2.69E-02
-2.05E-01	-4.38E-02	-2.26E-01	-1.32E-02	-2.27E-01	2.28E-02	-2.01E-01	5.24E-02	-1.59E-01	6.99E-02	-1.18E-01	7.78E-02	-8.80E-02	8.06E-02	-7.80E-02	6.50E-02	-8.27E-02	3.90E-02	-7.83E-02	2.13E-02
-1.98E-01	-3.73E-02	-2.25E-01	-8.78E-03	-2.32E-01	2.53E-02	-2.14E-01	5.32E-02	-1.77E-01	6.64E-02	-1.36E-01	6.76E-02	-1.03E-01	6.91E-02	-8.89E-02	5.84E-02	-9.24E-02	3.14E-02	-7.32E-02	2.02E-02
-2.05E-01	-2.61E-02	-2.20E-01	1.24E-03	-2.19E-01	3.20E-02	-2.00E-01	5.51E-02	-1.66E-01	6.32E-02	-1.30E-01	6.36E-02	-1.02E-01	6.57E-02	-8.80E-02	4.77E-02	-8.37E-02	2.84E-02	-8.00E-02	1.28E-02
-2.05E-01	-2.96E-02	-2.22E-01	-1.81E-03	-2.23E-01	3.05E-02	-2.02E-01	5.53E-02	-1.67E-01	6.65E-02	-1.29E-01	6.96E-02	-9.67E-02	6.59E-02	-7.95E-02	5.11E-02	-7.65E-02	3.19E-02	-6.62E-02	1.91E-02
-2.07E-01	-4.10E-02	-2.30E-01	-1.36E-02	-2.31E-01	2.24E-02	-2.08E-01	5.52E-02	-1.71E-01	7.57E-02	-1.32E-01	8.49E-02	-1.01E-01	8.11E-02	-8.42E-02	5.82E-02	-8.08E-02	3.19E-02	-6.86E-02	1.67E-02
-2.03E-01	-2.77E-02	-2.16E-01	-3.24E-03	-2.15E-01	2.47E-02	-1.99E-01	4.71E-02	-1.70E-01	5.70E-02	-1.39E-01	6.42E-02	-1.13E-01	6.63E-02	-9.67E-02	5.15E-02	-8.86E-02	3.25E-02	-7.12E-02	2.38E-02
-2.02E-01	-3.11E-02	-2.17E-01	-4.26E-03	-2.19E-01	2.56E-02	-2.01E-01	4.89E-02	-1.70E-01	6.32E-02	-1.38E-01	7.22E-02	-1.09E-01	6.96E-02	-9.04E-02	5.18E-02	-8.06E-02	3.23E-02	-6.23E-02	2.24E-02
-2.03E-01	-3.80E-02	-2.23E-01	-1.14E-02	-2.26E-01	2.26E-02	-2.08E-01	5.21E-02	-1.72E-01	6.82E-02	-1.35E-01	7.63E-02	-1.02E-01	7.44E-02	-8.57E-02	5.65E-02	-8.31E-02	3.34E-02	-6.41E-02	2.43E-02
-2.01E-01	-3.84E-02	-2.19E-01	-8.76E-03	-2.25E-01	2.38E-02	-2.07E-01	4.99E-02	-1.74E-01	6.59E-02	-1.40E-01	7.52E-02	-1.10E-01	7.06E-02	-9.29E-02	5.58E-02	-8.74E-02	3.54E-02	-7.40E-02	2.28E-02
-1.96E-01	-4.09E-02	-2.08E-01	-7.20E-03	-2.23E-01	2.58E-02	-2.14E-01	5.29E-02	-1.84E-01	6.84E-02	-1.50E-01	7.48E-02	-1.19E-01	7.19E-02	-9.68E-02	5.73E-02	-8.39E-02	4.06E-02	-6.87E-02	2.78E-02
-2.12E-01	-3.21E-02	-2.32E-01	-2.03E-03	-2.31E-01	3.22E-02	-2.06E-01	5.87E-02	-1.67E-01	7.06E-02	-1.25E-01	6.84E-02	-9.18E-02	6.83E-02	-8.25E-02	5.72E-02	-9.41E-02	2.93E-02	-7.78E-02	1.34E-02
-2.04E-01	-3.39E-02	-2.18E-01	-6.99E-03	-2.19E-01	2.65E-02	-2.04E-01	5.51E-02	-1.77E-01	7.05E-02	-1.46E-01	7.18E-02	-1.17E-01	6.39E-02	-9.61E-02	4.36E-02	-8.22E-02	2.60E-02	-6.08E-02	2.06E-02
-1.96E-01	-3.92E-02	-2.12E-01	-8.21E-03	-2.17E-01	2.85E-02	-2.02E-01	5.91E-02	-1.74E-01	7.67E-02	-1.41E-01	8.15E-02	-1.11E-01	7.58E-02	-9.35E-02	5.03E-02	-8.30E-02	2.69E-02	-6.11E-02	1.68E-02
-2.03E-01	-2.08E-02	-2.25E-01	3.29E-03	-2.27E-01	3.05E-02	-2.07E-01	5.27E-02	-1.72E-01	6.45E-02	-1.36E-01	7.06E-02	-1.05E-01	6.73E-02	-8.91E-02	4.53E-02	-8.41E-02	2.15E-02	-6.54E-02	1.03E-02
-1.97E-01	-3.98E-02	-2.13E-01	-1.43E-02	-2.15E-01	1.67E-02	-1.97E-01	4.37E-02	-1.70E-01	6.49E-02	-1.40E-01	7.59E-02	-1.14E-01	7.93E-02	-9.65E-02	6.08E-02	-8.39E-02	4.25E-02	-6.46E-02	3.65E-02
-2.02E-01	-3.62E-02	-2.19E-01	-9.40E-03	-2.19E-01	2.40E-02	-2.01E-01	5.29E-02	-1.69E-01	6.98E-02	-1.34E-01	7.54E-02	-1.03E-01	6.86E-02	-8.55E-02	5.02E-02	-7.95E-02	2.99E-02	-6.43E-02	2.13E-02
-2.04E-01	-2.86E-02	-2.19E-01	-4.40E-03	-2.18E-01	2.44E-02	-1.97E-01	4.89E-02	-1.67E-01	6.55E-02	-1.38E-01	7.49E-02	-1.10E-01	6.90E-02	-9.47E-02	4.84E-02	-8.58E-02	3.03E-02	-6.99E-02	1.84E-02
-2.02E-01	-3.99E-02	-2.14E-01	-1.08E-02	-2.16E-01	2.33E-02	-2.03E-01	5.05E-02	-1.71E-01	6.24E-02	-1.37E-01	6.64E-02	-1.06E-01	6.44E-02	-9.09E-02	5.22E-02	-8.71E-02	3.31E-02	-7.05E-02	2.53E-02
-2.03E-01	-4.30E-02	-2.25E-01	-1.27E-02	-2.32E-01	2.35E-02	-2.14E-01	5.38E-02	-1.78E-01	7.19E-02	-1.39E-01	7.94E-02	-1.06E-01	7.63E-02	-8.56E-02	5.44E-02	-7.70E-02	3.17E-02	-6.11E-02	1.95E-02
-2.01E-01	-4.36E-02	-2.16E-01	-1.15E-02	-2.19E-01	2.64E-02	-2.02E-01	5.72E-02	-1.70E-01	7.48E-02	-1.35E-01	8.18E-02	-1.07E-01	8.28E-02	-9.38E-02	5.39E-02	-7.84E-02	2.93E-02	-5.62E-02	1.98E-02
-2.08E-01	-3.43E-02	-2.29E-01	-8.17E-03	-2.26E-01	2.59E-02	-2.11E-01	5.84E-02	-1.77E-01	7.24E-02	-1.39E-01	7.42E-02	-1.05E-01	7.19E-02	-8.55E-02	5.43E-02	-7.77E-02	3.25E-02	-6.11E-02	2.14E-02
-2.01E-01	-3.18E-02	-2.15E-01	-4.72E-03	-2.13E-01	2.74E-02	-1.96E-01	5.56E-02	-1.66E-01	7.11E-02	-1.33E-01	7.54E-02	-1.05E-01	7.70E-02	-9.43E-02	5.16E-02	-8.30E-02	2.82E-02	-6.29E-02	1.79E-02
-2.03E-01	-4.27E-02	-2.22E-01	-1.33E-02	-2.26E-01	2.30E-02	-2.08E-01	5.38E-02	-1.73E-01	7.01E-02	-1.37E-01	7.88E-02	-1.07E-01	8.00E-02	-8.92E-02	5.81E-02	-8.13E-02	3.40E-02	-6.45E-02	2.24E-02
-2.01E-01	-3.76E-02	-2.12E-01	-5.45E-03	-2.03E-01	3.03E-02	-1.78E-01	6.17E-02	-1.44E-01	7.95E-02	-1.12E-01	8.85E-02	-8.81E-02	7.54E-02	-8.46E-02	4.88E-02	-1.03E-01	2.35E-02	-1.00E-01	1.23E-02
-2.01E-01	-3.17E-02	-2.22E-01	-5.20E-03	-2.24E-01	2.68E-02	-2.04E-01	5.43E-02	-1.71E-01	7.13E-02	-1.37E-01	7.77E-02	-1.09E-01	7.84E-02	-9.37E-02	5.34E-02	-8.34E-02	2.94E-02	-6.53E-02	1.67E-02
-2.04E-01	-3.39E-02	-2.24E-01	-9.06E-03	-2.19E-01	2.28E-02	-1.96E-01	5.31E-02	-1.59E-01	7.16E-02	-1.24E-01	8.09E-02	-9.46E-02	7.63E-02	-8.14E-02	5.56E-02	-7.98E-02	3.43E-02	-7.77E-02	1.84E-02
-2.01E-01	-3.63E-02	-2.24E-01	-1.30E-02	-2.26E-01	1.76E-02	-2.05E-01	4.57E-02	-1.72E-01	6.67E-02	-1.38E-01	7.91E-02	-1.11E-01	8.12E-02	-9.44E-02	5.85E-02	-8.19E-02	3.93E-02	-6.92E-02	2.55E-02
-2.10E-01	-4.60E-02	-2.26E-01	-1.46E-02	-2.24E-01	2.46E-02	-1.97E-01	5.81E-02	-1.60E-01	8.28E-02	-1.22E-01	9.20E-02	-9.09E-02	8.35E-02	-7.67E-02	5.95E-02	-7.79E-02	3.29E-02	-7.76E-02	1.73E-02
-2.16E-01	-3.40E-02	-2.33E-01	-8.00E-03	-2.29E-01	2.37E-02	-2.02E-01	5.03E-02	-1.64E-01	6.73E-02	-1.25E-01	7.38E-02	-9.37E-02	7.12E-02	-7.99E-02	4.88E-02	-7.85E-02	2.36E-02	-6.78E-02	8.14E-03
-2.02E-01	-3.63E-02	-2.21E-01	-1.42E-02	-2.20E-01	1.54E-02	-2.00E-01	4.40E-02	-1.70E-01	6.62E-02	-1.38E-01	7.63E-02	-1.09E-01	7.35E-02	-9.13E-02	5.37E-02	-8.07E-02	3.58E-02	-6.34E-02	2.54E-02
-2.06E-01	-4.23E-02	-2.27E-01	-1.25E-02	-2.27E-01	2.54E-02	-2.04E-01	5.83E-02	-1.66E-01	7.85E-02	-1.27E-01	8.55E-02	-9.53E-02	7.86E-02	-8.14E-02	5.62E-02	-8.29E-02	3.13E-02	-8.14E-02	1.24E-02
-2.04E-01	-3.44E-02	-2.21E-01	-8.47E-03	-2.22E-01	2.37E-02	-1.99E-01	5.11E-02	-1.66E-01	7.08E-02	-1.29E-01	7.62E-02	-9.95E-02	7.30E-02	-8.36E-02	5.38E-02	-7.86E-02	3.42E-02	-7.41E-02	1.85E-02

-1.97E-01	-3.73E-02	-2.14E-01	-9.49E-03	-2.19E-01	2.28E-02	-2.03E-01	4.94E-02	-1.73E-01	6.65E-02	-1.40E-01	7.51E-02	-1.10E-01	7.48E-02	-9.10E-02	5.65E-02	-7.83E-02	3.97E-02	-6.08E-02	3.05E-02
-2.03E-01	-2.88E-02	-2.18E-01	-3.07E-03	-2.17E-01	2.76E-02	-1.97E-01	5.35E-02	-1.66E-01	7.06E-02	-1.33E-01	7.68E-02	-1.04E-01	7.28E-02	-8.76E-02	5.21E-02	-8.04E-02	3.11E-02	-6.97E-02	1.77E-02
-1.97E-01	-3.90E-02	-2.15E-01	-1.40E-02	-2.16E-01	1.75E-02	-1.97E-01	4.59E-02	-1.66E-01	6.56E-02	-1.34E-01	7.84E-02	-1.06E-01	7.79E-02	-9.16E-02	5.85E-02	-8.81E-02	3.58E-02	-7.43E-02	2.28E-02
-2.04E-01	-2.31E-02	-2.21E-01	2.78E-03	-2.20E-01	3.33E-02	-2.02E-01	5.88E-02	-1.70E-01	6.97E-02	-1.36E-01	7.13E-02	-1.07E-01	6.06E-02	-8.89E-02	4.33E-02	-8.45E-02	2.34E-02	-7.38E-02	1.01E-02
-2.04E-01	-4.89E-02	-2.19E-01	-2.30E-02	-2.16E-01	1.21E-02	-1.95E-01	4.55E-02	-1.65E-01	7.10E-02	-1.33E-01	8.30E-02	-1.04E-01	8.06E-02	-8.75E-02	6.17E-02	-8.13E-02	3.83E-02	-6.90E-02	2.45E-02
-2.00E-01	-2.63E-02	-2.15E-01	-4.09E-03	-2.15E-01	2.31E-02	-1.98E-01	4.68E-02	-1.72E-01	6.32E-02	-1.41E-01	6.69E-02	-1.13E-01	6.10E-02	-9.29E-02	4.71E-02	-8.26E-02	3.27E-02	-6.62E-02	2.39E-02
-2.02E-01	-3.27E-02	-2.18E-01	-6.69E-03	-2.18E-01	2.57E-02	-1.99E-01	5.33E-02	-1.67E-01	7.09E-02	-1.35E-01	7.95E-02	-1.06E-01	7.27E-02	-8.90E-02	4.96E-02	-8.20E-02	2.65E-02	-6.57E-02	1.43E-02
-2.00E-01	-3.13E-02	-2.16E-01	-3.70E-03	-2.21E-01	2.77E-02	-2.03E-01	5.21E-02	-1.72E-01	6.66E-02	-1.38E-01	7.35E-02	-1.10E-01	7.10E-02	-9.12E-02	5.05E-02	-7.78E-02	3.41E-02	-6.78E-02	1.84E-02
-2.07E-01	-3.34E-02	-2.26E-01	-9.74E-03	-2.23E-01	2.23E-02	-2.04E-01	5.34E-02	-1.73E-01	7.17E-02	-1.37E-01	7.42E-02	-1.08E-01	7.52E-02	-9.26E-02	5.62E-02	-8.86E-02	3.23E-02	-7.21E-02	2.07E-02
-1.99E-01	-3.35E-02	-2.13E-01	-6.82E-03	-2.09E-01	2.55E-02	-1.92E-01	5.55E-02	-1.66E-01	7.54E-02	-1.36E-01	7.90E-02	-1.08E-01	7.31E-02	-9.25E-02	5.01E-02	-8.49E-02	2.81E-02	-6.58E-02	2.04E-02
-1.83E-01	-4.41E-02	-2.15E-01	-1.76E-02	-2.29E-01	1.78E-02	-2.15E-01	5.02E-02	-1.81E-01	7.05E-02	-1.41E-01	7.83E-02	-1.07E-01	7.77E-02	-8.87E-02	5.69E-02	-8.49E-02	3.31E-02	-7.07E-02	1.57E-02
-1.98E-01	-4.79E-02	-2.22E-01	-1.91E-02	-2.29E-01	1.79E-02	-2.12E-01	5.06E-02	-1.76E-01	7.09E-02	-1.36E-01	8.00E-02	-1.02E-01	7.81E-02	-8.31E-02	5.98E-02	-7.82E-02	3.77E-02	-6.48E-02	2.13E-02
-2.00E-01	-3.04E-02	-2.17E-01	-2.59E-03	-2.22E-01	2.83E-02	-2.06E-01	5.21E-02	-1.77E-01	6.54E-02	-1.44E-01	6.98E-02	-1.14E-01	6.92E-02	-9.42E-02	4.83E-02	-7.95E-02	3.02E-02	-5.58E-02	2.90E-02
-2.02E-01	-3.11E-02	-2.18E-01	-4.37E-03	-2.17E-01	2.65E-02	-1.99E-01	5.17E-02	-1.66E-01	6.47E-02	-1.33E-01	7.07E-02	-1.04E-01	7.07E-02	-9.21E-02	4.84E-02	-8.91E-02	2.50E-02	-6.95E-02	1.82E-02
-2.04E-01	-2.60E-02	-2.22E-01	1.01E-03	-2.23E-01	3.23E-02	-2.03E-01	5.75E-02	-1.72E-01	7.20E-02	-1.37E-01	7.18E-02	-1.06E-01	6.48E-02	-8.63E-02	4.70E-02	-7.76E-02	2.79E-02	-6.40E-02	1.69E-02
-2.06E-01	-4.01E-02	-2.23E-01	-8.83E-03	-2.22E-01	2.78E-02	-1.99E-01	5.75E-02	-1.60E-01	7.23E-02	-1.22E-01	8.11E-02	-9.19E-02	8.05E-02	-8.30E-02	6.22E-02	-9.20E-02	3.60E-02	-7.18E-02	2.62E-02
-2.01E-01	-3.78E-02	-2.24E-01	-9.72E-03	-2.25E-01	2.39E-02	-2.02E-01	5.24E-02	-1.65E-01	7.12E-02	-1.28E-01	8.27E-02	-9.75E-02	8.13E-02	-8.60E-02	5.99E-02	-9.05E-02	3.29E-02	-7.84E-02	1.71E-02
-2.05E-01	-4.15E-02	-2.22E-01	-1.20E-02	-2.20E-01	2.41E-02	-1.96E-01	5.38E-02	-1.56E-01	7.05E-02	-1.18E-01	8.01E-02	-9.05E-02	8.12E-02	-8.01E-02	6.17E-02	-8.37E-02	3.46E-02	-8.04E-02	1.71E-02
-2.09E-01	-3.48E-02	-2.25E-01	-7.56E-03	-2.23E-01	2.56E-02	-2.00E-01	5.34E-02	-1.62E-01	6.77E-02	-1.24E-01	7.46E-02	-9.31E-02	7.49E-02	-7.77E-02	5.72E-02	-7.41E-02	3.47E-02	-6.35E-02	2.01E-02
-2.05E-01	-4.38E-02	-2.27E-01	-1.33E-02	-2.28E-01	2.40E-02	-2.02E-01	5.55E-02	-1.60E-01	7.32E-02	-1.18E-01	8.18E-02	-8.54E-02	7.92E-02	-7.45E-02	5.94E-02	-7.98E-02	3.45E-02	-8.17E-02	1.68E-02
-2.16E-01	-3.67E-02	-2.29E-01	-1.04E-02	-2.23E-01	2.30E-02	-1.92E-01	4.97E-02	-1.51E-01	6.77E-02	-1.14E-01	7.82E-02	-8.58E-02	7.22E-02	-7.91E-02	5.07E-02	-8.90E-02	2.69E-02	-9.51E-02	1.07E-02
-2.08E-01	-2.99E-02	-2.25E-01	-5.45E-03	-2.23E-01	2.44E-02	-2.05E-01	5.11E-02	-1.73E-01	6.59E-02	-1.37E-01	6.85E-02	-1.05E-01	6.55E-02	-8.48E-02	5.03E-02	-7.64E-02	3.20E-02	-5.99E-02	2.35E-02
-2.06E-01	-3.21E-02	-2.23E-01	-5.38E-03	-2.24E-01	2.57E-02	-2.04E-01	5.07E-02	-1.71E-01	6.49E-02	-1.35E-01	6.94E-02	-1.07E-01	7.24E-02	-9.22E-02	5.09E-02	-8.45E-02	2.88E-02	-6.69E-02	1.79E-02
-2.12E-01	-3.29E-02	-2.33E-01	-1.02E-02	-2.27E-01	1.94E-02	-1.97E-01	4.71E-02	-1.56E-01	6.72E-02	-1.17E-01	7.70E-02	-8.88E-02	7.41E-02	-8.13E-02	5.49E-02	-8.90E-02	3.22E-02	-9.59E-02	1.60E-02
-2.03E-01	-3.76E-02	-2.16E-01	-1.28E-02	-2.13E-01	1.78E-02	-1.93E-01	4.49E-02	-1.62E-01	6.30E-02	-1.28E-01	7.02E-02	-9.91E-02	7.04E-02	-8.44E-02	5.92E-02	-8.01E-02	4.26E-02	-6.72E-02	3.50E-02
-2.04E-01	-2.80E-02	-2.14E-01	-6.21E-03	-2.11E-01	1.99E-02	-1.94E-01	4.19E-02	-1.66E-01	5.49E-02	-1.36E-01	6.00E-02	-1.09E-01	5.97E-02	-9.47E-02	5.26E-02	-9.00E-02	3.83E-02	-7.35E-02	3.55E-02
-2.05E-01	-3.51E-02	-2.22E-01	-1.02E-02	-2.23E-01	1.92E-02	-2.03E-01	4.36E-02	-1.70E-01	6.06E-02	-1.34E-01	6.86E-02	-1.03E-01	6.84E-02	-8.44E-02	5.28E-02	-7.50E-02	3.70E-02	-5.71E-02	3.23E-02
-2.00E-01	-4.02E-02	-2.19E-01	-2.04E-02	-2.18E-01	8.24E-03	-2.00E-01	3.61E-02	-1.68E-01	5.40E-02	-1.34E-01	6.50E-02	-1.05E-01	6.82E-02	-8.78E-02	5.79E-02	-7.96E-02	4.84E-02	-6.50E-02	4.47E-02
-2.00E-01	-4.21E-02	-2.16E-01	-1.87E-02	-2.15E-01	1.26E-02	-1.99E-01	4.19E-02	-1.69E-01	5.98E-02	-1.35E-01	6.77E-02	-1.05E-01	7.02E-02	-8.70E-02	5.71E-02	-7.71E-02	4.52E-02	-5.93E-02	4.31E-02
-1.97E-01	-5.16E-02	-2.18E-01	-2.32E-02	-2.22E-01	1.48E-02	-2.07E-01	4.98E-02	-1.76E-01	7.08E-02	-1.36E-01	7.47E-02	-1.01E-01	7.19E-02	-8.04E-02	6.15E-02	-7.18E-02	4.97E-02	-6.22E-02	3.31E-02
-2.08E-01	-4.24E-02	-2.21E-01	-1.92E-02	-2.18E-01	1.22E-02	-1.99E-01	4.16E-02	-1.65E-01	5.95E-02	-1.29E-01	6.98E-02	-9.96E-02	7.78E-02	-8.39E-02	6.99E-02	-7.80E-02	5.22E-02	-6.04E-02	4.82E-02
-2.02E-01	-2.96E-02	-2.15E-01	-8.82E-03	-2.12E-01	1.71E-02	-1.94E-01	4.11E-02	-1.65E-01	5.72E-02	-1.34E-01	6.47E-02	-1.04E-01	6.03E-02	-8.50E-02	5.44E-02	-7.48E-02	5.17E-02	-5.97E-02	5.01E-02
-2.10E-01	-3.21E-02	-2.22E-01	-8.37E-03	-2.19E-01	2.00E-02	-1.99E-01	4.41E-02	-1.66E-01	5.85E-02	-1.32E-01	6.62E-02	-1.03E-01	6.83E-02	-8.65E-02	5.93E-02	-7.98E-02	4.34E-02	-6.28E-02	3.85E-02
-1.95E-01	-4.30E-02	-2.14E-01	-2.05E-02	-2.15E-01	8.79E-03	-1.93E-01	3.71E-02	-1.73E-01	6.62E-02	-1.38E-01	7.26E-02	-1.06E-01	7.33E-02	-8.45E-02	6.50E-02	-7.42E-02	5.59E-02	-6.09E-02	4.45E-02
-1.95E-01	-4.07E-02	-2.16E-01	-1.66E-02	-2.18E-01	1.53E-02	-2.01E-01	4.52E-02	-1.71E-01	6.56E-02	-1.38E-01	7.61E-02	-1.09E-01	7.65E-02	-9.18E-02	6.10E-02	-8.39E-02	4.21E-02	-6.61E-02	3.27E-02
-2.00E-01	-3.88E-02	-2.13E-01	-1.31E-02	-2.15E-01	1.81E-02	-2.00E-01	4.50E-02	-1.72E-01	6.17E-02	-1.40E-01	6.92E-02	-1.11E-01	7.10E-02	-9.19E-02	5.71E-02	-8.00E-02	4.51E-02	-6.14E-02	4.42E-02
-2.02E-01	-4.76E-02	-2.21E-01	-2.58E-02	-2.20E-01	6.20E-03	-2.01E-01	3.90E-02	-1.71E-01	6.36E-02	-1.37E-01	7.64E-02	-1.05E-01	7.55E-02	-8.47E-02	6.24E-02	-7.44E-02	4.82E-02	-5.71E-02	4.09E-02
-2.00E-01	-4.94E-02	-2.18E-01	-2.46E-02	-2.18E-01	1.10E-02	-2.02E-01	4.51E-02	-1.71E-01	6.68E-02	-1.37E-01	7.76E-02	-1.06E-01	7.66E-02	-8.67E-02	6.18E-02	-7.79E-02	4.45E-02	-5.98E-02	3.86E-02
-1.90E-01	-3.62E-02	-2.15E-01	-1.53E-02	-2.19E-01	1.49E-02	-2.07E-01	4.46E-02	-1.80E-01	6.47E-02	-1.47E-01	7.19E-02	-1.16E-01	7.02E-02	-9.40E-02	5.22E-02	-7.80E-02	4.35E-02	-5.51E-02	4.44E-02
-1.98E-01	-2.88E-02	-2.12E-01	-9.96E-03	-2.09E-01	1.37E-02	-1.93E-01	3.61E-02	-1.67E-01	5.12E-02	-1.41E-01	6.23E-02	-1.16E-01	6.46E-02	-9.72E-02	5.22E-02	-8.31E-02	4.63E-02	-6.41E-02	4.45E-02
-1.99E-01	-4.39E-02	-2.17E-01	-2.14E-02	-2.15E-01	1.01E-02	-1.99E-01	4.16E-02	-1.69E-01	6.31E-02	-1.35E-01	7.31E-02	-1.05E-01	7.30E-02	-8.58E-02	6.18E-02	-7.64E-02	5.07E-02	-6.14E-02	4.22E-02
-2.00E-01	-4.22E-02	-2.12E-01	-1.94E-02	-2.12E-01	1.10E-02	-1.94E-01	3.95E-02	-1.65E-01	6.05E-02	-1.34E-01	7.08E-02	-1.07E-01	7.12E-02	-9.10E-02	6.01E-02	-8.46E-02	4.52E-02	-7.09E-02	3.68E-02
-2.02E-01	-4.21E-02	-2.12E-01	-1.77E-02	-2.10E-01	1.36E-02	-1.93E-01	4.20E-02	-1.63E-01	6.09E-02	-1.31E-01	7.01E-02	-1.00E-01	6.65E-02	-8.25E-02	6.12E-02	-7.55E-02	5.59E-02	-6.43E-02	5.07E-02
-2.00E-01	-3.89E-02	-2.13E-01	-1.80E-02	-2.12E-01	9.62E-03	-1.93E-01	3.59E-02	-1.66E-01	5.85E-02	-1.37E-01	6.97E-02	-1.07E-01	6.58E-02	-8.72E-02	5.52E-02	-7.66E-02	4.69E-02	-6.20E-02	4.21E-02
-2.01E-01	-2.95E-02	-2.18E-01	-5.74E-03	-2.18E-01	2.44E-02	-2.00E-01	5.13E-02	-1.71E-01	6.79E-02	-1.40E-01	7.39E-02	-1.13E-01	7.11E-02	-9.68E-02	4.97E-02	-8.85E-02	2.87E-02	-6.95E-02	1.86E-02
-2.02E-01	-3.20E-02	-2.20E-01	-9.92E-03	-2.21E-01	1.94E-02	-2.03E-01	4.65E-02	-1.72E-01	6.49E-02	-1.39E-01	7.50E-02	-1.09E-01	7.48E-02	-9.16E-02	5.79E-02	-8.39E-02	3.77E-02	-6.70E-02	2.72E-02
-2.07E-01	-3.25E-02	-2.22E-01	-7.41E-03	-2.18E-01	2.41E-02	-1.97E-01	5.23E-02	-1.64E-01	6.97E-02	-1.30E-01	7.52E-02	-1.01E-01	7.19E-02	-8.67E-02	5.53E-02	-8.37E-02	3.38E-02	-7.14E-02	2.11E-02
-2.12E-01	-4.51E-02	-2.29E-01	-1.45E-02	-2.23E-01	2.34E-02	-1.94E-01	5.62E-02	-1.52E-01	7.55E-02	-1.13E-01	8.52E-02	-8.45E-02	8.24E-02	-7.54E-02	5.83E-02	-8.17E-02	2.87E-02	-7.59E-02	9.88E-03
-2.11E-01	-3.41E-02	-2.27E-01	-5.86E-03	-2.25E-01	2.70E-02	-2.01E-01	5.41E-02	-1.66E-01	7.31E-02	-1.29E-01	7.83E-02	-9.73E-02	7.42E-02	-8.03E-02	5.17E-02	-7.33E-02	2.92E-02	-5.88E-02	1.56E-02
-1.97E-01	-4.12E-02	-2.17E-01	-1.09E-02	-2.19E-01	2.56E-02	-2.02E-01	5.68E-02	-1.68E-01	7.42E-02	-1.33E-01	8.34E-02	-1.01E-01	7.91E-02	-8.30E-02	5.92E-02	-7.69E-02	3.68E-02	-6.19E-02	2.52E-02
-2.06E-01	-4.55E-02	-2.28E-01	-1.50E-02	-2.30E-01	2.28E-02	-2.06E-01	5.49E-02	-1.65E-01	7.29E-02	-1.24E-01	8.28E-02	-9.03E-02	8.04E-02	-7.79E-02	6.17E-02	-8.32E-02	3.36E-02	-7.04E-02	1.72E-02
-2.04E-01	-4.04E-02	-2.21E-01	-9.62E-03	-2.21E-01	2.69E-02	-1.99E-01	5.74E-02	-1.63E-01	7.48E-02	-1.27E-01	8.34E-02	-9.90E-02	7.99E-02	-8.40E-02	5.65E-02	-7.75E-02	3.27E-02	-7.19E-02	1.69E-02
-2.04E-01	-3.93E-02	-2.24E-01	-7.23E-03	-2.24E-01	3.05E-02	-1.99E-01	6.09E-02	-1.61E-01	8.03E-02	-1.24E-01	8.66E-02	-9.30E-02	7.54E-02	-7.94E-02	5.02E-02	-8.28E-02	2.16E-02	-7.69E-02	2.77E-03
-2.00E-01	-4.76E-02	-2.21E-01	-1.46E-02	-2.24E-01	2.60E-02	-2.03E-01	6.04E-02	-1.65E-01	8.04E-02	-1.29E-01	9.15E-02	-9.67E-02	8.04E-02	-7.97E-02	5.70E-02	-7.86E-02	3.12E-02	-6.86E-02	1.32E-02
-2.00E-01	-3.58E-02	-2.17E-01	-6.92E-03	-2.18E-01	2.83E-02	-2.01E-01	5.86E-02	-1.70E-01	7.62E-02	-1.35E-01	8.05E-02	-1.05E-01	7.63E-02	-8.78E-02	5.64E-02	-8.12E-02	3.47E-02	-6.51E-02	2.24E-02
-2.08E-01	-3.05E-02	-2.25E-01	-2.08E-03	-2.25E-01	3.05E-02	-2.05E-01	5.64E-02	-1.70E-01	6.79E-02	-1.31E-01	6.90E-02	-9.77E-02	6.79E-02	-7.99E-02	5.25E-02	-7.50E-02	3.19E-02	-5.70E-02	2.32E-02
-1.98E-01	-3.34E-02	-2.13E-01	-6.23E-03	-2.14E-01	2.47E-02	-1.96E-01	4.98E-02	-1.64E-01	6.35E-02	-1.30E-01	6.90E-02	-1.02E-01	7.07E-02	-8.98E-02	5.85E-02	-8.88E-02	3.74E-02	-7.40E-02	2.76E-02
-1.97E-01	-3.85E-02	-2.11E-01	-8.02E-03	-2.14E-01	2.63E-02	-1.98E-01	5.35E-02	-1.65E-01	6.78E-02	-1.29E-01	7.15E-02	-9.94E-02	7.33E-02	-8.60E-02	6.08E-02	-8.52E-02	3.94E-02	-7.03E-02	2.87E-02
-2.07E-01	-4.16E-02	-2.27E-01	-1.54E-02	-2.25E-01	1.87E-02	-2.00E-01	4.94E-02	-1.61E-01	6.92E-02	-1.22E-01	7.55E-02	-9.04E-02	7.12E-02	-7.78E-02	5.37E-02	-8.04E-02	2.98E-02	-7.39E-02	1.38E-02
-2.00E-01	-3.47E-02	-2.17E-01	-3.31E-03	-2.22E-01	3.11E-02	-2.04E-01	5.76E-02	-1.71E-01	7.00E-02	-1.34E-01	6.91E-02	-1.01E-01	6.62E-02	-8.47E-02	5.61E-02	-8.19E-02	3.53E-02	-7.07E-02	2.39E-02
-2.02E-01	-4.09E-02	-2.17E-01	-1.61E-02	-2.16E-01	1.59E-02	-1.99E-01	4.51E-02	-1.67E-01	6.24E-02	-1.29E-01	6.64E-02	-9.77E-02	6.80E-02	-8.26E-02	5.78E-02	-7.97E-02	3.95E-02	-6.57E-02	3.10E-02
-2.17E-01	-3.64E-02	-2.35E-01	-1.17E-02	-2.27E-01	1.95E-02	-2.03E-01	4.92E-02	-1.63E-01	6.27E-02	-1.20E-01	5.89E-02	-8.48E-02	6.26E-02	-7.25E-02	5.99E-02	-7.64E-02	3.73E-02	-6.74E-02	2.04E-02
-2.09E-01	-2.84E-02	-2.24E-01	3.07E-04	-2.23E-01	3.36E-02	-2.01E-01	5.98E-02	-1.65E-01	7.26E-02	-1.28E-01	7.74E-02	-9.80E-02	7.52E-02	-8.50E-02	5.50E-02	-8.57E-02	2.90E-02	-7.15E-02	1.48E-02
-2.02E-01	-3.22E-02	-2.17E-01	-1.23E-02	-2.14E-01	1.37E-02	-1.97E-01	3.95E-02	-1.69E-01	5.88E-02	-1.38E-01	6.79E-02	-1.09E-01	6.76E-02	-9.00E-02	5.79E-02	-7.98E-02	4.90E-02	-6.34E-02	4.40E-02
-1.91E-01	-2.73E-02	-2.03E-01	-5.32E-03	-2.05E-01	2.19E-02	-1.94E-01	4.60E-02	-1.72E-01	6.15E-02	-1.49E-01	7.10E-02	-1.26E-01	7.15E-02	-1.09E-01	5.45E-02	-9.37E-02	4.23E-02	-7.25E-02	3.64E-02
-1.99E-01	-2.91E-02	-2.12E-01	-7.08E-03	-2.13E-01	1.98E-02	-1.97E-01	4.35E-02	-1.72E-01	5.99E-02	-1.43E-01	6.57E-02	-1.16E-01	6.60E-02	-9.86E-02	5.45E-02	-8.90E-02	4.16E-02	-7.08E-02	3.92E-02
-1.94E-01	-3.14E-02	-2.06E-01	-3.46E-03	-2.10E-01	2.85E-02	-2.01E-01	5.32E-02	-1.75E-01	6.41E-02	-1.45E-01	7.06E-02	-1.18E-01	6.73E-02	-1.00E-01	5.24E-02	-9.02E-02	3.86E-02	-7.36E-02	3.16E-02
-1.98E-01	-3.24E-02	-2.13E-01	-1.01E-02	-2.14E-01	1.82E-02	-2.01E-01	4.33E-02	-1.78E-01	5.90E-02	-1.49E-01	6.30E-02	-1.21E-01	5.93E-02	-1.01E-01	4.88E-02	-8.87E-02	4.13E-02	-7.23E-02	3.64E-02
-1.96E-01	-4.17E-02	-2.08E-01	-1.63E-02	-2.11E-01	1.61E-02	-1.98E-01	4.50E-02	-1.70E-01	6.25E-02	-1.38E-01	7.20E-02	-1.09E-01	7.17E-02	-9.18E-02	5.65E-02	-8.30E-02	4.44E-02	-6.82E-02	3.79E-02
-1.94E-01	-3.56E-02	-2.09E-01	-1.06E-02	-2.13E-01	1.98E-02	-1.98E-01	4.65E-02	-1.72E-01	6.56E-02	-1.45E-01	7.62E-02	-1.22E-01	7.71E-02	-1.05E-01	5.88E-02	-9.06E-02	4.40E-02	-7.24E-02	3.38E-02
-1.92E-01	-3.02E-02	-2.01E-01	-1.02E-02	-1.99E-01	1.51E-02	-1.87E-01	3.91E-02	-1.68E-01	5.65E-02	-1.45E-01	6.46E-02	-1.22E-01	6.66E-02	-1.05E-01	5.44E-02	-9.15E-02	4.75E-02	-7.35E-02	4.20E-02
-1.95E-01	-3.44E-02	-2.10E-01	-7.71E-03	-2.11E-01	2.37E-02	-1.94E-01	4.90E-02	-1.61E-01	6.24E-02	-1.30E-01	7.57E-02	-1.05E-01	7.77E-02	-9.46E-02	6.33E-02	-9.59E-02	4.15E-02	-8.75E-02	2.67E-02
-1.88E-01	-3.25E-02	-2.04E-01	-9.57E-03	-2.06E-01	1.90E-02	-1.98E-01	4.49E-02	-1.77E-01	6.07E-02	-1.51E-01	6.95E-02	-1.26E-01	6.77E-02	-1.05E-01	5.32E-02	-8.97E-02	4.27E-02	-6.86E-02	3.85E-02
-1.94E-01	-3.62E-02	-2.11E-01	-1.23E-02	-2.16E-01	1.57E-02	-2.04E-01	3.94E-02	-1.78E-01	5.36E-02	-1.49E-01	5.95E-02	-1.22E-01	5.98E-02	-1.03E-01	4.76E-02	-9.15E-02	3.78E-02	-7.21E-02	3.75E-02
-1.95E-01	-2.75E-02	-2.11E-01	-4.64E-03	-2.16E-01	2.31E-02	-2.05E-01	4.73E-02	-1.84E-01	6.31E-02	-1.57E-01	6.68E-02	-1.29E-01	6.39E-02	-1.07E-01	5.11E-02	-9.15E-02	4.24E-02	-7.15E-02	3.86E-02
-1.98E-01	-3.27E-02	-2.16E-01	-7.90E-03	-2.19E-01	2.21E-02	-2.05E-01	4.80E-02	-1.76E-01	6.33E-02	-1.43E-01	7.02E-02	-1.13E-01	6.81E-02	-9.30E-02	5.65E-02	-8.21E-02	4.57E-02	-6.56E-02	3.89E-02
-2.04E-01	-3.35E-02	-2.23E-01	-1.19E-02	-2.24E-01	1.77E-02	-2.07E-01	4.56E-02	-1.78E-01	6.43E-02	-1.47E-01	7.27E-02	-1.16E-01	6.75E-02	-9.56E-02	5.19E-02	-8.32E-02	3.96E-02	-6.70E-02	3.10E-02
-1.96E-01	-3.56E-02	-2.15E-01	-1.04E-02	-2.19E-01	2.27E-02	-2.05E-01	5.25E-02	-1.77E-01	7.15E-02	-1.48E-01	8.00E-02	-1.21E-01	7.58E-02	-1.05E-01	5.29E-02	-9.31E-02	3.28E-02	-7.59E-02	1.99E-02
-1.94E-01	-2.75E-02	-2.06E-01	-7.36E-03	-2.06E-01	1.77E-02	-1.94E-01	4.01E-02	-1.73E-01	5.44E-02	-1.49E-01	6.24E-02	-1.25E-01	6.27E-02	-1.07E-01	5.32E-02	-9.48E-02	4.68E-02	-7.70E-02	4.40E-02
-2.05E-01	-3.76E-02	-2.28E-01	-1.55E-02	-2.29E-01	1.74E-02	-2.11E-01	4.95E-02	-1.77E-01	7.02E-02	-1.40E-01	7.62E-02	-1.08E-01	6.98E-02	-8.92E-02	5.40E-02	-8.20E-02	4.00E-02	-7.25E-02	2.74E-02
-2.00E-01	-3.18E-02	-2.12E-01	-6.28E-03	-2.12E-01	2.45E-02	-1.98E-01	5.06E-02	-1.74E-01	6.55E-02	-1.44E-01	6.35E-02	-1.16E-01	6.42E-02	-9.72E-02	5.22E-02	-8.78E-02	3.52E-02	-6.99E-02	3.02E-02
-1.99E-01	-3.22E-02	-2.18E-01	-6.21E-03	-2.22E-01	2.52E-02	-2.09E-01	5.16E-02	-1.84E-01	6.63E-02	-1.51E-01	6.60E-02	-1.20E-01	5.99E-02	-9.87E-02	5.37E-02	-8.76E-02	4.28E-02	-7.18E-02	3.50E-02
-1.93E-01	-3.35E-02	-2.12E-01	-1.02E-02	-2.15E-01	2.10E-02	-2.08E-01	4.79E-02	-1.83E-01	6.21E-02	-1.54E-01	6.84E-02	-1.26E-01	6.58E-02	-1.04E-01	5.17E-02	-8.76E-02	4.15E-02	-6.63E-02	3.69E-02

-1.97E-01	-4.84E-02	-2.21E-01	-2.33E-02	-2.26E-01	1.26E-02	-2.12E-01	4.74E-02	-1.82E-01	7.18E-02	-1.48E-01	8.41E-02	-1.16E-01	8.07E-02	-9.40E-02	6.16E-02	-8.02E-02	4.52E-02	-6.14E-02	3.53E-02
-1.96E-01	-3.12E-02	-2.05E-01	-7.47E-03	-2.04E-01	2.04E-02	-1.89E-01	4.43E-02	-1.66E-01	5.98E-02	-1.37E-01	6.33E-02	-1.11E-01	6.49E-02	-9.89E-02	6.19E-02	-9.44E-02	5.22E-02	-8.17E-02	4.80E-02
-1.95E-01	-3.67E-02	-2.16E-01	-1.18E-02	-2.22E-01	1.94E-02	-2.10E-01	4.69E-02	-1.83E-01	6.42E-02	-1.53E-01	7.15E-02	-1.23E-01	6.73E-02	-1.00E-01	5.30E-02	-8.48E-02	4.10E-02	-6.45E-02	3.54E-02
-1.97E-01	-3.75E-02	-2.15E-01	-1.23E-02	-2.20E-01	1.92E-02	-2.08E-01	4.65E-02	-1.80E-01	6.25E-02	-1.49E-01	6.90E-02	-1.21E-01	6.68E-02	-1.01E-01	5.23E-02	-8.89E-02	3.91E-02	-7.01E-02	3.49E-02
-2.00E-01	-2.86E-02	-2.15E-01	-6.94E-03	-2.19E-01	1.92E-02	-2.03E-01	4.17E-02	-1.77E-01	5.93E-02	-1.48E-01	6.87E-02	-1.20E-01	6.72E-02	-1.00E-01	5.01E-02	-8.40E-02	3.94E-02	-6.24E-02	3.46E-02
-1.98E-01	-3.32E-02	-2.14E-01	-6.87E-03	-2.19E-01	2.43E-02	-2.04E-01	5.10E-02	-1.77E-01	6.84E-02	-1.46E-01	7.37E-02	-1.17E-01	6.89E-02	-9.57E-02	5.45E-02	-8.35E-02	4.07E-02	-6.59E-02	3.47E-02
-1.89E-01	-4.45E-02	-2.12E-01	-1.90E-02	-2.19E-01	1.65E-02	-2.13E-01	4.84E-02	-1.89E-01	6.82E-02	-1.58E-01	7.59E-02	-1.27E-01	7.45E-02	-1.05E-01	6.21E-02	-9.20E-02	5.05E-02	-7.29E-02	4.51E-02
-1.98E-01	-3.57E-02	-2.16E-01	-1.13E-02	-2.22E-01	1.90E-02	-2.06E-01	4.61E-02	-1.79E-01	6.60E-02	-1.50E-01	7.88E-02	-1.22E-01	7.75E-02	-1.01E-01	5.93E-02	-8.56E-02	4.40E-02	-6.37E-02	3.75E-02
-1.98E-01	-3.49E-02	-2.16E-01	-1.45E-02	-2.18E-01	1.26E-02	-2.01E-01	3.89E-02	-1.78E-01	6.24E-02	-1.50E-01	7.37E-02	-1.21E-01	7.11E-02	-1.01E-01	5.98E-02	-9.03E-02	4.68E-02	-7.20E-02	3.99E-02
-2.05E-01	-3.42E-02	-2.22E-01	-1.08E-02	-2.23E-01	2.05E-02	-2.04E-01	4.94E-02	-1.74E-01	6.91E-02	-1.40E-01	7.50E-02	-1.08E-01	6.84E-02	-8.72E-02	5.19E-02	-7.74E-02	3.49E-02	-5.90E-02	2.79E-02
-2.02E-01	-3.84E-02	-2.22E-01	-1.19E-02	-2.23E-01	2.15E-02	-2.03E-01	5.13E-02	-1.70E-01	7.05E-02	-1.34E-01	7.83E-02	-1.05E-01	7.61E-02	-8.99E-02	5.92E-02	-8.63E-02	3.92E-02	-7.79E-02	2.34E-02
-1.96E-01	-3.07E-02	-2.17E-01	-1.02E-02	-2.19E-01	1.83E-02	-2.06E-01	4.58E-02	-1.79E-01	6.37E-02	-1.48E-01	7.35E-02	-1.19E-01	7.29E-02	-9.89E-02	5.49E-02	-8.42E-02	4.21E-02	-6.42E-02	3.37E-02
-2.01E-01	-3.05E-02	-2.25E-01	-5.82E-03	-2.31E-01	2.48E-02	-2.16E-01	5.14E-02	-1.85E-01	6.71E-02	-1.48E-01	7.11E-02	-1.14E-01	6.73E-02	-9.11E-02	5.39E-02	-7.85E-02	4.23E-02	-6.08E-02	3.34E-02
-2.03E-01	-4.02E-02	-2.22E-01	-1.55E-02	-2.24E-01	1.67E-02	-2.06E-01	4.65E-02	-1.75E-01	6.73E-02	-1.40E-01	7.71E-02	-1.09E-01	7.60E-02	-8.97E-02	6.25E-02	-8.15E-02	4.45E-02	-6.29E-02	3.83E-02
-2.00E-01	-2.50E-02	-2.11E-01	-3.15E-03	-2.10E-01	2.32E-02	-1.92E-01	4.49E-02	-1.66E-01	6.02E-02	-1.36E-01	6.58E-02	-1.09E-01	5.98E-02	-9.15E-02	4.57E-02	-8.48E-02	2.95E-02	-6.98E-02	2.23E-02
-1.90E-01	-2.93E-02	-2.07E-01	-7.41E-03	-2.13E-01	1.88E-02	-2.01E-01	4.15E-02	-1.77E-01	5.73E-02	-1.48E-01	6.54E-02	-1.21E-01	6.72E-02	-1.03E-01	5.11E-02	-8.69E-02	4.49E-02	-6.65E-02	3.88E-02
-1.99E-01	-4.70E-02	-2.10E-01	-1.87E-02	-2.08E-01	1.67E-02	-1.90E-01	4.92E-02	-1.62E-01	7.36E-02	-1.30E-01	8.76E-02	-1.03E-01	9.07E-02	-8.84E-02	7.31E-02	-8.39E-02	4.96E-02	-7.08E-02	3.99E-02
-1.92E-01	-3.07E-02	-2.03E-01	-5.54E-03	-2.04E-01	2.52E-02	-1.93E-01	5.17E-02	-1.71E-01	6.77E-02	-1.47E-01	7.46E-02	-1.23E-01	7.19E-02	-1.08E-01	5.48E-02	-9.82E-02	3.71E-02	-8.17E-02	2.76E-02
-1.87E-01	-2.91E-02	-1.96E-01	-8.81E-03	-1.97E-01	1.63E-02	-1.85E-01	3.81E-02	-1.64E-01	5.48E-02	-1.42E-01	6.84E-02	-1.20E-01	7.08E-02	-1.06E-01	5.52E-02	-9.44E-02	3.98E-02	-7.38E-02	3.52E-02
-2.08E-01	-3.40E-02	-2.19E-01	-6.20E-03	-2.12E-01	2.71E-02	-1.95E-01	5.98E-02	-1.64E-01	7.40E-02	-1.29E-01	7.96E-02	-9.95E-02	7.87E-02	-8.84E-02	5.34E-02	-8.03E-02	2.96E-02	-6.01E-02	1.94E-02
-1.99E-01	-2.84E-02	-2.11E-01	-6.63E-03	-2.08E-01	2.00E-02	-1.88E-01	4.47E-02	-1.63E-01	6.67E-02	-1.36E-01	8.00E-02	-1.12E-01	8.08E-02	-9.73E-02	6.73E-02	-9.27E-02	4.76E-02	-8.11E-02	3.58E-02
-2.01E-01	-3.26E-02	-2.15E-01	-8.95E-03	-2.15E-01	2.02E-02	-1.97E-01	4.57E-02	-1.68E-01	6.36E-02	-1.36E-01	7.45E-02	-1.08E-01	7.63E-02	-9.32E-02	5.82E-02	-8.57E-02	3.89E-02	-6.69E-02	3.12E-02
-2.03E-01	-2.80E-02	-2.17E-01	-4.99E-03	-2.16E-01	2.29E-02	-1.99E-01	4.71E-02	-1.71E-01	6.27E-02	-1.41E-01	6.91E-02	-1.14E-01	6.30E-02	-9.32E-02	4.16E-02	-7.63E-02	2.59E-02	-5.61E-02	1.72E-02
-1.96E-01	-2.69E-02	-2.11E-01	-4.80E-03	-2.13E-01	2.28E-02	-1.98E-01	4.76E-02	-1.77E-01	6.67E-02	-1.54E-01	7.72E-02	-1.30E-01	6.76E-02	-1.09E-01	4.81E-02	-9.12E-02	3.18E-02	-6.74E-02	2.63E-02
-1.74E-01	-1.52E-02	-1.84E-01	1.37E-03	-1.85E-01	2.08E-02	-1.81E-01	3.90E-02	-1.68E-01	5.03E-02	-1.51E-01	5.66E-02	-1.31E-01	5.58E-02	-1.16E-01	4.80E-02	-1.05E-01	4.45E-02	-8.56E-02	4.16E-02
-2.02E-01	-2.71E-02	-2.14E-01	-5.20E-03	-2.15E-01	2.18E-02	-2.01E-01	4.53E-02	-1.75E-01	6.06E-02	-1.46E-01	6.75E-02	-1.17E-01	6.32E-02	-9.70E-02	4.69E-02	-8.35E-02	3.37E-02	-6.39E-02	2.59E-02
-1.98E-01	-2.58E-02	-2.06E-01	-2.91E-03	-2.02E-01	2.51E-02	-1.86E-01	4.94E-02	-1.62E-01	6.43E-02	-1.35E-01	6.90E-02	-1.12E-01	6.60E-02	-1.01E-01	4.82E-02	-9.94E-02	2.77E-02	-8.50E-02	1.83E-02
-1.97E-01	-3.02E-02	-2.11E-01	-3.55E-03	-2.11E-01	2.64E-02	-1.94E-01	5.08E-02	-1.66E-01	6.45E-02	-1.33E-01	6.42E-02	-1.05E-01	6.74E-02	-9.04E-02	5.07E-02	-8.49E-02	3.11E-02	-6.90E-02	2.31E-02
-1.93E-01	-2.48E-02	-2.05E-01	-1.47E-03	-2.04E-01	2.50E-02	-1.89E-01	4.55E-02	-1.63E-01	5.35E-02	-1.35E-01	5.45E-02	-1.16E-01	6.09E-02	-1.08E-01	3.99E-02	-9.77E-02	1.99E-02	-7.75E-02	1.26E-02
-1.96E-01	-2.88E-02	-2.13E-01	-1.10E-03	-2.15E-01	3.06E-02	-2.03E-01	5.66E-02	-1.76E-01	7.00E-02	-1.45E-01	7.30E-02	-1.15E-01	7.05E-02	-9.53E-02	5.48E-02	-8.34E-02	4.01E-02	-6.69E-02	3.22E-02
-1.92E-01	-3.55E-02	-2.08E-01	-1.08E-02	-2.12E-01	1.82E-02	-1.97E-01	4.33E-02	-1.72E-01	6.30E-02	-1.42E-01	7.24E-02	-1.15E-01	7.69E-02	-9.80E-02	6.56E-02	-9.02E-02	4.96E-02	-7.33E-02	4.18E-02
-1.94E-01	-1.44E-02	-2.08E-01	5.34E-03	-2.10E-01	2.80E-02	-1.98E-01	4.68E-02	-1.77E-01	6.10E-02	-1.52E-01	6.62E-02	-1.26E-01	6.02E-02	-1.05E-01	4.75E-02	-8.88E-02	3.80E-02	-6.85E-02	3.17E-02
-1.98E-01	-2.73E-02	-2.15E-01	-3.47E-03	-2.21E-01	2.55E-02	-2.08E-01	5.01E-02	-1.83E-01	6.68E-02	-1.53E-01	7.52E-02	-1.23E-01	7.28E-02	-1.02E-01	5.36E-02	-8.30E-02	4.19E-02	-6.01E-02	3.48E-02
-1.94E-01	-3.57E-02	-2.13E-01	-1.20E-02	-2.16E-01	1.64E-02	-2.01E-01	4.25E-02	-1.75E-01	6.22E-02	-1.43E-01	7.43E-02	-1.15E-01	8.05E-02	-9.61E-02	6.66E-02	-8.43E-02	5.27E-02	-6.81E-02	4.54E-02
-2.04E-01	-2.54E-02	-2.17E-01	-5.17E-03	-2.15E-01	2.09E-02	-1.99E-01	4.56E-02	-1.71E-01	6.11E-02	-1.40E-01	7.26E-02	-1.12E-01	7.37E-02	-9.53E-02	5.76E-02	-8.64E-02	4.14E-02	-7.03E-02	3.19E-02
-1.96E-01	-3.38E-02	-2.16E-01	-1.06E-02	-2.20E-01	1.76E-02	-2.04E-01	4.24E-02	-1.75E-01	6.13E-02	-1.43E-01	7.31E-02	-1.13E-01	7.55E-02	-9.53E-02	6.26E-02	-8.63E-02	4.76E-02	-6.73E-02	4.20E-02
-1.89E-01	-2.19E-02	-1.98E-01	-1.93E-03	-1.98E-01	2.27E-02	-1.86E-01	4.48E-02	-1.67E-01	6.16E-02	-1.46E-01	7.05E-02	-1.24E-01	6.77E-02	-1.06E-01	5.36E-02	-9.22E-02	4.14E-02	-7.24E-02	3.53E-02
-1.89E-01	-2.81E-02	-2.02E-01	-6.95E-03	-2.05E-01	1.92E-02	-1.95E-01	4.22E-02	-1.76E-01	5.66E-02	-1.53E-01	6.43E-02	-1.32E-01	6.77E-02	-1.18E-01	5.00E-02	-9.99E-02	3.91E-02	-7.62E-02	3.49E-02
-2.02E-01	-3.18E-02	-2.21E-01	-7.34E-03	-2.26E-01	2.02E-02	-2.10E-01	4.19E-02	-1.78E-01	5.39E-02	-1.43E-01	6.50E-02	-1.13E-01	7.02E-02	-9.30E-02	5.85E-02	-8.19E-02	4.62E-02	-6.35E-02	4.17E-02
-2.04E-01	-4.11E-02	-2.17E-01	-1.58E-02	-2.17E-01	1.63E-02	-1.98E-01	4.52E-02	-1.70E-01	6.65E-02	-1.39E-01	7.77E-02	-1.10E-01	7.54E-02	-9.15E-02	5.87E-02	-8.13E-02	4.19E-02	-6.96E-02	3.04E-02
-2.00E-01	-3.11E-02	-2.15E-01	-8.57E-03	-2.17E-01	1.94E-02	-2.01E-01	4.43E-02	-1.76E-01	6.33E-02	-1.48E-01	7.54E-02	-1.22E-01	7.49E-02	-1.03E-01	5.97E-02	-8.94E-02	4.61E-02	-7.35E-02	3.48E-02
-1.90E-01	-3.28E-02	-2.08E-01	-8.25E-03	-2.13E-01	2.15E-02	-2.01E-01	4.79E-02	-1.78E-01	6.69E-02	-1.51E-01	7.84E-02	-1.22E-01	7.43E-02	-9.96E-02	6.09E-02	-8.40E-02	4.98E-02	-6.40E-02	4.21E-02
-1.97E-01	-3.67E-02	-2.10E-01	-1.48E-02	-2.10E-01	1.39E-02	-1.93E-01	4.05E-02	-1.68E-01	6.07E-02	-1.42E-01	7.40E-02	-1.17E-01	7.42E-02	-1.01E-01	5.82E-02	-9.27E-02	4.18E-02	-7.45E-02	3.67E-02
-1.91E-01	-1.57E-02	-2.04E-01	1.44E-03	-2.02E-01	2.23E-02	-1.87E-01	4.21E-02	-1.66E-01	5.68E-02	-1.42E-01	6.34E-02	-1.20E-01	6.12E-02	-1.05E-01	4.99E-02	-9.66E-02	3.81E-02	-8.50E-02	2.71E-02
-1.86E-01	-2.69E-02	-2.03E-01	-3.97E-03	-2.06E-01	2.43E-02	-1.96E-01	4.93E-02	-1.74E-01	6.52E-02	-1.46E-01	7.22E-02	-1.22E-01	7.42E-02	-1.07E-01	6.51E-02	-1.02E-01	4.87E-02	-9.05E-02	3.92E-02
-1.98E-01	-2.36E-02	-2.13E-01	-3.83E-03	-2.14E-01	2.03E-02	-1.97E-01	4.17E-02	-1.71E-01	5.77E-02	-1.43E-01	6.78E-02	-1.18E-01	6.94E-02	-1.00E-01	5.42E-02	-8.89E-02	3.82E-02	-7.60E-02	2.66E-02
-1.91E-01	-4.41E-02	-2.11E-01	-1.51E-02	-2.19E-01	1.95E-02	-2.06E-01	4.91E-02	-1.77E-01	6.81E-02	-1.43E-01	7.76E-02	-1.12E-01	7.71E-02	-9.27E-02	6.04E-02	-8.27E-02	4.44E-02	-6.94E-02	3.29E-02
-1.96E-01	-2.26E-02	-2.08E-01	-3.50E-03	-2.05E-01	2.10E-02	-1.95E-01	4.64E-02	-1.75E-01	6.14E-02	-1.51E-01	6.76E-02	-1.27E-01	5.64E-02	-1.06E-01	4.05E-02	-8.95E-02	3.29E-02	-6.62E-02	3.08E-02
-2.04E-01	-2.31E-02	-2.28E-01	-1.13E-03	-2.28E-01	2.44E-02	-2.05E-01	4.69E-02	-1.79E-01	7.07E-02	-1.50E-01	7.55E-02	-1.21E-01	5.84E-02	-9.43E-02	4.76E-02	-8.52E-02	2.74E-02	-6.44E-02	2.01E-02
-1.95E-01	-2.74E-02	-2.11E-01	-4.97E-03	-2.14E-01	2.05E-02	-1.97E-01	4.13E-02	-1.70E-01	5.70E-02	-1.39E-01	6.40E-02	-1.11E-01	6.47E-02	-9.40E-02	5.31E-02	-8.75E-02	3.71E-02	-7.00E-02	3.22E-02
-1.97E-01	-3.49E-02	-2.02E-01	-1.58E-02	-2.01E-01	1.03E-02	-1.86E-01	3.44E-02	-1.65E-01	5.38E-02	-1.42E-01	6.61E-02	-1.19E-01	6.67E-02	-1.02E-01	5.27E-02	-8.98E-02	4.18E-02	-7.16E-02	3.60E-02
-1.96E-01	-3.28E-02	-2.12E-01	-1.27E-02	-2.12E-01	1.32E-02	-1.97E-01	3.83E-02	-1.72E-01	5.72E-02	-1.44E-01	6.89E-02	-1.17E-01	6.79E-02	-9.88E-02	5.54E-02	-8.86E-02	4.49E-02	-7.26E-02	3.89E-02
-1.88E-01	-1.47E-02	-1.96E-01	4.80E-03	-1.93E-01	2.81E-02	-1.83E-01	4.85E-02	-1.64E-01	5.88E-02	-1.44E-01	6.43E-02	-1.25E-01	6.53E-02	-1.14E-01	4.87E-02	-1.02E-01	3.26E-02	-8.34E-02	2.47E-02
-1.91E-01	-2.29E-02	-2.04E-01	-3.13E-03	-2.08E-01	2.03E-02	-1.95E-01	3.95E-02	-1.73E-01	5.60E-02	-1.45E-01	6.36E-02	-1.17E-01	5.99E-02	-9.60E-02	4.76E-02	-8.18E-02	3.96E-02	-6.21E-02	3.42E-02
-1.92E-01	-3.26E-02	-2.04E-01	-6.26E-03	-2.07E-01	2.46E-02	-1.96E-01	5.00E-02	-1.71E-01	6.27E-02	-1.42E-01	7.07E-02	-1.15E-01	7.19E-02	-9.91E-02	5.32E-02	-8.72E-02	3.67E-02	-6.95E-02	2.54E-02
-1.90E-01	-2.25E-02	-1.98E-01	-4.33E-03	-1.99E-01	1.87E-02	-1.88E-01	3.90E-02	-1.68E-01	5.37E-02	-1.44E-01	6.26E-02	-1.23E-01	6.66E-02	-1.09E-01	5.08E-02	-9.29E-02	3.98E-02	-7.06E-02	3.46E-02
-2.03E-01	-2.20E-02	-2.20E-01	1.33E-03	-2.20E-01	2.79E-02	-2.00E-01	4.94E-02	-1.68E-01	6.42E-02	-1.36E-01	7.06E-02	-1.08E-01	6.73E-02	-9.07E-02	5.47E-02	-8.37E-02	3.89E-02	-7.47E-02	2.72E-02
-1.96E-01	-2.24E-02	-2.12E-01	-7.08E-04	-2.14E-01	2.36E-02	-1.98E-01	4.37E-02	-1.72E-01	5.80E-02	-1.43E-01	6.72E-02	-1.14E-01	6.06E-02	-9.36E-02	4.71E-02	-8.30E-02	3.35E-02	-6.03E-02	3.10E-02
-1.91E-01	-3.90E-02	-2.04E-01	-9.29E-03	-2.08E-01	2.57E-02	-2.00E-01	5.52E-02	-1.80E-01	7.30E-02	-1.53E-01	7.97E-02	-1.26E-01	7.56E-02	-1.07E-01	5.60E-02	-9.06E-02	4.10E-02	-6.90E-02	3.15E-02
-1.91E-01	-4.35E-02	-2.06E-01	-1.94E-02	-2.05E-01	1.23E-02	-1.87E-01	4.28E-02	-1.60E-01	6.59E-02	-1.34E-01	8.11E-02	-1.09E-01	8.11E-02	-9.54E-02	6.39E-02	-9.00E-02	4.41E-02	-8.28E-02	3.05E-02
-1.98E-01	-2.61E-02	-2.19E-01	3.66E-04	-2.25E-01	2.94E-02	-2.06E-01	5.17E-02	-1.74E-01	6.55E-02	-1.38E-01	6.58E-02	-1.06E-01	6.59E-02	-9.03E-02	5.48E-02	-8.89E-02	3.42E-02	-7.57E-02	2.08E-02
-2.10E-01	-3.57E-02	-2.21E-01	-8.04E-03	-2.21E-01	2.56E-02	-1.95E-01	4.96E-02	-1.56E-01	6.49E-02	-1.19E-01	7.30E-02	-8.93E-02	7.07E-02	-8.12E-02	5.03E-02	-9.04E-02	2.47E-02	-7.97E-02	8.78E-03
-1.96E-01	-2.93E-02	-2.03E-01	-8.86E-03	-2.01E-01	1.71E-02	-1.89E-01	4.05E-02	-1.66E-01	5.47E-02	-1.42E-01	6.48E-02	-1.17E-01	6.48E-02	-1.02E-01	5.22E-02	-9.22E-02	3.84E-02	-7.59E-02	3.12E-02
-2.01E-01	-2.78E-02	-2.14E-01	-4.13E-03	-2.14E-01	2.38E-02	-1.93E-01	4.60E-02	-1.63E-01	6.07E-02	-1.33E-01	6.97E-02	-1.08E-01	6.86E-02	-9.87E-02	5.05E-02	-1.01E-01	2.74E-02	-8.93E-02	1.43E-02
-2.00E-01	-3.14E-02	-2.17E-01	-5.53E-03	-2.21E-01	2.41E-02	-2.03E-01	4.80E-02	-1.73E-01	6.42E-02	-1.39E-01	7.10E-02	-1.08E-01	6.96E-02	-8.84E-02	5.04E-02	-7.53E-02	3.36E-02	-5.35E-02	2.80E-02
-1.91E-01	-3.49E-02	-2.07E-01	-1.01E-02	-2.12E-01	1.87E-02	-1.97E-01	4.28E-02	-1.68E-01	5.92E-02	-1.38E-01	7.05E-02	-1.12E-01	7.14E-02	-9.92E-02	5.67E-02	-9.80E-02	3.72E-02	-8.15E-02	2.78E-02
-2.05E-01	-3.76E-02	-2.21E-01	-9.88E-03	-2.21E-01	2.37E-02	-1.97E-01	5.17E-02	-1.62E-01	6.93E-02	-1.25E-01	7.40E-02	-9.60E-02	6.71E-02	-8.30E-02	5.30E-02	-8.51E-02	3.31E-02	-8.37E-02	1.67E-02
-1.97E-01	-3.64E-02	-2.17E-01	-1.19E-02	-2.22E-01	1.81E-02	-2.04E-01	4.45E-02	-1.75E-01	6.39E-02	-1.43E-01	7.24E-02	-1.13E-01	7.13E-02	-9.58E-02	5.46E-02	-8.81E-02	3.51E-02	-6.96E-02	2.48E-02
-2.00E-01	-3.75E-02	-2.16E-01	-1.39E-02	-2.15E-01	1.55E-02	-1.96E-01	4.19E-02	-1.65E-01	6.06E-02	-1.32E-01	7.03E-02	-1.04E-01	7.06E-02	-9.05E-02	5.64E-02	-8.78E-02	3.79E-02	-7.55E-02	2.71E-02
-2.00E-01	-2.92E-02	-2.14E-01	-5.26E-03	-2.13E-01	2.33E-02	-1.94E-01	4.75E-02	-1.64E-01	6.50E-02	-1.34E-01	7.29E-02	-1.07E-01	6.94E-02	-9.14E-02	4.90E-02	-8.46E-02	2.75E-02	-7.27E-02	1.44E-02
-2.06E-01	-2.83E-02	-2.23E-01	-5.59E-03	-2.22E-01	2.17E-02	-1.99E-01	4.48E-02	-1.68E-01	6.42E-02	-1.35E-01	6.97E-02	-1.08E-01	6.70E-02	-9.40E-02	4.80E-02	-9.19E-02	2.42E-02	-7.43E-02	1.49E-02
-2.03E-01	-3.33E-02	-2.21E-01	-9.16E-03	-2.21E-01	2.16E-02	-2.00E-01	4.86E-02	-1.70E-01	6.90E-02	-1.40E-01	8.05E-02	-1.13E-01	7.43E-02	-9.70E-02	5.12E-02	-9.24E-02	2.61E-02	-7.36E-02	1.59E-02
-2.06E-01	-3.26E-02	-2.20E-01	-8.02E-03	-2.19E-01	2.37E-02	-1.97E-01	5.19E-02	-1.67E-01	7.14E-02	-1.36E-01	8.14E-02	-1.10E-01	7.45E-02	-9.38E-02	5.13E-02	-8.86E-02	2.59E-02	-7.28E-02	1.30E-02
-2.10E-01	-4.45E-02	-2.24E-01	-1.23E-02	-2.18E-01	2.59E-02	-1.90E-01	5.74E-02	-1.49E-01	7.54E-02	-1.10E-01	8.32E-02	-8.17E-02	7.68E-02	-7.61E-02	5.67E-02	-9.09E-02	3.01E-02	-9.00E-02	1.30E-02
-2.09E-01	-3.89E-02	-2.31E-01	-9.22E-03	-2.30E-01	2.74E-02	-2.03E-01	5.84E-02	-1.61E-01	7.53E-02	-1.18E-01	7.98E-02	-8.66E-02	7.37E-02	-7.87E-02	6.05E-02	-9.26E-02	3.68E-02	-9.63E-02	1.93E-02
-2.05E-01	-2.91E-02	-2.24E-01	-2.97E-04	-2.23E-01	3.23E-02	-1.97E-01	5.72E-02	-1.60E-01	7.43E-02	-1.25E-01	8.20E-02	-9.46E-02	6.96E-02	-7.92E-02	4.95E-02	-8.64E-02	2.40E-02	-7.09E-02	9.20E-03
-2.06E-01	-2.81E-02	-2.26E-01	2.29E-03	-2.23E-01	3.62E-02	-1.97E-01	6.33E-02	-1.60E-01	7.81E-02	-1.21E-01	7.74E-02	-9.09E-02	7.15E-02	-8.26E-02	4.87E-02	-9.14E-02	2.33E-02	-9.01E-02	5.82E-03
-2.03E-01	-4.36E-02	-2.22E-01	-1.55E-02	-2.23E-01	2.16E-02	-2.04E-01	5.47E-02	-1.69E-01	7.41E-02	-1.32E-01	8.12E-02	-9.83E-02	7.13E-02	-8.19E-02	5.45E-02	-8.52E-02	3.11E-02	-7.17E-02	1.43E-02
-1.99E-01	-4.84E-02	-2.25E-01	-1.72E-02	-2.31E-01	2.19E-02	-2.10E-01	5.47E-02	-1.69E-01	7.14E-02	-1.29E-01	8.49E-02	-9.78E-02	8.56E-02	-8.32E-02	5.92E-02	-8.10E-02	3.09E-02	-6.91E-02	1.29E-02
-2.09E-01	-2.54E-02	-2.27E-01	4.37E-04	-2.26E-01	2.92E-02	-1.99E-01	5.08E-02	-1.60E-01	6.58E-02	-1.24E-01	7.20E-02	-9.53E-02	6.69E-02	-8.57E-02	4.70E-02	-9.00E-02	2.36E-02	-9.05E-02	6.77E-03
-2.07E-01	-3.32E-02	-2.27E-01	-7.44E-03	-2.27E-01	2.44E-02	-2.03E-01	5.18E-02	-1.67E-01	7.03E-02	-1.30E-01	7.76E-02	-9.96E-02	7.31E-02	-8.66E-02	5.57E-02	-9.05E-02	3.09E-02	-7.59E-02	1.72E-02
-2.02E-01	-3.33E-02	-2.23E-01	-5.33E-03	-2.27E-01	2.70E-02	-2.07E-01	5.39E-02	-1.74E-01	7.26E-02	-1.40E-01	8.01E-02	-1.11E-01	7.43E-02	-9.38E-02	5.50E-02	-8.76E-02	3.34E-02	-8.14E-02	1.71E-02
-2.08E-01	-4.47E-02	-2.27E-01	-1.64E-02	-2.24E-01	2.08E-02	-1.98E-01	5.49E-02	-1.60E-01	7.82E-02	-1.24E-01	8.90E-02	-9.53E-02	8.51E-02	-8.57E-02	6.46E-02	-9.03E-02	3.77E-02	-8.86E-02	1.89E-02

-2.04E-01	-3.35E-02	-2.19E-01	-5.84E-03	-2.18E-01	2.85E-02	-2.02E-01	5.83E-02	-1.71E-01	7.30E-02	-1.38E-01	8.03E-02	-1.09E-01	7.48E-02	-9.36E-02	5.13E-02	-8.72E-02	2.70E-02	-7.25E-02	1.39E-02
-1.98E-01	-4.75E-02	-2.18E-01	-1.52E-02	-2.21E-01	2.42E-02	-2.00E-01	5.88E-02	-1.68E-01	8.41E-02	-1.35E-01	9.77E-02	-1.07E-01	9.61E-02	-9.22E-02	6.67E-02	-8.10E-02	3.86E-02	-6.79E-02	2.13E-02
-2.05E-01	-3.81E-02	-2.28E-01	-9.15E-03	-2.29E-01	2.69E-02	-2.04E-01	5.78E-02	-1.64E-01	7.55E-02	-1.25E-01	8.34E-02	-9.57E-02	7.64E-02	-8.31E-02	5.48E-02	-8.32E-02	3.74E-02	-9.68E-02	2.42E-02
-2.09E-01	-3.37E-02	-2.33E-01	-7.84E-03	-2.32E-01	2.46E-02	-2.07E-01	5.41E-02	-1.69E-01	7.40E-02	-1.31E-01	8.12E-02	-1.00E-01	7.53E-02	-8.74E-02	5.48E-02	-9.08E-02	3.00E-02	-8.71E-02	1.16E-02
-2.21E-01	-3.56E-02	-2.36E-01	-1.11E-03	-2.26E-01	3.73E-02	-1.90E-01	6.60E-02	-1.42E-01	8.05E-02	-9.71E-02	8.18E-02	-6.74E-02	7.05E-02	-6.60E-02	4.81E-02	-8.95E-02	2.26E-02	-1.04E-01	9.56E-03
-2.22E-01	-4.07E-02	-2.34E-01	-8.66E-03	-2.27E-01	3.05E-02	-1.95E-01	6.14E-02	-1.49E-01	7.83E-02	-1.06E-01	8.57E-02	-7.56E-02	7.61E-02	-6.90E-02	5.38E-02	-8.36E-02	2.66E-02	-9.46E-02	1.19E-02
-2.13E-01	-3.64E-02	-2.31E-01	-1.10E-02	-2.28E-01	2.07E-02	-1.98E-01	4.70E-02	-1.58E-01	6.69E-02	-1.22E-01	8.23E-02	-9.40E-02	8.08E-02	-8.12E-02	5.76E-02	-7.97E-02	3.40E-02	-8.52E-02	1.74E-02
-2.03E-01	-3.70E-02	-2.25E-01	-7.57E-03	-2.27E-01	2.68E-02	-2.04E-01	5.52E-02	-1.66E-01	7.29E-02	-1.30E-01	8.42E-02	-1.00E-01	8.04E-02	-8.74E-02	6.27E-02	-9.04E-02	3.58E-02	-8.28E-02	1.80E-02
-2.06E-01	-2.57E-02	-2.27E-01	8.28E-03	-2.27E-01	4.42E-02	-2.01E-01	6.95E-02	-1.61E-01	8.11E-02	-1.21E-01	8.25E-02	-9.07E-02	7.32E-02	-7.96E-02	5.17E-02	-8.75E-02	2.45E-02	-8.71E-02	8.65E-03
-2.02E-01	-4.02E-02	-2.18E-01	-8.00E-03	-2.20E-01	2.99E-02	-2.01E-01	6.02E-02	-1.65E-01	7.38E-02	-1.27E-01	7.78E-02	-9.72E-02	7.32E-02	-8.69E-02	5.80E-02	-9.31E-02	3.40E-02	-9.20E-02	1.61E-02
-1.98E-01	-4.41E-02	-2.17E-01	-9.52E-03	-2.19E-01	3.12E-02	-1.97E-01	6.45E-02	-1.61E-01	8.49E-02	-1.27E-01	9.54E-02	-9.94E-02	8.59E-02	-8.96E-02	5.86E-02	-9.76E-02	2.87E-02	-9.16E-02	9.12E-03
-2.04E-01	-4.68E-02	-2.24E-01	-1.91E-02	-2.24E-01	1.67E-02	-2.00E-01	4.96E-02	-1.69E-01	7.73E-02	-1.32E-01	8.80E-02	-1.02E-01	8.66E-02	-8.70E-02	6.21E-02	-8.29E-02	3.62E-02	-6.91E-02	2.14E-02
-2.09E-01	-3.21E-02	-2.26E-01	-7.77E-03	-2.21E-01	2.41E-02	-2.02E-01	5.44E-02	-1.71E-01	7.27E-02	-1.35E-01	7.58E-02	-1.05E-01	7.26E-02	-8.67E-02	5.42E-02	-7.86E-02	3.38E-02	-6.38E-02	2.11E-02
-2.05E-01	-3.96E-02	-2.25E-01	-1.09E-02	-2.27E-01	2.41E-02	-2.02E-01	5.33E-02	-1.65E-01	7.39E-02	-1.27E-01	8.04E-02	-9.81E-02	7.80E-02	-8.73E-02	5.68E-02	-9.00E-02	3.27E-02	-8.92E-02	1.46E-02
-1.99E-01	-4.47E-02	-2.26E-01	-1.78E-02	-2.25E-01	1.83E-02	-2.00E-01	5.43E-02	-1.68E-01	8.44E-02	-1.32E-01	9.57E-02	-9.86E-02	8.30E-02	-8.07E-02	6.29E-02	-8.26E-02	3.72E-02	-7.97E-02	1.68E-02
-2.15E-01	-3.98E-02	-2.35E-01	-8.26E-03	-2.31E-01	3.15E-02	-2.04E-01	6.74E-02	-1.64E-01	8.89E-02	-1.22E-01	9.17E-02	-8.83E-02	7.64E-02	-7.42E-02	5.72E-02	-8.56E-02	2.91E-02	-8.02E-02	8.49E-03
-1.95E-01	-4.97E-02	-2.20E-01	-1.80E-02	-2.29E-01	2.19E-02	-2.14E-01	5.75E-02	-1.80E-01	7.93E-02	-1.40E-01	8.40E-02	-1.05E-01	7.97E-02	-8.76E-02	6.53E-02	-8.64E-02	4.20E-02	-7.73E-02	2.40E-02
-1.94E-01	-4.64E-02	-2.26E-01	-1.89E-02	-2.35E-01	1.93E-02	-2.16E-01	5.61E-02	-1.82E-01	8.33E-02	-1.43E-01	9.29E-02	-1.09E-01	8.80E-02	-9.12E-02	6.52E-02	-8.68E-02	4.16E-02	-8.04E-02	2.12E-02
-1.96E-01	-4.78E-02	-2.16E-01	-1.47E-02	-2.30E-01	2.17E-02	-2.15E-01	5.37E-02	-1.81E-01	7.39E-02	-1.42E-01	8.06E-02	-1.08E-01	7.80E-02	-9.04E-02	6.21E-02	-8.53E-02	4.11E-02	-7.39E-02	2.61E-02
-2.01E-01	-4.59E-02	-2.25E-01	-1.59E-02	-2.27E-01	2.30E-02	-2.06E-01	5.86E-02	-1.70E-01	8.20E-02	-1.33E-01	9.34E-02	-1.03E-01	9.07E-02	-8.91E-02	6.71E-02	-8.91E-02	3.82E-02	-7.77E-02	1.95E-02
-2.17E-01	-4.53E-02	-2.41E-01	-1.64E-02	-2.36E-01	2.21E-02	-2.04E-01	5.65E-02	-1.56E-01	7.66E-02	-1.10E-01	8.22E-02	-7.75E-02	7.91E-02	-6.98E-02	6.46E-02	-8.23E-02	3.70E-02	-8.99E-02	1.71E-02
-2.01E-01	-3.25E-02	-2.24E-01	-6.82E-03	-2.28E-01	2.57E-02	-2.13E-01	5.56E-02	-1.85E-01	7.56E-02	-1.51E-01	8.18E-02	-1.20E-01	7.96E-02	-9.90E-02	5.92E-02	-8.37E-02	4.07E-02	-6.15E-02	3.12E-02
-1.97E-01	-4.01E-02	-2.18E-01	-1.47E-02	-2.23E-01	2.06E-02	-2.10E-01	5.30E-02	-1.80E-01	7.25E-02	-1.47E-01	8.06E-02	-1.16E-01	7.84E-02	-9.44E-02	5.80E-02	-7.91E-02	4.33E-02	-6.05E-02	3.49E-02
-1.98E-01	-4.11E-02	-2.23E-01	-1.11E-02	-2.32E-01	2.53E-02	-2.14E-01	5.66E-02	-1.79E-01	7.68E-02	-1.41E-01	8.39E-02	-1.05E-01	7.54E-02	-8.22E-02	6.03E-02	-7.50E-02	4.03E-02	-6.01E-02	2.58E-02
-2.06E-01	-3.83E-02	-2.27E-01	-1.16E-02	-2.29E-01	2.42E-02	-2.11E-01	5.64E-02	-1.76E-01	7.53E-02	-1.39E-01	8.25E-02	-1.06E-01	7.42E-02	-8.50E-02	5.36E-02	-7.63E-02	3.24E-02	-6.35E-02	1.82E-02
-2.09E-01	-3.78E-02	-2.27E-01	-9.37E-03	-2.26E-01	2.58E-02	-2.00E-01	5.62E-02	-1.66E-01	7.90E-02	-1.28E-01	8.26E-02	-9.76E-02	7.95E-02	-8.27E-02	5.77E-02	-7.94E-02	3.30E-02	-7.51E-02	1.62E-02
-2.00E-01	-4.70E-02	-2.24E-01	-1.60E-02	-2.30E-01	2.31E-02	-2.11E-01	5.72E-02	-1.73E-01	7.66E-02	-1.31E-01	8.23E-02	-9.76E-02	8.03E-02	-8.27E-02	6.32E-02	-8.44E-02	3.67E-02	-7.68E-02	1.81E-02
-2.05E-01	-4.01E-02	-2.29E-01	-1.46E-02	-2.31E-01	2.09E-02	-2.12E-01	5.50E-02	-1.79E-01	7.84E-02	-1.44E-01	8.92E-02	-1.13E-01	8.33E-02	-9.27E-02	5.94E-02	-8.13E-02	3.64E-02	-6.35E-02	2.14E-02
-2.05E-01	-3.85E-02	-2.24E-01	-1.13E-02	-2.26E-01	2.25E-02	-2.07E-01	5.23E-02	-1.72E-01	7.11E-02	-1.36E-01	8.19E-02	-1.06E-01	8.41E-02	-8.89E-02	6.81E-02	-8.24E-02	4.55E-02	-6.77E-02	3.29E-02
-2.08E-01	-4.08E-02	-2.30E-01	-1.23E-02	-2.35E-01	2.34E-02	-2.15E-01	5.42E-02	-1.78E-01	7.25E-02	-1.38E-01	7.89E-02	-1.05E-01	7.90E-02	-8.71E-02	6.22E-02	-8.17E-02	3.86E-02	-6.66E-02	2.62E-02
-2.01E-01	-4.14E-02	-2.21E-01	-1.40E-02	-2.23E-01	2.23E-02	-2.06E-01	5.52E-02	-1.75E-01	7.52E-02	-1.42E-01	8.43E-02	-1.12E-01	8.09E-02	-9.35E-02	5.80E-02	-8.22E-02	3.65E-02	-6.19E-02	2.68E-02
-2.01E-01	-5.02E-02	-2.24E-01	-2.01E-02	-2.30E-01	1.90E-02	-2.11E-01	5.50E-02	-1.76E-01	8.00E-02	-1.40E-01	9.22E-02	-1.07E-01	8.90E-02	-9.08E-02	7.33E-02	-8.85E-02	4.86E-02	-7.45E-02	3.36E-02
-1.98E-01	-3.54E-02	-2.16E-01	-7.86E-03	-2.19E-01	2.60E-02	-2.09E-01	5.44E-02	-1.82E-01	6.88E-02	-1.51E-01	7.72E-02	-1.20E-01	7.26E-02	-9.80E-02	5.75E-02	-8.57E-02	4.10E-02	-6.37E-02	3.58E-02
-2.00E-01	-3.35E-02	-2.26E-01	-6.34E-03	-2.31E-01	2.75E-02	-2.15E-01	5.77E-02	-1.84E-01	7.67E-02	-1.48E-01	8.51E-02	-1.14E-01	8.05E-02	-9.09E-02	6.28E-02	-7.85E-02	4.34E-02	-5.91E-02	3.24E-02
-1.98E-01	-3.83E-02	-2.12E-01	-9.59E-03	-2.16E-01	2.48E-02	-2.06E-01	5.34E-02	-1.79E-01	6.86E-02	-1.49E-01	7.71E-02	-1.18E-01	7.34E-02	-9.79E-02	5.99E-02	-8.81E-02	4.29E-02	-6.75E-02	3.51E-02
-1.90E-01	-4.40E-02	-2.15E-01	-1.78E-02	-2.25E-01	1.60E-02	-2.11E-01	4.72E-02	-1.81E-01	6.86E-02	-1.48E-01	8.10E-02	-1.15E-01	7.64E-02	-9.35E-02	5.64E-02	-8.31E-02	3.68E-02	-6.08E-02	2.88E-02
-2.07E-01	-3.82E-02	-2.28E-01	-1.09E-02	-2.28E-01	2.36E-02	-2.08E-01	5.42E-02	-1.73E-01	7.23E-02	-1.33E-01	7.55E-02	-9.98E-02	7.10E-02	-8.21E-02	6.14E-02	-7.80E-02	4.48E-02	-7.14E-02	3.12E-02
-1.94E-01	-3.14E-02	-2.18E-01	-4.60E-03	-2.25E-01	2.63E-02	-2.07E-01	5.18E-02	-1.76E-01	7.05E-02	-1.41E-01	7.66E-02	-1.11E-01	7.43E-02	-9.58E-02	5.66E-02	-9.47E-02	3.32E-02	-7.82E-02	2.12E-02
-2.00E-01	-4.30E-02	-2.18E-01	-1.38E-02	-2.21E-01	2.29E-02	-2.02E-01	5.48E-02	-1.68E-01	7.40E-02	-1.33E-01	8.16E-02	-1.03E-01	7.79E-02	-8.83E-02	5.92E-02	-8.49E-02	3.69E-02	-7.85E-02	1.98E-02
-2.01E-01	-4.03E-02	-2.19E-01	-1.88E-02	-2.17E-01	1.25E-02	-1.98E-01	4.41E-02	-1.72E-01	6.86E-02	-1.38E-01	7.46E-02	-1.09E-01	7.22E-02	-9.35E-02	5.66E-02	-8.97E-02	3.88E-02	-8.22E-02	2.35E-02
-1.93E-01	-4.56E-02	-2.08E-01	-1.46E-02	-2.11E-01	2.46E-02	-1.94E-01	5.92E-02	-1.63E-01	8.15E-02	-1.34E-01	9.31E-02	-1.07E-01	8.74E-02	-9.53E-02	6.81E-02	-9.75E-02	4.31E-02	-9.08E-02	2.65E-02
-2.00E-01	-5.31E-02	-2.20E-01	-1.80E-02	-2.22E-01	2.52E-02	-2.03E-01	6.29E-02	-1.62E-01	8.38E-02	-1.23E-01	9.88E-02	-9.21E-02	1.01E-01	-7.74E-02	7.95E-02	-7.60E-02	5.14E-02	-7.05E-02	3.29E-02
-1.97E-01	-5.03E-02	-2.20E-01	-2.13E-02	-2.25E-01	1.68E-02	-2.09E-01	5.14E-02	-1.75E-01	7.18E-02	-1.35E-01	7.71E-02	-1.00E-01	7.83E-02	-8.29E-02	6.26E-02	-7.98E-02	3.91E-02	-6.49E-02	2.51E-02
-2.00E-01	-3.87E-02	-2.22E-01	-1.09E-02	-2.26E-01	2.39E-02	-2.07E-01	5.44E-02	-1.72E-01	7.31E-02	-1.38E-01	8.26E-02	-1.08E-01	7.70E-02	-9.14E-02	5.87E-02	-8.72E-02	3.75E-02	-7.79E-02	2.26E-02
-2.00E-01	-4.24E-02	-2.26E-01	-1.57E-02	-2.34E-01	1.77E-02	-2.12E-01	4.83E-02	-1.77E-01	7.22E-02	-1.42E-01	8.51E-02	-1.09E-01	7.59E-02	-8.82E-02	5.77E-02	-7.99E-02	4.29E-02	-7.42E-02	2.51E-02
-2.15E-01	-4.54E-02	-2.35E-01	-1.43E-02	-2.33E-01	2.46E-02	-2.05E-01	5.77E-02	-1.62E-01	7.80E-02	-1.19E-01	8.72E-02	-8.62E-02	8.09E-02	-7.25E-02	5.89E-02	-7.44E-02	3.44E-02	-7.91E-02	1.65E-02
-2.07E-01	-5.66E-02	-2.32E-01	-2.78E-02	-2.34E-01	1.42E-02	-2.12E-01	5.38E-02	-1.67E-01	7.67E-02	-1.22E-01	8.97E-02	-8.89E-02	9.14E-02	-7.29E-02	7.27E-02	-7.20E-02	4.95E-02	-7.04E-02	2.89E-02
-2.06E-01	-4.97E-02	-2.23E-01	-2.06E-02	-2.25E-01	1.83E-02	-2.07E-01	5.29E-02	-1.68E-01	7.24E-02	-1.31E-01	8.47E-02	-1.03E-01	8.70E-02	-8.91E-02	6.91E-02	-8.70E-02	4.27E-02	-7.69E-02	2.64E-02
-1.98E-01	-4.20E-02	-2.23E-01	-1.72E-02	-2.28E-01	1.70E-02	-2.13E-01	4.94E-02	-1.81E-01	7.04E-02	-1.44E-01	7.77E-02	-1.10E-01	7.55E-02	-9.00E-02	5.89E-02	-8.35E-02	3.87E-02	-6.39E-02	2.79E-02
-2.01E-01	-3.70E-02	-2.15E-01	-9.94E-03	-2.18E-01	2.25E-02	-1.97E-01	5.00E-02	-1.66E-01	6.96E-02	-1.34E-01	7.85E-02	-1.04E-01	6.89E-02	-8.56E-02	5.39E-02	-7.92E-02	3.50E-02	-7.04E-02	2.08E-02
-2.07E-01	-6.42E-02	-2.26E-01	-2.99E-02	-2.29E-01	1.63E-02	-2.01E-01	5.84E-02	-1.62E-01	9.00E-02	-1.17E-01	9.78E-02	-8.29E-02	9.47E-02	-7.22E-02	7.14E-02	-7.99E-02	4.32E-02	-8.57E-02	2.40E-02
-2.04E-01	-3.32E-02	-2.22E-01	-7.54E-03	-2.20E-01	2.67E-02	-2.04E-01	5.87E-02	-1.74E-01	7.57E-02	-1.41E-01	8.05E-02	-1.10E-01	6.98E-02	-9.07E-02	5.05E-02	-8.53E-02	2.78E-02	-6.84E-02	1.65E-02
-2.00E-01	-4.60E-02	-2.25E-01	-1.75E-02	-2.30E-01	2.02E-02	-2.09E-01	5.55E-02	-1.78E-01	8.18E-02	-1.41E-01	8.98E-02	-1.07E-01	8.42E-02	-8.95E-02	6.24E-02	-8.67E-02	3.70E-02	-6.94E-02	2.44E-02
-2.00E-01	-3.48E-02	-2.20E-01	-1.15E-02	-2.24E-01	1.80E-02	-2.07E-01	4.51E-02	-1.82E-01	6.77E-02	-1.49E-01	7.20E-02	-1.18E-01	6.55E-02	-9.44E-02	4.86E-02	-7.95E-02	3.55E-02	-5.95E-02	2.88E-02
-2.00E-01	-4.17E-02	-2.22E-01	-1.31E-02	-2.29E-01	2.28E-02	-2.13E-01	5.40E-02	-1.79E-01	7.27E-02	-1.45E-01	8.42E-02	-1.11E-01	7.56E-02	-8.81E-02	5.77E-02	-7.96E-02	3.63E-02	-6.11E-02	2.37E-02
-2.03E-01	-4.29E-02	-2.24E-01	-1.38E-02	-2.25E-01	2.45E-02	-2.07E-01	5.91E-02	-1.73E-01	7.93E-02	-1.36E-01	8.65E-02	-1.04E-01	7.91E-02	-8.63E-02	6.25E-02	-8.48E-02	3.90E-02	-7.37E-02	2.26E-02
-1.96E-01	-4.32E-02	-2.16E-01	-1.63E-02	-2.18E-01	1.99E-02	-2.05E-01	5.21E-02	-1.73E-01	6.96E-02	-1.38E-01	7.69E-02	-1.09E-01	7.45E-02	-9.38E-02	5.73E-02	-9.03E-02	3.52E-02	-7.80E-02	2.14E-02
-1.93E-01	-3.97E-02	-2.10E-01	-1.57E-02	-2.13E-01	1.67E-02	-2.02E-01	4.61E-02	-1.78E-01	6.49E-02	-1.51E-01	7.58E-02	-1.25E-01	7.37E-02	-1.07E-01	6.11E-02	-9.69E-02	4.75E-02	-8.05E-02	3.91E-02
-1.97E-01	-4.68E-02	-2.19E-01	-1.79E-02	-2.26E-01	1.88E-02	-2.11E-01	5.22E-02	-1.80E-01	7.48E-02	-1.45E-01	8.34E-02	-1.13E-01	7.85E-02	-9.15E-02	5.81E-02	-8.05E-02	3.86E-02	-6.12E-02	2.89E-02
-1.99E-01	-3.51E-02	-2.25E-01	-1.20E-02	-2.31E-01	1.92E-02	-2.15E-01	4.85E-02	-1.84E-01	6.88E-02	-1.50E-01	7.85E-02	-1.18E-01	7.31E-02	-9.62E-02	5.68E-02	-8.31E-02	4.40E-02	-7.07E-02	2.90E-02
-1.80E-01	-5.06E-02	-2.16E-01	-2.20E-02	-2.33E-01	1.76E-02	-2.23E-01	5.44E-02	-1.88E-01	7.71E-02	-1.47E-01	8.90E-02	-1.11E-01	8.75E-02	-8.90E-02	6.57E-02	-7.82E-02	4.60E-02	-6.66E-02	2.90E-02
-2.08E-01	-3.87E-02	-2.32E-01	-1.26E-02	-2.36E-01	2.15E-02	-2.15E-01	5.22E-02	-1.80E-01	7.18E-02	-1.42E-01	7.91E-02	-1.09E-01	7.06E-02	-8.71E-02	5.21E-02	-7.77E-02	3.19E-02	-6.43E-02	1.80E-02
-2.07E-01	-4.07E-02	-2.26E-01	-1.65E-02	-2.23E-01	1.90E-02	-2.04E-01	5.41E-02	-1.72E-01	7.63E-02	-1.37E-01	8.37E-02	-1.09E-01	8.08E-02	-9.34E-02	5.68E-02	-8.60E-02	3.33E-02	-7.39E-02	1.67E-02
-2.07E-01	-4.36E-02	-2.27E-01	-1.83E-02	-2.26E-01	1.74E-02	-2.05E-01	5.11E-02	-1.69E-01	7.33E-02	-1.33E-01	8.33E-02	-1.04E-01	8.07E-02	-8.94E-02	6.56E-02	-8.63E-02	4.53E-02	-8.39E-02	2.75E-02
-2.03E-01	-3.82E-02	-2.35E-01	-1.02E-02	-2.48E-01	2.31E-02	-2.25E-01	5.29E-02	-1.85E-01	7.47E-02	-1.42E-01	8.32E-02	-1.05E-01	7.91E-02	-8.44E-02	5.95E-02	-7.87E-02	3.38E-02	-6.57E-02	1.63E-02
-1.93E-01	-4.64E-02	-2.21E-01	-1.78E-02	-2.32E-01	1.91E-02	-2.16E-01	5.30E-02	-1.84E-01	7.64E-02	-1.47E-01	8.54E-02	-1.14E-01	8.33E-02	-9.31E-02	6.30E-02	-8.32E-02	4.23E-02	-6.73E-02	2.85E-02
-2.03E-01	-3.85E-02	-2.24E-01	-1.27E-02	-2.28E-01	2.09E-02	-2.11E-01	5.12E-02	-1.79E-01	7.08E-02	-1.42E-01	7.82E-02	-1.11E-01	7.61E-02	-9.09E-02	5.72E-02	-8.09E-02	3.82E-02	-6.38E-02	2.94E-02
-1.96E-01	-4.38E-02	-2.21E-01	-1.47E-02	-2.29E-01	2.29E-02	-2.13E-01	5.74E-02	-1.83E-01	8.12E-02	-1.48E-01	8.91E-02	-1.19E-01	8.59E-02	-1.00E-01	5.85E-02	-8.16E-02	4.06E-02	-7.01E-02	2.15E-02
-1.87E-01	-5.92E-02	-2.22E-01	-2.77E-02	-2.39E-01	1.58E-02	-2.25E-01	5.76E-02	-1.88E-01	8.60E-02	-1.47E-01	9.67E-02	-1.09E-01	8.78E-02	-8.81E-02	6.58E-02	-8.77E-02	3.76E-02	-7.18E-02	1.99E-02
-2.08E-01	-3.21E-02	-2.29E-01	-4.11E-03	-2.31E-01	2.80E-02	-2.07E-01	5.44E-02	-1.70E-01	7.29E-02	-1.32E-01	7.91E-02	-1.02E-01	7.62E-02	-8.56E-02	6.44E-02	-8.35E-02	4.23E-02	-7.66E-02	2.50E-02
-1.96E-01	-3.78E-02	-2.15E-01	-1.26E-02	-2.18E-01	1.96E-02	-2.04E-01	4.89E-02	-1.75E-01	6.81E-02	-1.43E-01	7.72E-02	-1.15E-01	7.78E-02	-9.59E-02	5.99E-02	-8.37E-02	4.26E-02	-6.70E-02	3.29E-02
-2.03E-01	-5.20E-02	-2.30E-01	-2.21E-02	-2.33E-01	1.80E-02	-2.11E-01	5.63E-02	-1.75E-01	8.47E-02	-1.38E-01	9.80E-02	-1.05E-01	9.21E-02	-8.63E-02	6.70E-02	-8.12E-02	3.91E-02	-6.70E-02	2.19E-02
-1.93E-01	-3.81E-02	-2.17E-01	-1.32E-02	-2.23E-01	2.01E-02	-2.10E-01	5.00E-02	-1.78E-01	6.64E-02	-1.43E-01	7.63E-02	-1.12E-01	7.57E-02	-9.36E-02	6.01E-02	-8.73E-02	4.05E-02	-7.06E-02	3.16E-02
-1.96E-01	-4.59E-02	-2.20E-01	-1.47E-02	-2.29E-01	2.35E-02	-2.14E-01	5.68E-02	-1.79E-01	7.71E-02	-1.43E-01	8.78E-02	-1.12E-01	8.43E-02	-9.44E-02	6.01E-02	-8.75E-02	3.58E-02	-7.30E-02	1.90E-02
-2.02E-01	-4.06E-02	-2.28E-01	-1.64E-02	-2.33E-01	1.87E-02	-2.18E-01	5.13E-02	-1.83E-01	6.98E-02	-1.43E-01	7.78E-02	-1.08E-01	7.64E-02	-8.67E-02	6.01E-02	-7.74E-02	4.21E-02	-6.27E-02	2.95E-02
-1.94E-01	-3.96E-02	-2.20E-01	-1.61E-02	-2.29E-01	1.63E-02	-2.18E-01	4.63E-02	-1.90E-01	6.58E-02	-1.58E-01	7.61E-02	-1.27E-01	7.31E-02	-1.03E-01	5.43E-02	-8.47E-02	4.26E-02	-6.27E-02	3.57E-02
-2.03E-01	-4.49E-02	-2.20E-01	-1.64E-02	-2.20E-01	2.02E-02	-2.00E-01	5.35E-02	-1.67E-01	7.59E-02	-1.33E-01	8.82E-02	-1.03E-01	8.54E-02	-8.75E-02	6.56E-02	-8.47E-02	4.16E-02	-7.39E-02	2.75E-02
-2.05E-01	-4.17E-02	-2.26E-01	-1.09E-02	-2.25E-01	2.67E-02	-2.00E-01	5.91E-02	-1.62E-01	8.25E-02	-1.23E-01	9.06E-02	-9.21E-02	8.65E-02	-8.31E-02	6.61E-02	-9.26E-02	3.89E-02	-8.56E-02	2.09E-02
-1.95E-01	-5.11E-02	-2.20E-01	-2.14E-02	-2.26E-01	1.91E-02	-2.11E-01	5.63E-02	-1.76E-01	7.89E-02	-1.37E-01	8.65E-02	-1.05E-01	8.11E-02	-8.84E-02	6.20E-02	-8.68E-02	3.81E-02	-7.74E-02	2.11E-02
-1.99E-01	-3.31E-02	-2.16E-01	-4.41E-03	-2.23E-01	2.75E-02	-2.06E-01	5.32E-02	-1.77E-01	6.94E-02	-1.43E-01	7.29E-02	-1.13E-01	7.10E-02	-9.47E-02	5.36E-02	-8.57E-02	3.59E-02	-6.97E-02	2.71E-02
-1.91E-01	-3.24E-02	-2.26E-01	-1.02E-02	-2.38E-01	2.07E-02	-2.22E-01	5.05E-02	-1.89E-01	7.22E-02	-1.50E-01	8.08E-02	-1.15E-01	7.74E-02	-9.21E-02	6.03E-02	-8.22E-02	4.08E-02	-6.48E-02	2.66E-02
-1.89E-01	-4.03E-02	-2.11E-01	-1.12E-02	-2.23E-01	2.20E-02	-2.14E-01	5.05E-02	-1.87E-01	6.87E-02	-1.55E-01	7.74E-02	-1.23E-01	7.41E-02	-1.00E-01	5.97E-02	-8.71E-02	4.53E-02	-6.71E-02	3.79E-02
-1.98E-01	-5.13E-02	-2.22E-01	-2.44E-02	-2.25E-01	1.44E-02	-2.09E-01	5.19E-02	-1.76E-01	7.72E-02	-1.40E-01	8.82E-02	-1.09E-01	8.49E-02	-9.06E-02	6.20E-02	-8.13E-02	4.31E-02	-7.57E-02	2.52E-02

-1.97E-01	-3.29E-02	-2.19E-01	-4.56E-03	-2.27E-01	2.79E-02	-2.13E-01	5.42E-02	-1.81E-01	6.87E-02	-1.47E-01	7.58E-02	-1.17E-01	7.24E-02	-9.75E-02	5.24E-02	-8.83E-02	2.99E-02	-7.22E-02	1.60E-02
-1.99E-01	-3.31E-02	-2.14E-01	-8.73E-03	-2.18E-01	2.13E-02	-2.03E-01	4.78E-02	-1.77E-01	6.65E-02	-1.48E-01	7.66E-02	-1.20E-01	7.49E-02	-1.00E-01	5.81E-02	-8.59E-02	4.32E-02	-6.90E-02	3.37E-02
-2.09E-01	-3.69E-02	-2.27E-01	-7.69E-03	-2.29E-01	2.58E-02	-2.04E-01	5.20E-02	-1.65E-01	6.89E-02	-1.28E-01	8.12E-02	-9.69E-02	7.52E-02	-8.13E-02	5.85E-02	-8.08E-02	3.48E-02	-7.66E-02	1.76E-02
-2.07E-01	-4.04E-02	-2.23E-01	-1.33E-02	-2.25E-01	2.18E-02	-2.04E-01	5.26E-02	-1.71E-01	7.37E-02	-1.36E-01	8.28E-02	-1.08E-01	8.03E-02	-9.14E-02	5.87E-02	-8.38E-02	3.70E-02	-7.47E-02	2.04E-02
-2.00E-01	-3.57E-02	-2.19E-01	-1.22E-02	-2.19E-01	1.90E-02	-2.02E-01	4.85E-02	-1.71E-01	6.76E-02	-1.38E-01	7.80E-02	-1.09E-01	7.66E-02	-9.17E-02	5.74E-02	-8.33E-02	3.83E-02	-6.73E-02	2.67E-02
-1.96E-01	-4.65E-02	-2.17E-01	-1.79E-02	-2.20E-01	2.12E-02	-2.06E-01	5.70E-02	-1.75E-01	7.84E-02	-1.40E-01	8.48E-02	-1.09E-01	7.87E-02	-9.11E-02	5.63E-02	-8.12E-02	3.82E-02	-7.15E-02	2.33E-02
-1.93E-01	-4.73E-02	-2.21E-01	-1.91E-02	-2.26E-01	2.07E-02	-2.12E-01	5.88E-02	-1.82E-01	8.38E-02	-1.48E-01	9.38E-02	-1.19E-01	8.79E-02	-1.02E-01	6.01E-02	-9.16E-02	3.43E-02	-7.58E-02	1.88E-02
-2.08E-01	-3.84E-02	-2.25E-01	-1.26E-02	-2.26E-01	2.11E-02	-2.08E-01	5.18E-02	-1.75E-01	7.24E-02	-1.40E-01	8.34E-02	-1.10E-01	8.24E-02	-9.07E-02	6.16E-02	-8.00E-02	4.00E-02	-6.41E-02	2.61E-02
-2.03E-01	-3.72E-02	-2.23E-01	-9.94E-03	-2.26E-01	2.41E-02	-2.09E-01	5.34E-02	-1.75E-01	7.07E-02	-1.41E-01	7.86E-02	-1.12E-01	7.51E-02	-9.48E-02	5.34E-02	-8.72E-02	3.01E-02	-7.01E-02	1.76E-02
-2.03E-01	-3.91E-02	-2.24E-01	-1.14E-02	-2.26E-01	2.47E-02	-2.08E-01	5.73E-02	-1.75E-01	7.71E-02	-1.38E-01	8.35E-02	-1.07E-01	8.08E-02	-8.96E-02	6.19E-02	-8.54E-02	3.86E-02	-7.34E-02	2.24E-02
-1.94E-01	-3.93E-02	-2.11E-01	-1.38E-02	-2.14E-01	2.08E-02	-2.01E-01	5.32E-02	-1.77E-01	7.57E-02	-1.49E-01	8.58E-02	-1.23E-01	8.23E-02	-1.06E-01	6.38E-02	-9.80E-02	4.29E-02	-8.41E-02	2.85E-02
-1.94E-01	-4.06E-02	-2.21E-01	-1.39E-02	-2.31E-01	1.98E-02	-2.16E-01	4.98E-02	-1.83E-01	6.96E-02	-1.48E-01	8.06E-02	-1.16E-01	7.91E-02	-9.64E-02	6.25E-02	-8.87E-02	4.29E-02	-7.38E-02	2.92E-02
-2.03E-01	-5.12E-02	-2.23E-01	-1.79E-02	-2.22E-01	2.50E-02	-2.01E-01	6.36E-02	-1.63E-01	8.70E-02	-1.26E-01	9.78E-02	-9.67E-02	9.24E-02	-8.86E-02	6.51E-02	-9.69E-02	3.43E-02	-9.41E-02	1.44E-02
-2.02E-01	-3.80E-02	-2.26E-01	-9.29E-03	-2.32E-01	2.57E-02	-2.13E-01	5.63E-02	-1.81E-01	7.73E-02	-1.46E-01	8.31E-02	-1.14E-01	7.32E-02	-9.48E-02	5.57E-02	-9.08E-02	3.26E-02	-7.57E-02	2.12E-02
-2.06E-01	-3.86E-02	-2.34E-01	-1.09E-02	-2.38E-01	2.59E-02	-2.17E-01	5.90E-02	-1.80E-01	7.81E-02	-1.39E-01	7.97E-02	-1.03E-01	7.30E-02	-8.51E-02	5.53E-02	-8.49E-02	2.96E-02	-7.21E-02	1.17E-02
-2.07E-01	-4.79E-02	-2.32E-01	-1.63E-02	-2.31E-01	2.54E-02	-2.04E-01	6.31E-02	-1.64E-01	8.81E-02	-1.20E-01	8.99E-02	-8.51E-02	7.70E-02	-7.29E-02	5.90E-02	-8.45E-02	3.06E-02	-8.42E-02	1.01E-02
-2.09E-01	-3.85E-02	-2.27E-01	-1.19E-02	-2.31E-01	2.06E-02	-2.07E-01	4.86E-02	-1.74E-01	7.13E-02	-1.37E-01	8.05E-02	-1.05E-01	7.50E-02	-8.63E-02	5.63E-02	-8.02E-02	3.57E-02	-6.98E-02	1.99E-02
-2.02E-01	-3.85E-02	-2.21E-01	-9.99E-03	-2.24E-01	2.66E-02	-2.12E-01	5.88E-02	-1.83E-01	7.68E-02	-1.50E-01	8.37E-02	-1.18E-01	7.76E-02	-9.53E-02	5.74E-02	-8.07E-02	3.93E-02	-6.11E-02	2.96E-02
-1.99E-01	-4.70E-02	-2.21E-01	-1.72E-02	-2.25E-01	2.12E-02	-2.06E-01	5.61E-02	-1.73E-01	8.00E-02	-1.39E-01	8.97E-02	-1.08E-01	8.31E-02	-9.00E-02	5.91E-02	-8.05E-02	3.79E-02	-7.29E-02	2.01E-02
-2.02E-01	-3.45E-02	-2.23E-01	-1.28E-02	-2.26E-01	1.65E-02	-2.07E-01	4.43E-02	-1.78E-01	6.52E-02	-1.46E-01	7.66E-02	-1.17E-01	7.47E-02	-9.61E-02	5.91E-02	-8.38E-02	4.19E-02	-6.83E-02	3.06E-02
-1.95E-01	-4.35E-02	-2.20E-01	-1.54E-02	-2.30E-01	1.91E-02	-2.14E-01	5.14E-02	-1.84E-01	7.47E-02	-1.50E-01	8.51E-02	-1.16E-01	7.58E-02	-9.35E-02	6.25E-02	-8.42E-02	4.40E-02	-6.72E-02	3.15E-02
-2.03E-01	-3.93E-02	-2.26E-01	-1.22E-02	-2.31E-01	2.12E-02	-2.10E-01	5.01E-02	-1.74E-01	7.02E-02	-1.36E-01	7.92E-02	-1.03E-01	7.23E-02	-8.30E-02	5.72E-02	-7.74E-02	3.67E-02	-6.88E-02	2.10E-02
-2.03E-01	-5.61E-02	-2.25E-01	-2.34E-02	-2.31E-01	1.89E-02	-2.13E-01	5.70E-02	-1.76E-01	8.07E-02	-1.36E-01	8.99E-02	-1.02E-01	8.88E-02	-8.75E-02	7.18E-02	-8.91E-02	4.24E-02	-7.26E-02	2.59E-02
-2.00E-01	-5.25E-02	-2.30E-01	-2.05E-02	-2.37E-01	2.20E-02	-2.17E-01	6.05E-02	-1.76E-01	8.42E-02	-1.35E-01	9.55E-02	-1.01E-01	8.92E-02	-8.48E-02	6.63E-02	-8.34E-02	4.01E-02	-8.05E-02	2.06E-02
-1.96E-01	-3.35E-02	-2.19E-01	-1.07E-02	-2.25E-01	1.83E-02	-2.09E-01	4.47E-02	-1.79E-01	6.40E-02	-1.48E-01	7.74E-02	-1.18E-01	7.43E-02	-9.85E-02	5.35E-02	-8.58E-02	3.53E-02	-6.39E-02	2.87E-02
-2.01E-01	-3.43E-02	-2.21E-01	-8.37E-03	-2.25E-01	2.27E-02	-2.08E-01	4.81E-02	-1.73E-01	6.04E-02	-1.37E-01	6.92E-02	-1.08E-01	7.60E-02	-9.05E-02	6.00E-02	-8.23E-02	4.06E-02	-6.52E-02	3.12E-02
-1.95E-01	-3.42E-02	-2.22E-01	-7.82E-03	-2.30E-01	2.55E-02	-2.16E-01	5.39E-02	-1.82E-01	6.83E-02	-1.44E-01	7.37E-02	-1.13E-01	7.79E-02	-9.44E-02	5.55E-02	-8.32E-02	3.52E-02	-6.67E-02	2.05E-02
-2.04E-01	-5.66E-02	-2.29E-01	-3.01E-02	-2.36E-01	8.70E-03	-2.20E-01	4.58E-02	-1.85E-01	6.91E-02	-1.41E-01	7.38E-02	-1.05E-01	7.88E-02	-8.73E-02	7.31E-02	-8.21E-02	5.08E-02	-7.15E-02	3.21E-02
-2.01E-01	-4.72E-02	-2.27E-01	-2.13E-02	-2.29E-01	1.66E-02	-2.14E-01	5.40E-02	-1.81E-01	7.69E-02	-1.44E-01	8.80E-02	-1.13E-01	8.84E-02	-9.32E-02	6.20E-02	-7.87E-02	4.02E-02	-5.30E-02	3.48E-02
-1.97E-01	-4.05E-02	-2.19E-01	-9.89E-03	-2.29E-01	2.51E-02	-2.10E-01	5.42E-02	-1.79E-01	7.73E-02	-1.43E-01	8.27E-02	-1.12E-01	8.18E-02	-9.29E-02	5.89E-02	-8.39E-02	3.57E-02	-6.69E-02	2.19E-02
-2.05E-01	-4.97E-02	-2.29E-01	-1.62E-02	-2.30E-01	2.75E-02	-2.06E-01	6.48E-02	-1.60E-01	8.25E-02	-1.14E-01	8.58E-02	-8.57E-02	8.19E-02	-8.17E-02	5.26E-02	-9.28E-02	2.83E-02	-1.17E-01	2.45E-02
-1.99E-01	-5.21E-02	-2.20E-01	-2.14E-02	-2.24E-01	2.04E-02	-2.08E-01	5.93E-02	-1.76E-01	8.45E-02	-1.41E-01	9.45E-02	-1.09E-01	8.79E-02	-8.94E-02	6.55E-02	-8.00E-02	4.29E-02	-6.83E-02	2.81E-02
-2.03E-01	-4.11E-02	-2.20E-01	-1.29E-02	-2.25E-01	2.27E-02	-2.10E-01	5.33E-02	-1.77E-01	6.98E-02	-1.41E-01	7.64E-02	-1.11E-01	7.38E-02	-9.29E-02	5.44E-02	-8.48E-02	3.48E-02	-7.19E-02	2.00E-02
-2.06E-01	-3.45E-02	-2.30E-01	-6.26E-03	-2.36E-01	2.66E-02	-2.11E-01	5.41E-02	-1.72E-01	7.38E-02	-1.33E-01	7.89E-02	-1.00E-01	6.81E-02	-8.48E-02	5.20E-02	-8.55E-02	3.13E-02	-8.60E-02	1.41E-02
-1.96E-01	-4.78E-02	-2.20E-01	-1.54E-02	-2.28E-01	2.49E-02	-2.11E-01	6.01E-02	-1.76E-01	8.18E-02	-1.37E-01	8.87E-02	-1.03E-01	8.44E-02	-8.52E-02	6.53E-02	-8.26E-02	3.92E-02	-6.63E-02	2.65E-02
-2.02E-01	-3.82E-02	-2.24E-01	-1.10E-02	-2.27E-01	2.24E-02	-2.04E-01	5.08E-02	-1.67E-01	6.96E-02	-1.33E-01	8.47E-02	-1.04E-01	8.22E-02	-8.98E-02	5.95E-02	-8.83E-02	3.30E-02	-7.60E-02	1.58E-02
-2.05E-01	-4.41E-02	-2.22E-01	-1.25E-02	-2.26E-01	2.63E-02	-2.09E-01	5.86E-02	-1.72E-01	7.40E-02	-1.33E-01	7.92E-02	-1.01E-01	7.74E-02	-8.55E-02	6.29E-02	-8.43E-02	3.91E-02	-7.40E-02	2.36E-02
-1.97E-01	-4.65E-02	-2.17E-01	-1.81E-02	-2.23E-01	1.90E-02	-2.12E-01	5.17E-02	-1.82E-01	7.04E-02	-1.51E-01	8.13E-02	-1.25E-01	8.36E-02	-1.06E-01	5.78E-02	-8.56E-02	4.13E-02	-6.13E-02	3.55E-02
-1.92E-01	-3.95E-02	-2.19E-01	-1.66E-02	-2.25E-01	1.66E-02	-2.12E-01	4.88E-02	-1.83E-01	7.01E-02	-1.52E-01	8.21E-02	-1.21E-01	7.63E-02	-9.95E-02	5.53E-02	-8.49E-02	3.78E-02	-6.81E-02	2.28E-02
-2.02E-01	-3.99E-02	-2.17E-01	-1.31E-02	-2.18E-01	2.18E-02	-2.07E-01	5.27E-02	-1.81E-01	7.01E-02	-1.48E-01	7.42E-02	-1.19E-01	7.06E-02	-9.82E-02	5.48E-02	-8.66E-02	3.84E-02	-7.06E-02	2.78E-02
-2.03E-01	-2.77E-02	-2.22E-01	-3.35E-03	-2.23E-01	2.66E-02	-2.05E-01	5.31E-02	-1.74E-01	7.07E-02	-1.41E-01	7.88E-02	-1.10E-01	7.50E-02	-9.11E-02	5.31E-02	-7.95E-02	3.25E-02	-5.86E-02	2.36E-02
-2.03E-01	-3.15E-02	-2.20E-01	-1.03E-02	-2.15E-01	1.68E-02	-1.96E-01	4.46E-02	-1.67E-01	6.61E-02	-1.35E-01	7.31E-02	-1.04E-01	6.84E-02	-8.50E-02	5.49E-02	-7.77E-02	3.83E-02	-6.18E-02	3.05E-02
-2.05E-01	-4.19E-02	-2.24E-01	-1.21E-02	-2.24E-01	2.40E-02	-2.02E-01	5.46E-02	-1.65E-01	7.12E-02	-1.26E-01	7.62E-02	-9.49E-02	7.57E-02	-8.17E-02	5.47E-02	-8.19E-02	2.97E-02	-7.09E-02	1.21E-02
-2.00E-01	-4.04E-02	-2.17E-01	-1.21E-02	-2.18E-01	2.22E-02	-1.99E-01	5.12E-02	-1.66E-01	6.87E-02	-1.32E-01	7.99E-02	-1.03E-01	7.67E-02	-8.74E-02	5.54E-02	-8.40E-02	3.12E-02	-6.78E-02	1.73E-02
-1.89E-01	-4.72E-02	-2.15E-01	-1.35E-02	-2.29E-01	2.45E-02	-2.13E-01	5.73E-02	-1.78E-01	7.77E-02	-1.37E-01	8.42E-02	-1.02E-01	8.12E-02	-8.60E-02	6.97E-02	-8.70E-02	4.49E-02	-7.65E-02	2.78E-02
-2.08E-01	-4.75E-02	-2.25E-01	-1.62E-02	-2.27E-01	2.23E-02	-2.03E-01	5.46E-02	-1.63E-01	7.53E-02	-1.25E-01	8.85E-02	-9.60E-02	8.78E-02	-8.38E-02	6.68E-02	-8.52E-02	4.00E-02	-8.25E-02	2.10E-02
-2.04E-01	-3.61E-02	-2.24E-01	-8.02E-03	-2.25E-01	2.58E-02	-2.04E-01	5.40E-02	-1.68E-01	7.17E-02	-1.31E-01	7.81E-02	-9.97E-02	7.24E-02	-8.11E-02	5.10E-02	-7.36E-02	3.02E-02	-6.35E-02	1.44E-02
-1.99E-01	-4.15E-02	-2.23E-01	-1.26E-02	-2.28E-01	2.49E-02	-2.12E-01	5.78E-02	-1.78E-01	7.60E-02	-1.41E-01	8.48E-02	-1.07E-01	7.70E-02	-8.70E-02	5.92E-02	-8.42E-02	3.49E-02	-6.27E-02	2.73E-02
-1.93E-01	-5.96E-02	-1.96E-01	-2.34E-02	-2.17E-01	1.72E-02	-2.24E-01	5.29E-02	-1.91E-01	7.46E-02	-1.47E-01	8.43E-02	-1.09E-01	8.21E-02	-8.80E-02	6.80E-02	-8.44E-02	4.58E-02	-8.15E-02	2.57E-02
-2.04E-01	-3.68E-02	-2.26E-01	-1.14E-02	-2.26E-01	2.09E-02	-2.03E-01	5.03E-02	-1.69E-01	7.08E-02	-1.33E-01	7.81E-02	-1.03E-01	7.48E-02	-8.83E-02	5.57E-02	-8.51E-02	3.16E-02	-7.14E-02	1.85E-02
-1.98E-01	-4.22E-02	-2.15E-01	-1.43E-02	-2.19E-01	2.15E-02	-1.99E-01	5.37E-02	-1.71E-01	7.75E-02	-1.37E-01	8.36E-02	-1.06E-01	7.68E-02	-9.16E-02	6.09E-02	-9.22E-02	3.79E-02	-8.36E-02	2.09E-02
-2.05E-01	-3.69E-02	-2.21E-01	-9.23E-03	-2.21E-01	2.38E-02	-1.98E-01	5.08E-02	-1.63E-01	6.86E-02	-1.29E-01	7.81E-02	-9.92E-02	7.28E-02	-8.52E-02	5.79E-02	-8.55E-02	3.61E-02	-7.78E-02	2.04E-02
-1.97E-01	-3.13E-02	-2.17E-01	-5.79E-03	-2.19E-01	2.48E-02	-2.02E-01	5.14E-02	-1.72E-01	6.90E-02	-1.40E-01	7.76E-02	-1.13E-01	7.70E-02	-9.45E-02	5.92E-02	-8.38E-02	4.18E-02	-6.84E-02	2.96E-02
-2.07E-01	-3.77E-02	-2.21E-01	-1.15E-02	-2.18E-01	2.16E-02	-1.95E-01	5.09E-02	-1.63E-01	7.12E-02	-1.30E-01	7.97E-02	-1.01E-01	7.44E-02	-8.42E-02	5.51E-02	-7.86E-02	3.29E-02	-6.97E-02	1.87E-02
-1.99E-01	-3.69E-02	-2.21E-01	-9.39E-03	-2.26E-01	2.36E-02	-2.07E-01	5.16E-02	-1.73E-01	7.08E-02	-1.39E-01	7.99E-02	-1.09E-01	7.77E-02	-9.49E-02	5.94E-02	-9.24E-02	3.73E-02	-8.40E-02	2.17E-02
-1.99E-01	-4.72E-02	-2.22E-01	-1.80E-02	-2.26E-01	2.10E-02	-2.08E-01	5.63E-02	-1.73E-01	7.77E-02	-1.36E-01	8.53E-02	-1.04E-01	8.23E-02	-8.95E-02	6.12E-02	-9.01E-02	3.56E-02	-7.62E-02	1.79E-02
-1.80E-01	-5.24E-02	-2.09E-01	-2.33E-02	-2.23E-01	1.59E-02	-2.11E-01	5.30E-02	-1.80E-01	7.77E-02	-1.42E-01	8.81E-02	-1.08E-01	8.62E-02	-9.08E-02	7.01E-02	-8.78E-02	4.88E-02	-7.96E-02	3.15E-02
-1.99E-01	-3.56E-02	-2.19E-01	-1.03E-02	-2.22E-01	2.19E-02	-2.05E-01	5.09E-02	-1.75E-01	6.99E-02	-1.42E-01	7.74E-02	-1.11E-01	6.97E-02	-9.34E-02	5.24E-02	-8.84E-02	3.26E-02	-7.68E-02	1.76E-02
-1.99E-01	-3.12E-02	-2.18E-01	-7.07E-03	-2.17E-01	2.39E-02	-2.00E-01	5.27E-02	-1.72E-01	7.15E-02	-1.41E-01	7.87E-02	-1.13E-01	7.42E-02	-9.78E-02	5.57E-02	-9.47E-02	3.34E-02	-8.01E-02	1.92E-02
-1.98E-01	-3.86E-02	-2.22E-01	-1.36E-02	-2.25E-01	2.00E-02	-2.09E-01	5.14E-02	-1.78E-01	7.17E-02	-1.42E-01	8.04E-02	-1.09E-01	7.48E-02	-8.94E-02	5.42E-02	-8.31E-02	3.20E-02	-6.32E-02	2.41E-02
-2.11E-01	-4.02E-02	-2.37E-01	-9.50E-03	-2.35E-01	2.67E-02	-2.05E-01	5.60E-02	-1.58E-01	7.25E-02	-1.12E-01	8.12E-02	-7.89E-02	7.85E-02	-7.25E-02	6.05E-02	-9.01E-02	3.33E-02	-8.35E-02	1.28E-02
-2.03E-01	-4.13E-02	-2.25E-01	-1.10E-02	-2.24E-01	2.58E-02	-2.01E-01	5.70E-02	-1.62E-01	7.45E-02	-1.25E-01	8.67E-02	-9.69E-02	8.52E-02	-8.80E-02	6.55E-02	-9.48E-02	3.85E-02	-9.00E-02	2.11E-02
-1.98E-01	-3.74E-02	-2.27E-01	-1.02E-02	-2.32E-01	2.56E-02	-2.15E-01	5.81E-02	-1.79E-01	7.76E-02	-1.42E-01	8.57E-02	-1.08E-01	7.71E-02	-8.81E-02	6.22E-02	-8.85E-02	3.65E-02	-7.20E-02	2.26E-02
-2.01E-01	-4.34E-02	-2.30E-01	-1.51E-02	-2.34E-01	2.15E-02	-2.13E-01	5.38E-02	-1.73E-01	7.08E-02	-1.29E-01	7.25E-02	-9.38E-02	7.58E-02	-8.05E-02	6.58E-02	-8.40E-02	4.06E-02	-7.68E-02	2.22E-02
-2.04E-01	-3.88E-02	-2.29E-01	-1.26E-02	-2.30E-01	2.21E-02	-2.11E-01	5.38E-02	-1.74E-01	7.19E-02	-1.36E-01	7.97E-02	-1.02E-01	7.45E-02	-8.67E-02	5.33E-02	-8.75E-02	2.85E-02	-7.15E-02	1.32E-02
-1.98E-01	-3.47E-02	-2.40E-01	8.90E-04	-2.51E-01	4.15E-02	-2.20E-01	7.27E-02	-1.72E-01	8.90E-02	-1.19E-01	6.91E-02	-7.45E-02	6.11E-02	-5.92E-02	6.56E-02	-7.59E-02	3.97E-02	-1.03E-01	2.06E-02
-1.94E-01	-3.79E-02	-2.28E-01	-1.41E-02	-2.36E-01	2.10E-02	-2.19E-01	5.50E-02	-1.86E-01	7.85E-02	-1.50E-01	8.78E-02	-1.15E-01	7.78E-02	-9.12E-02	5.80E-02	-7.79E-02	4.25E-02	-7.19E-02	2.38E-02
-2.01E-01	-3.78E-02	-2.22E-01	-1.14E-02	-2.21E-01	2.42E-02	-2.03E-01	5.62E-02	-1.67E-01	7.31E-02	-1.34E-01	8.43E-02	-1.06E-01	7.89E-02	-9.09E-02	5.83E-02	-8.68E-02	3.67E-02	-8.41E-02	1.85E-02
-2.00E-01	-2.83E-02	-2.21E-01	-1.89E-03	-2.26E-01	2.57E-02	-2.02E-01	4.49E-02	-1.72E-01	6.83E-02	-1.38E-01	7.92E-02	-1.08E-01	8.05E-02	-9.42E-02	6.27E-02	-9.15E-02	3.99E-02	-7.81E-02	2.62E-02
-1.98E-01	-4.44E-02	-2.32E-01	-1.22E-02	-2.41E-01	2.92E-02	-2.19E-01	6.40E-02	-1.73E-01	7.97E-02	-1.25E-01	7.98E-02	-8.85E-02	7.31E-02	-7.86E-02	6.05E-02	-9.56E-02	3.41E-02	-1.03E-01	1.35E-02
-2.01E-01	-2.45E-02	-2.21E-01	2.72E-03	-2.25E-01	3.32E-02	-2.07E-01	5.73E-02	-1.74E-01	7.05E-02	-1.39E-01	7.40E-02	-1.07E-01	6.53E-02	-8.88E-02	5.53E-02	-8.28E-02	4.18E-02	-7.57E-02	2.70E-02
-1.95E-01	-2.91E-02	-2.11E-01	-4.00E-03	-2.16E-01	2.41E-02	-2.00E-01	4.74E-02	-1.73E-01	6.30E-02	-1.44E-01	7.05E-02	-1.18E-01	6.82E-02	-1.03E-01	5.10E-02	-9.58E-02	3.31E-02	-8.34E-02	2.01E-02
-1.90E-01	-4.65E-02	-2.20E-01	-1.30E-02	-2.30E-01	2.78E-02	-2.09E-01	6.27E-02	-1.68E-01	8.37E-02	-1.25E-01	9.22E-02	-9.20E-02	8.49E-02	-7.94E-02	6.34E-02	-8.46E-02	3.77E-02	-9.18E-02	1.79E-02
-2.03E-01	-3.58E-02	-2.24E-01	-8.49E-03	-2.25E-01	2.65E-02	-2.01E-01	5.59E-02	-1.62E-01	7.25E-02	-1.23E-01	7.86E-02	-9.33E-02	7.48E-02	-8.53E-02	5.12E-02	-9.60E-02	2.49E-02	-8.47E-02	8.29E-03
-2.08E-01	-3.10E-02	-2.27E-01	-7.34E-03	-2.25E-01	2.31E-02	-2.02E-01	5.07E-02	-1.66E-01	6.93E-02	-1.30E-01	7.74E-02	-9.91E-02	7.35E-02	-8.52E-02	5.66E-02	-8.72E-02	3.10E-02	-7.33E-02	1.71E-02
-1.95E-01	-3.48E-02	-2.18E-01	-7.88E-03	-2.24E-01	2.48E-02	-2.06E-01	5.31E-02	-1.75E-01	7.30E-02	-1.43E-01	8.33E-02	-1.13E-01	7.88E-02	-9.27E-02	5.93E-02	-8.25E-02	3.88E-02	-6.90E-02	2.42E-02
-2.12E-01	-4.37E-02	-2.28E-01	-1.46E-02	-2.21E-01	2.30E-02	-1.94E-01	5.68E-02	-1.52E-01	7.46E-02	-1.12E-01	8.51E-02	-8.34E-02	8.33E-02	-7.68E-02	6.18E-02	-8.67E-02	3.52E-02	-9.18E-02	1.82E-02
-1.98E-01	-2.84E-02	-2.10E-01	-1.45E-03	-2.13E-01	2.90E-02	-1.96E-01	5.20E-02	-1.66E-01	6.47E-02	-1.34E-01	6.92E-02	-1.05E-01	6.39E-02	-8.84E-02	4.85E-02	-8.25E-02	3.05E-02	-6.83E-02	2.13E-02
-2.05E-01	-3.17E-02	-2.23E-01	-6.42E-03	-2.22E-01	2.47E-02	-2.00E-01	5.18E-02	-1.68E-01	7.05E-02	-1.35E-01	7.79E-02	-1.07E-01	6.98E-02	-8.97E-02	4.92E-02	-8.39E-02	2.73E-02	-7.30E-02	1.30E-02
-1.99E-01	-3.05E-02	-2.33E-01	-4.58E-03	-2.34E-01	2.84E-02	-2.09E-01	5.89E-02	-1.69E-01	7.50E-02	-1.26E-01	7.26E-02	-9.37E-02	7.35E-02	-8.58E-02	4.94E-02	-9.63E-02	2.09E-02	-8.12E-02	3.04E-03
-1.99E-01	-2.69E-02	-2.15E-01	4.38E-03	-2.25E-01	3.49E-02	-2.08E-01	5.70E-02	-1.77E-01	6.98E-02	-1.44E-01	7.30E-02	-1.14E-01	6.96E-02	-9.66E-02	5.00E-02	-9.01E-02	2.71E-02	-7.21E-02	1.66E-02
-1.96E-01	-3.99E-02	-2.22E-01	-6.86E-03	-2.28E-01	3.39E-02	-2.13E-01	6.78E-02	-1.78E-01	8.33E-02	-1.36E-01	8.05E-02	-9.89E-02	6.99E-02	-8.09E-02	5.53E-02	-8.76E-02	2.90E-02	-7.13E-02	1.29E-02
-1.97E-01	-3.58E-02	-2.17E-01	-5.87E-03	-2.23E-01	2.82E-02	-2.05E-01	5.53E-02	-1.72E-01	6.98E-02	-1.35E-01	6.99E-02	-1.04E-01	7.42E-02	-8.78E-02	6.18E-02	-8.62E-02	3.86E-02	-6.92E-02	2.83E-02

-2.04E-01	-4.34E-02	-2.21E-01	-1.60E-02	-2.21E-01	1.89E-02	-1.99E-01	4.97E-02	-1.65E-01	7.05E-02	-1.30E-01	7.97E-02	-9.99E-02	7.32E-02	-8.44E-02	5.56E-02	-8.51E-02	3.14E-02	-7.13E-02	1.63E-02
-1.98E-01	-3.54E-02	-2.19E-01	-6.43E-03	-2.22E-01	2.67E-02	-2.02E-01	5.34E-02	-1.70E-01	6.91E-02	-1.32E-01	6.86E-02	-1.01E-01	7.37E-02	-8.63E-02	6.36E-02	-8.60E-02	3.99E-02	-7.26E-02	2.68E-02
-1.93E-01	-4.24E-02	-2.22E-01	-1.04E-02	-2.29E-01	3.13E-02	-2.16E-01	6.68E-02	-1.81E-01	8.25E-02	-1.38E-01	8.13E-02	-1.00E-01	6.89E-02	-8.07E-02	5.82E-02	-8.62E-02	3.21E-02	-7.21E-02	1.37E-02
-1.99E-01	-3.81E-02	-2.27E-01	-7.14E-03	-2.32E-01	3.06E-02	-2.13E-01	6.21E-02	-1.75E-01	7.63E-02	-1.33E-01	7.97E-02	-9.69E-02	7.25E-02	-8.04E-02	5.39E-02	-8.53E-02	2.77E-02	-6.87E-02	1.25E-02
-2.01E-01	-3.07E-02	-2.16E-01	-7.43E-03	-2.14E-01	2.21E-02	-1.96E-01	4.88E-02	-1.64E-01	6.25E-02	-1.31E-01	7.14E-02	-1.06E-01	7.41E-02	-9.48E-02	5.52E-02	-9.30E-02	3.27E-02	-8.07E-02	1.91E-02
-2.04E-01	-2.48E-02	-2.22E-01	-2.24E-03	-2.20E-01	2.48E-02	-1.98E-01	4.93E-02	-1.69E-01	6.95E-02	-1.37E-01	7.85E-02	-1.08E-01	7.44E-02	-8.98E-02	5.86E-02	-8.28E-02	3.83E-02	-6.73E-02	2.74E-02
-1.98E-01	-4.15E-02	-2.16E-01	-1.39E-02	-2.19E-01	2.04E-02	-2.01E-01	5.01E-02	-1.67E-01	6.74E-02	-1.32E-01	7.58E-02	-1.00E-01	7.22E-02	-8.78E-02	5.67E-02	-9.11E-02	3.53E-02	-7.96E-02	1.91E-02
-2.01E-01	-2.62E-02	-2.23E-01	4.59E-03	-2.30E-01	3.61E-02	-2.07E-01	5.98E-02	-1.75E-01	7.81E-02	-1.39E-01	7.59E-02	-1.08E-01	6.89E-02	-9.33E-02	5.01E-02	-9.26E-02	2.63E-02	-8.69E-02	9.51E-03
-2.15E-01	-3.39E-02	-2.32E-01	-2.48E-03	-2.29E-01	3.40E-02	-2.00E-01	5.97E-02	-1.57E-01	6.81E-02	-1.17E-01	6.13E-02	-9.14E-02	7.49E-02	-8.76E-02	5.30E-02	-1.00E-01	2.25E-02	-8.85E-02	1.35E-03
-2.02E-01	-3.58E-02	-2.25E-01	-6.18E-03	-2.29E-01	2.79E-02	-2.04E-01	5.54E-02	-1.64E-01	7.16E-02	-1.26E-01	8.11E-02	-9.80E-02	7.82E-02	-8.97E-02	5.96E-02	-9.86E-02	3.16E-02	-9.49E-02	1.15E-02
-2.18E-01	-3.47E-02	-2.38E-01	-4.65E-03	-2.29E-01	3.12E-02	-1.97E-01	6.19E-02	-1.51E-01	7.69E-02	-1.07E-01	7.96E-02	-7.67E-02	7.21E-02	-7.42E-02	5.86E-02	-9.79E-02	3.36E-02	-9.70E-02	1.17E-02
-2.01E-01	-3.03E-02	-2.18E-01	-3.99E-03	-2.19E-01	2.68E-02	-1.98E-01	5.12E-02	-1.63E-01	6.22E-02	-1.26E-01	6.86E-02	-9.64E-02	6.86E-02	-8.59E-02	5.69E-02	-9.19E-02	3.60E-02	-8.41E-02	2.05E-02
-2.07E-01	-3.10E-02	-2.20E-01	-8.21E-03	-2.20E-01	2.11E-02	-2.01E-01	4.67E-02	-1.70E-01	6.40E-02	-1.38E-01	7.28E-02	-1.10E-01	7.20E-02	-9.55E-02	5.60E-02	-9.06E-02	3.72E-02	-7.89E-02	2.52E-02
-1.99E-01	-3.68E-02	-2.25E-01	-8.21E-03	-2.30E-01	2.75E-02	-2.10E-01	5.84E-02	-1.75E-01	7.82E-02	-1.37E-01	8.61E-02	-1.03E-01	7.79E-02	-8.34E-02	5.68E-02	-8.25E-02	3.02E-02	-6.37E-02	1.76E-02
-1.98E-01	-2.85E-02	-2.21E-01	-3.87E-03	-2.24E-01	2.70E-02	-2.07E-01	5.37E-02	-1.75E-01	6.87E-02	-1.42E-01	7.50E-02	-1.13E-01	7.17E-02	-9.67E-02	5.55E-02	-9.29E-02	3.32E-02	-7.80E-02	2.08E-02
-2.01E-01	-3.19E-02	-2.19E-01	-4.29E-03	-2.17E-01	2.76E-02	-1.92E-01	5.41E-02	-1.58E-01	7.24E-02	-1.23E-01	7.56E-02	-9.49E-02	7.09E-02	-8.76E-02	5.56E-02	-9.79E-02	3.17E-02	-9.47E-02	1.38E-02
-2.04E-01	-3.43E-02	-2.24E-01	-7.49E-03	-2.26E-01	2.54E-02	-2.06E-01	5.37E-02	-1.73E-01	7.11E-02	-1.38E-01	7.79E-02	-1.08E-01	7.29E-02	-9.05E-02	5.63E-02	-8.64E-02	3.43E-02	-7.32E-02	1.94E-02
-2.02E-01	-3.49E-02	-2.24E-01	-1.00E-02	-2.28E-01	2.09E-02	-2.08E-01	4.87E-02	-1.75E-01	6.91E-02	-1.42E-01	8.03E-02	-1.13E-01	7.75E-02	-9.58E-02	5.92E-02	-8.64E-02	4.38E-02	-8.03E-02	2.88E-02
-2.14E-01	-4.38E-02	-2.35E-01	-1.17E-02	-2.24E-01	2.63E-02	-1.89E-01	6.15E-02	-1.43E-01	8.45E-02	-9.86E-02	8.90E-02	-6.93E-02	7.92E-02	-7.08E-02	5.43E-02	-9.70E-02	3.00E-02	-1.13E-01	1.72E-02
-1.93E-01	-3.44E-02	-2.11E-01	-8.57E-03	-2.15E-01	2.29E-02	-2.00E-01	4.98E-02	-1.69E-01	6.54E-02	-1.37E-01	7.39E-02	-1.10E-01	7.45E-02	-9.75E-02	6.22E-02	-9.69E-02	4.11E-02	-8.35E-02	2.87E-02
-1.98E-01	-2.55E-02	-2.16E-01	-3.06E-03	-2.16E-01	2.47E-02	-1.98E-01	4.92E-02	-1.68E-01	6.48E-02	-1.38E-01	7.09E-02	-1.11E-01	6.69E-02	-9.60E-02	5.08E-02	-9.02E-02	3.28E-02	-7.79E-02	2.20E-02
-1.97E-01	-3.79E-02	-2.23E-01	-8.45E-03	-2.31E-01	2.62E-02	-2.10E-01	5.57E-02	-1.74E-01	7.66E-02	-1.37E-01	8.64E-02	-1.03E-01	7.84E-02	-8.18E-02	5.92E-02	-7.40E-02	3.93E-02	-6.94E-02	2.11E-02
-1.88E-01	-2.70E-02	-2.13E-01	-5.66E-04	-2.19E-01	3.03E-02	-2.04E-01	5.51E-02	-1.73E-01	6.63E-02	-1.37E-01	6.93E-02	-1.05E-01	6.51E-02	-9.04E-02	4.92E-02	-9.15E-02	2.69E-02	-7.47E-02	1.55E-02
-1.99E-01	-2.94E-02	-2.22E-01	-3.26E-03	-2.25E-01	2.56E-02	-2.01E-01	4.80E-02	-1.69E-01	7.18E-02	-1.35E-01	8.26E-02	-1.04E-01	7.61E-02	-8.85E-02	5.81E-02	-8.74E-02	3.56E-02	-8.29E-02	2.14E-02
-1.92E-01	-3.87E-02	-2.18E-01	-1.24E-02	-2.23E-01	1.89E-02	-1.97E-01	4.66E-02	-1.63E-01	7.26E-02	-1.25E-01	8.16E-02	-9.47E-02	7.81E-02	-8.63E-02	6.13E-02	-9.65E-02	3.69E-02	-9.59E-02	1.85E-02
-1.95E-01	-5.29E-02	-2.23E-01	-1.93E-02	-2.33E-01	2.45E-02	-2.20E-01	6.39E-02	-1.86E-01	8.77E-02	-1.43E-01	9.24E-02	-1.07E-01	8.79E-02	-8.74E-02	6.81E-02	-8.37E-02	4.21E-02	-7.38E-02	2.23E-02
-1.95E-01	-4.27E-02	-2.17E-01	-1.72E-02	-2.20E-01	1.72E-02	-2.01E-01	4.92E-02	-1.66E-01	7.00E-02	-1.30E-01	8.44E-02	-1.02E-01	8.70E-02	-8.86E-02	7.18E-02	-8.80E-02	4.89E-02	-7.27E-02	3.32E-02
-2.02E-01	-3.03E-02	-2.19E-01	-5.11E-03	-2.20E-01	2.53E-02	-2.02E-01	5.09E-02	-1.70E-01	6.61E-02	-1.39E-01	7.56E-02	-1.11E-01	7.09E-02	-9.45E-02	5.37E-02	-9.00E-02	3.21E-02	-7.42E-02	2.02E-02
-1.99E-01	-3.53E-02	-2.15E-01	-9.56E-03	-2.18E-01	2.17E-02	-2.01E-01	4.86E-02	-1.73E-01	6.60E-02	-1.43E-01	7.52E-02	-1.16E-01	7.50E-02	-9.81E-02	5.64E-02	-8.74E-02	3.66E-02	-6.87E-02	2.81E-02
-1.98E-01	-5.25E-02	-2.24E-01	-2.33E-02	-2.29E-01	1.60E-02	-2.10E-01	5.17E-02	-1.69E-01	7.33E-02	-1.30E-01	8.59E-02	-9.67E-02	8.26E-02	-8.26E-02	6.62E-02	-8.43E-02	4.27E-02	-8.08E-02	2.25E-02
-2.07E-01	-3.58E-02	-2.28E-01	-9.18E-03	-2.29E-01	2.45E-02	-2.09E-01	5.42E-02	-1.73E-01	7.18E-02	-1.36E-01	7.94E-02	-1.04E-01	7.58E-02	-8.68E-02	5.95E-02	-8.30E-02	3.61E-02	-6.64E-02	2.35E-02
-2.04E-01	-3.42E-02	-2.25E-01	-4.61E-03	-2.29E-01	3.16E-02	-2.08E-01	6.23E-02	-1.73E-01	8.03E-02	-1.37E-01	8.71E-02	-1.07E-01	8.07E-02	-9.02E-02	5.86E-02	-8.50E-02	3.40E-02	-7.66E-02	1.59E-02
-1.99E-01	-4.29E-02	-2.20E-01	-1.23E-02	-2.26E-01	2.38E-02	-2.08E-01	5.42E-02	-1.74E-01	7.35E-02	-1.37E-01	8.06E-02	-1.06E-01	8.21E-02	-9.13E-02	6.08E-02	-8.85E-02	3.55E-02	-6.97E-02	2.21E-02
-2.00E-01	-4.86E-02	-2.23E-01	-2.11E-02	-2.27E-01	1.56E-02	-2.07E-01	4.90E-02	-1.68E-01	6.97E-02	-1.31E-01	8.29E-02	-1.02E-01	8.33E-02	-8.67E-02	6.51E-02	-8.41E-02	4.20E-02	-7.64E-02	2.47E-02
-2.02E-01	-3.63E-02	-2.21E-01	-1.08E-02	-2.23E-01	2.25E-02	-2.05E-01	5.23E-02	-1.73E-01	7.08E-02	-1.39E-01	7.83E-02	-1.08E-01	7.15E-02	-9.06E-02	5.74E-02	-8.49E-02	3.97E-02	-7.59E-02	2.57E-02
-1.99E-01	-3.37E-02	-2.20E-01	-4.47E-03	-2.29E-01	2.85E-02	-2.12E-01	5.50E-02	-1.78E-01	7.09E-02	-1.42E-01	7.77E-02	-1.11E-01	7.42E-02	-9.29E-02	5.72E-02	-8.74E-02	3.50E-02	-7.19E-02	2.24E-02
-2.05E-01	-2.98E-02	-2.24E-01	-4.87E-03	-2.24E-01	2.53E-02	-2.02E-01	5.11E-02	-1.67E-01	6.60E-02	-1.32E-01	7.48E-02	-1.04E-01	7.26E-02	-8.90E-02	5.60E-02	-8.46E-02	3.63E-02	-7.68E-02	2.09E-02
-2.02E-01	-3.86E-02	-2.21E-01	-1.22E-02	-2.24E-01	2.17E-02	-2.07E-01	5.22E-02	-1.76E-01	7.10E-02	-1.42E-01	7.92E-02	-1.11E-01	7.52E-02	-9.05E-02	5.33E-02	-7.73E-02	3.33E-02	-5.66E-02	2.14E-02
-1.98E-01	-2.56E-02	-2.17E-01	-3.62E-03	-2.19E-01	2.40E-02	-2.03E-01	4.94E-02	-1.77E-01	6.67E-02	-1.48E-01	7.37E-02	-1.22E-01	7.34E-02	-1.07E-01	5.37E-02	-9.63E-02	3.53E-02	-7.78E-02	2.46E-02
-1.97E-01	-4.14E-02	-2.09E-01	-1.50E-02	-2.12E-01	1.76E-02	-1.95E-01	4.63E-02	-1.67E-01	6.61E-02	-1.38E-01	7.63E-02	-1.12E-01	7.65E-02	-9.49E-02	6.05E-02	-8.62E-02	4.16E-02	-6.71E-02	3.47E-02
-2.04E-01	-3.37E-02	-2.25E-01	-8.46E-03	-2.28E-01	2.37E-02	-2.10E-01	5.25E-02	-1.78E-01	7.17E-02	-1.44E-01	7.95E-02	-1.13E-01	7.37E-02	-9.28E-02	5.88E-02	-8.35E-02	4.26E-02	-7.12E-02	2.98E-02
-2.00E-01	-4.09E-02	-2.22E-01	-6.50E-03	-2.29E-01	3.43E-02	-2.12E-01	6.82E-02	-1.80E-01	8.78E-02	-1.42E-01	8.96E-02	-1.08E-01	8.11E-02	-9.05E-02	5.62E-02	-8.71E-02	2.85E-02	-7.26E-02	1.13E-02
-2.04E-01	-5.72E-02	-2.24E-01	-2.56E-02	-2.28E-01	1.57E-02	-2.08E-01	5.33E-02	-1.71E-01	7.76E-02	-1.34E-01	8.99E-02	-1.01E-01	8.45E-02	-8.43E-02	6.38E-02	-8.15E-02	3.91E-02	-7.25E-02	2.17E-02
-2.05E-01	-3.84E-02	-2.24E-01	-1.08E-02	-2.27E-01	2.35E-02	-2.09E-01	5.18E-02	-1.72E-01	6.50E-02	-1.35E-01	7.33E-02	-1.05E-01	7.15E-02	-9.24E-02	5.44E-02	-9.52E-02	2.82E-02	-7.71E-02	1.61E-02
-1.96E-01	-4.41E-02	-2.25E-01	-1.18E-02	-2.33E-01	2.83E-02	-2.12E-01	6.36E-02	-1.78E-01	8.84E-02	-1.38E-01	9.01E-02	-1.02E-01	8.08E-02	-8.55E-02	5.85E-02	-8.92E-02	3.09E-02	-7.70E-02	1.13E-02
-2.03E-01	-5.29E-02	-2.13E-01	-2.74E-02	-2.08E-01	9.82E-03	-1.86E-01	4.65E-02	-1.58E-01	7.54E-02	-1.26E-01	8.76E-02	-1.00E-01	8.50E-02	-8.99E-02	6.86E-02	-9.04E-02	4.94E-02	-9.19E-02	3.13E-02
-1.99E-01	-3.93E-02	-2.18E-01	-1.29E-02	-2.21E-01	1.99E-02	-2.00E-01	4.79E-02	-1.65E-01	6.56E-02	-1.31E-01	7.75E-02	-1.02E-01	7.59E-02	-9.19E-02	5.79E-02	-9.73E-02	3.31E-02	-8.42E-02	1.71E-02
-2.00E-01	-2.45E-02	-2.12E-01	-1.13E-03	-2.10E-01	2.62E-02	-1.94E-01	4.92E-02	-1.68E-01	6.04E-02	-1.38E-01	6.16E-02	-1.12E-01	6.44E-02	-9.73E-02	4.88E-02	-9.05E-02	2.94E-02	-7.17E-02	2.35E-02
-2.04E-01	-2.62E-02	-2.11E-01	-2.97E-03	-2.08E-01	2.50E-02	-1.88E-01	4.74E-02	-1.59E-01	6.43E-02	-1.27E-01	7.09E-02	-1.01E-01	7.24E-02	-9.00E-02	6.28E-02	-9.04E-02	4.34E-02	-8.07E-02	3.17E-02
-2.01E-01	-2.85E-02	-2.10E-01	-5.74E-03	-2.06E-01	2.22E-02	-1.88E-01	4.71E-02	-1.59E-01	6.29E-02	-1.29E-01	7.27E-02	-1.03E-01	7.30E-02	-9.14E-02	6.28E-02	-9.03E-02	4.69E-02	-8.38E-02	3.52E-02
-1.97E-01	-3.12E-02	-2.17E-01	-8.66E-03	-2.21E-01	1.96E-02	-2.07E-01	4.43E-02	-1.78E-01	5.79E-02	-1.45E-01	6.66E-02	-1.14E-01	6.49E-02	-9.46E-02	5.07E-02	-8.42E-02	4.09E-02	-6.77E-02	3.52E-02
-1.94E-01	-2.69E-02	-2.07E-01	-9.33E-03	-2.04E-01	1.35E-02	-1.95E-01	3.76E-02	-1.73E-01	5.21E-02	-1.48E-01	6.32E-02	-1.23E-01	6.32E-02	-1.06E-01	4.86E-02	-9.21E-02	3.94E-02	-6.99E-02	3.51E-02
-1.93E-01	-3.58E-02	-2.09E-01	-1.37E-02	-2.13E-01	1.43E-02	-1.99E-01	4.03E-02	-1.77E-01	5.97E-02	-1.51E-01	6.96E-02	-1.28E-01	7.17E-02	-1.16E-01	6.11E-02	-1.13E-01	4.37E-02	-9.52E-02	3.66E-02
-1.91E-01	-2.33E-02	-2.12E-01	-1.71E-03	-2.20E-01	2.22E-02	-2.08E-01	4.28E-02	-1.86E-01	5.96E-02	-1.59E-01	6.70E-02	-1.31E-01	6.43E-02	-1.09E-01	5.30E-02	-9.34E-02	4.02E-02	-7.28E-02	3.66E-02
-1.96E-01	-2.99E-02	-2.04E-01	-8.29E-03	-2.06E-01	1.80E-02	-1.89E-01	3.90E-02	-1.64E-01	5.61E-02	-1.38E-01	6.79E-02	-1.15E-01	7.23E-02	-1.02E-01	5.66E-02	-9.21E-02	4.08E-02	-7.52E-02	3.06E-02
-2.00E-01	-2.72E-02	-2.15E-01	-6.48E-03	-2.14E-01	1.81E-02	-1.96E-01	4.05E-02	-1.70E-01	5.82E-02	-1.39E-01	6.63E-02	-1.11E-01	6.81E-02	-9.63E-02	5.86E-02	-9.10E-02	4.44E-02	-7.79E-02	3.88E-02
-2.01E-01	-2.99E-02	-2.19E-01	-5.16E-03	-2.23E-01	2.33E-02	-2.07E-01	4.69E-02	-1.78E-01	6.39E-02	-1.45E-01	7.14E-02	-1.14E-01	6.73E-02	-9.35E-02	5.11E-02	-8.08E-02	3.92E-02	-6.29E-02	2.88E-02
-2.03E-01	-4.08E-02	-2.24E-01	-1.56E-02	-2.22E-01	1.71E-02	-2.00E-01	4.79E-02	-1.65E-01	6.84E-02	-1.30E-01	8.20E-02	-1.01E-01	8.06E-02	-9.13E-02	6.26E-02	-9.73E-02	3.78E-02	-8.49E-02	2.31E-02
-1.96E-01	-2.65E-02	-2.06E-01	-2.21E-03	-2.07E-01	2.58E-02	-1.93E-01	4.84E-02	-1.68E-01	6.02E-02	-1.40E-01	6.66E-02	-1.15E-01	6.83E-02	-1.01E-01	5.26E-02	-9.44E-02	3.50E-02	-8.16E-02	2.33E-02
-1.96E-01	-3.14E-02	-2.08E-01	-5.87E-03	-2.11E-01	2.45E-02	-1.99E-01	5.04E-02	-1.75E-01	6.67E-02	-1.46E-01	7.21E-02	-1.16E-01	6.84E-02	-9.66E-02	5.52E-02	-8.55E-02	4.28E-02	-6.70E-02	3.41E-02
-2.02E-01	-2.27E-02	-2.13E-01	-3.43E-03	-2.13E-01	2.01E-02	-1.97E-01	4.00E-02	-1.70E-01	5.40E-02	-1.41E-01	6.09E-02	-1.13E-01	5.86E-02	-9.52E-02	4.70E-02	-8.62E-02	3.29E-02	-6.85E-02	2.52E-02
-1.88E-01	-1.90E-02	-1.94E-01	-1.07E-03	-1.95E-01	2.06E-02	-1.86E-01	3.92E-02	-1.69E-01	5.11E-02	-1.48E-01	6.00E-02	-1.27E-01	6.21E-02	-1.12E-01	4.98E-02	-9.92E-02	4.05E-02	-7.86E-02	3.48E-02
-1.96E-01	-1.98E-02	-2.09E-01	-4.34E-03	-2.08E-01	1.42E-02	-1.94E-01	3.17E-02	-1.72E-01	4.66E-02	-1.47E-01	5.54E-02	-1.21E-01	5.45E-02	-1.02E-01	4.42E-02	-8.91E-02	3.74E-02	-6.95E-02	3.47E-02
-1.85E-01	-3.29E-02	-2.02E-01	-7.49E-03	-2.05E-01	2.21E-02	-1.91E-01	4.79E-02	-1.67E-01	6.65E-02	-1.39E-01	7.66E-02	-1.15E-01	7.86E-02	-1.01E-01	5.85E-02	-9.15E-02	3.90E-02	-7.48E-02	2.82E-02
-1.97E-01	-2.14E-02	-2.10E-01	-5.80E-03	-2.10E-01	1.30E-02	-1.96E-01	3.03E-02	-1.72E-01	4.49E-02	-1.46E-01	5.53E-02	-1.20E-01	5.66E-02	-1.01E-01	4.54E-02	-8.70E-02	4.05E-02	-6.70E-02	3.70E-02
-1.98E-01	-2.06E-02	-2.10E-01	-8.72E-04	-2.09E-01	2.21E-02	-1.93E-01	4.14E-02	-1.67E-01	5.57E-02	-1.40E-01	6.78E-02	-1.16E-01	7.05E-02	-1.00E-01	5.82E-02	-9.08E-02	4.56E-02	-7.70E-02	3.65E-02
-2.06E-01	-2.84E-02	-2.13E-01	-5.41E-03	-2.03E-01	2.41E-02	-1.86E-01	5.26E-02	-1.56E-01	6.74E-02	-1.28E-01	7.57E-02	-1.04E-01	7.22E-02	-9.81E-02	5.46E-02	-1.08E-01	3.20E-02	-9.85E-02	1.95E-02
-1.85E-01	-1.41E-02	-1.97E-01	8.08E-03	-2.00E-01	3.17E-02	-1.90E-01	5.02E-02	-1.74E-01	6.29E-02	-1.53E-01	6.28E-02	-1.32E-01	5.39E-02	-1.11E-01	4.63E-02	-9.68E-02	3.64E-02	-7.78E-02	3.05E-02
-1.89E-01	-2.17E-02	-1.99E-01	-9.57E-04	-2.00E-01	2.29E-02	-1.87E-01	4.23E-02	-1.63E-01	5.37E-02	-1.39E-01	6.61E-02	-1.15E-01	6.86E-02	-9.76E-02	5.55E-02	-8.61E-02	4.15E-02	-6.83E-02	3.64E-02
-1.86E-01	-2.67E-02	-2.07E-01	-5.37E-03	-2.12E-01	2.05E-02	-1.99E-01	4.39E-02	-1.77E-01	6.38E-02	-1.55E-01	7.67E-02	-1.31E-01	7.40E-02	-1.12E-01	5.81E-02	-9.92E-02	4.35E-02	-8.29E-02	3.25E-02
-1.93E-01	-3.35E-02	-2.08E-01	-6.99E-03	-2.11E-01	2.36E-02	-1.96E-01	4.84E-02	-1.66E-01	6.17E-02	-1.38E-01	6.91E-02	-1.14E-01	6.59E-02	-1.04E-01	4.89E-02	-1.04E-01	2.61E-02	-9.46E-02	1.22E-02
-2.01E-01	-2.52E-02	-2.08E-01	-1.54E-04	-2.06E-01	2.88E-02	-1.89E-01	5.18E-02	-1.61E-01	6.48E-02	-1.32E-01	7.08E-02	-1.07E-01	6.57E-02	-9.38E-02	5.18E-02	-9.06E-02	3.47E-02	-8.56E-02	2.31E-02
-2.07E-01	-3.20E-02	-2.19E-01	-6.21E-03	-2.15E-01	2.56E-02	-1.94E-01	5.31E-02	-1.60E-01	6.91E-02	-1.29E-01	7.89E-02	-1.06E-01	8.05E-02	-9.66E-02	6.55E-02	-9.63E-02	4.66E-02	-9.64E-02	2.94E-02
-2.01E-01	-3.42E-02	-2.17E-01	-1.34E-02	-2.18E-01	1.37E-02	-2.03E-01	3.95E-02	-1.76E-01	5.86E-02	-1.45E-01	7.10E-02	-1.17E-01	7.19E-02	-9.90E-02	5.36E-02	-8.63E-02	3.80E-02	-6.35E-02	3.14E-02
-1.94E-01	-3.29E-02	-2.06E-01	-1.04E-02	-2.08E-01	1.58E-02	-1.93E-01	3.86E-02	-1.70E-01	5.72E-02	-1.41E-01	6.60E-02	-1.14E-01	6.87E-02	-9.83E-02	6.13E-02	-9.10E-02	5.15E-02	-7.69E-02	4.75E-02
-2.04E-01	-2.04E-02	-2.14E-01	-1.30E-03	-2.11E-01	2.09E-02	-1.94E-01	4.06E-02	-1.67E-01	5.18E-02	-1.39E-01	6.04E-02	-1.12E-01	5.86E-02	-9.62E-02	4.72E-02	-8.99E-02	3.29E-02	-7.19E-02	2.73E-02
-1.99E-01	-2.77E-02	-2.18E-01	-2.42E-03	-2.24E-01	2.56E-02	-2.09E-01	4.78E-02	-1.80E-01	6.17E-02	-1.49E-01	6.89E-02	-1.21E-01	6.90E-02	-1.01E-01	5.60E-02	-9.05E-02	4.01E-02	-7.56E-02	2.95E-02
-2.02E-01	-3.74E-02	-2.17E-01	-1.35E-02	-2.17E-01	1.50E-02	-1.98E-01	3.97E-02	-1.69E-01	5.92E-02	-1.36E-01	6.95E-02	-1.08E-01	7.49E-02	-9.13E-02	6.29E-02	-8.37E-02	4.63E-02	-6.55E-02	4.26E-02
-2.04E-01	-1.96E-02	-2.16E-01	1.49E-03	-2.14E-01	2.62E-02	-1.96E-01	4.69E-02	-1.66E-01	5.95E-02	-1.36E-01	6.81E-02	-1.09E-01	6.91E-02	-9.26E-02	5.23E-02	-8.34E-02	3.55E-02	-6.85E-02	2.35E-02
-1.99E-01	-2.29E-02	-2.14E-01	1.90E-03	-2.18E-01	2.87E-02	-2.04E-01	4.82E-02	-1.76E-01	5.88E-02	-1.45E-01	6.48E-02	-1.16E-01	6.62E-02	-9.70E-02	5.13E-02	-8.47E-02	3.72E-02	-6.53E-02	2.99E-02
