# Supplementary material for: THE CHANGING PACE OF INSULAR LIFE: 5000 YEARS OF MICROEVOLUTION IN THE ORKNEY VOLE (MICROTUS ARVALIS ORCADENSIS)
Source: Evolution. 2014 Jul 29;68(10):2804–20. doi: 10.1111/evo.12476 (PMC5366975; doi:10.1111/evo.12476)
Supplement: Supplementary file 1 — Table S1. [file EVO-68-2804-s001.zip › evo12476-sup-0002-table.pdf]

ID	WMO stations	Long	Lat	Alt	Pm	Pmax	Pmin	Tm	Tmax	Tmin
WSaud	Revel	2	43.4	800	69.92	94.78	32.67	13.81	22.75	5.78
WNcae	Caen	-0.36	49.18	78	57.13	93.78	36.33	10.97	17.70	4.83
WNcal	Lille	3.1	50.57	48	52.60	68.00	38.22	10.31	18.00	2.81
WNcan	Limoges	1.18	45.87	396	80.64	97.72	59.99	10.52	18.47	3.54
WNcdo	Dijon	5.08	47.27	614	64.00	84.31	51.54	10.32	19.64	1.82
WNhsa	Gap	6.1	44.7	775	73.90	145.38	43.00	10.42	20.13	1.54
WNidF	Trappes	2.02	48.77	168	56.99	70.55	48.00	10.02	17.87	3.20
WSlat	Nantes	-1.6	47.1	27	65.45	84.75	44.88	11.74	18.90	5.58
WSmor	Quimper	-3.45	47.7	52	89.05	145.33	47.00	11.77	17.98	6.11
Wsnoi	Chassiron/La Rochelle	-2.25	46.97	11	64.71	95.00	36.78	13.25	19.84	6.54
WNsm	Feins	-1.6	48.3	73	62.30	102.60	42.60	11.74	18.87	5.41
WSven	La Rochelle	1.15	46.15	4	64.71	95.00	36.78	13.10	20.63	6.24
WNvie	Poitiers	0.32	48.58	129	61.85	88.00	46.67	11.44	19.58	4.38
Wsyeu	Chassiron/La Rochelle	-2.35	46.71	20	64.71	95.00	36.78	13.25	19.84	6.54
CElsa	Hannover	9.7	52.47	102	82.32	128.67	45.67	11.04	16.59	5.71
Wnwol	Freiburg	7.85	48.0	56	55.36	76.62	34.98	8.99	17.41	1.11
OM	Kirkwall Airport	-2.9	58.95	40	83.57	115.06	49.02	7.76	12.80	3.74
Wngue	Guernsey Airport	-2.6	49.85	21	82.32	128.67	45.67	11.04	16.59	5.71
