# Supplementary material for: THE CHANGING PACE OF INSULAR LIFE: 5000 YEARS OF MICROEVOLUTION IN THE ORKNEY VOLE (MICROTUS ARVALIS ORCADENSIS)
Source: Evolution. 2014 Jul 29;68(10):2804–20. doi: 10.1111/evo.12476 (PMC5366975; doi:10.1111/evo.12476)
Supplement: Supplementary file 1 — Table S1. [file EVO-68-2804-s001.zip › evo12476-sup-0003-table.pdf]

SI3: geographic variables

Inter-island distances (metres) within the Orkney archipelago acquired from the EDINA Digimap collections software (Edinburgh University)

	Bur	Main	Rou	San	Shap	Sron	Wes
Burray	0						
Mainland	3005	0					
Rousay	48542	1463	0				
Sanday	41716	27707	20692	0			
Shapinsay	27403	2287	7711	12347	0		
S. Ronaldsay	658	6389	51057	44064	31407	0	
Westray	48965	28869	6499	10637	16196	52882	0

Distance (metres) from Mainland Orkney acquired from the EDINA Digimap collections software (Edinburgh University)

Rousay	1676
Burray	2066
Shapinsay	4473
S. Ronaldsay	5595
Westrey	15907
Sanday	22618

Island size (km2)

ID	Area
Mainland	538.93
Rousay	12.12
Westray	51.55
Sanday	57.97
Shapinsay	29.19
Burray	10.9
S. Ronaldsay	52.18
Noirmoutier	49
Yeu	23.32
Guenesey	78
