# Supplementary material for: THE CHANGING PACE OF INSULAR LIFE: 5000 YEARS OF MICROEVOLUTION IN THE ORKNEY VOLE (MICROTUS ARVALIS ORCADENSIS)
Source: Evolution. 2014 Jul 29;68(10):2804–20. doi: 10.1111/evo.12476 (PMC5366975; doi:10.1111/evo.12476)
Supplement: Supplementary file 1 — Table S1. [file EVO-68-2804-s001.zip › evo12476-sup-0004-table.pdf]

Supporting Information

Appendix S1. Specimen locations, sample size and cyt*b* sequences used for the calculation of pairwise genetic distances.

**Location**	**Locality/site**	**N**	**cyt *b***
Modern Orkney	Burray	2	MA254, MA255
	Mainland	19	MA176, MA261, MA177, MA178, MA179 MA180, MA181, MA182, MA221, MA201 MA223, MA267, MA202, MA204, MA263 MA264, MA219, MA233, MA220
	Rousay	1	MA256
	Sanday	3	MA248, MA250, MA247
	South Ronaldsay	15	MA136, MA137, MA138, MA139, MA140 MA141, MA142, MA156, MA157, MA158 MA159, MA160, MA173, MA174, MA175
	Westray	10	MA258, MA93, MA94, MA95, MA113 MA96, MA114, MA115, MA116, MA117
Ancient Orkney	Howe (Mainland)		R58, R59, R60
	Holm of Papa Westray North	3	R20, R23, R25
	(Westray)	3	
	Point of Cott (Westray)	3	R39, R44, R45
	Pierowall Quarry (Westray)	2	R99, R126
