# Supplementary material for: THE CHANGING PACE OF INSULAR LIFE: 5000 YEARS OF MICROEVOLUTION IN THE ORKNEY VOLE (MICROTUS ARVALIS ORCADENSIS)
Source: Evolution. 2014 Jul 29;68(10):2804–20. doi: 10.1111/evo.12476 (PMC5366975; doi:10.1111/evo.12476)
Supplement: Supplementary file 1 — Table S1. [file EVO-68-2804-s001.zip › evo12476-sup-0006-table.pdf]

	A_Bel_Med	A_Bel_Taw	A_Fr_Abeu	A_Fr_MG	A_Or_EB	A_Or_How	A_Or_HPW
A_Bel_Taw	1	-	-	-	-	-	-
A_Fr_Abeu	1	1	-	-	-	-	-
A_Fr_MG	1	1	1	-	-	-	-
A_Or_EB	0.0037	1	0.01548	4.80E-05	-	-	-
A_Or_How	4.30E-10	1	3.30E-10	1.70E-12	1	-	-
A_Or_HPW	1.30E-07	1	2.30E-07	6.70E-10	1	1	-
A_Or_LoN	2.70E-07	1	4.90E-07	1.40E-09	1	1	1
A_Or_PC	2.00E-08	1	2.30E-08	8.60E-11	1	1	1
A_Or_PQ1	4.70E-07	1	1.40E-06	3.90E-09	1	1	1
A_Or_PQ2	9.70E-08	1	2.00E-07	5.80E-10	1	1	1
A_Or_PQ4	3.30E-05	1	0.00011	3.10E-07	1	1	1
A_Or_QUA	0.00228	1	0.00844	2.10E-05	1	0.07665	1
A_Or_SB1	2.60E-08	1	4.50E-08	1.40E-10	1	1	1
A_Or_SB2	1.80E-06	1	5.80E-06	1.60E-08	1	1	1
A_Or_SBtr	5.00E-05	1	0.00018	5.10E-07	1	1	1
A_Or_Tof	9.20E-09	1	1.10E-08	4.20E-11	1	1	1
A_Sp_Abr0	<2e-16	0.00166	<2e-16	<2e-16	4.40E-07	0.00902	0.00079
A_Sp_Abr1	1	1	1	1	0.00062	8.00E-11	2.10E-08
M_Fr_Aude	6.50E-08	0.00088	2.60E-13	0.00037	<2e-16	<2e-16	<2e-16
M_Fr_Caen	2.70E-08	0.00058	8.30E-14	0.00018	<2e-16	<2e-16	<2e-16
M_Fr_Cal	3.40E-09	0.00017	1.00E-14	2.90E-05	<2e-16	<2e-16	<2e-16
M_Fr_Cant	0.32856	0.93179	0.00092	1	<2e-16	<2e-16	<2e-16
M_Fr_Cdo	0.00766	0.13569	4.80E-06	1	<2e-16	<2e-16	<2e-16
M_Fr_Hsa	0.09339	0.40886	0.00025	1	<2e-16	<2e-16	<2e-16
M_Fr_IDF	2.60E-05	0.00649	4.10E-09	0.02017	<2e-16	<2e-16	<2e-16
M_Fr_Lat	0.93915	1	0.01282	1	2.70E-16	<2e-16	<2e-16
M_Fr_Mor	0.00446	0.0613	7.60E-06	0.7186	<2e-16	<2e-16	<2e-16
M_Fr_Noi	1	1	0.30202	1	<2e-16	<2e-16	<2e-16
M_Fr_SM	1	1	0.06039	1	6.70E-13	<2e-16	<2e-16
M_Fr_Ven	0.07272	0.35906	0.00017	1	<2e-16	<2e-16	<2e-16
M_Fr_Vie	0.00049	0.01952	4.00E-07	0.13902	<2e-16	<2e-16	<2e-16
M_Fr_Yeu	1	1	1	1	1	8.30E-06	0.00065
M_Gr_Sax	0.00023	0.02489	3.80E-08	0.1317	<2e-16	<2e-16	<2e-16
M_Gr_Wol	1	1	0.01077	1	<2e-16	<2e-16	<2e-16
M_Guer	0.00501	1	0.019	4.70E-05	1	0.00684	1
M_Hung	0.21986	0.72068	0.0006	1	<2e-16	<2e-16	<2e-16
M_Ital	1.30E-05	0.00068	3.10E-08	0.00296	<2e-16	<2e-16	<2e-16
M_Or_Bur	1	1	1	1	1	1	1
M_Or_Main	0.03126	1	0.13042	0.00034	1	3.50E-05	0.0346
M_Or_Rou	0.261	1	1	0.00744	1	0.95658	1
M_Or_San	1	1	1	0.03526	1	6.10E-05	0.01595
M_Or_Sha	1	1	1	1	1	1	1
M_Or_Sro	1	1	1	0.03844	1	0.00039	0.05348
M_Or_Wes	1	1	1	1	0.2433	7.70E-12	1.70E-07
M_Spain	1	1	1	1	4.90E-05	9.00E-16	9.70E-12
M_Yug	0.31294	0.94659	0.00076	1	<2e-16	<2e-16	<2e-16

	A_Or_LoN	A_Or_PC	A_Or_PQ1	A_Or_PQ2	A_Or_PQ4	A_Or_QUA	A_Or_SB1
A_Bel_Taw	-	-	-	-	-	-	-
A_Fr_Abeu	-	-	-	-	-	-	-
A_Fr_MG	-	-	-	-	-	-	-
A_Or_EB	-	-	-	-	-	-	-
A_Or_How	-	-	-	-	-	-	-
A_Or_HPW	-	-	-	-	-	-	-
A_Or_LoN	-	-	-	-	-	-	-
A_Or_PC	1	-	-	-	-	-	-
A_Or_PQ1	1	1	-	-	-	-	-
A_Or_PQ2	1	1	1	-	-	-	-
A_Or_PQ4	1	1	1	1	-	-	-
A_Or_QUA	1	1	1	1	1	-	-
A_Or_SB1	1	1	1	1	1	1	-
A_Or_SB2	1	1	1	1	1	1	1
A_Or_SBtr	1	1	1	1	1	1	1
A_Or_Tof	1	1	1	1	1	0.82906	1
A_Sp_Abr0	0.0002	0.00035	0.14407	0.01124	0.00039	8.00E-10	0.00977
A_Sp_Abr1	4.40E-08	3.40E-09	7.50E-08	1.60E-08	5.40E-06	0.00036	4.40E-09
M_Fr_Aude	<2e-16	<2e-16	<2e-16	<2e-16	<2e-16	<2e-16	<2e-16
M_Fr_Caen	<2e-16	<2e-16	<2e-16	<2e-16	<2e-16	<2e-16	<2e-16
M_Fr_Cal	<2e-16	<2e-16	<2e-16	<2e-16	<2e-16	<2e-16	<2e-16
M_Fr_Cant	<2e-16	<2e-16	<2e-16	<2e-16	<2e-16	<2e-16	<2e-16
M_Fr_Cdo	<2e-16	<2e-16	<2e-16	<2e-16	<2e-16	<2e-16	<2e-16
M_Fr_Hsa	<2e-16	<2e-16	<2e-16	<2e-16	<2e-16	<2e-16	<2e-16
M_Fr_IDF	<2e-16	<2e-16	<2e-16	<2e-16	<2e-16	<2e-16	<2e-16
M_Fr_Lat	<2e-16	<2e-16	<2e-16	<2e-16	<2e-16	<2e-16	<2e-16
M_Fr_Mor	<2e-16	<2e-16	<2e-16	<2e-16	<2e-16	<2e-16	<2e-16
M_Fr_Noi	<2e-16	<2e-16	<2e-16	<2e-16	<2e-16	<2e-16	<2e-16
M_Fr_SM	<2e-16	<2e-16	<2e-16	<2e-16	9.10E-16	2.00E-14	<2e-16
M_Fr_Ven	<2e-16	<2e-16	<2e-16	<2e-16	<2e-16	<2e-16	<2e-16
M_Fr_Vie	<2e-16	<2e-16	<2e-16	<2e-16	<2e-16	<2e-16	<2e-16
M_Fr_Yeu	0.0013	0.0002	0.00075	0.00037	0.03219	1	0.00015
M_Gr_Sax	<2e-16	<2e-16	<2e-16	<2e-16	<2e-16	<2e-16	<2e-16
M_Gr_Wol	<2e-16	<2e-16	<2e-16	<2e-16	<2e-16	<2e-16	<2e-16
M_Guer	1	0.2917	1	0.6005	1	1	0.23994

M_Hung	<2e-16	<2e-16	<2e-16	<2e-16	<2e-16	<2e-16	<2e-16
M_Ital	<2e-16	<2e-16	<2e-16	<2e-16	<2e-16	<2e-16	<2e-16
M_Or_Bur	1	1	1	1	1	1	1
M_Or_Main	0.07714	0.00372	0.08219	0.02491	1	1	0.00653
M_Or_Rou	1	1	1	1	1	1	1
M_Or_San	0.03415	0.00304	0.02586	0.01	1	1	0.00327
M_Or_Sha	1	1	1	1	1	1	1
M_Or_Sro	0.11082	0.01387	0.06111	0.03054	1	1	0.01171
M_Or_Wes	3.50E-07	2.40E-09	8.60E-06	3.40E-07	0.001	0.07947	3.40E-08
M_Spain	2.00E-11	1.80E-13	9.40E-10	2.20E-11	1.10E-07	5.80E-06	2.10E-12
M_Yug	<2e-16	<2e-16	<2e-16	<2e-16	<2e-16	<2e-16	<2e-16
	A_Or_SB2	A_Or_SBtr	A_Or_Tof	A_Sp_Abr0	A_Sp_Abr1	M_Fr_Aude	M_Fr_Caen
A_Bel_Taw	-	-	-	-	-	-	-
A_Fr_Abeu	-	-	-	-	-	-	-
A_Fr_MG	-	-	-	-	-	-	-
A_Or_EB	-	-	-	-	-	-	-
A_Or_How	-	-	-	-	-	-	-
A_Or_HPW	-	-	-	-	-	-	-
A_Or_LoN	-	-	-	-	-	-	-
A_Or_PC	-	-	-	-	-	-	-
A_Or_PQ1	-	-	-	-	-	-	-
A_Or_PQ2	-	-	-	-	-	-	-
A_Or_PQ4	-	-	-	-	-	-	-
A_Or_QUA	-	-	-	-	-	-	-
A_Or_SB1	-	-	-	-	-	-	-
A_Or_SB2	-	-	-	-	-	-	-
A_Or_SBtr	1	-	-	-	-	-	-
A_Or_Tof	1	1	-	-	-	-	-
A_Sp_Abr0	0.05717	0.00062	0.00273	-	-	-	-
A_Sp_Abr1	3.00E-07	8.20E-06	1.60E-09	<2e-16	-	-	-
M_Fr_Aude	<2e-16	<2e-16	<2e-16	<2e-16	2.20E-05	-	-
M_Fr_Caen	<2e-16	<2e-16	<2e-16	<2e-16	1.00E-05	1	-
M_Fr_Cal	<2e-16	<2e-16	<2e-16	<2e-16	1.50E-06	1	1
M_Fr_Cant	<2e-16	<2e-16	<2e-16	<2e-16	1	0.01076	0.00413
M_Fr_Cdo	<2e-16	<2e-16	<2e-16	<2e-16	0.25645	1	1
M_Fr_Hsa	<2e-16	<2e-16	<2e-16	<2e-16	1	0.9766	0.4765
M_Fr_IDF	<2e-16	<2e-16	<2e-16	<2e-16	0.00203	1	1
M_Fr_Lat	<2e-16	<2e-16	<2e-16	<2e-16	1	1	0.67256
M_Fr_Mor	<2e-16	<2e-16	<2e-16	<2e-16	0.11678	1	1
M_Fr_Noi	<2e-16	<2e-16	<2e-16	<2e-16	1	2.00E-07	5.80E-08
M_Fr_SM	<2e-16	2.60E-15	<2e-16	<2e-16	1	1	1
M_Fr_Ven	<2e-16	<2e-16	<2e-16	<2e-16	1	1	0.65567
M_Fr_Vie	<2e-16	<2e-16	<2e-16	<2e-16	0.01882	1	1
M_Fr_Yeu	0.00229	0.04051	8.70E-05	2.50E-13	1	9.90E-13	3.50E-13
M_Gr_Sax	<2e-16	<2e-16	<2e-16	<2e-16	0.01478	1	1
M_Gr_Wol	<2e-16	<2e-16	<2e-16	<2e-16	1	0.05728	0.0257
M_Guer	1	1	0.12311	4.80E-11	0.00079	<2e-16	<2e-16
M_Hung	<2e-16	<2e-16	<2e-16	<2e-16	1	0.0544	0.02262
M_Ital	<2e-16	<2e-16	<2e-16	<2e-16	0.00041	1	1
M_Or_Bur	1	1	1	0.10349	1	3.90E-06	2.40E-06
M_Or_Main	0.27882	1	0.00179	2.70E-13	0.00504	<2e-16	<2e-16
M_Or_Rou	1	1	1	1.80E-07	0.05437	<2e-16	<2e-16
M_Or_San	0.08343	1	0.00134	5.30E-13	0.27732	<2e-16	<2e-16
M_Or_Sha	1	1	1	0.00016	1	2.90E-07	1.60E-07
M_Or_Sro	0.18196	1	0.00597	4.40E-12	0.28701	<2e-16	<2e-16
M_Or_Wes	4.50E-05	0.00186	2.10E-09	<2e-16	1	<2e-16	<2e-16
M_Spain	5.60E-09	2.50E-07	1.40E-13	<2e-16	1	1.50E-13	4.50E-14
M_Yug	<2e-16	<2e-16	<2e-16	<2e-16	1	0.00498	0.00183
	M_Fr_Cal	M_Fr_Cant	M_Fr_Cdo	M_Fr_Hsa	M_Fr_IDF	M_Fr_Lat	M_Fr_Mor
A_Bel_Taw	-	-	-	-	-	-	-
A_Fr_Abeu	-	-	-	-	-	-	-
A_Fr_MG	-	-	-	-	-	-	-
A_Or_EB	-	-	-	-	-	-	-
A_Or_How	-	-	-	-	-	-	-
A_Or_HPW	-	-	-	-	-	-	-
A_Or_LoN	-	-	-	-	-	-	-
A_Or_PC	-	-	-	-	-	-	-
A_Or_PQ1	-	-	-	-	-	-	-
A_Or_PQ2	-	-	-	-	-	-	-
A_Or_PQ4	-	-	-	-	-	-	-
A_Or_QUA	-	-	-	-	-	-	-
A_Or_SB1	-	-	-	-	-	-	-
A_Or_SB2	-	-	-	-	-	-	-
A_Or_SBtr	-	-	-	-	-	-	-
A_Or_Tof	-	-	-	-	-	-	-
A_Sp_Abr0	-	-	-	-	-	-	-
A_Sp_Abr1	-	-	-	-	-	-	-
M_Fr_Aude	-	-	-	-	-	-	-
M_Fr_Caen	-	-	-	-	-	-	-
M_Fr_Cal	-	-	-	-	-	-	-
M_Fr_Cant	0.00046	-	-	-	-	-	-
M_Fr_Cdo	0.31418	1	-	-	-	-	-
M_Fr_Hsa	0.07046	1	1	-	-	-	-

M_Fr_IDF	1	1	1	1	-	-	-
M_Fr_Lat	0.12162	1	1	1	1	-	-
M_Fr_Mor	1	1	1	1	1	1	-
M_Fr_Noi	6.60E-09	1	1	1	0.00253	1	0.92011
M_Fr_SM	1	1	1	1	1	1	1
M_Fr_Ven	0.09898	1	1	1	1	1	1
M_Fr_Vie	1	1	1	1	1	1	1
M_Fr_Yeu	4.30E-14	0.0003	2.30E-06	8.00E-05	3.10E-09	0.0031	2.70E-06
M_Gr_Sax	1	1	1	1	1	1	1
M_Gr_Wol	0.0034	1	1	1	1	1	1
M_Guer	<2e-16	<2e-16	<2e-16	<2e-16	<2e-16	<2e-16	<2e-16
M_Hung	0.00269	1	1	1	1	1	1
M_Ital	1	0.29608	1	1	1	1	1
M_Or_Bur	6.10E-07	0.01739	0.00165	0.00667	4.60E-05	0.02588	0.00073
M_Or_Main	<2e-16	<2e-16	<2e-16	<2e-16	<2e-16	<2e-16	<2e-16
M_Or_Rou	<2e-16	2.60E-14	<2e-16	1.60E-14	<2e-16	6.40E-11	7.00E-16
M_Or_San	<2e-16	<2e-16	<2e-16	<2e-16	<2e-16	1.10E-11	<2e-16
M_Or_Sha	3.20E-08	0.01177	0.00066	0.00395	8.80E-06	0.02317	0.0003
M_Or_Sro	<2e-16	3.60E-16	<2e-16	6.00E-16	<2e-16	3.80E-11	<2e-16
M_Or_Wes	<2e-16	1.00E-15	<2e-16	4.90E-15	<2e-16	1.80E-09	8.30E-16
M_Spain	5.90E-15	0.00458	1.80E-05	0.00122	1.10E-08	0.06896	3.50E-05
M_Yug	0.0002	1	1	1	1	1	1
	M_Fr_Noi	M_Fr_SM	M_Fr_Ven	M_Fr_Vie	M_Fr_Yeu	M_Gr_Sax	M_Gr_Wol
A_Bel_Taw	-	-	-	-	-	-	-
A_Fr_Abeu	-	-	-	-	-	-	-
A_Fr_MG	-	-	-	-	-	-	-
A_Or_EB	-	-	-	-	-	-	-
A_Or_How	-	-	-	-	-	-	-
A_Or_HPW	-	-	-	-	-	-	-
A_Or_LoN	-	-	-	-	-	-	-
A_Or_PC	-	-	-	-	-	-	-
A_Or_PQ1	-	-	-	-	-	-	-
A_Or_PQ2	-	-	-	-	-	-	-
A_Or_PQ4	-	-	-	-	-	-	-
A_Or_QUA	-	-	-	-	-	-	-
A_Or_SB1	-	-	-	-	-	-	-
A_Or_SB2	-	-	-	-	-	-	-
A_Or_SBtr	-	-	-	-	-	-	-
A_Or_Tof	-	-	-	-	-	-	-
A_Sp_Abr0	-	-	-	-	-	-	-
A_Sp_Abr1	-	-	-	-	-	-	-
M_Fr_Aude	-	-	-	-	-	-	-
M_Fr_Caen	-	-	-	-	-	-	-
M_Fr_Cal	-	-	-	-	-	-	-
M_Fr_Cant	-	-	-	-	-	-	-
M_Fr_Cdo	-	-	-	-	-	-	-
M_Fr_Hsa	-	-	-	-	-	-	-
M_Fr_IDF	-	-	-	-	-	-	-
M_Fr_Lat	-	-	-	-	-	-	-
M_Fr_Mor	-	-	-	-	-	-	-
M_Fr_Noi	-	-	-	-	-	-	-
M_Fr_SM	1	-	-	-	-	-	-
M_Fr_Ven	1	1	-	-	-	-	-
M_Fr_Vie	0.08952	1	1	-	-	-	-
M_Fr_Yeu	0.06992	0.014	5.80E-05	1.70E-07	-	-	-
M_Gr_Sax	0.02634	1	1	1	2.90E-08	-	-
M_Gr_Wol	1	1	1	1	0.00274	1	-
M_Guer	<2e-16	4.10E-14	<2e-16	<2e-16	1	<2e-16	<2e-16
M_Hung	1	1	1	1	0.00019	1	1
M_Ital	0.00225	1	1	1	1.00E-08	1	0.38208
M_Or_Bur	0.26237	0.03824	0.00568	0.00019	1	0.00021	0.03468
M_Or_Main	<2e-16	4.90E-13	<2e-16	<2e-16	1	<2e-16	<2e-16
M_Or_Rou	2.60E-10	6.20E-09	9.20E-15	<2e-16	1	<2e-16	8.80E-12
M_Or_San	3.20E-12	6.60E-09	<2e-16	<2e-16	1	<2e-16	3.00E-13
M_Or_Sha	0.31002	0.04174	0.00325	5.70E-05	1	5.20E-05	0.02998
M_Or_Sro	2.70E-11	1.20E-08	3.20E-16	<2e-16	1	<2e-16	1.70E-12
M_Or_Wes	3.90E-10	7.40E-07	2.40E-15	<2e-16	1	<2e-16	4.30E-11
M_Spain	1	0.29818	0.00084	1.60E-06	1	1.00E-07	0.05885
M_Yug	1	1	1	1	0.00026	1	1
	M_Guer	M_Hung	M_Ital	M_Or_Bur	M_Or_Main	M_Or_Rou	M_Or_San
A_Bel_Taw	-	-	-	-	-	-	-
A_Fr_Abeu	-	-	-	-	-	-	-
A_Fr_MG	-	-	-	-	-	-	-
A_Or_EB	-	-	-	-	-	-	-
A_Or_How	-	-	-	-	-	-	-
A_Or_HPW	-	-	-	-	-	-	-
A_Or_LoN	-	-	-	-	-	-	-
A_Or_PC	-	-	-	-	-	-	-
A_Or_PQ1	-	-	-	-	-	-	-
A_Or_PQ2	-	-	-	-	-	-	-
A_Or_PQ4	-	-	-	-	-	-	-
A_Or_QUA	-	-	-	-	-	-	-
A_Or_SB1	-	-	-	-	-	-	-

A_Or_SB2	-	-	-	-	-	-	-
A_Or_SBtr	-	-	-	-	-	-	-
A_Or_Tof	-	-	-	-	-	-	-
A_Sp_Abr0	-	-	-	-	-	-	-
A_Sp_Abr1	-	-	-	-	-	-	-
M_Fr_Aude	-	-	-	-	-	-	-
M_Fr_Caen	-	-	-	-	-	-	-
M_Fr_Cal	-	-	-	-	-	-	-
M_Fr_Cant	-	-	-	-	-	-	-
M_Fr_Cdo	-	-	-	-	-	-	-
M_Fr_Hsa	-	-	-	-	-	-	-
M_Fr_IDF	-	-	-	-	-	-	-
M_Fr_Lat	-	-	-	-	-	-	-
M_Fr_Mor	-	-	-	-	-	-	-
M_Fr_Noi	-	-	-	-	-	-	-
M_Fr_SM	-	-	-	-	-	-	-
M_Fr_Ven	-	-	-	-	-	-	-
M_Fr_Vie	-	-	-	-	-	-	-
M_Fr_Yeu	-	-	-	-	-	-	-
M_Gr_Sax	-	-	-	-	-	-	-
M_Gr_Wol	-	-	-	-	-	-	-
M_Guer	-	-	-	-	-	-	-
M_Hung	<2e-16	-	-	-	-	-	-
M_Ital	<2e-16	0.58053	-	-	-	-	-
M_Or_Bur	1	0.01286	5.30E-06	-	-	-	-
M_Or_Main	1	<2e-16	<2e-16	1	-	-	-
M_Or_Rou	1	2.10E-14	<2e-16	1	1	-	-
M_Or_San	1	<2e-16	<2e-16	1	1	1	-
M_Or_Sha	1	0.00833	1.40E-06	1	1	1	1
M_Or_Sro	1	4.10E-16	<2e-16	1	1	1	1
M_Or_Wes	0.19131	1.70E-15	1.50E-15	1	1	1	1
M_Spain	1.30E-05	0.00296	1.50E-07	1	0.00015	0.03096	0.12733
M_Yug	<2e-16	1	0.235	0.01746	<2e-16	1.30E-14	<2e-16
	M_Or_Sha	M_Or_Sro	M_Or_Wes	M_Spain			
A_Bel_Taw	-	-	-	-			
A_Fr_Abeu	-	-	-	-			
A_Fr_MG	-	-	-	-			
A_Or_EB	-	-	-	-			
A_Or_How	-	-	-	-			
A_Or_HPW	-	-	-	-			
A_Or_LoN	-	-	-	-			
A_Or_PC	-	-	-	-			
A_Or_PQ1	-	-	-	-			
A_Or_PQ2	-	-	-	-			
A_Or_PQ4	-	-	-	-			
A_Or_QUA	-	-	-	-			
A_Or_SB1	-	-	-	-			
A_Or_SB2	-	-	-	-			
A_Or_SBtr	-	-	-	-			
A_Or_Tof	-	-	-	-			
A_Sp_Abr0	-	-	-	-			
A_Sp_Abr1	-	-	-	-			
M_Fr_Aude	-	-	-	-			
M_Fr_Caen	-	-	-	-			
M_Fr_Cal	-	-	-	-			
M_Fr_Cant	-	-	-	-			
M_Fr_Cdo	-	-	-	-			
M_Fr_Hsa	-	-	-	-			
M_Fr_IDF	-	-	-	-			
M_Fr_Lat	-	-	-	-			
M_Fr_Mor	-	-	-	-			
M_Fr_Noi	-	-	-	-			
M_Fr_SM	-	-	-	-			
M_Fr_Ven	-	-	-	-			
M_Fr_Vie	-	-	-	-			
M_Fr_Yeu	-	-	-	-			
M_Gr_Sax	-	-	-	-			
M_Gr_Wol	-	-	-	-			
M_Guer	-	-	-	-			
M_Hung	-	-	-	-			
M_Ital	-	-	-	-			
M_Or_Bur	-	-	-	-			
M_Or_Main	-	-	-	-			
M_Or_Rou	-	-	-	-			
M_Or_San	-	-	-	-			
M_Or_Sha	-	-	-	-			
M_Or_Sro	1	-	-	-			
M_Or_Wes	1	1	-	-			
M_Spain	1	0.15363	1	-			
M_Yug	0.01161	<2e-16	<2e-16	0.00365			
